# Microbial diagnostic features identified across populations possess potential antitumor properties in breast cancer

**DOI:** 10.1128/msystems.00271-25

**Published:** 2025-06-23

**Authors:** Min Zhou, Qinghua Wang, Rui Yang, Zhen Zong, Bowen Huang, Junjie Chen, Xin Bao, Su Li, Lanfeng Shen, Jianfeng Dong, Xiaoqian Zhao, Yu Chen, Daozhen Chen

**Affiliations:** 1 Department of Breast Surgery, Affiliated Women’s Hospital of Jiangnan University, Wuxi Maternity and Child Health Care Hospital 680653, Wuxi, Jiangsu, China; 2 Research Institute for Reproductive Health and Genetic Diseases, Affiliated Women’s Hospital of Jiangnan University, Wuxi Maternity and Child Health Care Hospital 680653 , Wuxi, Jiangsu, China; 3 Department of Child Health Care, Qingmingqiao Street Community Health Service Center, Wuxi, China; 4 Wuxi Food Safety Inspection and Test Center, Wuxi, China; 5 School of Life Sciences, Xiamen University 199198 , Xiamen, Fujian, China; 6 Department of Thyroid and Breast Surgery, The Affiliated Yixing Hospital of Jiangsu University, Wuxi, China; 7 Department of Oncology, Affiliated Hospital of Jiangnan University 199193, Wuxi, Jiangsu, China; 8 Department of Laboratory Medicine, The Affiliated Wuxi No. 2 People’s Hospital of Nanjing Medical University, Jiangnan University Medical Center, Wuxi, China; 9 Department of Pathology, Wuxi Maternity and Child Health Care Hospital, Affiliated Women’s Hospital of Jiangnan University, Wuxi, China; 10 Wuxi Higher Health Vocational Technology School, Wuxi, China; Northern Arizona University, Flagstaff, Arizona, USA

**Keywords:** breast cancer, random forest, specific microbial feature, *Cutibacterium acnes*, culturomics, antitumor properties, propanoate metabolism

## Abstract

The consistency of the associations between the breast microbiome and breast cancer (BC) across various studies remains uncertain. Publicly accessible data sets from five BC studies, comprising 16S rRNA gene sequencing data from 161 BC tissues (BC_tissue), 195 BC adjacent non-cancerous tissues (BC_adjacent), and 451 normal breast tissues (normal_tissue), were retrieved from the European Nucleotide Archive. Overall, the microbial composition across the three breast tissue statuses was predominantly characterized by the phyla *Proteobacteria* and *Firmicutes*, a distribution likely attributable to the fatty acid-rich environment of the breast tissue. Comparative analysis revealed that the relative abundances of the genera *Cutibacterium* and *Burkholderia* were significantly increased in both BC_adjacent and normal_tissue compared to BC_tissue. This observation suggested a potential anticancer effect associated with these genera. Our analysis revealed a significant reduction in the abundance of *Cutibacterium* and *Cutibacterium acnes* in BC tissues, which served as specific diagnostic features for BC. This finding was corroborated by our in-house data set (*n* = 28), which yielded similar conclusions. Subsequent *in vitro* and *in vivo* experiments verified the potential antitumor effects of *C. acnes* supernatant in BC. In conclusion, our study highlighted the predictive capacity of microbial biomarkers in the onset of BC. Notably, specific bacterial species within the breast microbiome, such as *Cutibacterium* and *C. acnes*, exhibited potential as diagnostic markers for BC and may contribute significantly to antitumor activity. Nevertheless, the molecular mechanisms governing their interactions with cancer cells are not yet fully understood, necessitating further research to investigate their viability as targets for tumor prevention.

**IMPORTANCE:**

Although a growing number of studies have highlighted the significant role of microorganisms in BC, there is a lack of consensus regarding the specific microbial genera consistently associated with breast cancer. While some studies have identified certain genera in the breast cancer environment, the results are often inconsistent and influenced by factors such as study design, population, or methodologies used. Through a comprehensive analysis of five publicly available breast cancer studies, along with validation from an in-house cohort, we found a significantly reduced abundance of *Cutibacterium* and *C. acnes* in BC tissues. *In vivo* and *in vitro* experiments demonstrated the antitumor effects of *C. acnes* in BC. Understanding the antitumor mechanisms of *C. acnes* in BC may provide potential avenues for developing novel therapeutic strategies for this disease.

## INTRODUCTION

Breast cancer (BC) continues to be the most prevalent malignancy among women and constitutes the primary cause of cancer-related mortality in this population (1). It represents a critical global public health priority. Despite significant advancements in diagnostic techniques and therapeutic interventions that have markedly enhanced survival rates for BC patients, substantial gaps persist in our understanding of the disease’s underlying biological mechanisms.

Similar to other forms of cancer, the etiology of BC remains uncertain due to its complex multifactorial nature, likely arising from an interplay of genetic and non-hereditary factors. This complexity significantly complicates the treatment and management of the disease. In addition to genetic predispositions, such as mutations in the *BRCA1* and *BRCA2* genes, which are known to increase the risk of BC, numerous environmental and lifestyle factors are also strongly associated with its development (2). The etiology of approximately 80%–85% of BCs remains unidentified, categorizing them as sporadic (3). In this context, environmental and lifestyle factors may also play a role in influencing cancer risk in both familial and sporadic BCs (4). Nevertheless, the majority of factors contributing to breast cancer remain inadequately understood, thereby limiting the efficacy of prevention and treatment strategies (3, 4).

Microorganisms interact with host metabolism and modulate the local microenvironment, thereby influencing tissue homeostasis. Several mechanisms have been proposed to elucidate the connection between the microbiome and alterations in the tissue microenvironment, including the regulation of innate and adaptive immune responses (5), the induction of genomic instability and DNA damage (6), and metabolic activities that produce metabolites such as short-chain fatty acids (SCFAs), amino acids, or secondary bile acids (7, 8). These metabolites may either promote tumorigenesis or inhibit the proliferation of pathogenic bacteria. Consequently, bacterial communities residing within a host can be regarded as an additional environmental factor that may both influence and be influenced by the process of carcinogenesis. Recent research has revealed the presence of microbiota in internal organs previously thought to be sterile, such as the lung, pancreas, and breast (9). Concerning the origin of the microbiota within the breast, various hypotheses have been proposed and examined. These included transmission through the skin via the nipple-areolar orifices, nipple-oral contact during lactation and/or sexual activity, and more recently, translocation from the gastrointestinal tract (10, 11). Contemporary research indicated that variations in bacterial composition within human mammary tissue were linked to BC (2, 4, 5, 9–11). In recent years, researchers have made significant strides in elucidating the role of resident microbiota in the development of BC (12). It was suggested that organ-specific microbiota may influence the tissue microenvironment, tumor development, and therapeutic resistance (13). The microbiota composition in tissue adjacent to malignant breast tumors exhibited a distinct bacterial signature compared to that of tumor tissue, indicating potential oncogenic or antitumor roles for specific bacterial taxa (14).

Several independent studies have characterized the human breast microbiota in BC tissues (BC_tissue) from patients, as well as in BC adjacent non-cancerous tissues (BC_adjacent) and normal breast tissues (normal_tissue), utilizing 16S rRNA sequencing. However, the reproducibility and predictive accuracy of the microbial signatures identified independently in each study remain uncertain. Consequently, there is a necessity to conduct a comprehensive and multicohort analysis to furnish an unbiased and well-powered evaluation of the relationship between BC_tissue and BC_adjacent or normal_tissue. In this study, we integrated and reanalyzed raw 16S rRNA gene sequence data from five independent studies encompassing 877 breast tissue samples (15–19). The robustness of the associations between the microbiome and disease status was evaluated through multicohort comparisons, and bacterial biomarkers for the classification of different disease groups were identified and validated.

## MATERIALS AND METHODS

### Study selection

Due to the limited number of studies on the BC microbiome and the unavailability of publicly accessible 16S rRNA data from other relevant studies, only five datasets were included in this analysis. These data sets encompassed 16S rRNA gene sequencing data from 231 BC tissues, 195 BC adjacent tissues, and 451 normal breast tissues, which were obtained from the European Nucleotide Archive (ENA). The demographic and clinical characteristics of the included subjects are presented in Table 1. For the study by Esposito et al.(Esposito_2022) (15), raw data comprising 34 BC_tissues and 34 BC_adjacent tissues from Italian patients were retrieved under accession number PRJNA759366. For the study by Hoskinson et al. (Hoskinson_2022) (16), raw data of 46 BC_ tissues, 49 BC_adjacent tissues, and 46 normal_tissues from USA were downloaded with identifier PRJNA723425. We fetched the raw data of 51 BC_ tissues and 52 BC_adjacent tissues from Moroccan patients with BC for the study by Kartti et al. (Kartti_2023) from ENA under accession number PRJNA926328 (17). We also included all the 70 BC_ tissues collected in China from the study by Liu et al. (Liu_2023), and the raw sequence data were available in the ENA under accession number PRJNA769523 (18). Finally, for the study by German et al. (German_2023) (19), 30 BC_ tissues, 60 BC_adjacent tissues, and 402 normal_tissues of American patients were included with identifier PRJNA867176.

**TABLE 1:** Summary of sample characteristics of data sets included in this study

StudyID	Nation	Sample size^*a*^				Sequencing platform	Sequencing region	NCBI BioProject ID
		BC_tissue	BC_adjacent	Normal_tissue	Total			
Esposito_2022	Italy	34	34	–	68	Illumina MiSeq	V4–V6	PRJNA759366
Hoskinson_2022	USA	46	49	49	144	Illumina MiSeq	V3–V4	PRJNA723425
Kartti_2023	Morocco	51	52	–	103	Illumina MiSeq	V3–V4	PRJNA926328
Liu_2023	China	70	–	–	70	Illumina NovaSeq 6000	V3–V4	PRJNA769523
German_2023	USA	30	60	402	492	Illumina MiSeq	V1V2, V2V3, V3V4, V4V5, V5V7, and V7V9	PRJNA867176

^*a*^ The quantity of samples downloaded, as recorded in the ENA database.

### Sequencing data preprocessing

The 16S rRNA sequencing data sets acquired from the five included BC studies underwent quality filtering utilizing FastP software (version 0.18.0) (20). This process involved the elimination of reads containing over 10% of unknown nucleotides and those with more than 50% of bases exhibiting a quality score below 20. Subsequently, paired reads were merged into raw tags using FLASH software (version 1.2.11) (21), adhering to a minimum overlap requirement of 10 bp and a mismatch error rate threshold of 2%. To ensure data integrity, noisy sequences within the raw tags were filtered according to specific criteria, resulting in the generation of high-quality clean tags (22). The filtering criteria were defined as follows: first, raw tags were truncated at the initial occurrence of a low-quality base site, where the number of consecutive bases with a quality value at or below the default threshold of 3 reaches the specified length, which was set to 3 bp; second, tags were filtered out if the proportion of continuous high-quality bases was less than 75% of the total tag length. The clean tags were clustered into operational taxonomic units with a minimum similarity threshold of 97%, utilizing the UPARSE pipeline (version 9.2.64) (23). Chimeric tags were identified and eliminated using the UCHIME algorithm (24), resulting in the acquisition of effective tags for subsequent analysis. Within each cluster, the tag sequence exhibiting the highest abundance was designated as the representative sequence.

### Taxonomy annotation

The resulting sequences were taxonomically classified utilizing the Greengenes2 database (version 2022.10) in conjunction with the BLAST taxonomy classifier (version 2.6.0) under default settings (25). The abundance statistics for each taxonomic group were visualized using Krona (version 2.6) (26). Circular layout representations of species abundance were generated using CIRCOS (version 0.69-3) (27). The richness and abundance of species within each sample, referred to as alpha diversity, were estimated using Shannon’s and Simpson’s indices (28). The dissimilarity of microbial communities among samples (beta diversity) was quantified using the Bray-Curtis distance and subsequently visualized through principal coordinate analysis (PCoA) (29). To compare the community dissimilarity across sample groups, permutational multivariate analysis of variance (PERMANOVA) was employed, utilizing the Bray-Curtis distance with 1,000 permutations (30).

### Random forest-based machine learning and feature selection

Utilizing Wilcoxon’s rank-sum test, we identified differentially abundant features between two groups (BC_tissue vs. BC_adjacent, or BC_tissue vs. normal_tissue) on a per genera/species basis. The *P* values were adjusted using the conservative Bonferroni correction method. For the selection of “important features,” a criterion was applied by excluding features with *P* values greater than 0.05. Random forest (RF) models were constructed utilizing estimator trees, with each tree incorporating 10% of the total available features. An iterative feature elimination (IFE) procedure was then employed to filter features and enhance the performance of subsequent RF models. The most significant features from the highest-performing model were designated as “specific features.” We utilized abundance profiles encompassing the most prevalent microbial genera and species to evaluate the generalizability of classifiers. These classifiers were trained using cross-validation on one study and subsequently assessed for their performance across different studies, a process referred to as study-to-study transfer of classifiers. Additionally, we evaluated whether incorporating data from all but one study in the model training process enhanced the predictive accuracy for the excluded study. This approach was commonly referred to as leave-one-study-out (LOSO) validation. The permutation-based importance, as implemented by the permutation importance function in the ELI5 Python package (https://eli5.readthedocs.io), was employed to calculate the feature importance for the models. Utilizing the specific microbiome features, we constructed RF classifiers within the scikit-learn (version 0.19.2) package, employing stratified 10-fold cross-validation to differentiate between patients with BC and those with BC_adjacent or normal tissues. The performance of the models was assessed through receiver operating characteristic curves and the calculation of the area under the curve (AUC). To estimate the probability of BC onsets, we devised a robust scoring mechanism for the RF classifier, utilizing microbial features as input variables. This scoring mechanism, referred to as the RF score, was computed for both the training and validation data sets using the predict_proba() function from the scikit-learn package (version 0.19.2). Notably, a higher RF score corresponded to a decreased likelihood of patients developing BC (31).

### Function prediction

Functional prediction was conducted utilizing PICRUSt2 (32). The predicted functional genes were subsequently classified into MetaCyc metabolic pathways and Kyoto Encyclopedia of Genes and Genomes pathways. To identify significantly altered pathways between different breast statuses, the linear discriminant analysis (LDA) effect size method was employed, applying a threshold LDA score greater than 2 and a *P* value less than 0.05 (33).

### Human breast sample collection and 16s rRNA sequencing

The in-house cohort consisted of patients with BC (BC_tissue, *n* = 10), normal tissue adjacent to BC (BC_adjacent, *n* = 10), and benign fibroadenoma (benign_tissue, *n* = 8) (Table S1). Breast tissue samples were obtained from women who satisfied the following criteria: they were not lactating or pregnant at the time of collection, had no prior history of breast disease or intestinal inflammatory disease, and had not taken antibiotics during the sampling period.

Each tissue core (around 0.1 g) was divided into pieces using a sterile scalpel, and microbial DNA was extracted utilizing the HiPure Bacterial DNA Kits (Magen, China) in accordance with the manufacturer’s protocols. The 16S rDNA target region of the ribosomal RNA gene was amplified via polymerase chain reaction (PCR) under the following conditions: initial denaturation at 95°C for 5 min, followed by 30 cycles of denaturation at 95°C for 1 min, annealing at 60°C for 1 min, and extension at 72°C for 1 min, with a final extension at 72°C for 7 min. The full-length primer sequences designed for amplifying the V3–V4 hypervariable region of the 16S rRNA gene (341F/806R) were as follows: forward: CCTACGGGNGGCWGCAG and reverse: GGACTACHVGGGTATCTAAT. A 50 µL reaction mixture was prepared, comprising 10 µL of 5 × Q5 Reaction Buffer, 10 µL of 5 × Q5 High GC Enhancer, 1.5 µL of 2.5 mM deoxynucleoside triphosphates, 1.5 µL of each primer (10 µM), 0.2 µL of Q5 High-Fidelity DNA Polymerase, and 50 ng of template DNA. All PCR reagents were sourced from New England Biolabs, USA. The resultant amplicons were assessed using 2% agarose gel electrophoresis and subsequently purified with AMPure XP Beads (Beckman Coulter, USA) in accordance with the manufacturer’s instructions. Sequencing libraries were then generated using the Illumina DNA Prep Kit (Illumina, USA) following the manufacturer’s recommendations. The quality of the library was evaluated using the ABI StepOnePlus Real-time PCR System (Life Technologies, USA). Subsequently, sequencing was performed on the NovaSeq 6000 platform, producing 2 × 250 bp paired-end reads. The raw sequencing reads have been deposited in the ENA under accession number PRJNA1113855. The processing of 16S rRNA sequencing data and the corresponding taxonomy annotation were conducted as previously described (refer to Materials and Methods on sequencing data preprocessing).

### Culturomics and *Cutibacterium acnes* identification

In order to isolate *C. acnes* with a higher probability, BC, together with adjacent tissues (around 0.1 g), was minced into small pieces and then homogenized with a glass homogenizer in 1 mL ice-cold Dulbecco’s modified Eagle medium (DMEM) (HyClone, USA) under sterile conditions. A 100 µL aliquot of the homogenate was then plated onto Columbia blood agar (Changde BKMAM Biotechnology Co., Ltd., China) and placed in an anaerobic pouch (Changde BKMAM Biotechnology Co., Ltd.). The plate was incubated at 37°C under anaerobic conditions. Bacterial colonies were collected on days 3–5. Colonies were picked and identified using matrix-assisted laser desorption/ionization time-of-flight (MALDI-TOF) mass spectrometry (MS) systems (Autof MS1000; Autobio Diagnostics, China). Colonies that initially identified as *C. acnes* were subjected to 16S rRNA gene sequencing with the primers 27F (AGAGTTTGATCMTGGCTCAG) and 1492R (GGTTACCTTGTTACGACTT) (Beijing Ruibo BioTech Co., Ltd., China). The sequencing results were analyzed utilizing the NCBI BLAST algorithm to conduct homologous sequence searches against type strains. If the 16S rRNA gene sequence exhibits a similarity of 98.65% or greater to the nearest type strain, the isolate may be considered a novel species (34). Colonies of *C. acnes* were meticulously isolated from agar plates prepared using the standard method and subsequently cultured under anaerobic conditions in brain heart infusion (BHI) medium (Changde BKMAM Biotechnology Co., Ltd.) at 37°C within an incubator. The bacterial cultures were grown until they reached an optical density at 600 nm of 1.0–1.2. Supernatants were then collected, filtered through 0.22 µm filters, and subsequently diluted with the medium.

### High-performance liquid chromatography-mass spectrometry

Lipidomic analysis of supernatants was performed using high-performance liquid chromatography-mass spectrometry (HPLC-MS) (Thermo Fisher Scientific, USA) with Agilent XDB-C18 analytical column (Agilent Technologies, USA). Three SCFAs (acetate, propionate, and butyrate) with higher levels than background signals were quantified. The offline mass spectrometry data were imported into Compound Discoverer (version 3.2) software (Thermo Fisher Scientific). This analytical process yielded a data matrix encompassing information such as metabolite peak areas and identification results.

### Cell lines and culture conditions

Human breast cancer cell lines MCF-7 and MDA-MB-231 (American Type Culture Collection) were maintained in DMEM supplemented with 10% fetal bovine serum (FBS), 4 mg/mL human insulin, and 1% penicillin-streptomycin. The cultures were incubated at 37°C in a humidified chamber containing 5% CO_2_. Upon reaching 80% confluence, the cells were subcultured into fresh medium.

### Clonogenicity assay

Cells in the logarithmic growth phase were digested with 0.25% trypsin and dissociated into individual cells. These cells were then suspended in DMEM medium supplemented with 10% FBS. A total of 500 cells were inoculated into each dish containing 10 mL of prewarmed culture solution at 37°C. The cultures were incubated for a duration of 2–3 weeks in a cell culture incubator maintained at 37°C with 5% CO_2_ and saturated humidity. This incubation was conducted either in the absence or presence of 20% *C*. *acnes* supernatant or 1 mM sodium propionate (SP) (Solarbio, China). When macroscopic clones appeared in the culture dish, the culture was terminated. To fix the cells, a 4% paraformaldehyde solution was added to a final volume of 5 mL and incubated for 15 min at room temperature. After fixation, the fixative was removed, and the cells were washed with phosphate-buffered saline (PBS). Giemsa staining solution was then added to the cells, and staining was performed for 10–30 min. Finally, the cells were washed and counted.

### Scratched assay

Cells were cultured in six-well plates until they reached 90% confluence. A scratch was introduced using a sterile 200 µL pipette tip on the serum-starved cells at 90% confluence. Subsequently, the cells were incubated in serum-free DMEM medium for 24 and 48 hours, either in the absence or presence of 20% *C*. *acnes* supernatant or 1 mM SP. During the initial 24 hours, some cells may remain suspended and fail to adhere to the surface of the plates, potentially affecting subsequent imaging. To address this, the medium was gently replaced with fresh serum-free DMEM medium after washing the cells with PBS. After this initial medium change, the cells were cultured in the same medium for the remainder of the experiment. Cell migration into the wound area was assessed microscopically at 0, 24, and 48 hours. The extent of cell migration was quantified by calculating the percentage of the remaining cell-free area relative to the initial scratched area.

### Invasion assays

Transwell assays were undertaken using 8 µm Transwell chambers (Corning, USA). Cells (1 × 10^5^) in 200 µL of FBS-free DMEM medium were added to the upper chambers precoated with Matrigel (Corning). DMEM supplemented with 20% FBS was introduced into the lower chambers, with conditions either in the absence or presence of 20% *C*. *acnes* supernatant or 1 mM SP. After incubation at 37°C for 24 hours, cells on the lower membrane surface were stained with crystal violet and counted.

### *In vivo* experiments

Female BALB/c nude mice aged 4–5 weeks were purchased from Shanghai Lingchang Biotechnology Co., Ltd., China. MDA-MB-231 cells (1 × 10^7^ cells/35 µL PBS/tumor) were mixed with matrigel (15 µL/tumor), and 50 µL of cell suspension was injected subcutaneously into the right armpits of nude mice. Once the tumor volume reached approximately 100 mm^3^, five mice were allocated to each group: the *C. acnes* group and the control group. In the *C. acnes* group, 50 µL of *C. acnes* culture supernatant was injected into the tumor once every 3 days for 2 weeks; in the control group, mice were injected with 50 µL BHI medium only. After 2 weeks, the tumors were harvested, weighed, and fixed with paraformaldehyde for immunohistochemical staining.

### Immunohistochemistry

Tissue sections were first obtained from paraffin-embedded mouse tumor xenografts after they were harvested. The paraffin embedding was performed following tumor collection. Afterward, the tissue sections were deparaffinized, rehydrated, and subjected to antigen retrieval in citrate buffer (pH 6.0) using a microwave. Endogenous peroxidase activity was blocked with 3% hydrogen peroxide for 10 min. Sections were incubated with the primary antibody against Ki-67 (1:150; Boster, China) at 4°C overnight, followed by detection using a biotinylated secondary antibody and streptavidin-horseradish peroxidase complex. Diaminobenzidine (Solarbio) was used as the chromogen, and sections were counterstained with hematoxylin. Images were captured under a light microscope.

### Statistical analysis

The data represent the mean ± SD unless otherwise indicated. Data were analyzed by two-tailed unpaired Student’s *t*-test between two groups and by one-way analysis of variance followed by Bonferroni test for multiple comparisons. Significance was considered *P* < 0.05. Statistical analyses were performed using GraphPad (version 9.0).

## RESULTS

### Compositional overview of the breast microbiota between tissue types

**Fig 1:**
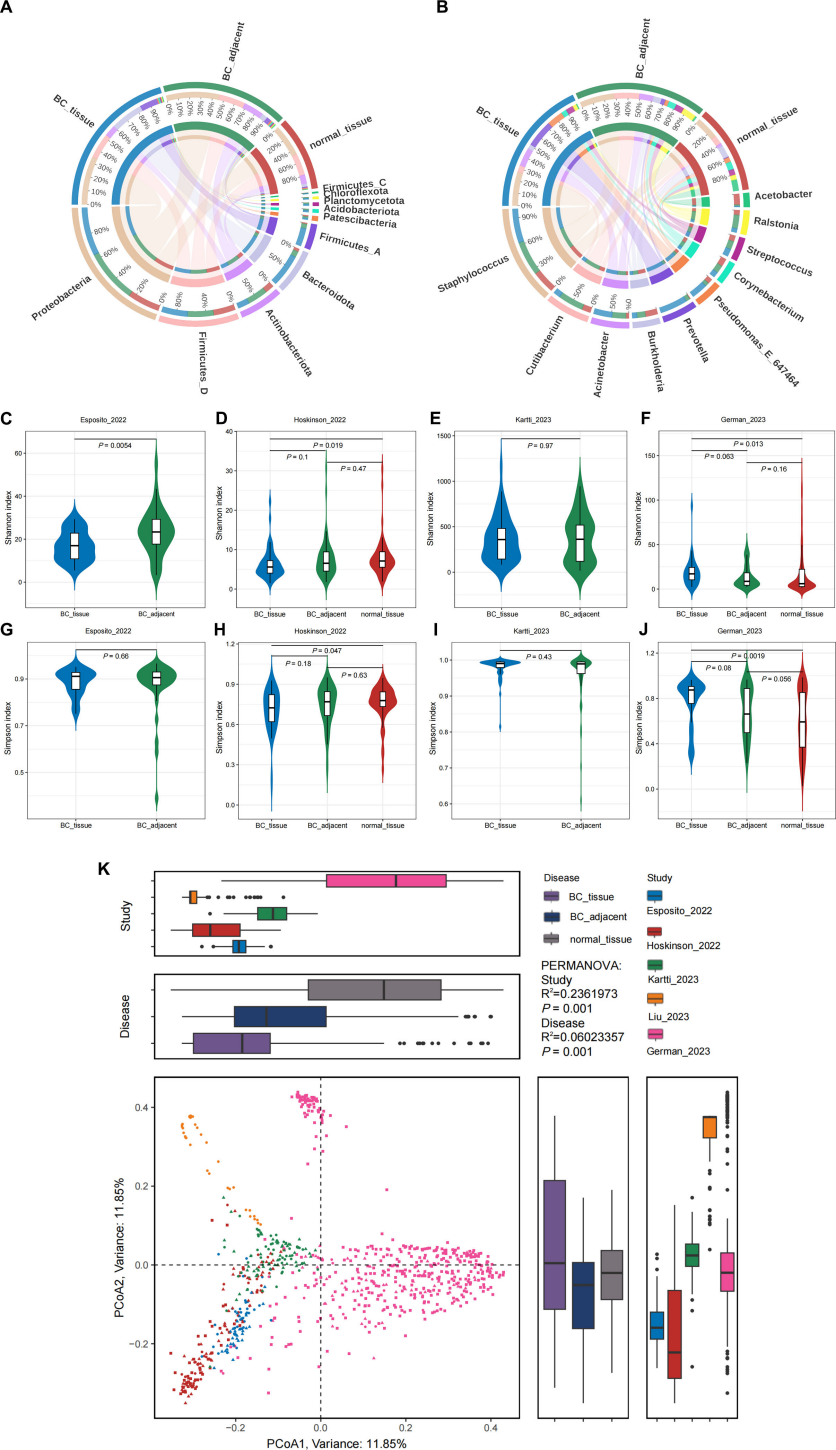
Compositional overview of the breast microbiota between BC_tissue, BC_adjacent, and normal_tissue samples. (**A**) Composition of microbial communities at the phylum level. (**B**) Composition of microbial communities at the genus level. (**C–F**) Alpha diversity, as measured by Shannon’s index, of patients with BC_tissue (blue), BC_adjacent (green), or normal_tissue (red) across the studies by Esposito_2022 (**C**), Hoskinson_2022 (**D**), Kartti_2023 (**E**), and German_2023 (**F**). (**G–J**) Alpha diversity, as measured by Simpson’s index, of patients with BC_tissue (blue), BC_adjacent (green), or normal_tissue (red) across the studies by Esposito_2022 (**G**), Hoskinson_2022 (**H**), Kartti_2023 (**I**), and German_2023 (**J**). (**K**) Principal coordinate analysis (PCoA) revealed a substantial variation in microbial composition among the five study populations (PERMANOVA *R*^2^ = 23.6% in “study,” *P* = 0.001; PERMANOVA *R*^2^ = 6.0% in “disease,” *P* = 0.001). The study was color-coded, and the group was indicated by different shapes. The upper and right boxplots depict the samples projected onto the first two principal coordinates, categorized by study and disease statuses, respectively. All boxplots illustrate the interquartile range (25th–75th percentile) of the distribution, with the median represented by a thick line at the center of the box. The whiskers extend to values within 1.5 times the interquartile range, and outliers are indicated by dots.

We conducted a compositional analysis of patients with BC_tissue, BC_adjacent, and normal_tissue at both the microbial phylum and genus levels. At the phylum level, the microbial communities were predominantly composed of *Proteobacteria*, *Firmicutes_D*, *Actinobacteriota*, *Bacteroidota*, and *Firmicutes_A* (Fig. 1A). Specifically, in BC_tissue, the most abundant phyla were *Proteobacteria* (36.2%), *Bacteroidota* (23.0%), *Firmicutes_D* (18.1%), *Firmicutes_A* (12.5%), and *Actinobacteriota* (8.0%) (Table S2A). In the BC_adjacent tissues, the predominant phyla were *Proteobacteria* (45.8%), *Firmicutes_D* (22.5%), *Actinobacteriota* (17.3%), *Bacteroidota* (5.1%), and *Firmicutes_A* (4.0%) (Table S2A). In normal_tissue samples, the most prevalent phyla were *Proteobacteria* (46.8%), *Firmicutes_D* (31.8%), *Actinobacteriota* (13.2%), *Bacteroidota* (4.4%), and *Firmicutes_A* (1.7%) (Table S2A). Overall, the microbial composition in the three breast tissue conditions was primarily dominated by *Proteobacteria* and *Firmicutes* at the phylum level. At the genus level, the microbial communities were predominantly composed of *Staphylococcus*, *Cutibacterium*, *Acinetobacter*, *Burkholderia*, and *Prevotella* (Fig. 1B). In the BC_tissue samples, the five most abundant genera were *Staphylococcus* (27.2%), *Prevotella* (22.0%), *Acinetobacter* (14.7%), *Pseudomonas_E_647464* (13.0%), and *Streptococcus* (6.0%) (Table S2B). In the BC_adjacent tissues, the top five genera were *Staphylococcus* (28.7%), *Cutibacterium* (20.8%), *Acinetobacter* (11.5%), *Ralstonia* (11.2%), and *Burkholderia* (7.0%) (Table S2B). In normal_tissue, the predominant genera were identified as *Staphylococcus* (27.9%), *Burkholderia* (14.9%), *Acinetobacter* (13.3%), *Cutibacterium* (10.8%), and *Corynebacterium* (9.5%) (Table S2B). Notably, *Staphylococcus* and *Acinetobacter* exhibited the highest prevalence across the three breast types. When compared to BC_tissue, the proportions of *Cutibacterium* and *Burkholderia* were elevated in both BC_adjacent and normal tissue groups.

To investigate the variations in breast microbiota, we assessed alpha diversity across different breast types using the Shannon (Fig. 1C through F) and Simpson (Fig. 1G through J) diversity indices. Given that the study by Liu_2023 included only a single disease state (BC_tissue), we excluded this study from the comparison. Instead, we conducted a comparative analysis of alpha diversity indices across groups in the remaining four studies, which included multiple disease states, as shown in Fig. 1C through J. Notably, in contrast to the other three studies, German_2023 exhibited higher trends in both the Shannon and Simpson indices within the BC_tissue group (Fig. 1F and J). This discrepancy may be attributable to the unequal distribution of cases between the groups in the study of German_2023 (BC_tissue, *n* = 30; BC_adjacent, *n* = 60; normal_tissue, *n* = 402).

PCoA revealed a substantial variation in microbial composition among the five study populations (PERMANOVA *R*^2^ = 23.6% in “study,” *P* = 0.001; PERMANOVA *R*^2^ = 6.0% in “disease,” *P* = 0.001; Fig. 1K). Here, study refers to the five datasets included in our analysis: Esposito_2022, Hoskinson_2022, Kartti_2023, Liu_2023, and German_2023. These studies differed in their study populations and experimental settings, which contributed to the observed variations in microbial composition. PCoA suggested that the variable study significantly influenced the breast microbial composition. Consequently, study was considered a blocking factor to account for batch effects in subsequent analyses. Additionally, substantial variations in microbial diversity existed among the different breast disease states, suggesting that the microbial composition of each breast disease group was distinctly different.

### Prediction performance of specific features in BC studies

**Fig 2:**
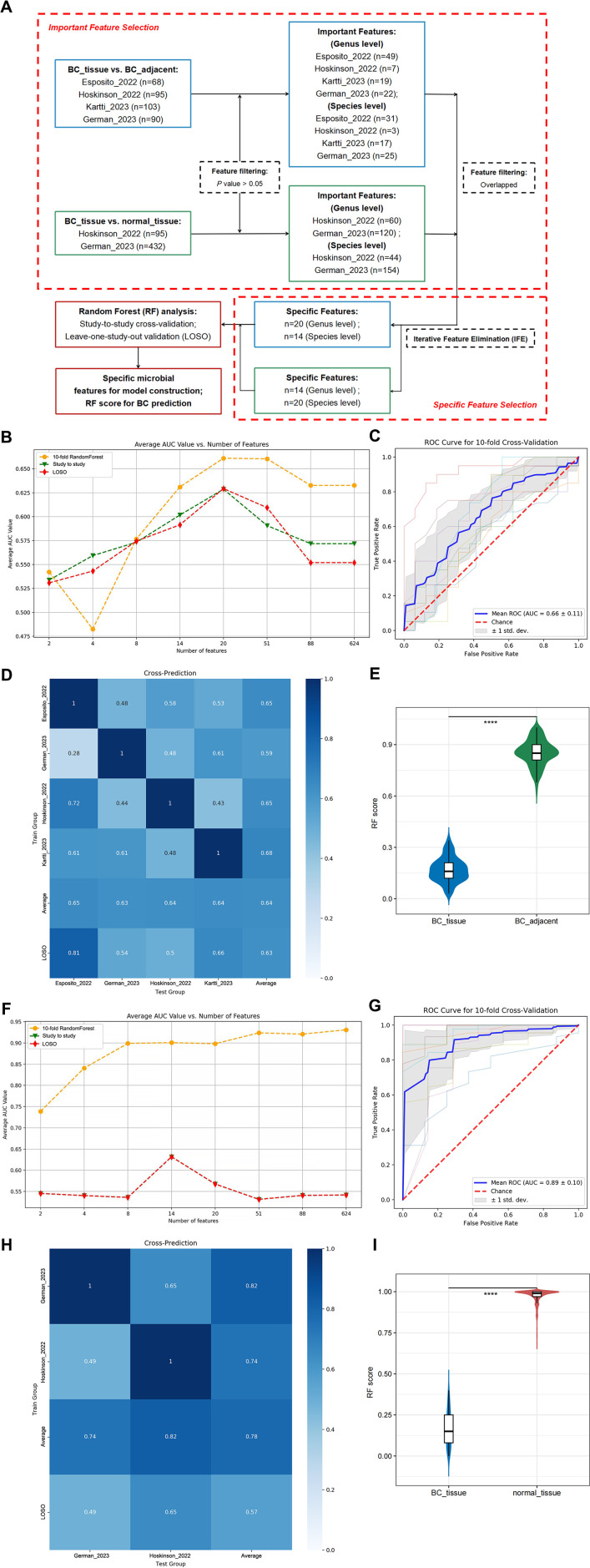
Prediction performance of specific features in BC studies at the genus level. (**A**) Flowchart for microbial model construction. (**B**) The average area under the curve (AUC) values for 10-fold random forest (RF) cross-validation, study-to-study transfer validation classifiers, and leave-one-study-out (LOSO) validation were evaluated for distinguishing between BC_tissue and BC_adjacent, utilizing varying numbers of features. (**C**) The AUC of the optimized models constructed with the 20 specific microbial features for distinguishing BC_tissue from BC_adjacent. Mean AUC and standard deviation of stratified 10-fold cross-validation are shown. (**D**) The prediction performance of specific features was assessed using study-to-study and LOSO validation methodologies. The heatmap illustrates the area under the receiver operating characteristic curve (AUROC) derived from cross-validations within each individual study (represented by blue boxes along the diagonal) and from study-to-study model transfer (external validations, represented by off-diagonal elements). The final column presented the average AUROC for study-to-study predictions. The bottom row indicates the AUROC for a model trained on all studies except one, corresponding to the LOSO validation approach. (**E**) A comparative analysis of RF score distributions, as determined by the BC_tissue and BC_adjacent-related classifier, was conducted between BC_tissue and BC_adjacent samples. Two-sided *P* values were computed utilizing the Wilcoxon rank-sum test. (**F**) The average AUC values for 10-fold RF cross-validation, study-to-study transfer validation classifiers, and LOSO validation were evaluated for distinguishing between BC_tissue and normal_tissue, utilizing varying numbers of features. (**G**) The AUC of the optimized models constructed with the 14 specific microbial features for distinguishing BC_tissue from normal_tissue. (**H**) The prediction performance of specific features was assessed using study-to-study and LOSO validation methodologies. (**I**) Comparison of RF score distributions calculated by the BC_tissue vs. normal_tissue-related classifier between BC_tissue and normal_tissue. ****P < 0.0001.

An important area of investigation involved identifying microbial biomarkers that can predict BC. The methodology for constructing the predictive model is detailed in Fig. 2A. Important microbial features of all comparisons are listed in Table S3A-L.

A total of 20 microbial features at the genus level were filtered to exhibit the highest average AUC and the most significant discriminatory power for distinguishing between BC_tissue and BC_adjacent following IFE (Fig. 2B). Based on the average AUC values, the 20 microbial features identified, in descending order, were *Cutibacterium*, *Acinetobacter*, *Ralstonia*, *Pseudomonas_O_647615*, *Pseudomonas_E_650326*, *Phocaeicola_A_858004*, *GWA2-37-10*, *Bradyrhizobium*, *Cloacibacterium*, *Escherichia_710834*, *Psychrobacter*, *Sporosarcina*, *Neisseria_563205*, *Lawsonella*, *Finegoldia*, *Thermus_A*, *Veillonella_A*, *Hydrogenophilus*, *Blastococcus*, and *Akkermansia* (Table S4A). Among these, *Cutibacterium* was identified as the highest-ranking biomarker, achieving an average AUC of 0.66 for distinguishing between BC_adjacent and BC_tissue (Fig. 2C). To assess the universality and robustness of the identified microbial features across multiple studies, we conducted study-to-study transfer validation and LOSO validation on the entire sample set (Fig. 2D). The AUC values for the study-to-study transfer validation ranged from 0.28 to 1.0, with an average of 0.64. Additionally, the AUC values obtained from the LOSO analysis ranged from 0.50 to 0.81, with an average AUC of 0.63, demonstrating comparable performance to that achieved through study-to-study transfer validation. Furthermore, the RF score derived from 20 differential microbes for BC_adjacent was significantly higher than that for BC_tissue, as determined by the Wilcoxon rank-sum test (Fig. 2E; Table S4B).

In the BC_tissue vs. normal_tissue models, the set of 14 microbial genera demonstrated superior predictive performance compared to other feature sets across all evaluation methods (Fig. 2F). Based on the average AUC value, the 14 microbial features were identified as *Cutibacterium*, *Acinetobacter*, *Burkholderia*, *Prevotella*, *Pseudomonas_E_647464*, *Corynebacterium*, *Streptococcus*, *Ralstonia*, *Lactobacillus*, *GWA2-37-10*, *Anaerococcus*, *Escherichia_710834*, *Duganella_571129*, and *Finegoldia* (Table S5A), in descending order of importance. Among the biomarkers analyzed, *Cutibacterium* emerged as the most prominent. An average AUC of 0.89 was achieved for distinguishing BCs from normal tissues (Fig. 2G). The AUC values for study-to-study transfer validation ranged from 0.49 to 1.0, with an average of 0.78 (Fig. 2H). In contrast, the AUC values for LOSO analysis ranged from 0.49 to 0.65, with an average AUC of 0.57, indicating lower performance compared to the study-to-study transfer validation (Fig. 2H). Furthermore, the RF score derived from 14 differential microbes was significantly higher for normal_tissue compared to BC_tissue (Fig. 2I; Table S5B).

**Fig 3:**
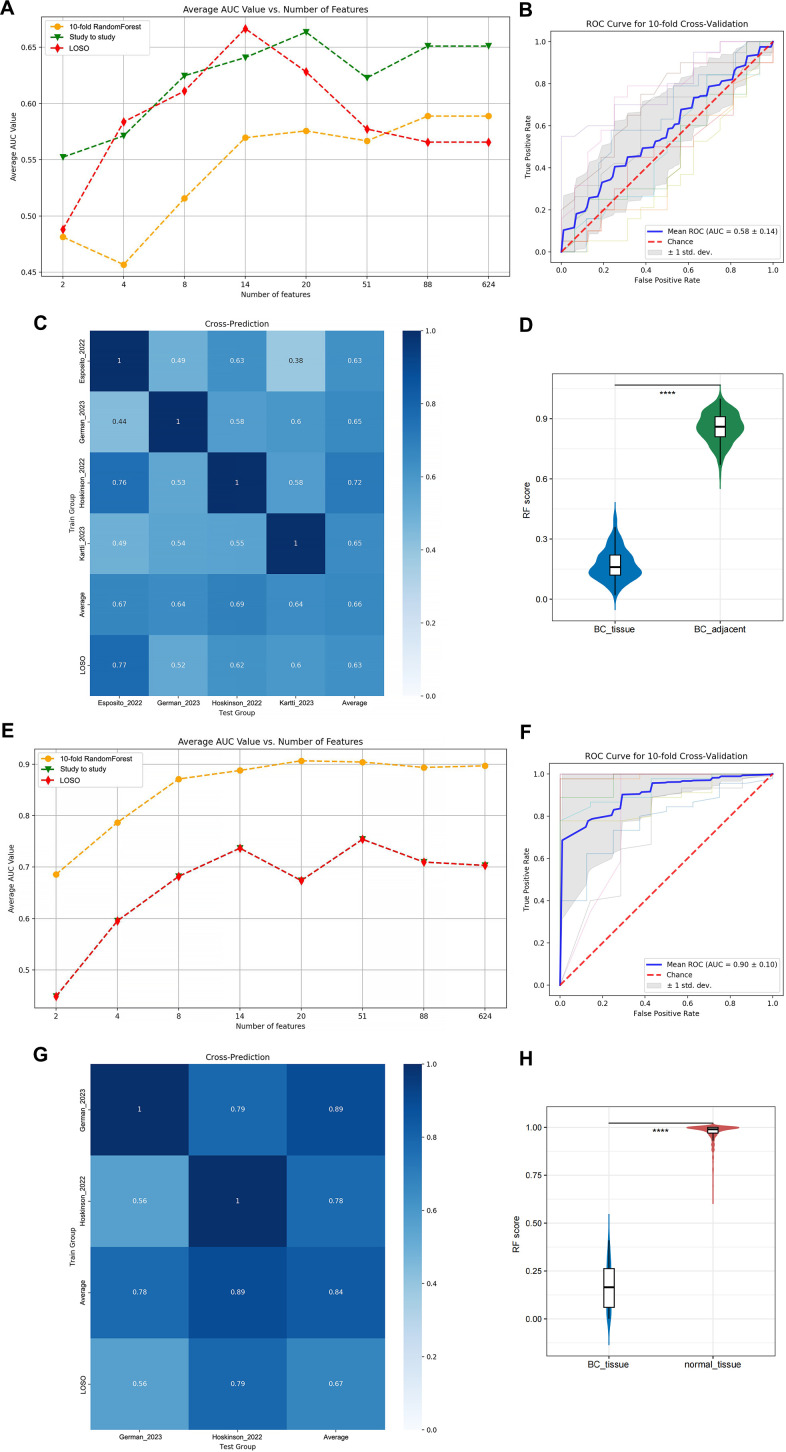
Prediction performance of specific features in BC studies at the species level. (**A**) The average AUC values for 10-fold RF cross-validation, study-to-study transfer validation classifiers, and LOSO validation were evaluated for distinguishing between BC_tissue and BC_adjacent, utilizing varying numbers of features. (**B**) The AUC of the optimized models constructed with the 14 specific microbial features for distinguishing BC_tissue from BC_adjacent. (**C**) The prediction performance of specific features was assessed using study-to-study and LOSO validation methodologies. (**D**) A comparative analysis of RF score distributions, as determined by the BC_tissue and BC_adjacent-related classifier, was conducted between BC_tissue and BC_adjacent samples. (**E**) The average AUC values for 10-fold RF cross-validation, study-to-study transfer validation classifiers, and LOSO validation were evaluated for distinguishing between BC_tissue and normal_tissue, utilizing varying numbers of features. (**F**) The AUC of the optimized models constructed with the 14 specific microbial features for distinguishing BC_tissue from normal_tissue. (**G**) The prediction performance of specific features was assessed using study-to-study and LOSO validation methodologies. (**H**) Comparison of RF score distributions calculated by the BC_tissue vs. normal_tissue-related classifier between BC_tissue and normal_tissue. ****P < 0.0001.

Considering the AUC performance of 10-fold RF, study-to-study and LOSO, in the comparative analysis of BC_tissue vs. BC_adjacent models, the subset of 14 microbial features at the species level exhibited superior predictive performance relative to other feature sets across all evaluation methods (Fig. 3A). Based on the average AUC values, the 14 microbial features identified were *C. acnes*, *Acinetobacter_ johnsonii*, *Pseudomonas_O_647615_parafulva*, *Ralstonia_pickettii_B*, *Atopostipes_suicloacalis*, *Rubrobacter_B_405439_xylanophilus*, *Novosphingobium_capsulatum*, *Psychrobacter_maritimus*, *Finegoldia_magna_H*, *Thermus_A_scotoductus*, *Phocaeicola_A_858004_vulgatus*, *Acinetobacter_harbinensis*, *Bifidobacterium_thermophilum*, and *Tepidimonas_fonticaldi* (Table S6A), respectively. Among these biomarkers, *C. acnes* was identified as the highest ranking. An average AUC of 0.58 was achieved for distinguishing BC_tissue from BC_adjacent (Fig. 3B). The AUC values for study-to-study transfer validation ranged from 0.38 to 1.0, with an average of 0.66 (Fig. 3C). Additionally, the AUC values from LOSO analysis ranged from 0.52 to 0.77, with an average AUC of 0.63, demonstrating comparable performance to the study-to-study transfer validation (Fig. 3C). Finally, the RF score derived from 14 differential microbes was significantly higher in BC_adjacent compared to BC_tissue (Fig. 3D; Table S6B).

A robust RF model was ultimately developed using a core set of optimal features, including 20 differential microbial markers at the species level (Fig. 3E). The 20 microbial features, ranked according to their average AUC values, were identified as follows: *C. acnes*, *Ralstonia_pickettii_B*, *JC017_sp004296775*, *Lactobacillus_iners*, *Lacticaseibacillus_paracasei*, *Acetobacter_garciniae*, *Burkholderia_lata*, *Burkholderia_cepacia_576714*, *Burkholderia_mallei*, *Atopostipes_suicloacalis*, *Oceanobacillus_luteolus*, *Escherichia_coli*, *Finegoldia_magna_H*, *Herbaspirillum_huttiense*, *Enterococcus_H_360604_faecalis*, *Veillonella_A_rogosae*, *Akkermansia_muciniphila_D_776786*, *Ureaplasma_sp900544585*, *QHXM01_sp003222945*, and *Arachnia_flavescens* (Table S7A). This model achieved an average AUC of 0.90 in distinguishing BCs from normal tissues (Fig. 3F). Among the biomarkers analyzed, *C. acnes* emerged as the most prominent. The AUC values for study-to-study transfer validation ranged from 0.56 to 1.0, with an average of 0.84 (Fig. 3G). In contrast, the AUC values for LOSO analysis ranged from 0.56 to 0.79, with an average AUC of 0.67, indicating lower performance compared to the study-to-study transfer validation (Fig. 3G). Additionally, the RF score derived from 20 differential microbes was significantly higher in normal_tissue compared to BC_tissue (Fig. 3H; Table S7B).

### Abundance of *C. acnes* varies between BC tissues and peritumoral or normal breast tissues

**Fig 4:**
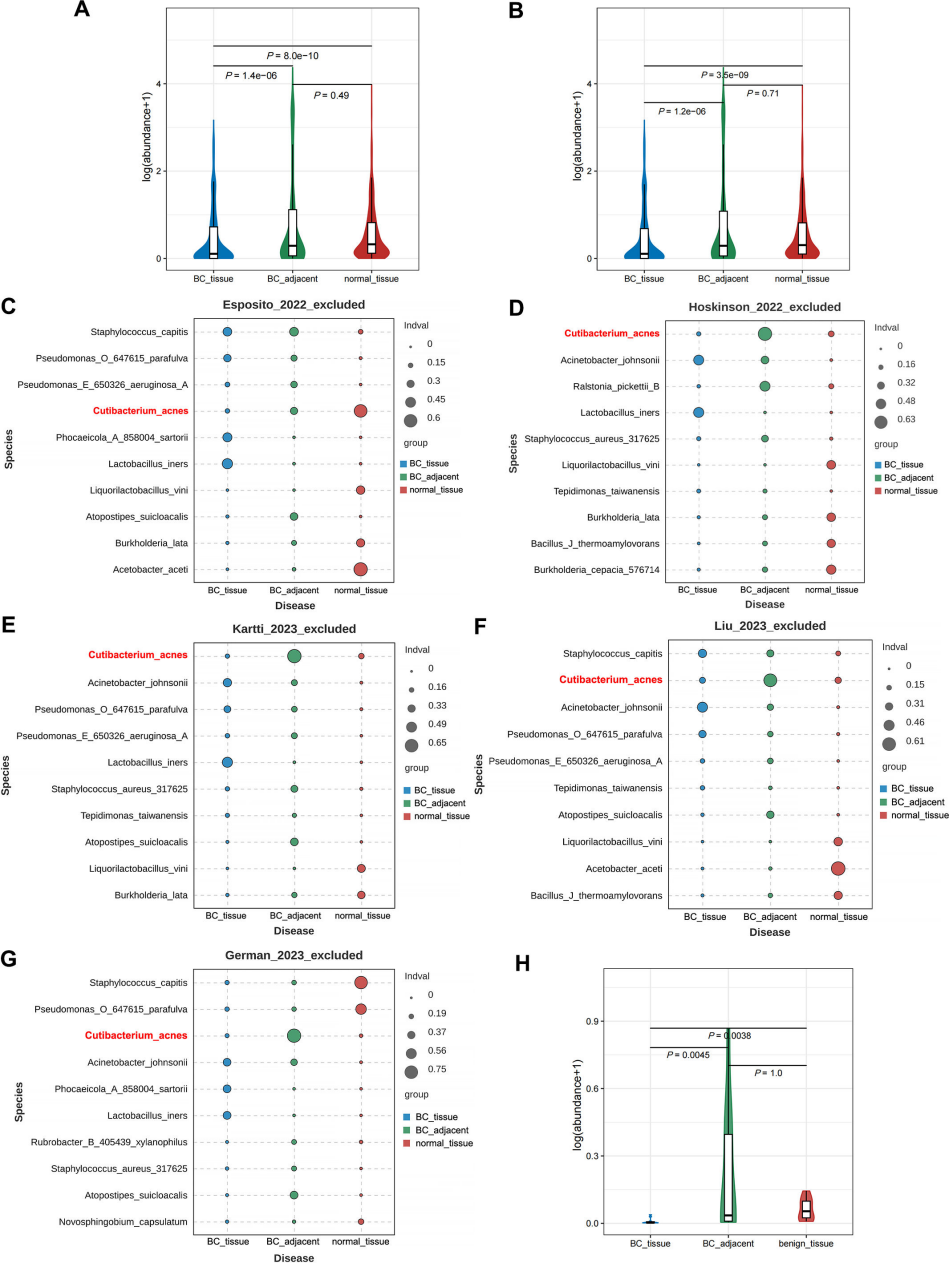
Abundance of *Cutibacterium* and *Cutibacterium acnes* varies between BC tissues and peritumoral or normal breast tissues. (**A**) The abundance difference of *Cutibacterium* in patients with BC_tissue, BC_adjacent, and normal_tissue. (**B**) The abundance difference of *C. acnes* in patients with BC_tissue, BC_adjacent, and normal_tissue. (**C–G**) The abundance of the top 10 species subsequent to the exclusion of Esposito_2022 (**C**), Hoskinson_2022 (**D**), Kartti_2023 (**E**), Liu_2023 (**F**), or German_2023 (**G**), respectively. (**H**) The abundance of *Cutibacterium* in patients with BC_tissue, BC_adjacent, and benign_tissue in our in-house data set.

Based on the aforementioned findings, it can be inferred that *Cutibacterium*, specifically *C. acnes*, served as a highly effective marker for differentiating BC tissues from peritumoral or normal breast tissues. At the genus level, the abundance of *Cutibacterium* in patients with BC_adjacent or normal_tissue was significantly greater than in those with BC_tissue (*P* < 0.001; Fig. 4A; Table S8A). Correspondingly, a significant increase in *C. acnes* abundance was observed in patients with BC_adjacent or normal_tissue compared to those with BC_tissue (both *P* < 0.001; Fig. 4B; Table S8B). However, no statistically significant difference in microbial abundance was observed between individuals with BC_adjacent tissue and those with normal_tissue (*P* > 0.05; Fig. 4A and B; Table S8A and B), irrespective of the taxonomic resolution at the genus or species level.

To assess the stability and robustness of our findings, we performed a validation step in which one study was excluded at a time, and the abundance of *C. acnes* was reevaluated in the remaining four studies. This approach allows us to determine whether the identified microorganisms consistently correlate with tumors across multiple data sets, thereby minimizing the risk of results being influenced by any specific biases or anomalies in a single study or data set. Figure 4C through G illustrated the top 10 species following the exclusion of Esposito_2022, Hoskinson_2022, Kartti_2023, Liu_2023, or German_2023, respectively. In the Esposito_2022_excluded study, the abundance of *C. acnes* in patients with BC_adjacent or normal_tissue was significantly higher than in those with BC_tissue (*P* < 0.001, Fig. 4C). Additionally, the median abundance of *C. acnes* was higher in patients with normal_tissue compared to those with BC_adjacent tissue (both *P* = 0.004, Fig. 4C). In the study by Hoskinson_2022_excluded, *C. acnes* was observed with a significantly increased abundance in patients with BC_adjacent compared with those with BC_tissue or normal_tissue individuals (both *P* < 0.001, Fig. 4D). Moreover, the abundance of *C. acnes* in patients with normal_tissue was significantly higher than that in BC_tissue (*P* = 0.041, Fig. 4D). For Kartti_2023_excluded, a significant increase in *C. acnes* abundance was observed in patients with BC_adjacent compared with BC_tissue or normal_tissue individuals (both *P* < 0.001, Fig. 4E). However, there was no significant difference in *C. acnes* abundance between the latter two groups (all *P* values > 0.05, Fig. 4E). In the study conducted by Liu_2023_excluded, a significant increase in the abundance of *C. acnes* was observed in patients with BC_adjacent tissue compared to those with BC_tissue or normal tissue (both *P* < 0.001, Fig. 4F). However, no significant difference in *C. acnes* abundance was found between the BC_tissue and normal tissue groups (*P* > 0.05, Fig. 4F). Similarly, in the study by German_2023_excluded, the median abundance of *C. acnes* was higher in patients with BC_adjacent tissue compared to those with BC_tissue or normal tissue (both *P* < 0.001, Fig. 4G), with no significant difference observed between the latter two groups (all *P* values > 0.05, Fig. 4G).

To further substantiate the role of *Cutibacterium* or *C. acnes* in BC prediction, a small-scale in-house data set was integrated into this study. At the genus level, the abundance of *Cutibacterium* in patients with BC_adjacent or benign_tissue was significantly higher than in those with BC_tissue (*P* = 0.0045 and 0.0038; Fig. 4H; Table S8C). However, due to the limited sample size, no statistically significant differences were observed in the abundance of *C. acnes* among patients with various breast diseases.

### Microbial functional alterations in different breast conditions

**Fig 5:**
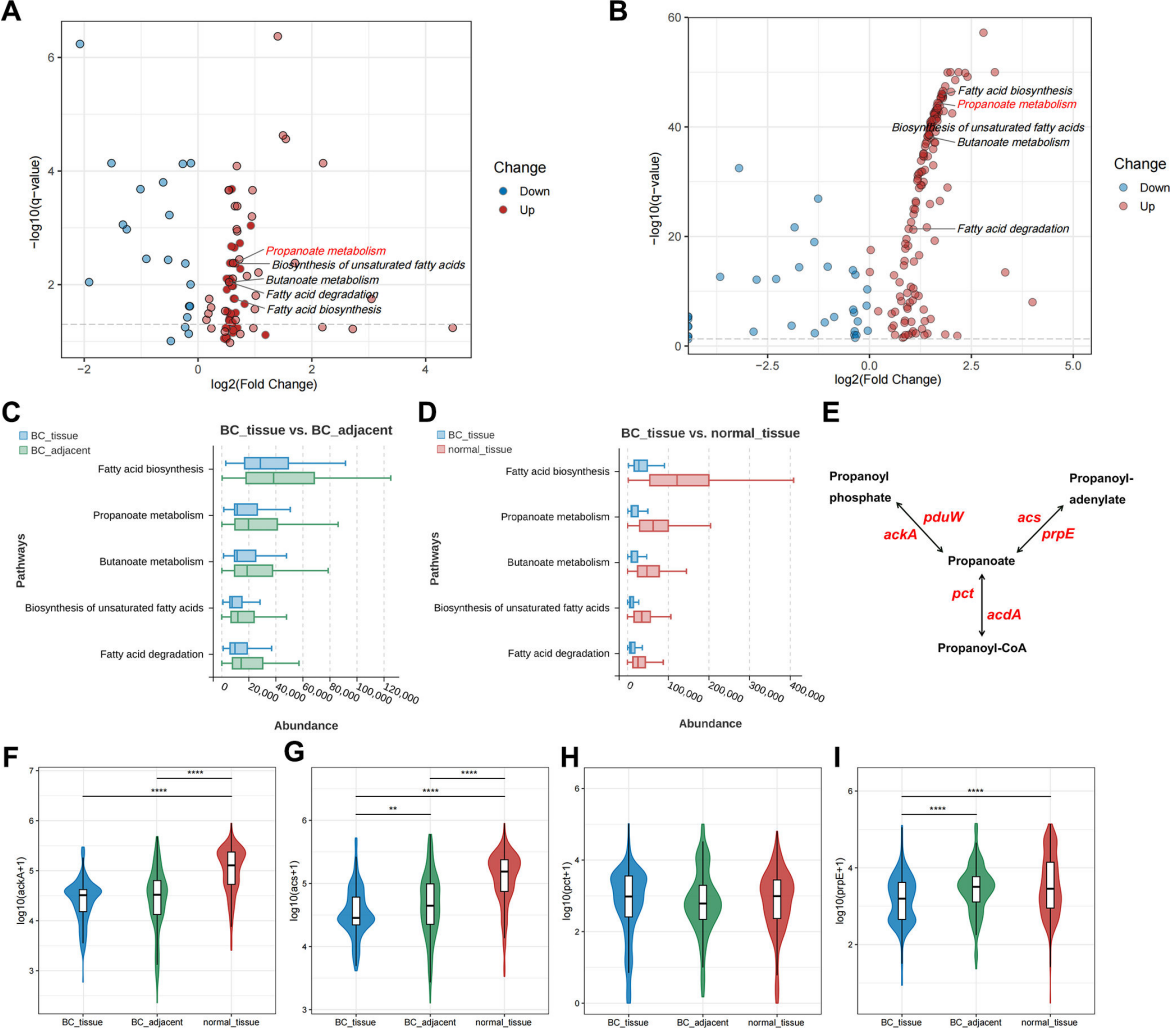
Microbial functional alterations in different breast conditions. (**A**) Visualization of differential pathways between BC_tissue and BC_adjacent by volcano plot, with specific emphasis on marking the fatty acid-related pathways (fatty acid biosynthesis, propanoate metabolism, butanoate metabolism, biosynthesis of unsaturated fatty acids, and fatty acid degradation). The red dots indicated the upregulation of the differential pathway in BC_adjacent, whereas the blue dots indicated the upregulation of the differential pathway in BC_tissue. (**B**) Visualization of differential pathways between BC_tissue and normal_tissue by volcano plot, with specific emphasis on marking the fatty acid-related pathways. The red dots indicate the upregulation of the differential pathway in normal_tissue, whereas the blue dots indicated the upregulation of the differential pathway in BC_tissue. (**C**) Comparison of the differences in abundance between BC_tissue (blue) and BC_adjacent (green) fatty acid pathways. (**D**) Comparison of the differences in abundance between BC_tissue (blue) and normal_tissue (red) fatty acid pathways. (**E**) Schematic representation of the propanoate biosynthesis pathway, highlighting its rate-limiting enzymes, including *acdA* (acetate-CoA ligase [ADP-forming], EC:6.2.1.13), *ackA* (acetate kinase, EC:2.7.2.1), *acs* (ACSS, acetyl-CoA synthetase, EC:6.2.1.1), *pct* (propionate CoA-transferase, EC:2.8.3.1), *pduW* (propionate kinase, EC:2.7.2.15), and *prpE* (propionyl-CoA synthetase, EC:6.2.1.17). (**F–I**) Comparison of the differences in abundance of *acs* (**F**), *prpE* (**G**), *ackA* (**H**), and *pct* (**I**) in patients with BC_tissue, BC_adjacent, and normal_tissue. **P < 0.01, and ****P < 0.0001.

We conducted an in-depth analysis of microbiome-based functional alterations across various BC disease conditions utilizing 16S rRNA sequencing data. Our findings revealed 96 differential pathways between BC_tissue and BC_adjacent (Fig. 5A; Table S9A), and 188 differential pathways between BC_tissue and normal_tissue (Fig. 5B; Table S9B), consistently identified across multiple studies. Furthermore, our discussion highlighted a significant variation in the expression of *C. acnes* across different breast tissue conditions. Consequently, the metabolic pathways associated with *C. acnes* warrant further investigation.

*C. acnes* utilizes triglycerides as its primary energy source and secretes significant quantities of fatty acids (35). Our study concentrated on the alterations in fatty acid-related metabolic pathways. Specifically, when comparing BC_tissue to BC_adjacent or normal_tissue, we observed a reduction in the pathways associated with fatty acid biosynthesis, propanoate metabolism, butanoate metabolism, biosynthesis of unsaturated fatty acids, and fatty acid degradation in BC samples (Fig. 5A through D).

**Fig 6:**
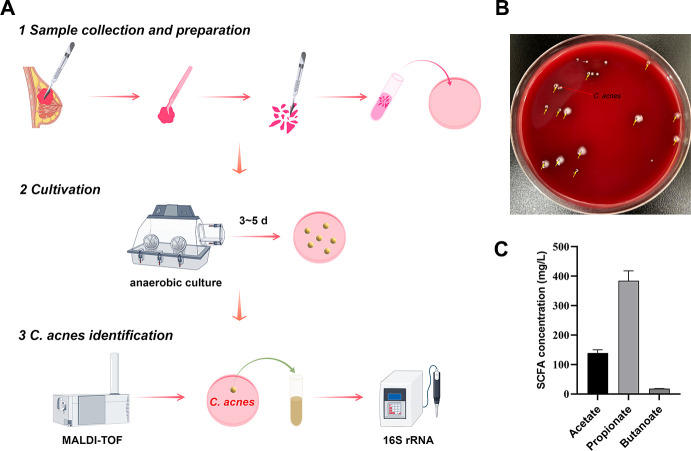
Culture and identification of *C. acnes*. (**A**) Cultureomics workflow for *C. acnes*. (**B**) Colonies of breast microbes on Columbia blood agar. The yellow arrows indicate the traces of the colonies selected for MALDI-TOF MS. The red arrow indicates that the colony had been preliminarily confirmed as *C. acnes* by MALDI-TOF MS. (**C**) Analysis of the SCFAs (acetate, propionate, and butanoate) in the supernatant of *C. acnes* using HPLC-MS.

*C. acnes* predominantly secretes SCFAs, with propionate being the most abundant (35, 36). To comprehensively investigate the metabolome of *C. acnes*, we conducted a non-targeted metabolomic analysis of the SCFAs present in the culture supernatants of *C. acnes* strains. The methodology for culturing and identifying *C. acnes* is detailed in Fig. 6A. Consistent with prior studies (35, 36), the SCFAs produced by *C. acnes* were predominantly composed of propionate (Fig. 6C).

Notably, we observed several rate-limiting enzymes linked to the propanoate biosynthesis pathway, including *acdA* (acetate-CoA ligase [ADP forming], EC 6.2.1.13), *ackA* (acetate kinase, EC 2.7.2.1), *acs* (ACSS, acetyl-CoA synthetase, EC 6.2.1.1), *pct* (propionate CoA-transferase, EC 2.8.3.1), *pduW* (propionate kinase, EC 2.7.2.15), and *prpE* (propionyl-CoA synthetase, EC 6.2.1.17) (Fig. 5E; Table S9C, D; Fig. 1). The abundance of *acdA* and *pduW* in over 10% of the three breast tissue samples was found to be zero, precluding further comparative analysis. In contrast to BC tissues, the expression levels of *acs* (Fig. 5F) and *prpE* (Fig. 5G) genes were significantly elevated in adjacent and normal breast tissues (all *P* values < 0.05). The abundance of *ackA* in normal tissue was significantly higher compared to both BC tissue and BC_adjacent tissue (Fig. 5H). However, there was no significant difference in the abundance of *pct* among the three breast tissue samples (Fig. 5I).

### *C. acnes* inhibited BC cell growth

Utilizing the Figdraw platform (https://www.figdraw.com/#/), we delineated the culturomics workflow (Fig. 6A). Bacterial isolation was conducted on 10 human BC samples from our in-house cohort. *C. acnes* was successfully cultured from 2 out of the 10 breast tissue samples (20%). Fig. 6B showed a picture of typical breast microbial colonies cultured on Columbia blood agar. Preliminary identification of these isolates as *C. acnes* strain_A and *C. acnes* strain_B was achieved using MALDI-TOF MS. Subsequent BLAST analysis and syntenic alignment revealed that *C. acnes* strain_A exhibited gene clusters with greater than 99% identity compared to the reference *C. acnes* strains NR113028.1 (99.928%) and NR040847.1 (99.928%) (Table S10). The *C. acnes* strain_B exhibited gene clusters with greater than 99% identity when compared to the reference strains NR113028.1 (100%) and NR040847.1 (100%) (Table S10). Finally, it can be concluded that *C. acnes* was successfully cultured from breast tissue samples.

Given that the supernatant of the bacterial culture contained macromolecules and bacterial metabolites, experiments were subsequently designed to elucidate the functional roles of the *C. acnes* strain. The supernatants of *C. acnes* were analyzed using HPLC-MS, and the procedure was conducted in triplicate. The results indicated that propionate was the predominant compound, with an average concentration of 384 mg/L (Fig. 6C). The molecular weight of propionic acid is 74.08, and its concentration in the supernatant of *C. acnes* was 0.005 mol/L, equivalent to 5 mM. The concentration of propionate in the supernatant of a 20% *C. acnes* culture was approximately 1 mM. Therefore, in the subsequent cell experiments, we treated the cells with 20% *C. acnes* supernatant and 1 mM SP, respectively.

**Fig 7:**
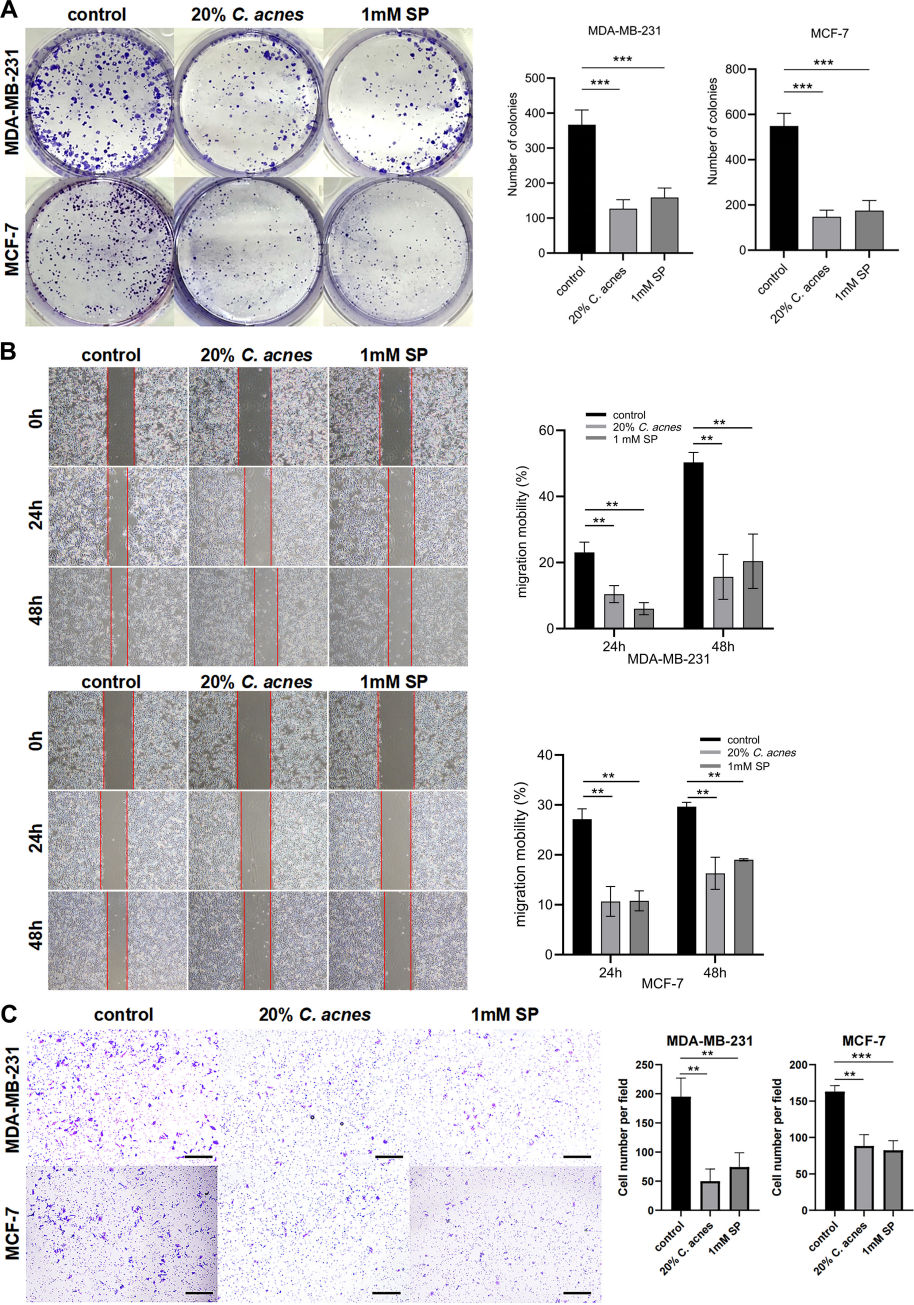
*C. acnes* inhibited BC cell growth. (**A**) Colony formation assays showed the proliferation of MDA-MB-231 and MCF-7 cells treated in either the absence or the presence of 20% *C. acnes* supernatant or 1 mM sodium propionate (SP). (**B**) Scratch assays of MDA-MB-231 cells (upper panel) and MCF-7 cells (lower panel) conducted in the absence or presence of either 20% *C. acnes* supernatant or 1 mM SP. (**C**) Transwell assays of MDA-MB-231 cells and MCF-7 cells conducted in the absence or presence of either 20% *C. acnes* supernatant or 1 mM SP (scale bar, 100 µm). **P < 0.01, and ***P < 0.001.

Next, two human BC cell lines (MDA-MB-231 and MCF-7) were respectively incubated with *C. acnes* supernatant and SP to investigate how they influence various cancer hallmarks of recipient cells. The colony formation assay demonstrated that 20% *C. acnes* supernatant and 1 mM SP significantly inhibited the proliferation of MDA-MB-231 and MCF-7 cells in comparison to the control group (*P* < 0.05; Fig. 7A). Experimental groups were subjected to treatment for either 24 or 48 hours with 20% *C. acnes* supernatant, 1 mM SP, or the control. The migration rate was subsequently assessed using a scratch wound healing assay. Relative to the control cells, the cells treated with 20% *C. acnes* supernatant or 1 mM SP demonstrated significantly larger wound areas at both 24 and 48 hour time points (*P* < 0.05; Fig. 7B). The Transwell assays demonstrated that both 20% *C. acnes* supernatant and 1 mM SP significantly reduced cell invasion in MDA-MB-231 and MCF-7 cells when compared to the control groups (*P* < 0.05; Fig. 7C). Collectively, *C. acnes* supernatant or SP could weaken the malignant behaviors of BC cells, which included proliferation, migration, and invasion.

### *C. acnes* exhibited antitumor effect *in vivo*

**Fig 8:**
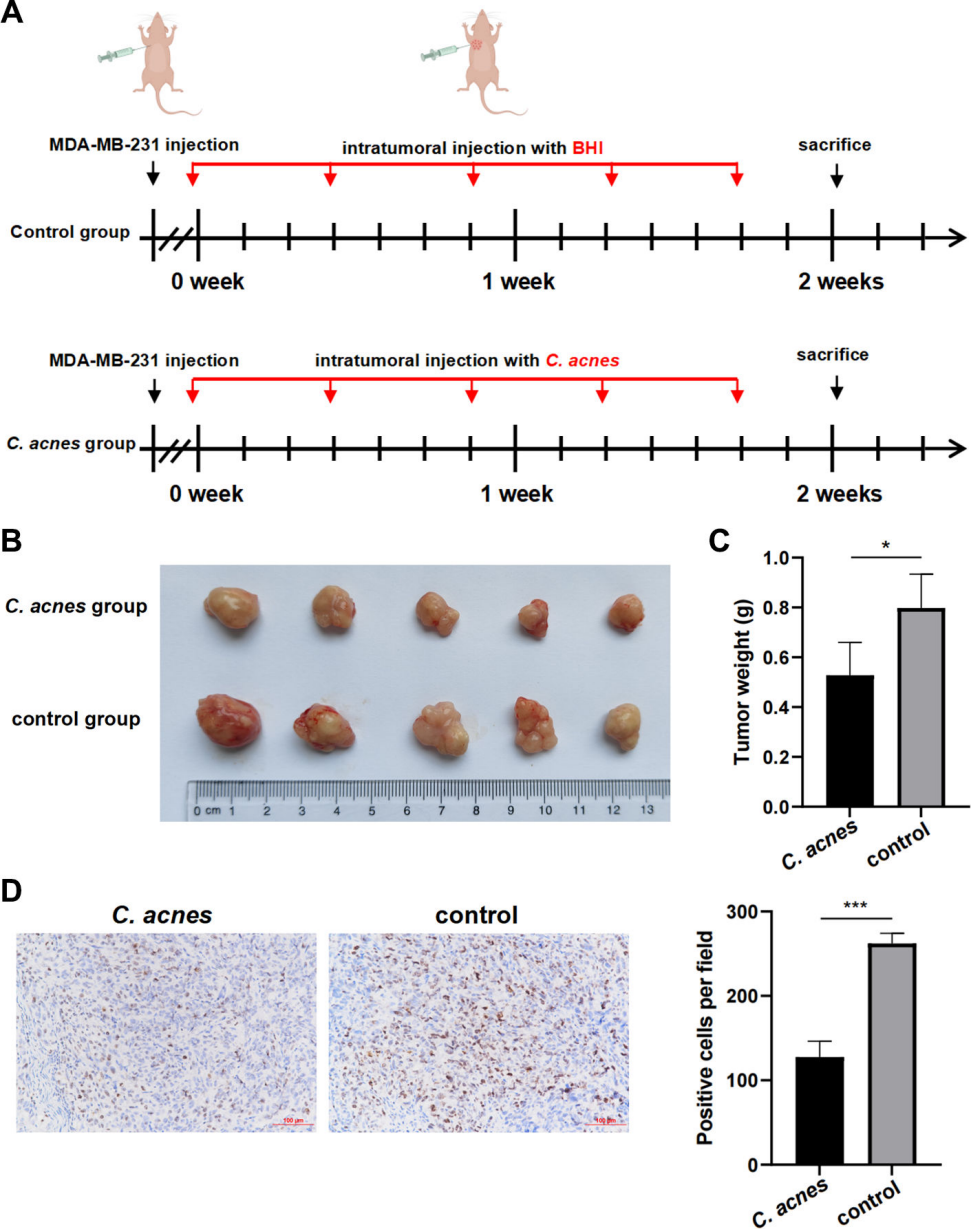
*C. acnes* exhibited an antitumor effect *in vivo*. (**A**) Experiment flowchart. MDA-MB-231 cells were subcutaneously implanted into BALB/c mice. After implantation, the mice were intratumorally injected with *C. acnes* or BHI every 3  days for 2  weeks. (**B**) Representative *in situ* images of tumors. The tumor growth in the *C. acnes* group was significantly inhibited. (**C**) Representative quantified graph of tumor weights. (**D**) Representative Ki-67 immunostaining of xenograft tumor tissues. Original magnification, ×20. Scale bar, 100  µm. *P <  0.05, *P < 0.01, and ***P <  0.001.

We assessed whether *C. acnes* exhibited potential antitumor properties *in vivo* using a subcutaneous xenograft mouse model with implantation of the BC cell line MDA-MB-231 (Fig. 8A). Our findings demonstrated that the *C. acnes* group exhibited a significant reduction in both tumor volume (Fig. 8B) and tumor weight (Fig. 8C) compared to the control group. Furthermore, *C. acnes* markedly inhibited cell proliferation within the tumor tissue, as evidenced by significantly lower Ki-67-positive cells (*P* < 0.05; Fig. 8D) relative to the control group. These consistent results from our *in vivo* experiments suggested that *C. acnes* may possess antitumor activity in BC.

## DISCUSSION

Several studies have linked the microbiome to the initiation and progression of different types of cancer, including BC (37, 38). The cooperation of microbial communities’ imbalance with diet, obesity, estrogens, and immune modulation has been considered an important promoter of BC (39). Notably, the majority of authors noted that their findings were hypothesis generators and support further investigations to identify a microbial risk signature for BC and potential microbial-based prevention and/or therapies (6, 12, 40). In this scenario, we reanalyzed five available public data sets of human breast microbiota via consistent pipelines and comprehensively assessed alterations of the breast microbiome across the BC cascade in an effort to address the issue of reproducibility among heterogeneous studies. The aim of our study was to evaluate the microbial composition of breast tumor tissues and healthy tissues in the attempt to shed light on the link between dysbiosis and BC, which, in turn, may indicate that a change in bacterial species could contribute to the modulation of cancer development. Generally speaking, the three breast states were predominantly composed of *Proteobacteria* and *Firmicutes* at the phylum level. While the breast microbiota shares some similarities with other body sites, such as the presence of *Proteobacteria* and *Firmicutes*, the microbial community in the breast is likely influenced by factors unique to the local microenvironment, including the fatty acid-rich environment of the breast. However, it is important to note that microbes such as *Bacteroides*, *Proteobacteria*, *Firmicutes*, and *Actinobacteria* are also commonly found in other body sites, particularly the gut. The presence of these microbes in the breast is not solely attributable to fatty acid richness (9). SCFAs, which are present in various body sites, are linked to multiple health conditions, including diabetes, obesity, and cancers in different organs. Therefore, the composition of the breast microbiota is likely shaped by a combination of local environmental factors and systemic influences, rather than being driven by a single factor such as fatty acid content. In the genus level, compared with BC_tissue, the proportions of *Cutibacterium* and *Burkholderia* in BC_adjacent and normal_tissue groups were obviously increased. This observation suggested a potential anticancer effect associated with these genera. By differential expression analysis, Chai et al. showed that *Burkholderia* had a high biomass in adjacent intrahepatic cholangiocarcinoma tissues and revealed antitumor potentials (41). Whether *Cutibacterium* has an antitumor effect needs to be demonstrated.

Our study aimed to identify microbial markers specific to BC, with the ultimate goal of advancing early detection methods for patients. We found that the breast microbiome is significantly altered in BC tissues, and microbial features hold potential for distinguishing BC from normal or adjacent tissues. The model we developed showed moderate accuracy, with an AUC of 0.66 at the genus level and 0.58 at the species level when distinguishing BC tissue from adjacent tissue. Notably, the model achieved higher accuracy when differentiating BC tissue from normal tissue, with AUC values of 0.89 at the genus level and 0.90 at the species level. Despite these promising results, the model’s ability to distinguish BC tissue from adjacent tissue was less accurate, which likely reflects the similarity between the microbiomes of BC and adjacent tissues. This finding highlights the challenges in detecting BC-specific microbial markers, as the microbiome in adjacent tissues may share key characteristics with that of the tumor, thus making the distinction more difficult. These findings underscore the potential of the breast microbiome as a diagnostic tool but also suggest that further refinement is needed to enhance the sensitivity and specificity of microbial markers for BC detection. The close resemblance of the microbiomes of BC and adjacent tissues suggests that the microbial changes associated with cancer may be subtle and localized, requiring more precise methods to identify robust biomarkers. Moreover, the observed differences between BC and normal tissues further support the relevance of the microbiome in cancer biology, warranting future exploration of its role in BC progression and prognosis.

Microbial communities varied between benign breast lesions and cancer during BC progression (9, 10). Nevertheless, *Cutibacterium* and *C. acnes* were identified as potential diagnostic markers, particularly for distinguishing BC from normal breast tissue. *C. acnes* was primarily known for its pathogenic involvement in acne, whereas recent studies have demonstrated its protective role in normal skin and atopic dermatitis. Therefore, the roles of *C. acnes* are far more complicated than previously thought (42). Besides skin diseases, *C. acnes* also plays a complex role in many human tumor diseases. *C. acnes* extracellular vesicles were taken up by renal carcinoma cells to enhance their proliferative potential. *C. acnes* extracellular vesicles also exhibited tumor-promoting activity in a mouse model of renal cancer allografts with enhanced angiogenesis (43). Jingushi et al. suggested that extracellular vesicles released by *C. acnes* localized in renal cell carcinoma tissues act in a tumor-promoting manner (43). Brüggemann et al. suggested that a role for *C. acnes* in the development of prostate cancer was possible. Experiments in laboratory animals revealed persistent colonization and inflammation of the prostate following *C. acnes* inoculation (44). In addition to its potential role in promoting cancer, *C. acnes* may also exhibit anticancer effects. The oncogenic transcription factor *Foxm1* is highly expressed in gastric cancer tissues. Lunger et al. verified that coinfection with thiopeptide-positive *C. acnes* would decrease *Foxm1* expression and alter *Helicobacter pylori*-induced pathogenesis (45). Chintalapati et al. revealed that *C. acnes*, belonging to the *Cutibacterium* genus isolated from the transplantation models of mouse sarcoma, showed good tumor-suppressing ability after injecting this bacterium through the tail vein by intravenous injection (46). Our findings indicated that, in comparison to BC tissues, the abundance of *C. acnes* was significantly elevated in adjacent and normal breast tissues. This observation led us to hypothesize that *C. acnes* may play a protective role in BC. To explore the potential role of this breast-associated microbe, we performed functional experiments to investigate the effects of *C. acnes*, the most abundant species identified in patients with BC. Our results showed that *C. acnes* remarkably suppressed the growth of *in vitro* cancer cells, suggesting a potential protective role for this microorganism. However, these *in vitro* findings are limited and should be interpreted with caution, as they do not establish causality. Further *in vivo* or longitudinal studies are necessary to better understand the protective potential of *C. acnes* and how dysbiosis might disrupt this balance, possibly contributing to the progression of BC.

Functional analysis revealed complex underlying mechanisms that could improve our understanding of BC carcinogenesis. We found that, compared to BC tissues, *C. acnes*-related propanoate metabolism was significantly enriched in adjacent tissues and controls. The increasing abundance of the propanoate metabolism pathway from BC to adjacent tissues and controls suggests that enhanced activity of this pathway might contribute to the progression of BC. Despite its potential relevance, propionate metabolism is often overlooked in cancer metabolism research. In recent years, a few studies have begun to report the effects of propionate metabolism alternates on tumors. Ryu et al. showed that gut-microbiome-derived propionate suppressed colorectal cancer growth by promoting the proteasomal degradation of euchromatic histone-lysine N-methyltransferase 2 through HECT domain E3 ubiquitin protein ligase 2 upregulation (47). Ramesh et al. reported that treatment with propionate *in vitro* reinforced the epithelial transcriptional program promoting cell-to-cell contact and cell adhesion while reducing the aggressive and chemo-resistant epithelial-mesenchymal transition phenotype in lung cancer cell lines (48). Gomes et al. found that dysregulation of propionate metabolism produces a pro-aggressive signature in BC cells, increasing their metastatic potential (49). The alteration of propionate metabolism was an important contributor to cancer and a valuable potential target in the therapeutic treatment of carcinomas. Moreover, our analysis revealed significant differential expression of a series of genes involved in propanoate biosynthesis within the propanoate metabolism pathway, including *acs* (ACSS, acetyl-CoA synthetase) and *prpE* (propionyl-CoA synthetase), between BC tissues and adjacent non-cancerous tissues. These findings suggested that propanoate biosynthesis and its associated genes may provide valuable insights and potential targets for therapeutic intervention in the carcinogenesis of BC.

### Conclusion

In conclusion, our study highlighted the predictive capacity of microbial biomarkers in the onset of BC. Notably, specific bacterial species within the breast microbiome, such as *Cutibacterium* and *C. acnes*, along with their metabolite propionate, exhibited potential as diagnostic markers for BC and may contribute significantly to antitumor activity. However, the relatively small number of studies included in this analysis may limit the generalizability of our findings. The restricted data set size could potentially affect the interpretation of the results, underscoring the need for future research with larger sample sizes and more comprehensive data sets to draw more robust conclusions about the role of the microbiome in breast cancer. Furthermore, the molecular mechanisms governing their interactions with cancer cells are not yet fully understood, necessitating further investigation to explore their viability as potential targets for tumor prevention.

## Data Availability

The 16S rDNA sequencing data sets have been deposited with links to BioProject accession numbers PRJNA759366, PRJNA723425, PRJNA926328, PRJNA769523, PRJNA867176, and PRJNA1113855 in the ENA database (https://www.ebi.ac.uk/ena). Supplementary files are provided with this paper.

## SUPPLEMENTAL MATERIAL

Table S1 msystems.00271-25-s0001.doc Clinical characteristics of in-house cohort.

Table S2 msystems.00271-25-s0002.doc The abundance of BC_tissue, BC_adjacent, and normal_tissue.

Table S3 msystems.00271-25-s0003.doc The characteristics of important bacterial features at the genus level compared between BC_tissue and BC_adjacent.

Table S4 msystems.00271-25-s0004.doc The abundance of the 20 specific genera in both BC_tissue and BC_adjacent samples.

Table S5 msystems.00271-25-s0005.doc The abundance of the 14 specific genera in both BC_tissue and normal_tissue samples.

Table S6 msystems.00271-25-s0006.doc The abundance of the 20 specific species in both BC_tissue and BC_adjacent samples.

Table S7 msystems.00271-25-s0007.doc The abundance of the 20 specific species in both BC_tissue and normal_tissue samples.

Table S8 msystems.00271-25-s0008.doc The abundance of *Cutibacterium* and *C. acnes*.

Table S9 msystems.00271-25-s0009.doc Differential pathways between BC_tissue and BC_adjacent.

Table S10 msystems.00271-25-s0010.doc 16S rRNA sequencing result of *C. acnes* strains.

ASM does not own the copyrights to Supplemental Material that may be linked to, or accessed through, an article. The authors have granted ASM a non-exclusive, world-wide license to publish the Supplemental Material files. Please contact the corresponding author directly for reuse.
