# Supplementary material for: Microbial diagnostic features identified across populations possess potential antitumor properties in breast cancer
Source: mSystems. 2025 Jun 23;10(7):e00271-25. doi: 10.1128/msystems.00271-25 (PMC12282184; doi:10.1128/msystems.00271-25)
Supplement: Table S1 — Clinical characteristics of in-house cohort. [file msystems.00271-25-s0001.doc]

**Table S1. Clinical characteristics of in-house cohort.**

| **Characteristic** | **BC_tissue/BC_adjacent (n=10)** | **Benign_tissue (n=8)** |
| --- | --- | --- |
| Age (years) | 46 (37, 62) | 33 (25, 37) |
| BMI (kg/m^2^) | 22.1 (21.4, 23.7) | 21.7 (20.8, 22.3) |
| Lesion location (left/right) | 6/4 | 2/6 |
| Pathological type | Invasive ductal carcinoma (n=8) | Benign fibroadenoma (n=8) |
|  | Invasive lobular carcinoma (n=1) |  |
|  | Mixed (n=1) |  |
| TNM stage (I/II/III) | 4/3/3 | - |
| Molecular type (Luminal/Her-2/TNBC) | 5/4/1 | - |
