# Supplementary material for: Microbial diagnostic features identified across populations possess potential antitumor properties in breast cancer
Source: mSystems. 2025 Jun 23;10(7):e00271-25. doi: 10.1128/msystems.00271-25 (PMC12282184; doi:10.1128/msystems.00271-25)
Supplement: Table S2 — The abundance of BC_tissue, BC_adjacent, and normal_tissue. [file msystems.00271-25-s0002.doc]

**Table S2A. The abundance of BC_tissue, BC_adjacent, and normal_tissue at phylum level.**

| **Phylum** | **BC_tissue** | **BC_adjacent** | **normal_tissue** |
| --- | --- | --- | --- |
| *Proteobacteria* | 26.45766926 | 29.08150615 | 17.58291441 |
| *Firmicutes_D* | 14.02180519 | 14.28223487 | 11.92935831 |
| *Actinobacteriota* | 6.182937229 | 10.98004 | 4.941643237 |
| *Bacteroidota* | 17.82775281 | 3.241307179 | 1.637353659 |
| *Firmicutes_A* | 9.706405628 | 2.536847692 | 0.622464523 |
| *Patescibacteria* | 0.724878788 | 0.951101538 | 0.646414634 |
| *Acidobacteriota* | 0.888849351 | 0.753353846 | 0.019159424 |
| *Planctomycetota* | 0.792038095 | 0.766621538 | 0.045615965 |
| *Chloroflexota* | 0.568330303 | 0.54674359 | 0.033574945 |
| *Firmicutes_C* | 0.283719913 | 0.282744615 | 0.086990909 |
| *Cyanobacteria* | 0.163390043 | 0.213987179 | 0.146399113 |
| *Deinococcota* | 0.114334199 | 0.257221538 | 0.110482262 |
| *Gemmatimonadota* | 0.267159307 | 0.206252821 | 0.01300643 |
| *Desulfobacterota_I* | 0.389432035 | 0.056201538 | 0.004055876 |
| *Fusobacteriota* | 0.184063636 | 0.113111282 | 0.053017295 |
| *Verrucomicrobiota* | 0.16587316 | 0.109485128 | 0.011776053 |
| *Desulfobacterota_B* | 0.146672294 | 0.137806154 | 0.001667849 |
| *Myxococcota_A_473307* | 0.114574892 | 0.149270256 | 0.011463193 |
| *Campylobacterota* | 0.132185281 | 0.069098974 | 0.010618847 |
| *Nitrospirota_A_437815* | 0.1137 | 0.083182564 | 0.007852993 |
| *Myxococcota_A_437813* | 0.04121039 | 0.0379 | 0.019563858 |
| *Firmicutes_B_370529* | 0.097730736 | 5.13E-06 | 0 |
| *Bdellovibrionota_E* | 0.025407792 | 0.018928205 | 0.028196231 |
| *Methylomirabilota* | 0.055522944 | 0.046211795 | 0.000179157 |
| *Spirochaetota* | 0.057976623 | 0.018270769 | 0.002127273 |
| *Firmicutes_B_370539* | 0.051914719 | 0.002863077 | 0.000860532 |
| *Dependentiae* | 0.028049351 | 0.002213333 | 0.007921729 |
| *Synergistota* | 0.005912554 | 0.022866667 | 9.11E-05 |
| *Armatimonadota* | 0.009930736 | 0.009201538 | 0.001026829 |
| *Chlamydiota* | 0.006298701 | 0.009867179 | 0.001800222 |
| *Tectomicrobia* | 0.011308225 | 0.004903077 | 0 |
| *Elusimicrobiota* | 0.009886147 | 0.004445128 | 2.59E-05 |
| *Deferribacterota* | 0.012835498 | 0 | 0.000385144 |
| *Krumholzibacteriota* | 0.00697013 | 0.007567179 | 8.05E-05 |
| *Eisenbacteria* | 0.007974459 | 0.002484615 | 5.59E-05 |
| *Bdellovibrionota_C* | 0.002871861 | 0.002046667 | 0.002587361 |
| *Fibrobacterota* | 0.007139394 | 0.000254359 | 0.000544346 |
| *Eremiobacterota* | 0.00077619 | 0.000713333 | 0.00218204 |
| *Methanobacteriota_A_1229* | 0 | 0.001187179 | 0.002259645 |
| *Nanoarchaeota* | 0.005221212 | 0 | 0 |
| *UBA10199* | 0.002746753 | 0.000724615 | 0.000573614 |
| *Desulfobacterota_G_459546* | 0.003177922 | 0 | 0.000410865 |
| *Thermoproteota* | 0.002364502 | 0.001136923 | 7.69E-05 |
| *Omnitrophota* | 0.002122944 | 0.001337949 | 3.59E-05 |
| *Firmicutes_G* | 0.002438961 | 9.74E-06 | 4.88E-06 |
| *Thermosulfidibacterota* | 0.001938095 | 0 | 1.44E-05 |
| *Aquificota* | 0 | 0.000754359 | 0.000413304 |
| *Desulfobacterota_G_459544* | 0.000720346 | 0.000606154 | 0 |
| *Firmicutes_B_370525* | 0 | 0 | 0.000408647 |
| *Halobacteriota* | 0 | 0 | 0.000407095 |
| *KSB1* | 0 | 0 | 0.000389357 |
| *Atribacterota* | 0 | 0 | 0.00024235 |

**Table S2B. The abundance of BC_tissue, BC_adjacent, and normal_tissue at the phylum level (top 50).**

| **Genus** | **BC_tissue** | **BC_adjacent** | **normal_tissue** |
| --- | --- | --- | --- |
| *Staphylococcus* | 6.13030303 | 6.253714872 | 4.121263415 |
| *Cutibacterium* | 1.24305671 | 4.539792821 | 1.587169623 |
| *Acinetobacter* | 3.318934199 | 2.503905641 | 0.526151885 |
| *Burkholderia* | 0.849035931 | 1.523746667 | 2.20164745 |
| *Prevotella* | 4.954682251 | 0.721426667 | 0.123960089 |
| *Pseudomonas_E_647464* | 2.92327013 | 0.658606154 | 0.893163858 |
| *Corynebacterium* | 0.968716883 | 1.420824615 | 1.406307539 |
| *Streptococcus* | 1.34247013 | 1.509393333 | 1.055605543 |
| *Ralstonia* | 0.702689177 | 2.448096923 | 0.87953592 |
| *Acetobacter* | 0.121663203 | 0.242850769 | 1.967360089 |
| *Pseudomonas_O_647615* | 1.289809091 | 1.255627692 | 0.531525055 |
| *Pseudomonas_E_650326* | 1.273981385 | 2.322526667 | 0.007134146 |
| *Lactobacillus* | 2.587444156 | 0.21693641 | 0.163082483 |
| *Liquorilactobacillus* | 0.007050649 | 0.01247641 | 1.499493348 |
| *Phocaeicola_A_858004* | 2.830455844 | 0.029994872 | 0.023905543 |
| *Pseudomonas_E_648040* | 1.090071861 | 1.629467692 | 0.174652993 |
| *Alloprevotella* | 2.439974459 | 0.036075897 | 0.009311973 |
| *JC017* | 0.565317749 | 0.816755385 | 0.533350333 |
| *GWA2-37-10* | 0.456811255 | 0.679625128 | 0.523848337 |
| *Bacteroides_H* | 1.306454545 | 0.220010769 | 0.058647894 |
| *Lacticaseibacillus* | 0.020636797 | 0.092883077 | 0.748683814 |
| *Lentilactobacillus* | 0.044515584 | 0.014230769 | 0.766170288 |
| *Bacillus_J* | 0.10428961 | 0.341730256 | 0.55068847 |
| *Tepidimonas* | 0.810177489 | 0.520368205 | 0.091332816 |
| *Bradyrhizobium* | 0.061386147 | 0.098231795 | 0.632065854 |
| *Schleiferilactobacillus* | 0.000481818 | 0.00936359 | 0.673978492 |
| *Paracoccus* | 0.267669697 | 0.293408205 | 0.401610865 |
| *Atopostipes* | 0.249142857 | 0.961047692 | 0.015099113 |
| *Iodobacter* | 7.62E-05 | 0.000202051 | 0.557292905 |
| *Rothia* | 0.386645887 | 0.577301538 | 0.075374501 |
| *Micrococcus* | 0.227310823 | 0.287953333 | 0.271996674 |
| *Cloacibacterium* | 0.221458874 | 0.326486667 | 0.257059424 |
| *Comamonas_F_589250* | 0.461863636 | 0.361175385 | 0.104272284 |
| *Enterococcus_E* | 0.041522511 | 0.096930769 | 0.419986475 |
| *Brevundimonas* | 0.365541991 | 0.228897436 | 0.193522395 |
| *Anaerococcus* | 0.328835065 | 0.552648718 | 0.065268293 |
| *Rubrobacter_B_405439* | 0.241522511 | 0.567305641 | 0.098092461 |
| *Methylobacterium* | 0.260157143 | 0.257635897 | 0.212550333 |
| *Oceanobacillus* | 0.351262338 | 0.630013846 | 0.004393792 |
| *Escherichia_710834* | 0.567112987 | 0.327863077 | 0.024305543 |
| *Massilia* | 0.308365368 | 0.130728718 | 0.222620177 |
| *Bifidobacterium_388775* | 0.386532468 | 0.165587179 | 0.162642572 |
| *Moraxella_A_651124* | 0.159081385 | 0.253266154 | 0.233062749 |
| *Acidovorax_A* | 0.354880952 | 0.434141538 | 0.027935477 |
| *Pelomonas* | 0.051872294 | 0.090377949 | 0.330484479 |
| *Pseudogracilibacillus* | 0.35391039 | 0.494819487 | 0.000242129 |
| *Psychrobacter* | 0.487703896 | 0.315269744 | 0.001997783 |
| *Sporosarcina* | 0.359806494 | 0.423164615 | 0.000526164 |
| *Duganella_571129* | 0.09902684 | 0.475197436 | 0.104554102 |
| *Neisseria_563205* | 0.195592208 | 0.390169744 | 0.090454324 |
