# Supplementary material for: Microbial diagnostic features identified across populations possess potential antitumor properties in breast cancer
Source: mSystems. 2025 Jun 23;10(7):e00271-25. doi: 10.1128/msystems.00271-25 (PMC12282184; doi:10.1128/msystems.00271-25)
Supplement: Table S3 — The characteristics of important bacterial features at the genus level compared between BC_tissue and BC_adjacent. [file msystems.00271-25-s0003.doc]

**Table S3A. The characteristics of important bacterial features at the genus level were compared between BC_tissue and BC_adjacent in the study by Esposito_2022.**

| **Genus** | **BC_adjacent** | **BC_tissue** |
| --- | --- | --- |
| *Acinetobacter* | 11.40798529 | 20.34736765 |
| *Cutibacterium* | 22.55283235 | 5.73700882 |
| *Pseudomonas_E_650326* | 3.73920294 | 2.16583824 |
| *Rubrobacter_B_405439* | 3.23861471 | 1.58995588 |
| *Xanthomonas_A_614439* | 2.17834412 | 0.84654118 |
| *Pseudomonas_B_650451* | 2.29852647 | 0.00104412 |
| *Serratia_D_727245* | 0.46671176 | 1.78629412 |
| *Neisseria_563205* | 1.21551765 | 0.53697353 |
| *Cupriavidus* | 0.9118 | 0.75840588 |
| *Pseudomonas_A* | 1.18353529 | 0.46107059 |
| *Haemophilus_A* | 1.33992941 | 0.06571176 |
| *Ralstonia* | 1.05915294 | 0.09997647 |
| *Hydrogenophilus* | 0.88232353 | 0.18076176 |
| *Escherichia_710834* | 0.33886176 | 0.53870294 |
| *Novosphingobium_485351* | 0.02142647 | 0.67125882 |
| *Sphingobium_A_485959* | 0.01015588 | 0.66159706 |
| *Agrobacterium* | 0.2323 | 0.19075882 |
| *Lawsonella* | 0.21219118 | 0.14624118 |
| *Phocaeicola_A_858004* | 0 | 0.35619118 |
| *Pedobacter_887417* | 0.30770588 | 0.01897941 |
| *Luteimonas_C_615545* | 0.03211765 | 0.28007647 |
| *Nocardioides_A_392796* | 0.24836176 | 0.06280294 |
| *Haliscomenobacter* | 0.29254412 | 0 |
| *Aeromonas* | 0.24455 | 0.00787647 |
| *Aliterella* | 0.21141176 | 0 |
| *Desulfovibrio_R_446353* | 0.14671765 | 0.05867353 |
| *Propionispira* | 0.20494706 | 0 |
| *Marihabitans_390548* | 0.12326176 | 0.05719706 |
| *Aggregatibacter_736122* | 0.16703235 | 0.00020588 |
| *Anaeromusa* | 0.12562353 | 0 |
| *Thermomonas_615664* | 0.08109412 | 0.01865882 |
| *Providencia_A_732258* | 0.09504706 | 0 |
| *Methyloversatilis* | 0.09240882 | 0.0019 |
| *Hydrogenophaga_590395* | 0.04260882 | 0.04173824 |
| *Cobetia* | 0.08033529 | 0 |
| *Lactococcus_A_343306* | 0.07883235 | 0 |
| *Algoriphagus* | 0.07861765 | 0 |
| *Tepidibaculum* | 0.07713529 | 0 |
| *Cytobacillus_298193* | 0 | 0.07477941 |
| *Anoxybacillus_A_295174* | 0 | 0.07431471 |
| *Pararheinheimera* | 0.06246471 | 0.00046471 |
| *Alcanivorax_A* | 0.06159118 | 0 |
| *Eisenbergiella* | 0.06060882 | 0 |
| *Rubrobacter_A_405431* | 0.05627353 | 0 |
| *Tepidiphilus* | 0.04458824 | 0.01064706 |
| *Massilibacillus* | 0.04354412 | 0 |
| *Phreatobacter* | 0.03598824 | 0 |
| *Brachybacterium* | 0 | 0.02272353 |
| *Salinisphaera* | 0.01486471 | 0 |

**Table S3B. The characteristics of important bacterial features at the genus level were compared between BC_tissue and BC_adjacent in the study by Hoskinson_2022.**

| **Genus** | **BC_adjacent** | **BC_tissue** |
| --- | --- | --- |
| *Bradyrhizobium* | 0.30008776 | 0.02751522 |
| *Thermus_A* | 0.45084898 | 0.09602826 |
| *Cellulosimicrobium* | 0.00103265 | 0.00130217 |
| *Adhaeribacter* | 0 | 0.12019348 |
| *Desulfovibrio_R_446353* | 0.01990204 | 0 |
| *Paenibacillus_J_366884* | 0 | 0.01523043 |
| *Oligella* | 0.0011102 | 0.00460652 |

**Table S3C. The characteristics of important bacterial features at the genus level were compared between BC_tissue and BC_adjacent in the study by Kartti_2023.**

| **Genus** | **BC_adjacent** | **BC_tissue** |
| --- | --- | --- |
| *Psychrobacter* | 1.12854615 | 1.90937647 |
| *Nitrospira_C* | 0.17368077 | 0.2836098 |
| *Leuconostoc_B* | 0.22680192 | 0 |
| *Pseudomonas_O_647615* | 0.11549038 | 0.09531176 |
| *Blastococcus* | 0.13394038 | 0.0597549 |
| *Finegoldia* | 0.16580577 | 0.02514902 |
| *Mediterraneibacter_A_155507* | 0.01806731 | 0.0879902 |
| *Akkermansia* | 0.00758077 | 0.05630392 |
| *UBA10511* | 0.00439808 | 0.04692353 |
| *Coprococcus_A_187866* | 0.04585385 | 0.00027059 |
| *Hydrogenophilus* | 0.01860962 | 0.02337843 |
| *RF16* | 0.00846154 | 0.02793333 |
| *Novosphingobium_485350* | 0.00259231 | 0.02702941 |
| *Hymenobacter_910554* | 0.01239423 | 0.01376471 |
| *GCA-2746885* | 0 | 0.02350392 |
| *UBA737* | 0 | 0.0095549 |
| *Aliicoccus* | 0.00830577 | 0 |
| *Solitalea* | 0.00747308 | 0 |
| *Odoribacter_865974* | 0 | 0.00578235 |

**Table S3D. The characteristics of important bacterial features at the genus level were compared between BC_tissue and BC_adjacent in the study by German_2023.**

| **Genus** | **BC_adjacent** | **BC_tissue** |
| --- | --- | --- |
| *Acetobacter* | 0.61085167 | 0.93641 |
| *GWA2-37-10* | 2.20659333 | 3.50841333 |
| *Cloacibacterium* | 0.72573 | 0.31459333 |
| *Anoxybacillus_A_295174* | 0.120995 | 0.61824333 |
| *Finegoldia* | 0.06205 | 0.38866667 |
| *Veillonella_A* | 0.070715 | 0.42381667 |
| *Prevotella* | 0.04190333 | 0.34959333 |
| *Abiotrophia* | 0.01223167 | 0.19182 |
| *Hymenobacter_910554* | 0.02045167 | 2.00E-05 |
| *Sporosarcina* | 0.17899 | 0 |
| *Microvirga* | 0.007075 | 0 |
| *Pseudomonas_E_650326* | 0.05146 | 0.01370333 |
| *Capnocytophaga_820690* | 0.00226167 | 0.01690667 |
| *Haemophilus_D_736121* | 8.33E-06 | 0.01694333 |
| *Pleomorphomonas* | 0 | 0.00707333 |
| *Lancefieldella* | 0 | 0.01161667 |
| *Akkermansia* | 0 | 0.00036667 |
| *Bergeyella_A_791830* | 0 | 0.01124 |
| *Anaerobacillus* | 0 | 0.00317333 |
| *GWA1-52-35* | 0 | 0.002 |
| *Euzebya* | 0 | 0.01097667 |
| *Vogesella* | 0 | 0.00236667 |

**Table S3E. The characteristics of important bacterial features at the genus level were compared between BC_tissue and normal_tissue in the study by Hoskinson_2022.**

| **Genus** | **normal_tissue** | **BC_tissue** |
| --- | --- | --- |
| *Pseudomonas_E_647464* | 7.52998571 | 3.54453261 |
| *Pseudomonas_E_650326* | 0.00529592 | 4.70028478 |
| *Corynebacterium* | 5.87542653 | 2.38013261 |
| *Bradyrhizobium* | 5.19010816 | 0.02751522 |
| *Iodobacter* | 5.12936939 | 0.00038261 |
| *Atopostipes* | 0.03852449 | 1.1339413 |
| *Oceanobacillus* | 0.00013673 | 1.7201413 |
| *Pseudogracilibacillus* | 5.51E-05 | 1.77720217 |
| *Anaerococcus* | 0.04241837 | 1.14588261 |
| *Alcaligenes* | 0.00050204 | 1.28388696 |
| *Lactococcus_A_346120* | 0.28812041 | 0.17272609 |
| *Sporosarcina* | 8.78E-05 | 1.00478913 |
| *Lactobacillus* | 0.55246531 | 1.16828913 |
| *Herbaspirillum* | 3.67E-05 | 1.33937826 |
| *Pseudomonas_E_648040* | 1.37052245 | 0.01584565 |
| *Enterococcus_H_360604* | 0.00044694 | 0.51811087 |
| *Brevibacterium* | 0.03046531 | 0.26070217 |
| *Brevundimonas* | 0.03287959 | 0.81474783 |
| *Bacteroides_H* | 0.34907959 | 0.26901522 |
| *Neisseria_563205* | 0.35406327 | 0.05646957 |
| *Tissierella_B_224124* | 6.33E-05 | 0.57727826 |
| *Brochothrix* | 0.91313265 | 0 |
| *Thermus_A* | 0.35158163 | 0.09602826 |
| *Finegoldia* | 0.12785714 | 0.33069565 |
| *Moraxella_A_651124* | 0.06126735 | 0.40928043 |
| *Dermabacter* | 0.09261633 | 0.16253043 |
| *Rubrobacter_B_405439* | 0.65753673 | 3.91E-05 |
| *Brachybacterium* | 0.00021837 | 0.1310913 |
| *Achromobacter* | 0 | 0.01453478 |
| *Frederiksenia* | 0 | 0.00041957 |
| *Escherichia_710834* | 0.03641837 | 0.13976087 |
| *Akkermansia* | 0.0014102 | 0.26887174 |
| *Carnobacterium_A_320617* | 0.37337347 | 3.91E-05 |
| *Lacticaseibacillus* | 0 | 0.04313043 |
| *Aerococcus* | 0 | 0.0005913 |
| *Rhodococcus_B* | 0 | 0.20733696 |
| *Bacillus_A* | 0 | 0.27434783 |
| *Bulleidia* | 0.00011224 | 0.21833043 |
| *Catonella* | 0.0001551 | 0.15198478 |
| *Lagierella* | 0 | 0.16891304 |
| *Conchiformibius* | 0 | 0.00016739 |
| *Cellulosimicrobium* | 0.13796531 | 0.00130217 |
| *Caulobacter_487784* | 0.13234694 | 0 |
| *Faecousia* | 0.00022245 | 0 |
| *Cloacibacterium* | 0 | 0.11403696 |
| *Pseudochrobactrum* | 0 | 0.06534348 |
| *Ureaplasma* | 0.00090612 | 0.03011304 |
| *Parabacteroides_B_862066* | 0.00327551 | 0.00202826 |
| *Pseudomonas_C* | 0.0004898 | 0.05091739 |
| *Clostridium_T* | 0.00167551 | 0.0011413 |
| *Ligilactobacillus* | 0.00851224 | 0.00816522 |
| *Erysipelatoclostridium* | 0.00149796 | 0.00044783 |
| *Morganella* | 0.00013265 | 8.00E-04 |
| *Eremococcus* | 0.00030204 | 0.00188261 |
| *Bilophila* | 0.00590408 | 0.00040652 |
| *Dolosicoccus* | 0.00646939 | 0.00406957 |
| *Alistipes_A_871400* | 0.00269796 | 0.0010913 |
| *Dubosiella* | 0.00053061 | 0.00553261 |
| *Oligella* | 0.00222245 | 0.00460652 |
| *Cryptobacteroides* | 0 | 0.0066587 |

**Table S3F. The characteristics of important bacterial features at the genus level were compared between BC_tissue and normal_tissue in the study by German_2023.**

| **Genus** | **normal_tissue** | **normal_tissue** |
| --- | --- | --- |
| *Burkholderia* | 2.47000746 | 6.40509333 |
| *Ralstonia* | 0.98673333 | 5.18258333 |
| *Acetobacter* | 2.20716269 | 0.93641 |
| *Cutibacterium* | 1.71410075 | 1.58394333 |
| *Liquorilactobacillus* | 1.68226741 | 0.04620667 |
| *GWA2-37-10* | 0.58739826 | 3.50841333 |
| *Streptococcus* | 0.71797687 | 2.18406667 |
| *Lentilactobacillus* | 0.8595592 | 0.11004333 |
| *Lacticaseibacillus* | 0.83994129 | 0.09087 |
| *JC017* | 0.55195398 | 0.3751 |
| *Schleiferilactobacillus* | 0.7561301 | 0.00371 |
| *Paracoccus* | 0.41903532 | 0.04718333 |
| *Cloacibacterium* | 0.28839254 | 0.31459333 |
| *Micrococcus* | 0.25884179 | 0.32017333 |
| *Xanthomonas_B* | 0.29781841 | 0 |
| *Methylobacterium* | 0.2218694 | 0.08551333 |
| *Lactobacillus* | 0.1156204 | 0.23878667 |
| *Anoxybacillus_A_295174* | 0.12482711 | 0.61824333 |
| *Cytobacillus_298193* | 0.1543398 | 0.01536333 |
| *Rothia* | 0.06570149 | 0.59724 |
| *Tepidimonas* | 0.10246095 | 0.04289 |
| *Neisseria_563205* | 0.05832289 | 0.68622 |
| *Finegoldia* | 0.05748781 | 0.38866667 |
| *Agrobacterium* | 0.09033035 | 0.00449333 |
| *Veillonella_A* | 0.04767687 | 0.42381667 |
| *Kocuria* | 0.06076368 | 0.13673667 |
| *Pediococcus* | 0.08257189 | 9.67E-05 |
| *Gemella* | 0.04494403 | 0.21744 |
| *Microbacterium_A_383312* | 0.04873109 | 0.02707 |
| *Prevotella* | 0.04276443 | 0.34959333 |
| *Tepidiphilus* | 0.04919701 | 0.11265333 |
| *Granulicatella* | 0.04235821 | 0.23611 |
| *Curtobacterium* | 0.06333308 | 0.01096667 |
| *Saccharimonas* | 0.05462413 | 0.01112333 |
| *Lawsonella* | 0.03786567 | 0.10734333 |
| *Haemophilus_D_735815* | 0.02992687 | 0.18229333 |
| *Proteus* | 0.00153358 | 0.00108333 |
| *Acidipropionibacterium* | 0.0427796 | 0.00012333 |
| *Ochrobactrum_A_498953* | 0.04261667 | 0 |
| *Noviherbaspirillum_A_569121* | 0.01092289 | 0.00326667 |
| *Abiotrophia* | 0.02361468 | 0.19182 |
| *Brucella_499025* | 0.03706716 | 0 |
| *Novosphingobium_485351* | 0.0326791 | 0.00503667 |
| *Ochrobactrum_A_499024* | 0.03428507 | 5.67E-05 |
| *Hymenobacter_910554* | 0.03152438 | 2.00E-05 |
| *Thermus_A* | 0.03298582 | 0.00141667 |
| *Asaia* | 0.03073582 | 0.00714333 |
| *Propionibacterium* | 0.03061965 | 0 |
| *Bacillus_P_294101* | 0.02181219 | 0 |
| *Clostridium_T* | 0.02704104 | 0.00051 |
| *Bosea* | 0.02636542 | 0.00154 |
| *Gluconacetobacter* | 0.02541393 | 0.00915333 |
| *Paenibacillus_J_366884* | 0.02576119 | 0 |
| *Escherichia_710834* | 0.0228291 | 0.00035667 |
| *Aureimonas_A_501505* | 0.02018308 | 0 |
| *Starkeya* | 0.01821542 | 0 |
| *Haemophilus_A* | 0.00759677 | 0.08086667 |
| *Roseomonas_A_507160* | 0.01677711 | 0.00039333 |
| *Metabacillus_B_289979* | 0.01636318 | 0 |
| *Janibacter_A_390549* | 0.00946144 | 0.08358333 |
| *Gulosibacter* | 0.01089328 | 0.00013333 |
| *Arachnia* | 0.00404677 | 0.09323333 |
| *Paenirhodobacter_493284* | 0.01213284 | 0 |
| *Angulomicrobium* | 0.01178657 | 0 |
| *Duganella_571129* | 3.04E-05 | 0.01236 |
| *Roseomonas_A_507136* | 0.00029428 | 0.01439 |
| *Planifilum* | 0.00398458 | 0.02156 |
| *Metabacillus_B_289500* | 0.007001 | 0 |
| *Gluconobacter_C* | 0.00683383 | 0 |
| *Kaistia* | 0.00911517 | 0 |
| *Chryseobacterium_796661* | 0.00872836 | 0.00065333 |
| *Enterococcus_H_360604* | 0.00032512 | 0.06633 |
| *Aliihoeflea_498503* | 0.0089694 | 0 |
| *Enterococcus_A* | 0.00031741 | 0.05145333 |
| *Neorhizobium* | 0.00757886 | 0 |
| *Phenylobacterium* | 0.00377214 | 0.00119 |
| *Pseudoclavibacter_A_383750* | 0.00755697 | 0 |
| *Rubellimicrobium* | 0.00073607 | 0.01925 |
| *Bifidobacterium_387352* | 0.00636244 | 0 |
| *Enterobacter_B_681665* | 0.00527836 | 0.00046333 |
| *Dermabacter* | 1.74E-06 | 0.01076333 |
| *Haematobacter* | 0.00413159 | 0 |
| *QHXM01* | 0.00020871 | 0.04334667 |
| *Paenibacillus_J_366882* | 0.00303806 | 0.00484 |
| *Mogibacterium* | 0.00014677 | 0.01817 |
| *Leptotrichia_A_993641* | 0.00232164 | 0.00848 |
| *Mobiluncus* | 0 | 0.03952 |
| *Alsobacter* | 0 | 6.00E-05 |
| *SZUA-47* | 0.00029751 | 2.33E-05 |
| *S5-A14a* | 0.00055522 | 0.01037 |
| *Spongiiferula* | 0 | 0.00019667 |
| *Lagierella* | 0 | 0.02446667 |
| *Centipeda* | 0.00109453 | 0.00901333 |
| *Halomonas_C_640386* | 0.00058284 | 0.01063667 |
| *Pseudomonas_D_641376* | 0.0016408 | 0.00017667 |
| *Urinicoccus* | 0.00033856 | 0.01426667 |
| *Stomatobaculum* | 0.00041194 | 0.00573333 |
| *SCN-70-22* | 0 | 0.00327333 |
| *Bergeyella_A_791830* | 0.00047413 | 0.01124 |
| *Wolbachia* | 5.30E-05 | 0.01644667 |
| *Alterileibacterium* | 0 | 0.01540667 |
| *Aestuariivirga* | 0.00071866 | 0.00559333 |
| *Anaerobacillus* | 0.00088408 | 0.00317333 |
| *Macrococcus_B* | 0.00070771 | 0.00069333 |
| *Dyella_B* | 0.00103731 | 0.00016667 |
| *Nanoperiomorbus* | 8.23E-05 | 0.01217333 |
| *GWA1-52-35* | 0.000801 | 0.002 |
| *Catonella* | 0.00051617 | 0.00519 |
| *UBA1436* | 0 | 0.01249667 |
| *Euzebya* | 0 | 0.01097667 |
| *Peptoniphilus_B_226777* | 0.00025348 | 0.00389667 |
| *Neomicrococcus* | 0.00055423 | 0.00301 |
| *Parageobacillus_294456* | 0 | 0.00114 |
| *Lachnospira* | 0 | 0.00878 |
| *Enterococcus_B* | 0 | 0.00078667 |
| *F0058* | 0 | 0.00671 |
| *Vogesella* | 0.00027114 | 0.00236667 |
| *Stenotrophobacter_427736* | 0 | 7.33E-05 |
| *Pseudactinotalea* | 0.00027886 | 8.00E-05 |
| *HOT-345* | 1.22E-05 | 0.00428 |
| *Solibacillus* | 0 | 0.00361333 |

**Table S3G. The characteristics of important bacterial features at the species level were compared between BC_tissue and BC_adjacent in the study by Esposito_2022.**

| **Species** | **BC_adjacent** | **BC_tissue** |
| --- | --- | --- |
| *Cutibacterium_acnes* | 22.12320882 | 5.68036765 |
| *Acinetobacter_johnsonii* | 6.74608529 | 14.43957353 |
| *Rubrobacter_B_405439_xylanophilus* | 3.21510588 | 1.58403235 |
| *Pseudomonas_B_650451_rhizoryzae* | 2.29826471 | 0.00104412 |
| *Neisseria_sp000186165* | 1.02379412 | 0.53697353 |
| *Haemophilus_A_sputorum* | 1.33992941 | 0.06571176 |
| *Ralstonia_pickettii_B* | 1.05740294 | 0.09997647 |
| *Hydrogenophilus_hirschii* | 0.85982941 | 0.18076176 |
| *Tepidimonas_fonticaldi* | 0.42332059 | 0.23352353 |
| *Sphingobium_A_485959_yanoikuyae* | 0.01015588 | 0.64140882 |
| *Stenotrophomonas_A_615274_acidaminiphila* | 0.19027059 | 0.1904 |
| *Luteimonas_C_615545_padinae* | 0.03211765 | 0.28007647 |
| *Pedobacter_nyackensis* | 0.30060294 | 0 |
| *Phocaeicola_A_858004_vulgatus* | 0 | 0.29789118 |
| *Haliscomenobacter_hydrossis* | 0.29254412 | 0 |
| *Sphingobacterium_endophyticum* | 0.20577059 | 0.00654706 |
| *Aliterella_sp000332075* | 0.21 | 0 |
| *Propionispira_arcuata* | 0.20494706 | 0 |
| *Comamonas_F_589250_denitrificans* | 0.16093824 | 0.02320588 |
| *Neisseria_elongata* | 0.1572 | 0 |
| *Atopostipes_suicloacalis* | 0.12645 | 0.01874118 |
| *Anaeromusa_acidaminophila* | 0.12562353 | 0 |
| *Desulfovibrio_R_446353_desulfuricans_A_446139* | 0.09488529 | 0 |
| *Methyloversatilis_universalis* | 0.09240882 | 0.0019 |
| *Cobetia_crustatorum* | 0.08033529 | 0 |
| *Lactococcus_A_343306_raffinolactis* | 0.07812941 | 0 |
| *Tepidibaculum_saccharolyticum* | 0.07713529 | 0 |
| *Pararheinheimera_tangshanensis* | 0.06246471 | 0.00046471 |
| *Tepidiphilus_succinatimandens* | 0.04458824 | 0.01064706 |
| *Rubrobacter_A_405431_bracarensis* | 0.04939412 | 0 |
| *Massilibacillus_massiliensis* | 0.04354412 | 0 |

**Table S3H. The characteristics of important bacterial features at the species level were compared between BC_tissue and BC_adjacent in the study by Hoskinson_2022.**

| **Species** | **BC_adjacent** | **BC_tissue** |
| --- | --- | --- |
| *Thermus_A_scotoductus* | 0.45084898 | 0.09602826 |
| *Lactobacillus_crispatus* | 0.0161449 | 0.2737 |
| *Neisseria_cinerea* | 0.29636122 | 0 |
| *Acinetobacter_towneri_A* | 0 | 0.05963043 |

**Table S3I. The characteristics of important bacterial features at the species level were compared between BC_tissue and BC_adjacent in the study by Kartti_2023.**

| **Species** | **BC_adjacent** | **BC_tissue** |
| --- | --- | --- |
| *Psychrobacter_maritimus* | 1.12228654 | 1.89290392 |
| *Acinetobacter_albensis* | 0.11675192 | 0.16240784 |
| *Pseudomonas_O_647615_parafulva* | 0.11549038 | 0.09531176 |
| *Acinetobacter_harbinensis* | 0.08098462 | 0.12948039 |
| *Finegoldia_magna_H* | 0.16580577 | 0.02514902 |
| *Blastococcus_aggregatus* | 0.11814808 | 0.05583333 |
| *Mediterraneibacter_A_155507_torques* | 0.01268462 | 0.08506078 |
| *Collinsella_stercoris* | 0.01928269 | 0.07182745 |
| *Cryptobacteroides_sp902792815* | 0.00262115 | 0.04043333 |
| *Hydrogenophilus_hirschii* | 0.01860962 | 0.02337843 |
| *Coprococcus_A_187866_catus* | 0.04059038 | 0 |
| *Novosphingobium_capsulatum* | 0.00259231 | 0.02702941 |
| *GCA-2746885_sp002746885* | 0 | 0.02350392 |
| *Bifidobacterium_thermophilum* | 0 | 0.01481373 |
| *UBA737_sp900547445* | 0 | 0.0095549 |
| *Aliicoccus_persicus* | 0.00830577 | 0 |
| *Odoribacter_splanchnicus* | 0 | 0.00578235 |

**Table S3J. The characteristics of important bacterial features at the species level were compared between BC_tissue and BC_adjacent in the study by German_2023.**

| **Species** | **BC_adjacent** | **BC_tissue** |
| --- | --- | --- |
| *Finegoldia_magna_H* | 0.06205 | 0.38866667 |
| *Liquorilactobacillus_ghanensis* | 0.00744 | 0.03828 |
| *Veillonella_A_rogosae* | 0.01448 | 0.23264333 |
| *Abiotrophia_defectiva* | 0.01223167 | 0.19182 |
| *Bifidobacterium_animalis* | 0 | 0.00073667 |
| *Bifidobacterium_longum* | 0.00256333 | 0.00288 |
| *Streptococcus_gordonii* | 0.00302167 | 0.0549 |
| *Prevotella_pallens* | 0 | 0.05875667 |
| *Streptococcus_parasanguinis* | 0.00628167 | 0.06494333 |
| *Neisseria_oralis* | 0.00239833 | 0.03019667 |
| *Prevotella_oris* | 0.00407333 | 0.03637667 |
| *Janibacter_A_390549_hoylei* | 0.00065167 | 0.02509 |
| *Pleomorphomonas_oryzae* | 0 | 0.00707333 |
| *Capnocytophaga_gingivalis* | 0 | 0.00864667 |
| *Methylobacterium_cerastii* | 0 | 0.00332333 |
| *Akkermansia_muciniphila_D_776786* | 0 | 0.00036667 |
| *Streptococcus_australis* | 0.00041333 | 0.00972333 |
| *Capnocytophaga_sputigena* | 0 | 0.00401667 |
| *Prevotella_jejuni* | 0 | 0.02231667 |
| *Bergeyella_A_791830_cardium* | 0 | 0.01124 |
| *Capnocytophaga_ochracea* | 0 | 0.01031 |
| *GWA1-52-35_sp001769805* | 0 | 0.002 |
| *Anaerobacillus_isosaccharinicus* | 0 | 0.00317333 |
| *Veillonella_A_montpellierensis* | 0 | 0.00509333 |
| *Lancefieldella_sp000564995* | 0 | 0.0036 |

**Table S3K. The characteristics of important bacterial features at the species level were compared between BC_tissue and normal_tissue in the study by Hoskinson_2022.**

| **Species** | **normal_tissue** | **BC_tissue** |
| --- | --- | --- |
| *Pseudomonas_E_650326_aeruginosa_A* | 0.00529592 | 4.70016957 |
| *Atopostipes_suicloacalis* | 0.03793265 | 0.43406522 |
| *Oceanobacillus_luteolus* | 1.00E-04 | 1.70796087 |
| *Alcaligenes_faecalis_595233* | 0.00050204 | 1.28388696 |
| *Herbaspirillum_huttiense* | 3.67E-05 | 1.33926304 |
| *Enterococcus_H_360604_faecalis* | 0.00044694 | 0.51811087 |
| *Acinetobacter_harbinensis* | 1.03989184 | 0.02691304 |
| *Tissierella_B_224124_carlieri* | 6.33E-05 | 0.57727826 |
| *Brochothrix_thermosphacta* | 0.91313265 | 0 |
| *Thermus_A_scotoductus* | 0.35158163 | 0.09602826 |
| *Finegoldia_magna_H* | 0.12785714 | 0.33069565 |
| *Lactobacillus_crispatus* | 0.53892857 | 0.2737 |
| *Moraxella_A_651124_cinereus* | 0.06126735 | 0.40925217 |
| *Dermabacter_vaginalis* | 0.09261633 | 0.16253043 |
| *Anaerococcus_vaginalis* | 0.02407551 | 0.5348087 |
| *Rubrobacter_B_405439_xylanophilus* | 0.65753673 | 3.91E-05 |
| *Brevundimonas_diminuta* | 0.00866531 | 0.47920217 |
| *Neisseria_cinerea* | 0.18000204 | 0 |
| *Escherichia_coli* | 0.03641837 | 0.13976087 |
| *Akkermansia_muciniphila_D_776786* | 0.0014102 | 0.26887174 |
| *Carnobacterium_A_320617_maltaromaticum* | 0.37337347 | 3.91E-05 |
| *Lacticaseibacillus_paracasei* | 0 | 0.04313043 |
| *Sphingomonas_L_486704_aerolata* | 4.49E-05 | 0.14633261 |
| *Rhodococcus_B_cerastii* | 0 | 0.20733696 |
| *Phocaeicola_A_858004_vulgatus* | 0.16567143 | 0.06772609 |
| *Bulleidia_moorei* | 0.00011224 | 0.21833043 |
| *Catonella_morbi* | 7.55E-05 | 0.15183261 |
| *Lagierella_massiliensis* | 0 | 0.16891304 |
| *Conchiformibius_steedae* | 0 | 0.00016739 |
| *Caulobacter_vibrioides_C_487720* | 0.13234694 | 0 |
| *Faecousia_sp000434635* | 0.00022245 | 0 |
| *Pseudomonas_E_647464_bubulae* | 0.11629592 | 0 |
| *Corynebacterium_matruchotii* | 0.08213265 | 0.0020413 |
| *Oceanobacillus_indicireducens* | 0 | 0.01183043 |
| *Pseudochrobactrum_asaccharolyticum* | 0 | 0.06534348 |
| *Ureaplasma_sp900544585* | 0.00090612 | 0.03011304 |
| *Acinetobacter_towneri_A* | 0 | 0.05963043 |
| *Pseudomonas_C_sp002342705* | 0.0004898 | 0.05091739 |
| *Clostridium_T_beijerinckii* | 0.00167551 | 0.0011413 |
| *Ligilactobacillus_apodemi* | 0.00851224 | 0.00816522 |
| *Morganella_morganii_731151* | 0.00013265 | 8.00E-04 |
| *Bilophila_wadsworthia* | 0.00590408 | 0.00040652 |
| *Dolosicoccus_paucivorans* | 0.00646939 | 0.00406957 |
| *Bacteroides_H_uniformis* | 0.00078367 | 0.00050652 |
| *Pseudomonas_E_650326_aeruginosa_A* | 0.00529592 | 4.70016957 |
| *Atopostipes_suicloacalis* | 0.03793265 | 0.43406522 |
| *Oceanobacillus_luteolus* | 1.00E-04 | 1.70796087 |
| *Alcaligenes_faecalis_595233* | 0.00050204 | 1.28388696 |
| *Herbaspirillum_huttiense* | 3.67E-05 | 1.33926304 |
| *Enterococcus_H_360604_faecalis* | 0.00044694 | 0.51811087 |
| *Acinetobacter_harbinensis* | 1.03989184 | 0.02691304 |
| *Tissierella_B_224124_carlieri* | 6.33E-05 | 0.57727826 |
| *Brochothrix_thermosphacta* | 0.91313265 | 0 |
| *Thermus_A_scotoductus* | 0.35158163 | 0.09602826 |
| *Finegoldia_magna_H* | 0.12785714 | 0.33069565 |
| *Lactobacillus_crispatus* | 0.53892857 | 0.2737 |
| *Moraxella_A_651124_cinereus* | 0.06126735 | 0.40925217 |
| *Dermabacter_vaginalis* | 0.09261633 | 0.16253043 |
| *Anaerococcus_vaginalis* | 0.02407551 | 0.5348087 |
| *Rubrobacter_B_405439_xylanophilus* | 0.65753673 | 3.91E-05 |
| *Brevundimonas_diminuta* | 0.00866531 | 0.47920217 |
| *Neisseria_cinerea* | 0.18000204 | 0 |
| *Escherichia_coli* | 0.03641837 | 0.13976087 |
| *Akkermansia_muciniphila_D_776786* | 0.0014102 | 0.26887174 |
| *Carnobacterium_A_320617_maltaromaticum* | 0.37337347 | 3.91E-05 |
| *Lacticaseibacillus_paracasei* | 0 | 0.04313043 |
| *Sphingomonas_L_486704_aerolata* | 4.49E-05 | 0.14633261 |
| *Rhodococcus_B_cerastii* | 0 | 0.20733696 |
| *Phocaeicola_A_858004_vulgatus* | 0.16567143 | 0.06772609 |
| *Bulleidia_moorei* | 0.00011224 | 0.21833043 |
| *Catonella_morbi* | 7.55E-05 | 0.15183261 |
| *Lagierella_massiliensis* | 0 | 0.16891304 |
| *Conchiformibius_steedae* | 0 | 0.00016739 |
| *Caulobacter_vibrioides_C_487720* | 0.13234694 | 0 |
| *Faecousia_sp000434635* | 0.00022245 | 0 |
| *Pseudomonas_E_647464_bubulae* | 0.11629592 | 0 |
| *Corynebacterium_matruchotii* | 0.08213265 | 0.0020413 |
| *Pseudochrobactrum_asaccharolyticum* | 0 | 0.06534348 |
| *Ureaplasma_sp900544585* | 0.00090612 | 0.03011304 |
| *Acinetobacter_towneri_A* | 0 | 0.05963043 |
| *Pseudomonas_C_sp002342705* | 0.0004898 | 0.05091739 |
| *Clostridium_T_beijerinckii* | 0.00167551 | 0.0011413 |
| *Ligilactobacillus_apodemi* | 0.00851224 | 0.00816522 |
| *Morganella_morganii_731151* | 0.00013265 | 8.00E-04 |
| *Bilophila_wadsworthia* | 0.00590408 | 0.00040652 |
| *Dolosicoccus_paucivorans* | 0.00646939 | 0.00406957 |
| *Bacteroides_H_uniformis* | 0.00078367 | 0.00050652 |

**Table S3L. The characteristics of important bacterial features at the species level were compared between BC_tissue and normal_tissue in the study by German_2023.**

| **Species** | **normal_tissue** | **BC_tissue** |
| --- | --- | --- |
| *Cutibacterium_acnes* | 1.66616269 | 1.52256 |
| *Ralstonia_pickettii_B* | 0.63334129 | 3.64376 |
| *Liquorilactobacillus_vini* | 1.43916642 | 0.00024 |
| *JC017_sp004296775* | 0.55195398 | 0.3751 |
| *Acetobacter_garciniae* | 0.79292164 | 0.32784333 |
| *Lacticaseibacillus_paracasei* | 0.81613134 | 0.09087 |
| *Lentilactobacillus_buchneri* | 0.81669876 | 0.11004333 |
| *Burkholderia_lata* | 0.50589776 | 1.74714333 |
| *Burkholderia_cepacia_576714* | 0.5162403 | 1.13516333 |
| *Schleiferilactobacillus_harbinensis* | 0.74035473 | 0.00371 |
| *Burkholderia_mallei* | 0.41125647 | 0.85306333 |
| *Xanthomonas_B_massiliensis* | 0.29781841 | 0 |
| *Corynebacterium_kroppenstedtii* | 0.17309502 | 0.00637333 |
| *Liquorilactobacillus_mali* | 0.1308791 | 0.00254667 |
| *Cytobacillus_kochii* | 0.1114505 | 0.00074333 |
| *Finegoldia_magna_H* | 0.05748781 | 0.38866667 |
| *Tepidimonas_fonticaldi* | 0.07375647 | 0.02571667 |
| *Streptococcus_agalactiae* | 0.06613433 | 0.03411 |
| *Streptococcus_oralis_E_351036* | 0.05042114 | 0.09450333 |
| *Methylobacterium_fujisawaense* | 0.06445398 | 0.01164 |
| *Corynebacterium_pyruviciproducens* | 0.00155249 | 0.66384333 |
| *Sphingomonas_L_486704_aerolata* | 0.02705597 | 0.02211 |
| *Liquorilactobacillus_hordei* | 0.05913557 | 0.00514 |
| *Lactobacillus_iners* | 0.03693905 | 0.21136667 |
| *Saccharimonas_aalborgensis* | 0.05462413 | 0.01112333 |
| *Veillonella_A_rogosae* | 0.02595846 | 0.23264333 |
| *Ochrobactrum_A_498953_teleogrylli* | 0.04261667 | 0 |
| *Abiotrophia_defectiva* | 0.02361468 | 0.19182 |
| *Neisseria_sp000186165* | 0.01168756 | 0.33464667 |
| *Brucella_pituitosa* | 0.03706716 | 0 |
| *Ochrobactrum_A_499024_pseudogrignonensis* | 0.03428507 | 5.67E-05 |
| *Rothia_sp001808955* | 0.01500672 | 0.16103 |
| *Thermus_A_scotoductus* | 0.032899 | 0.00141667 |
| *Rothia_dentocariosa* | 0.01835348 | 0.12607667 |
| *Asaia_bogorensis* | 0.03073582 | 0.00714333 |
| *Propionibacterium_freudenreichii* | 0.03055274 | 0 |
| *Lactobacillus_acidophilus* | 0.00035672 | 0.00162667 |
| *Gordonia_B_376796_sputi* | 0.02657139 | 8.00E-05 |
| *Microbacterium_A_383312_petrolearium* | 0.0167592 | 0.01437333 |
| *Rothia_aeria* | 0.00885746 | 0.17707667 |
| *Acinetobacter_junii* | 0.02353955 | 0.00698 |
| *Lentilactobacillus_rapi* | 0.02461294 | 0 |
| *Paracoccus_aminophilus* | 0.02444701 | 0 |
| *Pediococcus_ethanolidurans* | 0.02277662 | 0 |
| *Proteus_terrae_730456* | 0 | 9.67E-05 |
| *Acidipropionibacterium_jensenii* | 0.02136816 | 0 |
| *Aureimonas_A_501505_altamirensis* | 0.01801692 | 0 |
| *Bifidobacterium_animalis* | 0.01936667 | 0.00073667 |
| *Clostridium_T_beijerinckii* | 0.01772313 | 0 |
| *Streptococcus_sanguinis_H* | 0.01007537 | 0.05502333 |
| *Bifidobacterium_longum* | 0.01772662 | 0.00288 |
| *Gluconacetobacter_liquefaciens* | 0.01820572 | 0.00027667 |
| *Pediococcus_damnosus* | 0.01796269 | 0 |
| *Starkeya_novella* | 0.01748905 | 0 |
| *Haemophilus_A_sputorum* | 0.00688209 | 0.07811667 |
| *Schleiferilactobacillus_perolens* | 0.01577438 | 0 |
| *Niallia_taxi* | 0.0144398 | 0.00204 |
| *Lentilactobacillus_sunkii* | 0.01437562 | 0 |
| *Gulosibacter_faecalis* | 0.0107908 | 0.00013333 |
| *Anaerococcus_octavius* | 0.00619453 | 0.04008 |
| *Lacticaseibacillus_rhamnosus* | 0.01248582 | 0 |
| *Streptococcus_infantarius* | 0.00881294 | 0.02111667 |
| *Methylobacterium_pseudosasicola* | 0.01116667 | 0.00844667 |
| *Angulomicrobium_tetraedrale* | 0.01178657 | 0 |
| *Prevotella_loescheii* | 0.00357637 | 0.08337 |
| *Streptococcus_gordonii* | 0.00675448 | 0.0549 |
| *Roseomonas_aquatica* | 0.00029428 | 0.01439 |
| *Metabacillus_B_289500_idriensis* | 0.007001 | 0 |
| *Lacticaseibacillus_pantheris* | 0.01026741 | 0 |
| *Prevotella_pallens* | 0.00553731 | 0.05875667 |
| *Streptococcus_parasanguinis* | 0.00374975 | 0.06494333 |
| *Enterococcus_H_360604_faecalis* | 0.00032512 | 0.06633 |
| *Capnocytophaga_leadbetteri* | 0.00174876 | 0.09060667 |
| *Aliihoeflea_sp000497755* | 0.0089694 | 0 |
| *Planifilum_fimeticola* | 0.00330746 | 0.00608333 |
| *Granulicatella_elegans* | 0.00631741 | 0.02517 |
| *Haemophilus_D_735815_parainfluenzae* | 0.00477935 | 0.02872 |
| *Myroides_phaeus* | 0 | 0.00066 |
| *Sphingomonas_L_486704_sp902506605* | 0.00723781 | 0.00015667 |
| *Arachnia_propionica* | 0.00108035 | 0.08385 |
| *Neisseria_oralis* | 0.00446269 | 0.03019667 |
| *Prevotella_oris* | 0.00360746 | 0.03637667 |
| *Sphingomonas_L_486704_leidyi* | 0.00641095 | 0 |
| *Enterococcus_A_avium* | 5.00E-07 | 0.03968333 |
| *Loigolactobacillus_coryniformis* | 0.00633856 | 0 |
| *Chryseobacterium_bovis* | 0.00590945 | 0.00065333 |
| *Enterobacter_B_681665_hormaechei_681663* | 0.00527836 | 0.00046333 |
| *Dermabacter_vaginalis* | 1.74E-06 | 0.01076333 |
| *Metabacillus_B_289979_sp002871465* | 0.00542562 | 0 |
| *Gemella_morbillorum* | 0.00209677 | 0.02065667 |
| *Corynebacterium_simulans* | 0.00037587 | 0.05247333 |
| *Bifidobacterium_psychraerophilum* | 0.00466791 | 0 |
| *Bulleidia_moorei* | 0.00060796 | 0.02198667 |
| *Haematobacter_massiliensis* | 0.00413159 | 0 |
| *Staphylococcus_saprophyticus* | 0.00369552 | 0.00104667 |
| *Corynebacterium_glucuronolyticum* | 0.00346095 | 0.00780333 |
| *Limosilactobacillus_reuteri* | 0.00151841 | 0.01934 |
| *QHXM01_sp003222945* | 0.00020871 | 0.04334667 |
| *Neorhizobium_galegae_500181* | 0.00359378 | 0 |
| *Ezakiella_coagulans* | 1.24E-05 | 0.04276 |
| *Prevotella_melaninogenica* | 0.0011893 | 0.02272333 |
| *Lentilactobacillus_parabuchneri* | 0.00322015 | 0 |
| *Paracoccus_marinus_A_493398* | 0.00303085 | 0 |
| *Leptotrichia_A_sp001274535* | 0.00232164 | 0.00848 |
| *S5-A14a_sp000758905* | 0.00055522 | 0.01037 |
| *Leuconostoc_B_inhae* | 0.00170846 | 0.006 |
| *Mogibacterium_diversum* | 8.48E-05 | 0.01817 |
| *Mobiluncus_curtisii* | 0 | 0.02897333 |
| *Haemophilus_A_paraphrohaemolyticus* | 0.00071468 | 0.00274667 |
| *Lagierella_massiliensis* | 0 | 0.02446667 |
| *Streptococcus_australis* | 0.00109627 | 0.00972333 |
| *Leptotrichia_A_993758_hofstadii* | 0.0006092 | 0.01040333 |
| *Veillonella_A_atypica* | 0.0007898 | 0.01186 |
| *Prevotella_jejuni* | 2.74E-06 | 0.02231667 |
| *Pseudomonas_D_641376_profundi* | 0.0016408 | 0.00017667 |
| *Novosphingobium_meiothermophilum* | 0.00125522 | 0 |
| *Stomatobaculum_longum* | 0.00041194 | 0.00573333 |
| *Halomonas_C_640386_sp000246875* | 0.0004393 | 0.01063667 |
| *Bergeyella_A_791830_cardium* | 0.00047413 | 0.01124 |
| *Capnocytophaga_ochracea* | 0.00049552 | 0.01031 |
| *Chryseobacterium_luteum* | 0.00124129 | 1.67E-05 |
| *Aestuariivirga_litoralis* | 0.00071866 | 0.00559333 |
| *Porphyromonas_A_859423_sp000768875* | 0 | 0.01486333 |
| *Urinicoccus_timonensis* | 0.00033856 | 0.00801 |
| *Cellulomonas_sp001429255* | 0.00101567 | 0.00012333 |
| *Lacunisphaera_limnophila* | 2.79E-05 | 0.01321 |
| *Nanosynbacter_sp010202645* | 0.00024055 | 0.00728667 |
| *SCN-70-22_sp001724275* | 0 | 0.00327333 |
| *GWA1-52-35_sp001769805* | 0.000801 | 0.002 |
| *Catonella_morbi* | 0.00051617 | 0.00519 |
| *Centipeda_periodontii* | 0.00020871 | 0.00638667 |
| *Weissella_A_338544_cibaria* | 0.00086915 | 0.00034667 |
| *Pauljensenia_hongkongensis* | 8.06E-05 | 0.00317 |
| *Peptoniphilus_B_226777_duerdenii* | 0.00025348 | 0.00389667 |
| *Neomicrococcus_aestuarii* | 0.00055423 | 0.00301 |
| *Belnapia_soli* | 0 | 9.00E-05 |
| *Belnapia_rosea* | 0 | 1.67E-05 |
| *Parageobacillus_thermantarcticus* | 0 | 0.00114 |
| *Porphyromonas_A_859426_somerae* | 0 | 0.00871 |
| *Prevotella_pleuritidis* | 0 | 0.00866667 |
| *Enterococcus_B_lactis* | 0 | 0.00078667 |
| *Arachnia_flavescens* | 0 | 0.00699 |
| *F0058_sp000163695* | 0 | 0.00671 |
| *Anaerobacillus_isosaccharinicus* | 0.00026045 | 0.00317333 |
| *Flavobacterium_macacae* | 0.00013905 | 0.00460667 |
| *Urinicoccus_sp001299455* | 0 | 0.00626 |
| *Leptotrichia_A_993758_hongkongensis* | 6.67E-05 | 0.00510667 |
| *Stenotrophobacter_terrae* | 0 | 7.33E-05 |
| *Lawsonibacter_sp000177015* | 9.78E-05 | 0.00406 |
| *Veillonella_A_montpellierensis* | 0 | 0.00509333 |
| *Flavobacterium_rivuli* | 2.09E-05 | 0.00049667 |
| *HOT-345_sp003260355* | 1.22E-05 | 0.00428 |
| *Solibacillus_silvestris* | 0 | 0.00361333 |
| *Lancefieldella_sp000564995* | 0 | 0.0036 |
