# Supplementary material for: Microbial diagnostic features identified across populations possess potential antitumor properties in breast cancer
Source: mSystems. 2025 Jun 23;10(7):e00271-25. doi: 10.1128/msystems.00271-25 (PMC12282184; doi:10.1128/msystems.00271-25)
Supplement: Table S4 — The abundance of the 20 specific genera in both BC_tissue and BC_adjacent samples. [file msystems.00271-25-s0004.doc]

**Table S4A. The abundance of the 20 specific genera in both BC_tissue and BC_adjacent samples.**

| **Group** | **Cohort** | ***Cutibacterium*** | ***Acinetobacter*** | ***Ralstonia*** | ***Pseudomonas_O_647615*** | ***Pseudomonas_E_650326*** | ***Phocaeicola_A_858004*** | ***GWA2-37-10*** | ***Bradyrhizobium*** | ***Cloacibacterium*** | ***Escherichia_710834*** | ***Psychrobacter*** | ***Sporosarcina*** | ***Neisseria_563205*** | ***Lawsonella*** | ***Finegoldia*** | ***Thermus_A*** | ***Veillonella_A*** | ***Hydrogenophilus*** | ***Blastococcus*** | ***Akkermansia*** |
| --- | --- | --- | --- | --- | --- | --- | --- | --- | --- | --- | --- | --- | --- | --- | --- | --- | --- | --- | --- | --- | --- |
| BC_tissue | Hoskinson_2022 | 0.3511 | 0 | 0.0211 | 0.0042 | 57.2836 | 0 | 0.1607 | 0 | 0 | 0.1734 | 0 | 0 | 0.0042 | 0.0423 | 0 | 0 | 0 | 0 | 0 | 0 |
| BC_adjacent | Hoskinson_2022 | 0.0055 | 0.0036 | 0 | 0.0055 | 0 | 0 | 0 | 0 | 0 | 0.8802 | 0 | 0 | 19.5171 | 0.0109 | 0 | 0 | 0 | 0 | 0 | 0.0055 |
| BC_adjacent | Hoskinson_2022 | 0.026 | 5.5848 | 0 | 0.0015 | 0 | 0.0031 | 0 | 0 | 0 | 0.026 | 0 | 0 | 0 | 0 | 10.4421 | 0 | 0 | 0 | 0 | 0.0092 |
| BC_tissue | Hoskinson_2022 | 0.0017 | 0.0017 | 0 | 0.5226 | 87.5714 | 0 | 0 | 0 | 0 | 0.0035 | 0 | 0 | 0 | 0.0017 | 0 | 0 | 0 | 0 | 0 | 0 |
| BC_adjacent | Hoskinson_2022 | 0.0677 | 0 | 0 | 0.0038 | 64.7434 | 0 | 0 | 0.5078 | 0 | 0.0056 | 0 | 0 | 0 | 0 | 1.9728 | 1.2657 | 0 | 0 | 0 | 0 |
| BC_adjacent | Hoskinson_2022 | 0.0195 | 0 | 0 | 0.1598 | 0.0013 | 3.2584 | 0 | 0 | 0 | 3.9392 | 0 | 0 | 0 | 0 | 0 | 0 | 0.5989 | 0 | 0 | 1.3564 |
| BC_adjacent | Hoskinson_2022 | 0.0043 | 0.2192 | 0 | 0.7656 | 0 | 0 | 0 | 0 | 0 | 3.505 | 0 | 0 | 0 | 0 | 1.0424 | 2.0329 | 0 | 0 | 0 | 0.0014 |
| BC_tissue | Hoskinson_2022 | 0.4019 | 0.0014 | 0 | 0.476 | 0.0014 | 0 | 0.0029 | 0 | 0 | 2.6353 | 0 | 0 | 0 | 0 | 0 | 0 | 0 | 0 | 0 | 0 |
| BC_tissue | Hoskinson_2022 | 0.7355 | 0.0014 | 0 | 2.9998 | 0.0014 | 0 | 0.0029 | 0 | 0 | 0.0087 | 0 | 0 | 0 | 0.0014 | 0 | 0 | 0 | 0 | 0 | 0.0087 |
| BC_adjacent | Hoskinson_2022 | 2.3862 | 0 | 0 | 20.4207 | 0 | 0 | 0.0015 | 0 | 0 | 0.0015 | 0 | 0 | 0 | 0 | 0 | 1.6905 | 0 | 0 | 0 | 0 |
| BC_tissue | Hoskinson_2022 | 0.6603 | 0 | 0 | 2.233 | 0 | 0 | 0.0192 | 0.0137 | 0.0192 | 1.7124 | 0 | 0.0027 | 0 | 1.748 | 0 | 0 | 0.0027 | 0 | 0 | 0.0438 |
| BC_adjacent | Hoskinson_2022 | 0.5662 | 0.0061 | 0 | 0.1431 | 0 | 0 | 0 | 0 | 0 | 0.002 | 0 | 0 | 0 | 1.071 | 0 | 1.3735 | 0 | 0 | 0 | 0.0061 |
| BC_tissue | Hoskinson_2022 | 1.0333 | 0 | 0 | 23.5042 | 0 | 0 | 0.0021 | 0 | 0 | 0 | 0 | 0 | 0 | 0 | 0 | 0 | 0 | 0 | 0 | 0 |
| BC_adjacent | Hoskinson_2022 | 0.5848 | 0.1263 | 0 | 9.0582 | 0.0058 | 0 | 0.0058 | 0 | 0 | 0 | 0 | 0 | 0 | 0.0019 | 0 | 0 | 0 | 0 | 0 | 0 |
| BC_tissue | Hoskinson_2022 | 1.2246 | 0.3238 | 0 | 0.003 | 0 | 0 | 0.003 | 0 | 0 | 0 | 0 | 0 | 0 | 2.3965 | 0 | 0 | 1.5002 | 0 | 0 | 0.0015 |
| BC_adjacent | Hoskinson_2022 | 6.4788 | 0 | 0 | 18.6199 | 0 | 0 | 0 | 0 | 0 | 0 | 0 | 0 | 0 | 8.8668 | 0 | 1.6042 | 0 | 0 | 0 | 0 |
| BC_tissue | Hoskinson_2022 | 2.2896 | 0 | 0 | 0 | 0 | 0.0014 | 0 | 0 | 0 | 0 | 0 | 0 | 0 | 0.0014 | 0 | 0 | 0 | 0 | 0 | 0 |
| BC_adjacent | Hoskinson_2022 | 0.4337 | 0 | 0 | 1.237 | 0 | 0 | 0 | 0 | 0 | 0 | 0 | 0 | 0 | 0.4507 | 6.2304 | 0 | 0 | 0 | 0 | 0 |
| BC_adjacent | Hoskinson_2022 | 1.2882 | 3.0811 | 0 | 1.5837 | 0 | 0 | 0 | 0.0055 | 0 | 1.4717 | 0 | 0 | 0 | 1.7892 | 1.2772 | 0.0018 | 0 | 0 | 0 | 0 |
| BC_adjacent | Hoskinson_2022 | 0.4092 | 5.9986 | 0 | 6.9208 | 0 | 0.0015 | 0.0015 | 0.0015 | 0 | 0.0015 | 0 | 0 | 0 | 0.0015 | 0 | 0 | 0 | 0 | 0 | 0 |
| BC_tissue | Hoskinson_2022 | 0.6074 | 0.0011 | 0 | 11.5905 | 0 | 2.7396 | 0.0033 | 0 | 0 | 0 | 0 | 0 | 0 | 0.0011 | 0.0022 | 0 | 0 | 0 | 0 | 0.0022 |
| BC_adjacent | Hoskinson_2022 | 1.1226 | 0 | 0 | 0.007 | 0 | 0 | 0.001 | 0.001 | 0 | 0 | 0 | 0 | 0 | 0.001 | 0 | 2.4143 | 0 | 0 | 0 | 0 |
| BC_adjacent | Hoskinson_2022 | 1.1775 | 0 | 0 | 1.9041 | 0 | 0 | 0 | 0 | 0 | 0.0019 | 0 | 0 | 0 | 2.2494 | 0 | 0 | 0 | 0 | 0 | 0 |
| BC_tissue | Hoskinson_2022 | 0.3146 | 0.048 | 0 | 1.3487 | 0 | 0 | 0 | 0 | 0 | 0 | 0 | 0 | 0 | 0.6427 | 0 | 0 | 1.0763 | 0 | 0 | 0.0019 |
| BC_adjacent | Hoskinson_2022 | 2.6222 | 0 | 0 | 0.6384 | 0.0017 | 0.0017 | 0.0086 | 0 | 0 | 0 | 0 | 0 | 0 | 0 | 0 | 0 | 0 | 0 | 0 | 0.0034 |
| BC_tissue | Hoskinson_2022 | 0.4978 | 0 | 0.0025 | 15.0098 | 0.0075 | 0 | 0.005 | 0 | 0 | 0.0025 | 0 | 0 | 0 | 0 | 0 | 0 | 0 | 0 | 0 | 0.0101 |
| BC_tissue | Hoskinson_2022 | 3.1187 | 0 | 0 | 25.279 | 0 | 0 | 0.0015 | 0 | 0 | 0 | 0 | 0 | 0 | 0 | 0 | 0 | 0 | 0 | 0 | 0.0015 |
| BC_tissue | Hoskinson_2022 | 4.0837 | 0 | 0 | 0.9293 | 0 | 0.0034 | 0 | 0 | 0 | 0 | 0 | 0 | 0 | 0 | 0 | 0 | 0 | 0 | 0 | 0 |
| BC_adjacent | Hoskinson_2022 | 4.5148 | 0 | 0 | 7.6128 | 0.0015 | 0 | 0.0015 | 0 | 0 | 0 | 0 | 0 | 0 | 0.0015 | 0 | 1.148 | 0 | 0 | 0 | 2.6686 |
| BC_adjacent | Hoskinson_2022 | 0.0413 | 0 | 0 | 0.7032 | 0 | 0 | 0 | 0 | 0 | 0 | 0 | 0 | 0 | 0.0857 | 0.0032 | 0 | 0 | 0.0016 | 0 | 0.3571 |
| BC_tissue | Hoskinson_2022 | 0.0104 | 0 | 0 | 0 | 0 | 0 | 0 | 0 | 0 | 0 | 0 | 0 | 0 | 0.0017 | 0.0017 | 0 | 0 | 0 | 0 | 0 |
| BC_tissue | Hoskinson_2022 | 0.0276 | 0 | 0 | 1.2825 | 0 | 0 | 0 | 0 | 0 | 0 | 0 | 0 | 0 | 0 | 12.5322 | 0 | 0 | 0 | 0 | 0 |
| BC_tissue | Hoskinson_2022 | 1.134 | 0 | 0 | 15.7953 | 0.0016 | 0 | 0 | 0 | 0 | 0 | 0 | 0 | 0 | 14.5457 | 0 | 0 | 0 | 0 | 0 | 0.0016 |
| BC_tissue | Hoskinson_2022 | 1.3141 | 0.002 | 0 | 0.0334 | 0 | 0.0059 | 0 | 0.0059 | 0 | 0.0039 | 0 | 0 | 0 | 0.0059 | 0 | 0.002 | 0 | 0 | 0 | 0 |
| BC_adjacent | Hoskinson_2022 | 0.0512 | 0 | 0 | 0.425 | 5.6083 | 0 | 0 | 0.3263 | 0 | 0 | 0 | 0 | 0.0038 | 0 | 0 | 0 | 0 | 0 | 0 | 0 |
| BC_tissue | Hoskinson_2022 | 0.0836 | 0.0139 | 0 | 5.3383 | 0 | 0 | 0 | 0 | 0 | 0.1852 | 0 | 0 | 0 | 0 | 0.002 | 0 | 0 | 0 | 0 | 0.0279 |
| BC_adjacent | Hoskinson_2022 | 0.0113 | 0.0028 | 0 | 3.2305 | 0 | 0 | 0 | 0 | 0 | 0.0776 | 0 | 0 | 0 | 0.007 | 0 | 0.0014 | 0 | 0 | 0 | 0 |
| BC_tissue | Hoskinson_2022 | 0.0078 | 0.0033 | 0 | 2.1896 | 0 | 0 | 0 | 0 | 0 | 0.0089 | 0 | 0 | 2.5912 | 0 | 0 | 0 | 0 | 0 | 0 | 0.0045 |
| BC_tissue | Hoskinson_2022 | 0.0675 | 0.0051 | 0 | 0.0013 | 0 | 0 | 0 | 0 | 0 | 0.037 | 0 | 0 | 0 | 0 | 0.0013 | 0 | 0 | 0 | 0 | 0.0038 |
| BC_adjacent | Hoskinson_2022 | 0.1595 | 0.0082 | 0 | 5.8859 | 0 | 0 | 0.0016 | 0 | 0 | 0.0329 | 0 | 0 | 0 | 0 | 0 | 0 | 4.9699 | 0 | 0 | 0.0033 |
| BC_adjacent | Hoskinson_2022 | 0.2071 | 0.0018 | 0 | 8.4571 | 0 | 0.0037 | 0.0018 | 0.0018 | 0 | 0.0351 | 0 | 0 | 0 | 0 | 0.0018 | 0 | 0 | 0 | 0 | 0.0074 |
| BC_tissue | Hoskinson_2022 | 0 | 0.0255 | 0 | 0.0102 | 0 | 0 | 0 | 0 | 0 | 0 | 0 | 0 | 0 | 0.0013 | 1.3195 | 0 | 0 | 0 | 0 | 0 |
| BC_tissue | Hoskinson_2022 | 0 | 0.017 | 0 | 0 | 0 | 0 | 0 | 0 | 0 | 0 | 0 | 0 | 0 | 0.0183 | 1.3381 | 0 | 0 | 0 | 0 | 0 |
| BC_adjacent | Hoskinson_2022 | 0.0141 | 1.3326 | 0 | 4.76 | 0.477 | 0 | 0.0013 | 0.0013 | 0 | 0.0294 | 0 | 0 | 0.0013 | 0.0013 | 0 | 0 | 0 | 0 | 0 | 0 |
| BC_tissue | Hoskinson_2022 | 0.0772 | 0.018 | 0 | 6.5655 | 0 | 0.0026 | 0 | 0.0013 | 4.4182 | 0.0617 | 0 | 0 | 0 | 0 | 0 | 0 | 0 | 0 | 0 | 0.0116 |
| BC_adjacent | Hoskinson_2022 | 0.0108 | 0.0011 | 0 | 0.4779 | 0 | 0 | 0 | 3.2056 | 0 | 0.0054 | 0 | 0 | 0.6871 | 0.1355 | 0 | 0 | 0.6762 | 0 | 0 | 0.0011 |
| BC_tissue | Hoskinson_2022 | 0.0685 | 0.0011 | 0 | 0.0022 | 0.0011 | 0.0784 | 0.0011 | 0 | 0.806 | 0.0276 | 0 | 0 | 0 | 0 | 0 | 0 | 0 | 0 | 0 | 0.0022 |
| BC_tissue | Hoskinson_2022 | 0.1121 | 0.0046 | 0 | 12.6656 | 0 | 0.2831 | 0 | 0 | 0.0023 | 0.0289 | 0 | 0 | 0 | 0 | 0 | 0 | 0 | 0 | 0 | 12.1943 |
| BC_tissue | Hoskinson_2022 | 0.1576 | 0.0653 | 0 | 12.5637 | 0 | 0 | 0 | 0.0207 | 0 | 0.1449 | 0 | 0 | 0 | 0 | 0.0096 | 0 | 0.0016 | 0 | 0 | 0.0032 |
| BC_tissue | Hoskinson_2022 | 0.03 | 0 | 0 | 1.7021 | 0 | 0 | 0 | 0 | 0 | 0.012 | 0 | 0 | 0 | 0 | 0 | 0 | 0 | 0 | 0 | 0.0075 |
| BC_adjacent | Hoskinson_2022 | 0.2912 | 0.0919 | 0 | 5.979 | 0.0156 | 0 | 0.0016 | 0.0031 | 0 | 0.2336 | 0.0078 | 0 | 0 | 0.0016 | 0.0436 | 0 | 0.0016 | 0 | 0 | 0.0779 |
| BC_tissue | Hoskinson_2022 | 0.1623 | 0.005 | 0.0025 | 24 | 0 | 0 | 0.0012 | 0 | 0 | 0.0624 | 0 | 0.0087 | 0 | 0.0012 | 0 | 0 | 0 | 0 | 0 | 0.0087 |
| BC_tissue | Hoskinson_2022 | 0.0749 | 0.0029 | 0 | 12.6406 | 0 | 0 | 0 | 0 | 0 | 0.0214 | 0 | 0.0019 | 0 | 0.0019 | 0 | 0 | 0 | 0 | 0 | 0 |
| BC_tissue | Hoskinson_2022 | 0.2343 | 0.0205 | 0 | 19.5969 | 7.0381 | 0 | 0 | 0.0013 | 0 | 0.1703 | 0 | 0.0026 | 0 | 14.3631 | 0 | 0 | 0 | 0 | 0 | 0 |
| BC_adjacent | Hoskinson_2022 | 0 | 0 | 0 | 0.0288 | 0.0008 | 0 | 0 | 0 | 0 | 0.0008 | 0 | 46.7898 | 0 | 0.0015 | 0 | 0 | 0 | 0 | 0 | 0 |
| BC_tissue | Hoskinson_2022 | 0.0161 | 0 | 0 | 0.7572 | 0 | 0 | 0 | 0 | 0 | 0.0019 | 0 | 0.0038 | 0 | 0.0009 | 0 | 0 | 0 | 0 | 0 | 0.0009 |
| BC_adjacent | Hoskinson_2022 | 0.2074 | 0.0062 | 0 | 0.0114 | 0 | 0 | 0.001 | 0 | 0 | 0.0549 | 0 | 0.001 | 0 | 0 | 0 | 0 | 0 | 0 | 0 | 0.0041 |
| BC_tissue | Hoskinson_2022 | 0.1587 | 0.0517 | 0 | 39.7347 | 0.0018 | 0.0178 | 0 | 0 | 0 | 0.2228 | 0 | 0.0018 | 0 | 0.0018 | 0 | 0 | 0 | 0 | 0 | 0 |
| BC_adjacent | Hoskinson_2022 | 0.0038 | 0 | 0 | 0.0881 | 14.4625 | 0 | 0 | 0 | 0 | 0.0138 | 0 | 0.0013 | 0 | 0 | 0.0025 | 0.2064 | 0 | 0 | 0 | 0.0025 |
| BC_adjacent | Hoskinson_2022 | 0.8382 | 0 | 0 | 0.0216 | 0.0054 | 0 | 0.0027 | 0.0027 | 0 | 0.0433 | 0 | 0 | 0 | 8.6799 | 0.0108 | 0 | 0 | 0 | 0 | 0 |
| BC_adjacent | Hoskinson_2022 | 0.0721 | 0 | 0 | 0.8286 | 0.0057 | 0 | 0 | 0.0019 | 0 | 0.2351 | 0 | 0 | 0 | 0 | 0 | 0 | 0 | 0 | 0 | 0 |
| BC_adjacent | Hoskinson_2022 | 0.1887 | 0 | 0 | 0 | 0 | 0.0016 | 0 | 0 | 0 | 0.0607 | 0 | 0 | 0 | 0.0016 | 0 | 10.2937 | 0 | 0 | 0 | 0.0115 |
| BC_adjacent | Hoskinson_2022 | 0.0229 | 3.2818 | 0 | 0.7531 | 0 | 0 | 0 | 0 | 0 | 0.0094 | 0 | 0 | 0 | 0.3435 | 0 | 0 | 0 | 0 | 0 | 0.0013 |
| BC_tissue | Hoskinson_2022 | 0.2317 | 0.0116 | 0 | 0.007 | 0.0023 | 0 | 0 | 0 | 0 | 0.2201 | 0 | 34.6177 | 0 | 0 | 0 | 1.6914 | 0 | 0 | 0 | 0 |
| BC_adjacent | Hoskinson_2022 | 0.0676 | 0 | 0 | 1.613 | 0 | 0 | 0 | 0 | 0 | 0.0792 | 0 | 0.0033 | 0 | 0 | 0 | 0.0016 | 0 | 0 | 0 | 1.3491 |
| BC_tissue | Hoskinson_2022 | 0.0036 | 0.0036 | 0.0054 | 15.9687 | 0.0126 | 0 | 0.0036 | 0 | 0 | 0.1635 | 0 | 11.5776 | 0 | 0 | 0.0054 | 0.0018 | 0 | 0 | 0 | 0.0054 |
| BC_tissue | Hoskinson_2022 | 0.0054 | 0 | 0 | 3.6162 | 0.0361 | 0.0036 | 0 | 0.0036 | 0 | 0.0253 | 0 | 0 | 0 | 0 | 0 | 0 | 0 | 0 | 0 | 0.0018 |
| BC_tissue | Hoskinson_2022 | 0.2788 | 0.0047 | 0 | 0.0024 | 0.0118 | 0 | 0.0071 | 0 | 0 | 0.0425 | 0.0024 | 0 | 0 | 0 | 0 | 0 | 11.4818 | 0 | 0 | 0.0118 |
| BC_tissue | Hoskinson_2022 | 0.1035 | 0.0107 | 0 | 0.0161 | 0.0054 | 0 | 0.0214 | 1.2192 | 0 | 0.0232 | 0 | 0 | 0 | 0 | 0 | 2.7221 | 0 | 0 | 0 | 0 |
| BC_adjacent | Hoskinson_2022 | 0.0349 | 0.0016 | 0 | 3.7779 | 47.8731 | 0 | 0 | 0 | 0 | 0.0063 | 0 | 0 | 0 | 0 | 0 | 0 | 0 | 0 | 0 | 0.0016 |
| BC_adjacent | Hoskinson_2022 | 0.0057 | 0 | 0 | 6.7409 | 47.0867 | 0 | 0 | 7.1217 | 0 | 0.0377 | 0 | 0.0038 | 0 | 0 | 0 | 0 | 0 | 0 | 0 | 0 |
| BC_adjacent | Hoskinson_2022 | 0.0028 | 0 | 0 | 0.3563 | 69.6499 | 0 | 0 | 0 | 0 | 0.0014 | 0 | 0 | 0 | 0 | 0 | 0.0557 | 0 | 0 | 0 | 0 |
| BC_tissue | Hoskinson_2022 | 0.0249 | 0 | 0 | 29.5511 | 0.0083 | 0 | 0 | 0 | 0 | 0.0748 | 0 | 0 | 0 | 0 | 0 | 0 | 0 | 0 | 0 | 0.0111 |
| BC_tissue | Hoskinson_2022 | 0.2866 | 5.8527 | 0 | 0.0022 | 0 | 0 | 0.0065 | 0 | 0 | 0.0129 | 0 | 0 | 0.0022 | 0 | 0 | 0 | 0 | 0 | 0 | 0 |
| BC_adjacent | Hoskinson_2022 | 0.2389 | 0.0062 | 0 | 25.1511 | 8.0056 | 0 | 0 | 0 | 0 | 0.0519 | 0 | 0 | 0 | 0.0062 | 0 | 0 | 0 | 0 | 0 | 0 |
| BC_adjacent | Hoskinson_2022 | 0.1622 | 0.0053 | 0 | 0 | 0.0053 | 0 | 0.0213 | 0.0027 | 0.008 | 0.016 | 0 | 0 | 0 | 4.6721 | 0 | 0 | 0 | 0 | 0 | 0 |
| BC_tissue | Hoskinson_2022 | 0.0597 | 0.0043 | 0.0021 | 0 | 0 | 0 | 0 | 0 | 0 | 0 | 0 | 0 | 0 | 0 | 0 | 0 | 0 | 0 | 0 | 0.0021 |
| BC_adjacent | Hoskinson_2022 | 0.0024 | 0.0024 | 0.0024 | 17.0719 | 7.332 | 0 | 0 | 0 | 0 | 0.1062 | 0 | 0 | 0 | 1.7209 | 0 | 0 | 0 | 0 | 0 | 0 |
| BC_tissue | Hoskinson_2022 | 0.0071 | 0 | 0 | 0.6186 | 63.7318 | 0 | 0 | 0 | 0 | 0.0107 | 0 | 0 | 0 | 0 | 0 | 0 | 0.0018 | 0 | 0 | 0 |
| BC_adjacent | Hoskinson_2022 | 0.0185 | 0.0018 | 0 | 5.6056 | 0.4622 | 0 | 0 | 0 | 0 | 0.024 | 0 | 0 | 0.8634 | 0 | 0 | 0 | 0 | 0 | 0 | 0 |
| BC_adjacent | Hoskinson_2022 | 0.223 | 0.0058 | 0 | 0.0116 | 0 | 0 | 0.029 | 0 | 0 | 0.0319 | 0 | 0 | 0 | 1.4191 | 0 | 0 | 0 | 0 | 0 | 0 |
| BC_tissue | Hoskinson_2022 | 0.1233 | 0.0044 | 0 | 2.4616 | 0.0022 | 0 | 0.022 | 0 | 0 | 0.0727 | 0 | 0 | 0 | 0 | 0 | 0 | 0 | 0 | 0 | 0 |
| BC_tissue | Hoskinson_2022 | 0 | 0.0047 | 0 | 0.5154 | 0.189 | 0 | 0 | 0 | 0 | 0.0031 | 0 | 0.0016 | 0 | 0 | 0 | 0 | 0 | 0 | 0 | 0 |
| BC_adjacent | Hoskinson_2022 | 0.0014 | 0.0058 | 0 | 1.1991 | 55.2127 | 0 | 0 | 0 | 0 | 0.0087 | 0 | 0 | 0 | 0.0014 | 0 | 0 | 0 | 0 | 0 | 0 |
| BC_adjacent | Hoskinson_2022 | 0.1774 | 0.0019 | 0 | 14.6228 | 0 | 0 | 0.0019 | 0.0038 | 0 | 0.1507 | 0 | 0 | 0 | 0 | 0.0038 | 0.0019 | 0 | 0 | 0 | 0 |
| BC_adjacent | Hoskinson_2022 | 0.1818 | 0 | 0 | 33.5567 | 0 | 0 | 0 | 0 | 0 | 0.0701 | 0 | 0 | 0 | 0 | 0 | 0 | 0.0022 | 0 | 0 | 0 |
| BC_adjacent | Hoskinson_2022 | 0.0534 | 0.0037 | 0 | 6.4876 | 0.0018 | 0 | 0 | 0 | 0 | 0.081 | 0 | 0 | 0 | 0 | 0 | 0 | 0 | 0 | 0 | 0 |
| BC_adjacent | Hoskinson_2022 | 0.568 | 0.037 | 0 | 0 | 0.1062 | 0 | 0.0049 | 0 | 0.0099 | 0.0272 | 0 | 0 | 0 | 0 | 0 | 0 | 0 | 0 | 0 | 0 |
| BC_adjacent | Hoskinson_2022 | 1.1766 | 2.8427 | 0 | 0 | 0.0032 | 0 | 0 | 1.3193 | 0 | 0.0065 | 0 | 0 | 2.9238 | 0.0373 | 0 | 0 | 0 | 0 | 0 | 0 |
| BC_tissue | Hoskinson_2022 | 1.2746 | 0 | 0 | 0 | 0 | 0 | 0 | 0 | 0 | 0.227 | 0 | 0 | 0 | 0 | 0 | 0 | 0.519 | 0 | 0 | 0 |
| BC_tissue | Hoskinson_2022 | 2.0528 | 0.0015 | 0 | 0 | 0 | 0 | 0.0075 | 0 | 0 | 0.0134 | 0 | 0 | 0 | 1.3045 | 0 | 0 | 0 | 0 | 0 | 0 |
| BC_adjacent | Hoskinson_2022 | 0 | 0 | 0 | 7.4622 | 0 | 0 | 0 | 0 | 0.1936 | 0.063 | 0 | 0 | 1.9472 | 0 | 0 | 0 | 0 | 0 | 0 | 0.014 |
| BC_adjacent | Hoskinson_2022 | 0.0204 | 0 | 0 | 0.0167 | 0.0056 | 0 | 0 | 0 | 0 | 0.0389 | 0 | 0.0703 | 0 | 0 | 0 | 0 | 0.2165 | 0 | 0 | 0 |
| BC_tissue | Hoskinson_2022 | 0.0113 | 9.2969 | 0 | 0.8095 | 0.3057 | 0 | 0 | 0 | 0 | 0.0151 | 0 | 0.0019 | 0 | 0.7359 | 0 | 0 | 0 | 0 | 0 | 0 |
| BC_adjacent | Hoskinson_2022 | 0.0611 | 13.0352 | 0 | 14.1209 | 0.0054 | 0 | 0 | 2.1983 | 0 | 0.0845 | 0 | 0 | 0.0018 | 0 | 0.0072 | 0 | 0 | 0 | 0 | 0 |
| BC_tissue | Hoskinson_2022 | 3.8565 | 39.0873 | 0 | 0.0154 | 0 | 0 | 0 | 0 | 0 | 0 | 0 | 0 | 0 | 1.137 | 0 | 0 | 0 | 0 | 0 | 0 |
| BC_adjacent | Esposito_2022 | 0.9873 | 70.2324 | 0 | 0.0125 | 0.0375 | 0 | 0 | 0 | 0 | 0 | 0 | 0 | 0 | 0 | 0 | 0 | 0 | 0 | 0 | 0 |
| BC_tissue | Esposito_2022 | 1.473 | 28.1616 | 0 | 0.007 | 0 | 0 | 0 | 0 | 0.007 | 0 | 0 | 0 | 0 | 0 | 0 | 0 | 0 | 0 | 0 | 0 |
| BC_tissue | Esposito_2022 | 10.9037 | 11.0233 | 0 | 0 | 0 | 0 | 0 | 0 | 0 | 0.1839 | 0 | 0 | 0 | 0 | 0 | 0.1103 | 0 | 0 | 0 | 0 |
| BC_adjacent | Esposito_2022 | 26.1543 | 9.3427 | 0.8691 | 0.0136 | 8.6773 | 0 | 0 | 0 | 0 | 1.2629 | 0 | 0 | 0.7333 | 0 | 0 | 0 | 0 | 0 | 0 | 0 |
| BC_tissue | Esposito_2022 | 4.5813 | 8.6317 | 0 | 0 | 0.8495 | 0 | 0 | 0 | 0 | 0 | 0 | 0 | 0 | 1.9417 | 10.2549 | 0 | 0 | 0 | 0 | 0 |
| BC_adjacent | Esposito_2022 | 23.6458 | 21.0417 | 0 | 0 | 4.0625 | 0 | 0 | 0 | 0.2083 | 0 | 0 | 0 | 0 | 0.2083 | 0 | 0 | 0 | 0 | 0 | 0 |
| BC_tissue | Esposito_2022 | 0.2703 | 25.9073 | 0 | 0.0129 | 5.0965 | 4.1956 | 0 | 0 | 0 | 1.6731 | 0 | 0 | 0 | 0 | 0 | 0 | 0 | 0 | 0.0129 | 0 |
| BC_adjacent | Esposito_2022 | 34.5429 | 8.5379 | 0.3735 | 0 | 5.6563 | 0 | 0 | 0 | 1.5653 | 0.2134 | 0 | 0 | 0 | 0 | 0 | 0 | 0 | 1.6364 | 0 | 0 |
| BC_adjacent | Esposito_2022 | 12.1007 | 10.1162 | 2.4685 | 0 | 2.3717 | 0 | 0 | 0 | 0.484 | 1.0165 | 0 | 0 | 1.4037 | 0 | 0 | 0 | 0 | 1.2101 | 0 | 0 |
| BC_tissue | Esposito_2022 | 3.9569 | 29.0055 | 0 | 0 | 3.5506 | 0 | 0 | 0 | 0.0442 | 0 | 0 | 0 | 0 | 0.5211 | 0 | 0 | 0 | 1.5545 | 0.0442 | 0 |
| BC_adjacent | Esposito_2022 | 3.0973 | 19.9272 | 0 | 0.007 | 10.683 | 0 | 0 | 0 | 2.3384 | 0 | 0.2406 | 0 | 7.3289 | 0.0035 | 0 | 0 | 0 | 0.7043 | 0 | 0 |
| BC_tissue | Esposito_2022 | 22.7523 | 17.5098 | 0 | 0 | 0 | 0 | 0 | 0 | 0 | 2.4902 | 0 | 0 | 0 | 0 | 0 | 0 | 0 | 0 | 0 | 0 |
| BC_adjacent | Esposito_2022 | 3.0561 | 11.5312 | 0 | 0 | 0 | 0 | 0 | 0 | 0 | 0 | 0 | 0 | 0 | 0.189 | 0 | 0 | 0 | 0 | 0 | 0 |
| BC_tissue | Esposito_2022 | 0.1323 | 6.862 | 0 | 0.0378 | 0.0189 | 0.0945 | 0 | 0 | 0 | 0.1323 | 0 | 0 | 0 | 0 | 0 | 0 | 0 | 0 | 0 | 0 |
| BC_adjacent | Esposito_2022 | 22.716 | 13.2211 | 0 | 0 | 2.3345 | 0 | 0 | 0 | 1.7508 | 0.1796 | 0 | 0 | 0.7183 | 0.6958 | 0 | 0 | 0 | 0.2245 | 0 | 0 |
| BC_tissue | Esposito_2022 | 4.3525 | 0.3373 | 0 | 0 | 0 | 0 | 0 | 0 | 0 | 0.2036 | 0 | 0 | 0 | 0 | 0 | 0 | 0 | 0 | 0 | 0 |
| BC_tissue | Esposito_2022 | 14.0533 | 11.5056 | 0 | 0 | 0.5095 | 0 | 0 | 0 | 2.8928 | 0 | 0 | 0 | 7.6759 | 0 | 0.2794 | 0 | 0 | 0 | 0 | 0 |
| BC_adjacent | Esposito_2022 | 15.2513 | 4.5927 | 2.383 | 0 | 1.9497 | 0 | 0 | 0 | 3.2496 | 0.3899 | 0 | 0 | 0 | 0.26 | 0 | 0 | 0 | 1.6464 | 0.0433 | 0 |
| BC_tissue | Esposito_2022 | 3.7558 | 16.9324 | 0 | 0 | 6.0638 | 0 | 0 | 0 | 4.0285 | 0 | 1.0281 | 0 | 0 | 0.0839 | 0 | 0 | 0 | 0 | 0.021 | 0 |
| BC_adjacent | Esposito_2022 | 25.4764 | 6.7202 | 0.4514 | 0.0502 | 10.9328 | 0 | 0 | 0 | 0 | 0.2006 | 0 | 0 | 0.0502 | 0.0502 | 0.4012 | 0 | 0 | 0.652 | 0 | 0 |
| BC_tissue | Esposito_2022 | 1.5328 | 20.8708 | 0 | 0 | 0 | 0.0111 | 0 | 0 | 0 | 0.0111 | 5.9425 | 0 | 0 | 0 | 0 | 0 | 0 | 0 | 0 | 0 |
| BC_adjacent | Esposito_2022 | 29.8082 | 8.9863 | 2.6301 | 0 | 4 | 0 | 0 | 0 | 0.4932 | 0.0548 | 0 | 0 | 1.4247 | 0.6027 | 0 | 0 | 0 | 1.863 | 0 | 0 |
| BC_tissue | Esposito_2022 | 2.7168 | 25.238 | 0 | 0.019 | 2.6469 | 0 | 0 | 0 | 0 | 0 | 0 | 0 | 0 | 0 | 0 | 0 | 0 | 0 | 0 | 0 |
| BC_adjacent | Esposito_2022 | 27.1185 | 10.1423 | 3.8222 | 0 | 2.102 | 0 | 0 | 0.0047 | 0 | 0.1037 | 0 | 0 | 1.3243 | 0 | 0 | 0 | 0 | 0.1838 | 0 | 0 |
| BC_tissue | Esposito_2022 | 4.7498 | 38.2425 | 0 | 0.0049 | 0.1073 | 0 | 0 | 0 | 0.8632 | 0 | 0 | 0 | 0 | 0 | 0 | 0 | 0 | 0 | 0 | 0 |
| BC_adjacent | Esposito_2022 | 78.2609 | 1.8807 | 0 | 0 | 1.3953 | 0 | 0 | 0 | 0 | 0 | 0 | 0 | 0 | 0 | 0 | 0 | 0 | 0 | 0 | 0 |
| BC_adjacent | Esposito_2022 | 8.2096 | 29.9212 | 0 | 0 | 0.6811 | 0 | 0 | 0 | 0.0123 | 0.2503 | 1.1898 | 0 | 0.1108 | 0.0164 | 0.4349 | 0 | 0 | 0 | 0 | 0 |
| BC_tissue | Esposito_2022 | 2.9489 | 9.463 | 0 | 0.0147 | 9.2136 | 0 | 0 | 0 | 0 | 0 | 0 | 0 | 8.1719 | 0 | 0 | 0 | 0 | 0 | 0 | 0 |
| BC_adjacent | Esposito_2022 | 21.3752 | 4.9576 | 1.7317 | 0 | 3.3447 | 0 | 0 | 0 | 0.0509 | 0.3735 | 0 | 0 | 0 | 0.0849 | 0.3905 | 0 | 0 | 0.0509 | 0 | 0 |
| BC_tissue | Esposito_2022 | 5.1372 | 7.1958 | 0 | 0 | 0 | 0 | 0 | 0 | 0 | 0 | 6.2685 | 0 | 0 | 0.0835 | 0 | 0 | 0 | 4.0894 | 0 | 0 |
| BC_adjacent | Esposito_2022 | 18.0007 | 8.2789 | 6.2178 | 0 | 5.4964 | 0 | 0 | 0.1031 | 0.2748 | 0.9275 | 0 | 0 | 0 | 0.0344 | 0 | 0 | 0 | 1.752 | 0 | 0 |
| BC_tissue | Esposito_2022 | 10.0913 | 9.6011 | 2.7214 | 0 | 0.0169 | 0 | 0 | 0 | 5.3076 | 0 | 0 | 0 | 2.1805 | 0 | 0 | 0 | 0 | 0 | 0 | 0 |
| BC_adjacent | Esposito_2022 | 29.2122 | 5.6541 | 0 | 0.0045 | 0.4958 | 0 | 0 | 0.0136 | 0.2365 | 0 | 0.7005 | 0 | 0.3139 | 0.1137 | 0.1547 | 0.0045 | 0 | 0.6687 | 0 | 0 |
| BC_tissue | Esposito_2022 | 5.3085 | 2.9391 | 0 | 0.0038 | 0 | 0 | 0 | 0 | 0.1443 | 0 | 0 | 0 | 0 | 1.162 | 0 | 0 | 0 | 0 | 0 | 0 |
| BC_adjacent | Esposito_2022 | 12.6056 | 15.4091 | 0 | 0 | 1.2228 | 0 | 0 | 0 | 0.169 | 0 | 0 | 0 | 0 | 0 | 0 | 0 | 0 | 9.7226 | 0.1094 | 0 |
| BC_tissue | Esposito_2022 | 3.2134 | 0.0189 | 0 | 0 | 4.9621 | 7.8093 | 0 | 0 | 0 | 0 | 0 | 0 | 0.0063 | 0 | 0 | 0 | 0 | 0 | 0 | 0 |
| BC_tissue | Esposito_2022 | 2.3641 | 24.221 | 0 | 0.0362 | 1.3859 | 0 | 0 | 0.0181 | 0 | 0.7156 | 0 | 0 | 0 | 0 | 0 | 0 | 0 | 0 | 0 | 0 |
| BC_adjacent | Esposito_2022 | 13.9512 | 18.2602 | 5.6748 | 0 | 0.9919 | 0 | 0 | 0 | 0.0325 | 0 | 0 | 0 | 0 | 2.0813 | 0 | 0 | 0 | 1.5772 | 0 | 0 |
| BC_adjacent | Esposito_2022 | 37.5925 | 7.7783 | 1.6916 | 0 | 2.8092 | 0 | 0 | 0 | 0.0151 | 0.9515 | 0 | 0 | 1.1781 | 1.1932 | 0 | 0 | 0 | 0.3474 | 0 | 0 |
| BC_tissue | Esposito_2022 | 13.6104 | 0.0585 | 0 | 0 | 0 | 0 | 0 | 0 | 14.4739 | 0 | 0 | 0 | 0 | 0 | 0 | 0 | 0 | 0 | 0 | 0 |
| BC_adjacent | Esposito_2022 | 33.6852 | 4.2706 | 0.3359 | 0 | 6.9578 | 0 | 0 | 0 | 0.8157 | 0.048 | 0.3839 | 0 | 0.096 | 0.048 | 0.096 | 0 | 0 | 0.8637 | 0 | 0 |
| BC_tissue | Esposito_2022 | 15.7227 | 2.7971 | 0 | 0.0174 | 0 | 0 | 0 | 0 | 0 | 0 | 0 | 0 | 0 | 0 | 0 | 0 | 0 | 0 | 0 | 0 |
| BC_adjacent | Esposito_2022 | 41.9192 | 2.6936 | 0.8418 | 0 | 4.0404 | 0 | 0 | 0 | 0 | 0.3367 | 0 | 0 | 0 | 0 | 0 | 0 | 0 | 0.1684 | 0 | 0 |
| BC_tissue | Esposito_2022 | 1.8501 | 37.9767 | 0 | 0 | 0.0043 | 0 | 0 | 0 | 0 | 0 | 0 | 0 | 0 | 0 | 0 | 0 | 0 | 0 | 0 | 0 |
| BC_adjacent | Esposito_2022 | 1.1832 | 3.7723 | 0 | 0.0117 | 1.8393 | 0 | 0 | 0 | 0 | 0.3046 | 0 | 0 | 0 | 0.0234 | 0 | 0 | 0 | 0 | 0 | 0 |
| BC_tissue | Esposito_2022 | 12.8419 | 21.3005 | 0 | 0.003 | 0.0015 | 0 | 0 | 0 | 1.0749 | 0 | 0 | 0 | 0 | 0 | 0 | 0 | 0 | 0 | 0 | 0 |
| BC_adjacent | Esposito_2022 | 3.2217 | 6.5694 | 0 | 0 | 0.6821 | 0 | 0 | 0 | 0.0315 | 0 | 0 | 0 | 14.1778 | 0 | 0 | 0 | 0 | 0 | 0.0472 | 0 |
| BC_tissue | Esposito_2022 | 8.258 | 33.1258 | 0 | 0 | 7.0598 | 0 | 0 | 0 | 0 | 0 | 0 | 0 | 0 | 0 | 0 | 0 | 0 | 0 | 0 | 0 |
| BC_adjacent | Esposito_2022 | 5.392 | 16.1216 | 0 | 0.006 | 0 | 0 | 0 | 0 | 1.0881 | 1.3238 | 0 | 0 | 0 | 0.1813 | 0 | 0 | 0 | 0.8161 | 0 | 0 |
| BC_tissue | Esposito_2022 | 4.5332 | 55.5347 | 0.3046 | 0.0586 | 0.5388 | 0 | 0 | 0 | 0.0351 | 0 | 0 | 0 | 0 | 0.0351 | 0.0117 | 0 | 0 | 0 | 0 | 0 |
| BC_tissue | Esposito_2022 | 4.7662 | 19.7834 | 0 | 0 | 1.7304 | 0 | 0 | 0.7286 | 1.3256 | 7.0836 | 0 | 0 | 0 | 0 | 0 | 0 | 0 | 0 | 0 | 0 |
| BC_adjacent | Esposito_2022 | 30.2605 | 8.3166 | 0 | 0 | 3.7742 | 0 | 0 | 0.0334 | 0 | 0.6346 | 0 | 0 | 3.2064 | 0 | 0.0668 | 0 | 0 | 0.7682 | 0 | 0 |
| BC_tissue | Esposito_2022 | 1.9068 | 20.5394 | 0 | 0 | 0.0545 | 0 | 0 | 0 | 0 | 0 | 0 | 0 | 0 | 0 | 0 | 0 | 0 | 0 | 0 | 0 |
| BC_adjacent | Esposito_2022 | 26.5387 | 5.0298 | 0 | 0 | 2.4487 | 0 | 0 | 0 | 0 | 0.0662 | 0 | 0 | 0 | 0 | 0 | 0 | 0 | 1.3898 | 0 | 0 |
| BC_tissue | Esposito_2022 | 4.0889 | 29.7802 | 0 | 0 | 10.3837 | 0 | 0 | 0 | 0.3939 | 0 | 0 | 0 | 0 | 0.0079 | 0.2757 | 0 | 0 | 0 | 0 | 0 |
| BC_adjacent | Esposito_2022 | 18.5676 | 7.2502 | 1.1052 | 0 | 3.9788 | 0 | 0 | 0.0442 | 2.7851 | 0.1768 | 0 | 0 | 0 | 0 | 0 | 0 | 0 | 0.5305 | 0 | 0 |
| BC_tissue | Esposito_2022 | 0.9899 | 0 | 0 | 0 | 0 | 0 | 0 | 0 | 0 | 4.9367 | 0 | 0 | 0 | 0 | 0 | 0 | 0 | 0 | 0 | 0 |
| BC_adjacent | Esposito_2022 | 15.1443 | 10.2075 | 1.6933 | 0.0238 | 5.223 | 0 | 0 | 0 | 0 | 2.0033 | 0.0238 | 0 | 1.5025 | 0.6916 | 0 | 0 | 0 | 0 | 0 | 0 |
| BC_tissue | Esposito_2022 | 5.5232 | 51.2965 | 0 | 0.0066 | 4.0878 | 0 | 0 | 2.9898 | 0.0265 | 0.2183 | 1.7463 | 0 | 0 | 0 | 0 | 0 | 0 | 0 | 0 | 0 |
| BC_adjacent | Esposito_2022 | 29.2578 | 5.5575 | 0.6454 | 0 | 5.2349 | 0 | 0 | 0 | 0.0717 | 0.1076 | 0.0359 | 0 | 1.2908 | 0.6812 | 0 | 0 | 0 | 1.6493 | 0 | 0 |
| BC_adjacent | Esposito_2022 | 15.569 | 8.6486 | 2.2419 | 0.0156 | 3.2306 | 0 | 0 | 0 | 0.0234 | 0.5527 | 0 | 0 | 3.4953 | 0.0311 | 0.1479 | 0 | 0 | 0.6383 | 0 | 0 |
| BC_tissue | Esposito_2022 | 6.425 | 24.6601 | 0.3732 | 0 | 2.4793 | 0 | 0 | 0 | 0 | 0 | 0 | 0 | 0 | 0 | 0 | 0 | 0 | 0 | 0.0089 | 0 |
| BC_adjacent | Esposito_2022 | 60.2552 | 7.7527 | 0 | 0 | 5.2748 | 0 | 0 | 0 | 0.9814 | 0 | 0.1227 | 0 | 0 | 0.0245 | 0 | 0 | 0 | 0.1227 | 0 | 0 |
| BC_tissue | Esposito_2022 | 1.5557 | 27.4321 | 0 | 0 | 12.2202 | 0 | 0 | 1.1052 | 1.0207 | 0 | 0 | 0 | 0 | 0 | 0 | 0 | 0 | 0.4857 | 0 | 0 |
| BC_adjacent | Esposito_2022 | 9.4057 | 2.5529 | 0 | 0 | 10.4348 | 0 | 0 | 0 | 3.1312 | 0 | 0 | 0 | 0 | 0 | 0 | 0 | 0 | 0 | 0.2353 | 0 |
| BC_tissue | Esposito_2022 | 4.8356 | 34.7715 | 0 | 0.0054 | 0.6567 | 0 | 0 | 0 | 3.7718 | 0.6675 | 0.1628 | 0 | 0.2225 | 0 | 0 | 0 | 0 | 0.0163 | 0 | 0 |
| BC_adjacent | Esposito_2022 | 33.2335 | 12.5962 | 0.834 | 0 | 4.769 | 0 | 0 | 0 | 0 | 0.0428 | 0 | 0 | 2.9726 | 0 | 0 | 0 | 0 | 0.8127 | 0 | 0 |
| BC_adjacent | Kartti_2023 | 0.1992 | 0.7118 | 0.0664 | 0 | 0.0027 | 0 | 0 | 0 | 0 | 0 | 0.3904 | 0.3851 | 0 | 0 | 0.0159 | 0.0903 | 0 | 0 | 0 | 0 |
| BC_adjacent | Kartti_2023 | 0.0765 | 0.4483 | 0.0036 | 0 | 0.266 | 0 | 0 | 0 | 0.0073 | 0.0401 | 0.594 | 1.1917 | 0 | 0 | 0.0474 | 0.0146 | 0 | 0 | 0 | 0 |
| BC_adjacent | Kartti_2023 | 0.0552 | 0.8426 | 0.0074 | 0.0074 | 0 | 0 | 0 | 0 | 0 | 0.0589 | 0.7065 | 0.1177 | 0 | 0 | 0 | 0.0037 | 0 | 0.0221 | 0 | 0 |
| BC_tissue | Kartti_2023 | 0.0481 | 0.9698 | 0.0138 | 0.0069 | 0 | 0.4539 | 0 | 0 | 0 | 1.8181 | 1.4925 | 0.7864 | 0 | 0 | 0.1857 | 0 | 0 | 0 | 0 | 0 |
| BC_adjacent | Kartti_2023 | 0.0082 | 0.4309 | 0.0355 | 0 | 0 | 0 | 0 | 0 | 0 | 0.3109 | 0.7227 | 0.1909 | 0.0245 | 0 | 0.1091 | 0.0627 | 0 | 0 | 0 | 0 |
| BC_adjacent | Kartti_2023 | 0.0224 | 0.0583 | 0 | 0.0269 | 0 | 0 | 0 | 0.1165 | 0 | 0 | 0.6588 | 0.6857 | 0 | 0 | 0 | 0 | 0 | 0 | 0 | 0 |
| BC_adjacent | Kartti_2023 | 0.2709 | 0.1428 | 0.0861 | 0 | 0 | 0 | 0 | 0 | 0 | 0 | 1.088 | 0.3487 | 0 | 0.2667 | 0 | 0.0336 | 0 | 0 | 0.8212 | 0 |
| BC_adjacent | Kartti_2023 | 0.0153 | 0 | 0.4106 | 0 | 0 | 0 | 0 | 0 | 0 | 0 | 0.4221 | 0.4566 | 0.0537 | 0.188 | 0 | 0 | 0 | 0 | 0 | 0 |
| BC_adjacent | Kartti_2023 | 0.106 | 0.1927 | 0.0337 | 0 | 0 | 0 | 0 | 0 | 0 | 0.6167 | 1.2383 | 0 | 0 | 0 | 0 | 0 | 0 | 0 | 0.0048 | 0 |
| BC_adjacent | Kartti_2023 | 0.0039 | 0.2941 | 0.0039 | 0.0314 | 0 | 0 | 0 | 0 | 0 | 0.0863 | 0.4783 | 0.5019 | 4.0698 | 0 | 0.0314 | 0 | 0 | 0 | 0.2823 | 0 |
| BC_adjacent | Kartti_2023 | 0.1308 | 0.1398 | 0.0135 | 0 | 0 | 0 | 0.009 | 0 | 0 | 0.0226 | 0.76 | 0.3766 | 0 | 0 | 0.018 | 0.0135 | 0.0451 | 0 | 0.0586 | 0 |
| BC_adjacent | Kartti_2023 | 0.0651 | 0.0566 | 0.0255 | 0 | 0 | 0 | 0 | 0 | 0 | 0.2235 | 0.5544 | 0.9193 | 0 | 0.0028 | 0 | 0 | 0 | 0 | 0.1726 | 0 |
| BC_adjacent | Kartti_2023 | 0.4439 | 1.2663 | 0 | 0.0243 | 0 | 0.1213 | 0 | 0.5846 | 0 | 0 | 2.6853 | 0.7326 | 0 | 0.2668 | 0.0679 | 0.2911 | 0 | 0.0121 | 0 | 0 |
| BC_adjacent | Kartti_2023 | 0.3295 | 0.2508 | 0 | 0 | 0 | 0 | 0 | 0 | 0 | 0 | 3.2659 | 1.7554 | 0 | 0 | 0 | 0 | 0 | 0 | 0 | 0 |
| BC_tissue | Kartti_2023 | 0.1469 | 2.2529 | 0.1001 | 0.0532 | 0 | 0.0426 | 0 | 0 | 0.0298 | 2.0187 | 1.0221 | 0.3471 | 0.049 | 0 | 0 | 0.4493 | 0.1767 | 0 | 0 | 0 |
| BC_adjacent | Kartti_2023 | 0.4215 | 0.8181 | 0.062 | 0 | 0 | 0 | 0 | 0 | 0 | 0.564 | 0.3099 | 0.9173 | 0 | 0 | 0 | 0.2107 | 0 | 0 | 0.093 | 0 |
| BC_adjacent | Kartti_2023 | 0.0672 | 0.0651 | 0.0065 | 0 | 0 | 0 | 0 | 0 | 0 | 0 | 0.0564 | 0.3775 | 0.0239 | 0 | 0 | 0 | 0 | 0 | 0.0586 | 0 |
| BC_adjacent | Kartti_2023 | 0 | 0.0288 | 0.0192 | 0 | 0 | 0 | 0 | 0 | 0 | 0 | 0.5696 | 0.4832 | 0 | 0 | 0 | 0 | 0 | 0 | 0 | 0 |
| BC_adjacent | Kartti_2023 | 0.03 | 0.0629 | 0.018 | 0 | 0 | 0 | 0.006 | 0 | 0.015 | 0.3657 | 1.2798 | 0.8722 | 0.1858 | 0 | 0 | 0.003 | 0.051 | 0.0809 | 0.4586 | 0 |
| BC_adjacent | Kartti_2023 | 0.1811 | 0.1051 | 0.2246 | 0 | 0.0217 | 0 | 0 | 0 | 0 | 0.1703 | 1.1483 | 0.0072 | 0.029 | 0 | 0 | 0 | 0 | 0.0254 | 0 | 0 |
| BC_adjacent | Kartti_2023 | 0.2609 | 0.4011 | 0.1851 | 0 | 0 | 0 | 0 | 0 | 0 | 0 | 0.0757 | 0.9845 | 0 | 0 | 0 | 0.143 | 0 | 0 | 0 | 0 |
| BC_adjacent | Kartti_2023 | 0.162 | 1.181 | 0 | 0 | 0 | 0.0989 | 0 | 0 | 0 | 4.8256 | 2.9882 | 0.3241 | 0 | 0 | 0.1923 | 0.8377 | 0.6235 | 0 | 0.3845 | 0 |
| BC_adjacent | Kartti_2023 | 0.1426 | 0.7268 | 0.1173 | 0.1702 | 0 | 0 | 0 | 0 | 0 | 2.8497 | 1.5341 | 0 | 0 | 0 | 0 | 0.3427 | 0.184 | 0 | 0.0667 | 0 |
| BC_adjacent | Kartti_2023 | 0.2238 | 0.2709 | 0 | 0.07 | 0.5662 | 0.0929 | 0 | 0 | 0 | 0.0335 | 0.5845 | 0.5312 | 0.1279 | 0.0776 | 0.0411 | 0.688 | 0 | 0 | 0 | 0 |
| BC_adjacent | Kartti_2023 | 0.4593 | 0.4332 | 0.0019 | 0 | 0.0056 | 0 | 0 | 0 | 0 | 0 | 1.7047 | 0 | 0.0373 | 0.2894 | 0.0597 | 0.7188 | 0 | 0 | 0.0224 | 0 |
| BC_tissue | Kartti_2023 | 0.103 | 2.8304 | 0 | 0.0687 | 0 | 0 | 0 | 0 | 0 | 0.4156 | 0.8897 | 0.6939 | 0 | 0 | 0 | 0.5822 | 0.1563 | 0 | 0 | 0 |
| BC_adjacent | Kartti_2023 | 0.058 | 0.4331 | 0.0893 | 0.0491 | 0 | 0 | 0 | 0 | 0 | 0.0335 | 0.259 | 0.8171 | 0.3461 | 0.1273 | 0.0246 | 0.9645 | 0.3036 | 0 | 0.5135 | 0 |
| BC_adjacent | Kartti_2023 | 0.068 | 5.2964 | 0.7256 | 0 | 0.3103 | 0 | 0 | 0 | 0 | 4.2383 | 0 | 0 | 0.464 | 0 | 0 | 2.4842 | 0 | 0 | 0 | 0 |
| BC_adjacent | Kartti_2023 | 0.0644 | 0.3374 | 0 | 0.0103 | 0 | 0.018 | 0 | 0 | 0 | 0.5203 | 0.7495 | 0.4353 | 0 | 0.0103 | 0.2447 | 0.9839 | 0.0567 | 0.0773 | 0 | 0 |
| BC_adjacent | Kartti_2023 | 0.1775 | 2.4909 | 0 | 0 | 0.0093 | 0.576 | 0 | 0 | 0 | 5.9283 | 1.3046 | 0.6103 | 0 | 0 | 0.3954 | 1.8028 | 0 | 0.0093 | 0.0374 | 0 |
| BC_adjacent | Kartti_2023 | 0.0017 | 1.0166 | 0.0017 | 0 | 0 | 0.5603 | 0.0139 | 0 | 0 | 1.0998 | 0.5343 | 0.1978 | 0.085 | 0 | 0.0711 | 0.6124 | 0 | 0 | 0.3383 | 0 |
| BC_adjacent | Kartti_2023 | 0.0889 | 0.5263 | 0.0047 | 0 | 0.0866 | 0.1497 | 0 | 0 | 0.0351 | 0.414 | 2.7251 | 0.3556 | 0.0374 | 0.0187 | 0 | 0 | 0 | 0.0257 | 0.1146 | 0 |
| BC_adjacent | Kartti_2023 | 0.0197 | 0.1316 | 0 | 0 | 0 | 0.0461 | 0 | 0.2041 | 0 | 0.0461 | 1.356 | 0.3357 | 0 | 0 | 0 | 0.1316 | 0 | 0 | 0.2106 | 0 |
| BC_adjacent | Kartti_2023 | 2.0395 | 2.1231 | 0.0299 | 0.0239 | 0 | 0.006 | 0 | 0 | 0 | 0.4599 | 1.1108 | 1.1228 | 0 | 0.006 | 0 | 1.6513 | 0 | 0.003 | 0.3046 | 0 |
| BC_adjacent | Kartti_2023 | 0 | 0.3351 | 0.055 | 0.0035 | 0 | 0 | 0 | 0 | 0 | 0.0479 | 0.8564 | 0 | 0 | 0 | 0 | 0 | 0 | 0 | 0 | 0 |
| BC_adjacent | Kartti_2023 | 0.1444 | 0.1382 | 0.2641 | 0 | 0 | 0 | 0.0154 | 0 | 0 | 0.0522 | 1.8674 | 0 | 0.0031 | 0 | 0 | 0.1413 | 0 | 0 | 0 | 0 |
| BC_adjacent | Kartti_2023 | 0.0133 | 0.2519 | 0 | 0.0033 | 0 | 0.0232 | 0 | 0.285 | 0 | 0.0464 | 1.5114 | 0.7424 | 0 | 0 | 0 | 0.0199 | 0.0033 | 0.0133 | 0.3911 | 0.0365 |
| BC_adjacent | Kartti_2023 | 0.6993 | 0.7177 | 0 | 0.0046 | 0 | 0 | 0 | 0 | 0 | 0.1376 | 0.282 | 0.8874 | 0.0115 | 0.0229 | 0.0321 | 0.376 | 0 | 0.0367 | 0.0527 | 0.3577 |
| BC_adjacent | Kartti_2023 | 0.2651 | 0.4338 | 0.1188 | 0.0224 | 0 | 0 | 0 | 0.0516 | 0 | 0 | 1.2533 | 0.1016 | 0 | 0 | 0.0155 | 0 | 0 | 0 | 0 | 0 |
| BC_adjacent | Kartti_2023 | 0.1752 | 1.242 | 0.0833 | 0.0017 | 0 | 0 | 0 | 0 | 0 | 0.7129 | 1.8317 | 0.7372 | 0 | 0.0416 | 0.2342 | 1.0668 | 0.1353 | 0.0052 | 0.1561 | 0 |
| BC_adjacent | Kartti_2023 | 0 | 0.3836 | 0.034 | 0 | 0 | 0 | 0 | 0 | 0.008 | 0.1219 | 2.032 | 0.7413 | 0 | 0 | 0 | 0 | 0 | 0.0939 | 0 | 0 |
| BC_adjacent | Kartti_2023 | 0.0034 | 0.0448 | 0.0138 | 0.2793 | 0.0138 | 0 | 0 | 0 | 0 | 0.0724 | 0.4725 | 0.069 | 0 | 0 | 0 | 0 | 0 | 0 | 0.0724 | 0 |
| BC_adjacent | Kartti_2023 | 0.5648 | 3.5534 | 0.1808 | 0.5892 | 0 | 0 | 0 | 0.0163 | 0 | 0 | 2.3587 | 1.4892 | 0 | 0 | 0.7009 | 0.0264 | 0 | 0 | 0.5648 | 0 |
| BC_tissue | Kartti_2023 | 0.0396 | 0.9812 | 0.2163 | 0 | 0 | 0 | 0 | 0 | 0 | 0.3508 | 3.9724 | 2.3291 | 0 | 0 | 0 | 0 | 0 | 0 | 0 | 0 |
| BC_adjacent | Kartti_2023 | 0.5648 | 3.5516 | 0 | 0.3905 | 0.1904 | 0.2792 | 0 | 0 | 0 | 5.9204 | 0 | 0.1404 | 0.0855 | 0.1501 | 0 | 0.405 | 0.242 | 0.2307 | 0 | 0 |
| BC_adjacent | Kartti_2023 | 0.0261 | 2.2968 | 0 | 0.0681 | 0 | 0 | 0 | 0 | 0 | 0 | 4.1045 | 0.497 | 0 | 0.0481 | 0.1984 | 0.4549 | 0 | 0 | 0.3247 | 0 |
| BC_adjacent | Kartti_2023 | 0.0406 | 0 | 0.0736 | 0.0076 | 0 | 0 | 0 | 0 | 0 | 0 | 1.1856 | 0.1295 | 0 | 0 | 0 | 0.0305 | 0 | 0 | 0 | 0 |
| BC_adjacent | Kartti_2023 | 0.5927 | 1.741 | 0.1822 | 0.2027 | 0.0051 | 0.0847 | 0 | 0.2258 | 0 | 0.1014 | 2.5107 | 0.4259 | 0 | 0 | 0 | 0.4234 | 0 | 0 | 0 | 0 |
| BC_adjacent | Kartti_2023 | 0.0032 | 0.7138 | 0.0714 | 0 | 0 | 0 | 0 | 0 | 0 | 0.5321 | 0.9603 | 0.7138 | 0 | 0 | 0 | 0 | 0 | 0 | 0.2758 | 0 |
| BC_adjacent | Kartti_2023 | 0.0269 | 0.8522 | 0 | 0 | 0 | 0 | 0 | 0 | 0.0354 | 1.4046 | 1.2244 | 0 | 0.064 | 0.064 | 2.7183 | 0 | 0 | 0 | 0.64 | 0 |
| BC_adjacent | Kartti_2023 | 0.5954 | 1.2062 | 0.2481 | 0.0057 | 0 | 0 | 0 | 0 | 0 | 0.2195 | 1.0993 | 0.9695 | 0.2844 | 2.4371 | 0.5382 | 0.1985 | 0 | 0 | 0 | 0 |
| BC_adjacent | Kartti_2023 | 0.1832 | 13.2202 | 0.0526 | 0.056 | 0.0017 | 0.1798 | 0 | 0.0899 | 0 | 0.4477 | 0.6326 | 0.4223 | 0 | 0 | 0.0237 | 0.1272 | 0.3087 | 0.1102 | 0.0085 | 0 |
| BC_adjacent | Kartti_2023 | 0.2232 | 3.1761 | 0 | 0.1997 | 0.0764 | 0.2305 | 0 | 0 | 0 | 7.7412 | 0 | 0 | 0.0279 | 0.138 | 0 | 1.646 | 0 | 0.1997 | 0.1542 | 0 |
| BC_tissue | Kartti_2023 | 0.008 | 0.0121 | 0.008 | 0 | 0 | 0.1208 | 0 | 0.1771 | 0 | 0.0322 | 0.9982 | 0.5796 | 0 | 0 | 0 | 0 | 0 | 0 | 0.2294 | 0.0201 |
| BC_tissue | Kartti_2023 | 0.4286 | 0.2959 | 0.051 | 0.0383 | 0 | 0.0306 | 0 | 0 | 0.0612 | 2.0332 | 0.4898 | 0.1225 | 0.4796 | 0.0842 | 0 | 0.1735 | 0 | 0 | 0.0179 | 0.0918 |
| BC_tissue | Kartti_2023 | 0.0978 | 0.0652 | 0.003 | 0 | 0 | 0.0919 | 0 | 0.1363 | 0 | 0.0059 | 2.2378 | 0.7617 | 0 | 0.0059 | 0 | 0 | 0 | 0 | 0.2253 | 0.0415 |
| BC_tissue | Kartti_2023 | 0.0344 | 0.0929 | 0 | 0.0138 | 0 | 0.0619 | 0 | 0.1479 | 0 | 0.0447 | 1.8747 | 0.7911 | 0 | 0 | 0 | 0 | 0 | 0 | 0.3405 | 0.031 |
| BC_tissue | Kartti_2023 | 0.0114 | 0.0741 | 0 | 0 | 0 | 0.1597 | 0 | 0.2366 | 0 | 0.0342 | 1.6537 | 0.7955 | 0 | 0.0057 | 0 | 0 | 0 | 0 | 0.4476 | 0 |
| BC_tissue | Kartti_2023 | 1.2696 | 2.7624 | 0.0864 | 0.024 | 0 | 0.0288 | 0 | 0 | 0 | 0 | 0.792 | 2.2728 | 0 | 0 | 0 | 0 | 0 | 0 | 0.1512 | 0 |
| BC_tissue | Kartti_2023 | 0.4674 | 1.2471 | 0.0135 | 0.0022 | 0 | 0 | 0 | 0 | 0 | 0 | 0.4764 | 0.1281 | 0.0404 | 0 | 0 | 0 | 0.0966 | 0 | 0 | 0 |
| BC_tissue | Kartti_2023 | 0.0699 | 0.2291 | 0.0039 | 0.0815 | 0 | 0 | 0 | 0 | 0 | 0.0505 | 1.9336 | 0.2601 | 0 | 0.2213 | 0 | 0.0349 | 0 | 0 | 0 | 0 |
| BC_tissue | Kartti_2023 | 0.4053 | 0.9927 | 0.2026 | 0 | 0 | 0 | 0 | 0 | 0 | 0.1138 | 2.6251 | 0.9722 | 0 | 0.1138 | 0.0751 | 0.3233 | 0 | 0.1161 | 0.1389 | 0 |
| BC_tissue | Kartti_2023 | 0.1706 | 1.6368 | 0 | 0.2434 | 0.046 | 0 | 0 | 0 | 0 | 0 | 1.5179 | 0.9583 | 0.1514 | 0 | 0 | 1.4643 | 0.4408 | 0 | 0 | 0 |
| BC_tissue | Kartti_2023 | 0.0489 | 0.0285 | 0.0163 | 0 | 0 | 0 | 0 | 0 | 0 | 0.0285 | 0.1263 | 0.4849 | 0 | 0 | 0 | 0.0489 | 0 | 0 | 0 | 0 |
| BC_tissue | Kartti_2023 | 0.1353 | 0.2769 | 0.0666 | 0.0229 | 0 | 0 | 0 | 0 | 0 | 0.077 | 0.6641 | 0.3581 | 0.0104 | 0.1957 | 0 | 0.1166 | 0.0333 | 0 | 0.0042 | 0.0999 |
| BC_tissue | Kartti_2023 | 3.6618 | 3.2557 | 0.2062 | 0.4209 | 0 | 0.1242 | 0 | 0 | 0 | 0.7555 | 1.5868 | 0.7576 | 0.2631 | 2.0498 | 0 | 0.4988 | 0.2946 | 0 | 0 | 0 |
| BC_tissue | Kartti_2023 | 0.2607 | 0.538 | 0.1248 | 0.0638 | 0.0471 | 0.025 | 0 | 0 | 0 | 0.6184 | 0.4631 | 0.4909 | 0 | 0.0416 | 0 | 0 | 0.1193 | 0 | 0.0444 | 0 |
| BC_tissue | Kartti_2023 | 2.2865 | 0.7389 | 0.01 | 0.0849 | 0.0399 | 0 | 0 | 0 | 0.01 | 0.2995 | 1.8072 | 0.639 | 0 | 0 | 0 | 0.5392 | 0 | 0 | 0.3045 | 0 |
| BC_tissue | Kartti_2023 | 1.1064 | 0.6237 | 0 | 0 | 0.0054 | 0 | 0 | 0 | 0 | 0.4122 | 4.5667 | 1.3125 | 0.0217 | 0 | 0 | 0.1681 | 0 | 0 | 0.0651 | 0 |
| BC_tissue | Kartti_2023 | 0 | 0.516 | 0 | 0.0794 | 0 | 0 | 0 | 0 | 0 | 23.7273 | 2.3023 | 2.213 | 0 | 0 | 0 | 0.3672 | 0 | 0 | 0 | 0 |
| BC_tissue | Kartti_2023 | 0.7475 | 1.3833 | 0.0703 | 0 | 0 | 0 | 0 | 0 | 0 | 0.2204 | 3.562 | 0.5591 | 0 | 0.3003 | 0 | 0.2779 | 0 | 0.2268 | 0 | 0 |
| BC_tissue | Kartti_2023 | 0.3212 | 0.0347 | 0.1563 | 0 | 0 | 0 | 0 | 0 | 0 | 0.1302 | 2.7346 | 0.6468 | 0 | 0.1129 | 0 | 0 | 0 | 0 | 0 | 0 |
| BC_tissue | Kartti_2023 | 0.3334 | 0.1425 | 0.2422 | 0.1681 | 0 | 0 | 0 | 0.2507 | 0 | 0 | 1.4731 | 1.7923 | 0 | 0 | 0 | 0 | 0 | 0 | 0 | 0 |
| BC_tissue | Kartti_2023 | 1.7694 | 0.261 | 0.109 | 0 | 0.0258 | 0 | 0 | 0 | 0 | 0 | 1.4253 | 0.4101 | 0 | 0.1405 | 0 | 0 | 0 | 0 | 0 | 0 |
| BC_tissue | Kartti_2023 | 0.0071 | 1.5012 | 0 | 1.2255 | 0 | 0 | 0 | 0 | 0 | 0.8413 | 0 | 0 | 0 | 0.6387 | 0 | 0 | 0 | 0 | 0 | 0 |
| BC_tissue | Kartti_2023 | 0.0092 | 0.1892 | 0.0129 | 0.0643 | 0 | 0 | 0 | 0 | 0 | 0.5969 | 2.9259 | 2.4961 | 0 | 0 | 0 | 0 | 0.0404 | 0 | 0 | 0 |
| BC_tissue | Kartti_2023 | 1.3805 | 1.3727 | 0 | 0.2 | 0 | 0.0784 | 0 | 0 | 0.3961 | 0 | 4.6553 | 1.6707 | 0 | 0 | 0 | 0 | 0 | 0 | 0 | 0.8393 |
| BC_tissue | Kartti_2023 | 0.0714 | 0.0842 | 0.0918 | 0.1122 | 0.0332 | 0 | 0 | 0 | 0 | 4.0087 | 1.6779 | 0 | 0.0204 | 0 | 0.0127 | 0 | 0.2729 | 0 | 0 | 0.3902 |
| BC_tissue | Kartti_2023 | 0.0594 | 0.0054 | 0.0162 | 0 | 0.0243 | 0 | 0 | 0 | 0 | 1.5754 | 0.9782 | 0.3648 | 0.1486 | 0.0703 | 0 | 0.073 | 0 | 0 | 0 | 0 |
| BC_tissue | Kartti_2023 | 0.0475 | 1.664 | 0 | 0 | 0 | 0 | 0 | 0 | 0 | 0 | 0.2048 | 0.4791 | 0 | 0 | 0 | 0 | 0 | 0 | 0.2377 | 0 |
| BC_tissue | Kartti_2023 | 0.2294 | 0.6642 | 0.012 | 0.1994 | 0 | 0 | 0 | 0 | 0 | 0.0405 | 3.3931 | 0 | 0 | 0.1095 | 0 | 0 | 0 | 0 | 0 | 0.5263 |
| BC_tissue | Kartti_2023 | 0.2103 | 0.3836 | 0 | 0 | 0 | 0 | 0 | 0 | 0.0323 | 0.9659 | 4.5013 | 1.4627 | 0.1641 | 0 | 0 | 0.8342 | 1.0837 | 0.1479 | 0.3512 | 0 |
| BC_tissue | Kartti_2023 | 0.1595 | 1.3198 | 0.0348 | 0.0261 | 0.2146 | 0 | 0 | 0.0058 | 0.1421 | 2.0479 | 0 | 0 | 0.2553 | 0.0609 | 0 | 0.1276 | 0.0899 | 0 | 0 | 0 |
| BC_tissue | Kartti_2023 | 0.1433 | 2.1191 | 0.2242 | 0.0139 | 0 | 0 | 0 | 0 | 0 | 3.7598 | 0.7649 | 0 | 0.2727 | 0.0878 | 0 | 0.3605 | 0 | 0.6193 | 0 | 0 |
| BC_tissue | Kartti_2023 | 0.0469 | 0.4059 | 0.0193 | 0.0028 | 1.9714 | 0.0166 | 0 | 0 | 0 | 1.1017 | 0.5605 | 0.1822 | 0.011 | 0.0497 | 0.37 | 0.2292 | 0.1822 | 0 | 0.0552 | 0 |
| BC_tissue | Kartti_2023 | 0.149 | 1.1863 | 0.2206 | 0 | 0.0029 | 0.1146 | 0 | 0 | 0.0831 | 3.5647 | 1.1376 | 0.5674 | 0.1977 | 0.0831 | 0.3954 | 1.2751 | 0.0716 | 0 | 0.1289 | 0 |
| BC_tissue | Kartti_2023 | 0.2393 | 0.2479 | 0 | 0 | 0 | 0.0058 | 0 | 0 | 0 | 3.2896 | 1.0062 | 0.3489 | 0 | 0.0865 | 0.0865 | 0.3806 | 0.1557 | 0 | 0 | 0 |
| BC_tissue | Kartti_2023 | 0.4304 | 1.6157 | 0.2963 | 0.0776 | 0 | 0 | 0 | 0.2505 | 0 | 0.381 | 2.1202 | 0.9172 | 0 | 0 | 0 | 0.5009 | 0 | 0 | 0 | 0 |
| BC_tissue | Kartti_2023 | 0.6166 | 0.8071 | 0.043 | 0.0594 | 0 | 0 | 0 | 0.2417 | 0 | 0.0389 | 1.6286 | 0.8604 | 0.1229 | 0 | 0 | 1.3828 | 0.3933 | 0 | 0 | 0 |
| BC_tissue | Kartti_2023 | 0.0712 | 0.0522 | 0.121 | 0.038 | 0 | 0 | 0 | 0 | 0 | 0.1139 | 6.0027 | 0.2918 | 0.1732 | 0.0047 | 0.0142 | 0.465 | 0.0688 | 0 | 0.1732 | 0 |
| BC_tissue | Kartti_2023 | 0.2343 | 0.2965 | 0.0829 | 0.0539 | 0 | 0.1493 | 0 | 0.3214 | 0.027 | 0.3939 | 2.2351 | 0 | 0 | 0 | 0 | 0.1348 | 0.0456 | 0 | 0 | 0 |
| BC_tissue | Kartti_2023 | 1.162 | 1.8613 | 0.109 | 0.2599 | 0.6137 | 0.0134 | 0 | 0 | 0 | 3.258 | 0.4729 | 0 | 0.2716 | 0.0302 | 0 | 0.2515 | 2.698 | 0.0822 | 0 | 0 |
| BC_tissue | Kartti_2023 | 0.0644 | 0.7128 | 0 | 0 | 0 | 0.002 | 0 | 0 | 0 | 0.0765 | 7.5247 | 0.6584 | 0.1671 | 0 | 0.143 | 0.1389 | 0 | 0 | 0.1289 | 0.6605 |
| BC_tissue | Kartti_2023 | 0 | 0.1869 | 0.1189 | 0 | 0 | 0.3285 | 0 | 0 | 0 | 0 | 1.6198 | 0 | 0 | 0 | 0 | 0 | 0 | 0 | 0 | 0 |
| BC_tissue | Kartti_2023 | 0.3687 | 2.8068 | 0.0658 | 0.1674 | 0 | 0 | 0 | 0.3085 | 0 | 15.3586 | 3.6722 | 0.8936 | 0 | 0 | 0 | 0 | 0 | 0 | 0 | 0 |
| BC_tissue | Kartti_2023 | 0.6731 | 0.7644 | 0 | 0.0557 | 0.4346 | 0 | 0 | 0 | 0.2674 | 0.0022 | 3.0822 | 0.6619 | 0 | 0 | 0 | 0.2407 | 0 | 0 | 0 | 0 |
| BC_tissue | Kartti_2023 | 0.0103 | 0.6049 | 0.0533 | 0.1959 | 0.0584 | 0.0086 | 0 | 0 | 0 | 0.2285 | 0.8008 | 0.5602 | 0 | 0 | 0 | 0 | 0 | 0 | 0.0034 | 0 |
| BC_adjacent | Kartti_2023 | 0.5238 | 0.7502 | 0.02 | 3.7577 | 0.0377 | 0 | 0 | 0 | 0 | 0.0777 | 0 | 0.1443 | 0.1243 | 0.7236 | 0.0577 | 0.2264 | 0.5216 | 0.0222 | 0 | 0 |
| BC_adjacent | Kartti_2023 | 1.5595 | 0.8865 | 0.2932 | 0 | 0 | 0 | 0 | 0 | 0 | 0.0069 | 1.6532 | 0 | 0 | 0.1631 | 2.7843 | 0 | 0 | 0 | 0 | 0 |
| BC_adjacent | Kartti_2023 | 0.0937 | 0.015 | 0 | 0 | 0 | 0.0787 | 0 | 0.1574 | 0 | 0.045 | 0.9932 | 0.6634 | 0 | 0 | 0 | 0 | 0.015 | 0 | 0.3823 | 0 |
| BC_tissue | Kartti_2023 | 0.1556 | 1.1292 | 0.0058 | 0.0192 | 0 | 0.1018 | 0 | 0 | 0 | 0.6241 | 1.3942 | 0.5339 | 0 | 0 | 0 | 0.0096 | 0 | 0 | 0 | 0.1709 |
| BC_tissue | Kartti_2023 | 0.0955 | 0.4917 | 0.0202 | 0 | 0 | 0 | 0 | 0.1302 | 0 | 0 | 0.3934 | 1.0616 | 0 | 0 | 0 | 0 | 0 | 0 | 0 | 0 |
| BC_tissue | Kartti_2023 | 0.4427 | 6.0125 | 0 | 0.7177 | 0 | 0.5068 | 0 | 0.5906 | 0 | 0 | 1.9383 | 1.2546 | 0.592 | 0.0956 | 0 | 0 | 0 | 0 | 0 | 0 |
| BC_adjacent | German_2023 | 2.6681 | 0 | 2.7544 | 0 | 0.0009 | 0 | 0.0036 | 0 | 11.6686 | 0 | 0 | 0 | 0.0009 | 0 | 0 | 0 | 0.0009 | 0 | 0 | 0 |
| BC_adjacent | German_2023 | 0.0075 | 0 | 0.5315 | 0 | 0 | 0 | 0 | 0.0092 | 0 | 0 | 0 | 0 | 0 | 0 | 0 | 0 | 0 | 0 | 0 | 0 |
| BC_adjacent | German_2023 | 8.8078 | 0 | 0.8505 | 0 | 0 | 0 | 0.0023 | 1.1458 | 0.0011 | 0 | 0 | 0 | 0 | 0.3905 | 0 | 0 | 0 | 0 | 0 | 0 |
| BC_tissue | German_2023 | 2.5075 | 0.0007 | 0.1742 | 0 | 0 | 0 | 0.4779 | 0 | 0.0224 | 0 | 0 | 0 | 0 | 0.0014 | 0 | 0 | 0 | 0 | 0.0022 | 0 |
| BC_tissue | German_2023 | 0.8193 | 0.0053 | 0.5311 | 0 | 0 | 0 | 13.41 | 0 | 0 | 0 | 0 | 0 | 0.008 | 0.0027 | 0 | 0 | 1.4945 | 0 | 0 | 0 |
| BC_adjacent | German_2023 | 0.3366 | 0.0711 | 0.3375 | 0 | 0 | 0 | 0.1598 | 0.0003 | 0.0026 | 0 | 0 | 0 | 0.001 | 0 | 0 | 0 | 0.0013 | 0 | 0 | 0 |
| BC_adjacent | German_2023 | 7.2479 | 0.2153 | 0.5987 | 0 | 0 | 0 | 3.2668 | 0 | 0.0053 | 0 | 0 | 0 | 0 | 1.4653 | 0 | 0 | 0 | 0 | 0 | 0 |
| BC_adjacent | German_2023 | 0.3888 | 0.106 | 0.2386 | 0 | 0 | 0 | 2.7337 | 0 | 0.4006 | 0 | 0 | 0 | 0 | 0 | 0.9868 | 0.1149 | 0 | 0 | 0.0029 | 0 |
| BC_tissue | German_2023 | 0.0303 | 0 | 0.7668 | 0 | 0 | 0 | 10.6548 | 0 | 0.111 | 0 | 0 | 0 | 0 | 0.1211 | 0.3733 | 0 | 0 | 0 | 0 | 0 |
| BC_adjacent | German_2023 | 1.9674 | 0.0527 | 1.8812 | 0 | 0 | 0 | 0.1055 | 0 | 0.003 | 0 | 0 | 0 | 0.3702 | 0.3438 | 0 | 0 | 0 | 0 | 0.001 | 0 |
| BC_adjacent | German_2023 | 1.2607 | 0 | 0.0564 | 0 | 0 | 0 | 1.5949 | 0 | 0.0014 | 0 | 0.0406 | 0 | 0.0585 | 0.0612 | 0 | 0 | 0 | 0 | 0 | 0 |
| BC_tissue | German_2023 | 1.0872 | 0 | 0.5458 | 0 | 0 | 0 | 5.0081 | 0 | 0.1703 | 0 | 0 | 0 | 0 | 0.0218 | 0 | 0 | 0 | 0 | 0.0131 | 0 |
| BC_adjacent | German_2023 | 1.6939 | 0.7315 | 1.0013 | 0 | 0.0052 | 0 | 0.8794 | 0 | 0.0026 | 0 | 0 | 0 | 0.0026 | 0.0052 | 0.0026 | 0.0026 | 0 | 0 | 0 | 0 |
| BC_tissue | German_2023 | 1.1488 | 0.0436 | 0.2784 | 0 | 0 | 0 | 0.1122 | 0 | 0.04 | 0 | 0 | 0 | 0.0006 | 0 | 0 | 0 | 0 | 0 | 0 | 0.0003 |
| BC_adjacent | German_2023 | 0.116 | 0.009 | 0.9442 | 0 | 0.0015 | 0 | 2.4049 | 0 | 0.1114 | 0 | 0 | 0.0015 | 0 | 0 | 0.0015 | 0 | 0 | 0 | 0 | 0 |
| BC_tissue | German_2023 | 1.2002 | 0.0033 | 1.3236 | 0 | 0 | 0 | 7.3281 | 1.1536 | 0.6801 | 0 | 0 | 0 | 0.0133 | 0 | 0 | 0 | 0.0267 | 0 | 0 | 0 |
| BC_adjacent | German_2023 | 1.0282 | 0 | 0.1293 | 0 | 0.0006 | 0 | 0.8126 | 0.206 | 1.0797 | 0 | 0 | 0 | 0.0012 | 0 | 0 | 0 | 0 | 0 | 0.0018 | 0 |
| BC_tissue | German_2023 | 0.083 | 0.0917 | 0.334 | 0 | 0 | 0 | 1.3251 | 0.0873 | 0.8885 | 0 | 0.0022 | 0 | 0 | 0 | 0 | 0 | 0 | 0 | 0 | 0 |
| BC_adjacent | German_2023 | 0.0926 | 0.0301 | 0.2296 | 0 | 0 | 0 | 1.6841 | 0.0036 | 0.0012 | 0 | 0 | 0 | 0 | 0.0012 | 0.0012 | 0 | 0.0024 | 0 | 0 | 0 |
| BC_tissue | German_2023 | 0.253 | 0.0342 | 0.0507 | 0 | 0 | 0 | 0.4061 | 0 | 0.9469 | 0 | 0 | 0 | 0.0284 | 0 | 0.0003 | 0 | 0.1016 | 0 | 0 | 0 |
| BC_adjacent | German_2023 | 0.0731 | 0.0012 | 0.0875 | 0 | 0 | 0 | 6.0914 | 0 | 0.0587 | 0 | 0 | 0 | 0.0611 | 0.0012 | 0 | 0 | 0.0012 | 0 | 0.0024 | 0 |
| BC_tissue | German_2023 | 0.8912 | 0.6895 | 0.0624 | 0.0093 | 0 | 0 | 0.3314 | 0.0004 | 0.0673 | 0 | 0 | 0 | 6.2147 | 0.0385 | 0.0004 | 0 | 5.7974 | 0 | 0 | 0 |
| BC_tissue | German_2023 | 2.6606 | 0.0424 | 0.239 | 0 | 0 | 0 | 2.3106 | 0 | 0 | 0 | 0 | 0 | 0.0343 | 0.1773 | 0.6756 | 0 | 0.5164 | 0 | 0 | 0 |
| BC_adjacent | German_2023 | 0.4437 | 0.0025 | 0.0025 | 0 | 0 | 0 | 1.6303 | 0 | 0.0837 | 0 | 0 | 0 | 0 | 0 | 0 | 0 | 0 | 0 | 0 | 0 |
| BC_tissue | German_2023 | 0.1601 | 0 | 0.0946 | 0 | 0 | 0 | 1.1525 | 0.0049 | 0.2596 | 0 | 0 | 0 | 0.0024 | 0 | 0 | 0.0412 | 0.0024 | 0 | 0 | 0 |
| BC_adjacent | German_2023 | 0.0021 | 0.0432 | 0.0279 | 0 | 0 | 0 | 0.005 | 0.0003 | 0.0008 | 0 | 0 | 0 | 0.0003 | 0 | 0 | 0 | 0 | 0 | 1.1528 | 0 |
| BC_tissue | German_2023 | 0.745 | 0.6045 | 7.3567 | 0 | 0.2202 | 0 | 3.6362 | 0 | 0.0141 | 0 | 0 | 0 | 0 | 0.0047 | 0.5998 | 0 | 0 | 0 | 0.0047 | 0 |
| BC_adjacent | German_2023 | 0.0058 | 0 | 0.0712 | 0 | 0 | 0 | 0.8928 | 0 | 0 | 0 | 0 | 0 | 0 | 0 | 0 | 0 | 0 | 0 | 0.0023 | 0 |
| BC_adjacent | German_2023 | 1.8163 | 0.001 | 0.3222 | 0 | 0 | 0 | 0.1828 | 0 | 0.0114 | 0 | 0 | 0 | 0 | 0.228 | 0 | 0 | 0 | 0 | 0 | 0 |
| BC_adjacent | German_2023 | 0.5588 | 0.4183 | 0.3289 | 0 | 0 | 0 | 0.0851 | 0 | 0.0015 | 0 | 0 | 0 | 0.001 | 0 | 0 | 0 | 0 | 0 | 0.0003 | 0 |
| BC_adjacent | German_2023 | 0.8582 | 0.0015 | 0.1887 | 0 | 0 | 0 | 0.4278 | 0 | 0.0004 | 0 | 0 | 0 | 0.0947 | 0 | 0 | 0 | 0.0678 | 0 | 0 | 0 |
| BC_adjacent | German_2023 | 0 | 0 | 0.0016 | 0 | 0.0391 | 0 | 0 | 0.2031 | 0.0032 | 0.1859 | 0 | 0 | 0 | 0 | 0 | 0 | 0 | 0 | 0 | 0 |
| BC_tissue | German_2023 | 1.5127 | 0 | 1.9282 | 0 | 0 | 0 | 2.7804 | 0 | 0.0639 | 0.0107 | 0 | 0 | 0.6498 | 0 | 0.0107 | 0 | 1.8323 | 0 | 0.0107 | 0.0107 |
| BC_adjacent | German_2023 | 0.2101 | 0 | 0.0906 | 0 | 0 | 0 | 0.0005 | 0 | 0 | 0.0759 | 0 | 0 | 0 | 0 | 0 | 0 | 0 | 0 | 0 | 0 |
| BC_adjacent | German_2023 | 0.668 | 0 | 0.0009 | 0 | 0.1811 | 0 | 0.3579 | 0 | 0.0068 | 0 | 0 | 0 | 0 | 0 | 0.0034 | 0 | 0.0009 | 0 | 0 | 0 |
| BC_adjacent | German_2023 | 0.3917 | 0.8698 | 0.0588 | 0.1236 | 0.0406 | 0 | 0.607 | 0 | 0.0242 | 0 | 0.0476 | 0 | 0.0026 | 0 | 0.0026 | 0 | 0.0545 | 0 | 0.1686 | 0 |
| BC_tissue | German_2023 | 0.262 | 0.0472 | 0.1255 | 0 | 0 | 0 | 0.5171 | 0 | 0.0079 | 0 | 0 | 0 | 0.0163 | 0 | 0.001 | 0 | 0.1171 | 0 | 0 | 0 |
| BC_adjacent | German_2023 | 0.0902 | 0 | 0.3867 | 0 | 0 | 0 | 5.3235 | 0 | 11.1498 | 0 | 0 | 0.348 | 0 | 0 | 0 | 0 | 0 | 0 | 0 | 0 |
| BC_tissue | German_2023 | 0.1683 | 0 | 0 | 0 | 0 | 0 | 11.1989 | 0.1813 | 0.0906 | 0 | 0 | 0 | 0 | 0 | 0 | 0 | 0.0129 | 0 | 0 | 0 |
| BC_tissue | German_2023 | 0.5228 | 0 | 0.057 | 0 | 0 | 0 | 3.2031 | 0.0285 | 0.076 | 0 | 0 | 0 | 0 | 0 | 3.8019 | 0 | 0 | 0 | 0.0095 | 0 |
| BC_adjacent | German_2023 | 6.8703 | 0.3155 | 0.545 | 0 | 0.0143 | 0 | 10.3844 | 0.1004 | 0.2008 | 0 | 0 | 0 | 0.1434 | 0.2582 | 0.0574 | 0 | 2.1084 | 0.1865 | 0 | 0 |
| BC_tissue | German_2023 | 4.622 | 0.0913 | 0.0456 | 0 | 0.1883 | 0 | 3.6434 | 0.0029 | 0.0742 | 0 | 0 | 0 | 0 | 0.1512 | 0 | 0 | 0.3024 | 0 | 0 | 0 |
| BC_adjacent | German_2023 | 6.9845 | 0.6294 | 0.5472 | 0 | 0 | 0 | 0.8662 | 0 | 0.0061 | 0 | 0 | 0 | 0.0012 | 0 | 0.0012 | 0 | 0 | 0 | 0 | 0 |
| BC_tissue | German_2023 | 0.762 | 0 | 0.8654 | 0 | 0 | 0 | 2.4282 | 0 | 0.2002 | 0 | 0 | 0 | 0.0129 | 0 | 0 | 0 | 0 | 0 | 0 | 0 |
| BC_adjacent | German_2023 | 1.4141 | 0.0145 | 0.0397 | 0.001 | 0 | 0 | 1.2273 | 0 | 0.9186 | 0 | 0 | 0 | 0.0019 | 0.001 | 0 | 0 | 0.001 | 0 | 0.0377 | 0 |
| BC_adjacent | German_2023 | 0.929 | 0.0017 | 2.5988 | 0 | 0 | 0.0009 | 0.0328 | 0 | 0.0009 | 0 | 0 | 0 | 0.0069 | 0.0009 | 1.1621 | 0 | 0.6726 | 0 | 0 | 0 |
| BC_tissue | German_2023 | 8.0762 | 0.252 | 0 | 0 | 0 | 0 | 0.7644 | 0 | 0.0111 | 0 | 0 | 0 | 0 | 0 | 2.9358 | 0 | 0.0831 | 0 | 0 | 0 |
| BC_adjacent | German_2023 | 0.6379 | 0.083 | 0.3146 | 0 | 0 | 0 | 1.6597 | 0.1176 | 0 | 0 | 0 | 0.0035 | 0 | 0 | 0 | 0 | 0 | 0 | 0 | 0 |
| BC_adjacent | German_2023 | 0.7861 | 0.0005 | 0.7178 | 0 | 0 | 0 | 0.0161 | 0 | 0.0012 | 0 | 0 | 0 | 0.0002 | 0 | 0 | 0 | 0 | 0 | 0 | 0 |
| BC_adjacent | German_2023 | 0.0463 | 0.0041 | 0.0016 | 0 | 0 | 0 | 0.7244 | 0 | 0 | 0 | 0 | 0 | 0.0008 | 0 | 0 | 0 | 0 | 0 | 0 | 0 |
| BC_tissue | German_2023 | 0.4407 | 0 | 0.3815 | 0 | 0 | 0 | 12.3684 | 0 | 0.3945 | 0 | 0 | 0 | 0.0012 | 0.0012 | 0 | 0 | 0 | 0 | 0 | 0 |
| BC_adjacent | German_2023 | 0.124 | 0.1693 | 0.1349 | 0 | 0 | 0 | 2.9391 | 0.0812 | 0.0006 | 0.0006 | 0 | 0 | 0.0003 | 0 | 0 | 0.0364 | 0 | 0 | 0.0003 | 0 |
| BC_adjacent | German_2023 | 0.6306 | 0.309 | 0.4572 | 0 | 0 | 0 | 0.4351 | 0.09 | 6.7148 | 0 | 0 | 0.0007 | 0.0029 | 0.0022 | 0 | 0 | 0 | 0 | 0.0015 | 0 |
| BC_adjacent | German_2023 | 3.2055 | 1.202 | 0.0927 | 0 | 0.0008 | 0 | 0.0309 | 0 | 0 | 0.0017 | 0 | 0 | 0.3464 | 0 | 0 | 0 | 0.1311 | 0 | 0 | 0 |
| BC_adjacent | German_2023 | 0.5665 | 0.0039 | 0.0077 | 0 | 0 | 0 | 8.455 | 0 | 0.0848 | 0 | 0 | 0.0039 | 0.027 | 0 | 0 | 0 | 0.0039 | 0 | 0 | 0 |
| BC_adjacent | German_2023 | 0.5397 | 0.0002 | 0.0859 | 0 | 0 | 0 | 0.0078 | 0.0821 | 0.0051 | 0 | 0 | 0 | 0 | 0.1224 | 0.1595 | 0 | 0 | 0.0055 | 0 | 0 |
| BC_adjacent | German_2023 | 0.1989 | 0 | 0.6571 | 0 | 0.0908 | 0 | 3.385 | 0 | 3.6444 | 0 | 0 | 0 | 0 | 0 | 0 | 0.0022 | 0 | 0 | 0 | 0 |
| BC_adjacent | German_2023 | 0.0767 | 0.1057 | 0 | 0 | 0 | 0 | 0.122 | 0.0003 | 0.1047 | 0.0003 | 0 | 0 | 0 | 0 | 0 | 0 | 0.0003 | 0 | 0 | 0 |
| BC_adjacent | German_2023 | 0.4893 | 0.0013 | 0.0025 | 0 | 0 | 0 | 0.5822 | 0 | 0.008 | 0 | 0 | 0 | 0.1624 | 0.0423 | 0 | 0 | 0 | 0 | 0 | 0 |
| BC_adjacent | German_2023 | 2.3727 | 1.1503 | 0.7159 | 0 | 0 | 0 | 0.1182 | 0 | 0 | 0 | 0 | 0 | 0.0005 | 0.6575 | 0 | 0 | 0 | 0 | 0 | 0 |
| BC_adjacent | German_2023 | 0.7466 | 0 | 0.0542 | 0 | 0 | 0 | 0.1077 | 0.184 | 0.7292 | 0 | 0 | 0 | 0 | 0 | 0 | 0 | 0 | 0 | 0 | 0 |
| BC_tissue | German_2023 | 1.286 | 0 | 0.695 | 0 | 0 | 0 | 0.3943 | 0.0636 | 0 | 0 | 0 | 0 | 0.0003 | 0.0006 | 0 | 0 | 0 | 0 | 0 | 0 |
| BC_tissue | German_2023 | 2.3327 | 0.4486 | 0.6931 | 0 | 0 | 0.554 | 0.5428 | 0 | 5.215 | 0 | 0 | 0 | 12.5541 | 0 | 0 | 0 | 0.951 | 0 | 0 | 0 |
| BC_adjacent | German_2023 | 1.4638 | 0.0298 | 39.5866 | 0.0304 | 0.0013 | 0 | 0.1257 | 0 | 0 | 0 | 0 | 0.0026 | 0.3187 | 0.0032 | 0.1457 | 0 | 0.16 | 0 | 0 | 0 |
| BC_adjacent | German_2023 | 0.2378 | 0.1603 | 56.3343 | 0 | 0 | 0 | 0.004 | 0.0004 | 0 | 0 | 0 | 0 | 0 | 0 | 0 | 0 | 0 | 0 | 0 | 0 |
| BC_tissue | German_2023 | 1.0178 | 0 | 60.6139 | 0 | 0 | 0 | 0.034 | 0 | 0.0099 | 0 | 0 | 0 | 0 | 0.0018 | 0 | 0 | 0 | 0 | 0 | 0 |
| BC_tissue | German_2023 | 0.0654 | 0.0004 | 77.2513 | 0 | 0 | 0 | 0.8295 | 0 | 0.0011 | 0 | 0 | 0 | 0.0015 | 0 | 0 | 0 | 0.0008 | 0 | 0 | 0 |
| BC_adjacent | German_2023 | 0.1137 | 0.0381 | 0.0359 | 0 | 0 | 0 | 11.5233 | 0 | 0.0007 | 0 | 0 | 0 | 0 | 0.0007 | 0 | 0.0154 | 0.0161 | 0 | 0 | 0 |
| BC_tissue | German_2023 | 0.4093 | 0.0048 | 0.071 | 0 | 0.0026 | 0 | 0.0403 | 0 | 0.0026 | 0 | 0 | 0 | 0.0022 | 0.2098 | 0 | 0 | 0.3575 | 0 | 0 | 0 |
| BC_tissue | German_2023 | 0.6388 | 1.5092 | 0.0261 | 0 | 0 | 0 | 3.87 | 0 | 0 | 0 | 0 | 0 | 0.0075 | 0.0037 | 0.2988 | 0 | 0 | 0 | 0 | 0 |
| BC_adjacent | German_2023 | 2.7575 | 0.0793 | 0.001 | 0 | 0.0052 | 0 | 1.4792 | 0 | 0 | 0 | 0 | 0 | 0 | 0.0319 | 0 | 0 | 0 | 0 | 1.7491 | 0 |
| BC_tissue | German_2023 | 0.7209 | 0.0503 | 0.3521 | 0 | 0 | 0 | 9.7066 | 1.777 | 0.0335 | 0 | 0 | 0 | 0 | 0.0168 | 0 | 0 | 0 | 0 | 0 | 0 |
| BC_adjacent | German_2023 | 2.0746 | 0.2203 | 0.2263 | 0 | 0.2023 | 0 | 1.1434 | 0.0581 | 0.002 | 0 | 0 | 0.004 | 1.0313 | 0.0681 | 0 | 0.02 | 0 | 0 | 0 | 0 |
| BC_adjacent | German_2023 | 3.7831 | 0.1296 | 0.4339 | 0 | 1.0464 | 0 | 0.4712 | 0 | 0.0118 | 0 | 0 | 1.4626 | 0 | 0 | 0 | 0 | 0 | 0.6027 | 0 | 0 |
| BC_adjacent | German_2023 | 3.3934 | 0.0123 | 0.4072 | 0 | 0 | 0 | 2.3569 | 0 | 0.074 | 0 | 0 | 0 | 0 | 0 | 0 | 0 | 0 | 0 | 21.6436 | 0 |
| BC_adjacent | German_2023 | 0.3588 | 0.1082 | 75.6899 | 0 | 0.064 | 0 | 0.209 | 0.0389 | 0 | 0 | 0 | 0 | 0 | 0 | 0 | 0 | 0 | 0 | 0.0281 | 0 |
| BC_adjacent | German_2023 | 0.068 | 0.0262 | 78.4087 | 0 | 0 | 0.0328 | 0.2549 | 0 | 0 | 0 | 0 | 0 | 0 | 0 | 0 | 0 | 0 | 0 | 0.0004 | 0 |
| BC_adjacent | German_2023 | 2.2394 | 0.2488 | 25.3637 | 0 | 1.391 | 0 | 1.9512 | 0 | 0 | 0 | 0 | 0 | 0 | 0 | 0 | 0 | 1.0198 | 0 | 0 | 0 |
| BC_adjacent | German_2023 | 0.1892 | 0.0025 | 0.0025 | 0 | 0 | 0 | 7.4757 | 0 | 4.8495 | 0 | 0 | 0.0098 | 0.0123 | 0 | 0 | 0.0393 | 0 | 0 | 0 | 0 |
| BC_adjacent | German_2023 | 1.6288 | 0.0024 | 0.3046 | 0 | 0.0012 | 0 | 3.0951 | 0.0012 | 0.0024 | 0 | 0 | 0 | 0 | 0 | 0 | 0 | 0 | 0 | 0 | 0 |
| BC_adjacent | German_2023 | 0.0033 | 0.0022 | 0.2662 | 0 | 0 | 0 | 9.2845 | 0 | 0.0011 | 0 | 0 | 0 | 0 | 0 | 0 | 0 | 0 | 0 | 0 | 0 |
| BC_adjacent | German_2023 | 0.0052 | 0 | 76.6863 | 0.0017 | 0.0006 | 0 | 1.0462 | 0 | 0.0006 | 0 | 0 | 0 | 0 | 0 | 0 | 0.0035 | 0 | 0 | 0 | 0 |
| BC_adjacent | German_2023 | 2.2402 | 0.0283 | 0.3102 | 0 | 0 | 0 | 0.295 | 0 | 0.0011 | 0 | 0 | 0 | 0 | 0 | 0 | 0.0152 | 0 | 0 | 0 | 0 |
| BC_tissue | German_2023 | 10.1031 | 0.1057 | 0.0132 | 0 | 0 | 0 | 1.9122 | 0 | 0.0264 | 0 | 0 | 0 | 0.8283 | 0.3393 | 0.4979 | 0 | 0.3525 | 0 | 0 | 0 |
| BC_adjacent | German_2023 | 0.0164 | 0.001 | 64.9046 | 0 | 0 | 0 | 0.4707 | 0 | 0.001 | 0 | 0 | 6.065 | 0 | 0.001 | 0 | 0 | 0 | 0 | 0 | 0 |
| BC_adjacent | German_2023 | 0.7157 | 0.185 | 0 | 0 | 0 | 0 | 0.0131 | 0.0002 | 0.0675 | 0 | 0 | 0 | 0 | 0.0097 | 1.199 | 0.035 | 0 | 0 | 0 | 0 |
| BC_tissue | German_2023 | 0.8941 | 0.0565 | 0.1352 | 0 | 0 | 0 | 0.5685 | 0.0853 | 0.0289 | 0 | 0 | 0 | 0.2035 | 2.1284 | 2.4645 | 0.0013 | 0.0026 | 0 | 0 | 0 |
| BC_adjacent | German_2023 | 0.059 | 0 | 0.0312 | 0.0139 | 0 | 0 | 30.2688 | 0.1838 | 1.4912 | 0 | 0 | 0 | 0.0694 | 0 | 0 | 0 | 0 | 0 | 0 | 0 |
| BC_tissue | German_2023 | 2.0973 | 0.0018 | 0.4661 | 0 | 0 | 0 | 4.2973 | 0.8422 | 0.0018 | 0 | 0 | 0 | 0.0073 | 0 | 0 | 0 | 0.7633 | 0 | 0 | 0 |
| BC_adjacent | German_2023 | 0.4487 | 0.0832 | 0.4158 | 0 | 0.0007 | 0 | 0.5821 | 0.0142 | 0.0037 | 0 | 0 | 2.8378 | 0.0052 | 0.0007 | 0 | 0 | 0.0007 | 0 | 0 | 0 |

**Table S4B. The RF scores at the genus level in both BC_tissue and BC_adjacent samples.**

| **Group** | **Cohort** | **RF score** |
| --- | --- | --- |
| BC_tissue | Hoskinson_2022 | 0.3 |
| BC_adjacent | Hoskinson_2022 | 0.86 |
| BC_adjacent | Hoskinson_2022 | 0.67 |
| BC_tissue | Hoskinson_2022 | 0.25 |
| BC_adjacent | Hoskinson_2022 | 0.84 |
| BC_adjacent | Hoskinson_2022 | 0.73 |
| BC_adjacent | Hoskinson_2022 | 0.87 |
| BC_tissue | Hoskinson_2022 | 0.14 |
| BC_tissue | Hoskinson_2022 | 0.19 |
| BC_adjacent | Hoskinson_2022 | 0.84 |
| BC_tissue | Hoskinson_2022 | 0.3 |
| BC_adjacent | Hoskinson_2022 | 0.75 |
| BC_tissue | Hoskinson_2022 | 0.23 |
| BC_adjacent | Hoskinson_2022 | 0.9 |
| BC_tissue | Hoskinson_2022 | 0.11 |
| BC_adjacent | Hoskinson_2022 | 0.88 |
| BC_tissue | Hoskinson_2022 | 0.1 |
| BC_adjacent | Hoskinson_2022 | 0.72 |
| BC_adjacent | Hoskinson_2022 | 0.81 |
| BC_adjacent | Hoskinson_2022 | 0.87 |
| BC_tissue | Hoskinson_2022 | 0.16 |
| BC_adjacent | Hoskinson_2022 | 0.83 |
| BC_adjacent | Hoskinson_2022 | 0.81 |
| BC_tissue | Hoskinson_2022 | 0.19 |
| BC_adjacent | Hoskinson_2022 | 0.78 |
| BC_tissue | Hoskinson_2022 | 0.15 |
| BC_tissue | Hoskinson_2022 | 0.13 |
| BC_tissue | Hoskinson_2022 | 0.16 |
| BC_adjacent | Hoskinson_2022 | 0.84 |
| BC_adjacent | Hoskinson_2022 | 0.71 |
| BC_tissue | Hoskinson_2022 | 0.23 |
| BC_tissue | Hoskinson_2022 | 0.22 |
| BC_tissue | Hoskinson_2022 | 0.26 |
| BC_tissue | Hoskinson_2022 | 0.23 |
| BC_adjacent | Hoskinson_2022 | 0.88 |
| BC_tissue | Hoskinson_2022 | 0.1 |
| BC_adjacent | Hoskinson_2022 | 0.87 |
| BC_tissue | Hoskinson_2022 | 0.16 |
| BC_tissue | Hoskinson_2022 | 0.11 |
| BC_adjacent | Hoskinson_2022 | 0.75 |
| BC_adjacent | Hoskinson_2022 | 0.78 |
| BC_tissue | Hoskinson_2022 | 0.14 |
| BC_tissue | Hoskinson_2022 | 0.18 |
| BC_adjacent | Hoskinson_2022 | 0.88 |
| BC_tissue | Hoskinson_2022 | 0.18 |
| BC_adjacent | Hoskinson_2022 | 0.82 |
| BC_tissue | Hoskinson_2022 | 0.19 |
| BC_tissue | Hoskinson_2022 | 0.12 |
| BC_tissue | Hoskinson_2022 | 0.29 |
| BC_tissue | Hoskinson_2022 | 0.1 |
| BC_adjacent | Hoskinson_2022 | 0.77 |
| BC_tissue | Hoskinson_2022 | 0.18 |
| BC_tissue | Hoskinson_2022 | 0.15 |
| BC_tissue | Hoskinson_2022 | 0.21 |
| BC_adjacent | Hoskinson_2022 | 0.72 |
| BC_tissue | Hoskinson_2022 | 0.17 |
| BC_adjacent | Hoskinson_2022 | 0.76 |
| BC_tissue | Hoskinson_2022 | 0.13 |
| BC_adjacent | Hoskinson_2022 | 0.89 |
| BC_adjacent | Hoskinson_2022 | 0.86 |
| BC_adjacent | Hoskinson_2022 | 0.82 |
| BC_adjacent | Hoskinson_2022 | 0.86 |
| BC_adjacent | Hoskinson_2022 | 0.75 |
| BC_tissue | Hoskinson_2022 | 0.2 |
| BC_adjacent | Hoskinson_2022 | 0.74 |
| BC_tissue | Hoskinson_2022 | 0.11 |
| BC_tissue | Hoskinson_2022 | 0.25 |
| BC_tissue | Hoskinson_2022 | 0.12 |
| BC_tissue | Hoskinson_2022 | 0.34 |
| BC_adjacent | Hoskinson_2022 | 0.75 |
| BC_adjacent | Hoskinson_2022 | 0.86 |
| BC_adjacent | Hoskinson_2022 | 0.83 |
| BC_tissue | Hoskinson_2022 | 0.2 |
| BC_tissue | Hoskinson_2022 | 0.22 |
| BC_adjacent | Hoskinson_2022 | 0.86 |
| BC_adjacent | Hoskinson_2022 | 0.85 |
| BC_tissue | Hoskinson_2022 | 0.13 |
| BC_adjacent | Hoskinson_2022 | 0.85 |
| BC_tissue | Hoskinson_2022 | 0.31 |
| BC_adjacent | Hoskinson_2022 | 0.87 |
| BC_adjacent | Hoskinson_2022 | 0.88 |
| BC_tissue | Hoskinson_2022 | 0.3 |
| BC_tissue | Hoskinson_2022 | 0.19 |
| BC_adjacent | Hoskinson_2022 | 0.84 |
| BC_adjacent | Hoskinson_2022 | 0.84 |
| BC_adjacent | Hoskinson_2022 | 0.72 |
| BC_adjacent | Hoskinson_2022 | 0.81 |
| BC_adjacent | Hoskinson_2022 | 0.78 |
| BC_adjacent | Hoskinson_2022 | 0.89 |
| BC_tissue | Hoskinson_2022 | 0.09 |
| BC_tissue | Hoskinson_2022 | 0.21 |
| BC_adjacent | Hoskinson_2022 | 0.69 |
| BC_adjacent | Hoskinson_2022 | 0.88 |
| BC_tissue | Hoskinson_2022 | 0.23 |
| BC_adjacent | Hoskinson_2022 | 0.79 |
| BC_tissue | Hoskinson_2022 | 0.12 |
| BC_adjacent | Esposito_2022 | 0.62 |
| BC_tissue | Esposito_2022 | 0.04 |
| BC_tissue | Esposito_2022 | 0.12 |
| BC_adjacent | Esposito_2022 | 0.92 |
| BC_tissue | Esposito_2022 | 0.19 |
| BC_adjacent | Esposito_2022 | 0.79 |
| BC_tissue | Esposito_2022 | 0.11 |
| BC_adjacent | Esposito_2022 | 0.99 |
| BC_adjacent | Esposito_2022 | 0.96 |
| BC_tissue | Esposito_2022 | 0.13 |
| BC_adjacent | Esposito_2022 | 0.73 |
| BC_tissue | Esposito_2022 | 0.22 |
| BC_adjacent | Esposito_2022 | 0.71 |
| BC_tissue | Esposito_2022 | 0.13 |
| BC_adjacent | Esposito_2022 | 0.91 |
| BC_tissue | Esposito_2022 | 0.1 |
| BC_tissue | Esposito_2022 | 0.16 |
| BC_adjacent | Esposito_2022 | 0.97 |
| BC_tissue | Esposito_2022 | 0.23 |
| BC_adjacent | Esposito_2022 | 0.95 |
| BC_tissue | Esposito_2022 | 0.06 |
| BC_adjacent | Esposito_2022 | 1 |
| BC_tissue | Esposito_2022 | 0.03 |
| BC_adjacent | Esposito_2022 | 0.98 |
| BC_tissue | Esposito_2022 | 0.03 |
| BC_adjacent | Esposito_2022 | 0.91 |
| BC_adjacent | Esposito_2022 | 0.65 |
| BC_tissue | Esposito_2022 | 0.24 |
| BC_adjacent | Esposito_2022 | 0.94 |
| BC_tissue | Esposito_2022 | 0.1 |
| BC_adjacent | Esposito_2022 | 0.98 |
| BC_tissue | Esposito_2022 | 0.28 |
| BC_adjacent | Esposito_2022 | 0.93 |
| BC_tissue | Esposito_2022 | 0.13 |
| BC_adjacent | Esposito_2022 | 0.85 |
| BC_tissue | Esposito_2022 | 0.21 |
| BC_tissue | Esposito_2022 | 0.04 |
| BC_adjacent | Esposito_2022 | 0.86 |
| BC_adjacent | Esposito_2022 | 0.99 |
| BC_tissue | Esposito_2022 | 0.17 |
| BC_adjacent | Esposito_2022 | 0.97 |
| BC_tissue | Esposito_2022 | 0.12 |
| BC_adjacent | Esposito_2022 | 0.99 |
| BC_tissue | Esposito_2022 | 0.04 |
| BC_adjacent | Esposito_2022 | 0.8 |
| BC_tissue | Esposito_2022 | 0.04 |
| BC_adjacent | Esposito_2022 | 0.77 |
| BC_tissue | Esposito_2022 | 0.03 |
| BC_adjacent | Esposito_2022 | 0.77 |
| BC_tissue | Esposito_2022 | 0.14 |
| BC_tissue | Esposito_2022 | 0.04 |
| BC_adjacent | Esposito_2022 | 0.95 |
| BC_tissue | Esposito_2022 | 0.07 |
| BC_adjacent | Esposito_2022 | 0.99 |
| BC_tissue | Esposito_2022 | 0.11 |
| BC_adjacent | Esposito_2022 | 0.98 |
| BC_tissue | Esposito_2022 | 0.12 |
| BC_adjacent | Esposito_2022 | 0.89 |
| BC_tissue | Esposito_2022 | 0.07 |
| BC_adjacent | Esposito_2022 | 0.99 |
| BC_adjacent | Esposito_2022 | 0.98 |
| BC_tissue | Esposito_2022 | 0.13 |
| BC_adjacent | Esposito_2022 | 0.95 |
| BC_tissue | Esposito_2022 | 0.12 |
| BC_adjacent | Esposito_2022 | 0.83 |
| BC_tissue | Esposito_2022 | 0.16 |
| BC_adjacent | Esposito_2022 | 1 |
| BC_adjacent | Kartti_2023 | 0.95 |
| BC_adjacent | Kartti_2023 | 0.9 |
| BC_adjacent | Kartti_2023 | 0.93 |
| BC_tissue | Kartti_2023 | 0.14 |
| BC_adjacent | Kartti_2023 | 0.9 |
| BC_adjacent | Kartti_2023 | 0.85 |
| BC_adjacent | Kartti_2023 | 0.94 |
| BC_adjacent | Kartti_2023 | 0.91 |
| BC_adjacent | Kartti_2023 | 0.83 |
| BC_adjacent | Kartti_2023 | 0.84 |
| BC_adjacent | Kartti_2023 | 0.89 |
| BC_adjacent | Kartti_2023 | 0.92 |
| BC_adjacent | Kartti_2023 | 0.79 |
| BC_adjacent | Kartti_2023 | 0.76 |
| BC_tissue | Kartti_2023 | 0.08 |
| BC_adjacent | Kartti_2023 | 0.86 |
| BC_adjacent | Kartti_2023 | 0.84 |
| BC_adjacent | Kartti_2023 | 0.85 |
| BC_adjacent | Kartti_2023 | 0.88 |
| BC_adjacent | Kartti_2023 | 0.96 |
| BC_adjacent | Kartti_2023 | 0.85 |
| BC_adjacent | Kartti_2023 | 0.78 |
| BC_adjacent | Kartti_2023 | 0.71 |
| BC_adjacent | Kartti_2023 | 0.72 |
| BC_adjacent | Kartti_2023 | 0.87 |
| BC_tissue | Kartti_2023 | 0.15 |
| BC_adjacent | Kartti_2023 | 0.75 |
| BC_adjacent | Kartti_2023 | 0.83 |
| BC_adjacent | Kartti_2023 | 0.84 |
| BC_adjacent | Kartti_2023 | 0.85 |
| BC_adjacent | Kartti_2023 | 0.78 |
| BC_adjacent | Kartti_2023 | 0.83 |
| BC_adjacent | Kartti_2023 | 0.85 |
| BC_adjacent | Kartti_2023 | 0.84 |
| BC_adjacent | Kartti_2023 | 0.89 |
| BC_adjacent | Kartti_2023 | 0.78 |
| BC_adjacent | Kartti_2023 | 0.8 |
| BC_adjacent | Kartti_2023 | 0.86 |
| BC_adjacent | Kartti_2023 | 0.84 |
| BC_adjacent | Kartti_2023 | 0.82 |
| BC_adjacent | Kartti_2023 | 0.84 |
| BC_adjacent | Kartti_2023 | 0.89 |
| BC_adjacent | Kartti_2023 | 0.85 |
| BC_tissue | Kartti_2023 | 0.21 |
| BC_adjacent | Kartti_2023 | 0.84 |
| BC_adjacent | Kartti_2023 | 0.76 |
| BC_adjacent | Kartti_2023 | 0.88 |
| BC_adjacent | Kartti_2023 | 0.68 |
| BC_adjacent | Kartti_2023 | 0.89 |
| BC_adjacent | Kartti_2023 | 0.88 |
| BC_adjacent | Kartti_2023 | 0.84 |
| BC_adjacent | Kartti_2023 | 0.85 |
| BC_adjacent | Kartti_2023 | 0.9 |
| BC_tissue | Kartti_2023 | 0.18 |
| BC_tissue | Kartti_2023 | 0.13 |
| BC_tissue | Kartti_2023 | 0.1 |
| BC_tissue | Kartti_2023 | 0.11 |
| BC_tissue | Kartti_2023 | 0.21 |
| BC_tissue | Kartti_2023 | 0.15 |
| BC_tissue | Kartti_2023 | 0.2 |
| BC_tissue | Kartti_2023 | 0.17 |
| BC_tissue | Kartti_2023 | 0.21 |
| BC_tissue | Kartti_2023 | 0.16 |
| BC_tissue | Kartti_2023 | 0.35 |
| BC_tissue | Kartti_2023 | 0.17 |
| BC_tissue | Kartti_2023 | 0.18 |
| BC_tissue | Kartti_2023 | 0.18 |
| BC_tissue | Kartti_2023 | 0.12 |
| BC_tissue | Kartti_2023 | 0.12 |
| BC_tissue | Kartti_2023 | 0.16 |
| BC_tissue | Kartti_2023 | 0.11 |
| BC_tissue | Kartti_2023 | 0.18 |
| BC_tissue | Kartti_2023 | 0.21 |
| BC_tissue | Kartti_2023 | 0.19 |
| BC_tissue | Kartti_2023 | 0.2 |
| BC_tissue | Kartti_2023 | 0.13 |
| BC_tissue | Kartti_2023 | 0.07 |
| BC_tissue | Kartti_2023 | 0.14 |
| BC_tissue | Kartti_2023 | 0.23 |
| BC_tissue | Kartti_2023 | 0.22 |
| BC_tissue | Kartti_2023 | 0.12 |
| BC_tissue | Kartti_2023 | 0.11 |
| BC_tissue | Kartti_2023 | 0.14 |
| BC_tissue | Kartti_2023 | 0.2 |
| BC_tissue | Kartti_2023 | 0.26 |
| BC_tissue | Kartti_2023 | 0.16 |
| BC_tissue | Kartti_2023 | 0.16 |
| BC_tissue | Kartti_2023 | 0.15 |
| BC_tissue | Kartti_2023 | 0.08 |
| BC_tissue | Kartti_2023 | 0.2 |
| BC_tissue | Kartti_2023 | 0.12 |
| BC_tissue | Kartti_2023 | 0.32 |
| BC_tissue | Kartti_2023 | 0.14 |
| BC_tissue | Kartti_2023 | 0.22 |
| BC_tissue | Kartti_2023 | 0.06 |
| BC_tissue | Kartti_2023 | 0.11 |
| BC_tissue | Kartti_2023 | 0.19 |
| BC_adjacent | Kartti_2023 | 0.85 |
| BC_adjacent | Kartti_2023 | 0.75 |
| BC_adjacent | Kartti_2023 | 0.86 |
| BC_tissue | Kartti_2023 | 0.08 |
| BC_tissue | Kartti_2023 | 0.26 |
| BC_tissue | Kartti_2023 | 0.11 |
| BC_adjacent | German_2023 | 0.84 |
| BC_adjacent | German_2023 | 0.91 |
| BC_adjacent | German_2023 | 0.86 |
| BC_tissue | German_2023 | 0.3 |
| BC_tissue | German_2023 | 0.15 |
| BC_adjacent | German_2023 | 0.95 |
| BC_adjacent | German_2023 | 0.86 |
| BC_adjacent | German_2023 | 0.82 |
| BC_tissue | German_2023 | 0.23 |
| BC_adjacent | German_2023 | 0.94 |
| BC_adjacent | German_2023 | 0.83 |
| BC_tissue | German_2023 | 0.11 |
| BC_adjacent | German_2023 | 0.94 |
| BC_tissue | German_2023 | 0.29 |
| BC_adjacent | German_2023 | 0.88 |
| BC_tissue | German_2023 | 0.18 |
| BC_adjacent | German_2023 | 0.94 |
| BC_tissue | German_2023 | 0.34 |
| BC_adjacent | German_2023 | 0.92 |
| BC_tissue | German_2023 | 0.16 |
| BC_adjacent | German_2023 | 0.81 |
| BC_tissue | German_2023 | 0.13 |
| BC_tissue | German_2023 | 0.19 |
| BC_adjacent | German_2023 | 0.9 |
| BC_tissue | German_2023 | 0.28 |
| BC_adjacent | German_2023 | 0.94 |
| BC_tissue | German_2023 | 0.19 |
| BC_adjacent | German_2023 | 0.87 |
| BC_adjacent | German_2023 | 0.76 |
| BC_adjacent | German_2023 | 0.98 |
| BC_adjacent | German_2023 | 0.82 |
| BC_adjacent | German_2023 | 0.86 |
| BC_tissue | German_2023 | 0.1 |
| BC_adjacent | German_2023 | 0.83 |
| BC_adjacent | German_2023 | 0.9 |
| BC_adjacent | German_2023 | 0.82 |
| BC_tissue | German_2023 | 0.17 |
| BC_adjacent | German_2023 | 0.84 |
| BC_tissue | German_2023 | 0.27 |
| BC_tissue | German_2023 | 0.2 |
| BC_adjacent | German_2023 | 0.81 |
| BC_tissue | German_2023 | 0.09 |
| BC_adjacent | German_2023 | 0.9 |
| BC_tissue | German_2023 | 0.25 |
| BC_adjacent | German_2023 | 0.85 |
| BC_adjacent | German_2023 | 0.73 |
| BC_tissue | German_2023 | 0.16 |
| BC_adjacent | German_2023 | 0.86 |
| BC_adjacent | German_2023 | 0.87 |
| BC_adjacent | German_2023 | 0.88 |
| BC_tissue | German_2023 | 0.19 |
| BC_adjacent | German_2023 | 0.88 |
| BC_adjacent | German_2023 | 0.84 |
| BC_adjacent | German_2023 | 0.71 |
| BC_adjacent | German_2023 | 0.79 |
| BC_adjacent | German_2023 | 0.89 |
| BC_adjacent | German_2023 | 0.82 |
| BC_adjacent | German_2023 | 0.79 |
| BC_adjacent | German_2023 | 0.93 |
| BC_adjacent | German_2023 | 0.91 |
| BC_adjacent | German_2023 | 0.79 |
| BC_tissue | German_2023 | 0.29 |
| BC_tissue | German_2023 | 0.16 |
| BC_adjacent | German_2023 | 0.84 |
| BC_adjacent | German_2023 | 0.98 |
| BC_tissue | German_2023 | 0.22 |
| BC_tissue | German_2023 | 0.31 |
| BC_adjacent | German_2023 | 0.86 |
| BC_tissue | German_2023 | 0.17 |
| BC_tissue | German_2023 | 0.21 |
| BC_adjacent | German_2023 | 0.88 |
| BC_tissue | German_2023 | 0.19 |
| BC_adjacent | German_2023 | 0.88 |
| BC_adjacent | German_2023 | 0.91 |
| BC_adjacent | German_2023 | 0.84 |
| BC_adjacent | German_2023 | 0.93 |
| BC_adjacent | German_2023 | 0.83 |
| BC_adjacent | German_2023 | 0.82 |
| BC_adjacent | German_2023 | 0.86 |
| BC_adjacent | German_2023 | 0.92 |
| BC_adjacent | German_2023 | 0.89 |
| BC_adjacent | German_2023 | 0.88 |
| BC_adjacent | German_2023 | 0.95 |
| BC_tissue | German_2023 | 0.17 |
| BC_adjacent | German_2023 | 0.88 |
| BC_adjacent | German_2023 | 0.83 |
| BC_tissue | German_2023 | 0.28 |
| BC_adjacent | German_2023 | 0.79 |
| BC_tissue | German_2023 | 0.13 |
| BC_adjacent | German_2023 | 0.94 |
