# Supplementary material for: Microbial diagnostic features identified across populations possess potential antitumor properties in breast cancer
Source: mSystems. 2025 Jun 23;10(7):e00271-25. doi: 10.1128/msystems.00271-25 (PMC12282184; doi:10.1128/msystems.00271-25)
Supplement: Table S5 — The abundance of the 14 specific genera in both BC_tissue and normal_tissue samples. [file msystems.00271-25-s0005.doc]

**Table S5A. The abundance of the 14 specific genera in both BC_tissue and normal_tissue samples.**

| **Group** | **Cohort** | ***Cutibacterium*** | ***Acinetobacter*** | ***Burkholderia*** | ***Prevotella*** | ***Pseudomonas_E_647464*** | ***Corynebacterium*** | ***Streptococcus*** | ***Ralstonia*** | ***Lactobacillus*** | ***GWA2-37-10*** | ***Anaerococcus*** | ***Escherichia_710834*** | ***Duganella_571129*** | ***Finegoldia*** |
| --- | --- | --- | --- | --- | --- | --- | --- | --- | --- | --- | --- | --- | --- | --- | --- |
| BC_tissue | Hoskinson_2022 | 0.3511 | 0 | 0 | 0 | 0 | 2.1572 | 2.8593 | 0.0211 | 0.0804 | 0.1607 | 0.0338 | 0.1734 | 0 | 0 |
| BC_tissue | Hoskinson_2022 | 0.0017 | 0.0017 | 0 | 0.0017 | 0.0017 | 0.0348 | 1.1951 | 0 | 0 | 0 | 0.1725 | 0.0035 | 0 | 0 |
| BC_tissue | Hoskinson_2022 | 0.4019 | 0.0014 | 0 | 0.0014 | 0 | 14.6559 | 0.0043 | 0 | 0 | 0.0029 | 7.5595 | 2.6353 | 0 | 0 |
| BC_tissue | Hoskinson_2022 | 0.6603 | 0 | 0 | 0 | 0.0082 | 6.836 | 0.0192 | 0 | 0.0548 | 0.0192 | 0.6658 | 1.7124 | 0 | 0 |
| BC_tissue | Hoskinson_2022 | 1.0333 | 0 | 0 | 0 | 0 | 11.0039 | 0.0064 | 0 | 0.015 | 0.0021 | 0.0043 | 0 | 0 | 0 |
| BC_tissue | Hoskinson_2022 | 1.2246 | 0.3238 | 0 | 0 | 0 | 0.4233 | 0 | 0 | 0.0151 | 0.003 | 0 | 0 | 0 | 0 |
| BC_tissue | Hoskinson_2022 | 2.2896 | 0 | 0 | 0 | 0.0014 | 0 | 0 | 0 | 7.4082 | 0 | 13.1567 | 0 | 0 | 0 |
| BC_tissue | Hoskinson_2022 | 0.6074 | 0.0011 | 0 | 0 | 0 | 0 | 3.8609 | 0 | 0.0011 | 0.0033 | 0 | 0 | 0.0022 | 0.0022 |
| BC_tissue | Hoskinson_2022 | 0.3146 | 0.048 | 0 | 0 | 1.4427 | 0.2955 | 11.6801 | 0 | 10.2699 | 0 | 0 | 0 | 0.4163 | 0 |
| BC_tissue | Hoskinson_2022 | 0.4978 | 0 | 0 | 0 | 11.4572 | 3.8794 | 0 | 0.0025 | 0.0075 | 0.005 | 0 | 0.0025 | 0.0025 | 0 |
| BC_tissue | Hoskinson_2022 | 3.1187 | 0 | 0 | 0 | 0.527 | 0.0015 | 14.4345 | 0 | 0.0015 | 0.0015 | 0 | 0 | 12.1828 | 0 |
| BC_tissue | Hoskinson_2022 | 4.0837 | 0 | 0 | 0 | 0.0017 | 0 | 0.0051 | 0 | 0 | 0 | 0.0017 | 0 | 0 | 0 |
| BC_tissue | Hoskinson_2022 | 0.0104 | 0 | 0 | 0 | 0 | 1.1171 | 0 | 0 | 0 | 0 | 0.0017 | 0 | 0 | 0.0017 |
| BC_tissue | Hoskinson_2022 | 0.0276 | 0 | 0 | 4.5966 | 0.0263 | 1.7214 | 0 | 0 | 0 | 0 | 22.9317 | 0 | 0 | 12.5322 |
| BC_tissue | Hoskinson_2022 | 1.134 | 0 | 0 | 0.0016 | 0.2297 | 0.0048 | 0.0095 | 0 | 0.0016 | 0 | 0 | 0 | 0.0016 | 0 |
| BC_tissue | Hoskinson_2022 | 1.3141 | 0.002 | 0 | 0 | 28.2799 | 6.6236 | 0.0098 | 0 | 12.6029 | 0 | 0 | 0.0039 | 0.0039 | 0 |
| BC_tissue | Hoskinson_2022 | 0.0836 | 0.0139 | 0 | 0 | 0 | 4.3845 | 0.0119 | 0 | 0.0199 | 0 | 0.0159 | 0.1852 | 0.002 | 0.002 |
| BC_tissue | Hoskinson_2022 | 0.0078 | 0.0033 | 0 | 0 | 4.5332 | 0.2499 | 22.8477 | 0 | 0 | 0 | 0 | 0.0089 | 0 | 0 |
| BC_tissue | Hoskinson_2022 | 0.0675 | 0.0051 | 0 | 0 | 0 | 0.0038 | 41.0181 | 0 | 0.0064 | 0 | 0.0013 | 0.037 | 0 | 0.0013 |
| BC_tissue | Hoskinson_2022 | 0 | 0.0255 | 0 | 0.0396 | 0 | 0.9379 | 0.0013 | 0 | 0 | 0 | 3.6867 | 0 | 0 | 1.3195 |
| BC_tissue | Hoskinson_2022 | 0 | 0.017 | 0 | 0.168 | 0.0012 | 1.0507 | 0.0037 | 0 | 0 | 0 | 4.4221 | 0 | 0 | 1.3381 |
| BC_tissue | Hoskinson_2022 | 0.0772 | 0.018 | 0 | 0 | 0.0964 | 0.0026 | 0.0013 | 0 | 0.0013 | 0 | 0 | 0.0617 | 0 | 0 |
| BC_tissue | Hoskinson_2022 | 0.0685 | 0.0011 | 0 | 0 | 0 | 1.9333 | 0.0011 | 0 | 0.0033 | 0.0011 | 0.0011 | 0.0276 | 0 | 0 |
| BC_tissue | Hoskinson_2022 | 0.1121 | 0.0046 | 0 | 0.0046 | 0 | 16.3904 | 10.5006 | 0 | 0 | 0 | 0 | 0.0289 | 0 | 0 |
| BC_tissue | Hoskinson_2022 | 0.1576 | 0.0653 | 0 | 0.0175 | 0 | 7.6242 | 0 | 0 | 0.0064 | 0 | 0.0191 | 0.1449 | 0.0032 | 0.0096 |
| BC_tissue | Hoskinson_2022 | 0.03 | 0 | 0 | 0 | 0.7941 | 0.003 | 0.863 | 0 | 0.003 | 0 | 0.0015 | 0.012 | 0 | 0 |
| BC_tissue | Hoskinson_2022 | 0.1623 | 0.005 | 0 | 0 | 0 | 0.0012 | 0 | 0.0025 | 23.0225 | 0.0012 | 0 | 0.0624 | 0 | 0 |
| BC_tissue | Hoskinson_2022 | 0.0749 | 0.0029 | 0 | 0 | 0.001 | 0.3075 | 5.06 | 0 | 0.001 | 0 | 0.0029 | 0.0214 | 0 | 0 |
| BC_tissue | Hoskinson_2022 | 0.2343 | 0.0205 | 0 | 0 | 0.1153 | 0.0013 | 0 | 0 | 0.0154 | 0 | 0.0102 | 0.1703 | 0 | 0 |
| BC_tissue | Hoskinson_2022 | 0.0161 | 0 | 0 | 0 | 0.0009 | 3.991 | 0 | 0 | 0 | 0 | 0 | 0.0019 | 0 | 0 |
| BC_tissue | Hoskinson_2022 | 0.1587 | 0.0517 | 0 | 0 | 11.8751 | 0.107 | 0.0036 | 0 | 0.1123 | 0 | 0.0178 | 0.2228 | 0 | 0 |
| BC_tissue | Hoskinson_2022 | 0.2317 | 0.0116 | 0 | 0 | 0 | 0.0023 | 0 | 0 | 0.0139 | 0 | 0 | 0.2201 | 0.0046 | 0 |
| BC_tissue | Hoskinson_2022 | 0.0036 | 0.0036 | 0 | 0.0018 | 0.0036 | 3.0777 | 0 | 0.0054 | 0.0234 | 0.0036 | 0 | 0.1635 | 0.0036 | 0.0054 |
| BC_tissue | Hoskinson_2022 | 0.0054 | 0 | 0 | 29.7232 | 0 | 0 | 3.3689 | 0 | 0 | 0 | 0 | 0.0253 | 0 | 0 |
| BC_tissue | Hoskinson_2022 | 0.2788 | 0.0047 | 0 | 0 | 9.3366 | 0.0024 | 0.0024 | 0 | 0.0378 | 0.0071 | 0 | 0.0425 | 0 | 0 |
| BC_tissue | Hoskinson_2022 | 0.1035 | 0.0107 | 0 | 0.0018 | 0.0071 | 0 | 0.0089 | 0 | 0 | 0.0214 | 0 | 0.0232 | 0.0036 | 0 |
| BC_tissue | Hoskinson_2022 | 0.0597 | 0.0043 | 0 | 0 | 0 | 1.1371 | 0.0064 | 0.0021 | 0 | 0 | 0 | 0 | 0 | 0 |
| BC_tissue | Hoskinson_2022 | 0.0071 | 0 | 0 | 0 | 0.4867 | 0 | 0 | 0 | 0.0036 | 0 | 0 | 0.0107 | 0 | 0 |
| BC_tissue | Hoskinson_2022 | 0.1233 | 0.0044 | 0 | 0 | 0.0088 | 0.1299 | 28.8916 | 0 | 0 | 0.022 | 0 | 0.0727 | 0.0022 | 0 |
| BC_tissue | Hoskinson_2022 | 0 | 0.0047 | 0 | 0.0016 | 92.62 | 0.5748 | 0.4264 | 0 | 0 | 0 | 0 | 0.0031 | 0.0016 | 0 |
| BC_tissue | Hoskinson_2022 | 1.2746 | 0 | 0 | 0 | 0 | 5.207 | 0 | 0 | 0 | 0 | 0 | 0.227 | 0 | 0 |
| normal_tissue | Hoskinson_2022 | 0.1 | 3.4115 | 0 | 10.7935 | 0 | 0.0039 | 0.0039 | 0.0039 | 0 | 0.0432 | 0 | 0.0235 | 0 | 0 |
| normal_tissue | Hoskinson_2022 | 1.9958 | 0 | 0 | 0 | 0 | 7.4751 | 0.0043 | 0 | 0 | 0 | 0 | 0.0071 | 0.0014 | 0 |
| normal_tissue | Hoskinson_2022 | 0.0092 | 0.0055 | 0 | 18.3986 | 0.0018 | 0.0148 | 0 | 0 | 0.0129 | 0 | 0.0037 | 1.735 | 0.0037 | 0.0166 |
| normal_tissue | Hoskinson_2022 | 2.4097 | 0.002 | 0 | 0 | 3.1447 | 13.1483 | 0.0058 | 0 | 0.0058 | 0.0039 | 0 | 0.0039 | 0.0078 | 0 |
| normal_tissue | Hoskinson_2022 | 0 | 5.5255 | 0 | 0 | 32.0542 | 0.0193 | 0 | 0 | 0.0338 | 0 | 0.0024 | 0 | 0 | 0 |
| normal_tissue | Hoskinson_2022 | 0.5129 | 3.9133 | 0 | 0 | 10.5141 | 4.1071 | 0 | 0 | 0.004 | 0 | 0 | 0 | 0 | 0 |
| normal_tissue | Hoskinson_2022 | 0.9027 | 5.3633 | 0 | 0 | 34.7885 | 0.9293 | 0.0022 | 0 | 0.0044 | 0 | 0 | 0 | 0 | 0 |
| normal_tissue | Hoskinson_2022 | 1.7401 | 0 | 0 | 0 | 0.0015 | 0.0059 | 0.0015 | 0 | 5.3878 | 0.003 | 0 | 0 | 0 | 0 |
| normal_tissue | Hoskinson_2022 | 0.0088 | 4.7794 | 0 | 0.0058 | 34.5289 | 1.7627 | 0.8653 | 0 | 0.0029 | 0 | 0 | 0 | 0 | 0 |
| normal_tissue | Hoskinson_2022 | 0.3535 | 0.002 | 0 | 8.1934 | 1.8504 | 15.7001 | 0 | 0 | 0.0162 | 0.004 | 0 | 0 | 0 | 0 |
| normal_tissue | Hoskinson_2022 | 1.8791 | 0 | 0 | 0.0057 | 1.9001 | 0.5666 | 4.9487 | 0 | 0.0248 | 0.0076 | 0.0038 | 0 | 0.0019 | 0 |
| normal_tissue | Hoskinson_2022 | 1.2534 | 14.6345 | 0 | 0 | 0.0037 | 0.0056 | 0 | 0 | 0.0131 | 0 | 0 | 0 | 0 | 0 |
| normal_tissue | Hoskinson_2022 | 1.3219 | 13.6906 | 0 | 0 | 0.2239 | 5.673 | 0.0022 | 0 | 0.0129 | 0.0086 | 0 | 0.0108 | 0.0022 | 0 |
| normal_tissue | Hoskinson_2022 | 0.1521 | 2.3782 | 0 | 0.0042 | 50.0232 | 0.8829 | 0 | 0 | 1.0561 | 0.0042 | 0 | 0 | 0 | 0 |
| normal_tissue | Hoskinson_2022 | 0.4593 | 0.0014 | 0 | 0 | 0 | 0.0055 | 0.0028 | 0 | 0.0096 | 0.0014 | 0 | 0 | 0.0014 | 0 |
| normal_tissue | Hoskinson_2022 | 0.853 | 0.0885 | 0 | 0.0751 | 0.0858 | 0.1851 | 0 | 0 | 0.0027 | 0.0161 | 0.0107 | 0 | 0 | 0 |
| normal_tissue | Hoskinson_2022 | 0.0308 | 0.0014 | 0 | 1.2305 | 0.2265 | 1.3088 | 51.1619 | 0 | 0.0014 | 0 | 0 | 0 | 1.1522 | 0 |
| normal_tissue | Hoskinson_2022 | 0.1001 | 0 | 0 | 0 | 0.012 | 7.3532 | 0.0015 | 0 | 0.009 | 0.0015 | 0 | 0 | 0 | 0 |
| normal_tissue | Hoskinson_2022 | 0.0942 | 0 | 0 | 0 | 0.0536 | 12.6429 | 0.0032 | 0 | 0.0162 | 0.0016 | 0 | 0 | 0 | 0 |
| normal_tissue | Hoskinson_2022 | 0.0541 | 8.9122 | 0 | 0 | 0 | 0.6858 | 0.0036 | 0 | 0.0018 | 0.0018 | 0 | 0 | 0 | 0 |
| normal_tissue | Hoskinson_2022 | 1.2032 | 0 | 0 | 0 | 0.0363 | 14.336 | 4.9279 | 0 | 0.0126 | 0 | 0 | 0 | 7.6229 | 0 |
| normal_tissue | Hoskinson_2022 | 0.274 | 0 | 0 | 0 | 0.0488 | 12.6004 | 0.0031 | 0 | 0.0252 | 0.0016 | 0 | 0 | 0 | 0 |
| normal_tissue | Hoskinson_2022 | 0.4893 | 0 | 0 | 0 | 0.0807 | 5.6247 | 11.9907 | 0 | 0.0099 | 0 | 0.0016 | 0 | 0 | 0 |
| normal_tissue | Hoskinson_2022 | 0.1554 | 15.2331 | 0 | 0.0061 | 0.0464 | 3.8173 | 2.8686 | 0 | 0.002 | 0 | 0 | 0 | 1.3061 | 0 |
| normal_tissue | Hoskinson_2022 | 0.8166 | 7.9546 | 0 | 0 | 0.0809 | 2.2372 | 10.4435 | 0 | 0.0052 | 0.0013 | 2.0545 | 0 | 0 | 6.2484 |
| normal_tissue | Hoskinson_2022 | 1.6994 | 0.0018 | 0 | 0.0018 | 0.0018 | 40.519 | 0 | 0 | 0.0092 | 0.0037 | 0 | 0 | 0.0018 | 0 |
| normal_tissue | Hoskinson_2022 | 0.8235 | 0 | 0 | 0 | 0 | 23.588 | 0.0031 | 0 | 0.0234 | 0.0031 | 0 | 0 | 0.0016 | 0 |
| normal_tissue | Hoskinson_2022 | 0.2347 | 0.0036 | 0 | 0 | 0 | 14.5762 | 2.4601 | 0 | 0.0197 | 0 | 0.0018 | 0 | 21.8921 | 0 |
| normal_tissue | Hoskinson_2022 | 0.8384 | 0 | 0 | 0 | 0.0018 | 11.0889 | 0 | 0 | 0.0461 | 0.0018 | 0 | 0 | 0 | 0 |
| normal_tissue | Hoskinson_2022 | 0.2418 | 0 | 0 | 0 | 0.0014 | 0.7688 | 0 | 0 | 0.0545 | 0 | 0 | 0 | 0.0014 | 0 |
| normal_tissue | Hoskinson_2022 | 0.0883 | 4.513 | 0 | 0 | 45.9573 | 1.1567 | 0 | 0 | 0.0046 | 0 | 0 | 0 | 0.0023 | 0 |
| normal_tissue | Hoskinson_2022 | 0 | 0.0028 | 0 | 0 | 0.0056 | 34.0133 | 0 | 0 | 0.1208 | 0 | 0 | 0 | 0 | 0 |
| normal_tissue | Hoskinson_2022 | 0.2365 | 3.4413 | 0 | 0 | 30.8755 | 2.1543 | 0.0051 | 0 | 0.0025 | 0 | 0 | 0 | 0 | 0 |
| normal_tissue | Hoskinson_2022 | 0.4148 | 0 | 0 | 0 | 0.0018 | 3.5447 | 1.29 | 0 | 0.0091 | 0 | 0 | 0 | 0 | 0 |
| normal_tissue | Hoskinson_2022 | 0.7039 | 0.0063 | 0 | 0 | 2.1054 | 1.4414 | 7.5287 | 0 | 0.0126 | 0.0021 | 0 | 0 | 0.0021 | 0 |
| normal_tissue | Hoskinson_2022 | 0.0374 | 0 | 0 | 0 | 0.0917 | 0.7713 | 13.9907 | 0 | 0.0094 | 0 | 0 | 0 | 0 | 0 |
| normal_tissue | Hoskinson_2022 | 0.1786 | 0 | 0 | 0 | 0 | 0.0456 | 0.002 | 0 | 0.004 | 0 | 0 | 0 | 0 | 0 |
| normal_tissue | Hoskinson_2022 | 0.1565 | 0 | 0 | 0 | 0.0487 | 3.1237 | 0.0052 | 0 | 0.0174 | 0 | 0 | 0 | 0 | 0 |
| normal_tissue | Hoskinson_2022 | 0.4667 | 0 | 0 | 0 | 0.0758 | 1.1177 | 19.6359 | 0 | 12.6957 | 0 | 0 | 0 | 0 | 0 |
| normal_tissue | Hoskinson_2022 | 0.1677 | 11.0859 | 0 | 0 | 15.9859 | 1.8979 | 2.2069 | 0 | 0 | 0 | 0 | 0 | 0 | 0 |
| normal_tissue | Hoskinson_2022 | 0.0962 | 0.0016 | 0 | 0 | 0.0192 | 8.0856 | 3.5729 | 0 | 0.0112 | 0.0016 | 0 | 0 | 0 | 0 |
| normal_tissue | Hoskinson_2022 | 0.6512 | 0.0018 | 0 | 0 | 0.2304 | 5.0609 | 0.0018 | 0 | 0.0145 | 0.0036 | 0 | 0 | 0 | 0 |
| normal_tissue | Hoskinson_2022 | 0.0893 | 4.3423 | 0 | 0 | 34.2557 | 2.6498 | 0.0051 | 0 | 0.0051 | 0.0026 | 0 | 0.0026 | 0 | 0 |
| normal_tissue | Hoskinson_2022 | 0.2399 | 0 | 0 | 0 | 0.1762 | 13.3195 | 37.2859 | 0 | 0.0037 | 0 | 0 | 0 | 0 | 0 |
| normal_tissue | Hoskinson_2022 | 0 | 7.5506 | 0 | 0 | 27.523 | 0.0022 | 0.0045 | 0 | 0 | 0 | 0 | 0 | 0 | 0 |
| normal_tissue | Hoskinson_2022 | 0 | 4.3012 | 0 | 0 | 37.4261 | 0.0023 | 0.0045 | 0 | 0 | 0 | 0 | 0 | 0 | 0 |
| normal_tissue | Hoskinson_2022 | 0.451 | 0 | 0 | 0 | 4.3174 | 3.1681 | 0.1569 | 0 | 0.0085 | 0 | 0 | 0 | 0 | 0 |
| normal_tissue | Hoskinson_2022 | 0.8977 | 0 | 0 | 0 | 0.1607 | 0.7776 | 12.0514 | 0 | 7.3227 | 0.0032 | 0 | 0.0016 | 0 | 0 |
| normal_tissue | Hoskinson_2022 | 0.8623 | 0 | 0 | 0 | 0.0019 | 3.9309 | 0 | 0 | 0.0058 | 0 | 0 | 0 | 15.1408 | 0 |
| normal_tissue | German_2023 | 0.1964 | 0.0228 | 0.6026 | 0 | 0.0111 | 0.1731 | 0.6137 | 0.0694 | 0 | 0.1687 | 0 | 0 | 0 | 0.0466 |
| normal_tissue | German_2023 | 0.5116 | 0 | 0.9466 | 0 | 0.0208 | 0.7336 | 0.0805 | 0 | 0 | 0.0013 | 0 | 0 | 0 | 0 |
| normal_tissue | German_2023 | 0.0769 | 0 | 0.9947 | 0.1398 | 0.1686 | 0.1989 | 0 | 0.0791 | 0 | 0.1487 | 0 | 0 | 0 | 0 |
| normal_tissue | German_2023 | 0.055 | 0.0041 | 1.2879 | 0 | 0 | 0.0041 | 0.0122 | 0.1243 | 0.1651 | 1.3287 | 0 | 0 | 0 | 0 |
| normal_tissue | German_2023 | 0.2747 | 0.008 | 6.8528 | 0 | 0.008 | 1.5171 | 0.0199 | 1.5848 | 0 | 5.5109 | 0.004 | 0 | 0 | 0.004 |
| normal_tissue | German_2023 | 0.1449 | 0 | 0.8822 | 0 | 0.0072 | 0.0968 | 0.001 | 0.1088 | 0 | 0.0222 | 0 | 0.0188 | 0 | 0 |
| normal_tissue | German_2023 | 0.3261 | 0.0261 | 1.3924 | 0 | 0 | 0.1183 | 0 | 0 | 0 | 0.0039 | 0 | 0 | 0 | 0 |
| normal_tissue | German_2023 | 0.1874 | 0.1299 | 1.3749 | 0.0004 | 0.084 | 0.0513 | 0.0161 | 0 | 0 | 0.0021 | 0 | 0.0302 | 0 | 0 |
| normal_tissue | German_2023 | 0.0809 | 0 | 0.5998 | 0 | 0.0003 | 0.1949 | 0.0227 | 0.0003 | 0 | 0.0217 | 0 | 0 | 0 | 0 |
| normal_tissue | German_2023 | 0.2061 | 0 | 0.842 | 0.0005 | 0 | 0.1225 | 0 | 0 | 0.0005 | 0.0443 | 0 | 0.0033 | 0 | 0 |
| normal_tissue | German_2023 | 0.2657 | 0.0293 | 1.2093 | 0.0011 | 0 | 0.599 | 0.1509 | 0.1002 | 0.0034 | 0.6508 | 0.0011 | 0 | 0 | 0.0023 |
| normal_tissue | German_2023 | 0.0587 | 0.0576 | 0.6181 | 0.0291 | 0.1092 | 0.1361 | 0.0192 | 0.0307 | 0 | 0.0598 | 0 | 0 | 0 | 0 |
| normal_tissue | German_2023 | 0.0505 | 0.0005 | 0.211 | 0 | 0.0974 | 0.0117 | 0.0025 | 0.0749 | 0.002 | 0.1692 | 0.0005 | 0 | 0 | 0.0015 |
| normal_tissue | German_2023 | 1.3877 | 0.0105 | 1.5658 | 0.0026 | 0.0026 | 0.0052 | 0.3875 | 0.364 | 0.0157 | 0.7122 | 0 | 0 | 0 | 0 |
| normal_tissue | German_2023 | 0.021 | 0.0012 | 0.8525 | 0 | 0 | 0.0433 | 0.0012 | 0.073 | 0.0346 | 0.5865 | 0 | 0.0012 | 0 | 0 |
| normal_tissue | German_2023 | 0.1549 | 0 | 0.9443 | 0.0193 | 0 | 0.0307 | 0 | 0.0673 | 0 | 0.0381 | 0.0005 | 0 | 0 | 0 |
| normal_tissue | German_2023 | 0.1438 | 0.0935 | 0.6166 | 0 | 0.0734 | 0.0402 | 0.0402 | 0.0322 | 0 | 0.002 | 0 | 0.008 | 0 | 0 |
| normal_tissue | German_2023 | 0.6363 | 0.0298 | 3.3073 | 0 | 0.0511 | 0.2384 | 0.3426 | 0.1518 | 0 | 0.1717 | 0.0582 | 0 | 0 | 0 |
| normal_tissue | German_2023 | 0.19 | 0.272 | 5.4917 | 0 | 0.0033 | 0.0033 | 0.0033 | 0.4358 | 0 | 3.5552 | 0.0066 | 0 | 0 | 0.0066 |
| normal_tissue | German_2023 | 0.2322 | 0.0273 | 2.0693 | 0 | 0 | 0.5959 | 0 | 0.0666 | 0 | 0.0632 | 0 | 0 | 0 | 0 |
| normal_tissue | German_2023 | 0.1093 | 0.0011 | 1.1262 | 0 | 0.0033 | 0.1888 | 0.0011 | 0 | 0 | 0.0221 | 0 | 0 | 0 | 0 |
| normal_tissue | German_2023 | 0.0054 | 0.0447 | 0.3291 | 0.0465 | 0.0268 | 0.0537 | 0 | 0 | 0 | 0.0393 | 0 | 0 | 0 | 0 |
| normal_tissue | German_2023 | 0.141 | 0.4204 | 1.6661 | 0 | 0.0183 | 0.3003 | 0.0157 | 0.0601 | 0.1306 | 1.922 | 0 | 0 | 0 | 0 |
| normal_tissue | German_2023 | 0.0516 | 0 | 1.2652 | 0.0914 | 0 | 0.0655 | 0.4727 | 0.2046 | 0.0159 | 0.6793 | 0 | 0 | 0 | 0 |
| normal_tissue | German_2023 | 0.1108 | 0.001 | 0.6878 | 0.1806 | 0 | 0.0726 | 0.129 | 0.065 | 0 | 0 | 0 | 0 | 0 | 0 |
| normal_tissue | German_2023 | 0.0627 | 0.0501 | 0.7366 | 0.0236 | 0.0557 | 0.0224 | 0.013 | 0.0745 | 0 | 0.022 | 0.0004 | 0 | 0 | 0 |
| normal_tissue | German_2023 | 1.4844 | 0.3899 | 3.7983 | 0 | 0.0148 | 0.42 | 0.2888 | 24.6035 | 0.2993 | 0.9971 | 0.0004 | 0.0487 | 0 | 0 |
| normal_tissue | German_2023 | 0.2583 | 0 | 0.4761 | 0 | 0.0456 | 0.1256 | 0.0005 | 0.0228 | 0 | 0.0831 | 0.0481 | 0 | 0 | 0 |
| normal_tissue | German_2023 | 0.1896 | 0 | 3.2599 | 0 | 0 | 0.0008 | 0 | 0.0126 | 0 | 0.0164 | 0 | 0 | 0 | 0 |
| normal_tissue | German_2023 | 0.246 | 0.001 | 1.0322 | 0.066 | 0.0168 | 0.4083 | 0 | 0 | 0 | 0.0199 | 0 | 0 | 0 | 0 |
| normal_tissue | German_2023 | 0.05 | 0.0266 | 0.3046 | 0.0395 | 0.0137 | 0.0483 | 0.0556 | 0.0218 | 0 | 0.0137 | 0 | 0.0016 | 0 | 0 |
| normal_tissue | German_2023 | 0.0812 | 0.0352 | 2.1598 | 0 | 0.0291 | 0.3784 | 0 | 0.1783 | 0 | 0.0061 | 0.0934 | 0 | 0 | 0 |
| normal_tissue | German_2023 | 0.3178 | 0 | 1.2498 | 0.0695 | 0.0855 | 0.181 | 0.0004 | 0 | 0 | 0.9598 | 0.158 | 0 | 0 | 0 |
| normal_tissue | German_2023 | 2.9634 | 0.1902 | 0.4681 | 0.0323 | 0.0692 | 0.7496 | 1.2445 | 0.0674 | 0 | 1.7088 | 0 | 0.0028 | 0 | 0 |
| normal_tissue | German_2023 | 0.1069 | 0.2537 | 0.638 | 0 | 0 | 0.1114 | 0.0001 | 0.0183 | 0 | 0.0213 | 0 | 0 | 0 | 0 |
| normal_tissue | German_2023 | 3.1982 | 0.2927 | 0 | 0 | 0.1543 | 0.9685 | 0.4789 | 0.7876 | 0 | 4.6456 | 0.0053 | 0 | 0 | 0 |
| normal_tissue | German_2023 | 5.3651 | 0 | 1.4674 | 0.0013 | 0 | 0.0914 | 0 | 0.0013 | 0 | 1.4449 | 0 | 0 | 0 | 0 |
| normal_tissue | German_2023 | 0.0071 | 0.0127 | 0.2065 | 0 | 0 | 0 | 0.0005 | 0.0219 | 0 | 0.5281 | 0 | 0 | 0 | 0 |
| normal_tissue | German_2023 | 3.9965 | 0.2344 | 0.4379 | 0 | 0.0496 | 0.3977 | 0.6641 | 0.011 | 0.0154 | 0.6178 | 0.0171 | 0 | 0 | 0 |
| normal_tissue | German_2023 | 0.462 | 0 | 0 | 0.0107 | 0 | 0.0107 | 0.6339 | 0.9347 | 0 | 7.1659 | 0 | 0 | 0 | 0 |
| normal_tissue | German_2023 | 1.1683 | 0.2458 | 2.2909 | 0 | 0 | 0.2655 | 0.6454 | 0.6243 | 0 | 0.2083 | 0 | 0 | 0 | 0 |
| normal_tissue | German_2023 | 0.0277 | 0 | 0.2578 | 0 | 0.0227 | 0.0216 | 0.0004 | 0.0092 | 0 | 0.0308 | 0 | 0 | 0 | 0 |
| normal_tissue | German_2023 | 0.1578 | 0 | 0.1987 | 0.0309 | 0.0205 | 0.1714 | 0 | 0.0355 | 0 | 0.0323 | 0 | 0.0195 | 0 | 0 |
| normal_tissue | German_2023 | 0.1197 | 0.0004 | 0.2994 | 0 | 0.0075 | 0.0114 | 0.0522 | 0 | 0 | 0.062 | 0 | 0.0098 | 0 | 0 |
| normal_tissue | German_2023 | 0.1561 | 0.0058 | 0.4973 | 0 | 0 | 0.0405 | 0.0347 | 0.0289 | 0 | 15.3686 | 0 | 0 | 0 | 0 |
| normal_tissue | German_2023 | 0.105 | 0.0077 | 0.0004 | 0 | 0 | 0.0039 | 0.0319 | 0.0611 | 0 | 0.0007 | 0.0004 | 0 | 0 | 0.0004 |
| normal_tissue | German_2023 | 1.0276 | 0.0212 | 0.1271 | 0 | 0 | 0.8793 | 1.0276 | 0.0318 | 0.1271 | 2.0129 | 0 | 0 | 0 | 0.2755 |
| normal_tissue | German_2023 | 0.4321 | 0 | 1.2483 | 0.0144 | 0 | 0 | 0.0019 | 0.1143 | 0 | 0.4542 | 0 | 0 | 0 | 0 |
| normal_tissue | German_2023 | 11.3543 | 0.684 | 0.0047 | 0.0315 | 0.1736 | 2.5872 | 0.7368 | 0.0638 | 0 | 0.0132 | 0.1944 | 0 | 0 | 0.0145 |
| normal_tissue | German_2023 | 0.0059 | 0 | 0.8268 | 0.0059 | 0 | 0.2647 | 0.0178 | 0.0714 | 0 | 1.2878 | 0 | 0 | 0 | 0.0387 |
| normal_tissue | German_2023 | 0.0696 | 0.0177 | 0.5397 | 0.0403 | 0.0309 | 0.0481 | 0.0088 | 0.0381 | 0 | 0.1558 | 0 | 0.0006 | 0 | 0 |
| normal_tissue | German_2023 | 0.0006 | 0.1801 | 4.4823 | 0 | 0 | 0.3049 | 0 | 0.4374 | 0 | 0.2129 | 0 | 0 | 0 | 0 |
| normal_tissue | German_2023 | 0.1594 | 0.0003 | 1.5392 | 0 | 0 | 0.0938 | 0.0006 | 0.154 | 0 | 0.0006 | 0 | 0 | 0 | 0 |
| normal_tissue | German_2023 | 0.0153 | 0.0671 | 0.4948 | 0 | 0 | 0 | 0 | 0.0261 | 0 | 0.11 | 0 | 0 | 0 | 0 |
| normal_tissue | German_2023 | 0.611 | 0.0297 | 1.766 | 0 | 0 | 0 | 0.0019 | 0.1551 | 0 | 0.1643 | 0 | 0 | 0 | 0 |
| normal_tissue | German_2023 | 0.1641 | 0.0075 | 0 | 0 | 0.0373 | 0.0746 | 0.3058 | 0.1492 | 0.0373 | 2.6626 | 0 | 0.097 | 0 | 0 |
| normal_tissue | German_2023 | 0.1305 | 0.0046 | 5.3005 | 0 | 0.003 | 0.4976 | 0.6174 | 0.1365 | 0 | 0.2185 | 0 | 0 | 0 | 0 |
| normal_tissue | German_2023 | 1.0335 | 0.0572 | 3.2059 | 0 | 0.4794 | 0.0176 | 0.343 | 0.0748 | 0 | 1.7327 | 0 | 0 | 0 | 0 |
| normal_tissue | German_2023 | 1.1389 | 0.1201 | 3.8621 | 0.0017 | 0.0017 | 0.9605 | 0.0017 | 0.2751 | 0.1067 | 0.7354 | 0 | 0 | 0 | 0 |
| normal_tissue | German_2023 | 0.0686 | 0.0322 | 0.3741 | 0.0018 | 0 | 0.1178 | 0.0352 | 0.0182 | 0.0049 | 0.1937 | 0 | 0 | 0 | 0 |
| normal_tissue | German_2023 | 0.1746 | 0.0749 | 1.1947 | 0 | 0.0284 | 0.0933 | 0.0016 | 0.01 | 0 | 0.0681 | 0.0814 | 0 | 0 | 0 |
| normal_tissue | German_2023 | 0.6309 | 0 | 4.8875 | 0 | 0.0044 | 0.998 | 0.29 | 0.2172 | 0 | 0.0087 | 0 | 0 | 0 | 0.1954 |
| normal_tissue | German_2023 | 0.5416 | 0.2928 | 7.8998 | 0.0342 | 0.0005 | 0.5946 | 3.2002 | 0.9605 | 0.0731 | 0.0075 | 0.4001 | 0 | 0 | 0 |
| normal_tissue | German_2023 | 0.6973 | 0.2116 | 3.23 | 0 | 0.0116 | 0.2201 | 0.2769 | 0.0003 | 0.0013 | 0.0346 | 0 | 0 | 0 | 0 |
| normal_tissue | German_2023 | 4.2248 | 0.1239 | 2.2229 | 0.1506 | 0.492 | 1.0325 | 3.6077 | 1.171 | 0.2502 | 1.3107 | 0.0061 | 0 | 0 | 1.6411 |
| normal_tissue | German_2023 | 0.4468 | 0.211 | 1.0013 | 0.0007 | 0.0706 | 0.9198 | 0.0131 | 0.0087 | 0 | 0.3842 | 0 | 0 | 0 | 0 |
| normal_tissue | German_2023 | 1.2672 | 0.0437 | 8.5181 | 0 | 0 | 0.9206 | 0.1624 | 0.2667 | 0 | 0.0737 | 0 | 0 | 0 | 0 |
| normal_tissue | German_2023 | 1.4705 | 0.2967 | 8.882 | 0.001 | 0.467 | 1.8573 | 0.0529 | 0.5326 | 0 | 0.4278 | 0.001 | 0 | 0 | 0 |
| normal_tissue | German_2023 | 0 | 0.0578 | 2.4738 | 0 | 0.0669 | 0.3209 | 0.0006 | 0.1144 | 0 | 0.3684 | 0.0006 | 0 | 0 | 0.0006 |
| normal_tissue | German_2023 | 16.0378 | 1.22 | 14.0105 | 0.2338 | 0.3727 | 16.4273 | 3.1253 | 0.0006 | 0 | 0.0381 | 1.344 | 0 | 0 | 0 |
| normal_tissue | German_2023 | 7.8809 | 1.1838 | 0.001 | 1.2169 | 0.3479 | 1.5569 | 27.5239 | 0.0479 | 1.6876 | 0.079 | 1.0011 | 0.0017 | 0 | 0.6904 |
| normal_tissue | German_2023 | 9.8395 | 1.0852 | 2.9834 | 0.0008 | 0.0398 | 6.2705 | 2.6885 | 0.173 | 0 | 0.0024 | 0.0853 | 0 | 0 | 0 |
| normal_tissue | German_2023 | 1.4307 | 0.1718 | 3.8621 | 0 | 0.1653 | 1.4347 | 0.2537 | 0.2826 | 0 | 0.2966 | 0 | 0 | 0 | 0 |
| normal_tissue | German_2023 | 2.601 | 0.5077 | 0.1681 | 0.0033 | 0.0297 | 1.4604 | 0.4418 | 1.5659 | 0.089 | 3.9164 | 0.3824 | 0 | 0 | 0.3626 |
| normal_tissue | German_2023 | 0.62 | 0.0141 | 4.6921 | 0 | 0.0141 | 1.7754 | 0.1127 | 0.0141 | 0 | 3.8185 | 0.7327 | 0 | 0 | 0.0141 |
| normal_tissue | German_2023 | 1.0456 | 0.1743 | 4.5789 | 0 | 0.0025 | 0.639 | 0.0051 | 0.1566 | 0.0076 | 3.5914 | 0 | 0 | 0 | 0.0682 |
| normal_tissue | German_2023 | 0.6403 | 0.1866 | 2.5149 | 0.2552 | 0.2715 | 2.5998 | 7.0263 | 0.02 | 0 | 0.0728 | 0.0003 | 0 | 0 | 0 |
| normal_tissue | German_2023 | 0.8414 | 0.1175 | 5.1332 | 0.0003 | 0.1425 | 0.9038 | 0.4429 | 0.1154 | 0 | 0.1124 | 0 | 0 | 0 | 0 |
| normal_tissue | German_2023 | 0.4887 | 0.0168 | 1.3578 | 0.0002 | 0.022 | 0.4684 | 0.0954 | 0.1082 | 0 | 0.0519 | 0.0353 | 0 | 0 | 0 |
| normal_tissue | German_2023 | 0.6078 | 0.2598 | 7.5326 | 0.0005 | 0.0584 | 0.0516 | 0.1658 | 0.313 | 0 | 0.0454 | 0.0005 | 0 | 0 | 1.1836 |
| normal_tissue | German_2023 | 0.2941 | 0.0489 | 1.3273 | 0.0183 | 0 | 54.0878 | 0.1891 | 0.0963 | 0.2875 | 0.0413 | 1.6321 | 0 | 0 | 0.0005 |
| normal_tissue | German_2023 | 1.2682 | 0.3259 | 8.6859 | 0 | 0.029 | 1.64 | 0.0032 | 0.2638 | 0 | 0.1751 | 0.0004 | 0 | 0 | 0 |
| normal_tissue | German_2023 | 3.6458 | 0.5849 | 5.07 | 0 | 0.3938 | 1.8011 | 0.0003 | 0.2507 | 0 | 0.0613 | 0.0277 | 0 | 0 | 0 |
| normal_tissue | German_2023 | 5.2069 | 0 | 4.0148 | 0.1933 | 0.1037 | 4.5657 | 0.0105 | 0.0559 | 0 | 0.1948 | 0 | 0 | 0 | 0 |
| normal_tissue | German_2023 | 13.2883 | 0.5891 | 1.8836 | 0.1072 | 0.0852 | 3.9683 | 7.9903 | 0.2032 | 0.5595 | 0.0123 | 0.0613 | 0 | 0 | 0.5457 |
| normal_tissue | German_2023 | 0.6253 | 0.173 | 0 | 0 | 0.0067 | 0.0333 | 0.02 | 0.5122 | 0 | 1.6231 | 0.286 | 0.133 | 0 | 0 |
| normal_tissue | German_2023 | 3.8525 | 0.0012 | 3.3216 | 0 | 0.3725 | 0.7306 | 0.493 | 0.6795 | 0 | 0.004 | 0 | 0 | 0 | 0 |
| normal_tissue | German_2023 | 0.8032 | 0.2752 | 2.4062 | 0 | 0 | 0.6706 | 0.4302 | 0 | 0 | 0.1505 | 0 | 0 | 0 | 0 |
| normal_tissue | German_2023 | 4.1976 | 0.0219 | 0.2696 | 0 | 0 | 0.6049 | 2.3247 | 0.6413 | 0 | 3.877 | 0 | 0 | 0 | 0.532 |
| normal_tissue | German_2023 | 1.399 | 1.5484 | 2.5041 | 0.0037 | 0.1692 | 0.1927 | 0.0012 | 0.203 | 0 | 0.2121 | 0 | 0 | 0 | 0 |
| normal_tissue | German_2023 | 4.1764 | 0.4437 | 0.004 | 0.2027 | 0.0045 | 0.1351 | 4.0149 | 0.3254 | 0.0089 | 0.1028 | 2.6596 | 0.0005 | 0 | 0.004 |
| normal_tissue | German_2023 | 1.0496 | 0.5072 | 0 | 0 | 0.1467 | 0.1034 | 0.2318 | 0.0545 | 0.2079 | 0.3419 | 0.0009 | 0 | 0 | 0.0718 |
| normal_tissue | German_2023 | 0.0983 | 0.0754 | 0.4988 | 0.0008 | 0 | 0.4584 | 0.0274 | 0.0091 | 0 | 0.0743 | 0.0118 | 0 | 0 | 0 |
| normal_tissue | German_2023 | 1.2231 | 0.0125 | 1.0361 | 0 | 0 | 0.7931 | 0.0743 | 0.0324 | 0 | 0.0004 | 0 | 0 | 0 | 0 |
| normal_tissue | German_2023 | 3.13 | 2.0322 | 0.0646 | 0.0009 | 1.481 | 1.2428 | 10.3114 | 0.5161 | 0.5623 | 0.4986 | 0.1837 | 0 | 0 | 1.2566 |
| normal_tissue | German_2023 | 0.1576 | 0.1399 | 0 | 0.0015 | 0.147 | 0.0931 | 0.2512 | 0.6952 | 0.0035 | 0.0594 | 0.0277 | 0 | 0 | 0.0035 |
| normal_tissue | German_2023 | 2.0721 | 0.0038 | 2.0146 | 0.001 | 0 | 0.0489 | 0 | 5.7444 | 0 | 1.3526 | 0.8173 | 0 | 0 | 0.5459 |
| normal_tissue | German_2023 | 0.1582 | 0.0011 | 0.7376 | 0 | 0.0011 | 0 | 0.0023 | 3.6583 | 0 | 1.2885 | 0 | 0 | 0 | 0 |
| normal_tissue | German_2023 | 2.8401 | 0.1617 | 1.4414 | 0.0475 | 0.3792 | 1.2449 | 2.2338 | 2.7784 | 1.7813 | 0.0939 | 0.5779 | 0 | 0 | 0.2512 |
| normal_tissue | German_2023 | 0.0599 | 0.0507 | 2.1518 | 0 | 0 | 0.0046 | 0.0046 | 0.1705 | 0 | 2.0043 | 0 | 0 | 0 | 0 |
| normal_tissue | German_2023 | 1.7864 | 0.0133 | 7.418 | 0 | 0 | 0.3453 | 0.0066 | 0 | 0 | 3.9049 | 0 | 0.0066 | 0 | 0 |
| normal_tissue | German_2023 | 0.8833 | 0 | 6.5018 | 0 | 0.0965 | 0.339 | 0.0014 | 0.3087 | 0 | 0.2453 | 0 | 0 | 0 | 0 |
| normal_tissue | German_2023 | 0.2221 | 0 | 0.7374 | 0 | 0.0006 | 0.9333 | 0.0012 | 0.1815 | 0.0006 | 0.1809 | 0 | 0 | 0 | 0 |
| normal_tissue | German_2023 | 3.4635 | 1.0252 | 5.9644 | 0 | 0.0746 | 1.045 | 2.6576 | 23.5836 | 2.4989 | 0.0115 | 0 | 0 | 0 | 0.1225 |
| normal_tissue | German_2023 | 0.7421 | 0.0777 | 3.0009 | 0 | 0 | 1.2256 | 0.3981 | 7.2858 | 0 | 0.3756 | 0 | 0 | 0 | 0 |
| normal_tissue | German_2023 | 1.7 | 0.0174 | 2.2345 | 0.0052 | 0.6297 | 1.2972 | 2.3657 | 0.39 | 0.7162 | 0.0992 | 0.0209 | 0 | 0 | 0.202 |
| normal_tissue | German_2023 | 1.2082 | 0.2511 | 8.3545 | 0 | 0 | 0.0045 | 0 | 0.848 | 0 | 0.0094 | 0 | 0 | 0 | 0.001 |
| normal_tissue | German_2023 | 0.6536 | 0.0737 | 0.0426 | 0 | 0.0016 | 0.4693 | 0.0192 | 0.3037 | 0.0088 | 0.4158 | 0.0628 | 0.0213 | 0 | 0.0005 |
| normal_tissue | German_2023 | 3.3723 | 0.8944 | 6.0437 | 0.3068 | 0.4366 | 0.0047 | 0.6962 | 0.0071 | 0 | 0 | 0 | 0 | 0 | 0 |
| normal_tissue | German_2023 | 1.4173 | 0.0062 | 4.4954 | 0 | 0.0909 | 0.0015 | 0.2064 | 0 | 0 | 0.02 | 0 | 0 | 0 | 0 |
| normal_tissue | German_2023 | 0.4753 | 0 | 3.5678 | 0.0035 | 0 | 0.0619 | 0.0035 | 0.3279 | 0 | 0.0855 | 0 | 0 | 0 | 0 |
| normal_tissue | German_2023 | 0.1187 | 0 | 3.3951 | 0 | 0.0006 | 0.1714 | 0.0025 | 0.287 | 0 | 0.1137 | 0.0006 | 0 | 0 | 0 |
| normal_tissue | German_2023 | 1.6032 | 0.5953 | 8.5165 | 0.5615 | 0 | 2.0632 | 0 | 0 | 0 | 1.2785 | 0 | 0 | 0 | 0 |
| normal_tissue | German_2023 | 0.941 | 0 | 3.0015 | 0.1749 | 0 | 2.111 | 2.6012 | 0.404 | 0 | 0.4335 | 0 | 0 | 0 | 0 |
| normal_tissue | German_2023 | 1.7991 | 4.4419 | 20.6292 | 0.9042 | 0 | 0.0047 | 0 | 1.6826 | 0 | 0.7457 | 0 | 0.0047 | 0 | 0 |
| normal_tissue | German_2023 | 0.5248 | 0 | 3.7347 | 0 | 0 | 1.7797 | 0.0028 | 0.9713 | 0 | 0.2581 | 0.6454 | 0 | 0 | 0 |
| normal_tissue | German_2023 | 2.4423 | 0 | 6.1166 | 0 | 0 | 0.9106 | 0.0013 | 0.6289 | 0 | 0.445 | 0 | 0 | 0 | 0 |
| normal_tissue | German_2023 | 5.167 | 0.0024 | 0.0694 | 0.001 | 0.0019 | 2.668 | 0.0225 | 7.9116 | 0.001 | 0.0081 | 0.0019 | 0 | 0 | 0.3383 |
| BC_tissue | Hoskinson_2022 | 2.0528 | 0.0015 | 0 | 0 | 0 | 5.586 | 0.9362 | 0 | 0.006 | 0.0075 | 0 | 0.0134 | 0 | 0 |
| BC_tissue | Hoskinson_2022 | 0.0113 | 9.2969 | 0 | 0 | 0.7416 | 0 | 2.2926 | 0 | 0 | 0 | 0 | 0.0151 | 0 | 0 |
| BC_tissue | Hoskinson_2022 | 0.7355 | 0.0014 | 0 | 0.0014 | 0 | 1.775 | 12.2076 | 0 | 0.0043 | 0.0029 | 0.0043 | 0.0087 | 0 | 0 |
| BC_tissue | Hoskinson_2022 | 0.0249 | 0 | 0 | 0.0028 | 0.4489 | 4.7298 | 0 | 0 | 0.0028 | 0 | 0 | 0.0748 | 0 | 0 |
| BC_tissue | Hoskinson_2022 | 0.2866 | 5.8527 | 0 | 0.0022 | 0.0022 | 1.5214 | 0.0022 | 0 | 0 | 0.0065 | 0 | 0.0129 | 9.8522 | 0 |
| normal_tissue | German_2023 | 1.7835 | 0.0077 | 0.0017 | 0.0009 | 0.0043 | 0.8503 | 0.5059 | 4.2822 | 0.0068 | 0.5008 | 0 | 0 | 0 | 0.0009 |
| normal_tissue | German_2023 | 0.1036 | 0.0072 | 0 | 0.1642 | 0 | 0.2973 | 0.0646 | 0.0757 | 0.012 | 0.9637 | 0.0024 | 0 | 0 | 0.1793 |
| normal_tissue | German_2023 | 0.2117 | 0.4207 | 3.4613 | 0.0663 | 0.259 | 0.0009 | 0 | 0 | 0 | 0.0005 | 0 | 0 | 0 | 0 |
| normal_tissue | German_2023 | 3.1301 | 0.7793 | 2.1459 | 0.1403 | 0.0594 | 0.5221 | 1.2566 | 0.1304 | 0 | 0.1636 | 0 | 0.0809 | 0 | 0 |
| normal_tissue | German_2023 | 0.2158 | 0.0005 | 1.1323 | 0 | 0 | 0.2673 | 0 | 0.1216 | 0 | 0.0762 | 0 | 0 | 0 | 0 |
| normal_tissue | German_2023 | 0.2792 | 0.3749 | 6.0901 | 0 | 0 | 0.1281 | 0.1181 | 0.2328 | 0 | 0.1242 | 0 | 0 | 0 | 0 |
| normal_tissue | German_2023 | 0.148 | 0 | 0.7655 | 0.0339 | 0 | 0.0661 | 0 | 0 | 0 | 0.0101 | 0 | 0 | 0 | 0 |
| normal_tissue | German_2023 | 0.4583 | 0.0048 | 1.1026 | 0.0016 | 0.0443 | 0.0253 | 0.1536 | 0.0253 | 0.0008 | 0.2034 | 0 | 0 | 0 | 0 |
| normal_tissue | German_2023 | 0.2161 | 0.0059 | 0.8208 | 0.0306 | 0 | 0.2921 | 0 | 0.0373 | 0 | 0.0344 | 0.0785 | 0.005 | 0 | 0.0827 |
| normal_tissue | German_2023 | 0.4258 | 0.0366 | 0.5325 | 0 | 0 | 0.1262 | 0.1079 | 0 | 0.0006 | 0.0265 | 0.0059 | 0 | 0 | 0 |
| normal_tissue | German_2023 | 0.0134 | 0.0017 | 0.7693 | 0 | 0.0017 | 0.0017 | 0 | 0.0447 | 0 | 0.0453 | 0 | 0 | 0 | 0 |
| normal_tissue | German_2023 | 0.1091 | 0 | 0.5762 | 0 | 1.9886 | 0.014 | 0.1566 | 0.0308 | 0 | 0.014 | 0.0028 | 0 | 0 | 0 |
| normal_tissue | German_2023 | 6.9987 | 0 | 32.1164 | 0 | 0 | 1.8506 | 3.432 | 0.0168 | 0 | 0 | 0 | 0 | 0 | 0 |
| normal_tissue | German_2023 | 0.7199 | 1.0439 | 13.1007 | 0 | 0 | 0.3641 | 0.0006 | 0.2544 | 0 | 0.0019 | 0 | 0 | 0 | 0 |
| normal_tissue | German_2023 | 0.8656 | 0.0309 | 2.0003 | 0 | 0 | 0.1108 | 0.0009 | 0.0962 | 0 | 0.0241 | 0 | 0 | 0 | 0 |
| normal_tissue | German_2023 | 0.5787 | 0.0812 | 2.1997 | 0 | 0 | 1.3943 | 0.0068 | 0.0677 | 0 | 0.0745 | 0 | 0 | 0 | 0 |
| normal_tissue | German_2023 | 0.0694 | 0.1101 | 1.6042 | 0 | 0.0954 | 1.0846 | 0.2213 | 0.0235 | 0 | 0.1805 | 0 | 0 | 0 | 0 |
| normal_tissue | German_2023 | 0.0361 | 0.0338 | 0.8108 | 0 | 0 | 0 | 0 | 0 | 0 | 0.0015 | 0 | 0 | 0 | 0 |
| normal_tissue | German_2023 | 0.1732 | 0 | 2.1364 | 0 | 0 | 0.184 | 0 | 0.1973 | 0 | 0.3019 | 0 | 0 | 0 | 0 |
| normal_tissue | German_2023 | 0.1127 | 0 | 1.4011 | 0.2855 | 0.0038 | 0.2667 | 3.6437 | 0.0038 | 0 | 0.0639 | 0 | 0 | 0 | 0 |
| normal_tissue | German_2023 | 0.0231 | 0.0246 | 0.158 | 0 | 0 | 0.004 | 0.2183 | 0.0294 | 0 | 0.0375 | 0 | 0 | 0 | 0 |
| normal_tissue | German_2023 | 0.0037 | 0 | 0.7447 | 0 | 0 | 0 | 0.0037 | 0.0372 | 0 | 0.0745 | 0 | 0 | 0 | 0 |
| normal_tissue | German_2023 | 0.6444 | 0.0713 | 0.378 | 0.1386 | 0 | 0.5112 | 0.2906 | 0.0619 | 0.0282 | 0.7103 | 0.0161 | 0 | 0 | 0 |
| normal_tissue | German_2023 | 0.0359 | 0.0008 | 0.0444 | 0 | 0 | 0.0023 | 0.0023 | 0.117 | 0 | 0.2891 | 0.0008 | 0.0979 | 0 | 0 |
| normal_tissue | German_2023 | 0.0024 | 0.0191 | 1.0812 | 0 | 0 | 0.0383 | 0.0012 | 0.0287 | 0 | 0.0478 | 0 | 0 | 0 | 0 |
| normal_tissue | German_2023 | 0.3826 | 0.0785 | 0.2551 | 0 | 0 | 0.1733 | 0.0981 | 0.5625 | 0 | 2.0013 | 0.1537 | 0.0556 | 0 | 0 |
| normal_tissue | German_2023 | 0.0075 | 0 | 0.1035 | 0 | 0 | 0.0077 | 0.0234 | 0.0175 | 0 | 0.0026 | 0 | 0.0067 | 0 | 0 |
| normal_tissue | German_2023 | 3.348 | 0.2155 | 0.7199 | 0 | 0.0433 | 0.8395 | 1.8983 | 0.1307 | 0.0536 | 0.051 | 0.0009 | 0.0197 | 0 | 0 |
| normal_tissue | German_2023 | 0.0033 | 0.0631 | 0.3954 | 0 | 0 | 0.0083 | 0.0017 | 0.0083 | 0 | 0.0033 | 0 | 0 | 0 | 0.0781 |
| normal_tissue | German_2023 | 0.6077 | 0 | 2.4404 | 0 | 0 | 0.4951 | 0 | 0.2958 | 0.0003 | 0.0392 | 0.1015 | 0 | 0 | 0 |
| normal_tissue | German_2023 | 0.2948 | 0.0795 | 0.4743 | 0 | 0.0581 | 0.0914 | 0.135 | 0.0273 | 0 | 0.4153 | 0 | 0 | 0 | 0.0145 |
| normal_tissue | German_2023 | 0.1152 | 0.0073 | 0.6303 | 0.0364 | 0.0028 | 0.0178 | 0.0619 | 0.0457 | 0.0121 | 0.0894 | 0 | 0 | 0 | 0 |
| normal_tissue | German_2023 | 0.1545 | 0.0673 | 0.9053 | 0 | 0.0336 | 0.1784 | 0.0739 | 0.0041 | 0 | 0.1407 | 0 | 0 | 0 | 0 |
| normal_tissue | German_2023 | 1.8869 | 0.4129 | 0.7113 | 0.0651 | 0.0179 | 1.4749 | 0.341 | 0.1864 | 0 | 0.0979 | 0 | 0.0051 | 0 | 0 |
| normal_tissue | German_2023 | 0.1793 | 0 | 2.1603 | 0 | 0 | 0.1259 | 0 | 0.0003 | 0 | 0.0616 | 0 | 0 | 0 | 0 |
| normal_tissue | German_2023 | 0.2125 | 0.1611 | 2.0265 | 0.2485 | 0 | 0.0004 | 0.0059 | 0.2678 | 0.0018 | 0.0392 | 0 | 0 | 0 | 0 |
| normal_tissue | German_2023 | 1.4127 | 0.1582 | 3.1765 | 0 | 0.1511 | 0.3381 | 1.7828 | 0.4685 | 0.2267 | 0 | 0 | 0 | 0 | 0 |
| normal_tissue | German_2023 | 0.0425 | 0 | 1.8147 | 0 | 0 | 0.1032 | 0.1261 | 0.1989 | 0.0004 | 0.0035 | 0.0004 | 0.0412 | 0 | 0 |
| normal_tissue | German_2023 | 0.3659 | 0.0883 | 0 | 0 | 0 | 0.4164 | 0.1767 | 0.6688 | 0.0126 | 9.0095 | 0.0252 | 0.0126 | 0 | 0 |
| normal_tissue | German_2023 | 0.8029 | 0.028 | 0.1214 | 0 | 0.0093 | 0.1214 | 1.055 | 0.3828 | 0 | 2.3901 | 0.0093 | 0.3828 | 0 | 0 |
| normal_tissue | German_2023 | 4.4371 | 0 | 5.2732 | 0 | 0 | 0 | 0 | 0.0005 | 0 | 0.0009 | 0 | 0 | 0 | 0 |
| normal_tissue | German_2023 | 3.9571 | 0.0639 | 0.2495 | 0.2122 | 0.0524 | 0.9795 | 0.063 | 0.238 | 0.0009 | 0.2788 | 0.4893 | 0 | 0 | 0.2584 |
| normal_tissue | German_2023 | 0.8009 | 0.5297 | 0.9982 | 0 | 0.4478 | 0.6855 | 0.001 | 0.1825 | 0.1884 | 0.7417 | 0.1578 | 0 | 0 | 0 |
| normal_tissue | German_2023 | 0.768 | 0.2477 | 3.3567 | 0 | 0.0008 | 0.0248 | 0.6045 | 0 | 0 | 0.3171 | 0 | 0 | 0 | 0 |
| normal_tissue | German_2023 | 1.4599 | 0.0333 | 1.4342 | 0.0303 | 0 | 0.7012 | 0.0288 | 0.2287 | 0.1 | 0.7345 | 0.2953 | 0 | 0 | 0 |
| normal_tissue | German_2023 | 1.0706 | 0.3053 | 1.7882 | 0 | 0.0654 | 0.0478 | 0.566 | 0.0062 | 0 | 0.1952 | 0 | 0 | 0 | 0.0239 |
| normal_tissue | German_2023 | 0.0071 | 0.0495 | 1.1912 | 0.0008 | 0 | 0.0051 | 0.1905 | 0.0452 | 0 | 0.1646 | 0 | 0 | 0 | 0 |
| normal_tissue | German_2023 | 0.2504 | 0.1505 | 0 | 0.1093 | 0 | 9.4712 | 0.0023 | 0.4262 | 0.0005 | 0.0005 | 0 | 0 | 0 | 0 |
| normal_tissue | German_2023 | 0.3168 | 0.0922 | 0.1893 | 0 | 0.032 | 0.0414 | 0.4244 | 0.0712 | 0.0044 | 0.0541 | 0 | 0.0017 | 0 | 0 |
| normal_tissue | German_2023 | 2.6818 | 0.3657 | 3.8297 | 0 | 0.105 | 0.0339 | 0.1862 | 0 | 0 | 1.3138 | 0 | 0 | 0 | 0 |
| normal_tissue | German_2023 | 0.1196 | 0.4343 | 3.0694 | 0 | 0.0113 | 0.0063 | 0.0013 | 0.1977 | 0 | 0.4205 | 0 | 0 | 0 | 0 |
| normal_tissue | German_2023 | 0.0016 | 0.0322 | 0.4892 | 0 | 0 | 0.0006 | 0.099 | 0.0474 | 0 | 0.029 | 0.0268 | 0 | 0 | 0 |
| normal_tissue | German_2023 | 0.0592 | 0.0038 | 0.3311 | 0.0077 | 0.0307 | 0.1959 | 0 | 0.0115 | 0 | 0.2097 | 0 | 0.0077 | 0 | 0 |
| normal_tissue | German_2023 | 0.0554 | 0.0004 | 0 | 0.0146 | 0 | 0.1112 | 0.0039 | 0.0477 | 0 | 0.0219 | 0.0004 | 0.049 | 0 | 0 |
| normal_tissue | German_2023 | 0.8285 | 0.0036 | 0.0012 | 0.0131 | 0.0478 | 0.6029 | 0.2817 | 0.1516 | 0 | 0.1946 | 0.1122 | 0 | 0 | 0.2268 |
| normal_tissue | German_2023 | 0.2576 | 0.0051 | 0.352 | 0 | 0 | 0.0336 | 0 | 0.018 | 0 | 0.0149 | 0 | 0 | 0 | 0 |
| normal_tissue | German_2023 | 0.5724 | 0.0967 | 0.5735 | 0 | 0.0767 | 0.1745 | 0.0822 | 0.0178 | 0 | 1.6059 | 0 | 0 | 0.0122 | 0 |
| normal_tissue | German_2023 | 0.1381 | 0 | 0.8461 | 0 | 0.0011 | 0.2168 | 0.0518 | 0.0188 | 0 | 0.0555 | 0 | 0 | 0 | 0 |
| normal_tissue | German_2023 | 0.1481 | 0.1404 | 2.6394 | 0 | 0 | 0.0078 | 0 | 0.2339 | 0 | 2.1559 | 0 | 0 | 0 | 0 |
| normal_tissue | German_2023 | 0.1144 | 0.0512 | 0 | 0.0004 | 0 | 0.136 | 0.0014 | 0.1688 | 0 | 0.006 | 0 | 0 | 0 | 0 |
| normal_tissue | German_2023 | 0.2664 | 0.0444 | 7.7845 | 0 | 0 | 0 | 0 | 0.0592 | 0 | 4.7358 | 0 | 0.0148 | 0 | 0 |
| normal_tissue | German_2023 | 0.1904 | 0.1005 | 1.3765 | 0.0758 | 0.0219 | 0.4931 | 0.0006 | 0.0275 | 0.0006 | 0.8514 | 0 | 0 | 0 | 0 |
| normal_tissue | German_2023 | 0.9685 | 0.1091 | 4.8207 | 0 | 0.0006 | 0.0006 | 0.2472 | 0.1022 | 0 | 1.2334 | 0 | 0 | 0 | 0 |
| normal_tissue | German_2023 | 0.1435 | 0 | 1.763 | 0 | 0 | 0.3763 | 0.0562 | 0.1224 | 0 | 0.3361 | 0 | 0 | 0 | 0 |
| normal_tissue | German_2023 | 1.7517 | 0.0144 | 1.2744 | 0 | 0.0043 | 0.3007 | 0.3658 | 0.0025 | 0 | 0.1288 | 0 | 0 | 0 | 0 |
| normal_tissue | German_2023 | 1.1895 | 4.5289 | 1.3193 | 0.0704 | 0.2448 | 0.0813 | 0.4292 | 0.0396 | 1.4234 | 0.3618 | 0.0644 | 0 | 0 | 0 |
| normal_tissue | German_2023 | 1.0298 | 0.1634 | 7.9539 | 0.2527 | 0.1024 | 0.4951 | 0.0036 | 0.2066 | 0.367 | 0.0904 | 0 | 0 | 0 | 0 |
| normal_tissue | German_2023 | 1.7592 | 0.038 | 4.4453 | 0 | 0 | 0.4551 | 0.145 | 0.0941 | 0 | 0.2611 | 0 | 0 | 0 | 0 |
| normal_tissue | German_2023 | 2.159 | 0.2623 | 0.0009 | 0 | 0 | 2.0139 | 1.0511 | 1.8409 | 0.0009 | 0.0409 | 0 | 0 | 0 | 0 |
| normal_tissue | German_2023 | 5.2246 | 0.1362 | 0.5973 | 0 | 0.2421 | 0.4244 | 2.2625 | 0.0965 | 0.1693 | 0.4759 | 0.0284 | 0.0178 | 0 | 0 |
| normal_tissue | German_2023 | 0.2488 | 0.0003 | 6.74 | 1.1282 | 0.2602 | 6.998 | 2.4817 | 0.9661 | 0 | 0.0007 | 0 | 0 | 0 | 0 |
| normal_tissue | German_2023 | 0.5226 | 0.0675 | 2.1545 | 0.0065 | 0.2239 | 0.1448 | 0.484 | 0.1484 | 0 | 0.0881 | 0 | 0 | 0 | 0 |
| normal_tissue | German_2023 | 2.4996 | 0.4668 | 12.4538 | 0 | 0 | 0.8504 | 0 | 0.8704 | 0 | 0.0011 | 0 | 0 | 0 | 0 |
| normal_tissue | German_2023 | 27.5392 | 5.0007 | 0.4694 | 0 | 0.0112 | 3.4555 | 1.9698 | 0.4405 | 0 | 0.0083 | 0.0006 | 0 | 0 | 0.0012 |
| normal_tissue | German_2023 | 0.0254 | 0.8973 | 6.7726 | 0 | 0.1985 | 0.3418 | 0.0025 | 0.0004 | 0.5831 | 0.1147 | 0 | 0 | 0 | 0 |
| normal_tissue | German_2023 | 0.0755 | 0 | 1.1063 | 0.087 | 0.0435 | 0.0315 | 0.0005 | 0.118 | 0 | 0.091 | 0 | 0.018 | 0 | 0 |
| normal_tissue | German_2023 | 1.1453 | 1.2256 | 1.2515 | 0 | 0.1589 | 0.4397 | 0.373 | 0.0657 | 0 | 0.0647 | 0 | 0.0255 | 0 | 0.015 |
| normal_tissue | German_2023 | 0.3173 | 0.1003 | 0.9289 | 0.0148 | 0.1749 | 0.0517 | 0.7878 | 0.02 | 0.0282 | 0.8677 | 0 | 0.0009 | 0 | 0 |
| normal_tissue | German_2023 | 4.1091 | 1.0785 | 21.5844 | 0 | 5.64 | 1.8023 | 0.7784 | 1.5153 | 0 | 0.162 | 0 | 0 | 0 | 0 |
| normal_tissue | German_2023 | 0.0619 | 0.0387 | 1.2926 | 0 | 0.078 | 0.0035 | 0.0011 | 0.0028 | 0 | 0.0323 | 0 | 0 | 0 | 0 |
| normal_tissue | German_2023 | 3.0929 | 13.2906 | 0.0251 | 0.1595 | 0.0027 | 17.9017 | 12.0779 | 0.0007 | 0.0111 | 0.0023 | 0.0767 | 0 | 0 | 0.1521 |
| normal_tissue | German_2023 | 0.1866 | 0.0017 | 1.78 | 0.0625 | 0.0034 | 0.0051 | 0.2407 | 0 | 0.0574 | 0.1199 | 0 | 0 | 0 | 0 |
| normal_tissue | German_2023 | 4.5714 | 0.151 | 8.3164 | 0 | 0 | 2.6863 | 3.8854 | 0.0007 | 0 | 0.0063 | 0 | 0 | 0 | 0 |
| normal_tissue | German_2023 | 0.1117 | 0.0061 | 1.1843 | 0 | 0.0321 | 0.0627 | 0 | 0.0275 | 0 | 0.433 | 0 | 0 | 0 | 0 |
| normal_tissue | German_2023 | 0.6275 | 0.1914 | 0.0651 | 0.0583 | 0.0004 | 0.0365 | 1.5144 | 1.108 | 0.0544 | 0.0125 | 0.0007 | 0 | 0 | 0 |
| normal_tissue | German_2023 | 0.2297 | 0.2778 | 0.0427 | 0.0053 | 0.0427 | 0.0748 | 0.1549 | 0.1389 | 0.0374 | 8.3494 | 0.016 | 0 | 0 | 0.0481 |
| normal_tissue | German_2023 | 0.5083 | 0.0157 | 1.1093 | 0 | 0 | 0.2076 | 0.0013 | 0.0761 | 0 | 0.0309 | 0 | 0 | 0 | 0 |
| normal_tissue | German_2023 | 0.4266 | 0.0933 | 0.3162 | 0.1179 | 0.0191 | 0.3856 | 0.3336 | 0.0359 | 0 | 0.0468 | 0.1207 | 0 | 0 | 0 |
| normal_tissue | German_2023 | 0.5089 | 0.1491 | 0 | 0 | 0.0175 | 0.2632 | 0.0614 | 0.2369 | 0.1491 | 11.8881 | 0.0175 | 0 | 0 | 0.0088 |
| normal_tissue | German_2023 | 2.1489 | 0.3184 | 0.0012 | 0.0035 | 0.0012 | 0.1274 | 5.9049 | 0.4516 | 1.1787 | 0.5025 | 0.0081 | 0.0012 | 0 | 0.1343 |
| normal_tissue | German_2023 | 0.7191 | 0.0009 | 5.8187 | 0 | 0.0005 | 0.3664 | 0.0005 | 0.1563 | 0 | 0.1168 | 0 | 0.0005 | 0 | 0 |
| normal_tissue | German_2023 | 0.4101 | 0.0844 | 2.79 | 0 | 0.0068 | 0.0776 | 0.6819 | 0.081 | 0 | 0.7038 | 0 | 0 | 0 | 0 |
| normal_tissue | German_2023 | 0.2069 | 0 | 2.2163 | 0 | 0.0842 | 0.5506 | 0.1543 | 0.2139 | 0 | 0.9398 | 0 | 0 | 0 | 0 |
| normal_tissue | German_2023 | 15.2112 | 0.0664 | 0.0041 | 0 | 0.0027 | 0.2223 | 3.7882 | 0.206 | 0.4174 | 0.3429 | 0 | 0.0501 | 0 | 0 |
| normal_tissue | German_2023 | 0.0439 | 0.0219 | 1.0072 | 0.0024 | 0.0024 | 0.0854 | 0.1219 | 0.0902 | 0 | 0.7414 | 0 | 0 | 0 | 0 |
| normal_tissue | German_2023 | 1.188 | 0.002 | 2.529 | 0 | 0.0007 | 0.1835 | 0 | 0.0014 | 0 | 0.0163 | 0 | 0 | 0 | 0 |
| normal_tissue | German_2023 | 0.6066 | 0.212 | 3.8994 | 0 | 0 | 1.2181 | 0 | 0.6483 | 0.0331 | 0.3517 | 0.0012 | 0 | 0 | 0 |
| normal_tissue | German_2023 | 0.812 | 0 | 15.3481 | 0 | 0 | 4.5004 | 0.0099 | 1.1734 | 0 | 0.4307 | 0 | 0 | 0 | 0 |
| normal_tissue | German_2023 | 0.1952 | 0.0618 | 1.9882 | 0 | 0 | 0.3299 | 0 | 0.0049 | 0 | 0.477 | 0 | 0 | 0 | 0 |
| normal_tissue | German_2023 | 2.0427 | 0.6634 | 7.3867 | 0 | 0 | 0.5006 | 0 | 0.4402 | 0 | 0.0186 | 0 | 0 | 0 | 0 |
| normal_tissue | German_2023 | 0.2393 | 0 | 2.5905 | 0 | 0.0011 | 3.8601 | 0.0011 | 0.1344 | 0 | 0.0076 | 0 | 0 | 0 | 0 |
| normal_tissue | German_2023 | 0.1375 | 0.1439 | 3.664 | 0 | 0 | 0.4102 | 0.946 | 0 | 0 | 0.0207 | 0 | 0 | 0 | 0 |
| normal_tissue | German_2023 | 24.1956 | 0.1134 | 0.4733 | 0.2517 | 0.0569 | 0.6332 | 1.8303 | 0.5065 | 2.1691 | 0.1047 | 0.7936 | 0 | 0 | 0.5858 |
| normal_tissue | German_2023 | 4.9493 | 0.3273 | 0.0019 | 0.5144 | 0.0027 | 13.6385 | 0.655 | 0.3845 | 2.1201 | 0.0119 | 0.0092 | 0.0004 | 0 | 0.0031 |
| normal_tissue | German_2023 | 0.4506 | 0.2813 | 0.2526 | 0 | 0.1146 | 0.3021 | 0.0443 | 1.1173 | 0.0469 | 1.7893 | 0.0026 | 0 | 0 | 0 |
| normal_tissue | German_2023 | 2.4203 | 1.1274 | 4.7082 | 0 | 1.0818 | 0.0014 | 0.0014 | 0.0014 | 0 | 0.0014 | 0.0345 | 0.1504 | 0 | 0 |
| normal_tissue | German_2023 | 0.0006 | 0 | 2.6316 | 0 | 0.3627 | 0.1525 | 0.1443 | 0 | 0 | 0.0483 | 0 | 0 | 0 | 0 |
| normal_tissue | German_2023 | 4.6803 | 1.2411 | 0.0027 | 0.2908 | 0.0154 | 19.5374 | 8.242 | 2.1386 | 0.0131 | 0.1512 | 0.8129 | 0.0012 | 0 | 0.0085 |
| normal_tissue | German_2023 | 0.1283 | 0 | 0.5895 | 0.0124 | 0.0289 | 0.0532 | 0.0786 | 0.0522 | 0 | 0.0637 | 0 | 0 | 0 | 0 |
| normal_tissue | German_2023 | 0.0113 | 0.0676 | 0.8627 | 0 | 0.0477 | 0.0199 | 0.0444 | 0.0504 | 0.0179 | 0.0338 | 0 | 0 | 0 | 0 |
| normal_tissue | German_2023 | 0.2002 | 0 | 2.1469 | 0.0539 | 0 | 0.2456 | 0.0932 | 0 | 0 | 0.0331 | 0 | 0 | 0 | 0 |
| normal_tissue | German_2023 | 1.1415 | 0.5519 | 1.281 | 0 | 0.3424 | 0.6658 | 0.7644 | 0.1221 | 1.5774 | 0.0399 | 0.1753 | 0 | 0 | 0 |
| normal_tissue | German_2023 | 0.5265 | 0.0235 | 2.3048 | 0 | 0 | 0.2545 | 0.1303 | 0.2972 | 0 | 0.104 | 0.2102 | 0 | 0 | 0 |
| normal_tissue | German_2023 | 0.2154 | 0 | 0.8355 | 0.1542 | 0.0403 | 0.0436 | 0 | 0 | 0 | 0.0403 | 0 | 0 | 0 | 0 |
| normal_tissue | German_2023 | 0.0555 | 0.0003 | 0.7096 | 0.0758 | 0.0466 | 0.0241 | 0.0181 | 0.006 | 0 | 0.0698 | 0 | 0 | 0 | 0 |
| normal_tissue | German_2023 | 0.1409 | 0.0852 | 0.5483 | 0 | 0 | 0.1139 | 0.033 | 0.0026 | 0 | 0.0368 | 0 | 0 | 0 | 0 |
| normal_tissue | German_2023 | 0.1528 | 0 | 0.9913 | 0.0007 | 0.023 | 0.0162 | 0.0014 | 0.094 | 0 | 0.4571 | 0 | 0.0345 | 0 | 0 |
| normal_tissue | German_2023 | 0.0005 | 0 | 0.6606 | 0 | 0.0025 | 0.0797 | 0 | 0.0657 | 0 | 0.0229 | 0 | 0 | 0 | 0 |
| normal_tissue | German_2023 | 0.0938 | 0 | 0.8486 | 0 | 0.0118 | 0.0032 | 0.0007 | 0.0399 | 0 | 0.0282 | 0 | 0 | 0 | 0 |
| normal_tissue | German_2023 | 0.7159 | 0 | 2.7791 | 0 | 0 | 0.0784 | 0.293 | 0 | 0 | 0.1405 | 0 | 0 | 0 | 0 |
| normal_tissue | German_2023 | 1.5455 | 0.1118 | 0.7142 | 0 | 0 | 0.1091 | 0.837 | 0.0008 | 0.9607 | 0.0008 | 0.0954 | 0 | 0 | 0 |
| normal_tissue | German_2023 | 0.3173 | 0.0256 | 0.6085 | 0.0102 | 0 | 0.152 | 0.2071 | 0.0162 | 0.3685 | 0.0769 | 0 | 0 | 0 | 0 |
| normal_tissue | German_2023 | 0.0384 | 0.0088 | 0.7772 | 0.2706 | 0 | 3.258 | 0.4097 | 0.0717 | 0 | 0.4292 | 0 | 0 | 0 | 0 |
| normal_tissue | German_2023 | 0.1189 | 0 | 0.8796 | 0.0248 | 0.032 | 0.062 | 0.0176 | 0.0021 | 0.001 | 0.0041 | 0 | 0.0269 | 0 | 0 |
| normal_tissue | German_2023 | 0.7123 | 0.0008 | 1.0942 | 0.0008 | 0.0608 | 0.1859 | 0.6422 | 23.4828 | 0.0034 | 0.5526 | 0 | 0 | 0 | 0 |
| normal_tissue | German_2023 | 0.0862 | 0.144 | 0.6286 | 0 | 0.0002 | 0.0377 | 0.002 | 0.0002 | 0 | 0.0117 | 0 | 0 | 0 | 0 |
| normal_tissue | German_2023 | 1.2763 | 1.6929 | 0.2223 | 0.0563 | 0.2718 | 0.0325 | 0.3873 | 0.0273 | 0.0569 | 0.0026 | 0 | 0.0228 | 0 | 0.2966 |
| normal_tissue | German_2023 | 0.7353 | 0.2713 | 0.0516 | 0.0458 | 0 | 2.0325 | 0.0026 | 0.1552 | 0 | 0.175 | 0 | 0 | 0 | 0 |
| normal_tissue | German_2023 | 0.0692 | 0.0184 | 0.3307 | 0.0004 | 0.0054 | 0.021 | 0.3943 | 0.011 | 0.1469 | 0.0299 | 0 | 0 | 0 | 0 |
| normal_tissue | German_2023 | 0.2522 | 0 | 0.3169 | 0 | 0 | 0.0011 | 0.016 | 0.044 | 0 | 0.4193 | 0 | 0 | 0 | 0 |
| normal_tissue | German_2023 | 0.0973 | 0.0002 | 0.2264 | 0.0208 | 0.04 | 0.2006 | 0 | 0.0408 | 0.0266 | 0.1233 | 0 | 0 | 0 | 0 |
| normal_tissue | German_2023 | 0.3776 | 0.0631 | 0.5926 | 0 | 0.0374 | 0.1307 | 0.1098 | 0 | 0 | 0.0128 | 0 | 0.0355 | 0 | 0 |
| normal_tissue | German_2023 | 0.0717 | 0 | 0.4764 | 0 | 0.007 | 0.0279 | 0.0319 | 0.0002 | 0 | 0.0184 | 0 | 0 | 0 | 0 |
| normal_tissue | German_2023 | 3.3313 | 0.1701 | 1.1344 | 0 | 0.366 | 0.3362 | 1.4013 | 0.1255 | 0 | 0.2427 | 0 | 0.5216 | 0 | 0 |
| normal_tissue | German_2023 | 0.1431 | 0 | 1.2271 | 0 | 0.0885 | 0.2393 | 0.0008 | 0.0542 | 0 | 0.0658 | 0 | 0 | 0 | 0 |
| normal_tissue | German_2023 | 0.3545 | 0.0739 | 0.7833 | 0.0238 | 0.0556 | 0 | 0.1767 | 0.0718 | 0.0861 | 0.0561 | 0 | 0 | 0 | 0 |
| normal_tissue | German_2023 | 0.0667 | 0.0113 | 0.511 | 0.0385 | 0.0623 | 0.6851 | 0 | 0.0066 | 0 | 0.0429 | 0 | 0 | 0 | 0 |
| normal_tissue | German_2023 | 2.3985 | 0.0792 | 4.8603 | 0.0079 | 0.0079 | 0.0237 | 1.124 | 1.8127 | 0.0317 | 2.723 | 0 | 0 | 0 | 0.0317 |
| normal_tissue | German_2023 | 0.0664 | 0 | 0.3804 | 0.0053 | 0 | 0.2218 | 0 | 0.05 | 0 | 0.0343 | 0 | 0 | 0 | 0 |
| normal_tissue | German_2023 | 0.4737 | 0 | 2.5306 | 0 | 0 | 0.1826 | 0.0014 | 0.7757 | 0.0003 | 0.0064 | 0.0007 | 0 | 0 | 0 |
| normal_tissue | German_2023 | 0.1434 | 0 | 1.4651 | 0 | 0 | 0.0126 | 0.709 | 0.0688 | 0 | 0.0187 | 0.0003 | 0 | 0 | 0 |
| normal_tissue | German_2023 | 0.047 | 0.0004 | 0.5883 | 0 | 0 | 0.0838 | 0.0446 | 0.0203 | 0 | 0.0045 | 0 | 0 | 0 | 0.0006 |
| normal_tissue | German_2023 | 12.8971 | 0.0017 | 0.0514 | 1.245 | 0 | 0.0034 | 8.1893 | 0.0017 | 0 | 0.0137 | 0 | 0 | 0 | 0.0017 |
| normal_tissue | German_2023 | 0.6245 | 0 | 1.4865 | 0.0003 | 0 | 0 | 0 | 0.0008 | 0 | 0.0807 | 0.4674 | 0 | 0 | 0.0003 |
| normal_tissue | German_2023 | 0.0232 | 4.1341 | 0 | 0.0033 | 0 | 12.2466 | 7.1518 | 1.6066 | 0.0066 | 2.3056 | 0 | 0 | 0 | 0.0629 |
| normal_tissue | German_2023 | 4.9889 | 0.003 | 4.8999 | 0 | 0 | 0.0089 | 5.1223 | 4.8969 | 0.003 | 6.4393 | 0.0059 | 0 | 0 | 0 |
| normal_tissue | German_2023 | 1.4011 | 0.4296 | 3.4257 | 0 | 0.2255 | 0.3521 | 0.0003 | 0.007 | 0.0003 | 0.2623 | 0 | 0 | 0 | 0 |
| normal_tissue | German_2023 | 1.1712 | 0.0003 | 2.2063 | 0.0003 | 0 | 0.0006 | 0.6366 | 0.0637 | 0.149 | 0.6371 | 0 | 0.0538 | 0 | 0 |
| normal_tissue | German_2023 | 0.1503 | 0.0199 | 0.7167 | 0.0389 | 0.0876 | 0.0866 | 0.0015 | 0.0686 | 0 | 0.0681 | 0 | 0 | 0 | 0 |
| normal_tissue | German_2023 | 0.4628 | 0.003 | 0 | 0 | 1.1457 | 0.0769 | 0.0045 | 1.007 | 0 | 0.799 | 0 | 0 | 0 | 0 |
| normal_tissue | German_2023 | 0.1268 | 0.0007 | 15.4124 | 0 | 0 | 0.2147 | 0.0007 | 1.8194 | 0 | 0.9348 | 0 | 0 | 0 | 0 |
| normal_tissue | German_2023 | 0.0003 | 0.1116 | 2.4427 | 0 | 0 | 0.1874 | 0 | 4.7525 | 0 | 0.0454 | 0.0003 | 0 | 0 | 0.0003 |
| normal_tissue | German_2023 | 0.0151 | 0.0041 | 0.1369 | 0 | 0.0055 | 0.021 | 0 | 0.085 | 0.0069 | 0.0014 | 0 | 0 | 0 | 0 |
| normal_tissue | German_2023 | 0.1663 | 0 | 0.7065 | 0 | 0.0003 | 0.002 | 0.0227 | 0.092 | 0.0335 | 0.1147 | 0.0005 | 0 | 0 | 0 |
| normal_tissue | German_2023 | 0.1349 | 0 | 0.3754 | 0 | 0 | 0.0021 | 0.0415 | 0.0008 | 0 | 0.0152 | 0.0003 | 0.0165 | 0 | 0 |
| normal_tissue | German_2023 | 1.5488 | 0.1877 | 3.4477 | 0 | 0.0271 | 0.0009 | 0.6232 | 0.5658 | 0 | 0.1191 | 0.0003 | 0 | 0 | 0 |
| normal_tissue | German_2023 | 4.6163 | 0.5632 | 0.0005 | 0.0011 | 0.0016 | 17.2718 | 1.4056 | 0.0421 | 1.1001 | 0.0479 | 1.9025 | 0 | 0 | 0.0016 |
| normal_tissue | German_2023 | 0.0502 | 0.0006 | 0.9518 | 0.0209 | 0.0017 | 0.0344 | 0.0631 | 0.0417 | 0 | 0.0997 | 0 | 0 | 0 | 0 |
| normal_tissue | German_2023 | 0.8736 | 0 | 0.5828 | 0 | 0 | 0.0523 | 0.0214 | 0.0255 | 0.0013 | 0.6967 | 0.1233 | 0 | 0 | 0 |
| normal_tissue | German_2023 | 4.0327 | 0.1534 | 5.2367 | 0.3093 | 0 | 0.805 | 1.8252 | 52.3426 | 0.001 | 0.0098 | 0 | 0 | 0 | 0.0005 |
| normal_tissue | German_2023 | 0.244 | 0 | 4.0131 | 0 | 0 | 0.0036 | 0 | 0.6373 | 0 | 2.4873 | 0 | 0 | 0 | 0 |
| normal_tissue | German_2023 | 1.5915 | 0.0639 | 4.1938 | 0 | 0 | 0 | 0.1743 | 0.0058 | 0.1568 | 1.4521 | 0 | 0 | 0 | 0 |
| normal_tissue | German_2023 | 2.1633 | 0.637 | 4.3226 | 0 | 0 | 0 | 1.7547 | 0.004 | 0 | 0.2163 | 0 | 0 | 0 | 0 |
| normal_tissue | German_2023 | 0.0941 | 0.0328 | 0.4915 | 0.0022 | 0.0016 | 0.0525 | 0 | 0.0263 | 0 | 0.0695 | 0 | 0 | 0 | 0 |
| normal_tissue | German_2023 | 0.3619 | 0 | 12.3484 | 0 | 0 | 1.0603 | 0.077 | 1.2847 | 0 | 0.0887 | 0 | 0 | 0 | 0 |
| normal_tissue | German_2023 | 6.462 | 0.8712 | 7.8829 | 0 | 0.1184 | 0 | 6.9526 | 0 | 0.0085 | 0.3383 | 0 | 0 | 0 | 0 |
| normal_tissue | German_2023 | 0.1127 | 0.0005 | 0.8554 | 0.0272 | 0.0652 | 0.0272 | 0.0684 | 0.0214 | 0 | 0.0796 | 0.0775 | 0 | 0 | 0 |
| normal_tissue | German_2023 | 1.4797 | 0.3523 | 4.8266 | 0 | 0 | 0.0783 | 1.5032 | 0.0078 | 0 | 12.1076 | 0 | 0 | 0 | 0 |
| normal_tissue | German_2023 | 0.6376 | 0.2696 | 1.8586 | 0 | 0.16 | 0.0045 | 0.0006 | 77.0174 | 0.0017 | 0.0831 | 0 | 0 | 0 | 0 |
| normal_tissue | German_2023 | 1.749 | 1.1423 | 2.4244 | 0 | 0.0053 | 0.3415 | 1.1005 | 24.1098 | 0.0005 | 0.1374 | 0.0005 | 0 | 0 | 0 |
| normal_tissue | German_2023 | 3.3907 | 0.8974 | 4.7829 | 0 | 0.1695 | 0.3613 | 2.499 | 20.4206 | 0.4423 | 0.3381 | 0 | 0.0004 | 0 | 0 |
| normal_tissue | German_2023 | 5.3764 | 0.5617 | 3.6583 | 0.1176 | 0.0017 | 0.9875 | 4.4701 | 5.6829 | 1.4067 | 2.2616 | 0.1243 | 0 | 0 | 0.0017 |
| normal_tissue | German_2023 | 0.0691 | 0.048 | 1.3301 | 0.061 | 0.0374 | 0.1786 | 0.001 | 0.1118 | 0 | 0.0096 | 0 | 0 | 0 | 0 |
| normal_tissue | German_2023 | 2.3747 | 0 | 14.2397 | 0 | 0 | 0.0043 | 0 | 0.4306 | 0 | 1.37 | 0 | 0 | 0 | 0 |
| normal_tissue | German_2023 | 6.7645 | 1.4085 | 1.977 | 0.6507 | 0.3717 | 3.856 | 2.5605 | 10.772 | 2.7457 | 0.0326 | 2.2147 | 0 | 0 | 0.7221 |
| normal_tissue | German_2023 | 0.0888 | 0.0221 | 0.307 | 0.0656 | 0.0596 | 0.1741 | 0.0006 | 0.0805 | 0 | 0.2504 | 0 | 0 | 0 | 0 |
| normal_tissue | German_2023 | 0.1421 | 0 | 1.382 | 0.1667 | 0 | 0 | 0.0005 | 0.1466 | 0.001 | 0.0152 | 0 | 0 | 0 | 0 |
| normal_tissue | German_2023 | 3.1848 | 9.6457 | 20.2334 | 0 | 0 | 0.8527 | 0.002 | 2.4559 | 0 | 1.1739 | 0 | 0 | 0 | 0 |
| normal_tissue | German_2023 | 9.5544 | 0.3607 | 9.9902 | 0 | 0.2066 | 0.7928 | 0.8491 | 1.5254 | 0 | 0.2893 | 0 | 0 | 0 | 0 |
| normal_tissue | German_2023 | 2.0248 | 0.0003 | 11.7023 | 0 | 0 | 0.6835 | 0.017 | 0.4449 | 0 | 0.7505 | 0 | 0 | 0 | 0 |
| normal_tissue | German_2023 | 5.2359 | 8.6699 | 2.2751 | 0.1771 | 0.2459 | 1.866 | 1.4807 | 7.6961 | 1.4321 | 0.3084 | 0 | 0.0069 | 0 | 0 |
| normal_tissue | German_2023 | 0.1701 | 0.0033 | 5.2886 | 0 | 0.4335 | 0.1601 | 0.2934 | 0.1534 | 0 | 0.0834 | 0 | 0 | 0 | 0 |
| normal_tissue | German_2023 | 3.3029 | 0 | 3.5869 | 0 | 0 | 0.7683 | 0.38 | 0.0056 | 0 | 0.2032 | 0 | 0 | 0 | 0 |
| normal_tissue | German_2023 | 0.0785 | 0 | 0.6541 | 0.0186 | 0.0574 | 0.1766 | 0 | 0.072 | 0 | 0.0629 | 0 | 0 | 0 | 0 |
| normal_tissue | German_2023 | 0.1432 | 0.2785 | 1.8729 | 0.1621 | 0.0502 | 0.0718 | 1.0344 | 0.0193 | 0 | 0.0132 | 0 | 0 | 0 | 0 |
| normal_tissue | German_2023 | 0.1782 | 0 | 4.9685 | 0 | 0 | 0.0014 | 0.1206 | 0 | 0 | 0.4359 | 0 | 0 | 0 | 0.2823 |
| normal_tissue | German_2023 | 0.2757 | 0.0391 | 0.3603 | 0.0273 | 0.0082 | 0.0321 | 0.0621 | 0.1297 | 0 | 0.0164 | 0 | 0 | 0 | 0 |
| normal_tissue | German_2023 | 12.523 | 1.7633 | 7.7155 | 0.3844 | 1.8399 | 5.8779 | 9.3654 | 0.0421 | 0.6462 | 0.392 | 1.003 | 0.2419 | 0 | 8.5209 |
| normal_tissue | German_2023 | 0.4108 | 0.1524 | 1.3209 | 0 | 0 | 0.5199 | 0.8269 | 0 | 0.2737 | 0.2194 | 0 | 0 | 0 | 0 |
| normal_tissue | German_2023 | 0.0526 | 0.0889 | 1.0616 | 0 | 0 | 0.1774 | 0.0986 | 0.0704 | 0 | 0.0383 | 0 | 0 | 0 | 0 |
| normal_tissue | German_2023 | 0.0939 | 0.0638 | 0.614 | 0 | 0.0152 | 0.001 | 0.0225 | 0.0112 | 0 | 0.2235 | 0 | 0 | 0 | 0 |
| normal_tissue | German_2023 | 0.522 | 0.0018 | 2.7124 | 0 | 0.0009 | 0 | 0.0044 | 0.0018 | 0 | 0.9074 | 0 | 0 | 0 | 0 |
| normal_tissue | German_2023 | 4.6425 | 0 | 1.1376 | 0 | 0 | 0 | 0.1623 | 0.107 | 0.003 | 0.1217 | 0.0689 | 0 | 0 | 0 |
| normal_tissue | German_2023 | 0.1769 | 0.1123 | 0.5923 | 0 | 0.0057 | 0.0353 | 0.0363 | 0.0209 | 0.0003 | 0.0901 | 0 | 0 | 0 | 0 |
| normal_tissue | German_2023 | 0.1147 | 0 | 0.8065 | 0 | 0.0004 | 0 | 0.0023 | 0.0975 | 0.0008 | 0.046 | 0 | 0 | 0 | 0 |
| normal_tissue | German_2023 | 0.199 | 0 | 0.6311 | 0 | 0 | 0.0004 | 0.1234 | 0.0126 | 0.0594 | 0.0669 | 0 | 0.0072 | 0 | 0 |
| normal_tissue | German_2023 | 0.1633 | 0.0485 | 1.188 | 0 | 0 | 0.0003 | 0.0017 | 0.0706 | 0 | 0.0098 | 0 | 0 | 0 | 0 |
| normal_tissue | German_2023 | 0.0452 | 0.01 | 0.3922 | 0 | 0 | 0.0197 | 0.076 | 0.0013 | 0.0007 | 0.0599 | 0 | 0 | 0 | 0 |
| normal_tissue | German_2023 | 2.5581 | 0.0322 | 0.2896 | 0.1233 | 0.0876 | 1.557 | 6.7578 | 0.4993 | 0.4123 | 0.0477 | 0.9719 | 0.0006 | 0 | 0.3587 |
| normal_tissue | German_2023 | 0.2793 | 0.1064 | 1.3669 | 0 | 0 | 0.16 | 0.4494 | 0 | 0 | 0.0637 | 0 | 0 | 0 | 0 |
| normal_tissue | German_2023 | 0.1323 | 0 | 0.5372 | 0 | 0 | 0.0003 | 0.0209 | 0.0007 | 0.0003 | 0.0771 | 0 | 0.0199 | 0 | 0 |
| normal_tissue | German_2023 | 0.4308 | 0.1344 | 1.3309 | 0.0184 | 0 | 0.0018 | 0.0202 | 0.1086 | 0 | 0.8873 | 0 | 0.0018 | 0 | 0 |
| normal_tissue | German_2023 | 1.312 | 0.1256 | 2.1207 | 0 | 0 | 0.0016 | 0 | 0.0822 | 0 | 0.385 | 0 | 0.0145 | 0 | 0 |
| normal_tissue | German_2023 | 0.2247 | 0.0005 | 0.2692 | 0.0196 | 0.0117 | 0.0592 | 0.0117 | 0.0426 | 0 | 0.0646 | 0 | 0 | 0 | 0 |
| normal_tissue | German_2023 | 0.575 | 0 | 2.7423 | 0 | 0 | 0.0014 | 0.1133 | 0.0294 | 0 | 1.0297 | 0 | 0 | 0 | 0 |
| normal_tissue | German_2023 | 0.9863 | 0.031 | 1.0468 | 0 | 0 | 1.252 | 0 | 0 | 0 | 0.0722 | 0 | 0 | 0 | 0 |
| normal_tissue | German_2023 | 0.9857 | 0.6009 | 0.0007 | 0 | 0 | 0.002 | 1.7172 | 0.0054 | 0.0007 | 0.0455 | 0.0569 | 2.7317 | 0 | 0 |
| normal_tissue | German_2023 | 0.8208 | 0 | 3.1964 | 0 | 0.0968 | 0.1215 | 0.6128 | 0.1352 | 0.0768 | 0.0144 | 0 | 0 | 0 | 0 |
| normal_tissue | German_2023 | 3.0938 | 0 | 0 | 0 | 0 | 0.8003 | 7.3994 | 2.1225 | 12.8351 | 0.3888 | 0 | 1.3661 | 0 | 1.0439 |
| normal_tissue | German_2023 | 0.8988 | 0.0239 | 1.1029 | 0 | 0 | 0 | 0 | 0.1779 | 0.0639 | 0.0752 | 0 | 0 | 0 | 0 |
| normal_tissue | German_2023 | 10.8394 | 0.0148 | 5.2518 | 0 | 0.5305 | 0.8296 | 4.2978 | 0.5091 | 0 | 0.7532 | 0.2915 | 0.0581 | 0 | 0.0005 |
| normal_tissue | German_2023 | 0.2547 | 0.2052 | 0.0038 | 0.9198 | 0 | 1.718 | 0.0038 | 0.1862 | 0 | 3.4474 | 1.9232 | 0.5625 | 0 | 0.8818 |
| normal_tissue | German_2023 | 2.2176 | 0.0272 | 2.6999 | 0 | 0 | 1.1818 | 0.0509 | 0 | 0.0136 | 2.3127 | 0 | 0 | 0 | 0 |
| normal_tissue | German_2023 | 1.077 | 0.0003 | 2.5135 | 0.0003 | 0 | 0 | 0 | 0.1411 | 0 | 0.0921 | 0 | 0 | 0 | 0 |
| normal_tissue | German_2023 | 0.0485 | 0.0253 | 1.8887 | 0 | 0 | 0.0985 | 0.0844 | 0.1094 | 0 | 0.0413 | 0 | 0 | 0 | 0 |
| normal_tissue | German_2023 | 0.2193 | 0 | 1.2579 | 0 | 0 | 0 | 0.447 | 0.0931 | 0 | 0.1291 | 0 | 0 | 0 | 0 |
| normal_tissue | German_2023 | 2.0956 | 3.7824 | 17.5094 | 0 | 0.6943 | 0.2239 | 0 | 0.1899 | 0 | 0.512 | 0 | 0 | 0 | 0 |
| normal_tissue | German_2023 | 1.5115 | 0 | 20.3362 | 0 | 0.001 | 0.4584 | 0.001 | 1.0511 | 0 | 0.2538 | 0 | 1.3109 | 0 | 0 |
| normal_tissue | German_2023 | 0.7268 | 0.3697 | 5.7382 | 0.2925 | 0 | 0.3014 | 0.2361 | 0.0011 | 0 | 0.0535 | 0 | 0 | 0 | 0 |
| normal_tissue | German_2023 | 0.2776 | 0.0005 | 0.7433 | 0 | 0 | 0.0256 | 0.0587 | 0.001 | 0.0572 | 0.2083 | 0 | 0 | 0 | 0 |
| normal_tissue | German_2023 | 0.0222 | 0.0392 | 1.2621 | 0 | 0 | 0.1398 | 0.1549 | 0.1001 | 0.0099 | 0.1766 | 0 | 0.0316 | 0 | 0 |
| normal_tissue | German_2023 | 0.2111 | 0 | 1.4008 | 0 | 0.0947 | 0.7047 | 0.0801 | 0.1061 | 0 | 0.02 | 0 | 0 | 0 | 0 |
| normal_tissue | German_2023 | 0.0028 | 0 | 0.6913 | 0 | 0 | 0.0114 | 0.1356 | 0.001 | 0 | 0.0339 | 0 | 0 | 0 | 0 |
| normal_tissue | German_2023 | 0.0469 | 0.1261 | 0.914 | 0.0003 | 0 | 0.0003 | 0.1189 | 0.0266 | 0 | 0.0315 | 0 | 0 | 0 | 0 |
| normal_tissue | German_2023 | 0.5237 | 0.101 | 2.4108 | 0.0027 | 0 | 0.1181 | 0 | 0.1508 | 0 | 0.0201 | 0 | 0 | 0 | 0 |
| normal_tissue | German_2023 | 0.1735 | 0 | 1.1823 | 0 | 0.0169 | 0.0004 | 0.0777 | 0.1142 | 0 | 0.1543 | 0 | 0 | 0 | 0 |
| normal_tissue | German_2023 | 1.2999 | 0 | 1.9911 | 0 | 0 | 0 | 0 | 0.0338 | 0.0534 | 0.0883 | 0 | 0 | 0 | 0 |
| normal_tissue | German_2023 | 0.0671 | 0.034 | 0.3029 | 0 | 0.0122 | 0.0833 | 0 | 0.001 | 0 | 0.1159 | 0 | 0 | 0 | 0.0005 |
| normal_tissue | German_2023 | 7.6772 | 0 | 0 | 0 | 0 | 0 | 0.6496 | 0.6592 | 0.0007 | 0.0575 | 0 | 0 | 0 | 0 |
| normal_tissue | German_2023 | 0.9614 | 0.1944 | 2.3987 | 0 | 0 | 0.0785 | 0 | 0.0929 | 0 | 0.0821 | 0 | 0 | 0 | 0 |
| normal_tissue | German_2023 | 0.0582 | 0.0179 | 0.8478 | 0 | 0 | 0 | 0 | 0.0081 | 0 | 0.042 | 0 | 0 | 0 | 0 |
| normal_tissue | German_2023 | 0.2304 | 0 | 1.1929 | 0 | 0 | 0.1162 | 0 | 0 | 0 | 0.0188 | 0 | 0 | 0 | 0 |
| normal_tissue | German_2023 | 3.6801 | 0.1984 | 1.6838 | 0.0057 | 0.3543 | 0.507 | 1.7656 | 0.0672 | 0.0932 | 0.187 | 0 | 0.0653 | 0 | 0 |
| normal_tissue | German_2023 | 0.0141 | 0.0402 | 0.9494 | 0 | 0.0004 | 0.1078 | 0.0987 | 0.1625 | 0.0551 | 0.085 | 0 | 0 | 0 | 0 |
| normal_tissue | German_2023 | 0.3235 | 0 | 1.2124 | 0 | 0.0578 | 0 | 0.0258 | 0.0571 | 0 | 0.0506 | 0 | 0 | 0 | 0 |
| normal_tissue | German_2023 | 10.281 | 0.2204 | 18.0967 | 0 | 0.1043 | 0.0012 | 0 | 1.7233 | 0 | 0.694 | 0 | 0 | 0 | 0 |
| normal_tissue | German_2023 | 0.0413 | 0 | 0.5542 | 0 | 0.0003 | 0.0382 | 0.0361 | 0.0502 | 0 | 0.0218 | 0 | 0 | 0 | 0 |
| normal_tissue | German_2023 | 8.8291 | 0.1576 | 0.8313 | 0.1213 | 0 | 1.3836 | 1.7574 | 0 | 0.272 | 0.2678 | 1.1577 | 0 | 0 | 0 |
| normal_tissue | German_2023 | 0.0536 | 0 | 0.767 | 0 | 0.0005 | 0.0836 | 0 | 0 | 0 | 0.2487 | 0 | 0 | 0 | 0 |
| normal_tissue | German_2023 | 0.1886 | 0 | 14.3718 | 0 | 0.1576 | 0.0028 | 0 | 0.2421 | 0 | 1.4003 | 0 | 0 | 0 | 0 |
| normal_tissue | German_2023 | 0.0636 | 0 | 0.4366 | 0 | 0.0038 | 0.0002 | 0 | 0.1307 | 0.054 | 0.0056 | 0 | 0 | 0 | 0 |
| normal_tissue | German_2023 | 0.1065 | 0.0112 | 0.441 | 0 | 0 | 0.0561 | 0 | 0.0282 | 0 | 0.0252 | 0 | 0 | 0 | 0 |
| normal_tissue | German_2023 | 0.3021 | 0.0006 | 2.0498 | 0 | 0.2687 | 0.3344 | 0.0003 | 0.0331 | 0 | 0.0725 | 0 | 0.1528 | 0 | 0 |
| normal_tissue | German_2023 | 32.7876 | 2.179 | 2.8789 | 0.2762 | 0.0011 | 1.519 | 11.1077 | 0.273 | 0.6747 | 1.3514 | 0.155 | 0 | 0 | 0.0011 |
| normal_tissue | German_2023 | 2.2162 | 0 | 3.2991 | 0.3337 | 0 | 0.6485 | 1.0577 | 0.5918 | 0 | 3.9791 | 0 | 0 | 0 | 0 |
| normal_tissue | German_2023 | 0.4075 | 0.0156 | 0.9575 | 0 | 0 | 0.1582 | 0.4972 | 1.0681 | 0 | 0.1187 | 0 | 0 | 0 | 0 |
| normal_tissue | German_2023 | 52.2399 | 0.3603 | 7.5358 | 0.0006 | 0 | 0.5589 | 0 | 0.0079 | 0 | 0.0547 | 0 | 0 | 0 | 0 |
| normal_tissue | German_2023 | 1.8506 | 0.0004 | 0.1415 | 0 | 0 | 0.0047 | 0.0082 | 0.0145 | 0 | 0.007 | 0 | 0.008 | 0 | 0 |
| normal_tissue | German_2023 | 0.2765 | 0 | 0.6013 | 0 | 0 | 0.017 | 0.116 | 0.0147 | 0 | 0.0221 | 0 | 0 | 0 | 0 |
| normal_tissue | German_2023 | 0.0072 | 0 | 0.5004 | 0 | 0.0085 | 0.0075 | 0 | 0.0134 | 0.0003 | 0.347 | 0 | 0 | 0 | 0 |
| normal_tissue | German_2023 | 0.3087 | 0.0223 | 0.7765 | 0 | 0 | 0.0397 | 0.1707 | 0.0213 | 0 | 0.0928 | 0.0006 | 0.0124 | 0 | 0 |
| normal_tissue | German_2023 | 0.0011 | 0 | 1.0278 | 0 | 0 | 0.0512 | 0.0005 | 0.0827 | 0.0002 | 0.0444 | 0 | 0.0483 | 0 | 0 |
| normal_tissue | German_2023 | 3.4667 | 0.2371 | 0.2802 | 0.0472 | 0 | 3.1299 | 0.7774 | 0.2034 | 0 | 0.3665 | 0 | 0.0027 | 0 | 0 |
| normal_tissue | German_2023 | 0.59 | 0.0283 | 3.4372 | 0 | 0 | 0.1134 | 2.9038 | 0.0103 | 0.1185 | 0.2937 | 0 | 0 | 0 | 0 |
| normal_tissue | German_2023 | 0.8773 | 0.2052 | 0.4925 | 0 | 0 | 0.0051 | 0.4618 | 0.4669 | 0 | 2.2061 | 0.0051 | 0.0103 | 0 | 0.3591 |
| normal_tissue | German_2023 | 0.0785 | 0.015 | 0.9248 | 0 | 0.0005 | 0.0542 | 0 | 0.0826 | 0 | 0.0475 | 0 | 0 | 0 | 0.1363 |
| normal_tissue | German_2023 | 0.6821 | 0.0003 | 1.034 | 0.0003 | 0.0746 | 0.1033 | 0.6076 | 0.0305 | 0 | 1.2241 | 0 | 0 | 0 | 0 |
| normal_tissue | German_2023 | 0.9898 | 0.5309 | 4.2546 | 0.3572 | 0.0012 | 0.5634 | 1.639 | 0.3959 | 0.297 | 0.1095 | 0 | 0 | 0 | 0.0005 |
| normal_tissue | German_2023 | 0.6398 | 0 | 2.7995 | 0 | 0 | 0.4377 | 0.0005 | 0.3749 | 0 | 0.1865 | 0 | 0.0713 | 0 | 0 |
| normal_tissue | German_2023 | 0.0708 | 0.0111 | 0.2079 | 0 | 0 | 0.0008 | 0 | 0.0004 | 0 | 0.0423 | 0 | 0.0091 | 0 | 0 |
| normal_tissue | German_2023 | 0.665 | 0.1205 | 0.4862 | 0.019 | 0.0163 | 0.1395 | 0.0786 | 0.0637 | 0 | 0.2424 | 0.044 | 0.0318 | 0 | 0.1077 |
| normal_tissue | German_2023 | 0.2918 | 0.0195 | 0.4486 | 0 | 0.0644 | 0.1378 | 0.0008 | 0 | 0 | 0.109 | 0 | 0 | 0 | 0 |
| normal_tissue | German_2023 | 0.6227 | 0.192 | 0 | 0 | 0.0393 | 0.742 | 0.0053 | 1.4254 | 0.0027 | 0.28 | 0 | 0.0007 | 0 | 0 |
| normal_tissue | German_2023 | 0.0987 | 0.0385 | 0.1945 | 0 | 0.0231 | 0.0407 | 0.0308 | 0.0055 | 0 | 0.0191 | 0.0301 | 0 | 0 | 0 |
| normal_tissue | German_2023 | 0.0616 | 0.0279 | 1.0303 | 0 | 0 | 0.0286 | 0.0476 | 0.1384 | 0 | 0.4323 | 0 | 0 | 0 | 0 |
| normal_tissue | German_2023 | 0.1521 | 0.043 | 1.0912 | 0 | 0 | 0.0423 | 0.2062 | 0.0752 | 0 | 0.0509 | 0 | 0.0405 | 0 | 0 |
| normal_tissue | German_2023 | 42.7573 | 0.6256 | 0.004 | 0.0013 | 0.0013 | 2.1499 | 0.647 | 0.3161 | 0.0013 | 0.4487 | 0.8868 | 0 | 0 | 0.0027 |
| normal_tissue | German_2023 | 0.1545 | 0.0003 | 0.8011 | 0.0003 | 0.0213 | 0.0048 | 0.0625 | 0 | 0 | 0.0869 | 0 | 0.0092 | 0 | 0 |
| normal_tissue | German_2023 | 0.1438 | 0.0153 | 0.555 | 0.0988 | 0 | 2.1944 | 0.0003 | 0.0446 | 0 | 0.0218 | 0.1438 | 0 | 0 | 0 |
| normal_tissue | German_2023 | 0.2675 | 0.2959 | 0.928 | 0.0892 | 0.0955 | 0.0934 | 0.0923 | 0.0005 | 0.0005 | 0.15 | 0.0325 | 0 | 0 | 0 |
| normal_tissue | German_2023 | 0.9037 | 0.3086 | 2.7671 | 0 | 0 | 0.0631 | 0.2064 | 0.1884 | 0 | 0.1533 | 0 | 0.015 | 0 | 0 |
| normal_tissue | German_2023 | 1.0244 | 0.216 | 0.0441 | 0.0023 | 0.0023 | 0.8827 | 0.6829 | 0.4483 | 0 | 1.0801 | 0 | 0 | 0 | 0 |
| normal_tissue | German_2023 | 1.6679 | 0.0553 | 1.3875 | 0 | 0 | 0.1073 | 0.2901 | 0.0135 | 0.0849 | 0.0194 | 0 | 0.0494 | 0 | 0 |
| normal_tissue | German_2023 | 0.2239 | 0.0882 | 1.0303 | 0 | 0.0254 | 0.0148 | 0.117 | 0.0449 | 0 | 0.0449 | 0.0004 | 0 | 0 | 0 |
| normal_tissue | German_2023 | 2.9854 | 1.7069 | 0.0022 | 0.0011 | 0.7329 | 0.0097 | 0.0011 | 3.0349 | 0 | 0.2497 | 0 | 0 | 0 | 0 |
| normal_tissue | German_2023 | 0.8265 | 0 | 1.2247 | 0 | 0.0322 | 0.1508 | 0 | 0.098 | 0 | 0.0067 | 0 | 0 | 0 | 0 |
| normal_tissue | German_2023 | 0.2775 | 0.0448 | 1.2384 | 0 | 0 | 0.0003 | 1.6077 | 0.0569 | 0 | 0.2424 | 0 | 0 | 0 | 0 |
| normal_tissue | German_2023 | 0.3106 | 0 | 0.5399 | 0 | 0.0284 | 0.1221 | 0.0973 | 0.0118 | 0.0003 | 0.0024 | 0 | 0.0323 | 0 | 0 |
| normal_tissue | German_2023 | 0.3646 | 0.1417 | 0.7199 | 0.0463 | 0.0684 | 0.1025 | 0.0648 | 0.0933 | 0 | 0.6422 | 0.0427 | 0.0662 | 0 | 0 |
| normal_tissue | German_2023 | 1.8164 | 0.2026 | 3.3242 | 0 | 0 | 0.0006 | 0.3055 | 0.0546 | 0 | 0.1169 | 0 | 0 | 0 | 0 |
| normal_tissue | German_2023 | 3.4562 | 0.1848 | 0.8606 | 0.0414 | 1.033 | 1.1102 | 0.6454 | 0.08 | 0.3282 | 0.4868 | 0 | 0.0303 | 0 | 0.051 |
| normal_tissue | German_2023 | 0.2504 | 0.0218 | 0.8724 | 0.0765 | 0 | 0.4183 | 0.2474 | 0.0705 | 0 | 0.1209 | 0 | 0 | 0 | 0 |
| normal_tissue | German_2023 | 0.0935 | 0.0819 | 1.5698 | 0 | 0 | 0.0277 | 0.0025 | 0 | 0 | 0.0244 | 0.0004 | 0 | 0 | 0 |
| normal_tissue | German_2023 | 0.5785 | 0 | 12.0505 | 0 | 0 | 0.0023 | 0.0011 | 0.3416 | 0 | 0.3132 | 0 | 0 | 0 | 0 |
| BC_tissue | German_2023 | 2.5075 | 0.0007 | 18.7324 | 0 | 0.0007 | 0.0087 | 0.0036 | 0.1742 | 0 | 0.4779 | 0 | 0 | 0 | 0 |
| BC_tissue | German_2023 | 0.8193 | 0.0053 | 6.3327 | 0 | 0 | 0.411 | 0.9154 | 0.5311 | 0 | 13.41 | 0 | 0 | 0 | 0 |
| BC_tissue | German_2023 | 0.0303 | 0 | 2.4619 | 0 | 0.0706 | 0.0908 | 0 | 0.7668 | 0 | 10.6548 | 0.4843 | 0 | 0.0101 | 0.3733 |
| BC_tissue | German_2023 | 1.0872 | 0 | 5.0867 | 0 | 0 | 0.0568 | 0.0699 | 0.5458 | 0.0044 | 5.0081 | 0 | 0 | 0 | 0 |
| BC_tissue | German_2023 | 1.1488 | 0.0436 | 0.7654 | 0.0286 | 0.0387 | 0.1298 | 0.5064 | 0.2784 | 0.0026 | 0.1122 | 0 | 0 | 0 | 0 |
| BC_tissue | German_2023 | 1.2002 | 0.0033 | 2.4605 | 1.0135 | 0 | 1.2369 | 0.1034 | 1.3236 | 0 | 7.3281 | 0 | 0 | 0 | 0 |
| BC_tissue | German_2023 | 0.083 | 0.0917 | 1.5019 | 0 | 0.0087 | 0.0044 | 0 | 0.334 | 0 | 1.3251 | 0.0589 | 0 | 0 | 0 |
| BC_tissue | German_2023 | 0.253 | 0.0342 | 0.6455 | 0.0113 | 0.0006 | 0.2695 | 0.2562 | 0.0507 | 0.0009 | 0.4061 | 0 | 0 | 0 | 0.0003 |
| BC_tissue | German_2023 | 0.8912 | 0.6895 | 0.1874 | 3.7773 | 0.0695 | 0.8456 | 39.7186 | 0.0624 | 0.1497 | 0.3314 | 0.0303 | 0 | 0 | 0.0004 |
| BC_tissue | German_2023 | 2.6606 | 0.0424 | 2.2132 | 0.0568 | 0.0586 | 0.2675 | 1.2962 | 0.239 | 0.5106 | 2.3106 | 0 | 0 | 0 | 0.6756 |
| BC_tissue | German_2023 | 0.1601 | 0 | 2.1739 | 0.0024 | 0.0097 | 0.0024 | 0.0024 | 0.0946 | 0 | 1.1525 | 0 | 0 | 0 | 0 |
| BC_tissue | German_2023 | 0.745 | 0.6045 | 4.9904 | 0.0984 | 0 | 0.0187 | 0.9887 | 7.3567 | 0.0141 | 3.6362 | 0.0141 | 0 | 0 | 0.5998 |
| BC_tissue | German_2023 | 1.5127 | 0 | 11.0578 | 0.0107 | 0.0107 | 1.1825 | 3.4835 | 1.9282 | 2.6526 | 2.7804 | 0.6924 | 0.0107 | 0 | 0.0107 |
| BC_tissue | German_2023 | 0.262 | 0.0472 | 1.1712 | 0 | 0 | 0.1486 | 0.4021 | 0.1255 | 0.2546 | 0.5171 | 0 | 0 | 0 | 0.001 |
| BC_tissue | German_2023 | 0.1683 | 0 | 3.4697 | 0.0259 | 0 | 0.3107 | 0.0906 | 0 | 0.0259 | 11.1989 | 0 | 0 | 0 | 0 |
| BC_tissue | German_2023 | 0.5228 | 0 | 16.6809 | 0 | 0.019 | 0.8935 | 0 | 0.057 | 0 | 3.2031 | 0.0095 | 0 | 0.3517 | 3.8019 |
| BC_tissue | German_2023 | 4.622 | 0.0913 | 5.1498 | 0.9073 | 0.913 | 1.4979 | 2.4708 | 0.0456 | 2.3081 | 3.6434 | 0 | 0 | 0.0086 | 0 |
| BC_tissue | German_2023 | 0.762 | 0 | 6.671 | 0.2002 | 0 | 0.3616 | 0.7556 | 0.8654 | 0.2841 | 2.4282 | 0 | 0 | 0 | 0 |
| BC_tissue | German_2023 | 8.0762 | 0.252 | 29.8455 | 1.3654 | 0 | 2.7032 | 0.5401 | 0 | 0.0083 | 0.7644 | 0 | 0 | 0 | 2.9358 |
| BC_tissue | German_2023 | 0.4407 | 0 | 4.2531 | 0.0024 | 0.8968 | 0.0012 | 0.0059 | 0.3815 | 0 | 12.3684 | 0.0024 | 0 | 0 | 0 |
| BC_tissue | German_2023 | 2.3327 | 0.4486 | 23.3205 | 0 | 0 | 0.1727 | 2.3664 | 0.6931 | 0 | 0.5428 | 0.0022 | 0 | 0 | 0 |
| BC_tissue | German_2023 | 1.0178 | 0 | 1.6862 | 0 | 0.0004 | 1.6521 | 0.2293 | 60.6139 | 0.0004 | 0.034 | 0 | 0 | 0 | 0 |
| BC_tissue | German_2023 | 0.0654 | 0.0004 | 1.4335 | 0.0008 | 0 | 0.1701 | 0.0807 | 77.2513 | 0.0784 | 0.8295 | 0 | 0 | 0.0004 | 0 |
| BC_tissue | German_2023 | 0.4093 | 0.0048 | 1.6873 | 0.0003 | 0.0163 | 0.0022 | 1.2154 | 0.071 | 0.062 | 0.0403 | 0.0003 | 0 | 0 | 0 |
| BC_tissue | German_2023 | 0.6388 | 1.5092 | 2.641 | 0 | 0.0336 | 0.0299 | 0.7845 | 0.0261 | 0 | 3.87 | 0 | 0 | 0 | 0.2988 |
| BC_tissue | German_2023 | 0.7209 | 0.0503 | 22.9673 | 1.8441 | 0.0168 | 0.0671 | 4.2749 | 0.3521 | 0 | 9.7066 | 0 | 0 | 0 | 0 |
| BC_tissue | German_2023 | 1.286 | 0 | 2.7145 | 0.4815 | 0.0804 | 0.1492 | 0.0022 | 0.695 | 0 | 0.3943 | 0.4223 | 0 | 0 | 0 |
| BC_tissue | German_2023 | 10.1031 | 0.1057 | 1.4716 | 0.6609 | 0.2556 | 0.9121 | 4.1769 | 0.0132 | 0.7975 | 1.9122 | 0.0044 | 0 | 0 | 0.4979 |
| BC_tissue | German_2023 | 0.8941 | 0.0565 | 0.9138 | 0 | 0.0013 | 32.6016 | 0.7786 | 0.1352 | 0.0039 | 0.5685 | 0.9716 | 0 | 0 | 2.4645 |
| BC_tissue | German_2023 | 2.0973 | 0.0018 | 7.4662 | 0 | 8.5323 | 0.0037 | 0.0037 | 0.4661 | 0.0055 | 4.2973 | 0 | 0 | 0 | 0 |

**Table S5B. The RF scores at the genus level in both BC_tissue and normal_tissue samples.**

| **Group** | **Cohort** | **RF score** |
| --- | --- | --- |
| BC_tissue | Hoskinson_2022 | 0.08 |
| BC_tissue | Hoskinson_2022 | 0.14 |
| BC_tissue | Hoskinson_2022 | 0.07 |
| BC_tissue | Hoskinson_2022 | 0.26 |
| BC_tissue | Hoskinson_2022 | 0.23 |
| BC_tissue | Hoskinson_2022 | 0.35 |
| BC_tissue | Hoskinson_2022 | 0.14 |
| BC_tissue | Hoskinson_2022 | 0.18 |
| BC_tissue | Hoskinson_2022 | 0.15 |
| BC_tissue | Hoskinson_2022 | 0.32 |
| BC_tissue | Hoskinson_2022 | 0.18 |
| BC_tissue | Hoskinson_2022 | 0.18 |
| BC_tissue | Hoskinson_2022 | 0.07 |
| BC_tissue | Hoskinson_2022 | 0.05 |
| BC_tissue | Hoskinson_2022 | 0.25 |
| BC_tissue | Hoskinson_2022 | 0.24 |
| BC_tissue | Hoskinson_2022 | 0.03 |
| BC_tissue | Hoskinson_2022 | 0.02 |
| BC_tissue | Hoskinson_2022 | 0 |
| BC_tissue | Hoskinson_2022 | 0.12 |
| BC_tissue | Hoskinson_2022 | 0.12 |
| BC_tissue | Hoskinson_2022 | 0.01 |
| BC_tissue | Hoskinson_2022 | 0.04 |
| BC_tissue | Hoskinson_2022 | 0.04 |
| BC_tissue | Hoskinson_2022 | 0.08 |
| BC_tissue | Hoskinson_2022 | 0.09 |
| BC_tissue | Hoskinson_2022 | 0.06 |
| BC_tissue | Hoskinson_2022 | 0.05 |
| BC_tissue | Hoskinson_2022 | 0.04 |
| BC_tissue | Hoskinson_2022 | 0.01 |
| BC_tissue | Hoskinson_2022 | 0.03 |
| BC_tissue | Hoskinson_2022 | 0.01 |
| BC_tissue | Hoskinson_2022 | 0.09 |
| BC_tissue | Hoskinson_2022 | 0.1 |
| BC_tissue | Hoskinson_2022 | 0.09 |
| BC_tissue | Hoskinson_2022 | 0.05 |
| BC_tissue | Hoskinson_2022 | 0.03 |
| BC_tissue | Hoskinson_2022 | 0.09 |
| BC_tissue | Hoskinson_2022 | 0.11 |
| BC_tissue | Hoskinson_2022 | 0.19 |
| BC_tissue | Hoskinson_2022 | 0.32 |
| normal_tissue | Hoskinson_2022 | 0.67 |
| normal_tissue | Hoskinson_2022 | 0.69 |
| normal_tissue | Hoskinson_2022 | 0.72 |
| normal_tissue | Hoskinson_2022 | 0.84 |
| normal_tissue | Hoskinson_2022 | 0.98 |
| normal_tissue | Hoskinson_2022 | 0.95 |
| normal_tissue | Hoskinson_2022 | 0.98 |
| normal_tissue | Hoskinson_2022 | 0.89 |
| normal_tissue | Hoskinson_2022 | 0.96 |
| normal_tissue | Hoskinson_2022 | 0.93 |
| normal_tissue | Hoskinson_2022 | 0.73 |
| normal_tissue | Hoskinson_2022 | 0.94 |
| normal_tissue | Hoskinson_2022 | 0.77 |
| normal_tissue | Hoskinson_2022 | 0.98 |
| normal_tissue | Hoskinson_2022 | 0.9 |
| normal_tissue | Hoskinson_2022 | 0.99 |
| normal_tissue | Hoskinson_2022 | 0.91 |
| normal_tissue | Hoskinson_2022 | 0.97 |
| normal_tissue | Hoskinson_2022 | 1 |
| normal_tissue | Hoskinson_2022 | 0.99 |
| normal_tissue | Hoskinson_2022 | 0.9 |
| normal_tissue | Hoskinson_2022 | 1 |
| normal_tissue | Hoskinson_2022 | 0.97 |
| normal_tissue | Hoskinson_2022 | 0.96 |
| normal_tissue | Hoskinson_2022 | 0.85 |
| normal_tissue | Hoskinson_2022 | 0.89 |
| normal_tissue | Hoskinson_2022 | 0.86 |
| normal_tissue | Hoskinson_2022 | 0.98 |
| normal_tissue | Hoskinson_2022 | 0.97 |
| normal_tissue | Hoskinson_2022 | 0.91 |
| normal_tissue | Hoskinson_2022 | 0.93 |
| normal_tissue | Hoskinson_2022 | 0.75 |
| normal_tissue | Hoskinson_2022 | 0.99 |
| normal_tissue | Hoskinson_2022 | 0.85 |
| normal_tissue | Hoskinson_2022 | 0.85 |
| normal_tissue | Hoskinson_2022 | 1 |
| normal_tissue | Hoskinson_2022 | 0.99 |
| normal_tissue | Hoskinson_2022 | 0.92 |
| normal_tissue | Hoskinson_2022 | 0.93 |
| normal_tissue | Hoskinson_2022 | 0.97 |
| normal_tissue | Hoskinson_2022 | 1 |
| normal_tissue | Hoskinson_2022 | 1 |
| normal_tissue | Hoskinson_2022 | 0.94 |
| normal_tissue | Hoskinson_2022 | 0.96 |
| normal_tissue | Hoskinson_2022 | 0.98 |
| normal_tissue | Hoskinson_2022 | 0.98 |
| normal_tissue | Hoskinson_2022 | 0.97 |
| normal_tissue | Hoskinson_2022 | 0.94 |
| normal_tissue | Hoskinson_2022 | 0.92 |
| normal_tissue | German_2023 | 1 |
| normal_tissue | German_2023 | 0.98 |
| normal_tissue | German_2023 | 1 |
| normal_tissue | German_2023 | 0.98 |
| normal_tissue | German_2023 | 0.92 |
| normal_tissue | German_2023 | 1 |
| normal_tissue | German_2023 | 1 |
| normal_tissue | German_2023 | 0.99 |
| normal_tissue | German_2023 | 1 |
| normal_tissue | German_2023 | 1 |
| normal_tissue | German_2023 | 0.88 |
| normal_tissue | German_2023 | 1 |
| normal_tissue | German_2023 | 1 |
| normal_tissue | German_2023 | 0.98 |
| normal_tissue | German_2023 | 0.98 |
| normal_tissue | German_2023 | 1 |
| normal_tissue | German_2023 | 1 |
| normal_tissue | German_2023 | 1 |
| normal_tissue | German_2023 | 0.97 |
| normal_tissue | German_2023 | 1 |
| normal_tissue | German_2023 | 0.98 |
| normal_tissue | German_2023 | 0.99 |
| normal_tissue | German_2023 | 0.99 |
| normal_tissue | German_2023 | 0.95 |
| normal_tissue | German_2023 | 0.96 |
| normal_tissue | German_2023 | 1 |
| normal_tissue | German_2023 | 0.98 |
| normal_tissue | German_2023 | 1 |
| normal_tissue | German_2023 | 1 |
| normal_tissue | German_2023 | 1 |
| normal_tissue | German_2023 | 1 |
| normal_tissue | German_2023 | 0.99 |
| normal_tissue | German_2023 | 0.97 |
| normal_tissue | German_2023 | 0.98 |
| normal_tissue | German_2023 | 1 |
| normal_tissue | German_2023 | 0.95 |
| normal_tissue | German_2023 | 0.99 |
| normal_tissue | German_2023 | 0.99 |
| normal_tissue | German_2023 | 0.98 |
| normal_tissue | German_2023 | 0.85 |
| normal_tissue | German_2023 | 1 |
| normal_tissue | German_2023 | 1 |
| normal_tissue | German_2023 | 1 |
| normal_tissue | German_2023 | 1 |
| normal_tissue | German_2023 | 0.87 |
| normal_tissue | German_2023 | 1 |
| normal_tissue | German_2023 | 0.98 |
| normal_tissue | German_2023 | 0.99 |
| normal_tissue | German_2023 | 1 |
| normal_tissue | German_2023 | 0.99 |
| normal_tissue | German_2023 | 1 |
| normal_tissue | German_2023 | 1 |
| normal_tissue | German_2023 | 1 |
| normal_tissue | German_2023 | 1 |
| normal_tissue | German_2023 | 0.99 |
| normal_tissue | German_2023 | 0.98 |
| normal_tissue | German_2023 | 1 |
| normal_tissue | German_2023 | 1 |
| normal_tissue | German_2023 | 0.98 |
| normal_tissue | German_2023 | 0.99 |
| normal_tissue | German_2023 | 1 |
| normal_tissue | German_2023 | 0.99 |
| normal_tissue | German_2023 | 0.98 |
| normal_tissue | German_2023 | 1 |
| normal_tissue | German_2023 | 0.88 |
| normal_tissue | German_2023 | 1 |
| normal_tissue | German_2023 | 0.78 |
| normal_tissue | German_2023 | 0.9 |
| normal_tissue | German_2023 | 0.99 |
| normal_tissue | German_2023 | 0.99 |
| normal_tissue | German_2023 | 0.74 |
| normal_tissue | German_2023 | 0.99 |
| normal_tissue | German_2023 | 0.99 |
| normal_tissue | German_2023 | 0.96 |
| normal_tissue | German_2023 | 0.9 |
| normal_tissue | German_2023 | 0.96 |
| normal_tissue | German_2023 | 0.9 |
| normal_tissue | German_2023 | 0.99 |
| normal_tissue | German_2023 | 1 |
| normal_tissue | German_2023 | 0.84 |
| normal_tissue | German_2023 | 0.93 |
| normal_tissue | German_2023 | 0.99 |
| normal_tissue | German_2023 | 0.98 |
| normal_tissue | German_2023 | 0.98 |
| normal_tissue | German_2023 | 0.97 |
| normal_tissue | German_2023 | 0.99 |
| normal_tissue | German_2023 | 0.99 |
| normal_tissue | German_2023 | 0.99 |
| normal_tissue | German_2023 | 0.86 |
| normal_tissue | German_2023 | 1 |
| normal_tissue | German_2023 | 0.95 |
| normal_tissue | German_2023 | 0.98 |
| normal_tissue | German_2023 | 1 |
| normal_tissue | German_2023 | 0.98 |
| normal_tissue | German_2023 | 0.92 |
| normal_tissue | German_2023 | 0.95 |
| normal_tissue | German_2023 | 0.92 |
| normal_tissue | German_2023 | 0.96 |
| normal_tissue | German_2023 | 1 |
| normal_tissue | German_2023 | 1 |
| normal_tissue | German_2023 | 0.98 |
| normal_tissue | German_2023 | 0.98 |
| normal_tissue | German_2023 | 1 |
| normal_tissue | German_2023 | 0.94 |
| normal_tissue | German_2023 | 1 |
| normal_tissue | German_2023 | 1 |
| normal_tissue | German_2023 | 0.99 |
| normal_tissue | German_2023 | 0.99 |
| normal_tissue | German_2023 | 0.95 |
| normal_tissue | German_2023 | 1 |
| normal_tissue | German_2023 | 0.88 |
| normal_tissue | German_2023 | 1 |
| normal_tissue | German_2023 | 0.97 |
| normal_tissue | German_2023 | 0.97 |
| normal_tissue | German_2023 | 0.92 |
| normal_tissue | German_2023 | 0.97 |
| normal_tissue | German_2023 | 1 |
| normal_tissue | German_2023 | 0.99 |
| BC_tissue | Hoskinson_2022 | 0.13 |
| BC_tissue | Hoskinson_2022 | 0.1 |
| BC_tissue | Hoskinson_2022 | 0.1 |
| BC_tissue | Hoskinson_2022 | 0.03 |
| BC_tissue | Hoskinson_2022 | 0.17 |
| normal_tissue | German_2023 | 0.94 |
| normal_tissue | German_2023 | 0.97 |
| normal_tissue | German_2023 | 1 |
| normal_tissue | German_2023 | 1 |
| normal_tissue | German_2023 | 1 |
| normal_tissue | German_2023 | 1 |
| normal_tissue | German_2023 | 1 |
| normal_tissue | German_2023 | 1 |
| normal_tissue | German_2023 | 1 |
| normal_tissue | German_2023 | 1 |
| normal_tissue | German_2023 | 1 |
| normal_tissue | German_2023 | 0.99 |
| normal_tissue | German_2023 | 0.92 |
| normal_tissue | German_2023 | 1 |
| normal_tissue | German_2023 | 1 |
| normal_tissue | German_2023 | 1 |
| normal_tissue | German_2023 | 1 |
| normal_tissue | German_2023 | 1 |
| normal_tissue | German_2023 | 1 |
| normal_tissue | German_2023 | 0.92 |
| normal_tissue | German_2023 | 1 |
| normal_tissue | German_2023 | 0.99 |
| normal_tissue | German_2023 | 0.98 |
| normal_tissue | German_2023 | 0.99 |
| normal_tissue | German_2023 | 1 |
| normal_tissue | German_2023 | 0.98 |
| normal_tissue | German_2023 | 1 |
| normal_tissue | German_2023 | 0.92 |
| normal_tissue | German_2023 | 1 |
| normal_tissue | German_2023 | 0.95 |
| normal_tissue | German_2023 | 0.99 |
| normal_tissue | German_2023 | 0.97 |
| normal_tissue | German_2023 | 1 |
| normal_tissue | German_2023 | 0.96 |
| normal_tissue | German_2023 | 1 |
| normal_tissue | German_2023 | 0.99 |
| normal_tissue | German_2023 | 0.97 |
| normal_tissue | German_2023 | 0.99 |
| normal_tissue | German_2023 | 0.94 |
| normal_tissue | German_2023 | 0.95 |
| normal_tissue | German_2023 | 0.99 |
| normal_tissue | German_2023 | 0.99 |
| normal_tissue | German_2023 | 0.98 |
| normal_tissue | German_2023 | 1 |
| normal_tissue | German_2023 | 0.96 |
| normal_tissue | German_2023 | 1 |
| normal_tissue | German_2023 | 1 |
| normal_tissue | German_2023 | 0.96 |
| normal_tissue | German_2023 | 0.99 |
| normal_tissue | German_2023 | 0.99 |
| normal_tissue | German_2023 | 1 |
| normal_tissue | German_2023 | 1 |
| normal_tissue | German_2023 | 1 |
| normal_tissue | German_2023 | 0.94 |
| normal_tissue | German_2023 | 0.96 |
| normal_tissue | German_2023 | 1 |
| normal_tissue | German_2023 | 0.81 |
| normal_tissue | German_2023 | 0.96 |
| normal_tissue | German_2023 | 1 |
| normal_tissue | German_2023 | 0.97 |
| normal_tissue | German_2023 | 0.92 |
| normal_tissue | German_2023 | 1 |
| normal_tissue | German_2023 | 0.99 |
| normal_tissue | German_2023 | 1 |
| normal_tissue | German_2023 | 1 |
| normal_tissue | German_2023 | 1 |
| normal_tissue | German_2023 | 0.99 |
| normal_tissue | German_2023 | 1 |
| normal_tissue | German_2023 | 0.99 |
| normal_tissue | German_2023 | 1 |
| normal_tissue | German_2023 | 0.9 |
| normal_tissue | German_2023 | 0.99 |
| normal_tissue | German_2023 | 1 |
| normal_tissue | German_2023 | 1 |
| normal_tissue | German_2023 | 0.97 |
| normal_tissue | German_2023 | 1 |
| normal_tissue | German_2023 | 0.96 |
| normal_tissue | German_2023 | 0.98 |
| normal_tissue | German_2023 | 0.98 |
| normal_tissue | German_2023 | 1 |
| normal_tissue | German_2023 | 1 |
| normal_tissue | German_2023 | 0.97 |
| normal_tissue | German_2023 | 1 |
| normal_tissue | German_2023 | 1 |
| normal_tissue | German_2023 | 0.98 |
| normal_tissue | German_2023 | 0.93 |
| normal_tissue | German_2023 | 1 |
| normal_tissue | German_2023 | 1 |
| normal_tissue | German_2023 | 0.92 |
| normal_tissue | German_2023 | 0.98 |
| normal_tissue | German_2023 | 1 |
| normal_tissue | German_2023 | 1 |
| normal_tissue | German_2023 | 0.97 |
| normal_tissue | German_2023 | 0.99 |
| normal_tissue | German_2023 | 0.99 |
| normal_tissue | German_2023 | 0.99 |
| normal_tissue | German_2023 | 0.99 |
| normal_tissue | German_2023 | 0.98 |
| normal_tissue | German_2023 | 0.97 |
| normal_tissue | German_2023 | 1 |
| normal_tissue | German_2023 | 1 |
| normal_tissue | German_2023 | 1 |
| normal_tissue | German_2023 | 0.92 |
| normal_tissue | German_2023 | 0.98 |
| normal_tissue | German_2023 | 1 |
| normal_tissue | German_2023 | 0.93 |
| normal_tissue | German_2023 | 0.99 |
| normal_tissue | German_2023 | 0.99 |
| normal_tissue | German_2023 | 0.99 |
| normal_tissue | German_2023 | 1 |
| normal_tissue | German_2023 | 0.98 |
| normal_tissue | German_2023 | 1 |
| normal_tissue | German_2023 | 1 |
| normal_tissue | German_2023 | 1 |
| normal_tissue | German_2023 | 1 |
| normal_tissue | German_2023 | 1 |
| normal_tissue | German_2023 | 0.97 |
| normal_tissue | German_2023 | 1 |
| normal_tissue | German_2023 | 1 |
| normal_tissue | German_2023 | 1 |
| normal_tissue | German_2023 | 0.98 |
| normal_tissue | German_2023 | 0.99 |
| normal_tissue | German_2023 | 0.93 |
| normal_tissue | German_2023 | 0.87 |
| normal_tissue | German_2023 | 0.99 |
| normal_tissue | German_2023 | 1 |
| normal_tissue | German_2023 | 0.97 |
| normal_tissue | German_2023 | 1 |
| normal_tissue | German_2023 | 0.99 |
| normal_tissue | German_2023 | 1 |
| normal_tissue | German_2023 | 0.99 |
| normal_tissue | German_2023 | 0.98 |
| normal_tissue | German_2023 | 1 |
| normal_tissue | German_2023 | 1 |
| normal_tissue | German_2023 | 1 |
| normal_tissue | German_2023 | 1 |
| normal_tissue | German_2023 | 0.97 |
| normal_tissue | German_2023 | 0.87 |
| normal_tissue | German_2023 | 0.99 |
| normal_tissue | German_2023 | 0.98 |
| normal_tissue | German_2023 | 1 |
| normal_tissue | German_2023 | 1 |
| normal_tissue | German_2023 | 0.98 |
| normal_tissue | German_2023 | 0.96 |
| normal_tissue | German_2023 | 0.93 |
| normal_tissue | German_2023 | 0.93 |
| normal_tissue | German_2023 | 1 |
| normal_tissue | German_2023 | 0.97 |
| normal_tissue | German_2023 | 1 |
| normal_tissue | German_2023 | 0.96 |
| normal_tissue | German_2023 | 0.98 |
| normal_tissue | German_2023 | 0.94 |
| normal_tissue | German_2023 | 1 |
| normal_tissue | German_2023 | 0.99 |
| normal_tissue | German_2023 | 1 |
| normal_tissue | German_2023 | 1 |
| normal_tissue | German_2023 | 0.96 |
| normal_tissue | German_2023 | 1 |
| normal_tissue | German_2023 | 1 |
| normal_tissue | German_2023 | 0.86 |
| normal_tissue | German_2023 | 0.9 |
| normal_tissue | German_2023 | 0.99 |
| normal_tissue | German_2023 | 1 |
| normal_tissue | German_2023 | 0.99 |
| normal_tissue | German_2023 | 0.99 |
| normal_tissue | German_2023 | 0.96 |
| normal_tissue | German_2023 | 0.99 |
| normal_tissue | German_2023 | 0.92 |
| normal_tissue | German_2023 | 0.91 |
| normal_tissue | German_2023 | 0.95 |
| normal_tissue | German_2023 | 1 |
| normal_tissue | German_2023 | 0.91 |
| normal_tissue | German_2023 | 1 |
| normal_tissue | German_2023 | 1 |
| normal_tissue | German_2023 | 0.94 |
| normal_tissue | German_2023 | 1 |
| normal_tissue | German_2023 | 0.98 |
| normal_tissue | German_2023 | 0.98 |
| normal_tissue | German_2023 | 1 |
| normal_tissue | German_2023 | 1 |
| normal_tissue | German_2023 | 0.99 |
| normal_tissue | German_2023 | 1 |
| normal_tissue | German_2023 | 1 |
| normal_tissue | German_2023 | 0.99 |
| normal_tissue | German_2023 | 1 |
| normal_tissue | German_2023 | 0.97 |
| normal_tissue | German_2023 | 1 |
| normal_tissue | German_2023 | 0.91 |
| normal_tissue | German_2023 | 0.98 |
| normal_tissue | German_2023 | 1 |
| normal_tissue | German_2023 | 1 |
| normal_tissue | German_2023 | 0.99 |
| normal_tissue | German_2023 | 1 |
| normal_tissue | German_2023 | 1 |
| normal_tissue | German_2023 | 1 |
| normal_tissue | German_2023 | 1 |
| normal_tissue | German_2023 | 1 |
| normal_tissue | German_2023 | 1 |
| normal_tissue | German_2023 | 0.89 |
| normal_tissue | German_2023 | 0.93 |
| normal_tissue | German_2023 | 0.99 |
| normal_tissue | German_2023 | 1 |
| normal_tissue | German_2023 | 1 |
| normal_tissue | German_2023 | 1 |
| normal_tissue | German_2023 | 0.99 |
| normal_tissue | German_2023 | 0.98 |
| normal_tissue | German_2023 | 0.93 |
| normal_tissue | German_2023 | 1 |
| normal_tissue | German_2023 | 0.85 |
| normal_tissue | German_2023 | 0.98 |
| normal_tissue | German_2023 | 0.95 |
| normal_tissue | German_2023 | 0.84 |
| normal_tissue | German_2023 | 0.99 |
| normal_tissue | German_2023 | 0.99 |
| normal_tissue | German_2023 | 1 |
| normal_tissue | German_2023 | 1 |
| normal_tissue | German_2023 | 0.97 |
| normal_tissue | German_2023 | 0.92 |
| normal_tissue | German_2023 | 0.99 |
| normal_tissue | German_2023 | 1 |
| normal_tissue | German_2023 | 1 |
| normal_tissue | German_2023 | 1 |
| normal_tissue | German_2023 | 1 |
| normal_tissue | German_2023 | 1 |
| normal_tissue | German_2023 | 1 |
| normal_tissue | German_2023 | 1 |
| normal_tissue | German_2023 | 0.98 |
| normal_tissue | German_2023 | 1 |
| normal_tissue | German_2023 | 0.83 |
| normal_tissue | German_2023 | 1 |
| normal_tissue | German_2023 | 1 |
| normal_tissue | German_2023 | 1 |
| normal_tissue | German_2023 | 0.91 |
| normal_tissue | German_2023 | 1 |
| normal_tissue | German_2023 | 1 |
| normal_tissue | German_2023 | 0.97 |
| normal_tissue | German_2023 | 1 |
| normal_tissue | German_2023 | 0.83 |
| normal_tissue | German_2023 | 1 |
| normal_tissue | German_2023 | 0.95 |
| normal_tissue | German_2023 | 0.99 |
| normal_tissue | German_2023 | 1 |
| normal_tissue | German_2023 | 0.99 |
| normal_tissue | German_2023 | 0.88 |
| normal_tissue | German_2023 | 0.86 |
| normal_tissue | German_2023 | 1 |
| normal_tissue | German_2023 | 1 |
| normal_tissue | German_2023 | 0.99 |
| normal_tissue | German_2023 | 0.98 |
| normal_tissue | German_2023 | 1 |
| normal_tissue | German_2023 | 1 |
| normal_tissue | German_2023 | 1 |
| normal_tissue | German_2023 | 0.99 |
| normal_tissue | German_2023 | 0.95 |
| normal_tissue | German_2023 | 0.97 |
| normal_tissue | German_2023 | 1 |
| normal_tissue | German_2023 | 0.99 |
| normal_tissue | German_2023 | 0.97 |
| normal_tissue | German_2023 | 1 |
| normal_tissue | German_2023 | 1 |
| normal_tissue | German_2023 | 0.99 |
| normal_tissue | German_2023 | 1 |
| normal_tissue | German_2023 | 0.99 |
| normal_tissue | German_2023 | 1 |
| normal_tissue | German_2023 | 1 |
| normal_tissue | German_2023 | 1 |
| normal_tissue | German_2023 | 0.97 |
| normal_tissue | German_2023 | 1 |
| normal_tissue | German_2023 | 1 |
| normal_tissue | German_2023 | 1 |
| normal_tissue | German_2023 | 1 |
| normal_tissue | German_2023 | 0.99 |
| normal_tissue | German_2023 | 1 |
| normal_tissue | German_2023 | 1 |
| normal_tissue | German_2023 | 0.98 |
| normal_tissue | German_2023 | 1 |
| normal_tissue | German_2023 | 1 |
| normal_tissue | German_2023 | 1 |
| normal_tissue | German_2023 | 1 |
| normal_tissue | German_2023 | 1 |
| normal_tissue | German_2023 | 0.86 |
| normal_tissue | German_2023 | 1 |
| normal_tissue | German_2023 | 1 |
| normal_tissue | German_2023 | 1 |
| BC_tissue | German_2023 | 0.32 |
| BC_tissue | German_2023 | 0.23 |
| BC_tissue | German_2023 | 0.15 |
| BC_tissue | German_2023 | 0.35 |
| BC_tissue | German_2023 | 0.39 |
| BC_tissue | German_2023 | 0.25 |
| BC_tissue | German_2023 | 0.12 |
| BC_tissue | German_2023 | 0.4 |
| BC_tissue | German_2023 | 0.19 |
| BC_tissue | German_2023 | 0.23 |
| BC_tissue | German_2023 | 0.35 |
| BC_tissue | German_2023 | 0.26 |
| BC_tissue | German_2023 | 0.23 |
| BC_tissue | German_2023 | 0.3 |
| BC_tissue | German_2023 | 0.18 |
| BC_tissue | German_2023 | 0.21 |
| BC_tissue | German_2023 | 0.16 |
| BC_tissue | German_2023 | 0.26 |
| BC_tissue | German_2023 | 0.23 |
| BC_tissue | German_2023 | 0.24 |
| BC_tissue | German_2023 | 0.25 |
| BC_tissue | German_2023 | 0.32 |
| BC_tissue | German_2023 | 0.3 |
| BC_tissue | German_2023 | 0.12 |
| BC_tissue | German_2023 | 0.13 |
| BC_tissue | German_2023 | 0.11 |
| BC_tissue | German_2023 | 0.38 |
| BC_tissue | German_2023 | 0.29 |
| BC_tissue | German_2023 | 0.25 |
| BC_tissue | German_2023 | 0.24 |
