# Supplementary material for: Microbial diagnostic features identified across populations possess potential antitumor properties in breast cancer
Source: mSystems. 2025 Jun 23;10(7):e00271-25. doi: 10.1128/msystems.00271-25 (PMC12282184; doi:10.1128/msystems.00271-25)
Supplement: Table S6 — The abundance of the 20 specific species in both BC_tissue and BC_adjacent samples. [file msystems.00271-25-s0006.doc]

**Table S6A. The abundance of the 20 specific species in both BC_tissue and BC_adjacent samples.**

| **Group** | **Cohort** | ***Cutibacterium_acnes*** | ***Acinetobacter_johnsonii*** | ***Pseudomonas_O_647615_parafulva*** | ***Ralstonia_pickettii_B*** | ***Atopostipes_suicloacalis*** | ***Rubrobacter_B_405439_xylanophilus*** | ***Novosphingobium_capsulatum*** | ***Psychrobacter_maritimus*** | ***Finegoldia_magna_H*** | ***Thermus_A_scotoductus*** | ***Phocaeicola_A_858004_vulgatus*** | ***Acinetobacter_harbinensis*** | ***Bifidobacterium_thermophilum*** | ***Tepidimonas_fonticaldi*** |
| --- | --- | --- | --- | --- | --- | --- | --- | --- | --- | --- | --- | --- | --- | --- | --- |
| BC_tissue | Hoskinson_2022 | 0.3511 | 0 | 0.0042 | 0 | 0.055 | 0 | 0.0973 | 0 | 0 | 0 | 0 | 0 | 0 | 0 |
| BC_tissue | Hoskinson_2022 | 0.0017 | 0 | 0.5226 | 0 | 0.0157 | 0 | 0 | 0 | 0 | 0 | 0 | 0 | 0 | 0 |
| BC_tissue | Hoskinson_2022 | 0.4019 | 0 | 0.476 | 0 | 18.7692 | 0 | 3.9408 | 0 | 0 | 0 | 0 | 0 | 0 | 0 |
| BC_tissue | Hoskinson_2022 | 0.5535 | 0 | 2.233 | 0 | 0 | 0 | 0.9507 | 0 | 0 | 0 | 0 | 0 | 0 | 0 |
| BC_tissue | Hoskinson_2022 | 0.9261 | 0 | 23.5042 | 0 | 0.0021 | 0 | 0.0064 | 0 | 0 | 0 | 0 | 0 | 0 | 0 |
| BC_tissue | Hoskinson_2022 | 1.1357 | 0.3223 | 0.003 | 0 | 0.006 | 0 | 0.0587 | 0 | 0 | 0 | 0 | 0 | 0.003 | 0 |
| BC_tissue | Hoskinson_2022 | 2.2896 | 0 | 0 | 0 | 0 | 0 | 0.0014 | 0 | 0 | 0 | 0.0014 | 0 | 0 | 0 |
| BC_tissue | Hoskinson_2022 | 0.6074 | 0 | 11.5905 | 0 | 0 | 0 | 7.0664 | 0 | 0.0022 | 0 | 2.7396 | 0.0011 | 0 | 0 |
| BC_tissue | Hoskinson_2022 | 0.3146 | 0.0096 | 1.3487 | 0 | 0.6216 | 0 | 0 | 0 | 0 | 0 | 0 | 0 | 0 | 0 |
| BC_tissue | Hoskinson_2022 | 0.4978 | 0 | 15.0098 | 0 | 0.0101 | 0 | 0 | 0 | 0 | 0 | 0 | 0 | 0 | 0 |
| BC_tissue | Hoskinson_2022 | 3.1187 | 0 | 25.279 | 0 | 0 | 0 | 0 | 0 | 0 | 0 | 0 | 0 | 0 | 0 |
| BC_tissue | Hoskinson_2022 | 4.0837 | 0 | 0.9293 | 0 | 0 | 0 | 0 | 0 | 0 | 0 | 0.0034 | 0 | 0 | 0 |
| BC_tissue | Hoskinson_2022 | 0.0017 | 0 | 0 | 0 | 0 | 0 | 0 | 0 | 0.0017 | 0 | 0 | 0 | 0 | 0 |
| BC_tissue | Hoskinson_2022 | 0.0276 | 0 | 1.2825 | 0 | 0.0013 | 0 | 0 | 0 | 12.5322 | 0 | 0 | 0 | 0 | 0 |
| BC_tissue | Hoskinson_2022 | 1.134 | 0 | 15.7953 | 0 | 0 | 0 | 0 | 0 | 0 | 0 | 0 | 0 | 0 | 0 |
| BC_tissue | Hoskinson_2022 | 1.3141 | 0 | 0.0334 | 0 | 0 | 0 | 29.3602 | 0 | 0 | 0.002 | 0.0059 | 0 | 0 | 0 |
| BC_tissue | Hoskinson_2022 | 0.0836 | 0 | 5.3383 | 0 | 0.0777 | 0 | 0.002 | 0 | 0.002 | 0 | 0 | 0 | 0 | 0 |
| BC_tissue | Hoskinson_2022 | 0.0078 | 0 | 2.1896 | 0 | 0.0011 | 0 | 0 | 0 | 0 | 0 | 0 | 0 | 0 | 0 |
| BC_tissue | Hoskinson_2022 | 0.0675 | 0 | 0.0013 | 0 | 0.0344 | 0 | 0 | 0 | 0.0013 | 0 | 0 | 0 | 0 | 0 |
| BC_tissue | Hoskinson_2022 | 0 | 0 | 0.0102 | 0 | 0 | 0 | 0.0217 | 0 | 1.3195 | 0 | 0 | 0 | 0 | 0 |
| BC_tissue | Hoskinson_2022 | 0 | 0 | 0 | 0 | 0 | 0 | 0 | 0 | 1.3381 | 0 | 0 | 0.0085 | 0 | 0 |
| BC_tissue | Hoskinson_2022 | 0.0553 | 0 | 6.5655 | 0 | 0.018 | 0 | 0.0026 | 0 | 0 | 0 | 0 | 0 | 0 | 0 |
| BC_tissue | Hoskinson_2022 | 0.0685 | 0 | 0.0022 | 0 | 0.0055 | 0 | 0 | 0 | 0 | 0 | 0.0784 | 0.0011 | 0 | 0 |
| BC_tissue | Hoskinson_2022 | 0.1121 | 0.0023 | 12.6656 | 0 | 0.0127 | 0 | 0.0012 | 0 | 0 | 0 | 0.2831 | 0 | 0 | 0 |
| BC_tissue | Hoskinson_2022 | 0.1576 | 0 | 12.5637 | 0 | 0.0812 | 0 | 3.3041 | 0 | 0.0096 | 0 | 0 | 0 | 0 | 0 |
| BC_tissue | Hoskinson_2022 | 0.03 | 0 | 1.7021 | 0 | 0.003 | 0 | 0 | 0 | 0 | 0 | 0 | 0 | 0 | 0 |
| BC_tissue | Hoskinson_2022 | 0.1623 | 0.0012 | 24 | 0 | 0.02 | 0 | 0 | 0 | 0 | 0 | 0 | 0 | 0 | 0 |
| BC_tissue | Hoskinson_2022 | 0.0749 | 0 | 12.6406 | 0 | 0.0029 | 0 | 0 | 0 | 0 | 0 | 0 | 0 | 0 | 0 |
| BC_tissue | Hoskinson_2022 | 0.2343 | 0 | 19.5969 | 0 | 0.023 | 0 | 0 | 0 | 0 | 0 | 0 | 0 | 0 | 0 |
| BC_tissue | Hoskinson_2022 | 0.0066 | 0 | 0.7572 | 0 | 0 | 0 | 0 | 0 | 0 | 0 | 0 | 0 | 0 | 0 |
| BC_tissue | Hoskinson_2022 | 0.1587 | 0 | 39.7347 | 0 | 0.1123 | 0 | 0 | 0 | 0 | 0 | 0 | 0 | 0 | 0 |
| BC_tissue | Hoskinson_2022 | 0.2317 | 0 | 0.007 | 0 | 0.0255 | 0 | 0.0023 | 0 | 0 | 1.6914 | 0 | 0 | 0.0023 | 0 |
| BC_tissue | Hoskinson_2022 | 0.0036 | 0 | 15.9687 | 0 | 0.0234 | 0 | 5.1528 | 0 | 0.0054 | 0.0018 | 0 | 0 | 0 | 0 |
| BC_tissue | Hoskinson_2022 | 0.0054 | 0 | 3.6162 | 0 | 0.0036 | 0 | 0 | 0 | 0 | 0 | 0.0036 | 0 | 0 | 0 |
| BC_tissue | Hoskinson_2022 | 0.2788 | 0.0024 | 0.0024 | 0 | 0.0236 | 0 | 0 | 0.0024 | 0 | 0 | 0 | 0.0024 | 0 | 0.0024 |
| BC_tissue | Hoskinson_2022 | 0.1035 | 0.0054 | 0.0161 | 0 | 0 | 0.0018 | 0.0018 | 0 | 0 | 2.7221 | 0 | 0 | 0 | 0 |
| BC_tissue | Hoskinson_2022 | 0.0597 | 0.0021 | 0 | 0 | 0 | 0 | 0 | 0 | 0 | 0 | 0 | 0 | 0 | 0 |
| BC_tissue | Hoskinson_2022 | 0.0071 | 0 | 0.6186 | 0 | 0 | 0 | 0 | 0 | 0 | 0 | 0 | 0 | 0 | 0 |
| BC_tissue | Hoskinson_2022 | 0.1233 | 0 | 2.4616 | 0 | 0.0022 | 0 | 0.0022 | 0 | 0 | 0 | 0 | 0.0022 | 0 | 0 |
| BC_tissue | Hoskinson_2022 | 0 | 0.0047 | 0.5154 | 0 | 0 | 0 | 0 | 0 | 0 | 0 | 0 | 0 | 0 | 0 |
| BC_tissue | Hoskinson_2022 | 1.2373 | 0 | 0 | 0 | 0.0021 | 0 | 0 | 0 | 0 | 0 | 0 | 0 | 0 | 0 |
| BC_adjacent | German_2023 | 1.4141 | 0.0126 | 0.001 | 0.031 | 0 | 0 | 0 | 0 | 0 | 0 | 0 | 0 | 0.0068 | 0.0474 |
| BC_adjacent | German_2023 | 0.9264 | 0.0009 | 0 | 1.2407 | 0 | 0 | 0 | 0 | 1.1621 | 0 | 0.0009 | 0 | 0.0544 | 0.0009 |
| BC_adjacent | German_2023 | 0.6379 | 0.083 | 0 | 0.2922 | 0 | 0 | 0 | 0 | 0 | 0 | 0 | 0 | 0 | 0 |
| BC_adjacent | German_2023 | 0.7858 | 0 | 0 | 0.7173 | 0 | 0 | 0 | 0 | 0 | 0 | 0 | 0 | 0 | 0.0005 |
| BC_adjacent | German_2023 | 0.0463 | 0.0012 | 0 | 0.0012 | 0 | 0 | 0 | 0 | 0 | 0 | 0 | 0 | 0 | 0 |
| BC_adjacent | German_2023 | 0.124 | 0.056 | 0 | 0.1341 | 0 | 0 | 0 | 0 | 0 | 0.0364 | 0 | 0 | 0.0003 | 0 |
| BC_adjacent | German_2023 | 0.5465 | 0.1091 | 0 | 0.2581 | 0 | 0 | 0 | 0 | 0 | 0 | 0 | 0 | 0.059 | 0.9042 |
| BC_adjacent | German_2023 | 3.162 | 0.3089 | 0 | 0.0927 | 0.0008 | 0 | 0 | 0 | 0 | 0 | 0 | 0 | 0 | 0 |
| BC_adjacent | German_2023 | 0.5203 | 0.0039 | 0 | 0 | 0 | 0 | 0 | 0 | 0 | 0 | 0 | 0 | 0.0231 | 0 |
| BC_adjacent | German_2023 | 0.5393 | 0.0002 | 0 | 0.0859 | 0.2419 | 0.0002 | 0 | 0 | 0.1595 | 0 | 0 | 0 | 0.0004 | 0 |
| BC_adjacent | German_2023 | 0.1989 | 0 | 0 | 0.4172 | 0 | 0 | 0 | 0 | 0 | 0.0022 | 0 | 0 | 0.0086 | 0.8084 |
| BC_adjacent | German_2023 | 0.0764 | 0.0826 | 0 | 0 | 0 | 0 | 0 | 0 | 0 | 0 | 0 | 0 | 5.4146 | 0 |
| BC_adjacent | German_2023 | 0.4893 | 0 | 0 | 0.0025 | 0.139 | 0 | 0 | 0 | 0 | 0 | 0 | 0 | 0.0004 | 0 |
| BC_adjacent | German_2023 | 2.3613 | 0.7305 | 0 | 0.7159 | 0 | 0 | 0 | 0 | 0 | 0 | 0 | 0 | 0 | 0 |
| BC_adjacent | German_2023 | 0.7466 | 0 | 0 | 0.0542 | 0 | 0 | 0 | 0 | 0 | 0 | 0 | 0 | 0 | 0.0013 |
| BC_adjacent | German_2023 | 1.412 | 0.0298 | 0.0304 | 27.8143 | 0.011 | 0 | 0 | 0 | 0.1457 | 0 | 0 | 0 | 0.0641 | 0 |
| BC_adjacent | German_2023 | 0.144 | 0.0101 | 0 | 40.4147 | 0 | 0 | 0 | 0 | 0 | 0 | 0 | 0 | 0.0022 | 0 |
| BC_adjacent | German_2023 | 0.1122 | 0.0073 | 0 | 0.0345 | 0 | 0.0007 | 0 | 0 | 0 | 0.0154 | 0 | 0 | 0.0037 | 0 |
| BC_adjacent | German_2023 | 2.7565 | 0 | 0 | 0.001 | 0 | 0 | 0 | 0 | 0 | 0 | 0 | 0 | 0 | 0.001 |
| BC_adjacent | German_2023 | 1.9324 | 0.0821 | 0 | 0.0881 | 0 | 0 | 0 | 0 | 0 | 0.02 | 0 | 0.002 | 0 | 0.0761 |
| BC_adjacent | German_2023 | 3.7831 | 0.0452 | 0 | 0.0589 | 0.6734 | 0 | 0 | 0 | 0 | 0 | 0 | 0 | 0.2964 | 0.0079 |
| BC_adjacent | German_2023 | 3.3934 | 0.0123 | 0 | 0.4072 | 0 | 0 | 0 | 0 | 0 | 0 | 0 | 0 | 0.5183 | 0 |
| BC_adjacent | German_2023 | 0.3588 | 0.0454 | 0 | 52.2441 | 0 | 0 | 0 | 0 | 0 | 0 | 0 | 0 | 0 | 0 |
| BC_adjacent | German_2023 | 0.0676 | 0.0258 | 0 | 57.4617 | 0 | 0 | 0 | 0 | 0 | 0 | 0 | 0 | 0 | 0 |
| BC_adjacent | German_2023 | 2.2394 | 0 | 0 | 17.3769 | 0.4419 | 0 | 0 | 0 | 0 | 0 | 0 | 0 | 0 | 0 |
| BC_adjacent | German_2023 | 0.1867 | 0 | 0 | 0.0025 | 0 | 0 | 0 | 0 | 0 | 0.0393 | 0 | 0 | 0.0049 | 1.2431 |
| BC_adjacent | German_2023 | 1.6288 | 0.0012 | 0 | 0.3046 | 0 | 0 | 0 | 0 | 0 | 0 | 0 | 0 | 0 | 0.0842 |
| BC_adjacent | German_2023 | 0.0033 | 0.0011 | 0 | 0.2662 | 0.0011 | 0 | 0 | 0 | 0 | 0 | 0 | 0 | 0 | 0 |
| BC_adjacent | German_2023 | 0.0052 | 0 | 0.0017 | 54.7675 | 0 | 0 | 0 | 0 | 0 | 0.0035 | 0 | 0 | 0 | 0 |
| BC_adjacent | German_2023 | 2.1923 | 0.0283 | 0 | 0 | 0 | 0 | 0 | 0 | 0 | 0.0152 | 0 | 0 | 0 | 0 |
| BC_adjacent | German_2023 | 0.0164 | 0 | 0 | 43.9355 | 0 | 0 | 0 | 0 | 0 | 0 | 0 | 0 | 0 | 0 |
| BC_adjacent | German_2023 | 0.7157 | 0.0035 | 0 | 0 | 0 | 0 | 0 | 0 | 1.199 | 0.035 | 0 | 0 | 0 | 0 |
| BC_adjacent | German_2023 | 0.059 | 0 | 0.0139 | 0.0312 | 0 | 0 | 0 | 0 | 0 | 0 | 0 | 0 | 0.0312 | 0.2324 |
| BC_adjacent | German_2023 | 0.4487 | 0 | 0 | 0.3424 | 0 | 0 | 0 | 0 | 0 | 0 | 0 | 0 | 0.003 | 0.0007 |
| BC_tissue | Hoskinson_2022 | 1.9872 | 0 | 0 | 0 | 0 | 0 | 0.0015 | 0 | 0 | 0 | 0 | 0 | 0 | 0 |
| BC_tissue | Hoskinson_2022 | 0.0113 | 4.7287 | 0.8095 | 0 | 0.0038 | 0 | 0 | 0 | 0 | 0 | 0 | 1.2227 | 0 | 0 |
| BC_tissue | Hoskinson_2022 | 0.7355 | 0 | 2.9998 | 0 | 0.0014 | 0 | 0.0043 | 0 | 0 | 0 | 0 | 0 | 0 | 0 |
| BC_tissue | Hoskinson_2022 | 0.0249 | 0 | 29.5511 | 0 | 0 | 0 | 11.5822 | 0 | 0 | 0 | 0 | 0 | 0 | 0 |
| BC_tissue | Hoskinson_2022 | 0.2844 | 5.8527 | 0.0022 | 0 | 0.0086 | 0 | 0 | 0 | 0 | 0 | 0 | 0 | 0 | 0 |
| BC_tissue | Hoskinson_2022 | 3.8565 | 39.0797 | 0.0154 | 0 | 0 | 0 | 0 | 0 | 0 | 0 | 0 | 0 | 0 | 0 |
| BC_tissue | Esposito_2022 | 0.9329 | 10.7807 | 0.007 | 0 | 0 | 0 | 0 | 0 | 0 | 0 | 0 | 0 | 0 | 0 |
| BC_tissue | Esposito_2022 | 10.9037 | 0.5516 | 0 | 0 | 0 | 0.0276 | 0 | 0 | 0 | 0.1103 | 0 | 0 | 0 | 0 |
| BC_tissue | Esposito_2022 | 4.5661 | 5.6129 | 0 | 0 | 0 | 0 | 0 | 0 | 10.2549 | 0 | 0 | 0 | 0 | 0 |
| BC_tissue | Esposito_2022 | 0.2574 | 14.8391 | 0.0129 | 0 | 0 | 0 | 0 | 0 | 0 | 0 | 4.1956 | 0 | 0 | 0 |
| BC_tissue | Esposito_2022 | 3.9039 | 23.4323 | 0 | 0 | 0 | 6.6331 | 0 | 0 | 0 | 0 | 0 | 0 | 0 | 0 |
| BC_tissue | Esposito_2022 | 22.7523 | 0.0524 | 0 | 0 | 0 | 0.2097 | 0 | 0 | 0 | 0 | 0 | 0 | 0 | 0 |
| BC_tissue | Esposito_2022 | 0.1323 | 6.8053 | 0.0378 | 0 | 0 | 0.0567 | 0 | 0 | 0 | 0 | 0.0756 | 0 | 0 | 0 |
| BC_tissue | Esposito_2022 | 4.3525 | 0.3245 | 0 | 0 | 0 | 1.3109 | 0 | 0 | 0 | 0 | 0 | 0 | 0 | 0 |
| BC_tissue | Esposito_2022 | 14.0533 | 11.407 | 0 | 0 | 0 | 0 | 0 | 0 | 0.2794 | 0 | 0 | 0 | 0 | 0.0164 |
| BC_tissue | Esposito_2022 | 3.7558 | 16.7226 | 0 | 0 | 0.5665 | 2.3919 | 0 | 0 | 0 | 0 | 0 | 0 | 0 | 0 |
| BC_tissue | Esposito_2022 | 1.5328 | 3.5766 | 0 | 0 | 0 | 0 | 0 | 0 | 0 | 0 | 0.0111 | 0.0111 | 0 | 0 |
| BC_tissue | Esposito_2022 | 2.4819 | 16.161 | 0.019 | 0 | 0 | 1.9487 | 0 | 0 | 0 | 0 | 0 | 0 | 0 | 4.3037 |
| BC_tissue | Esposito_2022 | 4.7498 | 28.7038 | 0.0049 | 0 | 0 | 6.0811 | 0 | 0 | 0 | 0 | 0 | 0.0098 | 0 | 0.1463 |
| BC_tissue | Esposito_2022 | 2.9489 | 9.3897 | 0.0147 | 0 | 0 | 4.4748 | 0 | 0 | 0 | 0 | 0 | 0 | 0 | 0 |
| BC_tissue | Esposito_2022 | 5.1372 | 7.1866 | 0 | 0 | 0 | 1.4373 | 0 | 0 | 0 | 0 | 0 | 0 | 0 | 0 |
| BC_tissue | Esposito_2022 | 10.0744 | 8.4348 | 0 | 2.7214 | 0 | 2.2819 | 0 | 0 | 0 | 0 | 0 | 0 | 0 | 0 |
| BC_tissue | Esposito_2022 | 5.2516 | 2.3733 | 0.0038 | 0 | 0 | 0 | 0 | 0 | 0 | 0 | 0 | 0 | 0 | 0 |
| BC_tissue | Esposito_2022 | 3.2134 | 0 | 0 | 0 | 0 | 0.8965 | 0 | 0 | 0 | 0 | 5.846 | 0 | 0 | 0 |
| BC_tissue | Esposito_2022 | 2.3641 | 15.1449 | 0.0362 | 0 | 0 | 0.0543 | 0 | 0 | 0 | 0 | 0 | 0 | 0 | 0 |
| BC_tissue | Esposito_2022 | 13.5812 | 0.0585 | 0 | 0 | 0 | 2.7221 | 0 | 0 | 0 | 0 | 0 | 0 | 0 | 0 |
| BC_tissue | Esposito_2022 | 15.41 | 0.0174 | 0.0174 | 0 | 0 | 0 | 0 | 0 | 0 | 0 | 0 | 0 | 0 | 0 |
| BC_tissue | Esposito_2022 | 1.8501 | 37.966 | 0 | 0 | 0 | 4.1953 | 0 | 0 | 0 | 0 | 0 | 0 | 0 | 0 |
| BC_tissue | Esposito_2022 | 12.8221 | 20.3659 | 0.003 | 0 | 0.0473 | 4.58 | 0 | 0 | 0 | 0 | 0 | 0 | 0 | 0.0015 |
| BC_tissue | Esposito_2022 | 8.2345 | 2.2554 | 0 | 0 | 0 | 0 | 0 | 0 | 0 | 0 | 0 | 0.0352 | 0 | 0 |
| BC_tissue | Esposito_2022 | 4.1349 | 43.8093 | 0.0586 | 0.3046 | 0.0234 | 3.5727 | 0 | 0 | 0.0117 | 0 | 0 | 0.0117 | 0 | 0 |
| BC_tissue | Esposito_2022 | 4.7662 | 14.3088 | 0 | 0 | 0 | 0.9209 | 0 | 0 | 0 | 0 | 0 | 0 | 0 | 0.1316 |
| BC_tissue | Esposito_2022 | 1.9068 | 20.5121 | 0 | 0 | 0 | 1.0624 | 0 | 0 | 0 | 0 | 0 | 0 | 0 | 0 |
| BC_tissue | Esposito_2022 | 3.9313 | 29.4887 | 0 | 0 | 0 | 1.5284 | 0 | 0 | 0.2757 | 0 | 0 | 0 | 0 | 2.2138 |
| BC_tissue | Esposito_2022 | 0.9899 | 0 | 0 | 0 | 0 | 0 | 0 | 0 | 0 | 0 | 0 | 0 | 0 | 0 |
| BC_tissue | Esposito_2022 | 5.5166 | 46.739 | 0.0066 | 0 | 0 | 0.4961 | 0 | 0 | 0 | 0 | 0 | 0 | 0 | 0 |
| BC_tissue | Esposito_2022 | 6.3894 | 20.6612 | 0 | 0.3732 | 0 | 0.8531 | 0 | 0 | 0 | 0 | 0 | 0.0089 | 0 | 0.1333 |
| BC_tissue | Esposito_2022 | 1.5486 | 21.2516 | 0 | 0 | 0 | 5.8285 | 0 | 0 | 0 | 0 | 0 | 0 | 0 | 0 |
| BC_tissue | Esposito_2022 | 4.8301 | 12.9328 | 0.0054 | 0 | 0 | 0.2931 | 0 | 0 | 0 | 0 | 0 | 0 | 0 | 0.9932 |
| BC_tissue | Kartti_2023 | 0.0481 | 0.0436 | 0.0069 | 0.0138 | 0 | 0 | 0 | 1.4925 | 0.1857 | 0 | 0.4539 | 0.1032 | 0 | 0 |
| BC_tissue | Kartti_2023 | 0.1469 | 0.9433 | 0.0532 | 0.1001 | 0 | 0 | 0.0916 | 1.0178 | 0 | 0.4493 | 0 | 0.477 | 0 | 0 |
| BC_tissue | Kartti_2023 | 0.103 | 0.8107 | 0.0687 | 0 | 0.8364 | 0 | 0.3435 | 0.8742 | 0 | 0.5822 | 0 | 0.979 | 0 | 0 |
| BC_tissue | Kartti_2023 | 0.0396 | 0.8467 | 0 | 0 | 0 | 0 | 0 | 3.9724 | 0 | 0 | 0 | 0.0607 | 0 | 0 |
| BC_tissue | Kartti_2023 | 0.008 | 0 | 0 | 0.004 | 0 | 0 | 0 | 0.9982 | 0 | 0 | 0.1208 | 0 | 0 | 0 |
| BC_tissue | Kartti_2023 | 0.4286 | 0.1531 | 0.0383 | 0.051 | 0.0459 | 0 | 0 | 0.4847 | 0 | 0.1735 | 0.0306 | 0.0842 | 0.4031 | 0.023 |
| BC_tissue | Kartti_2023 | 0.0978 | 0.0059 | 0 | 0 | 0 | 0 | 0.003 | 2.2289 | 0 | 0 | 0.08 | 0.0267 | 0.003 | 0 |
| BC_tissue | Kartti_2023 | 0.0344 | 0.0413 | 0.0138 | 0 | 0 | 0 | 0 | 1.8678 | 0 | 0 | 0.0619 | 0.0447 | 0.0138 | 0 |
| BC_tissue | Kartti_2023 | 0.0057 | 0 | 0 | 0 | 0.2566 | 0 | 0 | 1.6423 | 0 | 0 | 0.154 | 0.0399 | 0.0399 | 0 |
| BC_tissue | Kartti_2023 | 1.2696 | 1.3344 | 0.024 | 0 | 0 | 0 | 0 | 0.792 | 0 | 0 | 0.0288 | 0.0432 | 0 | 0 |
| BC_tissue | Kartti_2023 | 0.4674 | 1.0044 | 0.0022 | 0 | 0 | 0 | 0 | 0.4764 | 0 | 0 | 0 | 0.0382 | 0.0247 | 0 |
| BC_tissue | Kartti_2023 | 0.0699 | 0 | 0.0815 | 0 | 0 | 0 | 0 | 1.9336 | 0 | 0.0349 | 0 | 0.0932 | 0 | 0 |
| BC_tissue | Kartti_2023 | 0.4053 | 0.3597 | 0 | 0.041 | 0 | 0 | 0 | 2.6206 | 0.0751 | 0.3233 | 0 | 0.1002 | 0 | 0 |
| BC_tissue | Kartti_2023 | 0.1706 | 0.6708 | 0.2434 | 0 | 0.0058 | 0 | 0.0517 | 1.5179 | 0 | 1.4604 | 0 | 0.2147 | 0 | 0 |
| BC_tissue | Kartti_2023 | 0.0204 | 0 | 0 | 0.0163 | 0 | 0 | 0 | 0.1263 | 0 | 0.0489 | 0 | 0 | 0 | 0 |
| BC_tissue | Kartti_2023 | 0.1353 | 0.2207 | 0.0229 | 0.0666 | 0.0042 | 0 | 0 | 0.6641 | 0 | 0.1166 | 0 | 0.0208 | 0 | 0 |
| BC_tissue | Kartti_2023 | 3.6618 | 2.8916 | 0.4209 | 0.0021 | 0.3199 | 0.5808 | 0 | 1.5868 | 0 | 0.4988 | 0.1242 | 0.0589 | 0 | 0 |
| BC_tissue | Kartti_2023 | 0.2607 | 0.3994 | 0.0638 | 0.0388 | 0 | 0 | 0 | 0.4631 | 0 | 0 | 0.025 | 0.0166 | 0 | 0 |
| BC_tissue | Kartti_2023 | 2.2865 | 0.2147 | 0.0849 | 0 | 0 | 0 | 0 | 1.7922 | 0 | 0.5392 | 0 | 0.1198 | 0 | 0.0499 |
| BC_tissue | Kartti_2023 | 1.0251 | 0.5478 | 0 | 0 | 0 | 0 | 0 | 4.5667 | 0 | 0.1681 | 0 | 0.0434 | 0 | 0 |
| BC_tissue | Kartti_2023 | 0 | 0.0298 | 0.0794 | 0 | 0 | 0 | 0 | 2.3023 | 0 | 0.3672 | 0 | 0.1389 | 0 | 0 |
| BC_tissue | Kartti_2023 | 0.7475 | 0.9264 | 0 | 0.0703 | 0 | 0 | 0 | 3.562 | 0 | 0.2779 | 0 | 0.1022 | 0.1853 | 0 |
| BC_tissue | Kartti_2023 | 0.3212 | 0.0347 | 0 | 0.1563 | 0 | 0 | 0 | 2.7346 | 0 | 0 | 0 | 0 | 0 | 0 |
| BC_tissue | Kartti_2023 | 0.3334 | 0.1396 | 0.1681 | 0.0057 | 0.0513 | 0.0142 | 0 | 1.4731 | 0 | 0 | 0 | 0.0028 | 0 | 0 |
| BC_tissue | Kartti_2023 | 1.6891 | 0.1979 | 0 | 0.109 | 0.0459 | 0 | 0 | 1.4253 | 0 | 0 | 0 | 0 | 0 | 0 |
| BC_tissue | Kartti_2023 | 0.0071 | 0.8084 | 1.2255 | 0 | 0 | 0 | 0 | 0 | 0 | 0 | 0 | 0.3299 | 0 | 0 |
| BC_tissue | Kartti_2023 | 0.0092 | 0.0386 | 0.0643 | 0.0129 | 0 | 0 | 0 | 2.9204 | 0 | 0 | 0 | 0.0514 | 0 | 0.0147 |
| BC_tissue | Kartti_2023 | 1.3805 | 0.6314 | 0.2 | 0 | 0 | 0.2235 | 0 | 4.6553 | 0 | 0 | 0.0784 | 0.3412 | 0 | 0 |
| BC_tissue | Kartti_2023 | 0.0714 | 0.0842 | 0.1122 | 0.0918 | 0 | 0 | 0 | 1.6779 | 0.0127 | 0 | 0 | 0 | 0 | 0 |
| BC_tissue | Kartti_2023 | 0.0594 | 0.0054 | 0 | 0.0162 | 0.0054 | 0 | 0 | 0.9782 | 0 | 0.073 | 0 | 0 | 0 | 0 |
| BC_tissue | Kartti_2023 | 0.0475 | 0.1755 | 0 | 0 | 0.1207 | 0 | 0 | 0.2048 | 0 | 0 | 0 | 0.7899 | 0 | 0 |
| BC_tissue | Kartti_2023 | 0.2294 | 0.105 | 0.1994 | 0.012 | 0 | 0 | 0.6462 | 3.3916 | 0 | 0 | 0 | 0 | 0 | 0 |
| BC_tissue | Kartti_2023 | 0.2103 | 0.1456 | 0 | 0 | 0 | 0 | 0 | 4.5013 | 0 | 0.8342 | 0 | 0 | 0 | 0.0832 |
| BC_tissue | Kartti_2023 | 0.1595 | 0.9108 | 0.0261 | 0.0348 | 0.087 | 0 | 0 | 0 | 0 | 0.1276 | 0 | 0.058 | 0 | 0 |
| BC_tissue | Kartti_2023 | 0.1433 | 2.0104 | 0.0139 | 0 | 0.0462 | 0 | 0 | 0.7649 | 0 | 0.3605 | 0 | 0.0046 | 0.0023 | 0 |
| BC_tissue | Kartti_2023 | 0.0469 | 0.1905 | 0.0028 | 0 | 0 | 0 | 0.0414 | 0.5605 | 0.37 | 0.2264 | 0.0166 | 0.0359 | 0 | 0 |
| BC_tissue | Kartti_2023 | 0.149 | 0.8539 | 0 | 0.2063 | 0 | 0 | 0 | 1.1204 | 0.3954 | 1.2751 | 0 | 0.043 | 0 | 0.0086 |
| BC_tissue | Kartti_2023 | 0.2393 | 0.1038 | 0 | 0 | 0 | 0 | 0 | 1.0062 | 0.0865 | 0.3806 | 0.0058 | 0.0519 | 0 | 0 |
| BC_tissue | Kartti_2023 | 0.4304 | 0.2681 | 0.0776 | 0 | 0.9736 | 0.1482 | 0.2011 | 2.1202 | 0 | 0.5009 | 0 | 0.2293 | 0.0564 | 0 |
| BC_tissue | Kartti_2023 | 0.6166 | 0.5695 | 0.0594 | 0.043 | 0 | 0 | 0 | 1.0632 | 0 | 1.3807 | 0 | 0.0266 | 0 | 0 |
| BC_tissue | Kartti_2023 | 0.0712 | 0 | 0.038 | 0.121 | 0 | 0 | 0 | 6.0027 | 0.0142 | 0.465 | 0 | 0.0261 | 0 | 0 |
| BC_tissue | Kartti_2023 | 0.1804 | 0.1928 | 0.0539 | 0.0829 | 0 | 0 | 0 | 2.2351 | 0 | 0.1348 | 0.1493 | 0.0311 | 0.027 | 0 |
| BC_tissue | Kartti_2023 | 1.1033 | 1.5091 | 0.2599 | 0.0017 | 0.2834 | 0 | 0 | 0.4628 | 0 | 0.2515 | 0 | 0.1744 | 0 | 0.0771 |
| BC_tissue | Kartti_2023 | 0.0644 | 0.2457 | 0 | 0 | 0 | 0 | 0 | 7.3616 | 0.143 | 0.1389 | 0.002 | 0.2879 | 0 | 0 |
| BC_tissue | Kartti_2023 | 0 | 0.0227 | 0 | 0.1189 | 0 | 0 | 0 | 1.6198 | 0 | 0 | 0.0312 | 0.1019 | 0 | 0 |
| BC_tissue | Kartti_2023 | 0.3687 | 0.7544 | 0.1674 | 0 | 0 | 0 | 0 | 3.6722 | 0 | 0 | 0 | 0.015 | 0 | 0.237 |
| BC_tissue | Kartti_2023 | 0.6731 | 0.419 | 0.0557 | 0 | 0 | 0 | 0 | 3.0822 | 0 | 0.2407 | 0 | 0.2563 | 0 | 0 |
| BC_tissue | Kartti_2023 | 0.0086 | 0.2251 | 0.1959 | 0.0533 | 0 | 0 | 0 | 0.8008 | 0 | 0 | 0.0086 | 0.0017 | 0 | 0 |
| BC_tissue | Kartti_2023 | 0.1556 | 0.6683 | 0.0192 | 0 | 0 | 0 | 0 | 1.3885 | 0 | 0.0096 | 0 | 0.2343 | 0 | 0 |
| BC_tissue | Kartti_2023 | 0.0955 | 0.0145 | 0 | 0.0058 | 0 | 0 | 0 | 0.3934 | 0 | 0 | 0 | 0.1417 | 0 | 0 |
| BC_tissue | Kartti_2023 | 0.4427 | 1.7837 | 0.7177 | 0 | 0 | 0 | 0 | 1.9383 | 0 | 0 | 0.5068 | 0.5631 | 0 | 0 |
| BC_tissue | German_2023 | 2.326 | 0 | 0 | 0.1641 | 0 | 0 | 0 | 0 | 0 | 0 | 0 | 0 | 0.0014 | 0.0087 |
| BC_tissue | German_2023 | 0.8113 | 0.0053 | 0 | 0.5311 | 0.6512 | 0 | 0 | 0 | 0 | 0 | 0 | 0 | 0.3096 | 0.0027 |
| BC_tissue | German_2023 | 0.0202 | 0 | 0 | 0.5146 | 0 | 0 | 0 | 0 | 0.3733 | 0 | 0 | 0 | 0.0101 | 0 |
| BC_tissue | German_2023 | 1.0872 | 0 | 0 | 0.1746 | 0 | 0 | 0 | 0 | 0 | 0 | 0 | 0 | 0.0044 | 0.0262 |
| BC_tissue | German_2023 | 1.1485 | 0.0254 | 0 | 0.0634 | 0 | 0 | 0 | 0 | 0 | 0 | 0 | 0 | 0 | 0.0003 |
| BC_tissue | German_2023 | 1.2002 | 0 | 0 | 1.2736 | 0 | 0 | 0 | 0 | 0 | 0 | 0 | 0 | 0 | 0.0167 |
| BC_tissue | German_2023 | 0.083 | 0 | 0 | 0.1834 | 0 | 0 | 0 | 0 | 0 | 0 | 0 | 0 | 0 | 0.1026 |
| BC_tissue | German_2023 | 0.2365 | 0.0003 | 0 | 0.0165 | 0 | 0 | 0 | 0 | 0.0003 | 0 | 0 | 0 | 0.0234 | 0.198 |
| BC_tissue | German_2023 | 0.842 | 0.1212 | 0.0093 | 0.0235 | 0 | 0 | 0 | 0 | 0.0004 | 0 | 0 | 0.0852 | 0.0025 | 0.0595 |
| BC_tissue | German_2023 | 2.5176 | 0.0054 | 0 | 0.189 | 0 | 0 | 0 | 0 | 0.6756 | 0 | 0 | 0 | 0.0063 | 0 |
| BC_tissue | German_2023 | 0.1601 | 0 | 0 | 0 | 0 | 0 | 0 | 0 | 0 | 0.0412 | 0 | 0 | 0.0121 | 0 |
| BC_tissue | German_2023 | 0.745 | 0 | 0 | 4.2594 | 0 | 0 | 0 | 0 | 0.5998 | 0 | 0 | 0 | 0 | 0.0094 |
| BC_tissue | German_2023 | 1.076 | 0 | 0 | 1.5766 | 0 | 0 | 0 | 0 | 0.0107 | 0 | 0 | 0 | 0 | 0.0107 |
| BC_tissue | German_2023 | 0.262 | 0.043 | 0 | 0.1003 | 0 | 0 | 0 | 0 | 0.001 | 0 | 0 | 0 | 0 | 0.0058 |
| BC_tissue | German_2023 | 0.1683 | 0 | 0 | 0 | 0 | 0 | 0 | 0 | 0 | 0 | 0 | 0 | 0 | 0.0259 |
| BC_tissue | German_2023 | 0.5228 | 0 | 0 | 0.057 | 0 | 0 | 0 | 0 | 3.8019 | 0 | 0 | 0 | 0 | 0 |
| BC_tissue | German_2023 | 4.4708 | 0.0827 | 0 | 0.0399 | 0.0029 | 0 | 0 | 0 | 0 | 0 | 0 | 0 | 0.0228 | 0.0599 |
| BC_tissue | German_2023 | 0.7556 | 0 | 0 | 0.536 | 0 | 0 | 0 | 0 | 0 | 0 | 0 | 0 | 0 | 0 |
| BC_tissue | German_2023 | 8.0762 | 0 | 0 | 0 | 0 | 0 | 0 | 0 | 2.9358 | 0 | 0 | 0 | 0.0028 | 0.0028 |
| BC_tissue | German_2023 | 0.4407 | 0 | 0 | 0.3815 | 0.0024 | 0.7641 | 0 | 0 | 0 | 0 | 0 | 0 | 0.0047 | 0.0012 |
| BC_tissue | German_2023 | 2.3327 | 0.397 | 0 | 0 | 0 | 0 | 0 | 0 | 0 | 0 | 0.554 | 0 | 0 | 0 |
| BC_tissue | German_2023 | 1.0178 | 0 | 0 | 42.4696 | 0 | 0 | 0 | 0 | 0 | 0 | 0 | 0 | 0 | 0.0025 |
| BC_tissue | German_2023 | 0.0654 | 0 | 0 | 55.2003 | 0 | 0 | 0 | 0 | 0 | 0 | 0 | 0 | 0 | 0.0008 |
| BC_tissue | German_2023 | 0.3725 | 0.0045 | 0 | 0.0102 | 0 | 0 | 0 | 0 | 0 | 0 | 0 | 0 | 0.0003 | 0 |
| BC_tissue | German_2023 | 0.6388 | 0.4819 | 0 | 0.0261 | 0 | 0 | 0 | 0 | 0.2988 | 0 | 0 | 0 | 0 | 0 |
| BC_tissue | German_2023 | 0.7209 | 0.0335 | 0 | 0.3521 | 0 | 0 | 0 | 0 | 0 | 0 | 0 | 0 | 0.1006 | 0.0168 |
| BC_tissue | German_2023 | 0.9711 | 0 | 0 | 0.6404 | 0.0003 | 0.0006 | 0 | 0 | 0 | 0 | 0 | 0 | 1.1092 | 0 |
| BC_tissue | German_2023 | 9.733 | 0.1013 | 0 | 0.0044 | 0 | 0 | 0 | 0 | 0.4979 | 0 | 0 | 0 | 0.0044 | 0 |
| BC_tissue | German_2023 | 0.7773 | 0.0551 | 0 | 0.0591 | 0 | 0 | 0 | 0 | 2.4645 | 0.0013 | 0 | 0 | 0.0289 | 0.0026 |
| BC_tissue | German_2023 | 2.0973 | 0 | 0 | 0.4661 | 0 | 0 | 0 | 0 | 0 | 0 | 0 | 0 | 0.0055 | 0.2184 |
| BC_adjacent | Hoskinson_2022 | 0.0055 | 0 | 0.0055 | 0 | 0 | 0 | 0 | 0 | 0 | 0 | 0 | 0 | 0 | 0 |
| BC_adjacent | Hoskinson_2022 | 0.026 | 0 | 0.0015 | 0 | 0.0031 | 0 | 0 | 0 | 10.4421 | 0 | 0.0031 | 0 | 1.2698 | 0 |
| BC_adjacent | Hoskinson_2022 | 0.0677 | 0 | 0.0038 | 0 | 0 | 0 | 0 | 0 | 1.9728 | 1.2657 | 0 | 0 | 0 | 0 |
| BC_adjacent | Hoskinson_2022 | 0.0195 | 0 | 0.1598 | 0 | 0.0013 | 0 | 0 | 0 | 0 | 0 | 1.7604 | 0 | 0 | 0 |
| BC_adjacent | Hoskinson_2022 | 0.0043 | 0.124 | 0.7656 | 0 | 10.1155 | 0 | 0 | 0 | 1.0424 | 2.0329 | 0 | 0 | 0 | 0 |
| BC_adjacent | Hoskinson_2022 | 2.3862 | 0 | 20.4207 | 0 | 0 | 0 | 24.7995 | 0 | 0 | 1.6905 | 0 | 0 | 0 | 0 |
| BC_adjacent | Hoskinson_2022 | 0.5662 | 0.002 | 0.1431 | 0 | 0.002 | 0 | 0.0041 | 0 | 0 | 1.3735 | 0 | 0 | 0 | 0.0102 |
| BC_adjacent | Hoskinson_2022 | 0.5848 | 0.1263 | 9.0582 | 0 | 0 | 0 | 0.0019 | 0 | 0 | 0 | 0 | 0 | 0 | 0 |
| BC_adjacent | Hoskinson_2022 | 6.4775 | 0 | 18.6199 | 0 | 0 | 0 | 0 | 0 | 0 | 1.6042 | 0 | 0 | 0 | 0 |
| BC_adjacent | Hoskinson_2022 | 0.4038 | 0 | 1.237 | 0 | 0 | 0 | 0 | 0 | 6.2304 | 0 | 0 | 0 | 0 | 0 |
| BC_adjacent | Hoskinson_2022 | 1.2222 | 0 | 1.5837 | 0 | 0 | 0 | 2.0241 | 0 | 1.2772 | 0.0018 | 0 | 2.8939 | 0 | 0 |
| BC_adjacent | Hoskinson_2022 | 0.4092 | 0 | 6.9208 | 0 | 9.548 | 0 | 3.6976 | 0 | 0 | 0 | 0.0015 | 0 | 0 | 0 |
| BC_adjacent | Hoskinson_2022 | 1.1226 | 0 | 0.007 | 0 | 3.2426 | 0 | 0 | 0 | 0 | 2.4143 | 0 | 0 | 0 | 0 |
| BC_adjacent | Hoskinson_2022 | 1.1775 | 0 | 1.9041 | 0 | 0 | 0 | 0 | 0 | 0 | 0 | 0 | 0 | 0 | 0 |
| BC_adjacent | Hoskinson_2022 | 2.6222 | 0 | 0.6384 | 0 | 0 | 0 | 0 | 0 | 0 | 0 | 0.0017 | 0 | 0 | 0 |
| BC_adjacent | Hoskinson_2022 | 4.5148 | 0 | 7.6128 | 0 | 0 | 0 | 0 | 0 | 0 | 1.148 | 0 | 0 | 0 | 0 |
| BC_adjacent | Hoskinson_2022 | 0.0413 | 0 | 0.7032 | 0 | 0 | 0 | 0 | 0 | 0.0032 | 0 | 0 | 0 | 0 | 0 |
| BC_adjacent | Hoskinson_2022 | 0.0512 | 0 | 0.425 | 0 | 6.9325 | 0 | 0.0057 | 0 | 0 | 0 | 0 | 0 | 0 | 0 |
| BC_adjacent | Hoskinson_2022 | 0.0113 | 0 | 3.2305 | 0 | 4.6138 | 0 | 0 | 0 | 0 | 0.0014 | 0 | 0 | 0 | 0 |
| BC_adjacent | Hoskinson_2022 | 0.1595 | 0 | 5.8859 | 0 | 0.028 | 0.0016 | 12.0038 | 0 | 0 | 0 | 0 | 0 | 0 | 0 |
| BC_adjacent | Hoskinson_2022 | 0.2071 | 0 | 8.4571 | 0 | 0.0222 | 0 | 0 | 0 | 0.0018 | 0 | 0.0037 | 0 | 0 | 0 |
| BC_adjacent | Hoskinson_2022 | 0.0141 | 0 | 4.76 | 0 | 0.0064 | 0 | 0.0038 | 0 | 0 | 0 | 0 | 0 | 0 | 0 |
| BC_adjacent | Hoskinson_2022 | 0.0108 | 0 | 0.4779 | 0 | 2.1652 | 0 | 0.8279 | 0 | 0 | 0 | 0 | 0 | 0 | 0 |
| BC_adjacent | Hoskinson_2022 | 0.2912 | 0 | 5.979 | 0 | 0.1262 | 0 | 13.2429 | 0.0078 | 0.0436 | 0 | 0 | 0 | 0 | 0 |
| BC_adjacent | Hoskinson_2022 | 0 | 0 | 0.0288 | 0 | 0 | 0 | 0 | 0 | 0 | 0 | 0 | 0 | 0 | 0 |
| BC_adjacent | Hoskinson_2022 | 0.2074 | 0.001 | 0.0114 | 0 | 0.0156 | 0 | 0.001 | 0 | 0 | 0 | 0 | 0 | 0 | 0 |
| BC_adjacent | Hoskinson_2022 | 0.0038 | 0 | 0.0881 | 0 | 0.0063 | 0 | 0.0013 | 0 | 0.0025 | 0.2064 | 0 | 0 | 0 | 0 |
| BC_adjacent | Hoskinson_2022 | 0.8382 | 0 | 0.0216 | 0 | 0 | 0 | 0 | 0 | 0.0108 | 0 | 0 | 0 | 0 | 0 |
| BC_adjacent | Hoskinson_2022 | 0.0721 | 0 | 0.8286 | 0 | 0.0076 | 0 | 0 | 0 | 0 | 0 | 0 | 0 | 0 | 0 |
| BC_adjacent | Hoskinson_2022 | 0.1887 | 0 | 0 | 0 | 0.0033 | 0 | 0 | 0 | 0 | 10.2937 | 0.0016 | 0 | 0 | 0 |
| BC_adjacent | Hoskinson_2022 | 0.0229 | 2.2701 | 0.7531 | 0 | 81.403 | 0 | 1.2287 | 0 | 0 | 0 | 0 | 0 | 0 | 0 |
| BC_adjacent | Hoskinson_2022 | 0.0676 | 0 | 1.613 | 0 | 0.0049 | 0 | 0 | 0 | 0 | 0.0016 | 0 | 0 | 0 | 0 |
| BC_adjacent | Hoskinson_2022 | 0.0349 | 0 | 3.7779 | 0 | 11.6081 | 0 | 0.8108 | 0 | 0 | 0 | 0 | 0 | 0 | 0 |
| BC_adjacent | Hoskinson_2022 | 0.0057 | 0 | 6.7409 | 0 | 0 | 0 | 0 | 0 | 0 | 0 | 0 | 0 | 0 | 0 |
| BC_adjacent | Hoskinson_2022 | 0.0028 | 0 | 0.3563 | 0 | 4.8777 | 0 | 0.263 | 0 | 0 | 0.0557 | 0 | 0 | 0 | 0 |
| BC_adjacent | Hoskinson_2022 | 0.2264 | 0 | 25.1511 | 0 | 0.0125 | 0 | 0 | 0 | 0 | 0 | 0 | 0 | 0 | 0 |
| BC_adjacent | Hoskinson_2022 | 0.1622 | 0.0027 | 0 | 0 | 0.0239 | 0 | 0.0027 | 0 | 0 | 0 | 0 | 0 | 0 | 0 |
| BC_adjacent | Hoskinson_2022 | 0.0024 | 0.0024 | 17.0719 | 0.0024 | 0 | 0 | 0 | 0 | 0 | 0 | 0 | 0 | 0 | 0 |
| BC_adjacent | Hoskinson_2022 | 0.0185 | 0.0018 | 5.6056 | 0 | 0.0037 | 0 | 0 | 0 | 0 | 0 | 0 | 0 | 0 | 0 |
| BC_adjacent | Hoskinson_2022 | 0.223 | 0.0058 | 0.0116 | 0 | 1.9114 | 0 | 0 | 0 | 0 | 0 | 0 | 0 | 0 | 0 |
| BC_adjacent | Hoskinson_2022 | 0.0014 | 0.0029 | 1.1991 | 0 | 0 | 0 | 0.0014 | 0 | 0 | 0 | 0 | 0.0014 | 0 | 0 |
| BC_adjacent | Hoskinson_2022 | 0.1774 | 0 | 14.6228 | 0 | 0 | 0 | 4.8724 | 0 | 0.0038 | 0.0019 | 0 | 0 | 0 | 0 |
| BC_adjacent | Hoskinson_2022 | 0.1818 | 0 | 33.5567 | 0 | 0.0022 | 0.0044 | 0.0022 | 0 | 0 | 0 | 0 | 0 | 0 | 0 |
| BC_adjacent | Hoskinson_2022 | 0.0534 | 0.0018 | 6.4876 | 0 | 7.9383 | 0 | 0.0018 | 0 | 0 | 0 | 0 | 0 | 0 | 0 |
| BC_adjacent | Hoskinson_2022 | 0.568 | 0 | 0 | 0 | 0.0222 | 0 | 0.0074 | 0 | 0 | 0 | 0 | 0 | 0 | 0 |
| BC_adjacent | Hoskinson_2022 | 1.1588 | 1.3225 | 0 | 0 | 0.6305 | 0 | 0 | 0 | 0 | 0 | 0 | 0 | 0 | 0 |
| BC_adjacent | Hoskinson_2022 | 0 | 0 | 7.4622 | 0 | 0.0023 | 0 | 0 | 0 | 0 | 0 | 0 | 0 | 0 | 0 |
| BC_adjacent | Hoskinson_2022 | 0.0204 | 0 | 0.0167 | 0 | 32.426 | 0 | 0 | 0 | 0 | 0 | 0 | 0 | 0 | 0 |
| BC_adjacent | Hoskinson_2022 | 0.0611 | 0 | 14.1209 | 0 | 0 | 0.0018 | 0 | 0 | 0.0072 | 0 | 0 | 0 | 0 | 0 |
| BC_adjacent | Esposito_2022 | 0.9873 | 36.8283 | 0.0125 | 0 | 0 | 0.3249 | 0 | 0 | 0 | 0 | 0 | 0.025 | 0 | 0 |
| BC_adjacent | Esposito_2022 | 24.0902 | 4.0875 | 0.0136 | 0.8691 | 0 | 5.6763 | 0 | 0 | 0 | 0 | 0 | 0 | 0 | 0.8691 |
| BC_adjacent | Esposito_2022 | 23.6458 | 10.9375 | 0 | 0 | 0 | 0.5208 | 0 | 0 | 0 | 0 | 0 | 0 | 0 | 0 |
| BC_adjacent | Esposito_2022 | 33.0132 | 4.1444 | 0 | 0.3735 | 0 | 3.593 | 0 | 0 | 0 | 0 | 0 | 0 | 0 | 1.8143 |
| BC_adjacent | Esposito_2022 | 12.1007 | 5.8567 | 0 | 2.4685 | 0.1936 | 4.1626 | 0 | 0 | 0 | 0 | 0 | 0 | 0 | 1.3069 |
| BC_adjacent | Esposito_2022 | 2.5418 | 18.5756 | 0.007 | 0 | 0 | 0.8914 | 0 | 0.0023 | 0 | 0 | 0 | 0 | 0 | 0.6102 |
| BC_adjacent | Esposito_2022 | 3.0561 | 5.5451 | 0 | 0 | 0 | 0.126 | 0 | 0 | 0 | 0 | 0 | 0 | 0 | 0 |
| BC_adjacent | Esposito_2022 | 22.3793 | 5.2076 | 0 | 0 | 0 | 6.1504 | 0 | 0 | 0 | 0 | 0 | 0 | 0 | 1.0325 |
| BC_adjacent | Esposito_2022 | 15.208 | 2.1664 | 0 | 2.383 | 0 | 3.5962 | 0 | 0 | 0 | 0 | 0 | 0 | 0 | 0 |
| BC_adjacent | Esposito_2022 | 24.6239 | 6.3691 | 0.0502 | 0.4514 | 0.0502 | 3.2598 | 0 | 0 | 0.4012 | 0 | 0 | 0 | 0 | 1.5045 |
| BC_adjacent | Esposito_2022 | 28.9863 | 5.6986 | 0 | 2.5753 | 0 | 4.4932 | 0 | 0 | 0 | 0 | 0 | 0 | 0 | 1.4247 |
| BC_adjacent | Esposito_2022 | 27.0101 | 7.1543 | 0 | 3.8175 | 0 | 3.5913 | 0 | 0 | 0 | 0 | 0 | 0 | 0 | 0.2451 |
| BC_adjacent | Esposito_2022 | 78.2609 | 0.0607 | 0 | 0 | 0 | 0.0809 | 0 | 0 | 0 | 0 | 0 | 0 | 0 | 0 |
| BC_adjacent | Esposito_2022 | 8.185 | 16.4355 | 0 | 0 | 0 | 0.9108 | 0 | 0 | 0.4349 | 0 | 0 | 0.0041 | 0 | 0 |
| BC_adjacent | Esposito_2022 | 20.8149 | 4.7708 | 0 | 1.7317 | 0 | 1.3243 | 0 | 0 | 0.3905 | 0 | 0 | 0 | 0 | 0 |
| BC_adjacent | Esposito_2022 | 17.6572 | 4.878 | 0 | 6.2178 | 0 | 9.5156 | 0 | 0 | 0 | 0 | 0 | 0 | 0 | 0.0344 |
| BC_adjacent | Esposito_2022 | 28.6436 | 3.9711 | 0.0045 | 0 | 0.1092 | 0.3639 | 0 | 0 | 0.1547 | 0.0045 | 0 | 0 | 0 | 0.0728 |
| BC_adjacent | Esposito_2022 | 12.5957 | 0.0398 | 0 | 0 | 1.4614 | 2.1573 | 0 | 0 | 0 | 0 | 0 | 0.0199 | 0 | 0 |
| BC_adjacent | Esposito_2022 | 13.6911 | 14.748 | 0 | 5.6748 | 0 | 9.8049 | 0 | 0 | 0 | 0 | 0 | 0 | 0 | 0 |
| BC_adjacent | Esposito_2022 | 37.4868 | 4.5008 | 0 | 1.6916 | 0.6343 | 3.4436 | 0 | 0 | 0 | 0 | 0 | 0 | 0 | 2.0088 |
| BC_adjacent | Esposito_2022 | 33.0614 | 3.8868 | 0 | 0.3359 | 0 | 3.8868 | 0 | 0 | 0.096 | 0 | 0 | 0 | 0 | 0.048 |
| BC_adjacent | Esposito_2022 | 41.9192 | 2.6936 | 0 | 0.8418 | 0 | 2.3569 | 0 | 0 | 0 | 0 | 0 | 0 | 0 | 0.1684 |
| BC_adjacent | Esposito_2022 | 1.1715 | 3.7723 | 0.0117 | 0 | 0 | 0.2929 | 0 | 0 | 0 | 0 | 0 | 0 | 0 | 0.0586 |
| BC_adjacent | Esposito_2022 | 3.2217 | 5.478 | 0 | 0 | 0.7923 | 6.9734 | 0 | 0 | 0 | 0 | 0 | 0 | 0 | 0 |
| BC_adjacent | Esposito_2022 | 5.3799 | 4.3704 | 0.006 | 0 | 0 | 0.0846 | 0 | 0 | 0 | 0 | 0 | 0.006 | 0 | 0 |
| BC_adjacent | Esposito_2022 | 30.2271 | 5.8784 | 0 | 0 | 0.2672 | 2.171 | 0 | 0 | 0.0668 | 0 | 0 | 0 | 0 | 1.1022 |
| BC_adjacent | Esposito_2022 | 25.8769 | 3.7723 | 0 | 0 | 0 | 2.3163 | 0 | 0 | 0 | 0 | 0 | 0 | 0 | 0 |
| BC_adjacent | Esposito_2022 | 18.5676 | 6.6313 | 0 | 1.1052 | 0 | 2.9178 | 0 | 0 | 0 | 0 | 0 | 0 | 0 | 0 |
| BC_adjacent | Esposito_2022 | 14.4527 | 7.2502 | 0.0238 | 1.6933 | 0.1669 | 5.0799 | 0 | 0 | 0 | 0 | 0 | 0 | 0 | 0 |
| BC_adjacent | Esposito_2022 | 29.0785 | 4.8404 | 0 | 0.6454 | 0 | 5.3424 | 0 | 0 | 0 | 0 | 0 | 0 | 0 | 0 |
| BC_adjacent | Esposito_2022 | 15.1643 | 3.4485 | 0.0156 | 2.2419 | 0.1323 | 8.3061 | 0 | 0 | 0.1479 | 0 | 0 | 0.0078 | 0 | 0.506 |
| BC_adjacent | Esposito_2022 | 60.1816 | 5.2748 | 0 | 0 | 0 | 1.055 | 0 | 0 | 0 | 0 | 0 | 0 | 0 | 1.0304 |
| BC_adjacent | Esposito_2022 | 7.3434 | 0 | 0 | 0 | 0 | 1.9984 | 0 | 0 | 0 | 0 | 0 | 0 | 0 | 0 |
| BC_adjacent | Esposito_2022 | 31.5654 | 10.0941 | 0 | 0.834 | 0.4919 | 2.5449 | 0 | 0 | 0 | 0 | 0 | 0 | 0 | 0.556 |
| BC_adjacent | Kartti_2023 | 0.1992 | 0.2231 | 0 | 0.0664 | 0.0266 | 0 | 0 | 0.3904 | 0.0159 | 0.0903 | 0 | 0 | 0 | 0 |
| BC_adjacent | Kartti_2023 | 0.0765 | 0.379 | 0 | 0 | 0 | 0 | 0.1348 | 0.594 | 0.0474 | 0.0146 | 0 | 0.0182 | 0 | 0 |
| BC_adjacent | Kartti_2023 | 0.0552 | 0.1472 | 0.0074 | 0 | 0 | 0 | 0 | 0.6623 | 0 | 0.0037 | 0 | 0.3716 | 0 | 0 |
| BC_adjacent | Kartti_2023 | 0.0082 | 0.4254 | 0 | 0.0355 | 0 | 0.0109 | 0 | 0.7227 | 0.1091 | 0.0627 | 0 | 0 | 0 | 0 |
| BC_adjacent | Kartti_2023 | 0.0224 | 0.0583 | 0.0269 | 0 | 0 | 0 | 0 | 0.6588 | 0 | 0 | 0 | 0 | 0 | 0 |
| BC_adjacent | Kartti_2023 | 0.1827 | 0.1428 | 0 | 0 | 0 | 0.1533 | 0 | 1.088 | 0 | 0.0336 | 0 | 0 | 0 | 0 |
| BC_adjacent | Kartti_2023 | 0.0153 | 0 | 0 | 0.2456 | 0 | 0 | 0 | 0.4221 | 0 | 0 | 0 | 0 | 0 | 0 |
| BC_adjacent | Kartti_2023 | 0.106 | 0.1349 | 0 | 0.0337 | 0 | 0 | 0 | 1.2383 | 0 | 0 | 0 | 0.0578 | 0 | 0 |
| BC_adjacent | Kartti_2023 | 0.0039 | 0.2901 | 0.0314 | 0.0039 | 0 | 0.0039 | 0 | 0.4666 | 0.0314 | 0 | 0 | 0.0039 | 0 | 0 |
| BC_adjacent | Kartti_2023 | 0.0992 | 0.124 | 0 | 0.0135 | 0 | 0.0113 | 0 | 0.76 | 0.018 | 0.0135 | 0 | 0.009 | 0 | 0 |
| BC_adjacent | Kartti_2023 | 0.0651 | 0.0566 | 0 | 0.0255 | 0.1754 | 0 | 0 | 0.5544 | 0 | 0 | 0 | 0 | 0 | 0 |
| BC_adjacent | Kartti_2023 | 0.4439 | 0.0631 | 0.0243 | 0 | 0 | 0.08 | 0 | 2.6853 | 0.0679 | 0.2911 | 0.1213 | 0.0024 | 0 | 0 |
| BC_adjacent | Kartti_2023 | 0.3295 | 0.2508 | 0 | 0 | 0 | 0 | 0 | 3.2659 | 0 | 0 | 0 | 0 | 0 | 0 |
| BC_adjacent | Kartti_2023 | 0.4215 | 0.5826 | 0 | 0.062 | 0 | 0 | 0 | 0.3099 | 0 | 0 | 0 | 0.0403 | 0 | 0 |
| BC_adjacent | Kartti_2023 | 0.0672 | 0.039 | 0 | 0.0065 | 0 | 0 | 0 | 0.0564 | 0 | 0 | 0 | 0 | 0 | 0 |
| BC_adjacent | Kartti_2023 | 0 | 0.0288 | 0 | 0 | 0 | 0 | 0 | 0.5696 | 0 | 0 | 0 | 0 | 0 | 0 |
| BC_adjacent | Kartti_2023 | 0.03 | 0 | 0 | 0.018 | 0.018 | 0 | 0 | 1.2798 | 0 | 0.003 | 0 | 0.012 | 0 | 0 |
| BC_adjacent | Kartti_2023 | 0.1811 | 0.058 | 0 | 0 | 0 | 0 | 0 | 1.1483 | 0 | 0 | 0 | 0 | 0 | 0 |
| BC_adjacent | Kartti_2023 | 0.2609 | 0.2721 | 0 | 0.1851 | 0 | 0 | 0 | 0.0757 | 0 | 0.143 | 0 | 0.0617 | 0 | 0 |
| BC_adjacent | Kartti_2023 | 0.162 | 0.953 | 0 | 0 | 0 | 0 | 0 | 2.9827 | 0.1923 | 0.8377 | 0.0906 | 0.0247 | 0 | 0 |
| BC_adjacent | Kartti_2023 | 0.1426 | 0.3772 | 0.1702 | 0.1173 | 0 | 0 | 0 | 1.5341 | 0 | 0.3427 | 0 | 0 | 0 | 0.0184 |
| BC_adjacent | Kartti_2023 | 0.2238 | 0.1766 | 0.07 | 0 | 0.0198 | 0 | 0 | 0.5845 | 0.0411 | 0.685 | 0 | 0.0167 | 0 | 0 |
| BC_adjacent | Kartti_2023 | 0.4593 | 0.3099 | 0 | 0 | 0.0149 | 0 | 0 | 1.7047 | 0.0597 | 0.7188 | 0 | 0.0635 | 0 | 0 |
| BC_adjacent | Kartti_2023 | 0.058 | 0.1541 | 0.0491 | 0.0893 | 0 | 0 | 0 | 0.259 | 0.0246 | 0.9645 | 0 | 0.0982 | 0 | 0 |
| BC_adjacent | Kartti_2023 | 0.068 | 4.6846 | 0 | 0.7256 | 1.5472 | 0 | 0 | 0 | 0 | 2.4842 | 0 | 0 | 0 | 0 |
| BC_adjacent | Kartti_2023 | 0.0644 | 0.0309 | 0.0103 | 0 | 0 | 0 | 0 | 0.7495 | 0.2447 | 0.9839 | 0.018 | 0.0103 | 0 | 0 |
| BC_adjacent | Kartti_2023 | 2.0395 | 0.9406 | 0.0239 | 0.0299 | 0 | 0 | 0 | 1.1108 | 0 | 1.6483 | 0.006 | 0.5166 | 0 | 0 |
| BC_adjacent | Kartti_2023 | 0.0017 | 0.7772 | 0 | 0 | 0 | 0 | 0 | 0.5343 | 0.0711 | 0.6089 | 0.2325 | 0 | 0 | 0 |
| BC_adjacent | Kartti_2023 | 0.0842 | 0.0936 | 0 | 0.0047 | 0 | 0 | 0 | 2.7251 | 0 | 0 | 0.1497 | 0.2152 | 0 | 0 |
| BC_adjacent | Kartti_2023 | 0.0197 | 0.0329 | 0 | 0 | 0 | 0 | 0 | 1.356 | 0 | 0.1316 | 0.0461 | 0.0724 | 0 | 0.0066 |
| BC_adjacent | Kartti_2023 | 0.1775 | 0.7909 | 0 | 0 | 0 | 0 | 0 | 1.3046 | 0.3954 | 1.8028 | 0.3612 | 0.1121 | 0 | 0 |
| BC_adjacent | Kartti_2023 | 0 | 0.0762 | 0.0035 | 0.0053 | 0 | 0 | 0 | 0.8564 | 0 | 0 | 0 | 0.0089 | 0 | 0 |
| BC_adjacent | Kartti_2023 | 0.0092 | 0.1382 | 0 | 0.2641 | 0.1628 | 0 | 0 | 1.8674 | 0 | 0.1413 | 0 | 0 | 0 | 0 |
| BC_adjacent | Kartti_2023 | 0.0133 | 0.1889 | 0.0033 | 0 | 0 | 0.0033 | 0 | 1.5114 | 0 | 0.0199 | 0.0099 | 0.0365 | 0 | 0 |
| BC_adjacent | Kartti_2023 | 0.6993 | 0.6672 | 0.0046 | 0 | 0 | 0 | 0 | 0.282 | 0.0321 | 0.376 | 0 | 0 | 0 | 0 |
| BC_adjacent | Kartti_2023 | 0.2651 | 0.0826 | 0.0224 | 0.0878 | 0 | 0 | 0 | 1.2533 | 0.0155 | 0 | 0 | 0 | 0 | 0 |
| BC_adjacent | Kartti_2023 | 0.1752 | 0.4111 | 0.0017 | 0.0434 | 0 | 0 | 0 | 1.8317 | 0.2342 | 1.0668 | 0 | 0.1145 | 0 | 0 |
| BC_adjacent | Kartti_2023 | 0 | 0.042 | 0 | 0.034 | 0 | 0 | 0 | 2.032 | 0 | 0 | 0 | 0.0939 | 0 | 0 |
| BC_adjacent | Kartti_2023 | 0.0034 | 0.0448 | 0.2793 | 0 | 0 | 0 | 0 | 0.4725 | 0 | 0 | 0 | 0 | 0 | 0 |
| BC_adjacent | Kartti_2023 | 0.5648 | 0.4287 | 0.5892 | 0.1808 | 0 | 0 | 0 | 2.0946 | 0.7009 | 0.0264 | 0 | 1.0849 | 0 | 0 |
| BC_adjacent | Kartti_2023 | 0.5648 | 3.0352 | 0.3905 | 0 | 0.4857 | 0.1936 | 0 | 0 | 0 | 0.1436 | 0.1 | 0.184 | 0 | 0 |
| BC_adjacent | Kartti_2023 | 0.0261 | 1.3869 | 0.0681 | 0 | 0 | 0 | 0 | 4.1045 | 0.1984 | 0.4549 | 0 | 0.4229 | 0 | 0 |
| BC_adjacent | Kartti_2023 | 0.0406 | 0 | 0.0076 | 0 | 0 | 0 | 0 | 1.1856 | 0 | 0.0305 | 0 | 0 | 0 | 0 |
| BC_adjacent | Kartti_2023 | 0.5927 | 1.0892 | 0.2027 | 0.1822 | 0 | 0.0462 | 0 | 2.5107 | 0 | 0.4105 | 0.0847 | 0.2669 | 0 | 0 |
| BC_adjacent | Kartti_2023 | 0.0032 | 0 | 0 | 0.0714 | 0 | 0 | 0 | 0.9603 | 0 | 0 | 0 | 0 | 0 | 0 |
| BC_adjacent | Kartti_2023 | 0.0269 | 0.8522 | 0 | 0 | 0 | 0 | 0 | 1.2244 | 2.7183 | 0 | 0 | 0 | 0 | 0.1482 |
| BC_adjacent | Kartti_2023 | 0.5954 | 0.792 | 0.0057 | 0.2481 | 0 | 0 | 0 | 1.0993 | 0.5382 | 0.1985 | 0 | 0 | 0 | 0 |
| BC_adjacent | Kartti_2023 | 0.1832 | 12.3586 | 0.056 | 0 | 0.1442 | 0 | 0 | 0.6326 | 0.0237 | 0.1272 | 0.1798 | 0.0746 | 0 | 0.1035 |
| BC_adjacent | Kartti_2023 | 0.2232 | 2.4316 | 0.1997 | 0 | 0 | 0 | 0 | 0 | 0 | 1.646 | 0.0308 | 0.21 | 0 | 0.022 |
| BC_adjacent | Kartti_2023 | 0.5238 | 0.748 | 3.7577 | 0.0067 | 0.1332 | 0 | 0 | 0 | 0.0577 | 0.2264 | 0 | 0 | 0 | 0 |
| BC_adjacent | Kartti_2023 | 1.5595 | 0.8639 | 0 | 0.2255 | 0.0156 | 0 | 0 | 1.6532 | 2.7843 | 0 | 0 | 0 | 0 | 0 |
| BC_adjacent | Kartti_2023 | 0.0937 | 0 | 0 | 0 | 0.0075 | 0 | 0 | 0.9932 | 0 | 0 | 0.0787 | 0.0075 | 0 | 0 |
| BC_adjacent | German_2023 | 1.4866 | 0 | 0 | 2.3451 | 0 | 0 | 0 | 0 | 0 | 0 | 0 | 0 | 0 | 1.5444 |
| BC_adjacent | German_2023 | 0.0075 | 0 | 0 | 0 | 0 | 0 | 0 | 0 | 0 | 0 | 0 | 0 | 0 | 0.0005 |
| BC_adjacent | German_2023 | 8.8063 | 0 | 0 | 0.0273 | 0 | 0 | 0 | 0 | 0 | 0 | 0 | 0 | 8.6754 | 0 |
| BC_adjacent | German_2023 | 0.3366 | 0 | 0 | 0.2555 | 0 | 0 | 0 | 0 | 0 | 0 | 0 | 0 | 0.0003 | 0.0003 |
| BC_adjacent | German_2023 | 7.2479 | 0.1786 | 0 | 0.5567 | 0 | 0 | 0 | 0 | 0 | 0 | 0 | 0 | 0.0053 | 0.0053 |
| BC_adjacent | German_2023 | 0.3888 | 0 | 0 | 0.1591 | 0 | 0 | 0 | 0 | 0.9868 | 0.1149 | 0 | 0 | 0.0118 | 0.0854 |
| BC_adjacent | German_2023 | 1.9664 | 0.001 | 0 | 0.001 | 0 | 0 | 0 | 0 | 0 | 0 | 0 | 0 | 0 | 0.001 |
| BC_adjacent | German_2023 | 1.2393 | 0 | 0 | 0.011 | 0 | 0 | 0 | 0 | 0 | 0 | 0 | 0 | 0.0014 | 0.0014 |
| BC_adjacent | German_2023 | 1.6914 | 0.0726 | 0 | 0.8768 | 0 | 0 | 0 | 0 | 0.0026 | 0.0026 | 0 | 0 | 0.0104 | 0 |
| BC_adjacent | German_2023 | 0.1129 | 0 | 0 | 0.5783 | 0 | 0 | 0 | 0 | 0.0015 | 0 | 0 | 0 | 0 | 0 |
| BC_adjacent | German_2023 | 1.0252 | 0 | 0 | 0.1216 | 0 | 0 | 0 | 0 | 0 | 0 | 0 | 0 | 0.0006 | 0.1036 |
| BC_adjacent | German_2023 | 0.0841 | 0.0132 | 0 | 0.1515 | 0 | 0 | 0 | 0 | 0.0012 | 0 | 0 | 0 | 0.006 | 0 |
| BC_adjacent | German_2023 | 0.0731 | 0 | 0 | 0.0563 | 0 | 0 | 0 | 0 | 0 | 0 | 0 | 0.0012 | 0 | 0.0072 |
| BC_adjacent | German_2023 | 0.4006 | 0.0025 | 0 | 0.0025 | 0 | 0 | 0 | 0 | 0 | 0 | 0 | 0 | 0.8671 | 0.0025 |
| BC_adjacent | German_2023 | 0.0021 | 0.0167 | 0 | 0.0151 | 0 | 0 | 0.0003 | 0 | 0 | 0 | 0 | 0 | 0 | 0.0003 |
| BC_adjacent | German_2023 | 0.0058 | 0 | 0 | 0.0712 | 0 | 0 | 0 | 0 | 0 | 0 | 0 | 0 | 0 | 0.1144 |
| BC_adjacent | German_2023 | 1.8154 | 0 | 0 | 0.3218 | 0 | 0.0005 | 0 | 0 | 0 | 0 | 0 | 0 | 0 | 0 |
| BC_adjacent | German_2023 | 0.5588 | 0.195 | 0 | 0.1488 | 0 | 0 | 0 | 0 | 0 | 0 | 0 | 0.0248 | 0 | 0.2622 |
| BC_adjacent | German_2023 | 0.793 | 0.0011 | 0 | 0.1815 | 0 | 0 | 0 | 0 | 0 | 0 | 0 | 0 | 0.0394 | 0.0004 |
| BC_adjacent | German_2023 | 0 | 0 | 0 | 0.0005 | 0 | 0 | 0 | 0 | 0 | 0 | 0 | 0 | 0 | 0.0011 |
| BC_adjacent | German_2023 | 0.2035 | 0 | 0 | 0.0734 | 0 | 0 | 0 | 0 | 0 | 0 | 0 | 0 | 0 | 0 |
| BC_adjacent | German_2023 | 0.6671 | 0 | 0 | 0.0009 | 0 | 0 | 0 | 0 | 0.0034 | 0 | 0 | 0 | 0.0034 | 0.0017 |
| BC_adjacent | German_2023 | 0.3917 | 0.4833 | 0.1236 | 0.0337 | 0 | 0 | 0 | 0 | 0.0026 | 0 | 0 | 0.0553 | 0 | 0.0017 |
| BC_adjacent | German_2023 | 0.0902 | 0 | 0 | 0.3867 | 0 | 0 | 0 | 0 | 0 | 0 | 0 | 0 | 0.0129 | 1.1601 |
| BC_adjacent | German_2023 | 6.4257 | 0.1865 | 0 | 0.4733 | 0 | 0 | 0 | 0 | 0.0574 | 0 | 0 | 0 | 0.2008 | 0.2869 |
| BC_adjacent | German_2023 | 6.9833 | 0.2601 | 0 | 0 | 0 | 0 | 0 | 0 | 0.0012 | 0 | 0 | 0 | 0 | 0 |

**Table S6B. The RF scores at the species level in both BC_tissue and BC_adjacent samples.**

| **Group** | **Cohort** | **RF score** |
| --- | --- | --- |
| BC_tissue | Hoskinson_2022 | 0.18 |
| BC_tissue | Hoskinson_2022 | 0.15 |
| BC_tissue | Hoskinson_2022 | 0.41 |
| BC_tissue | Hoskinson_2022 | 0.13 |
| BC_tissue | Hoskinson_2022 | 0.2 |
| BC_tissue | Hoskinson_2022 | 0.11 |
| BC_tissue | Hoskinson_2022 | 0.08 |
| BC_tissue | Hoskinson_2022 | 0.13 |
| BC_tissue | Hoskinson_2022 | 0.17 |
| BC_tissue | Hoskinson_2022 | 0.07 |
| BC_tissue | Hoskinson_2022 | 0.19 |
| BC_tissue | Hoskinson_2022 | 0.22 |
| BC_tissue | Hoskinson_2022 | 0.26 |
| BC_tissue | Hoskinson_2022 | 0.17 |
| BC_tissue | Hoskinson_2022 | 0.15 |
| BC_tissue | Hoskinson_2022 | 0.14 |
| BC_tissue | Hoskinson_2022 | 0.2 |
| BC_tissue | Hoskinson_2022 | 0.2 |
| BC_tissue | Hoskinson_2022 | 0.25 |
| BC_tissue | Hoskinson_2022 | 0.15 |
| BC_tissue | Hoskinson_2022 | 0.1 |
| BC_tissue | Hoskinson_2022 | 0.19 |
| BC_tissue | Hoskinson_2022 | 0.1 |
| BC_tissue | Hoskinson_2022 | 0.15 |
| BC_tissue | Hoskinson_2022 | 0.12 |
| BC_tissue | Hoskinson_2022 | 0.19 |
| BC_tissue | Hoskinson_2022 | 0.12 |
| BC_tissue | Hoskinson_2022 | 0.31 |
| BC_tissue | Hoskinson_2022 | 0.2 |
| BC_tissue | Hoskinson_2022 | 0.17 |
| BC_tissue | Hoskinson_2022 | 0.2 |
| BC_tissue | Hoskinson_2022 | 0.26 |
| BC_tissue | Hoskinson_2022 | 0.24 |
| BC_tissue | Hoskinson_2022 | 0.23 |
| BC_tissue | Hoskinson_2022 | 0.12 |
| BC_tissue | Hoskinson_2022 | 0.29 |
| BC_tissue | Hoskinson_2022 | 0.21 |
| BC_tissue | Hoskinson_2022 | 0.21 |
| BC_tissue | Hoskinson_2022 | 0.16 |
| BC_tissue | Hoskinson_2022 | 0.3 |
| BC_tissue | Hoskinson_2022 | 0.09 |
| BC_adjacent | German_2023 | 0.83 |
| BC_adjacent | German_2023 | 0.76 |
| BC_adjacent | German_2023 | 0.91 |
| BC_adjacent | German_2023 | 0.85 |
| BC_adjacent | German_2023 | 0.86 |
| BC_adjacent | German_2023 | 0.95 |
| BC_adjacent | German_2023 | 0.89 |
| BC_adjacent | German_2023 | 0.79 |
| BC_adjacent | German_2023 | 0.83 |
| BC_adjacent | German_2023 | 0.89 |
| BC_adjacent | German_2023 | 0.92 |
| BC_adjacent | German_2023 | 0.93 |
| BC_adjacent | German_2023 | 0.87 |
| BC_adjacent | German_2023 | 0.86 |
| BC_adjacent | German_2023 | 0.89 |
| BC_adjacent | German_2023 | 0.84 |
| BC_adjacent | German_2023 | 0.95 |
| BC_adjacent | German_2023 | 0.93 |
| BC_adjacent | German_2023 | 0.84 |
| BC_adjacent | German_2023 | 0.88 |
| BC_adjacent | German_2023 | 0.82 |
| BC_adjacent | German_2023 | 0.86 |
| BC_adjacent | German_2023 | 0.94 |
| BC_adjacent | German_2023 | 0.94 |
| BC_adjacent | German_2023 | 0.81 |
| BC_adjacent | German_2023 | 0.89 |
| BC_adjacent | German_2023 | 0.87 |
| BC_adjacent | German_2023 | 0.95 |
| BC_adjacent | German_2023 | 0.93 |
| BC_adjacent | German_2023 | 0.81 |
| BC_adjacent | German_2023 | 0.91 |
| BC_adjacent | German_2023 | 0.84 |
| BC_adjacent | German_2023 | 0.82 |
| BC_adjacent | German_2023 | 0.86 |
| BC_tissue | Hoskinson_2022 | 0.14 |
| BC_tissue | Hoskinson_2022 | 0.13 |
| BC_tissue | Hoskinson_2022 | 0.11 |
| BC_tissue | Hoskinson_2022 | 0.22 |
| BC_tissue | Hoskinson_2022 | 0.18 |
| BC_tissue | Hoskinson_2022 | 0.06 |
| BC_tissue | German_2023 | 0.15 |
| BC_tissue | German_2023 | 0.19 |
| BC_tissue | German_2023 | 0.28 |
| BC_tissue | German_2023 | 0.15 |
| BC_tissue | German_2023 | 0.23 |
| BC_tissue | German_2023 | 0.17 |
| BC_tissue | German_2023 | 0.39 |
| BC_tissue | German_2023 | 0.4 |
| BC_tissue | German_2023 | 0.11 |
| BC_tissue | German_2023 | 0.1 |
| BC_tissue | German_2023 | 0.3 |
| BC_tissue | German_2023 | 0.13 |
| BC_tissue | German_2023 | 0.1 |
| BC_tissue | German_2023 | 0.19 |
| BC_tissue | German_2023 | 0.22 |
| BC_tissue | German_2023 | 0.24 |
| BC_tissue | German_2023 | 0.29 |
| BC_tissue | German_2023 | 0.28 |
| BC_tissue | German_2023 | 0.11 |
| BC_tissue | German_2023 | 0.33 |
| BC_tissue | German_2023 | 0.09 |
| BC_tissue | German_2023 | 0.3 |
| BC_tissue | German_2023 | 0.29 |
| BC_tissue | German_2023 | 0.22 |
| BC_tissue | German_2023 | 0.36 |
| BC_tissue | German_2023 | 0.28 |
| BC_tissue | German_2023 | 0.29 |
| BC_tissue | German_2023 | 0.19 |
| BC_tissue | German_2023 | 0.32 |
| BC_tissue | German_2023 | 0.15 |
| BC_adjacent | Hoskinson_2022 | 0.77 |
| BC_adjacent | Hoskinson_2022 | 0.82 |
| BC_adjacent | Hoskinson_2022 | 0.85 |
| BC_adjacent | Hoskinson_2022 | 0.7 |
| BC_adjacent | Hoskinson_2022 | 0.94 |
| BC_adjacent | Hoskinson_2022 | 0.83 |
| BC_adjacent | Hoskinson_2022 | 0.81 |
| BC_adjacent | Hoskinson_2022 | 0.79 |
| BC_adjacent | Hoskinson_2022 | 0.88 |
| BC_adjacent | Hoskinson_2022 | 0.78 |
| BC_adjacent | Hoskinson_2022 | 0.82 |
| BC_adjacent | Hoskinson_2022 | 0.78 |
| BC_adjacent | Hoskinson_2022 | 0.92 |
| BC_adjacent | Hoskinson_2022 | 0.77 |
| BC_adjacent | Hoskinson_2022 | 0.7 |
| BC_adjacent | Hoskinson_2022 | 0.9 |
| BC_adjacent | Hoskinson_2022 | 0.85 |
| BC_adjacent | Hoskinson_2022 | 0.96 |
| BC_adjacent | Hoskinson_2022 | 0.91 |
| BC_adjacent | Hoskinson_2022 | 0.68 |
| BC_adjacent | Hoskinson_2022 | 0.74 |
| BC_adjacent | Hoskinson_2022 | 0.84 |
| BC_adjacent | Hoskinson_2022 | 0.87 |
| BC_adjacent | Hoskinson_2022 | 0.74 |
| BC_adjacent | Hoskinson_2022 | 0.81 |
| BC_adjacent | Hoskinson_2022 | 0.75 |
| BC_adjacent | Hoskinson_2022 | 0.82 |
| BC_adjacent | Hoskinson_2022 | 0.85 |
| BC_adjacent | Hoskinson_2022 | 0.84 |
| BC_adjacent | Hoskinson_2022 | 0.87 |
| BC_adjacent | Hoskinson_2022 | 0.86 |
| BC_adjacent | Hoskinson_2022 | 0.75 |
| BC_adjacent | Hoskinson_2022 | 0.9 |
| BC_adjacent | Hoskinson_2022 | 0.75 |
| BC_adjacent | Hoskinson_2022 | 0.95 |
| BC_adjacent | Hoskinson_2022 | 0.75 |
| BC_adjacent | Hoskinson_2022 | 0.82 |
| BC_adjacent | Hoskinson_2022 | 0.87 |
| BC_adjacent | Hoskinson_2022 | 0.89 |
| BC_adjacent | Hoskinson_2022 | 0.86 |
| BC_adjacent | Hoskinson_2022 | 0.82 |
| BC_adjacent | Hoskinson_2022 | 0.88 |
| BC_adjacent | Hoskinson_2022 | 0.79 |
| BC_adjacent | Hoskinson_2022 | 0.94 |
| BC_adjacent | Hoskinson_2022 | 0.77 |
| BC_adjacent | Hoskinson_2022 | 0.85 |
| BC_adjacent | Hoskinson_2022 | 0.79 |
| BC_adjacent | Hoskinson_2022 | 0.94 |
| BC_adjacent | Hoskinson_2022 | 0.81 |
| BC_adjacent | German_2023 | 0.89 |
| BC_adjacent | German_2023 | 0.78 |
| BC_adjacent | German_2023 | 0.79 |
| BC_adjacent | German_2023 | 0.9 |
| BC_adjacent | German_2023 | 0.77 |
| BC_adjacent | German_2023 | 0.88 |
| BC_adjacent | German_2023 | 0.84 |
| BC_adjacent | German_2023 | 0.84 |
| BC_adjacent | German_2023 | 0.92 |
| BC_adjacent | German_2023 | 0.85 |
| BC_adjacent | German_2023 | 0.76 |
| BC_adjacent | German_2023 | 0.88 |
| BC_adjacent | German_2023 | 0.87 |
| BC_adjacent | German_2023 | 0.92 |
| BC_adjacent | German_2023 | 0.94 |
| BC_adjacent | German_2023 | 0.87 |
| BC_adjacent | German_2023 | 0.83 |
| BC_adjacent | German_2023 | 0.87 |
| BC_adjacent | German_2023 | 0.77 |
| BC_adjacent | German_2023 | 0.94 |
| BC_adjacent | German_2023 | 0.94 |
| BC_adjacent | German_2023 | 0.87 |
| BC_adjacent | German_2023 | 0.72 |
| BC_adjacent | German_2023 | 0.83 |
| BC_adjacent | German_2023 | 0.9 |
| BC_adjacent | German_2023 | 0.73 |
