# Supplementary material for: Microbial diagnostic features identified across populations possess potential antitumor properties in breast cancer
Source: mSystems. 2025 Jun 23;10(7):e00271-25. doi: 10.1128/msystems.00271-25 (PMC12282184; doi:10.1128/msystems.00271-25)
Supplement: Table S7 — The abundance of the 20 specific species in both BC_tissue and normal_tissue samples. [file msystems.00271-25-s0007.doc]

**Table S7A. The abundance of the 20 specific species in both BC_tissue and normal_tissue samples.**

| **Group** | **Cohort** | ***Cutibacterium_acnes*** | ***Ralstonia_pickettii_B*** | ***JC017_sp004296775*** | ***Lactobacillus_iners*** | ***Lacticaseibacillus_paracasei*** | ***Acetobacter_garciniae*** | ***Burkholderia_lata*** | ***Burkholderia_cepacia_576714*** | ***Burkholderia_mallei*** | ***Atopostipes_suicloacalis*** | ***Oceanobacillus_luteolus*** | ***Escherichia_coli*** | ***Finegoldia_magna_H*** | ***Herbaspirillum_huttiense*** | ***Enterococcus_H_360604_faecalis*** | ***Veillonella_A_rogosae*** | ***Akkermansia_muciniphila_D_776786*** | ***Ureaplasma_sp900544585*** | ***QHXM01_sp003222945*** | ***Arachnia_flavescens*** |
| --- | --- | --- | --- | --- | --- | --- | --- | --- | --- | --- | --- | --- | --- | --- | --- | --- | --- | --- | --- | --- | --- |
| BC_tissue | Hoskinson_2022 | 0.3511 | 0 | 0.0254 | 0 | 0 | 0 | 0 | 0 | 0 | 0.055 | 0 | 0.1734 | 0 | 0 | 0 | 0 | 0 | 0.0719 | 0 | 0 |
| BC_tissue | Hoskinson_2022 | 0.0017 | 0 | 0 | 0 | 0 | 0 | 0 | 0 | 0 | 0.0157 | 0 | 0.0035 | 0 | 0.0017 | 0 | 0 | 0 | 0.0017 | 0 | 0 |
| BC_tissue | Hoskinson_2022 | 0.4019 | 0 | 0 | 0 | 0 | 0 | 0 | 0 | 0 | 18.7692 | 0 | 2.6353 | 0 | 0 | 1.602 | 0 | 0 | 0 | 0 | 0 |
| BC_tissue | Hoskinson_2022 | 0.5535 | 0 | 0 | 0 | 0 | 0 | 0 | 0 | 0 | 0 | 0 | 1.7124 | 0 | 0 | 0 | 0.0027 | 0.0438 | 0 | 0 | 0 |
| BC_tissue | Hoskinson_2022 | 0.9261 | 0 | 0 | 0 | 0 | 0 | 0 | 0 | 0 | 0.0021 | 0 | 0 | 0 | 0 | 0 | 0 | 0 | 0.0086 | 0 | 0 |
| BC_tissue | Hoskinson_2022 | 1.1357 | 0 | 0 | 0 | 0 | 0 | 0 | 0 | 0 | 0.006 | 0 | 0 | 0 | 0 | 0 | 1.5002 | 0.0015 | 0 | 0 | 0 |
| BC_tissue | Hoskinson_2022 | 2.2896 | 0 | 0 | 7.4039 | 0 | 0 | 0 | 0 | 0 | 0 | 0 | 0 | 0 | 0 | 0 | 0 | 0 | 0 | 0 | 0 |
| BC_tissue | Hoskinson_2022 | 0.6074 | 0 | 0 | 0 | 0 | 0 | 0 | 0 | 0 | 0 | 0 | 0 | 0.0022 | 0 | 0 | 0 | 0.0022 | 0 | 0 | 0 |
| BC_tissue | Hoskinson_2022 | 0.3146 | 0 | 0 | 0 | 0.0019 | 0 | 0 | 0 | 0 | 0.6216 | 0 | 0 | 0 | 0.0038 | 2.4404 | 0.5583 | 0.0019 | 0 | 0 | 0 |
| BC_tissue | Hoskinson_2022 | 0.4978 | 0 | 0 | 0 | 0 | 0 | 0 | 0 | 0 | 0.0101 | 0 | 0.0025 | 0 | 0.0025 | 0 | 0 | 0.0101 | 0 | 0 | 0 |
| BC_tissue | Hoskinson_2022 | 3.1187 | 0 | 0 | 0 | 0.0015 | 0 | 0 | 0 | 0 | 0 | 0 | 0 | 0 | 0.0031 | 0 | 0 | 0.0015 | 0 | 0 | 0 |
| BC_tissue | Hoskinson_2022 | 4.0837 | 0 | 0 | 0 | 0 | 0 | 0 | 0 | 0 | 0 | 0.0068 | 0 | 0 | 30.3313 | 0 | 0 | 0 | 0 | 0 | 0 |
| BC_tissue | Hoskinson_2022 | 0.0017 | 0 | 0 | 0 | 0 | 0 | 0 | 0 | 0 | 0 | 59.7915 | 0 | 0.0017 | 0.0017 | 0.0348 | 0 | 0 | 0 | 0 | 0 |
| BC_tissue | Hoskinson_2022 | 0.0276 | 0 | 0 | 0 | 0 | 0 | 0 | 0 | 0 | 0.0013 | 0.0039 | 0 | 12.5322 | 0 | 1.6557 | 0 | 0 | 0 | 0 | 0 |
| BC_tissue | Hoskinson_2022 | 1.134 | 0 | 0 | 0 | 0 | 0 | 0 | 0 | 0 | 0 | 0.0048 | 0 | 0 | 0 | 0 | 0 | 0.0016 | 0 | 0 | 0 |
| BC_tissue | Hoskinson_2022 | 1.3141 | 0 | 0.0059 | 0 | 0 | 0 | 0 | 0 | 0 | 0 | 0 | 0.0039 | 0 | 0 | 0.002 | 0 | 0 | 0 | 0 | 0 |
| BC_tissue | Hoskinson_2022 | 0.0836 | 0 | 0 | 0 | 0 | 0 | 0 | 0 | 0 | 0.0777 | 0.01 | 0.1852 | 0.002 | 5.8142 | 0.0179 | 0 | 0.0279 | 0.1055 | 0 | 0 |
| BC_tissue | Hoskinson_2022 | 0.0078 | 0 | 0 | 0 | 0 | 0 | 0 | 0 | 0 | 0.0011 | 18.7384 | 0.0089 | 0 | 10.8199 | 0.0022 | 0 | 0.0045 | 0.0067 | 0 | 0 |
| BC_tissue | Hoskinson_2022 | 0.0675 | 0 | 0 | 0 | 0 | 0 | 0 | 0 | 0 | 0.0344 | 0.0025 | 0.037 | 0.0013 | 0 | 0.0025 | 0 | 0.0038 | 0.0229 | 0 | 0 |
| BC_tissue | Hoskinson_2022 | 0 | 0 | 0 | 0 | 0 | 0 | 0 | 0 | 0 | 0 | 0.0026 | 0 | 1.3195 | 0 | 0 | 0 | 0 | 0 | 0 | 0 |
| BC_tissue | Hoskinson_2022 | 0 | 0 | 0 | 0 | 0 | 0 | 0 | 0 | 0 | 0 | 0.0012 | 0 | 1.3381 | 0.0012 | 0 | 0 | 0 | 0 | 0 | 0 |
| BC_tissue | Hoskinson_2022 | 0.0553 | 0 | 0 | 0 | 0 | 0 | 0 | 0 | 0 | 0.018 | 0 | 0.0617 | 0 | 0 | 0.0039 | 0 | 0.0116 | 0.1196 | 0 | 0 |
| BC_tissue | Hoskinson_2022 | 0.0685 | 0 | 0 | 0 | 0 | 0 | 0 | 0 | 0 | 0.0055 | 0 | 0.0276 | 0 | 0 | 0.0022 | 0 | 0.0022 | 0.0188 | 0 | 0 |
| BC_tissue | Hoskinson_2022 | 0.1121 | 0 | 0 | 0 | 0 | 0 | 0 | 0 | 0 | 0.0127 | 0 | 0.0289 | 0 | 5.7465 | 0 | 0 | 12.1943 | 0.0277 | 0 | 0 |
| BC_tissue | Hoskinson_2022 | 0.1576 | 0 | 0 | 0 | 0 | 0 | 0 | 0 | 0 | 0.0812 | 0 | 0.1449 | 0.0096 | 0 | 0 | 0.0016 | 0.0032 | 0.1242 | 0 | 0 |
| BC_tissue | Hoskinson_2022 | 0.03 | 0 | 0 | 0 | 0 | 0 | 0 | 0 | 0 | 0.003 | 0.0045 | 0.012 | 0 | 0.0015 | 0 | 0 | 0.0075 | 0.009 | 0 | 0 |
| BC_tissue | Hoskinson_2022 | 0.1623 | 0 | 0.0012 | 23.015 | 0 | 0 | 0 | 0 | 0 | 0.02 | 0 | 0.0624 | 0 | 0 | 0 | 0 | 0.0087 | 0.0312 | 0 | 0 |
| BC_tissue | Hoskinson_2022 | 0.0749 | 0 | 0 | 0 | 0 | 0 | 0 | 0 | 0 | 0.0029 | 0 | 0.0214 | 0 | 0 | 0 | 0 | 0 | 0.0185 | 0 | 0 |
| BC_tissue | Hoskinson_2022 | 0.2343 | 0 | 0 | 0.0013 | 0 | 0 | 0 | 0 | 0 | 0.023 | 0 | 0.1703 | 0 | 0 | 0 | 0 | 0 | 0.0896 | 0 | 0 |
| BC_tissue | Hoskinson_2022 | 0.0066 | 0 | 0 | 0 | 0 | 0 | 0 | 0 | 0 | 0 | 0 | 0.0019 | 0 | 0 | 3.5643 | 0 | 0.0009 | 0.0009 | 0 | 0 |
| BC_tissue | Hoskinson_2022 | 0.1587 | 0 | 0 | 0.0036 | 0 | 0 | 0 | 0 | 0 | 0.1123 | 0 | 0.2228 | 0 | 0 | 0.016 | 0 | 0 | 0.1783 | 0 | 0 |
| BC_tissue | Hoskinson_2022 | 0.2317 | 0 | 0 | 0 | 0 | 0 | 0 | 0 | 0 | 0.0255 | 0 | 0.2201 | 0 | 0 | 0.0023 | 0 | 0 | 0.1344 | 0 | 0 |
| BC_tissue | Hoskinson_2022 | 0.0036 | 0 | 0.0018 | 0 | 0.009 | 0 | 0 | 0 | 0 | 0.0234 | 0 | 0.1635 | 0.0054 | 0 | 0.0234 | 0 | 0.0054 | 0.115 | 0 | 0 |
| BC_tissue | Hoskinson_2022 | 0.0054 | 0 | 0.0018 | 0 | 0 | 0 | 0 | 0 | 0 | 0.0036 | 0 | 0.0253 | 0 | 0.0018 | 0 | 0 | 0.0018 | 0.0072 | 0 | 0 |
| BC_tissue | Hoskinson_2022 | 0.2788 | 0 | 0 | 0 | 0 | 0 | 0 | 0 | 0 | 0.0236 | 0 | 0.0425 | 0 | 0 | 0.0047 | 11.4818 | 0.0118 | 0.0969 | 0 | 0 |
| BC_tissue | Hoskinson_2022 | 0.1035 | 0 | 16.8719 | 0 | 0 | 0 | 0 | 0 | 0 | 0 | 0 | 0.0232 | 0 | 8.8769 | 0 | 0 | 0 | 0.0161 | 0 | 0 |
| BC_tissue | Hoskinson_2022 | 0.0597 | 0 | 0.0021 | 0 | 0 | 0 | 0 | 0 | 0 | 0 | 0 | 0 | 0 | 0 | 0.0085 | 0 | 0.0021 | 0.0043 | 0 | 0 |
| BC_tissue | Hoskinson_2022 | 0.0071 | 0 | 0 | 0 | 1.9716 | 0 | 0 | 0 | 0 | 0 | 0 | 0.0107 | 0 | 0 | 12.2807 | 0 | 0 | 0.0053 | 0 | 0 |
| BC_tissue | Hoskinson_2022 | 0.1233 | 0 | 0.0022 | 0 | 0 | 0 | 0 | 0 | 0 | 0.0022 | 0 | 0.0727 | 0 | 0 | 0 | 0 | 0 | 0.0286 | 0 | 0 |
| BC_tissue | Hoskinson_2022 | 0 | 0 | 0 | 0 | 0 | 0 | 0 | 0 | 0 | 0 | 0 | 0.0031 | 0 | 0 | 0 | 0 | 0 | 0.0016 | 0 | 0 |
| BC_tissue | Hoskinson_2022 | 1.2373 | 0 | 0 | 0 | 0 | 0 | 0 | 0 | 0 | 0.0021 | 0 | 0.227 | 0 | 0 | 0 | 0.3357 | 0 | 0 | 0 | 0 |
| normal_tissue | Hoskinson_2022 | 0.1 | 0 | 4.4394 | 0 | 0 | 0 | 0 | 0 | 0 | 0.0216 | 0 | 0.0235 | 0 | 0 | 0 | 0 | 0 | 0.0137 | 0 | 0 |
| normal_tissue | Hoskinson_2022 | 1.9958 | 0 | 0 | 0 | 0 | 0 | 0 | 0 | 0 | 0 | 0 | 0.0071 | 0 | 0 | 0 | 0 | 0 | 0.01 | 0 | 0 |
| normal_tissue | Hoskinson_2022 | 0.0092 | 0 | 0 | 0 | 0 | 0 | 0 | 0 | 0 | 1.0299 | 0 | 1.735 | 0.0166 | 0 | 0.0018 | 0 | 0.0018 | 0.0185 | 0 | 0 |
| normal_tissue | Hoskinson_2022 | 2.4097 | 0 | 0 | 0 | 0 | 0 | 0 | 0 | 0 | 0.002 | 0 | 0.0039 | 0 | 0 | 0 | 0 | 0.0078 | 0 | 0 | 0 |
| normal_tissue | Hoskinson_2022 | 0 | 0 | 0.0145 | 0.0338 | 0 | 0 | 0 | 0 | 0 | 0 | 0 | 0 | 0 | 0 | 0 | 0 | 0 | 0 | 0 | 0 |
| normal_tissue | Hoskinson_2022 | 0.5008 | 0 | 0.002 | 0 | 0 | 0 | 0 | 0 | 0 | 0 | 0 | 0 | 0 | 0 | 0 | 0 | 0.0061 | 0 | 0 | 0 |
| normal_tissue | Hoskinson_2022 | 0.9027 | 0 | 0 | 0 | 0 | 0 | 0 | 0 | 0 | 0 | 0 | 0 | 0 | 0 | 0 | 0 | 0 | 0 | 0 | 0 |
| normal_tissue | Hoskinson_2022 | 1.5978 | 0 | 0 | 0 | 0 | 0 | 0 | 0 | 0 | 0 | 0 | 0 | 0 | 0 | 0 | 0 | 0.0074 | 0 | 0 | 0 |
| normal_tissue | Hoskinson_2022 | 0.0088 | 0 | 0 | 0 | 0 | 0 | 0 | 0 | 0 | 0 | 0 | 0 | 0 | 0 | 0 | 0 | 0 | 0 | 0 | 0 |
| normal_tissue | Hoskinson_2022 | 0.3535 | 0 | 0 | 0 | 0 | 0 | 0 | 0 | 0 | 0 | 0 | 0 | 0 | 0 | 0 | 0 | 0.002 | 0 | 0 | 0 |
| normal_tissue | Hoskinson_2022 | 1.8791 | 0 | 0 | 0 | 0 | 0 | 0 | 0 | 0 | 0 | 0 | 0 | 0 | 0 | 0.0076 | 0 | 0.0057 | 0 | 0 | 0 |
| normal_tissue | Hoskinson_2022 | 1.2534 | 0 | 0 | 0 | 0 | 0 | 0 | 0 | 0 | 0.0037 | 0 | 0 | 0 | 0 | 0 | 0 | 0.0037 | 0 | 0 | 0 |
| normal_tissue | Hoskinson_2022 | 1.0506 | 0 | 0 | 0 | 0 | 0 | 0 | 0 | 0 | 0 | 0 | 0.0108 | 0 | 0 | 0 | 0 | 0.0043 | 0.0022 | 0 | 0 |
| normal_tissue | Hoskinson_2022 | 0.1521 | 0 | 0 | 0 | 0 | 0 | 0 | 0 | 0 | 0.0042 | 0 | 0 | 0 | 0 | 0 | 0 | 0.0084 | 0 | 0 | 0 |
| normal_tissue | Hoskinson_2022 | 0.4593 | 0 | 0.0069 | 0 | 0 | 0 | 0 | 0 | 0 | 0 | 0 | 0 | 0 | 0 | 0 | 0 | 0.0014 | 0 | 0 | 0 |
| normal_tissue | Hoskinson_2022 | 0.7457 | 0 | 6.1692 | 0 | 0 | 0 | 0 | 0 | 0 | 0.0268 | 0 | 0 | 0 | 0 | 0 | 0 | 0 | 0 | 0 | 0 |
| normal_tissue | Hoskinson_2022 | 0.0308 | 0 | 0.0014 | 0 | 0 | 0 | 0 | 0 | 0 | 0 | 0 | 0 | 0 | 0 | 0 | 0.0014 | 0 | 0 | 0 | 0 |
| normal_tissue | Hoskinson_2022 | 0.1001 | 0 | 0 | 0 | 0 | 0 | 0 | 0 | 0 | 0 | 0 | 0 | 0 | 0 | 0 | 0 | 0.0015 | 0 | 0 | 0 |
| normal_tissue | Hoskinson_2022 | 0.0942 | 0 | 0 | 0 | 0 | 0 | 0 | 0 | 0 | 0 | 0 | 0 | 0 | 0 | 0 | 0 | 0 | 0 | 0 | 0 |
| normal_tissue | Hoskinson_2022 | 0.0541 | 0 | 0 | 0 | 0 | 0 | 0 | 0 | 0 | 0 | 0 | 0 | 0 | 0 | 0 | 0 | 0 | 0 | 0 | 0 |
| normal_tissue | Hoskinson_2022 | 1.2032 | 0 | 4.0165 | 0 | 0 | 0 | 0 | 0 | 0 | 0.0016 | 0 | 0 | 0 | 0 | 0 | 0 | 0.0016 | 0 | 0 | 0 |
| normal_tissue | Hoskinson_2022 | 0.274 | 0 | 0 | 0 | 0 | 0 | 0 | 0 | 0 | 0 | 0 | 0 | 0 | 0 | 0 | 0 | 0.0031 | 0 | 0 | 0 |
| normal_tissue | Hoskinson_2022 | 0.4893 | 0 | 0 | 0 | 0 | 0 | 0 | 0 | 0 | 0 | 0 | 0 | 0 | 0 | 0 | 0 | 0 | 0 | 0 | 0 |
| normal_tissue | Hoskinson_2022 | 0.1554 | 0 | 0 | 0 | 0 | 0 | 0 | 0 | 0 | 0 | 0 | 0 | 0 | 0 | 0 | 0.004 | 0 | 0 | 0 | 0 |
| normal_tissue | Hoskinson_2022 | 0.8166 | 0 | 0.4161 | 0 | 0 | 0 | 0 | 0 | 0 | 0.7657 | 0 | 0 | 6.2484 | 0 | 0.0013 | 0 | 0 | 0 | 0 | 0 |
| normal_tissue | Hoskinson_2022 | 1.6994 | 0 | 0 | 0 | 0 | 0 | 0 | 0 | 0 | 0 | 0 | 0 | 0 | 0 | 0.0018 | 0 | 0 | 0 | 0 | 0 |
| normal_tissue | Hoskinson_2022 | 0.8235 | 0 | 0 | 0 | 0 | 0 | 0 | 0 | 0 | 0 | 0.0016 | 0 | 0 | 0 | 0 | 0 | 0.0016 | 0 | 0 | 0 |
| normal_tissue | Hoskinson_2022 | 0.2347 | 0 | 0 | 0 | 0 | 0 | 0 | 0 | 0 | 0 | 0 | 0 | 0 | 0 | 0 | 0 | 0 | 0 | 0 | 0 |
| normal_tissue | Hoskinson_2022 | 0.8384 | 0 | 0.0018 | 0 | 0 | 0 | 0 | 0 | 0 | 0.0018 | 0 | 0 | 0 | 0.0018 | 0 | 0 | 0.0071 | 0 | 0 | 0 |
| normal_tissue | Hoskinson_2022 | 0.2418 | 0 | 2.8475 | 0 | 0 | 0 | 0 | 0 | 0 | 0.0014 | 0.0014 | 0 | 0 | 0 | 0 | 0 | 0.0056 | 0 | 0 | 0 |
| normal_tissue | Hoskinson_2022 | 0.0883 | 0 | 0 | 0 | 0 | 0 | 0 | 0 | 0 | 0 | 0 | 0 | 0 | 0 | 0 | 0 | 0 | 0 | 0 | 0 |
| normal_tissue | Hoskinson_2022 | 0 | 0 | 0 | 0 | 0 | 0 | 0 | 0 | 0 | 0 | 0 | 0 | 0 | 0 | 0.0056 | 0 | 0 | 0 | 0 | 0 |
| normal_tissue | Hoskinson_2022 | 0.2365 | 0 | 0.7402 | 0 | 0 | 0 | 0 | 0 | 0 | 0 | 0 | 0 | 0 | 0 | 0 | 0 | 0 | 0 | 0 | 0 |
| normal_tissue | Hoskinson_2022 | 0.4002 | 0 | 0 | 0 | 0 | 0 | 0 | 0 | 0 | 0 | 0 | 0 | 0 | 0 | 0 | 2.5087 | 0 | 0 | 0 | 0 |
| normal_tissue | Hoskinson_2022 | 0.7039 | 0 | 0 | 0 | 0 | 0 | 0 | 0 | 0 | 0 | 0 | 0 | 0 | 0 | 0.0021 | 0 | 0 | 0 | 0 | 0 |
| normal_tissue | Hoskinson_2022 | 0.0374 | 0 | 0 | 0 | 0 | 0 | 0 | 0 | 0 | 0 | 0.0019 | 0 | 0 | 0 | 0 | 0 | 0 | 0 | 0 | 0 |
| normal_tissue | Hoskinson_2022 | 0.1786 | 0 | 0 | 0 | 0 | 0 | 0 | 0 | 0 | 0 | 0 | 0 | 0 | 0 | 0 | 0 | 0 | 0 | 0 | 0 |
| normal_tissue | Hoskinson_2022 | 0.1565 | 0 | 0 | 0 | 0 | 0 | 0 | 0 | 0 | 0 | 0 | 0 | 0 | 0 | 0.0017 | 0 | 0 | 0 | 0 | 0 |
| normal_tissue | Hoskinson_2022 | 0.4667 | 0 | 0 | 0 | 0 | 0 | 0 | 0 | 0 | 0 | 0 | 0 | 0 | 0 | 0 | 0 | 0 | 0 | 0 | 0 |
| normal_tissue | Hoskinson_2022 | 0.1569 | 0 | 0 | 0 | 0 | 0 | 0 | 0 | 0 | 0 | 0 | 0 | 0 | 0 | 0 | 0 | 0 | 0 | 0 | 0 |
| normal_tissue | Hoskinson_2022 | 0.0962 | 0 | 0 | 0 | 0 | 0 | 0 | 0 | 0 | 0 | 0 | 0 | 0 | 0 | 0 | 0 | 0 | 0 | 0 | 0 |
| normal_tissue | Hoskinson_2022 | 0.6512 | 0 | 0 | 0 | 0 | 0 | 0 | 0 | 0 | 0 | 0 | 0 | 0 | 0 | 0 | 0 | 0 | 0 | 0 | 0 |
| normal_tissue | Hoskinson_2022 | 0.0893 | 0 | 0 | 0 | 0 | 0 | 0 | 0 | 0 | 0 | 0 | 0.0026 | 0 | 0 | 0 | 0 | 0 | 0 | 0 | 0 |
| normal_tissue | Hoskinson_2022 | 0.2399 | 0 | 0 | 0 | 0 | 0 | 0 | 0 | 0 | 0 | 0 | 0 | 0 | 0 | 0 | 0 | 0 | 0 | 0 | 0 |
| normal_tissue | Hoskinson_2022 | 0 | 0 | 0 | 0 | 0 | 0 | 0 | 0 | 0 | 0 | 0 | 0 | 0 | 0 | 0 | 0 | 0 | 0 | 0 | 0 |
| normal_tissue | Hoskinson_2022 | 0 | 0 | 0 | 0 | 0 | 0 | 0 | 0 | 0 | 0 | 0 | 0 | 0 | 0 | 0 | 0 | 0 | 0 | 0 | 0 |
| normal_tissue | Hoskinson_2022 | 0.451 | 0 | 0 | 0 | 0 | 0 | 0 | 0 | 0 | 0 | 0 | 0 | 0 | 0 | 0 | 0 | 0 | 0 | 0 | 0 |
| normal_tissue | Hoskinson_2022 | 0.8977 | 0 | 0 | 0 | 0 | 0 | 0 | 0 | 0 | 0 | 0 | 0.0016 | 0 | 0 | 0 | 0 | 0 | 0 | 0 | 0 |
| normal_tissue | Hoskinson_2022 | 0.8623 | 0 | 0 | 0 | 0 | 0 | 0 | 0 | 0 | 0 | 0 | 0 | 0 | 0 | 0 | 0 | 0 | 0 | 0 | 0 |
| normal_tissue | German_2023 | 0.1803 | 0.0671 | 0.0416 | 0 | 4.0503 | 5.148 | 0.1865 | 0.0549 | 0.0932 | 0.0466 | 0 | 0 | 0.0466 | 0 | 0 | 0 | 0 | 0 | 0 | 0 |
| normal_tissue | German_2023 | 0.5116 | 0 | 0.8323 | 0 | 5.7976 | 5.2081 | 0.1584 | 0.3142 | 0.148 | 0 | 0 | 0 | 0 | 0 | 0 | 0 | 0 | 0 | 0 | 0 |
| normal_tissue | German_2023 | 0.0769 | 0.0377 | 0.0422 | 0 | 5.9957 | 7.7529 | 0.1472 | 0.4031 | 0.0703 | 0 | 0 | 0 | 0 | 0 | 0 | 0 | 0 | 0 | 0 | 0 |
| normal_tissue | German_2023 | 0.055 | 0.0346 | 0.002 | 0.0632 | 0 | 0 | 0.2527 | 0.1793 | 0.2079 | 0 | 0 | 0 | 0 | 0 | 0 | 0 | 0 | 0 | 0 | 0 |
| normal_tissue | German_2023 | 0.2708 | 1.2503 | 0.004 | 0 | 0 | 0 | 1.6326 | 1.0432 | 0.8442 | 0 | 0 | 0 | 0.004 | 0 | 0 | 0 | 0 | 0 | 0 | 0 |
| normal_tissue | German_2023 | 0.1291 | 0.1088 | 0.0096 | 0 | 4.6981 | 3.7961 | 0.1859 | 0.039 | 0.1734 | 0 | 0 | 0 | 0 | 0 | 0 | 0 | 0 | 0 | 0 | 0 |
| normal_tissue | German_2023 | 0.2417 | 0 | 0.1234 | 0 | 4.9971 | 0.4388 | 0.3986 | 0.1444 | 0.2031 | 0 | 0 | 0 | 0 | 0 | 0 | 0 | 0 | 0 | 0 | 0 |
| normal_tissue | German_2023 | 0.1398 | 0 | 0.0008 | 0 | 4.6644 | 4.0221 | 0.2643 | 0.141 | 0.2751 | 0 | 0 | 0 | 0 | 0 | 0 | 0 | 0 | 0 | 0 | 0 |
| normal_tissue | German_2023 | 0.0007 | 0.0003 | 0.0158 | 0 | 2.9736 | 3.0593 | 0.1074 | 0.0833 | 0.1098 | 0.0003 | 0 | 0 | 0 | 0 | 0 | 0 | 0 | 0 | 0 | 0 |
| normal_tissue | German_2023 | 0.1072 | 0 | 0.0208 | 0 | 3.1694 | 3.7429 | 0.2958 | 0.053 | 0.0831 | 0 | 0 | 0 | 0 | 0 | 0 | 0 | 0 | 0 | 0 | 0 |
| normal_tissue | German_2023 | 0.259 | 0.0687 | 0.0124 | 0.0023 | 0.0045 | 0.1452 | 0.2781 | 0.4245 | 0.1396 | 0.0011 | 0 | 0 | 0.0023 | 0 | 0 | 0 | 0 | 0 | 0 | 0 |
| normal_tissue | German_2023 | 0.0587 | 0 | 0 | 0 | 4.436 | 4.504 | 0.107 | 0.2026 | 0.1274 | 0 | 0 | 0 | 0 | 0 | 0.0005 | 0 | 0 | 0 | 0 | 0 |
| normal_tissue | German_2023 | 0.0433 | 0.0336 | 0.0607 | 0 | 0.0092 | 0.0765 | 0.0627 | 0.0153 | 0.0265 | 0.0005 | 0 | 0 | 0.0015 | 0 | 0 | 0 | 0 | 0 | 0 | 0 |
| normal_tissue | German_2023 | 1.2673 | 0.2828 | 0.3221 | 0.0052 | 0 | 0.2985 | 0.1623 | 0.5106 | 0.3823 | 0.0655 | 0 | 0 | 0 | 0 | 0 | 0 | 0 | 0 | 0 | 0 |
| normal_tissue | German_2023 | 0.021 | 0.0606 | 0.0037 | 0.0346 | 0.1695 | 0.1485 | 0.1695 | 0.1844 | 0.1868 | 0 | 0 | 0.0012 | 0 | 0 | 0 | 0 | 0 | 0 | 0 | 0 |
| normal_tissue | German_2023 | 0.147 | 0.0673 | 0.0049 | 0 | 3.6887 | 4.0673 | 0.2697 | 0.1415 | 0.1673 | 0 | 0 | 0 | 0 | 0 | 0 | 0 | 0 | 0 | 0 | 0 |
| normal_tissue | German_2023 | 0.1398 | 0 | 0 | 0 | 4.9527 | 2.7208 | 0.1127 | 0.2806 | 0.0895 | 0 | 0 | 0 | 0 | 0 | 0 | 0 | 0 | 0 | 0 | 0 |
| normal_tissue | German_2023 | 0.6094 | 0.1518 | 0.2476 | 0 | 5.869 | 2.6639 | 0.7548 | 0.5661 | 0.3739 | 0 | 0 | 0 | 0 | 0 | 0 | 0 | 0 | 0 | 0 | 0 |
| normal_tissue | German_2023 | 0.1868 | 0.2097 | 0 | 0 | 0 | 0.213 | 1.2779 | 0.8618 | 0.8421 | 0 | 0 | 0 | 0.0066 | 0 | 0 | 0 | 0 | 0 | 0 | 0 |
| normal_tissue | German_2023 | 0.1998 | 0.0666 | 0 | 0 | 2.1786 | 0.6949 | 0.9185 | 0.152 | 0.2612 | 0 | 0 | 0 | 0 | 0 | 0 | 0 | 0 | 0 | 0 | 0 |
| normal_tissue | German_2023 | 0.0994 | 0 | 0.0155 | 0 | 3.6303 | 2.3628 | 0.255 | 0.1248 | 0.2164 | 0 | 0 | 0 | 0 | 0 | 0 | 0 | 0 | 0 | 0 | 0 |
| normal_tissue | German_2023 | 0.0018 | 0 | 0.0107 | 0 | 2.4984 | 2.9866 | 0.0572 | 0.0304 | 0.0894 | 0 | 0 | 0 | 0 | 0 | 0 | 0 | 0 | 0 | 0 | 0 |
| normal_tissue | German_2023 | 0.141 | 0.047 | 0.0078 | 0.0026 | 0.0679 | 0.0392 | 0.0731 | 0.47 | 0.3917 | 0 | 0 | 0 | 0 | 0 | 0 | 0 | 0 | 0 | 0 | 0 |
| normal_tissue | German_2023 | 0.0516 | 0.0854 | 0 | 0.0159 | 0 | 0 | 0.4727 | 0.0894 | 0.0993 | 0 | 0 | 0 | 0 | 0 | 0 | 0.0457 | 0 | 0 | 0 | 0 |
| normal_tissue | German_2023 | 0.1013 | 0.022 | 0.0468 | 0 | 4.2932 | 3.4 | 0.1767 | 0.0525 | 0.1634 | 0 | 0 | 0 | 0 | 0 | 0 | 0.106 | 0 | 0 | 0 | 0 |
| normal_tissue | German_2023 | 0.0452 | 0.0745 | 0.0358 | 0 | 3.2046 | 3.601 | 0.0948 | 0.0749 | 0.1872 | 0.0273 | 0 | 0 | 0 | 0 | 0.0004 | 0 | 0 | 0 | 0 | 0 |
| normal_tissue | German_2023 | 1.4844 | 15.7524 | 0.0826 | 0 | 0 | 0.1592 | 0.365 | 2.3638 | 0.4822 | 0 | 0 | 0 | 0 | 0 | 0 | 0.0004 | 0 | 0 | 0 | 0 |
| normal_tissue | German_2023 | 0.2259 | 0.0228 | 0.0203 | 0 | 4.6163 | 4.282 | 0.0071 | 0.2092 | 0.0957 | 0 | 0 | 0 | 0 | 0 | 0 | 0 | 0 | 0 | 0 | 0 |
| normal_tissue | German_2023 | 0.1896 | 0.0126 | 0 | 0 | 6.3651 | 0.4784 | 0 | 0.1005 | 1.302 | 0.2737 | 0 | 0 | 0 | 0 | 0 | 0 | 0 | 0 | 0 | 0 |
| normal_tissue | German_2023 | 0.2418 | 0 | 0.0199 | 0 | 3.8097 | 3.8924 | 0.1884 | 0.1413 | 0.1183 | 0 | 0 | 0 | 0 | 0 | 0 | 0 | 0 | 0 | 0 | 0 |
| normal_tissue | German_2023 | 0.0347 | 0.0113 | 0.0105 | 0 | 1.9283 | 2.017 | 0.0749 | 0.1072 | 0.0258 | 0 | 0 | 0 | 0 | 0 | 0 | 0 | 0 | 0 | 0 | 0 |
| normal_tissue | German_2023 | 0.0497 | 0 | 0.2268 | 0 | 4.5281 | 3.9921 | 0.3686 | 0.3965 | 0.4996 | 0 | 0 | 0 | 0 | 0 | 0 | 0 | 0 | 0 | 0 | 0 |
| normal_tissue | German_2023 | 0.3178 | 0 | 0.0247 | 0 | 0.1919 | 0.4593 | 0.4029 | 0.3278 | 0.1567 | 0 | 0 | 0 | 0 | 0 | 0 | 0 | 0 | 0 | 0 | 0 |
| normal_tissue | German_2023 | 2.8212 | 0.0332 | 0.1025 | 0 | 0.0443 | 0.1588 | 0.0305 | 0.3176 | 0.0231 | 0.1616 | 0 | 0 | 0 | 0 | 0 | 0 | 0 | 0 | 0 | 0 |
| normal_tissue | German_2023 | 0.1068 | 0 | 0.028 | 0 | 0.059 | 0.0149 | 0.1291 | 0.108 | 0.1053 | 0 | 0 | 0 | 0 | 0 | 0 | 0 | 0 | 0 | 0 | 0 |
| normal_tissue | German_2023 | 3.1982 | 0.6066 | 0.3406 | 0 | 0.1809 | 0.1756 | 0 | 0 | 0 | 0 | 0 | 0 | 0 | 0.1064 | 0 | 0.0053 | 0 | 0 | 0 | 0 |
| normal_tissue | German_2023 | 5.3624 | 0.0013 | 0.1086 | 0 | 0.0265 | 0.0119 | 0.2649 | 0.5695 | 0.1934 | 0 | 0 | 0 | 0 | 0 | 0 | 0 | 0 | 0 | 0 | 0 |
| normal_tissue | German_2023 | 0.0071 | 0.0219 | 0 | 0 | 0.0046 | 0.0168 | 0.0549 | 0.1068 | 0.0188 | 0 | 0 | 0 | 0 | 0 | 0 | 0 | 0 | 0 | 0 | 0 |
| normal_tissue | German_2023 | 3.8446 | 0.011 | 0.0268 | 0.0154 | 0.0187 | 0.0162 | 0.0426 | 0.1584 | 0.0727 | 0 | 0 | 0 | 0 | 0 | 0 | 0.0037 | 0 | 0 | 0 | 0 |
| normal_tissue | German_2023 | 0.462 | 0.5372 | 0.0322 | 0 | 0.0322 | 0 | 0 | 0 | 0 | 0 | 0 | 0 | 0 | 0 | 0 | 0 | 0 | 0 | 0 | 0 |
| normal_tissue | German_2023 | 1.1683 | 0.6243 | 0 | 0 | 0 | 0.3261 | 0.0034 | 1.0287 | 0.1968 | 0 | 0 | 0 | 0 | 0 | 0 | 0 | 0 | 0 | 0 | 0 |
| normal_tissue | German_2023 | 0.0143 | 0.0092 | 0.0197 | 0 | 2.5462 | 3.3049 | 0.0697 | 0.022 | 0.0416 | 0.0031 | 0 | 0 | 0 | 0 | 0 | 0 | 0 | 0 | 0 | 0 |
| normal_tissue | German_2023 | 0.0968 | 0.0286 | 0.0223 | 0 | 5.223 | 7.9752 | 0.0236 | 0.0195 | 0.0291 | 0.0182 | 0 | 0 | 0 | 0 | 0 | 0 | 0 | 0 | 0 | 0 |
| normal_tissue | German_2023 | 0.1193 | 0 | 0.0133 | 0 | 0.0098 | 0.0086 | 0.0734 | 0.0549 | 0.0541 | 0 | 0 | 0.0098 | 0 | 0 | 0 | 0 | 0 | 0 | 0 | 0 |
| normal_tissue | German_2023 | 0.1561 | 0.0289 | 0.1041 | 0 | 0.0058 | 0.1099 | 0.0578 | 0.1388 | 0.0867 | 0 | 0 | 0 | 0 | 0 | 0 | 0 | 0 | 0 | 0 | 0 |
| normal_tissue | German_2023 | 0.105 | 0.052 | 0 | 0 | 0.0081 | 0.0256 | 0.0004 | 0 | 0 | 0 | 0 | 0 | 0.0004 | 0 | 0 | 0 | 0 | 0 | 0 | 0 |
| normal_tissue | German_2023 | 1.0276 | 0.0212 | 0.0212 | 0 | 0.0848 | 1.3985 | 0 | 0.1271 | 0 | 0 | 0 | 0 | 0.2755 | 0 | 0 | 0 | 0 | 0 | 0 | 0 |
| normal_tissue | German_2023 | 0.4321 | 0.1143 | 0.0019 | 0 | 0.0547 | 0.2266 | 0.1133 | 0.145 | 0.4801 | 0 | 0 | 0 | 0 | 0 | 0 | 0 | 0 | 0 | 0 | 0 |
| normal_tissue | German_2023 | 11.3365 | 0.0442 | 0.1093 | 0 | 0.0068 | 0.0094 | 0 | 0.0004 | 0.0043 | 0 | 0 | 0 | 0.0145 | 0 | 0 | 0.0179 | 0 | 0 | 0 | 0 |
| normal_tissue | German_2023 | 0.0059 | 0.0684 | 0.0089 | 0 | 0.342 | 0.1636 | 0.1993 | 0.1011 | 0.1398 | 0 | 0 | 0 | 0.0387 | 0 | 0 | 0 | 0 | 0 | 0 | 0 |
| normal_tissue | German_2023 | 0.0348 | 0.0144 | 0.0287 | 0 | 5.0436 | 6.4312 | 0.0597 | 0.1503 | 0.1293 | 0 | 0 | 0 | 0 | 0 | 0.0011 | 0 | 0 | 0 | 0 | 0 |
| normal_tissue | German_2023 | 0.0006 | 0.4374 | 0.0006 | 0 | 0.047 | 0 | 0.2489 | 1.9761 | 0.3062 | 0 | 0 | 0 | 0 | 0 | 0 | 0 | 0 | 0 | 0 | 0 |
| normal_tissue | German_2023 | 0.1591 | 0.0378 | 0.1318 | 0 | 0 | 0.0006 | 0.2537 | 0.4966 | 0.2415 | 0 | 0 | 0 | 0 | 0 | 0 | 0 | 0 | 0 | 0 | 0 |
| normal_tissue | German_2023 | 0.0143 | 0.0261 | 0.0005 | 0 | 0 | 0.0488 | 0.2338 | 0.0996 | 0.04 | 0 | 0 | 0 | 0 | 0 | 0 | 0 | 0 | 0 | 0 | 0 |
| normal_tissue | German_2023 | 0.611 | 0 | 0.0009 | 0 | 0.1476 | 0 | 0.611 | 0.3203 | 0.1337 | 0 | 0 | 0 | 0 | 0 | 0 | 0 | 0 | 0 | 0 | 0 |
| normal_tissue | German_2023 | 0.1641 | 0.082 | 0.0373 | 0 | 0.1044 | 0.4251 | 0 | 0 | 0 | 0 | 0 | 0.0149 | 0 | 0 | 0 | 0.0075 | 0 | 0 | 0 | 0 |
| normal_tissue | German_2023 | 0.1289 | 0.0015 | 0.0091 | 0 | 0 | 0 | 0.6789 | 0.531 | 1.2455 | 0 | 0 | 0 | 0 | 0 | 0 | 0 | 0 | 0 | 0 | 0 |
| normal_tissue | German_2023 | 1.0335 | 0.0748 | 0 | 0 | 0.2771 | 0.2727 | 0.9411 | 0.2419 | 0.3914 | 0 | 0 | 0 | 0 | 0 | 0 | 0 | 0 | 0 | 0 | 0 |
| normal_tissue | German_2023 | 1.1389 | 0.2735 | 0.1467 | 0 | 0.1317 | 0.4636 | 1.1123 | 1.3107 | 0.4002 | 0 | 0 | 0 | 0 | 0 | 0 | 0 | 0 | 0 | 0 | 0 |
| normal_tissue | German_2023 | 0.068 | 0.0182 | 0.0601 | 0.0049 | 0.0607 | 0.0638 | 0.0298 | 0.0911 | 0.0723 | 0 | 0 | 0 | 0 | 0 | 0 | 0 | 0 | 0 | 0 | 0 |
| normal_tissue | German_2023 | 0.112 | 0.01 | 0.0465 | 0 | 0.2857 | 0.1537 | 0.1746 | 0.4849 | 0.0449 | 0 | 0 | 0 | 0 | 0 | 0 | 0 | 0 | 0 | 0 | 0 |
| normal_tissue | German_2023 | 0.6309 | 0.1475 | 0.0367 | 0 | 0.0006 | 0 | 2.189 | 0.7772 | 0.4156 | 0 | 0 | 0 | 0.1954 | 0 | 0 | 0 | 0 | 0 | 0 | 0 |
| normal_tissue | German_2023 | 0.5416 | 0.2619 | 1.3996 | 0 | 0.0005 | 0 | 0.9905 | 2.3713 | 2.3231 | 0 | 0 | 0 | 0 | 0 | 0 | 0 | 0 | 0 | 0 | 0 |
| normal_tissue | German_2023 | 0.6973 | 0.0003 | 0.0005 | 0.0013 | 0.0003 | 0.0622 | 0.592 | 0.9562 | 0.5685 | 0 | 0 | 0 | 0 | 0 | 0 | 0 | 0 | 0 | 0 | 0 |
| normal_tissue | German_2023 | 4.2199 | 0.8831 | 0.0158 | 0 | 0.7179 | 1.4285 | 0 | 0.1628 | 0.9876 | 0 | 0 | 0 | 1.6411 | 0 | 0 | 0.0024 | 0.0036 | 0 | 0 | 0 |
| normal_tissue | German_2023 | 0.4468 | 0.0087 | 0.0473 | 0 | 0.0466 | 0.107 | 0.3238 | 0.2969 | 0.1601 | 0 | 0 | 0 | 0 | 0.0538 | 0 | 0 | 0 | 0 | 0 | 0 |
| normal_tissue | German_2023 | 1.2555 | 0.2667 | 0.0003 | 0 | 0 | 0.0563 | 1.6788 | 1.8312 | 1.6146 | 0 | 0 | 0 | 0 | 0 | 0.004 | 0 | 0 | 0 | 0 | 0 |
| normal_tissue | German_2023 | 1.4705 | 0.0764 | 0.0157 | 0 | 0.0999 | 0.2115 | 1.9493 | 2.9636 | 0.5453 | 0 | 0 | 0 | 0 | 0 | 0.0029 | 0 | 0 | 0 | 0 | 0 |
| normal_tissue | German_2023 | 0 | 0.1144 | 0.0686 | 0 | 0.0578 | 0.2774 | 0.5886 | 0.2597 | 0.6303 | 0 | 0 | 0 | 0.0006 | 0 | 0 | 0 | 0 | 0 | 0 | 0 |
| normal_tissue | German_2023 | 15.8163 | 0 | 2.4943 | 0 | 0 | 0.0685 | 2.7391 | 2.8986 | 2.3018 | 0 | 0 | 0 | 0 | 0 | 0 | 0 | 0 | 0 | 0 | 0 |
| normal_tissue | German_2023 | 7.3781 | 0.0055 | 0.5924 | 0.1035 | 0.151 | 0.1717 | 0 | 0.0003 | 0.0003 | 0 | 0 | 0 | 0.6904 | 0 | 0.1079 | 0.0003 | 0 | 0 | 0 | 0 |
| normal_tissue | German_2023 | 9.7721 | 0.173 | 0.052 | 0 | 0 | 0.0032 | 0.0512 | 1.4718 | 0.9487 | 0 | 0 | 0 | 0 | 0 | 0 | 0 | 0 | 0 | 0 | 0 |
| normal_tissue | German_2023 | 1.4192 | 0.1883 | 0.2846 | 0 | 0.0749 | 0.1977 | 0.4245 | 1.2534 | 0.4379 | 0 | 0 | 0 | 0 | 0 | 0 | 0 | 0 | 0 | 0 | 0 |
| normal_tissue | German_2023 | 2.601 | 0.8802 | 0.1747 | 0.0824 | 0.8703 | 1.1703 | 0 | 0.1615 | 0 | 0 | 0 | 0 | 0.3626 | 0 | 0 | 0 | 0 | 0 | 0 | 0 |
| normal_tissue | German_2023 | 0.62 | 0 | 0.0705 | 0 | 0 | 0 | 1.5922 | 0.465 | 0.775 | 0 | 0 | 0 | 0.0141 | 0 | 0 | 0 | 0 | 0 | 0 | 0 |
| normal_tissue | German_2023 | 1.0456 | 0.1541 | 0 | 0 | 0 | 0.1288 | 0.6592 | 1.6669 | 0.7425 | 0 | 0 | 0 | 0.0682 | 0 | 0 | 0 | 0 | 0 | 0 | 0 |
| normal_tissue | German_2023 | 0.6147 | 0.02 | 0.1051 | 0 | 0 | 0.1341 | 0.5057 | 0.8517 | 0.0883 | 0 | 0 | 0 | 0 | 0 | 0 | 0 | 0 | 0 | 0 | 0 |
| normal_tissue | German_2023 | 0.8315 | 0.0684 | 0 | 0 | 0 | 0.2889 | 1.6163 | 1.2855 | 0.6992 | 0 | 0 | 0 | 0 | 0 | 0.0003 | 0 | 0 | 0 | 0 | 0 |
| normal_tissue | German_2023 | 0.4884 | 0.0921 | 0.0667 | 0 | 0 | 0.0689 | 0.2587 | 0.2942 | 0.2176 | 0 | 0 | 0 | 0 | 0 | 0 | 0 | 0 | 0 | 0 | 0 |
| normal_tissue | German_2023 | 0.6073 | 0.1735 | 0.0005 | 0 | 0.0589 | 1.1697 | 1.7197 | 1.0778 | 0.7121 | 0 | 0 | 0 | 1.1836 | 0 | 0 | 0 | 0 | 0 | 0 | 0 |
| normal_tissue | German_2023 | 0.2936 | 0.0958 | 0.1188 | 0 | 0.0112 | 0.0306 | 0.2773 | 0.7951 | 0.0454 | 0.0133 | 0 | 0 | 0.0005 | 0 | 0 | 0 | 0 | 0 | 0 | 0 |
| normal_tissue | German_2023 | 1.2678 | 0.2448 | 0.0016 | 0 | 0 | 0.0137 | 1.4844 | 3.7432 | 1.6747 | 0 | 0 | 0 | 0 | 0 | 0 | 0 | 0 | 0 | 0 | 0 |
| normal_tissue | German_2023 | 3.6455 | 0.1782 | 0.4413 | 0 | 0 | 0.3517 | 0.8143 | 0.5756 | 1.16 | 0 | 0 | 0 | 0 | 0 | 0 | 0 | 0 | 0 | 0 | 0 |
| normal_tissue | German_2023 | 5.1111 | 0.0004 | 0.1229 | 0 | 0.1175 | 0.0961 | 0.5389 | 1.5276 | 0.3772 | 0 | 0 | 0 | 0 | 0 | 0 | 0 | 0 | 0 | 0 | 0 |
| normal_tissue | German_2023 | 13.2878 | 0.0807 | 8.7641 | 0.0904 | 0 | 0.0291 | 0.2083 | 0.147 | 0.6003 | 0 | 0 | 0 | 0.5457 | 0 | 0 | 0 | 0 | 0 | 0 | 0 |
| normal_tissue | German_2023 | 0.6253 | 0.1863 | 0.0067 | 0 | 0.133 | 0 | 0 | 0 | 0 | 0 | 0 | 0 | 0 | 0 | 0 | 0 | 0 | 0 | 0 | 0 |
| normal_tissue | German_2023 | 3.4425 | 0.5026 | 0.1557 | 0 | 0.214 | 0.2647 | 0.3988 | 0.7066 | 0.7426 | 0 | 0 | 0 | 0 | 0 | 0 | 0 | 0 | 0 | 0 | 0 |
| normal_tissue | German_2023 | 0.8027 | 0 | 0.1604 | 0 | 0.0005 | 0.1987 | 0.1252 | 0.9984 | 0.4158 | 0 | 0 | 0 | 0 | 0 | 0 | 0.0089 | 0 | 0 | 0 | 0 |
| normal_tissue | German_2023 | 3.877 | 0.5976 | 0.0073 | 0 | 0.1239 | 0.1603 | 0 | 0.2696 | 0 | 0 | 0 | 0 | 0.532 | 0 | 0 | 0.8235 | 0 | 0 | 0 | 0 |
| normal_tissue | German_2023 | 1.3152 | 0.13 | 0.8988 | 0 | 0.0004 | 0.2307 | 0.6054 | 0.9987 | 0.1494 | 0 | 0 | 0 | 0 | 0 | 0 | 0 | 0 | 0 | 0 | 0 |
| normal_tissue | German_2023 | 4.1714 | 0.3239 | 0.0109 | 0.0005 | 4.1133 | 11.2046 | 0 | 0.001 | 0.001 | 0 | 0 | 0 | 0.004 | 0 | 0.0005 | 0 | 0 | 0 | 0 | 0 |
| normal_tissue | German_2023 | 1.0168 | 0.0028 | 0.0176 | 0.0009 | 0.0297 | 0.0028 | 0 | 0 | 0 | 0 | 0 | 0 | 0.0718 | 0 | 0.0003 | 0 | 0 | 0 | 0 | 0 |
| normal_tissue | German_2023 | 0.0983 | 0.0061 | 0.0164 | 0 | 0 | 0.0541 | 0.0735 | 0.2256 | 0.0667 | 0 | 0 | 0 | 0 | 0 | 0 | 0 | 0 | 0 | 0 | 0 |
| normal_tissue | German_2023 | 1.2231 | 0.0004 | 0.0004 | 0 | 0 | 0.0717 | 0.0794 | 0.1541 | 0.3061 | 0 | 0 | 0 | 0 | 0 | 0 | 0 | 0 | 0 | 0 | 0 |
| normal_tissue | German_2023 | 3.13 | 0.2696 | 5.3607 | 0.0369 | 0.156 | 0.6325 | 0 | 0.0646 | 0 | 0 | 0 | 0 | 1.2566 | 0 | 0 | 0.2308 | 0.4903 | 0 | 0 | 0 |
| normal_tissue | German_2023 | 0.1571 | 0.3936 | 0.0035 | 0.0005 | 0.1168 | 1.1885 | 0 | 0 | 0 | 0 | 0 | 0 | 0.0035 | 0 | 0 | 0 | 0.0005 | 0 | 0 | 0 |
| normal_tissue | German_2023 | 2.0712 | 3.0353 | 1.8467 | 0 | 0 | 0.1804 | 0.4375 | 0.4461 | 0.1852 | 0 | 0 | 0 | 0.5459 | 0 | 0 | 0 | 0 | 0 | 0 | 0 |
| normal_tissue | German_2023 | 0.1582 | 2.6384 | 0 | 0 | 0 | 0.0046 | 0.1377 | 0.1833 | 0.1628 | 0 | 0 | 0 | 0 | 0 | 0 | 0 | 0 | 0 | 0 | 0 |
| normal_tissue | German_2023 | 2.7889 | 2.0859 | 0.9515 | 1.6989 | 0 | 0.0887 | 0.2702 | 0.1965 | 0.3982 | 0 | 0 | 0 | 0.2512 | 0 | 0 | 0 | 0 | 0 | 0 | 0 |
| normal_tissue | German_2023 | 0.0046 | 0.0369 | 0.0783 | 0 | 0 | 0 | 0.6727 | 0.2949 | 0.2811 | 0 | 0 | 0 | 0 | 0 | 0 | 0 | 0 | 0 | 0 | 0 |
| normal_tissue | German_2023 | 1.7798 | 0 | 0.0066 | 0 | 0 | 0.0066 | 3.0681 | 0.4383 | 0.8899 | 0 | 0 | 0 | 0 | 0 | 0 | 0 | 0 | 0 | 0 | 0 |
| normal_tissue | German_2023 | 0.8833 | 0.3087 | 0.3197 | 0 | 0 | 0.5857 | 1.3919 | 0.6835 | 1.2899 | 0 | 0 | 0 | 0 | 0 | 0 | 0 | 0 | 0 | 0 | 0 |
| normal_tissue | German_2023 | 0.0466 | 0.1373 | 0.0263 | 0 | 0.2054 | 1.0784 | 0.0836 | 0.1009 | 0.1451 | 0 | 0 | 0 | 0 | 0 | 0 | 0 | 0 | 0 | 0 | 0 |
| normal_tissue | German_2023 | 3.0347 | 13.7563 | 1.1077 | 0.0004 | 0 | 0 | 1.4412 | 1.2826 | 1.0367 | 0 | 0 | 0 | 0.1225 | 0 | 0 | 0.0004 | 0 | 0 | 0 | 0 |
| normal_tissue | German_2023 | 0.6284 | 4.7714 | 0 | 0 | 0 | 0.3828 | 0.4379 | 0.5089 | 0.7377 | 0 | 0 | 0 | 0 | 0 | 0 | 0 | 0 | 0 | 0 | 0 |
| normal_tissue | German_2023 | 1.6965 | 0.3099 | 0.509 | 0.5903 | 0.6529 | 1.5856 | 0 | 0.6291 | 0.6466 | 0 | 0 | 0 | 0.202 | 0 | 0 | 0.0006 | 0 | 0 | 0 | 0 |
| normal_tissue | German_2023 | 1.2082 | 0.584 | 0.003 | 0 | 0 | 0.1905 | 2.0164 | 1.2866 | 1.8775 | 0.4287 | 0 | 0 | 0.001 | 0 | 0 | 0 | 0 | 0 | 0 | 0 |
| normal_tissue | German_2023 | 0.5145 | 0.3001 | 0.001 | 0 | 0.0047 | 0.053 | 0 | 0.001 | 0.0415 | 0.001 | 0 | 0 | 0.0005 | 0 | 0 | 0 | 0 | 0 | 0 | 0 |
| normal_tissue | German_2023 | 3.3416 | 0.0047 | 0.7858 | 0 | 0 | 0 | 1.5858 | 0.8661 | 0.5853 | 0 | 0 | 0 | 0 | 0 | 0 | 0 | 0 | 0 | 0 | 0 |
| normal_tissue | German_2023 | 1.4173 | 0 | 1.7932 | 0 | 0 | 0 | 0.6039 | 1.442 | 0.7025 | 0 | 0 | 0 | 0 | 0 | 0 | 0 | 0 | 0 | 0 | 0 |
| normal_tissue | German_2023 | 0.4753 | 0.3148 | 0.2468 | 0 | 0.0105 | 0.5451 | 0.5704 | 2.0294 | 0.1962 | 0 | 0 | 0 | 0 | 0 | 0 | 0 | 0 | 0 | 0 | 0 |
| normal_tissue | German_2023 | 0.1187 | 0.206 | 0.0019 | 0 | 0 | 0.2072 | 0.9394 | 0.7026 | 0.5061 | 0 | 0 | 0 | 0 | 0 | 0 | 0 | 0 | 0 | 0 | 0 |
| normal_tissue | German_2023 | 1.6032 | 0 | 0.0068 | 0 | 0 | 1.5355 | 1.84 | 2.7058 | 1.0079 | 0 | 0 | 0 | 0 | 0 | 0 | 0 | 0 | 0 | 0 | 0 |
| normal_tissue | German_2023 | 0.941 | 0 | 0.0123 | 0 | 0 | 0.0813 | 0.282 | 0.9323 | 0.2488 | 0 | 0 | 0 | 0 | 0 | 0 | 0 | 0 | 0 | 0 | 0 |
| normal_tissue | German_2023 | 1.7991 | 1.6826 | 0.8017 | 0 | 0.5174 | 1.2025 | 4.2414 | 1.7945 | 7.7511 | 0 | 0 | 0 | 0 | 0 | 0 | 0 | 0 | 0 | 0 | 0 |
| normal_tissue | German_2023 | 0.0236 | 0.6463 | 0 | 0 | 0 | 0.0009 | 0.7386 | 0.7207 | 0.5182 | 0 | 0 | 0 | 0 | 0 | 0 | 0 | 0 | 0 | 0 | 0 |
| normal_tissue | German_2023 | 2.4423 | 0.4604 | 0 | 0 | 0 | 0.3614 | 2.0758 | 1.1356 | 0.9967 | 0 | 0 | 0 | 0 | 0 | 0 | 0 | 0 | 0 | 0 | 0 |
| normal_tissue | German_2023 | 5.167 | 2.8225 | 0.0062 | 0 | 0.0033 | 0.0986 | 0.067 | 0 | 0 | 0 | 0 | 0 | 0.3383 | 0 | 0 | 0.0005 | 0.0005 | 0 | 0 | 0 |
| BC_tissue | Hoskinson_2022 | 1.9872 | 0 | 0 | 0 | 0 | 0 | 0 | 0 | 0 | 0 | 0 | 0.0134 | 0 | 0 | 0.0015 | 0 | 0 | 0.0224 | 0 | 0 |
| BC_tissue | Hoskinson_2022 | 0.0113 | 0 | 0 | 0 | 0 | 0 | 0 | 0 | 0 | 0.0038 | 0 | 0.0151 | 0 | 0 | 2.1681 | 0 | 0 | 0.0151 | 0 | 0 |
| BC_tissue | Hoskinson_2022 | 0.7355 | 0 | 0 | 0 | 0 | 0 | 0 | 0 | 0 | 0.0014 | 0 | 0.0087 | 0 | 0 | 0 | 0 | 0.0087 | 0.0072 | 0 | 0 |
| BC_tissue | Hoskinson_2022 | 0.0249 | 0 | 0 | 0 | 0 | 0 | 0 | 0 | 0 | 0 | 0 | 0.0748 | 0 | 0 | 0 | 0 | 0.0111 | 0.0831 | 0 | 0 |
| BC_tissue | Hoskinson_2022 | 0.2844 | 0 | 0 | 0 | 0 | 0 | 0 | 0 | 0 | 0.0086 | 0 | 0.0129 | 0 | 0 | 0 | 0 | 0 | 0.0129 | 0 | 0 |
| normal_tissue | German_2023 | 1.7835 | 1.3408 | 0.0026 | 0 | 0.9298 | 0.6708 | 0 | 0 | 0 | 0 | 0 | 0 | 0.0009 | 0 | 0 | 0 | 0.0009 | 0 | 0 | 0 |
| normal_tissue | German_2023 | 0.0988 | 0.0709 | 0.0056 | 0.0112 | 0 | 0.2575 | 0 | 0 | 0 | 0 | 0 | 0 | 0.1793 | 0 | 0 | 0 | 0 | 0 | 0 | 0 |
| normal_tissue | German_2023 | 0.2117 | 0 | 0.2835 | 0 | 3.8389 | 6.6724 | 1.0444 | 0.264 | 0.6855 | 0 | 0 | 0 | 0 | 0 | 0.0009 | 0 | 0 | 0 | 0 | 0 |
| normal_tissue | German_2023 | 3.129 | 0.0838 | 2.3153 | 0 | 0 | 0.2549 | 0.1705 | 0.8224 | 0.3434 | 0.096 | 0 | 0.0128 | 0 | 0 | 0 | 0.0006 | 0 | 0 | 0 | 0 |
| normal_tissue | German_2023 | 0.1651 | 0.0577 | 0.0781 | 0 | 4.3016 | 1.2336 | 0.3076 | 0.0998 | 0.0871 | 0 | 0 | 0 | 0 | 0 | 0.0005 | 0 | 0 | 0 | 0 | 0 |
| normal_tissue | German_2023 | 0.2261 | 0.1125 | 0.0011 | 0 | 5.2838 | 1.33 | 0.8348 | 1.1017 | 1.6367 | 0 | 0 | 0 | 0 | 0 | 0.0011 | 0 | 0 | 0 | 0 | 0 |
| normal_tissue | German_2023 | 0.0577 | 0 | 0.0004 | 0 | 2.0571 | 2.3039 | 0.1843 | 0.2735 | 0.0315 | 0 | 0 | 0 | 0 | 0 | 0 | 0 | 0 | 0 | 0 | 0 |
| normal_tissue | German_2023 | 0.4504 | 0.0253 | 0.0752 | 0 | 6.3403 | 7.3622 | 0.1789 | 0.1488 | 0.2865 | 0 | 0 | 0 | 0 | 0 | 0 | 0 | 0 | 0 | 0 | 0 |
| normal_tissue | German_2023 | 0.2161 | 0.0373 | 0.0008 | 0 | 0.0025 | 0.0852 | 0.2207 | 0.0533 | 0.1838 | 0 | 0 | 0 | 0.0827 | 0 | 0 | 0 | 0 | 0 | 0 | 0 |
| normal_tissue | German_2023 | 0.4258 | 0 | 0 | 0 | 0.0142 | 0.0047 | 0.0649 | 0.066 | 0.102 | 0 | 0 | 0 | 0 | 0 | 0 | 0 | 0 | 0 | 0 | 0 |
| normal_tissue | German_2023 | 0.0006 | 0.029 | 0.0157 | 0 | 6.3927 | 7.4326 | 0.1817 | 0.025 | 0.0993 | 0 | 0 | 0 | 0 | 0 | 0 | 0 | 0 | 0 | 0 | 0 |
| normal_tissue | German_2023 | 0.1091 | 0.0308 | 0.0196 | 0 | 0 | 0.0028 | 0.1454 | 0.0056 | 0.0923 | 0 | 0 | 0 | 0 | 0 | 0 | 0 | 0 | 0 | 0 | 0 |
| normal_tissue | German_2023 | 6.9818 | 0 | 2.2039 | 0 | 1.8338 | 0.0673 | 3.718 | 5.4004 | 8.8829 | 0 | 0 | 0 | 0 | 0 | 0 | 0 | 0 | 0 | 0 | 0 |
| normal_tissue | German_2023 | 0.3577 | 0 | 0.8464 | 0 | 0.0045 | 0.0058 | 2.4249 | 2.9833 | 1.9652 | 0 | 0 | 0 | 0 | 0 | 0 | 0 | 0 | 0 | 0 | 0 |
| normal_tissue | German_2023 | 0.7449 | 0.096 | 0.0056 | 0 | 0.3539 | 0.0009 | 0.8027 | 0.0021 | 0.2031 | 0 | 0 | 0 | 0 | 0 | 0 | 0 | 0 | 0 | 0 | 0 |
| normal_tissue | German_2023 | 0.5076 | 0.0677 | 1.4857 | 0 | 0.0305 | 0.264 | 0.5855 | 0.0474 | 0.555 | 0 | 0 | 0 | 0 | 0 | 0 | 0 | 0 | 0 | 0 | 0 |
| normal_tissue | German_2023 | 0.0688 | 0.0235 | 0.1494 | 0 | 3.4945 | 0.8093 | 0.3422 | 0.1912 | 0.2585 | 0 | 0 | 0 | 0 | 0.281 | 0 | 0 | 0 | 0 | 0 | 0 |
| normal_tissue | German_2023 | 0.0361 | 0 | 0 | 0 | 0.0004 | 0.0065 | 0.0962 | 0.2473 | 0.2386 | 0 | 0 | 0 | 0 | 0 | 0 | 0 | 0 | 0 | 0 | 0 |
| normal_tissue | German_2023 | 0.1167 | 0.1371 | 0 | 0 | 0.178 | 0.0782 | 0.3236 | 0.4595 | 0.4018 | 0 | 0 | 0 | 0 | 0 | 0 | 0 | 0 | 0 | 0 | 0 |
| normal_tissue | German_2023 | 0.1127 | 0.0038 | 0.2329 | 0 | 0.1728 | 0.0075 | 0.7363 | 0.0376 | 0.0826 | 0 | 0 | 0 | 0 | 0 | 0 | 0.9316 | 0 | 0 | 0 | 0 |
| normal_tissue | German_2023 | 0.0231 | 0.0121 | 0.0119 | 0 | 0.0204 | 0.0117 | 0.0335 | 0.0339 | 0.0273 | 0 | 0 | 0 | 0 | 0 | 0 | 0 | 0 | 0 | 0 | 0 |
| normal_tissue | German_2023 | 0.0037 | 0.0372 | 0.0037 | 0 | 0 | 0.0372 | 0.0894 | 0.108 | 0.175 | 0 | 0 | 0 | 0 | 0 | 0 | 0 | 0 | 0 | 0 | 0 |
| normal_tissue | German_2023 | 0.6417 | 0.0619 | 0.0888 | 0 | 0 | 0.0336 | 0.1197 | 0.0309 | 0.0108 | 0 | 0 | 0 | 0 | 0 | 0 | 0 | 0 | 0 | 0 | 0 |
| normal_tissue | German_2023 | 0.0359 | 0.117 | 0.0015 | 0 | 0.0275 | 0.0314 | 0 | 0 | 0.0268 | 0 | 0 | 0.0421 | 0 | 0 | 0 | 0 | 0 | 0 | 0 | 0 |
| normal_tissue | German_2023 | 0.0024 | 0.0287 | 0 | 0 | 0 | 0.0718 | 0.1328 | 0.0993 | 0.2559 | 0 | 0 | 0 | 0 | 0 | 0 | 0 | 0 | 0 | 0 | 0 |
| normal_tissue | German_2023 | 0.3826 | 0.1995 | 0.0589 | 0 | 0.0294 | 0.1766 | 0.0491 | 0.1537 | 0 | 0 | 0 | 0.0556 | 0 | 0 | 0 | 0.0458 | 0 | 0 | 0 | 0 |
| normal_tissue | German_2023 | 0.0075 | 0.0175 | 0 | 0 | 0.008 | 0.0031 | 0.026 | 0.0242 | 0.0057 | 0 | 0 | 0 | 0 | 0 | 0 | 0.0003 | 0 | 0 | 0 | 0 |
| normal_tissue | German_2023 | 3.1076 | 0.1238 | 0.4894 | 0.0523 | 0.0634 | 0.0326 | 0.0566 | 0.1598 | 0.1453 | 0.1071 | 0 | 0.0197 | 0 | 0 | 0 | 0.1234 | 0 | 0 | 0 | 0 |
| normal_tissue | German_2023 | 0.0033 | 0.0083 | 0.0532 | 0 | 0.0017 | 0.0216 | 0.1063 | 0.0465 | 0.1063 | 0 | 0 | 0 | 0.0781 | 0 | 0 | 0 | 0 | 0 | 0 | 0 |
| normal_tissue | German_2023 | 0.6077 | 0.2024 | 0 | 0 | 0.0003 | 0 | 0.6645 | 0.1373 | 0.3958 | 0 | 0 | 0 | 0 | 0 | 0 | 0 | 0.0003 | 0 | 0 | 0 |
| normal_tissue | German_2023 | 0.2948 | 0.0273 | 0.1307 | 0 | 0.0385 | 0.0077 | 0.0188 | 0.1342 | 0.1222 | 0 | 0 | 0 | 0.0145 | 0 | 0 | 0 | 0 | 0 | 0 | 0 |
| normal_tissue | German_2023 | 0.1152 | 0.0194 | 0 | 0 | 0.021 | 0 | 0.1185 | 0.1217 | 0.11 | 0 | 0 | 0 | 0 | 0 | 0 | 0 | 0 | 0 | 0 | 0 |
| normal_tissue | German_2023 | 0.1539 | 0.0036 | 0 | 0 | 0 | 0.0066 | 0.1361 | 0.2977 | 0.1488 | 0 | 0 | 0 | 0 | 0 | 0 | 0 | 0 | 0 | 0 | 0 |
| normal_tissue | German_2023 | 1.8861 | 0.109 | 0.3465 | 0 | 0.0762 | 0.3635 | 0.0822 | 0.0004 | 0.1332 | 0.2443 | 0 | 0 | 0 | 0 | 0 | 0.166 | 0 | 0 | 0 | 0 |
| normal_tissue | German_2023 | 0.1793 | 0 | 0.159 | 0 | 0 | 0 | 0.6261 | 0.3599 | 0.041 | 0 | 0 | 0 | 0 | 0 | 0 | 0 | 0 | 0 | 0 | 0 |
| normal_tissue | German_2023 | 0.2125 | 0.1026 | 0.2881 | 0 | 0.1458 | 0.1566 | 0.5294 | 0.1868 | 0.1958 | 0 | 0 | 0 | 0 | 0 | 0 | 0 | 0 | 0 | 0 | 0 |
| normal_tissue | German_2023 | 1.4127 | 0.3518 | 0.2236 | 0.2267 | 0 | 0 | 0.6362 | 0.8773 | 0.342 | 0 | 0 | 0 | 0 | 0 | 0 | 0 | 0 | 0 | 0 | 0 |
| normal_tissue | German_2023 | 0.039 | 0.1755 | 0.0364 | 0 | 3.9423 | 0.7108 | 0.3818 | 0.3866 | 0.5366 | 0 | 0 | 0 | 0 | 0 | 0.0004 | 0 | 0 | 0 | 0 | 0 |
| normal_tissue | German_2023 | 0.3659 | 0.6688 | 0.1388 | 0 | 0.3155 | 0.0126 | 0 | 0 | 0 | 0 | 0 | 0 | 0 | 0 | 0 | 0 | 0 | 0 | 0 | 0 |
| normal_tissue | German_2023 | 0.7936 | 0.2707 | 0.0373 | 0 | 0.2614 | 0.3828 | 0 | 0 | 0 | 0 | 0 | 0.0093 | 0 | 0 | 0 | 0 | 0 | 0 | 0 | 0 |
| normal_tissue | German_2023 | 4.4362 | 0.0005 | 0.7009 | 0 | 0 | 0 | 0.6899 | 0.2434 | 1.4897 | 0 | 0 | 0 | 0 | 0 | 0 | 0 | 0 | 0 | 0 | 0 |
| normal_tissue | German_2023 | 3.9553 | 0.0826 | 0.0462 | 0 | 0.0151 | 0.19 | 0 | 0.1758 | 0.0551 | 0 | 0 | 0 | 0.2584 | 0 | 0 | 0 | 0 | 0 | 0 | 0 |
| normal_tissue | German_2023 | 0.8009 | 0.1825 | 0 | 0.0138 | 0.2949 | 0.1716 | 0.1046 | 0.1608 | 0.3778 | 0 | 0 | 0 | 0 | 0 | 0 | 0.0434 | 0 | 0 | 0 | 0 |
| normal_tissue | German_2023 | 0.668 | 0 | 0 | 0 | 0.5194 | 0.0818 | 0.6763 | 0.986 | 0.379 | 0 | 0 | 0 | 0 | 0 | 0 | 0 | 0 | 0 | 0 | 0 |
| normal_tissue | German_2023 | 1.4599 | 0.0969 | 0.1439 | 0 | 0 | 0.1348 | 0.4013 | 0.2544 | 0.2529 | 0 | 0 | 0 | 0 | 0 | 0 | 0 | 0 | 0 | 0 | 0 |
| normal_tissue | German_2023 | 1.0706 | 0.0052 | 0.0249 | 0 | 0.1308 | 0.1765 | 0.1163 | 0.4766 | 0.3967 | 0.0831 | 0 | 0 | 0.0239 | 0 | 0 | 0 | 0 | 0 | 0 | 0 |
| normal_tissue | German_2023 | 0.0067 | 0.0452 | 0.0004 | 0 | 0.031 | 0.0672 | 0.1784 | 0.2789 | 0.2911 | 0 | 0 | 0 | 0 | 0 | 0 | 0 | 0 | 0 | 0 | 0 |
| normal_tissue | German_2023 | 0.2504 | 0.2902 | 0.0005 | 0 | 0 | 0.0234 | 0 | 0 | 0 | 0 | 0 | 0 | 0 | 0 | 0 | 0.0014 | 0 | 0 | 0 | 0 |
| normal_tissue | German_2023 | 0.3168 | 0.0138 | 0.1507 | 0 | 0 | 0.2031 | 0.0695 | 0 | 0 | 0 | 0 | 0.0006 | 0 | 0 | 0 | 0 | 0 | 0 | 0 | 0 |
| normal_tissue | German_2023 | 2.6751 | 0 | 0.1422 | 0 | 0.1117 | 0.4978 | 1.1344 | 0.0677 | 0.6975 | 0 | 0 | 0 | 0 | 0 | 0 | 0 | 0 | 0 | 0 | 0 |
| normal_tissue | German_2023 | 0.1196 | 0.1977 | 0 | 0 | 0.5539 | 0.2115 | 0.1775 | 0.7491 | 0.8939 | 0 | 0 | 0 | 0 | 0 | 0 | 0 | 0 | 0 | 0 | 0 |
| normal_tissue | German_2023 | 0.0016 | 0.0381 | 0.0016 | 0 | 0.0597 | 0 | 0.0013 | 0.0784 | 0.1516 | 0 | 0 | 0 | 0 | 0 | 0 | 0 | 0 | 0 | 0 | 0 |
| normal_tissue | German_2023 | 0.0307 | 0 | 0.0384 | 0 | 4.9175 | 5.1987 | 0.0323 | 0.1352 | 0.0522 | 0 | 0 | 0 | 0 | 0 | 0 | 0 | 0 | 0 | 0 | 0 |
| normal_tissue | German_2023 | 0.055 | 0.0464 | 0.0004 | 0 | 0.0021 | 0.0794 | 0 | 0 | 0 | 0 | 0 | 0.0009 | 0 | 0 | 0 | 0 | 0 | 0 | 0 | 0 |
| normal_tissue | German_2023 | 0.8166 | 0.1182 | 0 | 0 | 0.5121 | 1.1807 | 0 | 0 | 0 | 0.006 | 0 | 0 | 0.2268 | 0 | 0 | 0 | 0.0012 | 0 | 0 | 0 |
| normal_tissue | German_2023 | 0.2576 | 0.018 | 0 | 0 | 0.0238 | 0.0472 | 0.0563 | 0.02 | 0.0978 | 0 | 0 | 0 | 0 | 0 | 0 | 0 | 0 | 0 | 0 | 0 |
| normal_tissue | German_2023 | 0.5712 | 0.0156 | 3.3486 | 0 | 0.0278 | 0.0467 | 0.0589 | 0.0922 | 0.13 | 0 | 0 | 0 | 0 | 0 | 0 | 0.0167 | 0 | 0 | 0 | 0 |
| normal_tissue | German_2023 | 0.1381 | 0.0004 | 0 | 0 | 0.0381 | 0.1595 | 0.091 | 0.2646 | 0.1976 | 0 | 0 | 0 | 0 | 0 | 0 | 0 | 0 | 0 | 0 | 0 |
| normal_tissue | German_2023 | 0.1481 | 0.2339 | 0.1442 | 0 | 0.1793 | 0.2222 | 0.8499 | 0.5029 | 0.308 | 0 | 0 | 0 | 0 | 0 | 0 | 0 | 0 | 0 | 0 | 0 |
| normal_tissue | German_2023 | 0.0975 | 0.1684 | 0 | 0 | 0.0321 | 0.0011 | 0 | 0 | 0 | 0 | 0 | 0 | 0 | 0 | 0 | 0 | 0 | 0 | 0 | 0 |
| normal_tissue | German_2023 | 0.2664 | 0.0444 | 0.1776 | 0 | 0 | 0.0444 | 1.9091 | 1.1988 | 0.888 | 0 | 0 | 0 | 0 | 0 | 0 | 0 | 0 | 0 | 0 | 0 |
| normal_tissue | German_2023 | 0.1898 | 0.0275 | 0.0343 | 0.0006 | 0.0399 | 0.1174 | 0.182 | 0.2836 | 0.2415 | 0 | 0 | 0 | 0 | 0 | 0 | 0 | 0 | 0 | 0 | 0 |
| normal_tissue | German_2023 | 0.9685 | 0.0006 | 0.0013 | 0 | 0.302 | 0.0013 | 0.8267 | 0.2718 | 1.2927 | 0 | 0 | 0 | 0 | 0 | 0 | 0 | 0 | 0 | 0 | 0 |
| normal_tissue | German_2023 | 0.1435 | 0.1224 | 0.0813 | 0 | 0.0893 | 0 | 0.3 | 0.1575 | 0.5529 | 0 | 0 | 0 | 0 | 0 | 0 | 0 | 0 | 0 | 0 | 0 |
| normal_tissue | German_2023 | 1.7506 | 0.0022 | 0.0011 | 0 | 0 | 0 | 0.3291 | 0.2007 | 0.1716 | 0 | 0 | 0 | 0 | 0 | 0 | 0 | 0 | 0 | 0 | 0 |
| normal_tissue | German_2023 | 1.1439 | 0.0387 | 0.005 | 1.4234 | 0.113 | 0.1953 | 0.3152 | 0.1586 | 0.2022 | 0 | 0 | 0 | 0 | 0 | 0 | 0 | 0 | 0 | 0 | 0 |
| normal_tissue | German_2023 | 0.7592 | 0.2066 | 8.573 | 0.367 | 0 | 0 | 1.7704 | 2.7739 | 0.8639 | 0 | 0 | 0 | 0 | 0 | 0 | 0 | 0 | 0 | 0 | 0 |
| normal_tissue | German_2023 | 1.7592 | 0.0941 | 0.0941 | 0 | 0 | 0.1612 | 1.2061 | 0.2462 | 0.5866 | 0 | 0 | 0 | 0 | 0 | 0 | 0 | 0 | 0 | 0 | 0 |
| normal_tissue | German_2023 | 2.159 | 1.3116 | 0.0009 | 0 | 0 | 0 | 0 | 0 | 0.0009 | 0 | 0 | 0 | 0 | 0 | 0 | 0 | 0 | 0 | 0 | 0 |
| normal_tissue | German_2023 | 4.8446 | 0.0876 | 0.0923 | 0.1693 | 0.0024 | 0.1853 | 0.0574 | 0.1515 | 0.1592 | 0.0474 | 0 | 0 | 0 | 0 | 0 | 0 | 0 | 0 | 0 | 0 |
| normal_tissue | German_2023 | 0.2488 | 0.9661 | 0.1899 | 0 | 0 | 0.0451 | 0.6333 | 0.7689 | 1.4264 | 0 | 0 | 0 | 0 | 0 | 0 | 0 | 0 | 0 | 0 | 0 |
| normal_tissue | German_2023 | 0.5219 | 0.1484 | 0 | 0 | 0.0307 | 0.4298 | 0.4363 | 0.4222 | 0.4663 | 0 | 0 | 0 | 0 | 0 | 0 | 0 | 0 | 0 | 0 | 0 |
| normal_tissue | German_2023 | 2.4996 | 0.8704 | 0.901 | 0 | 0 | 0.0053 | 3.3595 | 2.1202 | 1.1297 | 0 | 0 | 0 | 0 | 0 | 0 | 0 | 0 | 0 | 0 | 0 |
| normal_tissue | German_2023 | 27.5357 | 0 | 0.3395 | 0 | 0.2368 | 0 | 0 | 0 | 0.2557 | 0 | 0 | 0 | 0.0012 | 0 | 0 | 0 | 0 | 0 | 0 | 0 |
| normal_tissue | German_2023 | 0.0254 | 0.0004 | 0.3266 | 0.5831 | 0.1234 | 0.0004 | 1.7642 | 0.6027 | 0.9358 | 0 | 0 | 0 | 0 | 0 | 0 | 0 | 0 | 0 | 0 | 0 |
| normal_tissue | German_2023 | 0.0755 | 0.0375 | 0 | 0 | 0.028 | 0.2001 | 0.0745 | 0.1335 | 0.2446 | 0 | 0 | 0 | 0 | 0 | 0 | 0 | 0 | 0 | 0 | 0 |
| normal_tissue | German_2023 | 1.0575 | 0.065 | 0.0351 | 0 | 0.014 | 0.0517 | 0.1321 | 0.2607 | 0.2379 | 0 | 0 | 0 | 0.015 | 0 | 0 | 0.3056 | 0 | 0 | 0 | 0 |
| normal_tissue | German_2023 | 0.3169 | 0.0191 | 0 | 0.0282 | 0.0451 | 0.0143 | 0.211 | 0.2166 | 0.1346 | 0 | 0 | 0 | 0 | 0 | 0 | 0 | 0 | 0 | 0 | 0 |
| normal_tissue | German_2023 | 3.3176 | 0.7258 | 0 | 0 | 0 | 0.551 | 5.2016 | 5.5894 | 2.8777 | 0 | 0 | 0 | 0 | 0 | 0 | 0 | 0 | 0 | 0 | 0 |
| normal_tissue | German_2023 | 0.0619 | 0 | 0.0492 | 0 | 0.0686 | 0.096 | 0.4373 | 0.1522 | 0.1487 | 0 | 0 | 0 | 0 | 0 | 0 | 0 | 0 | 0 | 0 | 0 |
| normal_tissue | German_2023 | 3.052 | 0.0007 | 42.6942 | 0.002 | 0 | 0 | 0.009 | 0.0013 | 0.0054 | 0 | 0 | 0 | 0.1521 | 0 | 0 | 0 | 0 | 0 | 0 | 0 |
| normal_tissue | German_2023 | 0.1858 | 0 | 0.1224 | 0.0574 | 0.0304 | 0.022 | 0.6198 | 0.1925 | 0.1925 | 0 | 0 | 0 | 0 | 0 | 0 | 0.0211 | 0 | 0 | 0 | 0 |
| normal_tissue | German_2023 | 3.8672 | 0 | 1.1901 | 0 | 0 | 0 | 1.1753 | 1.117 | 1.6282 | 0 | 0 | 0 | 0 | 0 | 0 | 0 | 0 | 0 | 0 | 0 |
| normal_tissue | German_2023 | 0.1117 | 0.0275 | 0 | 0 | 0.0275 | 0.2555 | 0.2922 | 0.303 | 0.0949 | 0 | 0 | 0 | 0 | 0 | 0 | 0 | 0 | 0 | 0 | 0 |
| normal_tissue | German_2023 | 0.6275 | 0.8797 | 0 | 0 | 0.0837 | 0.0043 | 0 | 0.0651 | 0 | 0 | 0 | 0 | 0 | 0 | 0 | 0.0265 | 0 | 0 | 0 | 0 |
| normal_tissue | German_2023 | 0.2244 | 0.0855 | 0.0588 | 0 | 0.0801 | 0.0908 | 0 | 0 | 0.0214 | 0 | 0 | 0 | 0.0481 | 0 | 0 | 0 | 0 | 0 | 0 | 0 |
| normal_tissue | German_2023 | 0.4363 | 0.0022 | 11.3704 | 0 | 0.0492 | 0.2322 | 0.2582 | 0.1065 | 0.1879 | 0 | 0 | 0 | 0 | 0 | 0 | 0 | 0 | 0 | 0 | 0 |
| normal_tissue | German_2023 | 0.4266 | 0.0314 | 0.1914 | 0 | 0.0185 | 0.0243 | 0.0588 | 0.0506 | 0.0544 | 0 | 0 | 0 | 0 | 0 | 0 | 0 | 0 | 0 | 0 | 0 |
| normal_tissue | German_2023 | 0.4562 | 0.0965 | 0.1579 | 0.0877 | 0.272 | 0.1404 | 0 | 0 | 0 | 0 | 0 | 0 | 0.0088 | 0 | 0 | 0 | 0 | 0 | 0 | 0 |
| normal_tissue | German_2023 | 2.1466 | 0.3045 | 0.0116 | 0.3531 | 0.0023 | 0.0787 | 0 | 0 | 0.0012 | 0.0012 | 0 | 0 | 0.1343 | 0 | 0 | 0 | 0 | 0 | 0 | 0 |
| normal_tissue | German_2023 | 0.6828 | 0.0212 | 0.5552 | 0 | 0.0005 | 0.0758 | 2.4163 | 0.6419 | 0.3607 | 0 | 0 | 0.0005 | 0 | 0 | 0 | 0 | 0 | 0 | 0 | 0 |
| normal_tissue | German_2023 | 0.4085 | 0.0371 | 0.1468 | 0 | 0.13 | 0.4236 | 0.5806 | 0.4692 | 0.1418 | 0.0287 | 0.0338 | 0 | 0 | 0 | 0 | 0 | 0 | 0 | 0 | 0 |
| normal_tissue | German_2023 | 0.2069 | 0.0631 | 0 | 0 | 0.1192 | 0.0877 | 0.5856 | 0.3577 | 0.3542 | 0 | 0 | 0 | 0 | 0 | 0 | 0 | 0 | 0 | 0 | 0 |
| normal_tissue | German_2023 | 15.2112 | 0.1979 | 0.0027 | 0.0014 | 0.37 | 1.1128 | 0 | 0 | 0.0014 | 0 | 0 | 0 | 0 | 0 | 0 | 0 | 0 | 0 | 0 | 0 |
| normal_tissue | German_2023 | 0.0439 | 0.0902 | 0.0634 | 0 | 0.061 | 0.0658 | 0.2146 | 0.3341 | 0.2122 | 0 | 0 | 0 | 0 | 0 | 0 | 0 | 0 | 0 | 0 | 0 |
| normal_tissue | German_2023 | 1.1873 | 0.0014 | 0.0643 | 0 | 0.1388 | 0.3542 | 0.0501 | 0.5059 | 0.9468 | 0 | 0 | 0 | 0 | 0 | 0 | 0 | 0 | 0 | 0 | 0 |
| normal_tissue | German_2023 | 0.3345 | 0.4326 | 0 | 0 | 0 | 0.4265 | 0.4779 | 0.8443 | 1.2389 | 0 | 0 | 0 | 0 | 0 | 0 | 0 | 0 | 0 | 0 | 0 |
| normal_tissue | German_2023 | 0.812 | 0.5941 | 1.3071 | 0 | 0 | 0.1436 | 2.7428 | 0.4753 | 4.0846 | 0 | 0 | 0 | 0 | 0 | 0 | 0 | 0 | 0 | 0 | 0 |
| normal_tissue | German_2023 | 0.1952 | 0.0049 | 0 | 0 | 0 | 0.4535 | 0.3213 | 0.8378 | 0.0877 | 0 | 0 | 0 | 0 | 0 | 0 | 0 | 0 | 0 | 0 | 0 |
| normal_tissue | German_2023 | 2.0427 | 0.1178 | 0.0016 | 0 | 0.1534 | 0.2294 | 1.1268 | 1.8134 | 1.4584 | 0 | 0 | 0 | 0 | 0 | 0 | 0 | 0 | 0 | 0 | 0 |
| normal_tissue | German_2023 | 0.2393 | 0.0972 | 0.2207 | 0 | 0 | 0 | 1.121 | 0.4053 | 0.1322 | 0 | 0 | 0 | 0 | 0 | 0 | 0 | 0 | 0 | 0 | 0 |
| normal_tissue | German_2023 | 0.1375 | 0 | 0 | 0 | 0 | 0.0501 | 0.1391 | 1.8101 | 0.9317 | 0 | 0 | 0 | 0 | 0 | 0 | 0 | 0 | 0 | 0 | 0 |
| normal_tissue | German_2023 | 23.049 | 0.2987 | 0.0836 | 0.3298 | 0 | 0 | 0.1289 | 0.0246 | 0.0759 | 0 | 0 | 0 | 0.5858 | 0 | 0 | 0.3707 | 0 | 0 | 0 | 0 |
| normal_tissue | German_2023 | 4.9481 | 0.1552 | 0.0038 | 0 | 0.5543 | 1.4098 | 0 | 0.0008 | 0 | 0 | 0 | 0 | 0.0031 | 0 | 0 | 0 | 0 | 0 | 0 | 0 |
| normal_tissue | German_2023 | 0.4402 | 0.8829 | 0.0182 | 0.0391 | 0.0026 | 0.2162 | 0 | 0 | 0.2526 | 0 | 0 | 0 | 0 | 0 | 0 | 0 | 0 | 0 | 0 | 0 |
| normal_tissue | German_2023 | 2.4203 | 0 | 0 | 0 | 0 | 0 | 1.0501 | 1.0377 | 0.5727 | 0 | 0 | 0 | 0 | 0 | 0 | 0 | 0 | 0 | 0 | 0 |
| normal_tissue | German_2023 | 0.0006 | 0 | 0.0013 | 0 | 0.0552 | 0.1142 | 1.0918 | 0.0019 | 0.0897 | 0 | 0 | 0 | 0 | 0 | 0 | 0 | 0 | 0 | 0 | 0 |
| normal_tissue | German_2023 | 2.1682 | 1.4488 | 0.9279 | 0.0015 | 0.991 | 0.5936 | 0 | 0 | 0 | 0 | 0 | 0 | 0.0085 | 0 | 0 | 0 | 0 | 0 | 0 | 0 |
| normal_tissue | German_2023 | 0.0791 | 0.0522 | 0 | 0 | 5.948 | 5.9968 | 0.1627 | 0.1821 | 0.0005 | 0.0005 | 0 | 0 | 0 | 0 | 0.001 | 0 | 0 | 0 | 0 | 0 |
| normal_tissue | German_2023 | 0.0113 | 0.0292 | 0 | 0 | 6.1911 | 7.5651 | 0.1366 | 0.2003 | 0.1943 | 0 | 0 | 0 | 0 | 0 | 0 | 0 | 0 | 0 | 0 | 0 |
| normal_tissue | German_2023 | 0.1595 | 0 | 0 | 0 | 4.4234 | 1.4622 | 0.336 | 0.7202 | 0.3677 | 0 | 0 | 0 | 0 | 0 | 0.0005 | 0 | 0 | 0 | 0 | 0 |
| normal_tissue | German_2023 | 0.9586 | 0.0644 | 0.3112 | 0 | 4.2304 | 0.6152 | 0.0051 | 0.3316 | 0.3516 | 0 | 0 | 0 | 0 | 0 | 0 | 0 | 0 | 0 | 0 | 0 |
| normal_tissue | German_2023 | 0.5265 | 0.1067 | 0.0602 | 0 | 8.5379 | 2.0224 | 0.5096 | 0.41 | 0.3169 | 0 | 0 | 0 | 0 | 0 | 0 | 0 | 0 | 0 | 0 | 0 |
| normal_tissue | German_2023 | 0.1945 | 0 | 0 | 0 | 3.6431 | 4.5596 | 0.1295 | 0.1086 | 0.2472 | 0 | 0 | 0 | 0 | 0 | 0.0005 | 0 | 0 | 0 | 0 | 0 |
| normal_tissue | German_2023 | 0.0545 | 0.0006 | 0.0209 | 0 | 3.6123 | 7.6168 | 0.1259 | 0.1665 | 0.0685 | 0 | 0 | 0 | 0 | 0 | 0 | 0 | 0 | 0 | 0 | 0 |
| normal_tissue | German_2023 | 0.0874 | 0 | 0.0004 | 0 | 3.1359 | 2.3743 | 0.1362 | 0.1264 | 0.0964 | 0 | 0 | 0 | 0 | 0 | 0.0004 | 0 | 0 | 0 | 0 | 0 |
| normal_tissue | German_2023 | 0.0737 | 0.094 | 0 | 0 | 5.2822 | 4.5675 | 0.2914 | 0.0446 | 0.1237 | 0.0663 | 0 | 0 | 0 | 0 | 0.0007 | 0 | 0 | 0 | 0 | 0 |
| normal_tissue | German_2023 | 0.0005 | 0.0334 | 0 | 0 | 2.8511 | 7.4437 | 0.0941 | 0.2559 | 0.0269 | 0.0065 | 0 | 0 | 0 | 0 | 0.0005 | 0 | 0 | 0 | 0 | 0 |
| normal_tissue | German_2023 | 0.0852 | 0.0189 | 0.1116 | 0 | 2.5862 | 3.1678 | 0.0624 | 0.1355 | 0.1943 | 0 | 0 | 0 | 0 | 0 | 0 | 0 | 0 | 0 | 0 | 0 |
| normal_tissue | German_2023 | 0.7159 | 0 | 0.0857 | 0 | 6.8606 | 0.9926 | 0.6691 | 0.3056 | 0.5071 | 0 | 0 | 0 | 0 | 0 | 0 | 0 | 0 | 0 | 0 | 0 |
| normal_tissue | German_2023 | 1.4696 | 0.0004 | 0.0942 | 0.9607 | 2.5329 | 1.7435 | 0.0939 | 0.0206 | 0.2442 | 0 | 0 | 0 | 0 | 0 | 0 | 0 | 0 | 0 | 0 | 0 |
| normal_tissue | German_2023 | 0.2929 | 0.0162 | 0.0512 | 0.3685 | 3.1141 | 2.7375 | 0.1285 | 0.2344 | 0.0393 | 0 | 0 | 0 | 0 | 0 | 0 | 0 | 0 | 0 | 0 | 0 |
| normal_tissue | German_2023 | 0.034 | 0.0371 | 0 | 0 | 2.7325 | 3.4109 | 0.2568 | 0.022 | 0.1636 | 0 | 0 | 0 | 0 | 0 | 0 | 0.0283 | 0 | 0 | 0 | 0 |
| normal_tissue | German_2023 | 0.0972 | 0 | 0.06 | 0 | 5.4081 | 5.4257 | 0.2222 | 0.3256 | 0.0569 | 0 | 0 | 0 | 0 | 0 | 0.0021 | 0 | 0 | 0 | 0 | 0 |
| normal_tissue | German_2023 | 0.7123 | 16.0597 | 0.0499 | 0.0034 | 0 | 0.0668 | 0.0346 | 0.3566 | 0.289 | 0.0008 | 0 | 0 | 0 | 0 | 0 | 0.0431 | 0 | 0 | 0 | 0 |
| normal_tissue | German_2023 | 0.0862 | 0 | 0.0825 | 0 | 0.0007 | 0.0004 | 0.2547 | 0.0622 | 0.0362 | 0 | 0 | 0 | 0 | 0 | 0 | 0 | 0 | 0 | 0 | 0 |
| normal_tissue | German_2023 | 1.2619 | 0.0174 | 0.1927 | 0.0569 | 0.3689 | 0.0209 | 0.0466 | 0.0383 | 0.0244 | 0.0261 | 0 | 0 | 0.2966 | 0 | 0 | 0 | 0 | 0 | 0 | 0 |
| normal_tissue | German_2023 | 0.7348 | 0.1531 | 0.001 | 0 | 0.0016 | 0 | 0.0036 | 0 | 0.0479 | 0 | 0 | 0 | 0 | 0 | 0 | 0 | 0 | 0 | 0 | 0 |
| normal_tissue | German_2023 | 0.0688 | 0.0004 | 0.0022 | 0.1469 | 0.0291 | 0.0607 | 0.0844 | 0.0754 | 0.0377 | 0 | 0 | 0 | 0 | 0 | 0 | 0.0142 | 0 | 0 | 0 | 0 |
| normal_tissue | German_2023 | 0.2522 | 0.0223 | 0.7779 | 0 | 0.0692 | 0.0126 | 0.0944 | 0.0074 | 0.0349 | 0 | 0 | 0 | 0 | 0 | 0 | 0 | 0 | 0 | 0 | 0 |
| normal_tissue | German_2023 | 0.0808 | 0.0144 | 0.0534 | 0 | 2.78 | 3.026 | 0.0427 | 0.065 | 0.0283 | 0 | 0 | 0 | 0 | 0 | 0.0002 | 0 | 0 | 0 | 0 | 0 |
| normal_tissue | German_2023 | 0.3776 | 0 | 0.0843 | 0 | 0 | 0.0139 | 0.0843 | 0.1501 | 0.1061 | 0 | 0 | 0.0355 | 0 | 0 | 0 | 0 | 0 | 0 | 0 | 0 |
| normal_tissue | German_2023 | 0.0715 | 0.0002 | 0.0285 | 0 | 0.0051 | 0.0248 | 0.0995 | 0.0993 | 0.0719 | 0 | 0 | 0 | 0 | 0 | 0 | 0 | 0 | 0 | 0 | 0 |
| normal_tissue | German_2023 | 3.2788 | 0.0272 | 3.4318 | 0 | 0.1392 | 0.0067 | 0.2375 | 0.379 | 0.0957 | 0.0964 | 0 | 0.2048 | 0 | 0 | 0 | 0 | 0 | 0 | 0 | 0 |
| normal_tissue | German_2023 | 0.1066 | 0.0208 | 0.0131 | 0 | 3.6504 | 3.922 | 0.2831 | 0.1081 | 0.2173 | 0 | 0 | 0 | 0 | 0 | 0 | 0 | 0 | 0 | 0 | 0 |
| normal_tissue | German_2023 | 0.3545 | 0.0713 | 0.1681 | 0.0861 | 0.0715 | 0.0185 | 0.1778 | 0.0465 | 0.1968 | 0 | 0 | 0 | 0 | 0 | 0 | 0 | 0 | 0 | 0 | 0 |
| normal_tissue | German_2023 | 0.0667 | 0 | 0.0094 | 0 | 0.0055 | 0.0374 | 0.1021 | 0.1356 | 0.0647 | 0 | 0 | 0 | 0 | 0 | 0 | 0 | 0 | 0 | 0 | 0 |
| normal_tissue | German_2023 | 1.8206 | 1.124 | 0.0633 | 0 | 0 | 0 | 1.1874 | 0.2691 | 0.6412 | 0 | 0 | 0 | 0.0317 | 0 | 0 | 0.6253 | 0 | 0 | 0 | 0 |
| normal_tissue | German_2023 | 0.0664 | 0.0399 | 0.0473 | 0 | 0.0158 | 0 | 0.0938 | 0.0641 | 0.0691 | 0 | 0 | 0 | 0 | 0 | 0 | 0 | 0 | 0 | 0 | 0 |
| normal_tissue | German_2023 | 0.4737 | 0.1998 | 0.0568 | 0.0003 | 0.2668 | 0.0815 | 0.3006 | 0.3148 | 0.3608 | 0 | 0 | 0 | 0 | 0 | 0 | 0 | 0 | 0 | 0 | 0 |
| normal_tissue | German_2023 | 0.1434 | 0.0305 | 0.0037 | 0 | 0.0016 | 0.1065 | 0.2445 | 0.2852 | 0.2416 | 0 | 0 | 0 | 0 | 0 | 0 | 0 | 0 | 0 | 0 | 0 |
| normal_tissue | German_2023 | 0.0466 | 0.0203 | 0.0151 | 0 | 0.0004 | 0.0008 | 0.14 | 0.1261 | 0.0753 | 0 | 0 | 0 | 0.0006 | 0 | 0 | 0 | 0 | 0 | 0 | 0 |
| normal_tissue | German_2023 | 12.8971 | 0 | 2.9199 | 0 | 0.4795 | 0.2072 | 0 | 0.0017 | 0 | 0 | 0 | 0 | 0.0017 | 0 | 0 | 0 | 0 | 0 | 0 | 0 |
| normal_tissue | German_2023 | 0.6245 | 0 | 0.0003 | 0 | 0.0252 | 0.1244 | 0.1858 | 0.3572 | 0.0928 | 0 | 0 | 0 | 0.0003 | 0 | 0 | 0.0928 | 0 | 0 | 0 | 0 |
| normal_tissue | German_2023 | 0.0199 | 1.6066 | 0 | 0 | 0.0033 | 0.0663 | 0 | 0 | 0 | 0 | 0 | 0 | 0.0629 | 0 | 0 | 0 | 0 | 0 | 0 | 0 |
| normal_tissue | German_2023 | 4.9889 | 0.3144 | 0.003 | 0 | 0 | 0 | 0 | 4.8999 | 0 | 0 | 0 | 0 | 0 | 0 | 0 | 0 | 0 | 0 | 0 | 0 |
| normal_tissue | German_2023 | 1.4011 | 0 | 0.9478 | 0 | 0.5115 | 0.0648 | 0.5088 | 1.3166 | 0.3915 | 0 | 0 | 0 | 0 | 0 | 0 | 0 | 0 | 0 | 0 | 0 |
| normal_tissue | German_2023 | 1.1712 | 0.0628 | 0.3315 | 0.0749 | 0.0155 | 0.0814 | 0.171 | 0.4352 | 0.5383 | 0 | 0 | 0 | 0 | 0 | 0 | 0 | 0 | 0 | 0 | 0 |
| normal_tissue | German_2023 | 0.1309 | 0.0472 | 0.0078 | 0 | 5.1511 | 5.5389 | 0.1562 | 0.0842 | 0.0735 | 0.0209 | 0 | 0 | 0 | 0 | 0.001 | 0.0146 | 0 | 0 | 0 | 0 |
| normal_tissue | German_2023 | 0.4628 | 0.4839 | 0.0151 | 0 | 0 | 0.3889 | 0 | 0 | 0 | 0 | 0 | 0 | 0 | 0 | 0 | 0.0015 | 0 | 0 | 0 | 0 |
| normal_tissue | German_2023 | 0.1268 | 1.818 | 0 | 0 | 0 | 0.2609 | 3.7497 | 3.1333 | 2.3743 | 0 | 0 | 0 | 0 | 0 | 0 | 0 | 0 | 0 | 0 | 0 |
| normal_tissue | German_2023 | 0.0003 | 2.9006 | 0.1358 | 0 | 0.099 | 0.0467 | 0.0841 | 0.7957 | 0.4689 | 0 | 0 | 0 | 0.0003 | 0 | 0 | 0 | 0 | 0 | 0 | 0 |
| normal_tissue | German_2023 | 0.0151 | 0.0478 | 0.0045 | 0.0069 | 0 | 0.0034 | 0.0162 | 0.063 | 0.0155 | 0 | 0 | 0 | 0 | 0 | 0 | 0 | 0 | 0 | 0 | 0 |
| normal_tissue | German_2023 | 0.1653 | 0.069 | 0.0003 | 0.0008 | 0.0353 | 0.0434 | 0.0312 | 0.0677 | 0.2593 | 0 | 0 | 0 | 0 | 0 | 0 | 0 | 0 | 0 | 0 | 0 |
| normal_tissue | German_2023 | 0.1349 | 0.0005 | 0.0253 | 0 | 0.0013 | 0.042 | 0.067 | 0.0668 | 0.0366 | 0 | 0 | 0 | 0 | 0 | 0 | 0 | 0 | 0 | 0 | 0 |
| normal_tissue | German_2023 | 1.5485 | 0.4522 | 0.265 | 0 | 0.0121 | 0.079 | 0.6099 | 0.6396 | 0.4256 | 0 | 0 | 0 | 0 | 0 | 0 | 0.0003 | 0 | 0 | 0 | 0 |
| normal_tissue | German_2023 | 4.5183 | 0.039 | 0.55 | 0.5074 | 0.0005 | 0.0058 | 0 | 0 | 0.0005 | 0.2292 | 0 | 0 | 0.0016 | 0 | 0 | 0 | 0 | 0 | 0 | 0 |
| normal_tissue | German_2023 | 0.0428 | 0.0417 | 0.0665 | 0 | 2.6537 | 4.1115 | 0.1279 | 0.2637 | 0.2254 | 0.0259 | 0 | 0 | 0 | 0 | 0 | 0 | 0 | 0 | 0 | 0 |
| normal_tissue | German_2023 | 0.8723 | 0.0107 | 0 | 0 | 0.4998 | 0.5038 | 0.1018 | 0.2787 | 0.0844 | 0 | 0 | 0 | 0 | 0 | 0 | 0 | 0 | 0 | 0 | 0 |
| normal_tissue | German_2023 | 4.0327 | 32.84 | 0.0623 | 0 | 0 | 0.2745 | 0.6628 | 0.7996 | 0.9888 | 0 | 0 | 0 | 0.0005 | 0 | 0 | 0 | 0 | 0 | 0 | 0 |
| normal_tissue | German_2023 | 0.2404 | 0.1311 | 0 | 0 | 0 | 0.0182 | 0.8412 | 0.8776 | 0.4006 | 0 | 0 | 0 | 0 | 0 | 0 | 0 | 0 | 0 | 0 | 0 |
| normal_tissue | German_2023 | 1.3999 | 0.0058 | 0.3485 | 0 | 0.0116 | 0.0058 | 0.9642 | 0.3195 | 1.0688 | 0 | 0 | 0 | 0 | 0 | 0 | 0 | 0 | 0 | 0 | 0 |
| normal_tissue | German_2023 | 2.1633 | 0.004 | 0 | 0 | 0.004 | 0.012 | 0.6089 | 1.8789 | 0.7171 | 0 | 0 | 0 | 0 | 0 | 0 | 0 | 0 | 0 | 0 | 0 |
| normal_tissue | German_2023 | 0.0903 | 0 | 0.0044 | 0 | 4.1626 | 3.7679 | 0.1095 | 0.0914 | 0.0848 | 0 | 0 | 0 | 0 | 0 | 0 | 0 | 0 | 0 | 0 | 0 |
| normal_tissue | German_2023 | 0.3613 | 0.4094 | 0.2003 | 0 | 3.7279 | 0.0999 | 2.1876 | 3.0535 | 3.6779 | 0 | 0 | 0 | 0 | 0 | 0 | 0 | 0 | 0 | 0 | 0 |
| normal_tissue | German_2023 | 6.3689 | 0 | 0 | 0 | 0 | 0.0085 | 2.6305 | 0.9558 | 1.2856 | 0 | 0 | 0 | 0 | 0 | 0 | 0 | 0 | 0 | 0 | 0 |
| normal_tissue | German_2023 | 0.0807 | 0.0198 | 0.055 | 0 | 4.2327 | 4.9273 | 0.2917 | 0.1004 | 0.133 | 0 | 0 | 0 | 0 | 0 | 0 | 0 | 0 | 0 | 0 | 0 |
| normal_tissue | German_2023 | 1.4797 | 0.0039 | 0.5363 | 0 | 0.3327 | 0.5833 | 0.826 | 1.1939 | 0.4306 | 0 | 0 | 0 | 0 | 0 | 0 | 0 | 0 | 0 | 0 | 0 |
| normal_tissue | German_2023 | 0.637 | 51.0621 | 0.1374 | 0.0006 | 0.0006 | 0.0011 | 0.6218 | 0.1374 | 0.29 | 0 | 0 | 0 | 0 | 0 | 0 | 0 | 0 | 0 | 0 | 0 |
| normal_tissue | German_2023 | 1.5881 | 17.166 | 0.001 | 0.0005 | 0 | 0.0778 | 0.4266 | 0.2291 | 0.478 | 0 | 0 | 0 | 0 | 0 | 0 | 0.6091 | 0 | 0 | 0 | 0 |
| normal_tissue | German_2023 | 3.3872 | 13.1339 | 0.3819 | 0.1143 | 0.0004 | 0 | 1.4164 | 0.5956 | 0.7029 | 0 | 0 | 0 | 0 | 0 | 0 | 0 | 0 | 0 | 0 | 0 |
| normal_tissue | German_2023 | 5.2936 | 3.6368 | 0.116 | 1.405 | 0 | 0 | 0.3678 | 1.352 | 0.5318 | 0 | 0 | 0 | 0.0017 | 0 | 0 | 0.3761 | 0.0414 | 0 | 0 | 0 |
| normal_tissue | German_2023 | 0.001 | 0.0283 | 0.0101 | 0 | 3.2123 | 5.0766 | 0.0941 | 0.457 | 0.264 | 0 | 0 | 0 | 0 | 0 | 0.0005 | 0 | 0 | 0 | 0 | 0 |
| normal_tissue | German_2023 | 2.3747 | 0.0087 | 0.2697 | 0 | 0.6654 | 1.1656 | 4.7451 | 2.1703 | 0.4567 | 0 | 0 | 0 | 0 | 0 | 0 | 0 | 0 | 0 | 0 | 0 |
| normal_tissue | German_2023 | 6.4991 | 8.2563 | 3.0474 | 1.0928 | 0.0007 | 0.0578 | 0.2972 | 0.7221 | 0.2373 | 0 | 0 | 0 | 0.7221 | 0 | 0 | 0.0634 | 0 | 0 | 0 | 0 |
| normal_tissue | German_2023 | 0.087 | 0.0656 | 0.0733 | 0 | 5.4913 | 5.6648 | 0.0972 | 0.0626 | 0.0143 | 0 | 0 | 0 | 0 | 0 | 0.0006 | 0 | 0 | 0 | 0 | 0 |
| normal_tissue | German_2023 | 0.0674 | 0.0772 | 0.1067 | 0.0005 | 2.2068 | 0.7038 | 0.1264 | 0.1544 | 0.3182 | 0 | 0 | 0 | 0 | 0 | 0 | 0 | 0 | 0 | 0 | 0 |
| normal_tissue | German_2023 | 1.9264 | 1.5374 | 0 | 0 | 0 | 0 | 4.8047 | 6.4638 | 2.9657 | 0 | 0 | 0 | 0 | 0 | 0 | 0 | 0 | 0 | 0 | 0 |
| normal_tissue | German_2023 | 9.5544 | 0.3607 | 0.2705 | 0 | 0.0225 | 0.9693 | 4.5612 | 0.2592 | 0.3457 | 0 | 0 | 0 | 0 | 0 | 0 | 0 | 0 | 0 | 0 | 0 |
| normal_tissue | German_2023 | 2.024 | 0.0115 | 0.0003 | 0 | 0.2403 | 0.2314 | 2.978 | 3.5803 | 1.8222 | 0 | 0 | 0 | 0 | 0 | 0 | 0 | 0 | 0 | 0 | 0 |
| normal_tissue | German_2023 | 5.1045 | 5.8538 | 20.5089 | 1.3968 | 0.0816 | 0.118 | 0.5474 | 0.7262 | 0.2557 | 0 | 0 | 0 | 0 | 0 | 0 | 0 | 0 | 0 | 0 | 0 |
| normal_tissue | German_2023 | 0.1701 | 0 | 0 | 0 | 0 | 0.0067 | 0.4001 | 1.7006 | 0.8203 | 0 | 0 | 0 | 0 | 0 | 0 | 0 | 0 | 0 | 0 | 0 |
| normal_tissue | German_2023 | 3.3016 | 0.0056 | 0.174 | 0 | 0.0042 | 0.0056 | 1.0787 | 0.3187 | 0.9047 | 0 | 0 | 0 | 0 | 0 | 0 | 0 | 0 | 0 | 0 | 0 |
| normal_tissue | German_2023 | 0.0619 | 0.072 | 0.1359 | 0 | 5.819 | 6.9646 | 0.3064 | 0.0815 | 0.0423 | 0 | 0 | 0 | 0 | 0 | 0.0005 | 0 | 0 | 0 | 0 | 0 |
| normal_tissue | German_2023 | 0.1432 | 0.0193 | 0 | 0 | 0.0008 | 0.0369 | 0.2937 | 0.6159 | 0.4145 | 0 | 0 | 0 | 0 | 0 | 0 | 0.0004 | 0 | 0 | 0.0839 | 0 |
| normal_tissue | German_2023 | 0.1782 | 0 | 0 | 0 | 0.0055 | 0 | 1.08 | 0.9279 | 0.7648 | 0 | 0 | 0 | 0.2823 | 0 | 0 | 0 | 0 | 0 | 0 | 0 |
| normal_tissue | German_2023 | 0.2257 | 0.0297 | 0 | 0 | 2.5385 | 2.6354 | 0.0794 | 0.0003 | 0.0679 | 0 | 0 | 0 | 0 | 0 | 0 | 0 | 0 | 0 | 0 | 0 |
| normal_tissue | German_2023 | 12.5222 | 0.0184 | 0.0069 | 0 | 0 | 0 | 2.8628 | 2.0244 | 0.0008 | 0 | 0 | 0.2419 | 8.5209 | 0 | 0 | 0.8323 | 0 | 0 | 0 | 0 |
| normal_tissue | German_2023 | 0.4108 | 0 | 0.0004 | 0.2737 | 0.0289 | 0.0158 | 0.1288 | 0.6543 | 0.1485 | 0 | 0 | 0 | 0 | 0 | 0 | 0 | 0 | 0 | 0 | 0 |
| normal_tissue | German_2023 | 0.0525 | 0.0704 | 0 | 0 | 0.0001 | 0.0427 | 0.1352 | 0.2744 | 0.2359 | 0 | 0 | 0 | 0 | 0 | 0 | 0 | 0 | 0 | 0 | 0 |
| normal_tissue | German_2023 | 0.0939 | 0.0109 | 0 | 0 | 0.0208 | 0.0615 | 0.1395 | 0.0962 | 0.1078 | 0 | 0 | 0 | 0 | 0 | 0 | 0 | 0 | 0 | 0 | 0 |
| normal_tissue | German_2023 | 0.522 | 0.0009 | 0 | 0 | 0 | 0.1411 | 0.313 | 0.5361 | 0.7636 | 0 | 0 | 0 | 0 | 0 | 0 | 0 | 0 | 0 | 0 | 0 |
| normal_tissue | German_2023 | 4.6417 | 0.0761 | 0.0004 | 0 | 0.0004 | 0.1266 | 0.1665 | 0.2818 | 0.1367 | 0 | 0 | 0 | 0 | 0 | 0 | 0 | 0 | 0 | 0 | 0 |
| normal_tissue | German_2023 | 0.1769 | 0.0209 | 0.0131 | 0 | 0.0034 | 0.0558 | 0.0145 | 0.1332 | 0.1641 | 0 | 0 | 0 | 0 | 0 | 0 | 0 | 0 | 0 | 0 | 0 |
| normal_tissue | German_2023 | 0.1147 | 0.0975 | 0.0012 | 0 | 0 | 0.0585 | 0.1467 | 0.2029 | 0.2134 | 0 | 0 | 0 | 0 | 0 | 0 | 0 | 0 | 0 | 0 | 0 |
| normal_tissue | German_2023 | 0.1986 | 0.0115 | 0 | 0.0587 | 0 | 0.0327 | 0.0813 | 0.0648 | 0.2087 | 0 | 0 | 0 | 0 | 0 | 0 | 0 | 0 | 0 | 0 | 0 |
| normal_tissue | German_2023 | 0.1629 | 0.0706 | 0 | 0 | 0.02 | 0 | 0.3398 | 0.1056 | 0.2315 | 0 | 0 | 0 | 0 | 0 | 0 | 0 | 0 | 0 | 0 | 0 |
| normal_tissue | German_2023 | 0.0452 | 0.0013 | 0 | 0 | 0.01 | 0.0007 | 0.072 | 0.0378 | 0.078 | 0 | 0 | 0 | 0 | 0 | 0 | 0 | 0 | 0 | 0 | 0 |
| normal_tissue | German_2023 | 2.5581 | 0.267 | 0 | 0.0608 | 0 | 0.0763 | 0.0763 | 0 | 0 | 0 | 0 | 0 | 0.3587 | 0 | 0 | 0.1919 | 0.351 | 0 | 0 | 0 |
| normal_tissue | German_2023 | 0.1351 | 0 | 0 | 0 | 7.3471 | 1.7334 | 0.2065 | 0.5859 | 0.103 | 0 | 0 | 0 | 0 | 0 | 0.0014 | 0 | 0 | 0 | 0 | 0 |
| normal_tissue | German_2023 | 0.1323 | 0.0007 | 0 | 0 | 0 | 0.0306 | 0.1023 | 0.1138 | 0.0922 | 0 | 0 | 0 | 0 | 0 | 0 | 0.0024 | 0 | 0 | 0 | 0 |
| normal_tissue | German_2023 | 0.4308 | 0.1086 | 0.1896 | 0 | 0.1528 | 0.3056 | 0.2485 | 0.1012 | 0.3295 | 0 | 0 | 0.0018 | 0 | 0 | 0 | 0 | 0 | 0 | 0 | 0 |
| normal_tissue | German_2023 | 1.312 | 0.0362 | 0 | 0 | 0.2537 | 0 | 0.3053 | 0.828 | 0.2996 | 0 | 0 | 0 | 0 | 0 | 0 | 0 | 0 | 0 | 0 | 0 |
| normal_tissue | German_2023 | 0.2061 | 0.0318 | 0.0186 | 0 | 3.1501 | 4.8429 | 0.0005 | 0.0568 | 0.0984 | 0 | 0 | 0 | 0 | 0 | 0 | 0 | 0 | 0 | 0 | 0 |
| normal_tissue | German_2023 | 0.575 | 0.0294 | 0.0028 | 0 | 0.0742 | 0.0784 | 0.2616 | 0.3106 | 0.8605 | 0.2532 | 0 | 0 | 0 | 0 | 0 | 0 | 0 | 0 | 0 | 0 |
| normal_tissue | German_2023 | 0.9838 | 0 | 0.2337 | 0 | 0.0452 | 0 | 0.2586 | 0.0534 | 0.0488 | 0 | 0 | 0 | 0 | 0 | 0 | 0 | 0 | 0 | 0 | 0 |
| normal_tissue | German_2023 | 0.012 | 0.002 | 0.0007 | 0 | 0.0013 | 0.0756 | 0 | 0.0007 | 0 | 0 | 0 | 0.5749 | 0 | 0 | 0 | 0.0007 | 0 | 0 | 0 | 0 |
| normal_tissue | German_2023 | 0.8156 | 0.1352 | 0 | 0 | 0.1138 | 0.0754 | 0.7768 | 0.8426 | 0.4008 | 0 | 0 | 0 | 0 | 0 | 0 | 0 | 0 | 0 | 0 | 0 |
| normal_tissue | German_2023 | 3.0938 | 1.584 | 0.4236 | 0.1392 | 0 | 0.0817 | 0 | 0 | 0 | 0 | 0 | 0.3767 | 1.0439 | 0 | 0 | 0 | 0 | 0 | 0 | 0 |
| normal_tissue | German_2023 | 0.8875 | 0.1779 | 0.0006 | 0.0639 | 0.0656 | 0.1689 | 0.2047 | 0.0203 | 0.2996 | 0 | 0 | 0 | 0 | 0 | 0 | 0 | 0 | 0 | 0 | 0 |
| normal_tissue | German_2023 | 10.8384 | 0.2395 | 1.1629 | 0 | 0.3649 | 0.2293 | 2.1275 | 1.0686 | 0 | 0 | 0 | 0.0581 | 0.0005 | 0 | 0 | 0.6584 | 0 | 0 | 0 | 0 |
| normal_tissue | German_2023 | 0.2547 | 0.1862 | 0 | 0 | 0 | 0.3763 | 0.0038 | 0 | 0 | 0 | 0 | 0 | 0.8818 | 0 | 0 | 0 | 0 | 0 | 0 | 0 |
| normal_tissue | German_2023 | 2.2176 | 0 | 0.489 | 0 | 0.1392 | 0.9984 | 0.8864 | 0.6724 | 0.5128 | 0 | 0 | 0 | 0 | 0.0034 | 0 | 0 | 0 | 0 | 0 | 0 |
| normal_tissue | German_2023 | 0.9478 | 0.1411 | 0.0003 | 0 | 0 | 0.1185 | 0.3239 | 0.545 | 0.2332 | 0 | 0 | 0 | 0 | 0 | 0 | 0 | 0 | 0 | 0 | 0 |
| normal_tissue | German_2023 | 0.0485 | 0.1094 | 0.0036 | 0 | 0.0594 | 0.1709 | 0.3034 | 0.5576 | 0.2339 | 0 | 0 | 0 | 0 | 0 | 0 | 0 | 0 | 0 | 0 | 0 |
| normal_tissue | German_2023 | 0.2188 | 0.0931 | 0.0005 | 0 | 0 | 0.0725 | 0.2754 | 0.0393 | 0.31 | 0 | 0 | 0 | 0 | 0 | 0 | 0 | 0 | 0 | 0 | 0 |
| normal_tissue | German_2023 | 2.0956 | 0.1308 | 0.2516 | 0 | 0 | 0 | 4.0201 | 1.1472 | 2.6289 | 0 | 0 | 0 | 0 | 0 | 0 | 0 | 0 | 0 | 0 | 0 |
| normal_tissue | German_2023 | 1.5115 | 0.9027 | 0.0742 | 0 | 0.001 | 0.001 | 4.7561 | 4.9697 | 2.5776 | 0 | 0 | 1.3109 | 0 | 0 | 0 | 0 | 0 | 0 | 0 | 0 |
| normal_tissue | German_2023 | 0.7268 | 0.0007 | 0.12 | 0 | 0.1883 | 0.0004 | 1.5665 | 1.0179 | 0.628 | 0 | 0 | 0 | 0 | 0 | 0 | 0 | 0 | 0 | 0 | 0 |
| normal_tissue | German_2023 | 0.1972 | 0.001 | 0.0472 | 0.0572 | 0.001 | 0.0783 | 0.0522 | 0.4673 | 0.0934 | 0 | 0 | 0 | 0 | 0 | 0 | 0 | 0 | 0 | 0 | 0 |
| normal_tissue | German_2023 | 0.0222 | 0.0996 | 0 | 0 | 0.0855 | 0.1402 | 0.2011 | 0.2994 | 0.186 | 0 | 0 | 0 | 0 | 0 | 0 | 0 | 0 | 0 | 0 | 0 |
| normal_tissue | German_2023 | 0.1678 | 0.1055 | 0 | 0 | 7.7641 | 1.0761 | 0.1332 | 0.2755 | 0.3264 | 0.0103 | 0 | 0 | 0 | 0 | 0 | 0 | 0 | 0 | 0 | 0 |
| normal_tissue | German_2023 | 0.0028 | 0.0008 | 0 | 0 | 0 | 0.0949 | 0.1498 | 0.3206 | 0.0349 | 0.0003 | 0 | 0 | 0 | 0 | 0 | 0 | 0 | 0 | 0 | 0 |
| normal_tissue | German_2023 | 0.0469 | 0.0266 | 0.0016 | 0 | 0.0145 | 0.0211 | 0.271 | 0.1373 | 0.091 | 0 | 0 | 0 | 0 | 0 | 0 | 0 | 0 | 0 | 0 | 0 |
| normal_tissue | German_2023 | 0.5233 | 0.0771 | 0.0216 | 0 | 0.0118 | 0 | 0.3201 | 0.1823 | 0.6631 | 0 | 0 | 0 | 0 | 0 | 0 | 0 | 0 | 0 | 0 | 0 |
| normal_tissue | German_2023 | 0.1707 | 0.0695 | 0.0224 | 0 | 0.0487 | 0.0754 | 0.3788 | 0.1868 | 0.0813 | 0 | 0 | 0 | 0 | 0 | 0 | 0 | 0 | 0 | 0 | 0 |
| normal_tissue | German_2023 | 1.2999 | 0.0338 | 0 | 0 | 0.0569 | 0.042 | 0.4211 | 0.1444 | 0.305 | 0 | 0 | 0 | 0 | 0 | 0 | 0 | 0 | 0 | 0 | 0 |
| normal_tissue | German_2023 | 0.0574 | 0.001 | 0.0193 | 0 | 4.1829 | 4.6479 | 0.0452 | 0.0833 | 0.0717 | 0.0005 | 0 | 0 | 0.0005 | 0 | 0 | 0.0005 | 0 | 0 | 0 | 0 |
| normal_tissue | German_2023 | 7.6772 | 0.5161 | 0 | 0 | 0 | 0 | 0 | 0 | 0 | 0 | 0 | 0 | 0 | 0 | 0 | 0.8214 | 0 | 0 | 0 | 0 |
| normal_tissue | German_2023 | 0.9611 | 0.0161 | 0 | 0 | 0.0907 | 0.0491 | 0.2033 | 0.563 | 0.5576 | 0 | 0 | 0 | 0 | 0 | 0 | 0 | 0 | 0 | 0 | 0 |
| normal_tissue | German_2023 | 0.014 | 0.0081 | 0 | 0 | 0 | 0.0188 | 0.1696 | 0.1775 | 0.0423 | 0.0003 | 0 | 0 | 0 | 0 | 0 | 0 | 0 | 0 | 0 | 0 |
| normal_tissue | German_2023 | 0.2304 | 0 | 0 | 0 | 0 | 0.0171 | 0.2515 | 0.3791 | 0.1227 | 0 | 0 | 0 | 0 | 0 | 0 | 0 | 0 | 0 | 0 | 0 |
| normal_tissue | German_2023 | 3.6795 | 0.0589 | 0.7694 | 0.0539 | 0 | 0.1679 | 0.2548 | 0.5526 | 0.1996 | 0 | 0 | 0 | 0 | 0 | 0 | 0 | 0 | 0 | 0 | 0 |
| normal_tissue | German_2023 | 0.0141 | 0.1625 | 0.0021 | 0.0017 | 0.16 | 0.0381 | 0.1534 | 0.5671 | 0.0224 | 0 | 0 | 0 | 0 | 0 | 0 | 0 | 0 | 0 | 0 | 0 |
| normal_tissue | German_2023 | 0.3231 | 0.0452 | 0.0223 | 0 | 0.0298 | 0.0696 | 0.2908 | 0.2255 | 0.074 | 0.0111 | 0 | 0 | 0 | 0 | 0 | 0 | 0 | 0 | 0 | 0 |
| normal_tissue | German_2023 | 10.2798 | 1.5791 | 0 | 0 | 0.0012 | 0 | 2.2285 | 3.9307 | 2.6623 | 0 | 0 | 0 | 0 | 0 | 0 | 0 | 0 | 0 | 0 | 0 |
| normal_tissue | German_2023 | 0.0413 | 0.012 | 0 | 0 | 0 | 0.0051 | 0.0595 | 0.1341 | 0.1051 | 0 | 0 | 0 | 0 | 0 | 0 | 0.0108 | 0 | 0 | 0 | 0 |
| normal_tissue | German_2023 | 8.8291 | 0 | 0.0014 | 0 | 0.0544 | 0.0404 | 0 | 0 | 0 | 0 | 0 | 0 | 0 | 0 | 0 | 1.4827 | 0 | 0 | 0 | 0 |
| normal_tissue | German_2023 | 0.0536 | 0 | 0.0005 | 0 | 0.0979 | 0.0399 | 0.1088 | 0.1159 | 0.1782 | 0 | 0 | 0 | 0 | 0 | 0 | 0 | 0 | 0 | 0 | 0 |
| normal_tissue | German_2023 | 0.1886 | 0 | 1.1062 | 0 | 0 | 0 | 2.4277 | 1.3201 | 3.3734 | 0 | 0 | 0 | 0 | 0 | 0 | 0 | 0 | 0 | 0 | 0 |
| normal_tissue | German_2023 | 0.0636 | 0.0978 | 0.0007 | 0 | 0.0036 | 0.0002 | 0.0016 | 0.0925 | 0.1027 | 0 | 0 | 0 | 0 | 0 | 0 | 0 | 0 | 0 | 0 | 0 |
| normal_tissue | German_2023 | 0.1062 | 0 | 0.0452 | 0 | 0 | 0.0279 | 0.0534 | 0.1648 | 0.0516 | 0 | 0 | 0 | 0 | 0 | 0 | 0 | 0 | 0 | 0 | 0 |
| normal_tissue | German_2023 | 0.3021 | 0 | 9.053 | 0 | 0 | 0.3107 | 0.7533 | 0.7105 | 0.0003 | 0 | 0 | 0.1528 | 0 | 0 | 0 | 0 | 0 | 0 | 0 | 0 |
| normal_tissue | German_2023 | 32.1636 | 0.0822 | 0.5134 | 0.6747 | 0.0011 | 0 | 0.9045 | 0.7274 | 0 | 0 | 0 | 0 | 0.0011 | 0 | 0 | 0.5819 | 0 | 0 | 0 | 0 |
| normal_tissue | German_2023 | 2.2162 | 0.2267 | 0 | 0 | 0 | 0.0063 | 0.5603 | 1.2529 | 0.3715 | 0 | 0 | 0 | 0 | 0 | 0 | 0 | 0 | 0 | 0 | 0 |
| normal_tissue | German_2023 | 0.4075 | 0.7101 | 0.0004 | 0 | 0 | 0.0875 | 0.2996 | 0.3174 | 0.0789 | 0.0562 | 0 | 0 | 0 | 0 | 0 | 0 | 0 | 0 | 0 | 0 |
| normal_tissue | German_2023 | 51.9496 | 0.0079 | 0.1902 | 0 | 0 | 0 | 0.6561 | 1.1762 | 1.8171 | 0 | 0 | 0 | 0 | 0 | 0 | 0 | 0 | 0 | 0 | 0 |
| normal_tissue | German_2023 | 1.85 | 0.0119 | 0.0051 | 0 | 0.0004 | 0.0041 | 0.0119 | 0.0139 | 0.0438 | 0 | 0 | 0 | 0 | 0 | 0 | 0 | 0 | 0 | 0 | 0 |
| normal_tissue | German_2023 | 0.2238 | 0.0147 | 0.0079 | 0 | 0 | 0.0003 | 0.0594 | 0.0872 | 0.1047 | 0 | 0 | 0 | 0 | 0 | 0 | 0 | 0 | 0 | 0 | 0 |
| normal_tissue | German_2023 | 0.0072 | 0.0052 | 0.0275 | 0 | 0.0455 | 0.0481 | 0.0281 | 0.0667 | 0.1079 | 0.0003 | 0 | 0 | 0 | 0 | 0 | 0 | 0 | 0 | 0 | 0 |
| normal_tissue | German_2023 | 0.3087 | 0.0209 | 0.0645 | 0 | 0.0868 | 0.1397 | 0.0919 | 0.1649 | 0.1572 | 0 | 0 | 0 | 0 | 0 | 0 | 0.0002 | 0 | 0 | 0 | 0 |
| normal_tissue | German_2023 | 0.0011 | 0.002 | 0.1214 | 0.0002 | 0.0544 | 0.0617 | 0.1311 | 0.326 | 0.1493 | 0 | 0 | 0 | 0 | 0 | 0 | 0 | 0 | 0 | 0 | 0 |
| normal_tissue | German_2023 | 3.4667 | 0.1765 | 0.1334 | 0 | 0 | 0.0377 | 0 | 0 | 0.0013 | 0 | 0 | 0.0027 | 0 | 0 | 0 | 0.0013 | 0 | 0 | 0 | 0 |
| normal_tissue | German_2023 | 0.5875 | 0.0103 | 0.438 | 0.1185 | 0 | 0.0026 | 0.9456 | 0.2525 | 0.7627 | 0 | 0 | 0 | 0 | 0 | 0 | 0.3865 | 0 | 0 | 0 | 0 |
| normal_tissue | German_2023 | 0.8722 | 0.2719 | 0.0103 | 0 | 0.0051 | 0.0308 | 0.2924 | 0 | 0 | 0 | 0 | 0.0103 | 0.3591 | 0 | 0 | 0 | 0 | 0 | 0 | 0 |
| normal_tissue | German_2023 | 0.0057 | 0 | 0 | 0 | 3.4083 | 3.6128 | 0.1291 | 0.2029 | 0.2489 | 0 | 0 | 0 | 0.1363 | 0 | 0 | 0 | 0 | 0 | 0 | 0 |
| normal_tissue | German_2023 | 0.6821 | 0.0296 | 0.0012 | 0 | 0.1635 | 0.059 | 0.262 | 0.1952 | 0.1752 | 0 | 0 | 0 | 0 | 0 | 0 | 0 | 0 | 0 | 0 | 0 |
| normal_tissue | German_2023 | 0.9898 | 0.1939 | 0.2123 | 0.297 | 0.0079 | 0.3791 | 0.4827 | 1.3465 | 0.808 | 0 | 0 | 0 | 0.0005 | 0 | 0 | 0 | 0 | 0 | 0 | 0 |
| normal_tissue | German_2023 | 0.6398 | 0.2635 | 0.0798 | 0 | 0.0718 | 0.1898 | 0.5416 | 1.1224 | 0.2578 | 0 | 0 | 0 | 0 | 0 | 0 | 0 | 0 | 0 | 0 | 0 |
| normal_tissue | German_2023 | 0.0708 | 0 | 0 | 0 | 0 | 0.0455 | 0.0775 | 0 | 0.0111 | 0 | 0 | 0 | 0 | 0 | 0 | 0 | 0 | 0 | 0 | 0 |
| normal_tissue | German_2023 | 0.5661 | 0.0386 | 0.0217 | 0 | 0.1781 | 0.1084 | 0.1138 | 0.0725 | 0.107 | 0 | 0 | 0 | 0.1077 | 0 | 0 | 0 | 0 | 0 | 0 | 0 |
| normal_tissue | German_2023 | 0.2391 | 0 | 0.0016 | 0 | 0.0203 | 0.0336 | 0.1783 | 0.0997 | 0.0146 | 0 | 0 | 0 | 0 | 0 | 0 | 0.0004 | 0 | 0 | 0 | 0 |
| normal_tissue | German_2023 | 0.6227 | 0.6847 | 0.0007 | 0 | 0 | 0.0007 | 0 | 0 | 0 | 0 | 0 | 0 | 0 | 0 | 0 | 0 | 0 | 0 | 0 | 0 |
| normal_tissue | German_2023 | 0.0976 | 0 | 48.3918 | 0 | 0.0253 | 0.007 | 0.0591 | 0.0485 | 0 | 0.0257 | 0 | 0 | 0 | 0 | 0 | 0 | 0 | 0 | 0 | 0 |
| normal_tissue | German_2023 | 0.0609 | 0.1276 | 0.0076 | 0 | 0.0565 | 0.0482 | 0.2641 | 0.1917 | 0.1466 | 0 | 0 | 0 | 0 | 0 | 0 | 0 | 0 | 0 | 0 | 0 |
| normal_tissue | German_2023 | 0.1518 | 0.0003 | 0.0035 | 0 | 0.0911 | 0.0024 | 0.2824 | 0.1099 | 0.1164 | 0 | 0 | 0 | 0 | 0 | 0 | 0 | 0 | 0 | 0 | 0 |
| normal_tissue | German_2023 | 42.7077 | 0.3121 | 0.2853 | 0 | 0.0027 | 0.004 | 0 | 0 | 0.0013 | 0 | 0 | 0 | 0.0027 | 0 | 0 | 0 | 0 | 0 | 0 | 0 |
| normal_tissue | German_2023 | 0.1542 | 0 | 0.0098 | 0 | 0 | 0.0422 | 0.1434 | 0.0996 | 0.1393 | 0 | 0 | 0 | 0 | 0 | 0 | 0 | 0 | 0 | 0 | 0 |
| normal_tissue | German_2023 | 0.1438 | 0.0361 | 0.0491 | 0 | 0.0531 | 0.0729 | 0.0869 | 0.2364 | 0.1284 | 0 | 0 | 0 | 0 | 0 | 0 | 0 | 0 | 0 | 0 | 0 |
| normal_tissue | German_2023 | 0.2675 | 0.0005 | 0.1107 | 0.0005 | 0.1673 | 0.0299 | 0.0451 | 0.5917 | 0.0588 | 0 | 0 | 0 | 0 | 0 | 0 | 0 | 0 | 0 | 0 | 0 |
| normal_tissue | German_2023 | 0.9037 | 0.1884 | 0.2074 | 0 | 0.0922 | 0.2575 | 0.6001 | 0.3757 | 0.3296 | 0 | 0 | 0 | 0 | 0 | 0 | 0 | 0 | 0 | 0 | 0 |
| normal_tissue | German_2023 | 1.0244 | 0.4413 | 0.0023 | 0 | 0.2997 | 0.1324 | 0 | 0.0023 | 0 | 0 | 0 | 0 | 0 | 0 | 0 | 0.0023 | 0 | 0 | 0 | 0 |
| normal_tissue | German_2023 | 1.4832 | 0.0004 | 0.2 | 0 | 0.0613 | 0.092 | 0.4236 | 0.077 | 0.1593 | 0 | 0 | 0 | 0 | 0 | 0 | 0 | 0 | 0 | 0 | 0 |
| normal_tissue | German_2023 | 0.2052 | 0.0449 | 0.0589 | 0 | 0 | 0.017 | 0.195 | 0.2039 | 0.1459 | 0 | 0 | 0 | 0 | 0 | 0 | 0 | 0 | 0 | 0 | 0 |
| normal_tissue | German_2023 | 2.9854 | 2.5194 | 0.0032 | 0 | 0 | 0 | 0 | 0 | 0 | 0 | 0 | 0 | 0 | 0 | 0 | 0 | 0 | 0 | 0 | 0 |
| normal_tissue | German_2023 | 0.7938 | 0.098 | 0 | 0 | 5.1406 | 4.9813 | 0.2148 | 0.3987 | 0.2833 | 0 | 0 | 0 | 0 | 0 | 0 | 0 | 0 | 0 | 0 | 0 |
| normal_tissue | German_2023 | 0.2775 | 0.0569 | 0.0951 | 0 | 0.0594 | 0.1611 | 0.0384 | 0.3305 | 0.2722 | 0 | 0 | 0 | 0 | 0 | 0 | 0 | 0 | 0 | 0 | 0 |
| normal_tissue | German_2023 | 0.2985 | 0.0115 | 0.1888 | 0.0003 | 0.0266 | 0.0278 | 0.068 | 0.1051 | 0.1363 | 0 | 0 | 0 | 0 | 0 | 0 | 0 | 0 | 0 | 0 | 0 |
| normal_tissue | German_2023 | 0.2499 | 0.0933 | 0.1032 | 0 | 0.0498 | 0.2186 | 0.2343 | 0.2179 | 0.0057 | 0 | 0 | 0.0662 | 0 | 0 | 0 | 0 | 0 | 0 | 0 | 0 |
| normal_tissue | German_2023 | 1.8164 | 0.0546 | 0.3125 | 0 | 0.0064 | 0.0807 | 1.0168 | 0.7399 | 0.4128 | 0 | 0 | 0 | 0 | 0 | 0 | 0 | 0 | 0 | 0 | 0 |
| normal_tissue | German_2023 | 3.3389 | 0.0138 | 0.0359 | 0.3282 | 0.1269 | 0.04 | 0.2179 | 0.0579 | 0.2634 | 0 | 0 | 0 | 0.051 | 0 | 0 | 0.2855 | 0 | 0 | 0 | 0 |
| normal_tissue | German_2023 | 0.2504 | 0.0402 | 0.0034 | 0 | 0.1803 | 0.1192 | 0.2098 | 0.0735 | 0.1034 | 0 | 0 | 0 | 0 | 0 | 0 | 0 | 0 | 0 | 0 | 0 |
| normal_tissue | German_2023 | 0.0587 | 0 | 0 | 0 | 4.3152 | 0.8252 | 0.0852 | 0.3752 | 0.4261 | 0 | 0 | 0 | 0 | 0 | 0 | 0 | 0 | 0 | 0 | 0 |
| normal_tissue | German_2023 | 0.5785 | 0.3394 | 0 | 0 | 0.0137 | 0.9896 | 1.7765 | 3.5997 | 0.9543 | 0 | 0 | 0 | 0 | 0 | 0 | 0 | 0 | 0 | 0 | 0 |
| BC_tissue | German_2023 | 2.326 | 0.1641 | 5.7554 | 0 | 0 | 0.0007 | 2.9377 | 2.3303 | 3.5783 | 0 | 0 | 0 | 0 | 0 | 0.0014 | 0 | 0 | 0 | 1.2812 | 0 |
| BC_tissue | German_2023 | 0.8113 | 0.5311 | 0.3042 | 0 | 0 | 0.0053 | 1.0995 | 2.287 | 1.0381 | 0.6512 | 0 | 0 | 0 | 0 | 0 | 0.8646 | 0 | 0 | 0 | 0 |
| BC_tissue | German_2023 | 0.0202 | 0.5146 | 0.0101 | 0 | 0 | 0 | 0.4238 | 0.4641 | 0.6155 | 0 | 0 | 0 | 0.3733 | 0 | 0 | 0 | 0 | 0 | 0.0101 | 0 |
| BC_tissue | German_2023 | 1.0872 | 0.1746 | 0.0087 | 0.0044 | 0 | 0.0131 | 0.3886 | 1.5806 | 0.9999 | 0 | 0 | 0 | 0 | 0 | 0 | 0 | 0 | 0 | 0.0087 | 0 |
| BC_tissue | German_2023 | 1.1485 | 0.0634 | 0.0104 | 0 | 0.0332 | 0.0758 | 0.2924 | 0.0953 | 0.1112 | 0 | 0 | 0 | 0 | 0 | 0 | 0 | 0.0003 | 0 | 0 | 0.0007 |
| BC_tissue | German_2023 | 1.2002 | 1.2736 | 0.0067 | 0 | 0 | 0 | 0.0233 | 0.6235 | 0.5868 | 0 | 0 | 0 | 0 | 0 | 0.0033 | 0.02 | 0 | 0 | 0 | 0 |
| BC_tissue | German_2023 | 0.083 | 0.1834 | 0.0546 | 0 | 0.048 | 0.0371 | 0.3777 | 0.2576 | 0.262 | 0 | 0 | 0 | 0 | 0 | 0.0677 | 0 | 0 | 0 | 0 | 0 |
| BC_tissue | German_2023 | 0.2365 | 0.0165 | 0.1158 | 0.0009 | 0.0067 | 0.077 | 0.2093 | 0.0553 | 0.0851 | 0 | 0 | 0 | 0.0003 | 0 | 0.0003 | 0 | 0 | 0 | 0 | 0 |
| BC_tissue | German_2023 | 0.842 | 0.0235 | 1.1196 | 0.1397 | 0 | 0 | 0.0502 | 0.0039 | 0.0431 | 0 | 0 | 0 | 0.0004 | 0 | 0 | 3.0535 | 0 | 0 | 0 | 0 |
| BC_tissue | German_2023 | 2.5176 | 0.189 | 0.115 | 0.3486 | 0 | 0.2192 | 0.3919 | 0.1966 | 0.4591 | 0 | 0 | 0 | 0.6756 | 0 | 0 | 0.2034 | 0 | 0 | 0 | 0 |
| BC_tissue | German_2023 | 0.1601 | 0 | 0.0024 | 0 | 0.0049 | 0.097 | 0.5483 | 0.0825 | 0.4416 | 0 | 0 | 0 | 0 | 0 | 0 | 0.0024 | 0 | 0 | 0 | 0 |
| BC_tissue | German_2023 | 0.745 | 4.2594 | 0.7966 | 0.0141 | 0 | 0 | 1.9446 | 0.6748 | 0.3514 | 0 | 0 | 0 | 0.5998 | 0 | 0 | 0 | 0 | 0 | 0 | 0 |
| BC_tissue | German_2023 | 1.076 | 1.5766 | 0.277 | 2.5993 | 0.5007 | 0 | 3.7286 | 0.8416 | 0.8949 | 0 | 0 | 0.0107 | 0.0107 | 0 | 0 | 1.8323 | 0.0107 | 0 | 0 | 0.0107 |
| BC_tissue | German_2023 | 0.262 | 0.1003 | 0.0488 | 0.0598 | 0 | 0.085 | 0.2593 | 0.0871 | 0.2089 | 0 | 0 | 0 | 0.001 | 0 | 0 | 0.116 | 0 | 0 | 0 | 0 |
| BC_tissue | German_2023 | 0.1683 | 0 | 0.0777 | 0 | 0 | 0 | 0.6862 | 0.8156 | 0.725 | 0 | 0 | 0 | 0 | 0 | 0 | 0.0129 | 0 | 0 | 0 | 0.0129 |
| BC_tissue | German_2023 | 0.5228 | 0.057 | 0.3612 | 0 | 0 | 0 | 6.1686 | 2.1956 | 2.6233 | 0 | 0 | 0 | 3.8019 | 0 | 0 | 0 | 0 | 0 | 0 | 0 |
| BC_tissue | German_2023 | 4.4708 | 0.0399 | 0.0685 | 2.2596 | 0.0628 | 0.3138 | 0.9073 | 1.8973 | 0.4765 | 0.0029 | 0 | 0 | 0 | 0 | 0 | 0 | 0 | 0 | 0 | 0.1854 |
| BC_tissue | German_2023 | 0.7556 | 0.536 | 0.0065 | 0.2841 | 0 | 0.0129 | 1.4918 | 1.1818 | 0.7491 | 0 | 0 | 0 | 0 | 0 | 0 | 0 | 0 | 0 | 0 | 0 |
| BC_tissue | German_2023 | 8.0762 | 0 | 0.709 | 0 | 0 | 4.3068 | 9.317 | 8.8296 | 3.2017 | 0 | 0 | 0 | 2.9358 | 0 | 0 | 0 | 0 | 0 | 0 | 0 |
| BC_tissue | German_2023 | 0.4407 | 0.3815 | 0.0971 | 0 | 0 | 0.0012 | 0.4004 | 0.0083 | 1.2961 | 0.0024 | 0 | 0 | 0 | 0 | 0 | 0 | 0 | 0 | 0 | 0 |
| BC_tissue | German_2023 | 2.3327 | 0 | 0.0022 | 0 | 0.0045 | 1.5768 | 12.0965 | 3.0572 | 0.9712 | 0 | 0 | 0 | 0 | 0 | 0.0022 | 0 | 0 | 0 | 0 | 0 |
| BC_tissue | German_2023 | 1.0178 | 42.4696 | 0.0291 | 0.0004 | 0 | 0.0004 | 0.5475 | 0.1956 | 0.1938 | 0 | 0 | 0 | 0 | 0 | 0 | 0 | 0 | 0 | 0.0004 | 0 |
| BC_tissue | German_2023 | 0.0654 | 55.2003 | 0.3536 | 0.0784 | 0 | 0.0019 | 0.4235 | 0.1158 | 0.2982 | 0 | 0 | 0 | 0 | 0 | 0 | 0.0004 | 0 | 0 | 0 | 0 |
| BC_tissue | German_2023 | 0.3725 | 0.0102 | 0.2037 | 0.062 | 0 | 0.0122 | 0.1762 | 0.5103 | 0.431 | 0 | 0 | 0 | 0 | 0 | 0.3629 | 0.2411 | 0 | 0 | 0 | 0 |
| BC_tissue | German_2023 | 0.6388 | 0.0261 | 0.269 | 0 | 0.0112 | 0.0897 | 0.6575 | 0.777 | 0.3848 | 0 | 0 | 0 | 0.2988 | 0 | 1.5353 | 0 | 0 | 0 | 0 | 0 |
| BC_tissue | German_2023 | 0.7209 | 0.3521 | 0.2012 | 0 | 0.0503 | 0.0335 | 4.0905 | 1.9614 | 3.6044 | 0 | 0 | 0 | 0 | 0 | 0.0168 | 0 | 0 | 0 | 0 | 0 |
| BC_tissue | German_2023 | 0.9711 | 0.6404 | 0.0897 | 0 | 0 | 0 | 0.7577 | 0.2653 | 0.2783 | 0.0003 | 0 | 0 | 0 | 0 | 0 | 0 | 0 | 0 | 0 | 0 |
| BC_tissue | German_2023 | 9.733 | 0.0044 | 0.0176 | 0.4803 | 0.1102 | 0.0969 | 0.141 | 0.7314 | 0.119 | 0 | 0 | 0 | 0.4979 | 0 | 0 | 0.0088 | 0 | 0 | 0 | 0 |
| BC_tissue | German_2023 | 0.7773 | 0.0591 | 0.1379 | 0.0039 | 0 | 0.067 | 0.1838 | 0.0407 | 0.1287 | 0 | 0 | 0 | 2.4645 | 0 | 0 | 0 | 0 | 0 | 0 | 0 |
| BC_tissue | German_2023 | 2.0973 | 0.4661 | 0.0037 | 0.0055 | 1.8936 | 2.712 | 1.6936 | 1.8918 | 0.4349 | 0 | 0 | 0 | 0 | 0 | 0 | 0.6239 | 0 | 0 | 0 | 0 |

**Table S7B. The RF score at the species level in both BC_tissue and normal_tissue samples.**

| **Group** | **Cohort** | **RF score** |
| --- | --- | --- |
| BC_tissue | Hoskinson_2022 | 0.03 |
| BC_tissue | Hoskinson_2022 | 0.06 |
| BC_tissue | Hoskinson_2022 | 0.11 |
| BC_tissue | Hoskinson_2022 | 0.19 |
| BC_tissue | Hoskinson_2022 | 0.16 |
| BC_tissue | Hoskinson_2022 | 0.32 |
| BC_tissue | Hoskinson_2022 | 0.3 |
| BC_tissue | Hoskinson_2022 | 0.34 |
| BC_tissue | Hoskinson_2022 | 0.07 |
| BC_tissue | Hoskinson_2022 | 0.19 |
| BC_tissue | Hoskinson_2022 | 0.28 |
| BC_tissue | Hoskinson_2022 | 0.11 |
| BC_tissue | Hoskinson_2022 | 0.05 |
| BC_tissue | Hoskinson_2022 | 0.07 |
| BC_tissue | Hoskinson_2022 | 0.2 |
| BC_tissue | Hoskinson_2022 | 0.25 |
| BC_tissue | Hoskinson_2022 | 0 |
| BC_tissue | Hoskinson_2022 | 0.03 |
| BC_tissue | Hoskinson_2022 | 0 |
| BC_tissue | Hoskinson_2022 | 0.16 |
| BC_tissue | Hoskinson_2022 | 0.19 |
| BC_tissue | Hoskinson_2022 | 0 |
| BC_tissue | Hoskinson_2022 | 0.01 |
| BC_tissue | Hoskinson_2022 | 0.02 |
| BC_tissue | Hoskinson_2022 | 0.05 |
| BC_tissue | Hoskinson_2022 | 0.04 |
| BC_tissue | Hoskinson_2022 | 0.03 |
| BC_tissue | Hoskinson_2022 | 0.07 |
| BC_tissue | Hoskinson_2022 | 0.02 |
| BC_tissue | Hoskinson_2022 | 0.1 |
| BC_tissue | Hoskinson_2022 | 0 |
| BC_tissue | Hoskinson_2022 | 0 |
| BC_tissue | Hoskinson_2022 | 0.05 |
| BC_tissue | Hoskinson_2022 | 0.09 |
| BC_tissue | Hoskinson_2022 | 0 |
| BC_tissue | Hoskinson_2022 | 0.18 |
| BC_tissue | Hoskinson_2022 | 0.16 |
| BC_tissue | Hoskinson_2022 | 0.1 |
| BC_tissue | Hoskinson_2022 | 0.02 |
| BC_tissue | Hoskinson_2022 | 0.25 |
| BC_tissue | Hoskinson_2022 | 0.24 |
| normal_tissue | Hoskinson_2022 | 0.62 |
| normal_tissue | Hoskinson_2022 | 0.69 |
| normal_tissue | Hoskinson_2022 | 0.7 |
| normal_tissue | Hoskinson_2022 | 0.84 |
| normal_tissue | Hoskinson_2022 | 0.98 |
| normal_tissue | Hoskinson_2022 | 1 |
| normal_tissue | Hoskinson_2022 | 0.98 |
| normal_tissue | Hoskinson_2022 | 0.95 |
| normal_tissue | Hoskinson_2022 | 1 |
| normal_tissue | Hoskinson_2022 | 0.93 |
| normal_tissue | Hoskinson_2022 | 0.82 |
| normal_tissue | Hoskinson_2022 | 0.85 |
| normal_tissue | Hoskinson_2022 | 0.72 |
| normal_tissue | Hoskinson_2022 | 0.9 |
| normal_tissue | Hoskinson_2022 | 0.97 |
| normal_tissue | Hoskinson_2022 | 0.91 |
| normal_tissue | Hoskinson_2022 | 0.99 |
| normal_tissue | Hoskinson_2022 | 0.95 |
| normal_tissue | Hoskinson_2022 | 1 |
| normal_tissue | Hoskinson_2022 | 1 |
| normal_tissue | Hoskinson_2022 | 0.91 |
| normal_tissue | Hoskinson_2022 | 0.98 |
| normal_tissue | Hoskinson_2022 | 1 |
| normal_tissue | Hoskinson_2022 | 0.96 |
| normal_tissue | Hoskinson_2022 | 0.78 |
| normal_tissue | Hoskinson_2022 | 0.94 |
| normal_tissue | Hoskinson_2022 | 0.88 |
| normal_tissue | Hoskinson_2022 | 1 |
| normal_tissue | Hoskinson_2022 | 0.77 |
| normal_tissue | Hoskinson_2022 | 0.96 |
| normal_tissue | Hoskinson_2022 | 1 |
| normal_tissue | Hoskinson_2022 | 0.84 |
| normal_tissue | Hoskinson_2022 | 1 |
| normal_tissue | Hoskinson_2022 | 0.91 |
| normal_tissue | Hoskinson_2022 | 0.88 |
| normal_tissue | Hoskinson_2022 | 0.94 |
| normal_tissue | Hoskinson_2022 | 1 |
| normal_tissue | Hoskinson_2022 | 0.96 |
| normal_tissue | Hoskinson_2022 | 1 |
| normal_tissue | Hoskinson_2022 | 1 |
| normal_tissue | Hoskinson_2022 | 1 |
| normal_tissue | Hoskinson_2022 | 1 |
| normal_tissue | Hoskinson_2022 | 0.89 |
| normal_tissue | Hoskinson_2022 | 1 |
| normal_tissue | Hoskinson_2022 | 0.99 |
| normal_tissue | Hoskinson_2022 | 0.99 |
| normal_tissue | Hoskinson_2022 | 1 |
| normal_tissue | Hoskinson_2022 | 0.96 |
| normal_tissue | Hoskinson_2022 | 0.99 |
| normal_tissue | German_2023 | 1 |
| normal_tissue | German_2023 | 1 |
| normal_tissue | German_2023 | 1 |
| normal_tissue | German_2023 | 0.98 |
| normal_tissue | German_2023 | 0.95 |
| normal_tissue | German_2023 | 1 |
| normal_tissue | German_2023 | 1 |
| normal_tissue | German_2023 | 1 |
| normal_tissue | German_2023 | 1 |
| normal_tissue | German_2023 | 1 |
| normal_tissue | German_2023 | 0.97 |
| normal_tissue | German_2023 | 1 |
| normal_tissue | German_2023 | 0.99 |
| normal_tissue | German_2023 | 0.91 |
| normal_tissue | German_2023 | 1 |
| normal_tissue | German_2023 | 1 |
| normal_tissue | German_2023 | 1 |
| normal_tissue | German_2023 | 1 |
| normal_tissue | German_2023 | 0.99 |
| normal_tissue | German_2023 | 1 |
| normal_tissue | German_2023 | 1 |
| normal_tissue | German_2023 | 1 |
| normal_tissue | German_2023 | 1 |
| normal_tissue | German_2023 | 0.94 |
| normal_tissue | German_2023 | 0.99 |
| normal_tissue | German_2023 | 1 |
| normal_tissue | German_2023 | 0.97 |
| normal_tissue | German_2023 | 1 |
| normal_tissue | German_2023 | 1 |
| normal_tissue | German_2023 | 1 |
| normal_tissue | German_2023 | 1 |
| normal_tissue | German_2023 | 0.99 |
| normal_tissue | German_2023 | 1 |
| normal_tissue | German_2023 | 1 |
| normal_tissue | German_2023 | 1 |
| normal_tissue | German_2023 | 0.96 |
| normal_tissue | German_2023 | 1 |
| normal_tissue | German_2023 | 1 |
| normal_tissue | German_2023 | 0.99 |
| normal_tissue | German_2023 | 0.99 |
| normal_tissue | German_2023 | 0.96 |
| normal_tissue | German_2023 | 0.99 |
| normal_tissue | German_2023 | 1 |
| normal_tissue | German_2023 | 0.99 |
| normal_tissue | German_2023 | 1 |
| normal_tissue | German_2023 | 1 |
| normal_tissue | German_2023 | 1 |
| normal_tissue | German_2023 | 1 |
| normal_tissue | German_2023 | 0.98 |
| normal_tissue | German_2023 | 1 |
| normal_tissue | German_2023 | 1 |
| normal_tissue | German_2023 | 0.97 |
| normal_tissue | German_2023 | 0.99 |
| normal_tissue | German_2023 | 0.99 |
| normal_tissue | German_2023 | 1 |
| normal_tissue | German_2023 | 0.96 |
| normal_tissue | German_2023 | 0.99 |
| normal_tissue | German_2023 | 1 |
| normal_tissue | German_2023 | 1 |
| normal_tissue | German_2023 | 0.99 |
| normal_tissue | German_2023 | 1 |
| normal_tissue | German_2023 | 0.99 |
| normal_tissue | German_2023 | 0.97 |
| normal_tissue | German_2023 | 0.99 |
| normal_tissue | German_2023 | 0.95 |
| normal_tissue | German_2023 | 0.97 |
| normal_tissue | German_2023 | 0.85 |
| normal_tissue | German_2023 | 0.91 |
| normal_tissue | German_2023 | 1 |
| normal_tissue | German_2023 | 0.98 |
| normal_tissue | German_2023 | 0.76 |
| normal_tissue | German_2023 | 0.97 |
| normal_tissue | German_2023 | 0.99 |
| normal_tissue | German_2023 | 1 |
| normal_tissue | German_2023 | 0.96 |
| normal_tissue | German_2023 | 1 |
| normal_tissue | German_2023 | 1 |
| normal_tissue | German_2023 | 0.99 |
| normal_tissue | German_2023 | 1 |
| normal_tissue | German_2023 | 0.91 |
| normal_tissue | German_2023 | 0.97 |
| normal_tissue | German_2023 | 0.98 |
| normal_tissue | German_2023 | 0.98 |
| normal_tissue | German_2023 | 0.99 |
| normal_tissue | German_2023 | 0.91 |
| normal_tissue | German_2023 | 0.99 |
| normal_tissue | German_2023 | 1 |
| normal_tissue | German_2023 | 0.88 |
| normal_tissue | German_2023 | 0.96 |
| normal_tissue | German_2023 | 0.99 |
| normal_tissue | German_2023 | 0.99 |
| normal_tissue | German_2023 | 0.97 |
| normal_tissue | German_2023 | 1 |
| normal_tissue | German_2023 | 1 |
| normal_tissue | German_2023 | 0.88 |
| normal_tissue | German_2023 | 1 |
| normal_tissue | German_2023 | 0.91 |
| normal_tissue | German_2023 | 0.98 |
| normal_tissue | German_2023 | 0.95 |
| normal_tissue | German_2023 | 0.97 |
| normal_tissue | German_2023 | 0.98 |
| normal_tissue | German_2023 | 0.99 |
| normal_tissue | German_2023 | 1 |
| normal_tissue | German_2023 | 0.93 |
| normal_tissue | German_2023 | 0.99 |
| normal_tissue | German_2023 | 0.98 |
| normal_tissue | German_2023 | 0.95 |
| normal_tissue | German_2023 | 0.98 |
| normal_tissue | German_2023 | 0.99 |
| normal_tissue | German_2023 | 1 |
| normal_tissue | German_2023 | 0.99 |
| normal_tissue | German_2023 | 1 |
| normal_tissue | German_2023 | 0.98 |
| normal_tissue | German_2023 | 1 |
| normal_tissue | German_2023 | 0.99 |
| normal_tissue | German_2023 | 0.94 |
| normal_tissue | German_2023 | 1 |
| normal_tissue | German_2023 | 0.97 |
| BC_tissue | Hoskinson_2022 | 0.12 |
| BC_tissue | Hoskinson_2022 | 0.01 |
| BC_tissue | Hoskinson_2022 | 0.14 |
| BC_tissue | Hoskinson_2022 | 0.06 |
| BC_tissue | Hoskinson_2022 | 0.08 |
| normal_tissue | German_2023 | 1 |
| normal_tissue | German_2023 | 0.97 |
| normal_tissue | German_2023 | 1 |
| normal_tissue | German_2023 | 0.97 |
| normal_tissue | German_2023 | 0.99 |
| normal_tissue | German_2023 | 1 |
| normal_tissue | German_2023 | 1 |
| normal_tissue | German_2023 | 1 |
| normal_tissue | German_2023 | 0.96 |
| normal_tissue | German_2023 | 1 |
| normal_tissue | German_2023 | 1 |
| normal_tissue | German_2023 | 1 |
| normal_tissue | German_2023 | 0.96 |
| normal_tissue | German_2023 | 0.99 |
| normal_tissue | German_2023 | 0.95 |
| normal_tissue | German_2023 | 0.98 |
| normal_tissue | German_2023 | 0.98 |
| normal_tissue | German_2023 | 1 |
| normal_tissue | German_2023 | 1 |
| normal_tissue | German_2023 | 0.95 |
| normal_tissue | German_2023 | 1 |
| normal_tissue | German_2023 | 1 |
| normal_tissue | German_2023 | 0.99 |
| normal_tissue | German_2023 | 0.96 |
| normal_tissue | German_2023 | 1 |
| normal_tissue | German_2023 | 1 |
| normal_tissue | German_2023 | 1 |
| normal_tissue | German_2023 | 0.98 |
| normal_tissue | German_2023 | 0.99 |
| normal_tissue | German_2023 | 0.99 |
| normal_tissue | German_2023 | 0.99 |
| normal_tissue | German_2023 | 1 |
| normal_tissue | German_2023 | 1 |
| normal_tissue | German_2023 | 0.97 |
| normal_tissue | German_2023 | 0.99 |
| normal_tissue | German_2023 | 1 |
| normal_tissue | German_2023 | 0.91 |
| normal_tissue | German_2023 | 1 |
| normal_tissue | German_2023 | 0.98 |
| normal_tissue | German_2023 | 0.99 |
| normal_tissue | German_2023 | 0.98 |
| normal_tissue | German_2023 | 1 |
| normal_tissue | German_2023 | 0.97 |
| normal_tissue | German_2023 | 1 |
| normal_tissue | German_2023 | 0.99 |
| normal_tissue | German_2023 | 1 |
| normal_tissue | German_2023 | 0.99 |
| normal_tissue | German_2023 | 0.99 |
| normal_tissue | German_2023 | 1 |
| normal_tissue | German_2023 | 0.99 |
| normal_tissue | German_2023 | 0.99 |
| normal_tissue | German_2023 | 0.99 |
| normal_tissue | German_2023 | 1 |
| normal_tissue | German_2023 | 1 |
| normal_tissue | German_2023 | 0.97 |
| normal_tissue | German_2023 | 1 |
| normal_tissue | German_2023 | 1 |
| normal_tissue | German_2023 | 1 |
| normal_tissue | German_2023 | 1 |
| normal_tissue | German_2023 | 0.99 |
| normal_tissue | German_2023 | 1 |
| normal_tissue | German_2023 | 0.99 |
| normal_tissue | German_2023 | 0.96 |
| normal_tissue | German_2023 | 1 |
| normal_tissue | German_2023 | 0.99 |
| normal_tissue | German_2023 | 0.98 |
| normal_tissue | German_2023 | 0.88 |
| normal_tissue | German_2023 | 0.99 |
| normal_tissue | German_2023 | 0.98 |
| normal_tissue | German_2023 | 0.99 |
| normal_tissue | German_2023 | 0.99 |
| normal_tissue | German_2023 | 1 |
| normal_tissue | German_2023 | 0.98 |
| normal_tissue | German_2023 | 0.95 |
| normal_tissue | German_2023 | 0.96 |
| normal_tissue | German_2023 | 1 |
| normal_tissue | German_2023 | 0.96 |
| normal_tissue | German_2023 | 0.99 |
| normal_tissue | German_2023 | 1 |
| normal_tissue | German_2023 | 0.99 |
| normal_tissue | German_2023 | 0.91 |
| normal_tissue | German_2023 | 0.89 |
| normal_tissue | German_2023 | 1 |
| normal_tissue | German_2023 | 0.99 |
| normal_tissue | German_2023 | 1 |
| normal_tissue | German_2023 | 1 |
| normal_tissue | German_2023 | 1 |
| normal_tissue | German_2023 | 1 |
| normal_tissue | German_2023 | 0.99 |
| normal_tissue | German_2023 | 0.99 |
| normal_tissue | German_2023 | 0.98 |
| normal_tissue | German_2023 | 0.88 |
| normal_tissue | German_2023 | 1 |
| normal_tissue | German_2023 | 0.99 |
| normal_tissue | German_2023 | 1 |
| normal_tissue | German_2023 | 1 |
| normal_tissue | German_2023 | 1 |
| normal_tissue | German_2023 | 0.95 |
| normal_tissue | German_2023 | 1 |
| normal_tissue | German_2023 | 1 |
| normal_tissue | German_2023 | 0.98 |
| normal_tissue | German_2023 | 1 |
| normal_tissue | German_2023 | 0.84 |
| normal_tissue | German_2023 | 1 |
| normal_tissue | German_2023 | 0.97 |
| normal_tissue | German_2023 | 0.98 |
| normal_tissue | German_2023 | 0.98 |
| normal_tissue | German_2023 | 1 |
| normal_tissue | German_2023 | 1 |
| normal_tissue | German_2023 | 1 |
| normal_tissue | German_2023 | 1 |
| normal_tissue | German_2023 | 1 |
| normal_tissue | German_2023 | 1 |
| normal_tissue | German_2023 | 1 |
| normal_tissue | German_2023 | 1 |
| normal_tissue | German_2023 | 1 |
| normal_tissue | German_2023 | 1 |
| normal_tissue | German_2023 | 1 |
| normal_tissue | German_2023 | 1 |
| normal_tissue | German_2023 | 1 |
| normal_tissue | German_2023 | 1 |
| normal_tissue | German_2023 | 1 |
| normal_tissue | German_2023 | 0.99 |
| normal_tissue | German_2023 | 0.96 |
| normal_tissue | German_2023 | 0.93 |
| normal_tissue | German_2023 | 1 |
| normal_tissue | German_2023 | 0.98 |
| normal_tissue | German_2023 | 1 |
| normal_tissue | German_2023 | 0.98 |
| normal_tissue | German_2023 | 1 |
| normal_tissue | German_2023 | 0.99 |
| normal_tissue | German_2023 | 0.96 |
| normal_tissue | German_2023 | 1 |
| normal_tissue | German_2023 | 0.96 |
| normal_tissue | German_2023 | 1 |
| normal_tissue | German_2023 | 0.97 |
| normal_tissue | German_2023 | 1 |
| normal_tissue | German_2023 | 0.96 |
| normal_tissue | German_2023 | 1 |
| normal_tissue | German_2023 | 0.97 |
| normal_tissue | German_2023 | 1 |
| normal_tissue | German_2023 | 1 |
| normal_tissue | German_2023 | 0.99 |
| normal_tissue | German_2023 | 0.98 |
| normal_tissue | German_2023 | 0.99 |
| normal_tissue | German_2023 | 0.99 |
| normal_tissue | German_2023 | 1 |
| normal_tissue | German_2023 | 0.97 |
| normal_tissue | German_2023 | 0.99 |
| normal_tissue | German_2023 | 1 |
| normal_tissue | German_2023 | 1 |
| normal_tissue | German_2023 | 0.98 |
| normal_tissue | German_2023 | 0.99 |
| normal_tissue | German_2023 | 0.95 |
| normal_tissue | German_2023 | 1 |
| normal_tissue | German_2023 | 0.98 |
| normal_tissue | German_2023 | 0.96 |
| normal_tissue | German_2023 | 1 |
| normal_tissue | German_2023 | 1 |
| normal_tissue | German_2023 | 0.96 |
| normal_tissue | German_2023 | 1 |
| normal_tissue | German_2023 | 1 |
| normal_tissue | German_2023 | 0.99 |
| normal_tissue | German_2023 | 1 |
| normal_tissue | German_2023 | 0.94 |
| normal_tissue | German_2023 | 0.99 |
| normal_tissue | German_2023 | 1 |
| normal_tissue | German_2023 | 1 |
| normal_tissue | German_2023 | 0.82 |
| normal_tissue | German_2023 | 0.9 |
| normal_tissue | German_2023 | 0.92 |
| normal_tissue | German_2023 | 0.79 |
| normal_tissue | German_2023 | 1 |
| normal_tissue | German_2023 | 0.99 |
| normal_tissue | German_2023 | 0.89 |
| normal_tissue | German_2023 | 1 |
| normal_tissue | German_2023 | 1 |
| normal_tissue | German_2023 | 0.95 |
| normal_tissue | German_2023 | 0.98 |
| normal_tissue | German_2023 | 1 |
| normal_tissue | German_2023 | 0.98 |
| normal_tissue | German_2023 | 0.96 |
| normal_tissue | German_2023 | 1 |
| normal_tissue | German_2023 | 0.99 |
| normal_tissue | German_2023 | 0.89 |
| normal_tissue | German_2023 | 0.99 |
| normal_tissue | German_2023 | 1 |
| normal_tissue | German_2023 | 0.78 |
| normal_tissue | German_2023 | 0.98 |
| normal_tissue | German_2023 | 1 |
| normal_tissue | German_2023 | 1 |
| normal_tissue | German_2023 | 1 |
| normal_tissue | German_2023 | 1 |
| normal_tissue | German_2023 | 0.99 |
| normal_tissue | German_2023 | 1 |
| normal_tissue | German_2023 | 1 |
| normal_tissue | German_2023 | 0.99 |
| normal_tissue | German_2023 | 1 |
| normal_tissue | German_2023 | 0.89 |
| normal_tissue | German_2023 | 0.98 |
| normal_tissue | German_2023 | 0.98 |
| normal_tissue | German_2023 | 1 |
| normal_tissue | German_2023 | 0.99 |
| normal_tissue | German_2023 | 1 |
| normal_tissue | German_2023 | 0.99 |
| normal_tissue | German_2023 | 0.98 |
| normal_tissue | German_2023 | 0.91 |
| normal_tissue | German_2023 | 1 |
| normal_tissue | German_2023 | 0.93 |
| normal_tissue | German_2023 | 0.99 |
| normal_tissue | German_2023 | 0.96 |
| normal_tissue | German_2023 | 0.88 |
| normal_tissue | German_2023 | 1 |
| normal_tissue | German_2023 | 1 |
| normal_tissue | German_2023 | 1 |
| normal_tissue | German_2023 | 0.96 |
| normal_tissue | German_2023 | 0.96 |
| normal_tissue | German_2023 | 0.94 |
| normal_tissue | German_2023 | 0.99 |
| normal_tissue | German_2023 | 0.99 |
| normal_tissue | German_2023 | 1 |
| normal_tissue | German_2023 | 1 |
| normal_tissue | German_2023 | 1 |
| normal_tissue | German_2023 | 1 |
| normal_tissue | German_2023 | 0.99 |
| normal_tissue | German_2023 | 0.99 |
| normal_tissue | German_2023 | 0.99 |
| normal_tissue | German_2023 | 1 |
| normal_tissue | German_2023 | 0.92 |
| normal_tissue | German_2023 | 1 |
| normal_tissue | German_2023 | 0.98 |
| normal_tissue | German_2023 | 1 |
| normal_tissue | German_2023 | 0.94 |
| normal_tissue | German_2023 | 1 |
| normal_tissue | German_2023 | 1 |
| normal_tissue | German_2023 | 0.97 |
| normal_tissue | German_2023 | 0.98 |
| normal_tissue | German_2023 | 0.95 |
| normal_tissue | German_2023 | 1 |
| normal_tissue | German_2023 | 0.93 |
| normal_tissue | German_2023 | 1 |
| normal_tissue | German_2023 | 1 |
| normal_tissue | German_2023 | 0.97 |
| normal_tissue | German_2023 | 0.9 |
| normal_tissue | German_2023 | 1 |
| normal_tissue | German_2023 | 0.97 |
| normal_tissue | German_2023 | 0.97 |
| normal_tissue | German_2023 | 1 |
| normal_tissue | German_2023 | 1 |
| normal_tissue | German_2023 | 1 |
| normal_tissue | German_2023 | 1 |
| normal_tissue | German_2023 | 1 |
| normal_tissue | German_2023 | 0.99 |
| normal_tissue | German_2023 | 0.88 |
| normal_tissue | German_2023 | 0.93 |
| normal_tissue | German_2023 | 1 |
| normal_tissue | German_2023 | 0.99 |
| normal_tissue | German_2023 | 0.94 |
| normal_tissue | German_2023 | 1 |
| normal_tissue | German_2023 | 1 |
| normal_tissue | German_2023 | 1 |
| normal_tissue | German_2023 | 0.99 |
| normal_tissue | German_2023 | 0.98 |
| normal_tissue | German_2023 | 0.99 |
| normal_tissue | German_2023 | 1 |
| normal_tissue | German_2023 | 1 |
| normal_tissue | German_2023 | 0.99 |
| normal_tissue | German_2023 | 1 |
| normal_tissue | German_2023 | 1 |
| normal_tissue | German_2023 | 1 |
| normal_tissue | German_2023 | 1 |
| normal_tissue | German_2023 | 0.99 |
| normal_tissue | German_2023 | 1 |
| normal_tissue | German_2023 | 1 |
| normal_tissue | German_2023 | 0.97 |
| normal_tissue | German_2023 | 1 |
| normal_tissue | German_2023 | 1 |
| normal_tissue | German_2023 | 0.99 |
| normal_tissue | German_2023 | 1 |
| normal_tissue | German_2023 | 0.99 |
| normal_tissue | German_2023 | 0.92 |
| normal_tissue | German_2023 | 0.99 |
| normal_tissue | German_2023 | 1 |
| normal_tissue | German_2023 | 1 |
| BC_tissue | German_2023 | 0.16 |
| BC_tissue | German_2023 | 0.33 |
| BC_tissue | German_2023 | 0.21 |
| BC_tissue | German_2023 | 0.26 |
| BC_tissue | German_2023 | 0.41 |
| BC_tissue | German_2023 | 0.31 |
| BC_tissue | German_2023 | 0.16 |
| BC_tissue | German_2023 | 0.4 |
| BC_tissue | German_2023 | 0.25 |
| BC_tissue | German_2023 | 0.23 |
| BC_tissue | German_2023 | 0.34 |
| BC_tissue | German_2023 | 0.26 |
| BC_tissue | German_2023 | 0.17 |
| BC_tissue | German_2023 | 0.29 |
| BC_tissue | German_2023 | 0.23 |
| BC_tissue | German_2023 | 0.28 |
| BC_tissue | German_2023 | 0.2 |
| BC_tissue | German_2023 | 0.35 |
| BC_tissue | German_2023 | 0.17 |
| BC_tissue | German_2023 | 0.27 |
| BC_tissue | German_2023 | 0.32 |
| BC_tissue | German_2023 | 0.29 |
| BC_tissue | German_2023 | 0.28 |
| BC_tissue | German_2023 | 0.13 |
| BC_tissue | German_2023 | 0.14 |
| BC_tissue | German_2023 | 0.25 |
| BC_tissue | German_2023 | 0.39 |
| BC_tissue | German_2023 | 0.34 |
| BC_tissue | German_2023 | 0.21 |
| BC_tissue | German_2023 | 0.38 |
