# Supplementary material for: Microbial diagnostic features identified across populations possess potential antitumor properties in breast cancer
Source: mSystems. 2025 Jun 23;10(7):e00271-25. doi: 10.1128/msystems.00271-25 (PMC12282184; doi:10.1128/msystems.00271-25)
Supplement: Table S8 — The abundance of Cutibacterium and C. acnes. [file msystems.00271-25-s0008.doc]

**Table S8A. The abundance of *Cutibacterium* in BC_tissue, BC_adjacent, and normal_tissue samples.**

| **BC_tissue** | **Abundance** | **BC_adjacent** | **Abundance** | **Normal_tissue** | **Abundance** |
| --- | --- | --- | --- | --- | --- |
| Hoskinson_2022 | 0.3511 | Hoskinson_2022 | 0.0055 | Hoskinson_2022 | 0.1 |
| Hoskinson_2022 | 0.0017 | Hoskinson_2022 | 0.026 | Hoskinson_2022 | 1.9958 |
| Hoskinson_2022 | 0.4019 | Hoskinson_2022 | 0.0677 | Hoskinson_2022 | 0.0092 |
| Hoskinson_2022 | 0.6603 | Hoskinson_2022 | 0.0195 | Hoskinson_2022 | 2.4097 |
| Hoskinson_2022 | 1.0333 | Hoskinson_2022 | 0.0043 | Hoskinson_2022 | 0 |
| Hoskinson_2022 | 1.2246 | Hoskinson_2022 | 2.3862 | Hoskinson_2022 | 0.5129 |
| Hoskinson_2022 | 2.2896 | Hoskinson_2022 | 0.5662 | Hoskinson_2022 | 0.9027 |
| Hoskinson_2022 | 0.6074 | Hoskinson_2022 | 0.5848 | Hoskinson_2022 | 1.7401 |
| Hoskinson_2022 | 0.3146 | Hoskinson_2022 | 6.4788 | Hoskinson_2022 | 0.0088 |
| Hoskinson_2022 | 0.4978 | Hoskinson_2022 | 0.4337 | Hoskinson_2022 | 0.3535 |
| Hoskinson_2022 | 3.1187 | Hoskinson_2022 | 1.2882 | Hoskinson_2022 | 1.8791 |
| Hoskinson_2022 | 4.0837 | Hoskinson_2022 | 0.4092 | Hoskinson_2022 | 1.2534 |
| Hoskinson_2022 | 0.0104 | Hoskinson_2022 | 1.1226 | Hoskinson_2022 | 1.3219 |
| Hoskinson_2022 | 0.0276 | Hoskinson_2022 | 1.1775 | Hoskinson_2022 | 0.1521 |
| Hoskinson_2022 | 1.134 | Hoskinson_2022 | 2.6222 | Hoskinson_2022 | 0.4593 |
| Hoskinson_2022 | 1.3141 | Hoskinson_2022 | 4.5148 | Hoskinson_2022 | 0.853 |
| Hoskinson_2022 | 0.0836 | Hoskinson_2022 | 0.0413 | Hoskinson_2022 | 0.0308 |
| Hoskinson_2022 | 0.0078 | Hoskinson_2022 | 0.0512 | Hoskinson_2022 | 0.1001 |
| Hoskinson_2022 | 0.0675 | Hoskinson_2022 | 0.0113 | Hoskinson_2022 | 0.0942 |
| Hoskinson_2022 | 0 | Hoskinson_2022 | 0.1595 | Hoskinson_2022 | 0.0541 |
| Hoskinson_2022 | 0 | Hoskinson_2022 | 0.2071 | Hoskinson_2022 | 1.2032 |
| Hoskinson_2022 | 0.0772 | Hoskinson_2022 | 0.0141 | Hoskinson_2022 | 0.274 |
| Hoskinson_2022 | 0.0685 | Hoskinson_2022 | 0.0108 | Hoskinson_2022 | 0.4893 |
| Hoskinson_2022 | 0.1121 | Hoskinson_2022 | 0.2912 | Hoskinson_2022 | 0.1554 |
| Hoskinson_2022 | 0.1576 | Hoskinson_2022 | 0 | Hoskinson_2022 | 0.8166 |
| Hoskinson_2022 | 0.03 | Hoskinson_2022 | 0.2074 | Hoskinson_2022 | 1.6994 |
| Hoskinson_2022 | 0.1623 | Hoskinson_2022 | 0.0038 | Hoskinson_2022 | 0.8235 |
| Hoskinson_2022 | 0.0749 | Hoskinson_2022 | 0.8382 | Hoskinson_2022 | 0.2347 |
| Hoskinson_2022 | 0.2343 | Hoskinson_2022 | 0.0721 | Hoskinson_2022 | 0.8384 |
| Hoskinson_2022 | 0.0161 | Hoskinson_2022 | 0.1887 | Hoskinson_2022 | 0.2418 |
| Hoskinson_2022 | 0.1587 | Hoskinson_2022 | 0.0229 | Hoskinson_2022 | 0.0883 |
| Hoskinson_2022 | 0.2317 | Hoskinson_2022 | 0.0676 | Hoskinson_2022 | 0 |
| Hoskinson_2022 | 0.0036 | Hoskinson_2022 | 0.0349 | Hoskinson_2022 | 0.2365 |
| Hoskinson_2022 | 0.0054 | Hoskinson_2022 | 0.0057 | Hoskinson_2022 | 0.4148 |
| Hoskinson_2022 | 0.2788 | Hoskinson_2022 | 0.0028 | Hoskinson_2022 | 0.7039 |
| Hoskinson_2022 | 0.1035 | Hoskinson_2022 | 0.2389 | Hoskinson_2022 | 0.0374 |
| Hoskinson_2022 | 0.0597 | Hoskinson_2022 | 0.1622 | Hoskinson_2022 | 0.1786 |
| Hoskinson_2022 | 0.0071 | Hoskinson_2022 | 0.0024 | Hoskinson_2022 | 0.1565 |
| Hoskinson_2022 | 0.1233 | Hoskinson_2022 | 0.0185 | Hoskinson_2022 | 0.4667 |
| Hoskinson_2022 | 0 | Hoskinson_2022 | 0.223 | Hoskinson_2022 | 0.1677 |
| Hoskinson_2022 | 1.2746 | Hoskinson_2022 | 0.0014 | Hoskinson_2022 | 0.0962 |
| Hoskinson_2022 | 2.0528 | Hoskinson_2022 | 0.1774 | Hoskinson_2022 | 0.6512 |
| Hoskinson_2022 | 0.0113 | Hoskinson_2022 | 0.1818 | Hoskinson_2022 | 0.0893 |
| Hoskinson_2022 | 0.7355 | Hoskinson_2022 | 0.0534 | Hoskinson_2022 | 0.2399 |
| Hoskinson_2022 | 0.0249 | Hoskinson_2022 | 0.568 | Hoskinson_2022 | 0 |
| Hoskinson_2022 | 0.2866 | Hoskinson_2022 | 1.1766 | Hoskinson_2022 | 0 |
| Esposito_2022 | 3.8565 | Hoskinson_2022 | 0 | Hoskinson_2022 | 0.451 |
| Esposito_2022 | 1.473 | Hoskinson_2022 | 0.0204 | Hoskinson_2022 | 0.8977 |
| Esposito_2022 | 10.9037 | Hoskinson_2022 | 0.0611 | Hoskinson_2022 | 0.8623 |
| Esposito_2022 | 4.5813 | Esposito_2022 | 0.9873 | German_2023 | 0.1964 |
| Esposito_2022 | 0.2703 | Esposito_2022 | 26.1543 | German_2023 | 0.5116 |
| Esposito_2022 | 3.9569 | Esposito_2022 | 23.6458 | German_2023 | 0.0769 |
| Esposito_2022 | 22.7523 | Esposito_2022 | 34.5429 | German_2023 | 0.055 |
| Esposito_2022 | 0.1323 | Esposito_2022 | 12.1007 | German_2023 | 0.2747 |
| Esposito_2022 | 4.3525 | Esposito_2022 | 3.0973 | German_2023 | 0.1449 |
| Esposito_2022 | 14.0533 | Esposito_2022 | 3.0561 | German_2023 | 0.3261 |
| Esposito_2022 | 3.7558 | Esposito_2022 | 22.716 | German_2023 | 0.1874 |
| Esposito_2022 | 1.5328 | Esposito_2022 | 15.2513 | German_2023 | 0.0809 |
| Esposito_2022 | 2.7168 | Esposito_2022 | 25.4764 | German_2023 | 0.2061 |
| Esposito_2022 | 4.7498 | Esposito_2022 | 29.8082 | German_2023 | 0.2657 |
| Esposito_2022 | 2.9489 | Esposito_2022 | 27.1185 | German_2023 | 0.0587 |
| Esposito_2022 | 5.1372 | Esposito_2022 | 78.2609 | German_2023 | 0.0505 |
| Esposito_2022 | 10.0913 | Esposito_2022 | 8.2096 | German_2023 | 1.3877 |
| Esposito_2022 | 5.3085 | Esposito_2022 | 21.3752 | German_2023 | 0.021 |
| Esposito_2022 | 3.2134 | Esposito_2022 | 18.0007 | German_2023 | 0.1549 |
| Esposito_2022 | 2.3641 | Esposito_2022 | 29.2122 | German_2023 | 0.1438 |
| Esposito_2022 | 13.6104 | Esposito_2022 | 12.6056 | German_2023 | 0.6363 |
| Esposito_2022 | 15.7227 | Esposito_2022 | 13.9512 | German_2023 | 0.19 |
| Esposito_2022 | 1.8501 | Esposito_2022 | 37.5925 | German_2023 | 0.2322 |
| Esposito_2022 | 12.8419 | Esposito_2022 | 33.6852 | German_2023 | 0.1093 |
| Esposito_2022 | 8.258 | Esposito_2022 | 41.9192 | German_2023 | 0.0054 |
| Esposito_2022 | 4.5332 | Esposito_2022 | 1.1832 | German_2023 | 0.141 |
| Esposito_2022 | 4.7662 | Esposito_2022 | 3.2217 | German_2023 | 0.0516 |
| Esposito_2022 | 1.9068 | Esposito_2022 | 5.392 | German_2023 | 0.1108 |
| Esposito_2022 | 4.0889 | Esposito_2022 | 30.2605 | German_2023 | 0.0627 |
| Esposito_2022 | 0.9899 | Esposito_2022 | 26.5387 | German_2023 | 1.4844 |
| Esposito_2022 | 5.5232 | Esposito_2022 | 18.5676 | German_2023 | 0.2583 |
| Esposito_2022 | 6.425 | Esposito_2022 | 15.1443 | German_2023 | 0.1896 |
| Esposito_2022 | 1.5557 | Esposito_2022 | 29.2578 | German_2023 | 0.246 |
| Esposito_2022 | 4.8356 | Esposito_2022 | 15.569 | German_2023 | 0.05 |
| Liu_2023 | 0 | Esposito_2022 | 60.2552 | German_2023 | 0.0812 |
| Liu_2023 | 0 | Esposito_2022 | 9.4057 | German_2023 | 0.3178 |
| Liu_2023 | 0 | Esposito_2022 | 33.2335 | German_2023 | 2.9634 |
| Liu_2023 | 0 | Kartti_2023 | 0.1992 | German_2023 | 0.1069 |
| Liu_2023 | 0 | Kartti_2023 | 0.0765 | German_2023 | 3.1982 |
| Liu_2023 | 0 | Kartti_2023 | 0.0552 | German_2023 | 5.3651 |
| Liu_2023 | 0 | Kartti_2023 | 0.0082 | German_2023 | 0.0071 |
| Liu_2023 | 0 | Kartti_2023 | 0.0224 | German_2023 | 3.9965 |
| Liu_2023 | 0 | Kartti_2023 | 0.2709 | German_2023 | 0.462 |
| Liu_2023 | 0 | Kartti_2023 | 0.0153 | German_2023 | 1.1683 |
| Liu_2023 | 0 | Kartti_2023 | 0.106 | German_2023 | 0.0277 |
| Liu_2023 | 0 | Kartti_2023 | 0.0039 | German_2023 | 0.1578 |
| Liu_2023 | 0 | Kartti_2023 | 0.1308 | German_2023 | 0.1197 |
| Liu_2023 | 0 | Kartti_2023 | 0.0651 | German_2023 | 0.1561 |
| Liu_2023 | 0 | Kartti_2023 | 0.4439 | German_2023 | 0.105 |
| Liu_2023 | 0 | Kartti_2023 | 0.3295 | German_2023 | 1.0276 |
| Liu_2023 | 0 | Kartti_2023 | 0.4215 | German_2023 | 0.4321 |
| Liu_2023 | 0 | Kartti_2023 | 0.0672 | German_2023 | 11.3543 |
| Liu_2023 | 0 | Kartti_2023 | 0 | German_2023 | 0.0059 |
| Liu_2023 | 0 | Kartti_2023 | 0.03 | German_2023 | 0.0696 |
| Liu_2023 | 0 | Kartti_2023 | 0.1811 | German_2023 | 6.00E-04 |
| Liu_2023 | 0 | Kartti_2023 | 0.2609 | German_2023 | 0.1594 |
| Liu_2023 | 0 | Kartti_2023 | 0.162 | German_2023 | 0.0153 |
| Liu_2023 | 0 | Kartti_2023 | 0.1426 | German_2023 | 0.611 |
| Liu_2023 | 0 | Kartti_2023 | 0.2238 | German_2023 | 0.1641 |
| Liu_2023 | 0 | Kartti_2023 | 0.4593 | German_2023 | 0.1305 |
| Liu_2023 | 0 | Kartti_2023 | 0.058 | German_2023 | 1.0335 |
| Liu_2023 | 0 | Kartti_2023 | 0.068 | German_2023 | 1.1389 |
| Liu_2023 | 0 | Kartti_2023 | 0.0644 | German_2023 | 0.0686 |
| Liu_2023 | 0 | Kartti_2023 | 2.0395 | German_2023 | 0.1746 |
| Liu_2023 | 0 | Kartti_2023 | 0.0017 | German_2023 | 0.6309 |
| Liu_2023 | 0 | Kartti_2023 | 0.0889 | German_2023 | 0.5416 |
| Liu_2023 | 0 | Kartti_2023 | 0.0197 | German_2023 | 0.6973 |
| Liu_2023 | 0 | Kartti_2023 | 0.1775 | German_2023 | 4.2248 |
| Liu_2023 | 0 | Kartti_2023 | 0 | German_2023 | 0.4468 |
| Liu_2023 | 0 | Kartti_2023 | 0.1444 | German_2023 | 1.2672 |
| Liu_2023 | 0 | Kartti_2023 | 0.0133 | German_2023 | 1.4705 |
| Liu_2023 | 0 | Kartti_2023 | 0.6993 | German_2023 | 0 |
| Liu_2023 | 0 | Kartti_2023 | 0.2651 | German_2023 | 16.0378 |
| Liu_2023 | 0 | Kartti_2023 | 0.1752 | German_2023 | 7.8809 |
| Liu_2023 | 0 | Kartti_2023 | 0 | German_2023 | 9.8395 |
| Liu_2023 | 0 | Kartti_2023 | 0.0034 | German_2023 | 1.4307 |
| Liu_2023 | 0 | Kartti_2023 | 0.5648 | German_2023 | 2.601 |
| Liu_2023 | 0 | Kartti_2023 | 0.5648 | German_2023 | 0.62 |
| Liu_2023 | 0 | Kartti_2023 | 0.0261 | German_2023 | 1.0456 |
| Liu_2023 | 0 | Kartti_2023 | 0.0406 | German_2023 | 0.6403 |
| Liu_2023 | 0 | Kartti_2023 | 0.5927 | German_2023 | 0.8414 |
| Liu_2023 | 0 | Kartti_2023 | 0.0032 | German_2023 | 0.4887 |
| Liu_2023 | 0 | Kartti_2023 | 0.0269 | German_2023 | 0.6078 |
| Liu_2023 | 0 | Kartti_2023 | 0.5954 | German_2023 | 0.2941 |
| Liu_2023 | 0 | Kartti_2023 | 0.1832 | German_2023 | 1.2682 |
| Liu_2023 | 0 | Kartti_2023 | 0.2232 | German_2023 | 3.6458 |
| Liu_2023 | 0 | Kartti_2023 | 0.5238 | German_2023 | 5.2069 |
| Liu_2023 | 0 | Kartti_2023 | 1.5595 | German_2023 | 13.2883 |
| Liu_2023 | 0 | Kartti_2023 | 0.0937 | German_2023 | 0.6253 |
| Liu_2023 | 0 | German_2023 | 2.6681 | German_2023 | 3.8525 |
| Liu_2023 | 0 | German_2023 | 0.0075 | German_2023 | 0.8032 |
| Liu_2023 | 0 | German_2023 | 8.8078 | German_2023 | 4.1976 |
| Liu_2023 | 0 | German_2023 | 0.3366 | German_2023 | 1.399 |
| Liu_2023 | 0 | German_2023 | 7.2479 | German_2023 | 4.1764 |
| Liu_2023 | 0 | German_2023 | 0.3888 | German_2023 | 1.0496 |
| Liu_2023 | 0 | German_2023 | 1.9674 | German_2023 | 0.0983 |
| Liu_2023 | 0 | German_2023 | 1.2607 | German_2023 | 1.2231 |
| Liu_2023 | 0 | German_2023 | 1.6939 | German_2023 | 3.13 |
| Liu_2023 | 0 | German_2023 | 0.116 | German_2023 | 0.1576 |
| Liu_2023 | 0 | German_2023 | 1.0282 | German_2023 | 2.0721 |
| Liu_2023 | 0 | German_2023 | 0.0926 | German_2023 | 0.1582 |
| Liu_2023 | 0 | German_2023 | 0.0731 | German_2023 | 2.8401 |
| Liu_2023 | 0 | German_2023 | 0.4437 | German_2023 | 0.0599 |
| Liu_2023 | 0 | German_2023 | 0.0021 | German_2023 | 1.7864 |
| Kartti_2023 | 0.0481 | German_2023 | 0.0058 | German_2023 | 0.8833 |
| Kartti_2023 | 0.1469 | German_2023 | 1.8163 | German_2023 | 0.2221 |
| Kartti_2023 | 0.103 | German_2023 | 0.5588 | German_2023 | 3.4635 |
| Kartti_2023 | 0.0396 | German_2023 | 0.8582 | German_2023 | 0.7421 |
| Kartti_2023 | 0.008 | German_2023 | 0 | German_2023 | 1.7 |
| Kartti_2023 | 0.4286 | German_2023 | 0.2101 | German_2023 | 1.2082 |
| Kartti_2023 | 0.0978 | German_2023 | 0.668 | German_2023 | 0.6536 |
| Kartti_2023 | 0.0344 | German_2023 | 0.3917 | German_2023 | 3.3723 |
| Kartti_2023 | 0.0114 | German_2023 | 0.0902 | German_2023 | 1.4173 |
| Kartti_2023 | 1.2696 | German_2023 | 6.8703 | German_2023 | 0.4753 |
| Kartti_2023 | 0.4674 | German_2023 | 6.9845 | German_2023 | 0.1187 |
| Kartti_2023 | 0.0699 | German_2023 | 1.4141 | German_2023 | 1.6032 |
| Kartti_2023 | 0.4053 | German_2023 | 0.929 | German_2023 | 0.941 |
| Kartti_2023 | 0.1706 | German_2023 | 0.6379 | German_2023 | 1.7991 |
| Kartti_2023 | 0.0489 | German_2023 | 0.7861 | German_2023 | 0.5248 |
| Kartti_2023 | 0.1353 | German_2023 | 0.0463 | German_2023 | 2.4423 |
| Kartti_2023 | 3.6618 | German_2023 | 0.124 | German_2023 | 5.167 |
| Kartti_2023 | 0.2607 | German_2023 | 0.6306 | German_2023 | 1.7835 |
| Kartti_2023 | 2.2865 | German_2023 | 3.2055 | German_2023 | 0.1036 |
| Kartti_2023 | 1.1064 | German_2023 | 0.5665 | German_2023 | 0.2117 |
| Kartti_2023 | 0 | German_2023 | 0.5397 | German_2023 | 3.1301 |
| Kartti_2023 | 0.7475 | German_2023 | 0.1989 | German_2023 | 0.2158 |
| Kartti_2023 | 0.3212 | German_2023 | 0.0767 | German_2023 | 0.2792 |
| Kartti_2023 | 0.3334 | German_2023 | 0.4893 | German_2023 | 0.148 |
| Kartti_2023 | 1.7694 | German_2023 | 2.3727 | German_2023 | 0.4583 |
| Kartti_2023 | 0.0071 | German_2023 | 0.7466 | German_2023 | 0.2161 |
| Kartti_2023 | 0.0092 | German_2023 | 1.4638 | German_2023 | 0.4258 |
| Kartti_2023 | 1.3805 | German_2023 | 0.2378 | German_2023 | 0.0134 |
| Kartti_2023 | 0.0714 | German_2023 | 0.1137 | German_2023 | 0.1091 |
| Kartti_2023 | 0.0594 | German_2023 | 2.7575 | German_2023 | 6.9987 |
| Kartti_2023 | 0.0475 | German_2023 | 2.0746 | German_2023 | 0.7199 |
| Kartti_2023 | 0.2294 | German_2023 | 3.7831 | German_2023 | 0.8656 |
| Kartti_2023 | 0.2103 | German_2023 | 3.3934 | German_2023 | 0.5787 |
| Kartti_2023 | 0.1595 | German_2023 | 0.3588 | German_2023 | 0.0694 |
| Kartti_2023 | 0.1433 | German_2023 | 0.068 | German_2023 | 0.0361 |
| Kartti_2023 | 0.0469 | German_2023 | 2.2394 | German_2023 | 0.1732 |
| Kartti_2023 | 0.149 | German_2023 | 0.1892 | German_2023 | 0.1127 |
| Kartti_2023 | 0.2393 | German_2023 | 1.6288 | German_2023 | 0.0231 |
| Kartti_2023 | 0.4304 | German_2023 | 0.0033 | German_2023 | 0.0037 |
| Kartti_2023 | 0.6166 | German_2023 | 0.0052 | German_2023 | 0.6444 |
| Kartti_2023 | 0.0712 | German_2023 | 2.2402 | German_2023 | 0.0359 |
| Kartti_2023 | 0.2343 | German_2023 | 0.0164 | German_2023 | 0.0024 |
| Kartti_2023 | 1.162 | German_2023 | 0.7157 | German_2023 | 0.3826 |
| Kartti_2023 | 0.0644 | German_2023 | 0.059 | German_2023 | 0.0075 |
| Kartti_2023 | 0 | German_2023 | 0.4487 | German_2023 | 3.348 |
| Kartti_2023 | 0.3687 |  |  | German_2023 | 0.0033 |
| Kartti_2023 | 0.6731 |  |  | German_2023 | 0.6077 |
| Kartti_2023 | 0.0103 |  |  | German_2023 | 0.2948 |
| Kartti_2023 | 0.1556 |  |  | German_2023 | 0.1152 |
| Kartti_2023 | 0.0955 |  |  | German_2023 | 0.1545 |
| Kartti_2023 | 0.4427 |  |  | German_2023 | 1.8869 |
| German_2023 | 2.5075 |  |  | German_2023 | 0.1793 |
| German_2023 | 0.8193 |  |  | German_2023 | 0.2125 |
| German_2023 | 0.0303 |  |  | German_2023 | 1.4127 |
| German_2023 | 1.0872 |  |  | German_2023 | 0.0425 |
| German_2023 | 1.1488 |  |  | German_2023 | 0.3659 |
| German_2023 | 1.2002 |  |  | German_2023 | 0.8029 |
| German_2023 | 0.083 |  |  | German_2023 | 4.4371 |
| German_2023 | 0.253 |  |  | German_2023 | 3.9571 |
| German_2023 | 0.8912 |  |  | German_2023 | 0.8009 |
| German_2023 | 2.6606 |  |  | German_2023 | 0.768 |
| German_2023 | 0.1601 |  |  | German_2023 | 1.4599 |
| German_2023 | 0.745 |  |  | German_2023 | 1.0706 |
| German_2023 | 1.5127 |  |  | German_2023 | 0.0071 |
| German_2023 | 0.262 |  |  | German_2023 | 0.2504 |
| German_2023 | 0.1683 |  |  | German_2023 | 0.3168 |
| German_2023 | 0.5228 |  |  | German_2023 | 2.6818 |
| German_2023 | 4.622 |  |  | German_2023 | 0.1196 |
| German_2023 | 0.762 |  |  | German_2023 | 0.0016 |
| German_2023 | 8.0762 |  |  | German_2023 | 0.0592 |
| German_2023 | 0.4407 |  |  | German_2023 | 0.0554 |
| German_2023 | 2.3327 |  |  | German_2023 | 0.8285 |
| German_2023 | 1.0178 |  |  | German_2023 | 0.2576 |
| German_2023 | 0.0654 |  |  | German_2023 | 0.5724 |
| German_2023 | 0.4093 |  |  | German_2023 | 0.1381 |
| German_2023 | 0.6388 |  |  | German_2023 | 0.1481 |
| German_2023 | 0.7209 |  |  | German_2023 | 0.1144 |
| German_2023 | 1.286 |  |  | German_2023 | 0.2664 |
| German_2023 | 10.1031 |  |  | German_2023 | 0.1904 |
| German_2023 | 0.8941 |  |  | German_2023 | 0.9685 |
| German_2023 | 2.0973 |  |  | German_2023 | 0.1435 |
|  |  |  |  | German_2023 | 1.7517 |
|  |  |  |  | German_2023 | 1.1895 |
|  |  |  |  | German_2023 | 1.0298 |
|  |  |  |  | German_2023 | 1.7592 |
|  |  |  |  | German_2023 | 2.159 |
|  |  |  |  | German_2023 | 5.2246 |
|  |  |  |  | German_2023 | 0.2488 |
|  |  |  |  | German_2023 | 0.5226 |
|  |  |  |  | German_2023 | 2.4996 |
|  |  |  |  | German_2023 | 27.5392 |
|  |  |  |  | German_2023 | 0.0254 |
|  |  |  |  | German_2023 | 0.0755 |
|  |  |  |  | German_2023 | 1.1453 |
|  |  |  |  | German_2023 | 0.3173 |
|  |  |  |  | German_2023 | 4.1091 |
|  |  |  |  | German_2023 | 0.0619 |
|  |  |  |  | German_2023 | 3.0929 |
|  |  |  |  | German_2023 | 0.1866 |
|  |  |  |  | German_2023 | 4.5714 |
|  |  |  |  | German_2023 | 0.1117 |
|  |  |  |  | German_2023 | 0.6275 |
|  |  |  |  | German_2023 | 0.2297 |
|  |  |  |  | German_2023 | 0.5083 |
|  |  |  |  | German_2023 | 0.4266 |
|  |  |  |  | German_2023 | 0.5089 |
|  |  |  |  | German_2023 | 2.1489 |
|  |  |  |  | German_2023 | 0.7191 |
|  |  |  |  | German_2023 | 0.4101 |
|  |  |  |  | German_2023 | 0.2069 |
|  |  |  |  | German_2023 | 15.2112 |
|  |  |  |  | German_2023 | 0.0439 |
|  |  |  |  | German_2023 | 1.188 |
|  |  |  |  | German_2023 | 0.6066 |
|  |  |  |  | German_2023 | 0.812 |
|  |  |  |  | German_2023 | 0.1952 |
|  |  |  |  | German_2023 | 2.0427 |
|  |  |  |  | German_2023 | 0.2393 |
|  |  |  |  | German_2023 | 0.1375 |
|  |  |  |  | German_2023 | 24.1956 |
|  |  |  |  | German_2023 | 4.9493 |
|  |  |  |  | German_2023 | 0.4506 |
|  |  |  |  | German_2023 | 2.4203 |
|  |  |  |  | German_2023 | 6.00E-04 |
|  |  |  |  | German_2023 | 4.6803 |
|  |  |  |  | German_2023 | 0.1283 |
|  |  |  |  | German_2023 | 0.0113 |
|  |  |  |  | German_2023 | 0.2002 |
|  |  |  |  | German_2023 | 1.1415 |
|  |  |  |  | German_2023 | 0.5265 |
|  |  |  |  | German_2023 | 0.2154 |
|  |  |  |  | German_2023 | 0.0555 |
|  |  |  |  | German_2023 | 0.1409 |
|  |  |  |  | German_2023 | 0.1528 |
|  |  |  |  | German_2023 | 5.00E-04 |
|  |  |  |  | German_2023 | 0.0938 |
|  |  |  |  | German_2023 | 0.7159 |
|  |  |  |  | German_2023 | 1.5455 |
|  |  |  |  | German_2023 | 0.3173 |
|  |  |  |  | German_2023 | 0.0384 |
|  |  |  |  | German_2023 | 0.1189 |
|  |  |  |  | German_2023 | 0.7123 |
|  |  |  |  | German_2023 | 0.0862 |
|  |  |  |  | German_2023 | 1.2763 |
|  |  |  |  | German_2023 | 0.7353 |
|  |  |  |  | German_2023 | 0.0692 |
|  |  |  |  | German_2023 | 0.2522 |
|  |  |  |  | German_2023 | 0.0973 |
|  |  |  |  | German_2023 | 0.3776 |
|  |  |  |  | German_2023 | 0.0717 |
|  |  |  |  | German_2023 | 3.3313 |
|  |  |  |  | German_2023 | 0.1431 |
|  |  |  |  | German_2023 | 0.3545 |
|  |  |  |  | German_2023 | 0.0667 |
|  |  |  |  | German_2023 | 2.3985 |
|  |  |  |  | German_2023 | 0.0664 |
|  |  |  |  | German_2023 | 0.4737 |
|  |  |  |  | German_2023 | 0.1434 |
|  |  |  |  | German_2023 | 0.047 |
|  |  |  |  | German_2023 | 12.8971 |
|  |  |  |  | German_2023 | 0.6245 |
|  |  |  |  | German_2023 | 0.0232 |
|  |  |  |  | German_2023 | 4.9889 |
|  |  |  |  | German_2023 | 1.4011 |
|  |  |  |  | German_2023 | 1.1712 |
|  |  |  |  | German_2023 | 0.1503 |
|  |  |  |  | German_2023 | 0.4628 |
|  |  |  |  | German_2023 | 0.1268 |
|  |  |  |  | German_2023 | 3.00E-04 |
|  |  |  |  | German_2023 | 0.0151 |
|  |  |  |  | German_2023 | 0.1663 |
|  |  |  |  | German_2023 | 0.1349 |
|  |  |  |  | German_2023 | 1.5488 |
|  |  |  |  | German_2023 | 4.6163 |
|  |  |  |  | German_2023 | 0.0502 |
|  |  |  |  | German_2023 | 0.8736 |
|  |  |  |  | German_2023 | 4.0327 |
|  |  |  |  | German_2023 | 0.244 |
|  |  |  |  | German_2023 | 1.5915 |
|  |  |  |  | German_2023 | 2.1633 |
|  |  |  |  | German_2023 | 0.0941 |
|  |  |  |  | German_2023 | 0.3619 |
|  |  |  |  | German_2023 | 6.462 |
|  |  |  |  | German_2023 | 0.1127 |
|  |  |  |  | German_2023 | 1.4797 |
|  |  |  |  | German_2023 | 0.6376 |
|  |  |  |  | German_2023 | 1.749 |
|  |  |  |  | German_2023 | 3.3907 |
|  |  |  |  | German_2023 | 5.3764 |
|  |  |  |  | German_2023 | 0.0691 |
|  |  |  |  | German_2023 | 2.3747 |
|  |  |  |  | German_2023 | 6.7645 |
|  |  |  |  | German_2023 | 0.0888 |
|  |  |  |  | German_2023 | 0.1421 |
|  |  |  |  | German_2023 | 3.1848 |
|  |  |  |  | German_2023 | 9.5544 |
|  |  |  |  | German_2023 | 2.0248 |
|  |  |  |  | German_2023 | 5.2359 |
|  |  |  |  | German_2023 | 0.1701 |
|  |  |  |  | German_2023 | 3.3029 |
|  |  |  |  | German_2023 | 0.0785 |
|  |  |  |  | German_2023 | 0.1432 |
|  |  |  |  | German_2023 | 0.1782 |
|  |  |  |  | German_2023 | 0.2757 |
|  |  |  |  | German_2023 | 12.523 |
|  |  |  |  | German_2023 | 0.4108 |
|  |  |  |  | German_2023 | 0.0526 |
|  |  |  |  | German_2023 | 0.0939 |
|  |  |  |  | German_2023 | 0.522 |
|  |  |  |  | German_2023 | 4.6425 |
|  |  |  |  | German_2023 | 0.1769 |
|  |  |  |  | German_2023 | 0.1147 |
|  |  |  |  | German_2023 | 0.199 |
|  |  |  |  | German_2023 | 0.1633 |
|  |  |  |  | German_2023 | 0.0452 |
|  |  |  |  | German_2023 | 2.5581 |
|  |  |  |  | German_2023 | 0.2793 |
|  |  |  |  | German_2023 | 0.1323 |
|  |  |  |  | German_2023 | 0.4308 |
|  |  |  |  | German_2023 | 1.312 |
|  |  |  |  | German_2023 | 0.2247 |
|  |  |  |  | German_2023 | 0.575 |
|  |  |  |  | German_2023 | 0.9863 |
|  |  |  |  | German_2023 | 0.9857 |
|  |  |  |  | German_2023 | 0.8208 |
|  |  |  |  | German_2023 | 3.0938 |
|  |  |  |  | German_2023 | 0.8988 |
|  |  |  |  | German_2023 | 10.8394 |
|  |  |  |  | German_2023 | 0.2547 |
|  |  |  |  | German_2023 | 2.2176 |
|  |  |  |  | German_2023 | 1.077 |
|  |  |  |  | German_2023 | 0.0485 |
|  |  |  |  | German_2023 | 0.2193 |
|  |  |  |  | German_2023 | 2.0956 |
|  |  |  |  | German_2023 | 1.5115 |
|  |  |  |  | German_2023 | 0.7268 |
|  |  |  |  | German_2023 | 0.2776 |
|  |  |  |  | German_2023 | 0.0222 |
|  |  |  |  | German_2023 | 0.2111 |
|  |  |  |  | German_2023 | 0.0028 |
|  |  |  |  | German_2023 | 0.0469 |
|  |  |  |  | German_2023 | 0.5237 |
|  |  |  |  | German_2023 | 0.1735 |
|  |  |  |  | German_2023 | 1.2999 |
|  |  |  |  | German_2023 | 0.0671 |
|  |  |  |  | German_2023 | 7.6772 |
|  |  |  |  | German_2023 | 0.9614 |
|  |  |  |  | German_2023 | 0.0582 |
|  |  |  |  | German_2023 | 0.2304 |
|  |  |  |  | German_2023 | 3.6801 |
|  |  |  |  | German_2023 | 0.0141 |
|  |  |  |  | German_2023 | 0.3235 |
|  |  |  |  | German_2023 | 10.281 |
|  |  |  |  | German_2023 | 0.0413 |
|  |  |  |  | German_2023 | 8.8291 |
|  |  |  |  | German_2023 | 0.0536 |
|  |  |  |  | German_2023 | 0.1886 |
|  |  |  |  | German_2023 | 0.0636 |
|  |  |  |  | German_2023 | 0.1065 |
|  |  |  |  | German_2023 | 0.3021 |
|  |  |  |  | German_2023 | 32.7876 |
|  |  |  |  | German_2023 | 2.2162 |
|  |  |  |  | German_2023 | 0.4075 |
|  |  |  |  | German_2023 | 52.2399 |
|  |  |  |  | German_2023 | 1.8506 |
|  |  |  |  | German_2023 | 0.2765 |
|  |  |  |  | German_2023 | 0.0072 |
|  |  |  |  | German_2023 | 0.3087 |
|  |  |  |  | German_2023 | 0.0011 |
|  |  |  |  | German_2023 | 3.4667 |
|  |  |  |  | German_2023 | 0.59 |
|  |  |  |  | German_2023 | 0.8773 |
|  |  |  |  | German_2023 | 0.0785 |
|  |  |  |  | German_2023 | 0.6821 |
|  |  |  |  | German_2023 | 0.9898 |
|  |  |  |  | German_2023 | 0.6398 |
|  |  |  |  | German_2023 | 0.0708 |
|  |  |  |  | German_2023 | 0.665 |
|  |  |  |  | German_2023 | 0.2918 |
|  |  |  |  | German_2023 | 0.6227 |
|  |  |  |  | German_2023 | 0.0987 |
|  |  |  |  | German_2023 | 0.0616 |
|  |  |  |  | German_2023 | 0.1521 |
|  |  |  |  | German_2023 | 42.7573 |
|  |  |  |  | German_2023 | 0.1545 |
|  |  |  |  | German_2023 | 0.1438 |
|  |  |  |  | German_2023 | 0.2675 |
|  |  |  |  | German_2023 | 0.9037 |
|  |  |  |  | German_2023 | 1.0244 |
|  |  |  |  | German_2023 | 1.6679 |
|  |  |  |  | German_2023 | 0.2239 |
|  |  |  |  | German_2023 | 2.9854 |
|  |  |  |  | German_2023 | 0.8265 |
|  |  |  |  | German_2023 | 0.2775 |
|  |  |  |  | German_2023 | 0.3106 |
|  |  |  |  | German_2023 | 0.3646 |
|  |  |  |  | German_2023 | 1.8164 |
|  |  |  |  | German_2023 | 3.4562 |
|  |  |  |  | German_2023 | 0.2504 |
|  |  |  |  | German_2023 | 0.0935 |
|  |  |  |  | German_2023 | 0.5785 |

**Table S8B. The abundance of *C. acnes* in BC_tissue, BC_adjacent, and normal_tissue samples.**

| **BC_tissue** | **Abundance** | **BC_adjacent** | **Abundance** | **Normal_tissue** | **Abundance** |
| --- | --- | --- | --- | --- | --- |
| Hoskinson_2022 | 0.3511 | Hoskinson_2022 | 0.0055 | Hoskinson_2022 | 0.1 |
| Hoskinson_2022 | 0.0017 | Hoskinson_2022 | 0.026 | Hoskinson_2022 | 1.9958 |
| Hoskinson_2022 | 0.4019 | Hoskinson_2022 | 0.0677 | Hoskinson_2022 | 0.0092 |
| Hoskinson_2022 | 0.5535 | Hoskinson_2022 | 0.0195 | Hoskinson_2022 | 2.4097 |
| Hoskinson_2022 | 0.9261 | Hoskinson_2022 | 0.0043 | Hoskinson_2022 | 0 |
| Hoskinson_2022 | 1.1357 | Hoskinson_2022 | 2.3862 | Hoskinson_2022 | 0.5008 |
| Hoskinson_2022 | 2.2896 | Hoskinson_2022 | 0.5662 | Hoskinson_2022 | 0.9027 |
| Hoskinson_2022 | 0.6074 | Hoskinson_2022 | 0.5848 | Hoskinson_2022 | 1.5978 |
| Hoskinson_2022 | 0.3146 | Hoskinson_2022 | 6.4775 | Hoskinson_2022 | 0.0088 |
| Hoskinson_2022 | 0.4978 | Hoskinson_2022 | 0.4038 | Hoskinson_2022 | 0.3535 |
| Hoskinson_2022 | 3.1187 | Hoskinson_2022 | 1.2222 | Hoskinson_2022 | 1.8791 |
| Hoskinson_2022 | 4.0837 | Hoskinson_2022 | 0.4092 | Hoskinson_2022 | 1.2534 |
| Hoskinson_2022 | 0.0017 | Hoskinson_2022 | 1.1226 | Hoskinson_2022 | 1.0506 |
| Hoskinson_2022 | 0.0276 | Hoskinson_2022 | 1.1775 | Hoskinson_2022 | 0.1521 |
| Hoskinson_2022 | 1.134 | Hoskinson_2022 | 2.6222 | Hoskinson_2022 | 0.4593 |
| Hoskinson_2022 | 1.3141 | Hoskinson_2022 | 4.5148 | Hoskinson_2022 | 0.7457 |
| Hoskinson_2022 | 0.0836 | Hoskinson_2022 | 0.0413 | Hoskinson_2022 | 0.0308 |
| Hoskinson_2022 | 0.0078 | Hoskinson_2022 | 0.0512 | Hoskinson_2022 | 0.1001 |
| Hoskinson_2022 | 0.0675 | Hoskinson_2022 | 0.0113 | Hoskinson_2022 | 0.0942 |
| Hoskinson_2022 | 0 | Hoskinson_2022 | 0.1595 | Hoskinson_2022 | 0.0541 |
| Hoskinson_2022 | 0 | Hoskinson_2022 | 0.2071 | Hoskinson_2022 | 1.2032 |
| Hoskinson_2022 | 0.0553 | Hoskinson_2022 | 0.0141 | Hoskinson_2022 | 0.274 |
| Hoskinson_2022 | 0.0685 | Hoskinson_2022 | 0.0108 | Hoskinson_2022 | 0.4893 |
| Hoskinson_2022 | 0.1121 | Hoskinson_2022 | 0.2912 | Hoskinson_2022 | 0.1554 |
| Hoskinson_2022 | 0.1576 | Hoskinson_2022 | 0 | Hoskinson_2022 | 0.8166 |
| Hoskinson_2022 | 0.03 | Hoskinson_2022 | 0.2074 | Hoskinson_2022 | 1.6994 |
| Hoskinson_2022 | 0.1623 | Hoskinson_2022 | 0.0038 | Hoskinson_2022 | 0.8235 |
| Hoskinson_2022 | 0.0749 | Hoskinson_2022 | 0.8382 | Hoskinson_2022 | 0.2347 |
| Hoskinson_2022 | 0.2343 | Hoskinson_2022 | 0.0721 | Hoskinson_2022 | 0.8384 |
| Hoskinson_2022 | 0.0066 | Hoskinson_2022 | 0.1887 | Hoskinson_2022 | 0.2418 |
| Hoskinson_2022 | 0.1587 | Hoskinson_2022 | 0.0229 | Hoskinson_2022 | 0.0883 |
| Hoskinson_2022 | 0.2317 | Hoskinson_2022 | 0.0676 | Hoskinson_2022 | 0 |
| Hoskinson_2022 | 0.0036 | Hoskinson_2022 | 0.0349 | Hoskinson_2022 | 0.2365 |
| Hoskinson_2022 | 0.0054 | Hoskinson_2022 | 0.0057 | Hoskinson_2022 | 0.4002 |
| Hoskinson_2022 | 0.2788 | Hoskinson_2022 | 0.0028 | Hoskinson_2022 | 0.7039 |
| Hoskinson_2022 | 0.1035 | Hoskinson_2022 | 0.2264 | Hoskinson_2022 | 0.0374 |
| Hoskinson_2022 | 0.0597 | Hoskinson_2022 | 0.1622 | Hoskinson_2022 | 0.1786 |
| Hoskinson_2022 | 0.0071 | Hoskinson_2022 | 0.0024 | Hoskinson_2022 | 0.1565 |
| Hoskinson_2022 | 0.1233 | Hoskinson_2022 | 0.0185 | Hoskinson_2022 | 0.4667 |
| Hoskinson_2022 | 0 | Hoskinson_2022 | 0.223 | Hoskinson_2022 | 0.1569 |
| Hoskinson_2022 | 1.2373 | Hoskinson_2022 | 0.0014 | Hoskinson_2022 | 0.0962 |
| Hoskinson_2022 | 1.9872 | Hoskinson_2022 | 0.1774 | Hoskinson_2022 | 0.6512 |
| Hoskinson_2022 | 0.0113 | Hoskinson_2022 | 0.1818 | Hoskinson_2022 | 0.0893 |
| Hoskinson_2022 | 0.7355 | Hoskinson_2022 | 0.0534 | Hoskinson_2022 | 0.2399 |
| Hoskinson_2022 | 0.0249 | Hoskinson_2022 | 0.568 | Hoskinson_2022 | 0 |
| Hoskinson_2022 | 0.2844 | Hoskinson_2022 | 1.1588 | Hoskinson_2022 | 0 |
| Esposito_2022 | 3.8565 | Hoskinson_2022 | 0 | Hoskinson_2022 | 0.451 |
| Esposito_2022 | 0.9329 | Hoskinson_2022 | 0.0204 | Hoskinson_2022 | 0.8977 |
| Esposito_2022 | 10.9037 | Hoskinson_2022 | 0.0611 | Hoskinson_2022 | 0.8623 |
| Esposito_2022 | 4.5661 | Esposito_2022 | 0.9873 | German_2023 | 0.1803 |
| Esposito_2022 | 0.2574 | Esposito_2022 | 24.0902 | German_2023 | 0.5116 |
| Esposito_2022 | 3.9039 | Esposito_2022 | 23.6458 | German_2023 | 0.0769 |
| Esposito_2022 | 22.7523 | Esposito_2022 | 33.0132 | German_2023 | 0.055 |
| Esposito_2022 | 0.1323 | Esposito_2022 | 12.1007 | German_2023 | 0.2708 |
| Esposito_2022 | 4.3525 | Esposito_2022 | 2.5418 | German_2023 | 0.1291 |
| Esposito_2022 | 14.0533 | Esposito_2022 | 3.0561 | German_2023 | 0.2417 |
| Esposito_2022 | 3.7558 | Esposito_2022 | 22.3793 | German_2023 | 0.1398 |
| Esposito_2022 | 1.5328 | Esposito_2022 | 15.208 | German_2023 | 7.00E-04 |
| Esposito_2022 | 2.4819 | Esposito_2022 | 24.6239 | German_2023 | 0.1072 |
| Esposito_2022 | 4.7498 | Esposito_2022 | 28.9863 | German_2023 | 0.259 |
| Esposito_2022 | 2.9489 | Esposito_2022 | 27.0101 | German_2023 | 0.0587 |
| Esposito_2022 | 5.1372 | Esposito_2022 | 78.2609 | German_2023 | 0.0433 |
| Esposito_2022 | 10.0744 | Esposito_2022 | 8.185 | German_2023 | 1.2673 |
| Esposito_2022 | 5.2516 | Esposito_2022 | 20.8149 | German_2023 | 0.021 |
| Esposito_2022 | 3.2134 | Esposito_2022 | 17.6572 | German_2023 | 0.147 |
| Esposito_2022 | 2.3641 | Esposito_2022 | 28.6436 | German_2023 | 0.1398 |
| Esposito_2022 | 13.5812 | Esposito_2022 | 12.5957 | German_2023 | 0.6094 |
| Esposito_2022 | 15.41 | Esposito_2022 | 13.6911 | German_2023 | 0.1868 |
| Esposito_2022 | 1.8501 | Esposito_2022 | 37.4868 | German_2023 | 0.1998 |
| Esposito_2022 | 12.8221 | Esposito_2022 | 33.0614 | German_2023 | 0.0994 |
| Esposito_2022 | 8.2345 | Esposito_2022 | 41.9192 | German_2023 | 0.0018 |
| Esposito_2022 | 4.1349 | Esposito_2022 | 1.1715 | German_2023 | 0.141 |
| Esposito_2022 | 4.7662 | Esposito_2022 | 3.2217 | German_2023 | 0.0516 |
| Esposito_2022 | 1.9068 | Esposito_2022 | 5.3799 | German_2023 | 0.1013 |
| Esposito_2022 | 3.9313 | Esposito_2022 | 30.2271 | German_2023 | 0.0452 |
| Esposito_2022 | 0.9899 | Esposito_2022 | 25.8769 | German_2023 | 1.4844 |
| Esposito_2022 | 5.5166 | Esposito_2022 | 18.5676 | German_2023 | 0.2259 |
| Esposito_2022 | 6.3894 | Esposito_2022 | 14.4527 | German_2023 | 0.1896 |
| Esposito_2022 | 1.5486 | Esposito_2022 | 29.0785 | German_2023 | 0.2418 |
| Esposito_2022 | 4.8301 | Esposito_2022 | 15.1643 | German_2023 | 0.0347 |
| Liu_2023 | 0 | Esposito_2022 | 60.1816 | German_2023 | 0.0497 |
| Liu_2023 | 0 | Esposito_2022 | 7.3434 | German_2023 | 0.3178 |
| Liu_2023 | 0 | Esposito_2022 | 31.5654 | German_2023 | 2.8212 |
| Liu_2023 | 0 | Kartti_2023 | 0.1992 | German_2023 | 0.1068 |
| Liu_2023 | 0 | Kartti_2023 | 0.0765 | German_2023 | 3.1982 |
| Liu_2023 | 0 | Kartti_2023 | 0.0552 | German_2023 | 5.3624 |
| Liu_2023 | 0 | Kartti_2023 | 0.0082 | German_2023 | 0.0071 |
| Liu_2023 | 0 | Kartti_2023 | 0.0224 | German_2023 | 3.8446 |
| Liu_2023 | 0 | Kartti_2023 | 0.1827 | German_2023 | 0.462 |
| Liu_2023 | 0 | Kartti_2023 | 0.0153 | German_2023 | 1.1683 |
| Liu_2023 | 0 | Kartti_2023 | 0.106 | German_2023 | 0.0143 |
| Liu_2023 | 0 | Kartti_2023 | 0.0039 | German_2023 | 0.0968 |
| Liu_2023 | 0 | Kartti_2023 | 0.0992 | German_2023 | 0.1193 |
| Liu_2023 | 0 | Kartti_2023 | 0.0651 | German_2023 | 0.1561 |
| Liu_2023 | 0 | Kartti_2023 | 0.4439 | German_2023 | 0.105 |
| Liu_2023 | 0 | Kartti_2023 | 0.3295 | German_2023 | 1.0276 |
| Liu_2023 | 0 | Kartti_2023 | 0.4215 | German_2023 | 0.4321 |
| Liu_2023 | 0 | Kartti_2023 | 0.0672 | German_2023 | 11.3365 |
| Liu_2023 | 0 | Kartti_2023 | 0 | German_2023 | 0.0059 |
| Liu_2023 | 0 | Kartti_2023 | 0.03 | German_2023 | 0.0348 |
| Liu_2023 | 0 | Kartti_2023 | 0.1811 | German_2023 | 6.00E-04 |
| Liu_2023 | 0 | Kartti_2023 | 0.2609 | German_2023 | 0.1591 |
| Liu_2023 | 0 | Kartti_2023 | 0.162 | German_2023 | 0.0143 |
| Liu_2023 | 0 | Kartti_2023 | 0.1426 | German_2023 | 0.611 |
| Liu_2023 | 0 | Kartti_2023 | 0.2238 | German_2023 | 0.1641 |
| Liu_2023 | 0 | Kartti_2023 | 0.4593 | German_2023 | 0.1289 |
| Liu_2023 | 0 | Kartti_2023 | 0.058 | German_2023 | 1.0335 |
| Liu_2023 | 0 | Kartti_2023 | 0.068 | German_2023 | 1.1389 |
| Liu_2023 | 0 | Kartti_2023 | 0.0644 | German_2023 | 0.068 |
| Liu_2023 | 0 | Kartti_2023 | 2.0395 | German_2023 | 0.112 |
| Liu_2023 | 0 | Kartti_2023 | 0.0017 | German_2023 | 0.6309 |
| Liu_2023 | 0 | Kartti_2023 | 0.0842 | German_2023 | 0.5416 |
| Liu_2023 | 0 | Kartti_2023 | 0.0197 | German_2023 | 0.6973 |
| Liu_2023 | 0 | Kartti_2023 | 0.1775 | German_2023 | 4.2199 |
| Liu_2023 | 0 | Kartti_2023 | 0 | German_2023 | 0.4468 |
| Liu_2023 | 0 | Kartti_2023 | 0.0092 | German_2023 | 1.2555 |
| Liu_2023 | 0 | Kartti_2023 | 0.0133 | German_2023 | 1.4705 |
| Liu_2023 | 0 | Kartti_2023 | 0.6993 | German_2023 | 0 |
| Liu_2023 | 0 | Kartti_2023 | 0.2651 | German_2023 | 15.8163 |
| Liu_2023 | 0 | Kartti_2023 | 0.1752 | German_2023 | 7.3781 |
| Liu_2023 | 0 | Kartti_2023 | 0 | German_2023 | 9.7721 |
| Liu_2023 | 0 | Kartti_2023 | 0.0034 | German_2023 | 1.4192 |
| Liu_2023 | 0 | Kartti_2023 | 0.5648 | German_2023 | 2.601 |
| Liu_2023 | 0 | Kartti_2023 | 0.5648 | German_2023 | 0.62 |
| Liu_2023 | 0 | Kartti_2023 | 0.0261 | German_2023 | 1.0456 |
| Liu_2023 | 0 | Kartti_2023 | 0.0406 | German_2023 | 0.6147 |
| Liu_2023 | 0 | Kartti_2023 | 0.5927 | German_2023 | 0.8315 |
| Liu_2023 | 0 | Kartti_2023 | 0.0032 | German_2023 | 0.4884 |
| Liu_2023 | 0 | Kartti_2023 | 0.0269 | German_2023 | 0.6073 |
| Liu_2023 | 0 | Kartti_2023 | 0.5954 | German_2023 | 0.2936 |
| Liu_2023 | 0 | Kartti_2023 | 0.1832 | German_2023 | 1.2678 |
| Liu_2023 | 0 | Kartti_2023 | 0.2232 | German_2023 | 3.6455 |
| Liu_2023 | 0 | Kartti_2023 | 0.5238 | German_2023 | 5.1111 |
| Liu_2023 | 0 | Kartti_2023 | 1.5595 | German_2023 | 13.2878 |
| Liu_2023 | 0 | Kartti_2023 | 0.0937 | German_2023 | 0.6253 |
| Liu_2023 | 0 | German_2023 | 1.4866 | German_2023 | 3.4425 |
| Liu_2023 | 0 | German_2023 | 0.0075 | German_2023 | 0.8027 |
| Liu_2023 | 0 | German_2023 | 8.8063 | German_2023 | 3.877 |
| Liu_2023 | 0 | German_2023 | 0.3366 | German_2023 | 1.3152 |
| Liu_2023 | 0 | German_2023 | 7.2479 | German_2023 | 4.1714 |
| Liu_2023 | 0 | German_2023 | 0.3888 | German_2023 | 1.0168 |
| Liu_2023 | 0 | German_2023 | 1.9664 | German_2023 | 0.0983 |
| Liu_2023 | 0 | German_2023 | 1.2393 | German_2023 | 1.2231 |
| Liu_2023 | 0 | German_2023 | 1.6914 | German_2023 | 3.13 |
| Liu_2023 | 0 | German_2023 | 0.1129 | German_2023 | 0.1571 |
| Liu_2023 | 0 | German_2023 | 1.0252 | German_2023 | 2.0712 |
| Liu_2023 | 0 | German_2023 | 0.0841 | German_2023 | 0.1582 |
| Liu_2023 | 0 | German_2023 | 0.0731 | German_2023 | 2.7889 |
| Liu_2023 | 0 | German_2023 | 0.4006 | German_2023 | 0.0046 |
| Liu_2023 | 0 | German_2023 | 0.0021 | German_2023 | 1.7798 |
| Kartti_2023 | 0.0481 | German_2023 | 0.0058 | German_2023 | 0.8833 |
| Kartti_2023 | 0.1469 | German_2023 | 1.8154 | German_2023 | 0.0466 |
| Kartti_2023 | 0.103 | German_2023 | 0.5588 | German_2023 | 3.0347 |
| Kartti_2023 | 0.0396 | German_2023 | 0.793 | German_2023 | 0.6284 |
| Kartti_2023 | 0.008 | German_2023 | 0 | German_2023 | 1.6965 |
| Kartti_2023 | 0.4286 | German_2023 | 0.2035 | German_2023 | 1.2082 |
| Kartti_2023 | 0.0978 | German_2023 | 0.6671 | German_2023 | 0.5145 |
| Kartti_2023 | 0.0344 | German_2023 | 0.3917 | German_2023 | 3.3416 |
| Kartti_2023 | 0.0057 | German_2023 | 0.0902 | German_2023 | 1.4173 |
| Kartti_2023 | 1.2696 | German_2023 | 6.4257 | German_2023 | 0.4753 |
| Kartti_2023 | 0.4674 | German_2023 | 6.9833 | German_2023 | 0.1187 |
| Kartti_2023 | 0.0699 | German_2023 | 1.4141 | German_2023 | 1.6032 |
| Kartti_2023 | 0.4053 | German_2023 | 0.9264 | German_2023 | 0.941 |
| Kartti_2023 | 0.1706 | German_2023 | 0.6379 | German_2023 | 1.7991 |
| Kartti_2023 | 0.0204 | German_2023 | 0.7858 | German_2023 | 0.0236 |
| Kartti_2023 | 0.1353 | German_2023 | 0.0463 | German_2023 | 2.4423 |
| Kartti_2023 | 3.6618 | German_2023 | 0.124 | German_2023 | 5.167 |
| Kartti_2023 | 0.2607 | German_2023 | 0.5465 | German_2023 | 1.7835 |
| Kartti_2023 | 2.2865 | German_2023 | 3.162 | German_2023 | 0.0988 |
| Kartti_2023 | 1.0251 | German_2023 | 0.5203 | German_2023 | 0.2117 |
| Kartti_2023 | 0 | German_2023 | 0.5393 | German_2023 | 3.129 |
| Kartti_2023 | 0.7475 | German_2023 | 0.1989 | German_2023 | 0.1651 |
| Kartti_2023 | 0.3212 | German_2023 | 0.0764 | German_2023 | 0.2261 |
| Kartti_2023 | 0.3334 | German_2023 | 0.4893 | German_2023 | 0.0577 |
| Kartti_2023 | 1.6891 | German_2023 | 2.3613 | German_2023 | 0.4504 |
| Kartti_2023 | 0.0071 | German_2023 | 0.7466 | German_2023 | 0.2161 |
| Kartti_2023 | 0.0092 | German_2023 | 1.412 | German_2023 | 0.4258 |
| Kartti_2023 | 1.3805 | German_2023 | 0.144 | German_2023 | 6.00E-04 |
| Kartti_2023 | 0.0714 | German_2023 | 0.1122 | German_2023 | 0.1091 |
| Kartti_2023 | 0.0594 | German_2023 | 2.7565 | German_2023 | 6.9818 |
| Kartti_2023 | 0.0475 | German_2023 | 1.9324 | German_2023 | 0.3577 |
| Kartti_2023 | 0.2294 | German_2023 | 3.7831 | German_2023 | 0.7449 |
| Kartti_2023 | 0.2103 | German_2023 | 3.3934 | German_2023 | 0.5076 |
| Kartti_2023 | 0.1595 | German_2023 | 0.3588 | German_2023 | 0.0688 |
| Kartti_2023 | 0.1433 | German_2023 | 0.0676 | German_2023 | 0.0361 |
| Kartti_2023 | 0.0469 | German_2023 | 2.2394 | German_2023 | 0.1167 |
| Kartti_2023 | 0.149 | German_2023 | 0.1867 | German_2023 | 0.1127 |
| Kartti_2023 | 0.2393 | German_2023 | 1.6288 | German_2023 | 0.0231 |
| Kartti_2023 | 0.4304 | German_2023 | 0.0033 | German_2023 | 0.0037 |
| Kartti_2023 | 0.6166 | German_2023 | 0.0052 | German_2023 | 0.6417 |
| Kartti_2023 | 0.0712 | German_2023 | 2.1923 | German_2023 | 0.0359 |
| Kartti_2023 | 0.1804 | German_2023 | 0.0164 | German_2023 | 0.0024 |
| Kartti_2023 | 1.1033 | German_2023 | 0.7157 | German_2023 | 0.3826 |
| Kartti_2023 | 0.0644 | German_2023 | 0.059 | German_2023 | 0.0075 |
| Kartti_2023 | 0 | German_2023 | 0.4487 | German_2023 | 3.1076 |
| Kartti_2023 | 0.3687 |  |  | German_2023 | 0.0033 |
| Kartti_2023 | 0.6731 |  |  | German_2023 | 0.6077 |
| Kartti_2023 | 0.0086 |  |  | German_2023 | 0.2948 |
| Kartti_2023 | 0.1556 |  |  | German_2023 | 0.1152 |
| Kartti_2023 | 0.0955 |  |  | German_2023 | 0.1539 |
| Kartti_2023 | 0.4427 |  |  | German_2023 | 1.8861 |
| German_2023 | 2.326 |  |  | German_2023 | 0.1793 |
| German_2023 | 0.8113 |  |  | German_2023 | 0.2125 |
| German_2023 | 0.0202 |  |  | German_2023 | 1.4127 |
| German_2023 | 1.0872 |  |  | German_2023 | 0.039 |
| German_2023 | 1.1485 |  |  | German_2023 | 0.3659 |
| German_2023 | 1.2002 |  |  | German_2023 | 0.7936 |
| German_2023 | 0.083 |  |  | German_2023 | 4.4362 |
| German_2023 | 0.2365 |  |  | German_2023 | 3.9553 |
| German_2023 | 0.842 |  |  | German_2023 | 0.8009 |
| German_2023 | 2.5176 |  |  | German_2023 | 0.668 |
| German_2023 | 0.1601 |  |  | German_2023 | 1.4599 |
| German_2023 | 0.745 |  |  | German_2023 | 1.0706 |
| German_2023 | 1.076 |  |  | German_2023 | 0.0067 |
| German_2023 | 0.262 |  |  | German_2023 | 0.2504 |
| German_2023 | 0.1683 |  |  | German_2023 | 0.3168 |
| German_2023 | 0.5228 |  |  | German_2023 | 2.6751 |
| German_2023 | 4.4708 |  |  | German_2023 | 0.1196 |
| German_2023 | 0.7556 |  |  | German_2023 | 0.0016 |
| German_2023 | 8.0762 |  |  | German_2023 | 0.0307 |
| German_2023 | 0.4407 |  |  | German_2023 | 0.055 |
| German_2023 | 2.3327 |  |  | German_2023 | 0.8166 |
| German_2023 | 1.0178 |  |  | German_2023 | 0.2576 |
| German_2023 | 0.0654 |  |  | German_2023 | 0.5712 |
| German_2023 | 0.3725 |  |  | German_2023 | 0.1381 |
| German_2023 | 0.6388 |  |  | German_2023 | 0.1481 |
| German_2023 | 0.7209 |  |  | German_2023 | 0.0975 |
| German_2023 | 0.9711 |  |  | German_2023 | 0.2664 |
| German_2023 | 9.733 |  |  | German_2023 | 0.1898 |
| German_2023 | 0.7773 |  |  | German_2023 | 0.9685 |
| German_2023 | 2.0973 |  |  | German_2023 | 0.1435 |
|  |  |  |  | German_2023 | 1.7506 |
|  |  |  |  | German_2023 | 1.1439 |
|  |  |  |  | German_2023 | 0.7592 |
|  |  |  |  | German_2023 | 1.7592 |
|  |  |  |  | German_2023 | 2.159 |
|  |  |  |  | German_2023 | 4.8446 |
|  |  |  |  | German_2023 | 0.2488 |
|  |  |  |  | German_2023 | 0.5219 |
|  |  |  |  | German_2023 | 2.4996 |
|  |  |  |  | German_2023 | 27.5357 |
|  |  |  |  | German_2023 | 0.0254 |
|  |  |  |  | German_2023 | 0.0755 |
|  |  |  |  | German_2023 | 1.0575 |
|  |  |  |  | German_2023 | 0.3169 |
|  |  |  |  | German_2023 | 3.3176 |
|  |  |  |  | German_2023 | 0.0619 |
|  |  |  |  | German_2023 | 3.052 |
|  |  |  |  | German_2023 | 0.1858 |
|  |  |  |  | German_2023 | 3.8672 |
|  |  |  |  | German_2023 | 0.1117 |
|  |  |  |  | German_2023 | 0.6275 |
|  |  |  |  | German_2023 | 0.2244 |
|  |  |  |  | German_2023 | 0.4363 |
|  |  |  |  | German_2023 | 0.4266 |
|  |  |  |  | German_2023 | 0.4562 |
|  |  |  |  | German_2023 | 2.1466 |
|  |  |  |  | German_2023 | 0.6828 |
|  |  |  |  | German_2023 | 0.4085 |
|  |  |  |  | German_2023 | 0.2069 |
|  |  |  |  | German_2023 | 15.2112 |
|  |  |  |  | German_2023 | 0.0439 |
|  |  |  |  | German_2023 | 1.1873 |
|  |  |  |  | German_2023 | 0.3345 |
|  |  |  |  | German_2023 | 0.812 |
|  |  |  |  | German_2023 | 0.1952 |
|  |  |  |  | German_2023 | 2.0427 |
|  |  |  |  | German_2023 | 0.2393 |
|  |  |  |  | German_2023 | 0.1375 |
|  |  |  |  | German_2023 | 23.049 |
|  |  |  |  | German_2023 | 4.9481 |
|  |  |  |  | German_2023 | 0.4402 |
|  |  |  |  | German_2023 | 2.4203 |
|  |  |  |  | German_2023 | 6.00E-04 |
|  |  |  |  | German_2023 | 2.1682 |
|  |  |  |  | German_2023 | 0.0791 |
|  |  |  |  | German_2023 | 0.0113 |
|  |  |  |  | German_2023 | 0.1595 |
|  |  |  |  | German_2023 | 0.9586 |
|  |  |  |  | German_2023 | 0.5265 |
|  |  |  |  | German_2023 | 0.1945 |
|  |  |  |  | German_2023 | 0.0545 |
|  |  |  |  | German_2023 | 0.0874 |
|  |  |  |  | German_2023 | 0.0737 |
|  |  |  |  | German_2023 | 5.00E-04 |
|  |  |  |  | German_2023 | 0.0852 |
|  |  |  |  | German_2023 | 0.7159 |
|  |  |  |  | German_2023 | 1.4696 |
|  |  |  |  | German_2023 | 0.2929 |
|  |  |  |  | German_2023 | 0.034 |
|  |  |  |  | German_2023 | 0.0972 |
|  |  |  |  | German_2023 | 0.7123 |
|  |  |  |  | German_2023 | 0.0862 |
|  |  |  |  | German_2023 | 1.2619 |
|  |  |  |  | German_2023 | 0.7348 |
|  |  |  |  | German_2023 | 0.0688 |
|  |  |  |  | German_2023 | 0.2522 |
|  |  |  |  | German_2023 | 0.0808 |
|  |  |  |  | German_2023 | 0.3776 |
|  |  |  |  | German_2023 | 0.0715 |
|  |  |  |  | German_2023 | 3.2788 |
|  |  |  |  | German_2023 | 0.1066 |
|  |  |  |  | German_2023 | 0.3545 |
|  |  |  |  | German_2023 | 0.0667 |
|  |  |  |  | German_2023 | 1.8206 |
|  |  |  |  | German_2023 | 0.0664 |
|  |  |  |  | German_2023 | 0.4737 |
|  |  |  |  | German_2023 | 0.1434 |
|  |  |  |  | German_2023 | 0.0466 |
|  |  |  |  | German_2023 | 12.8971 |
|  |  |  |  | German_2023 | 0.6245 |
|  |  |  |  | German_2023 | 0.0199 |
|  |  |  |  | German_2023 | 4.9889 |
|  |  |  |  | German_2023 | 1.4011 |
|  |  |  |  | German_2023 | 1.1712 |
|  |  |  |  | German_2023 | 0.1309 |
|  |  |  |  | German_2023 | 0.4628 |
|  |  |  |  | German_2023 | 0.1268 |
|  |  |  |  | German_2023 | 3.00E-04 |
|  |  |  |  | German_2023 | 0.0151 |
|  |  |  |  | German_2023 | 0.1653 |
|  |  |  |  | German_2023 | 0.1349 |
|  |  |  |  | German_2023 | 1.5485 |
|  |  |  |  | German_2023 | 4.5183 |
|  |  |  |  | German_2023 | 0.0428 |
|  |  |  |  | German_2023 | 0.8723 |
|  |  |  |  | German_2023 | 4.0327 |
|  |  |  |  | German_2023 | 0.2404 |
|  |  |  |  | German_2023 | 1.3999 |
|  |  |  |  | German_2023 | 2.1633 |
|  |  |  |  | German_2023 | 0.0903 |
|  |  |  |  | German_2023 | 0.3613 |
|  |  |  |  | German_2023 | 6.3689 |
|  |  |  |  | German_2023 | 0.0807 |
|  |  |  |  | German_2023 | 1.4797 |
|  |  |  |  | German_2023 | 0.637 |
|  |  |  |  | German_2023 | 1.5881 |
|  |  |  |  | German_2023 | 3.3872 |
|  |  |  |  | German_2023 | 5.2936 |
|  |  |  |  | German_2023 | 0.001 |
|  |  |  |  | German_2023 | 2.3747 |
|  |  |  |  | German_2023 | 6.4991 |
|  |  |  |  | German_2023 | 0.087 |
|  |  |  |  | German_2023 | 0.0674 |
|  |  |  |  | German_2023 | 1.9264 |
|  |  |  |  | German_2023 | 9.5544 |
|  |  |  |  | German_2023 | 2.024 |
|  |  |  |  | German_2023 | 5.1045 |
|  |  |  |  | German_2023 | 0.1701 |
|  |  |  |  | German_2023 | 3.3016 |
|  |  |  |  | German_2023 | 0.0619 |
|  |  |  |  | German_2023 | 0.1432 |
|  |  |  |  | German_2023 | 0.1782 |
|  |  |  |  | German_2023 | 0.2257 |
|  |  |  |  | German_2023 | 12.5222 |
|  |  |  |  | German_2023 | 0.4108 |
|  |  |  |  | German_2023 | 0.0525 |
|  |  |  |  | German_2023 | 0.0939 |
|  |  |  |  | German_2023 | 0.522 |
|  |  |  |  | German_2023 | 4.6417 |
|  |  |  |  | German_2023 | 0.1769 |
|  |  |  |  | German_2023 | 0.1147 |
|  |  |  |  | German_2023 | 0.1986 |
|  |  |  |  | German_2023 | 0.1629 |
|  |  |  |  | German_2023 | 0.0452 |
|  |  |  |  | German_2023 | 2.5581 |
|  |  |  |  | German_2023 | 0.1351 |
|  |  |  |  | German_2023 | 0.1323 |
|  |  |  |  | German_2023 | 0.4308 |
|  |  |  |  | German_2023 | 1.312 |
|  |  |  |  | German_2023 | 0.2061 |
|  |  |  |  | German_2023 | 0.575 |
|  |  |  |  | German_2023 | 0.9838 |
|  |  |  |  | German_2023 | 0.012 |
|  |  |  |  | German_2023 | 0.8156 |
|  |  |  |  | German_2023 | 3.0938 |
|  |  |  |  | German_2023 | 0.8875 |
|  |  |  |  | German_2023 | 10.8384 |
|  |  |  |  | German_2023 | 0.2547 |
|  |  |  |  | German_2023 | 2.2176 |
|  |  |  |  | German_2023 | 0.9478 |
|  |  |  |  | German_2023 | 0.0485 |
|  |  |  |  | German_2023 | 0.2188 |
|  |  |  |  | German_2023 | 2.0956 |
|  |  |  |  | German_2023 | 1.5115 |
|  |  |  |  | German_2023 | 0.7268 |
|  |  |  |  | German_2023 | 0.1972 |
|  |  |  |  | German_2023 | 0.0222 |
|  |  |  |  | German_2023 | 0.1678 |
|  |  |  |  | German_2023 | 0.0028 |
|  |  |  |  | German_2023 | 0.0469 |
|  |  |  |  | German_2023 | 0.5233 |
|  |  |  |  | German_2023 | 0.1707 |
|  |  |  |  | German_2023 | 1.2999 |
|  |  |  |  | German_2023 | 0.0574 |
|  |  |  |  | German_2023 | 7.6772 |
|  |  |  |  | German_2023 | 0.9611 |
|  |  |  |  | German_2023 | 0.014 |
|  |  |  |  | German_2023 | 0.2304 |
|  |  |  |  | German_2023 | 3.6795 |
|  |  |  |  | German_2023 | 0.0141 |
|  |  |  |  | German_2023 | 0.3231 |
|  |  |  |  | German_2023 | 10.2798 |
|  |  |  |  | German_2023 | 0.0413 |
|  |  |  |  | German_2023 | 8.8291 |
|  |  |  |  | German_2023 | 0.0536 |
|  |  |  |  | German_2023 | 0.1886 |
|  |  |  |  | German_2023 | 0.0636 |
|  |  |  |  | German_2023 | 0.1062 |
|  |  |  |  | German_2023 | 0.3021 |
|  |  |  |  | German_2023 | 32.1636 |
|  |  |  |  | German_2023 | 2.2162 |
|  |  |  |  | German_2023 | 0.4075 |
|  |  |  |  | German_2023 | 51.9496 |
|  |  |  |  | German_2023 | 1.85 |
|  |  |  |  | German_2023 | 0.2238 |
|  |  |  |  | German_2023 | 0.0072 |
|  |  |  |  | German_2023 | 0.3087 |
|  |  |  |  | German_2023 | 0.0011 |
|  |  |  |  | German_2023 | 3.4667 |
|  |  |  |  | German_2023 | 0.5875 |
|  |  |  |  | German_2023 | 0.8722 |
|  |  |  |  | German_2023 | 0.0057 |
|  |  |  |  | German_2023 | 0.6821 |
|  |  |  |  | German_2023 | 0.9898 |
|  |  |  |  | German_2023 | 0.6398 |
|  |  |  |  | German_2023 | 0.0708 |
|  |  |  |  | German_2023 | 0.5661 |
|  |  |  |  | German_2023 | 0.2391 |
|  |  |  |  | German_2023 | 0.6227 |
|  |  |  |  | German_2023 | 0.0976 |
|  |  |  |  | German_2023 | 0.0609 |
|  |  |  |  | German_2023 | 0.1518 |
|  |  |  |  | German_2023 | 42.7077 |
|  |  |  |  | German_2023 | 0.1542 |
|  |  |  |  | German_2023 | 0.1438 |
|  |  |  |  | German_2023 | 0.2675 |
|  |  |  |  | German_2023 | 0.9037 |
|  |  |  |  | German_2023 | 1.0244 |
|  |  |  |  | German_2023 | 1.4832 |
|  |  |  |  | German_2023 | 0.2052 |
|  |  |  |  | German_2023 | 2.9854 |
|  |  |  |  | German_2023 | 0.7938 |
|  |  |  |  | German_2023 | 0.2775 |
|  |  |  |  | German_2023 | 0.2985 |
|  |  |  |  | German_2023 | 0.2499 |
|  |  |  |  | German_2023 | 1.8164 |
|  |  |  |  | German_2023 | 3.3389 |
|  |  |  |  | German_2023 | 0.2504 |
|  |  |  |  | German_2023 | 0.0587 |
|  |  |  |  | German_2023 | 0.5785 |

**Supplementary Table S8C. The abundance of *Cutibacterium* in inhort dataset.**

| Sample | Aabundance | Sample | Aabundance | Sample | Aabundance |
| --- | --- | --- | --- | --- | --- |
| BC_tissue_1 | 0.0025 | BC_adjacent_1 | 0.6278 | benign_tissue_1 | 0.1138 |
| BC_tissue_2 | 0.0019 | BC_adjacent_2 | 0.0231 | benign_tissue_2 | 0.0311 |
| BC_tissue_3 | 0.0394 | BC_adjacent_3 | 0.0106 | benign_tissue_3 | 0.1552 |
| BC_tissue_4 | 0.0304 | BC_adjacent_4 | 0.4879 | benign_tissue_4 | 0.027 |
| BC_tissue_5 | 0.0019 | BC_adjacent_5 | 0.0092 | benign_tissue_5 | 0.1006 |
| BC_tissue_6 | 0.003 | BC_adjacent_6 | 0.4806 | benign_tissue_6 | 0.009 |
| BC_tissue_7 | 0.0028 | BC_adjacent_7 | 1.3758 | benign_tissue_7 | 0.0803 |
| BC_tissue_8 | 0.0088 | BC_adjacent_8 | 0.0069 | benign_tissue_8 | 0.0188 |
| BC_tissue_9 | 0.0019 | BC_adjacent_9 | 0.0491 |  |  |
| BC_tissue_10 | 0.0029 | BC_adjacent_10 | 0.0048 |  |  |
