# Supplementary material for: Microbial diagnostic features identified across populations possess potential antitumor properties in breast cancer
Source: mSystems. 2025 Jun 23;10(7):e00271-25. doi: 10.1128/msystems.00271-25 (PMC12282184; doi:10.1128/msystems.00271-25)
Supplement: Table S9 — Differential pathways between BC_tissue and BC_adjacent. [file msystems.00271-25-s0009.doc]

**Table S9A. The abundance of 96 differential pathways between BC_tissue and BC_adjacent.**

| **Differential pathways** | **BC_tissue** | **BC_adjacent** | **fold change** | ***P* value** |
| --- | --- | --- | --- | --- |
| Valine, leucine and isoleucine biosynthesis | 45443.77236 | 71954.18274 | 1.58336729 | 0.009576417 |
| C5-Branched dibasic acid metabolism | 39013.06457 | 64953.75863 | 1.66492326 | 0.000222643 |
| Fatty acid biosynthesis | 40312.62485 | 62209.09657 | 1.54316661 | 0.004461857 |
| Lipoic acid metabolism | 34088.27708 | 57042.9151 | 1.67338804 | 0.000931866 |
| Pantothenate and CoA biosynthesis | 37915.59253 | 58054.77706 | 1.53115838 | 0.02937253 |
| D-Alanine metabolism | 36143.82436 | 51047.48598 | 1.41234324 | 0.041406745 |
| Bacterial chemotaxis | 32701.73875 | 52825.56746 | 1.61537488 | 0.021443142 |
| Carbon fixation pathways in prokaryotes | 27935.07041 | 42814.38651 | 1.53263929 | 0.025761284 |
| Pyruvate metabolism | 28978.89366 | 42717.76747 | 1.47409932 | 0.012366654 |
| Flagellar assembly | 21264.46507 | 37504.72035 | 1.76372743 | 0.005953832 |
| Citrate cycle (TCA cycle) | 27157.62791 | 40471.6655 | 1.49025039 | 0.010186482 |
| Sulfur relay system | 28563.62101 | 42155.63501 | 1.47585052 | 0.002034041 |
| Synthesis and degradation of ketone bodies | 31840.7132 | 49623.55079 | 1.5584937 | 0.00028842 |
| Glycine, serine and threonine metabolism | 25957.47761 | 36966.25009 | 1.42410794 | 0.037406751 |
| Glycolysis / Gluconeogenesis | 25113.20885 | 34477.34189 | 1.3728768 | 0.039401372 |
| Propanoate metabolism | 21889.60133 | 34396.66787 | 1.57137023 | 0.000678796 |
| Bacterial secretion system | 20994.34367 | 31265.11151 | 1.48921595 | 0.01585438 |
| Base excision repair | 20275.38756 | 28523.25423 | 1.40679206 | 0.037334557 |
| Butanoate metabolism | 20563.35424 | 31216.64176 | 1.51807149 | 0.001842935 |
| Tetracycline biosynthesis | 11829.50101 | 22495.87269 | 1.90167554 | 9.18E-05 |
| Glyoxylate and dicarboxylate metabolism | 17922.35762 | 26865.48748 | 1.49899294 | 0.016733848 |
| ABC transporters | 17763.91448 | 25231.24661 | 1.42036524 | 0.022822459 |
| Valine, leucine and isoleucine degradation | 19030.49032 | 28000.7832 | 1.47136425 | 0.001546663 |
| Glutathione metabolism | 18321.1817 | 26722.78899 | 1.45857344 | 0.01427911 |
| beta-Alanine metabolism | 16610.90899 | 24344.60133 | 1.46557912 | 0.015015818 |
| Biosynthesis of unsaturated fatty acids | 13276.5596 | 20973.35453 | 1.57972812 | 0.000719062 |
| Fatty acid degradation | 17296.95101 | 25452.58436 | 1.47150699 | 0.001949273 |
| Oxidative phosphorylation | 12195.7106 | 18632.88717 | 1.527823 | 0.016769901 |
| Nitrotoluene degradation | 6035.772962 | 13724.57929 | 2.27387269 | 0.033084338 |
| Glycerophospholipid metabolism | 12951.1365 | 18161.91594 | 1.40234148 | 0.021622754 |
| Toluene degradation | 11188.83369 | 17134.31628 | 1.53137644 | 0.002530489 |
| Geraniol degradation | 16239.12044 | 23051.38721 | 1.41949728 | 0.002979006 |
| Phenylalanine metabolism | 10431.83365 | 16315.6497 | 1.5640251 | 0.004585024 |
| Tryptophan metabolism | 11552.62903 | 17325.86228 | 1.49973328 | 0.000263888 |
| Two-component system | 10867.55241 | 15487.26602 | 1.42509237 | 0.008916317 |
| Tyrosine metabolism | 9316.52607 | 14073.35643 | 1.51057984 | 0.002465399 |
| D-Arginine and D-ornithine metabolism | 10167.47437 | 15345.29528 | 1.5092534 | 1.09E-05 |
| Ascorbate and aldarate metabolism | 8677.625923 | 12814.16305 | 1.47668996 | 0.000667131 |
| Lysine degradation | 9785.721913 | 13865.83323 | 1.41694536 | 0.001580597 |
| Secondary bile acid biosynthesis | 10844.00463 | 7092.977897 | 0.65409211 | 7.57E-06 |
| Inositol phosphate metabolism | 6357.394792 | 9700.984832 | 1.52593714 | 0.001567792 |
| Benzoate degradation | 8936.614085 | 13699.21014 | 1.53293071 | 0.000642494 |
| Caprolactam degradation | 8569.936282 | 13437.02757 | 1.56792619 | 3.38E-05 |
| Phosphotransferase system (PTS) | 8056.388615 | 9231.556364 | 1.14586781 | 0.004781437 |
| Other glycan degradation | 15043.11548 | 6313.466072 | 0.41969139 | 0.000112622 |
| Aminobenzoate degradation | 6471.281859 | 10426.80857 | 1.61124315 | 0.000132287 |
| Styrene degradation | 7485.025196 | 11986.36381 | 1.60137922 | 0.000116695 |
| Biofilm formation - Vibrio cholerae | 4822.869473 | 7124.701066 | 1.47727429 | 0.048395958 |
| Chlorocyclohexane and chlorobenzene degradation | 4567.509156 | 8807.123456 | 1.92821145 | 5.73E-05 |
| Fluorobenzoate degradation | 5100.780932 | 9252.608412 | 1.81395918 | 0.001316171 |
| Dioxin degradation | 4341.661061 | 6334.996754 | 1.45911822 | 0.001980673 |
| Atrazine degradation | 4114.564385 | 6888.827374 | 1.67425436 | 0.031519977 |
| Linoleic acid metabolism | 3921.068646 | 4613.124068 | 1.17649664 | 0.02334718 |
| Polyketide sugar unit biosynthesis | 4412.399824 | 4045.969076 | 0.91695432 | 1.99E-06 |
| Penicillin and cephalosporin biosynthesis | 2284.995626 | 3336.380996 | 1.46012577 | 1.52E-05 |
| Glycosaminoglycan degradation | 4937.535003 | 1978.317967 | 0.40066915 | 8.39E-05 |
| Ribosome biogenesis in eukaryotes | 1508.622948 | 2099.801552 | 1.39186637 | 0.008651815 |
| Sphingolipid metabolism | 4002.22845 | 1985.729821 | 0.49615604 | 1.20E-05 |
| Non-homologous end-joining | 1642.266762 | 2645.500169 | 1.61088334 | 3.35E-05 |
| Carotenoid biosynthesis | 2073.684802 | 3010.298654 | 1.45166645 | 1.56E-05 |
| Staphylococcus aureus infection | 2034.895892 | 1825.95047 | 0.89731886 | 0.006775931 |
| Primary bile acid biosynthesis | 1462.338942 | 1253.050867 | 0.85688128 | 0.000731568 |
| Epithelial cell signaling in Helicobacter pylori infection | 2115.749351 | 1856.014086 | 0.87723722 | 0.012066486 |
| Polycyclic aromatic hydrocarbon degradation | 1081.53719 | 1788.479142 | 1.65364553 | 0.000498759 |
| Apoptosis | 1068.75163 | 1184.247962 | 1.10806658 | 0.013755263 |
| Novobiocin biosynthesis | 1891.353121 | 1688.750013 | 0.89287928 | 0.030882458 |
| Neomycin, kanamycin and gentamicin biosynthesis | 1118.171019 | 595.980821 | 0.53299613 | 0.000471001 |
| Betalain biosynthesis | 534.1351732 | 1078.655795 | 2.01944348 | 0.003879476 |
| Flavonoid biosynthesis | 372.0138615 | 776.4105487 | 2.08704736 | 0.001111891 |
| African trypanosomiasis | 602.6443597 | 683.9455205 | 1.13490736 | 0.010209562 |
| Steroid biosynthesis | 286.1768078 | 833.3815672 | 2.91212126 | 5.21E-07 |
| NOD-like receptor signaling pathway | 790.7283446 | 716.3963872 | 0.90599558 | 0.006681706 |
| Steroid hormone biosynthesis | 655.4898225 | 598.632558 | 0.91325988 | 0.002278891 |
| Photosynthesis - antenna proteins | 148.9102745 | 980.4001667 | 6.58383157 | 0.024231921 |
| Protein digestion and absorption | 642.0840139 | 223.735081 | 0.34845141 | 2.19E-06 |
| Meiosis - yeast | 185.8525931 | 293.8442564 | 1.58106084 | 0.014248158 |
| Proteasome | 213.5891727 | 342.5452041 | 1.60375734 | 3.52E-06 |
| Renin-angiotensin system | 81.37261169 | 158.0107251 | 1.94181705 | 1.39E-05 |
| Basal transcription factors | 151.8144862 | 105.4988564 | 0.69491956 | 0.000525331 |
| Biosynthesis of type II polyketide products | 81.36255411 | 264.8566154 | 3.25526427 | 0.000642652 |
| Bacterial invasion of epithelial cells | 39.41907922 | 78.73442359 | 1.99736841 | 0.007881183 |
| Vibrio cholerae infection | 33.39275152 | 88.19612872 | 2.64117585 | 2.03E-09 |
| Pathways in cancer | 81.09177489 | 95.10437795 | 1.17279931 | 0.007236966 |
| Shigellosis | 9.4258013 | 43.24036769 | 4.5874474 | 2.43E-06 |
| Parkinson disease | 139.5882745 | 33.15745949 | 0.23753757 | 5.55E-09 |
| Toxoplasmosis | 15.30543377 | 69.58801385 | 4.54662148 | 0.020659748 |
| Systemic lupus erythematosus | 17.88034719 | 14.85330205 | 0.83070546 | 2.86E-06 |
| Sesquiterpenoid and triterpenoid biosynthesis | 14.22520779 | 12.16644103 | 0.85527334 | 0.020536738 |
| Wnt signaling pathway | 2.47591861 | 3.43477282 | 1.3872721 | 0.027554538 |
| Vasopressin-regulated water reabsorption | 1.23185714 | 0.32699846 | 0.26545161 | 0.002032805 |
| Brassinosteroid biosynthesis | 0.63549784 | 0.45641026 | 0.71819325 | 0.044745637 |
| Calcium signaling pathway | 0.04264372 | 0.08369179 | 1.96258181 | 0.022544986 |
| Salivary secretion | 0.37335065 | 0.26341949 | 0.70555519 | 5.13E-05 |
| Phagosome | 0.08979048 | 0.25282256 | 2.81569466 | 3.39E-07 |
| Complement and coagulation cascades | 0.00259697 | 0.05772872 | 22.22926129 | 0.022001239 |
| Adherens junction | 0.06608961 | 0.5463241 | 8.26641433 | 0.004727975 |

**Table S9B. The abundance of 188 differential pathways between BC_tissue and normal-tissue.**

| **Differential pathways** | **BC_tissue** | **normal_adjacent** | **fold change** | ***P* value** |
| --- | --- | --- | --- | --- |
| Biosynthesis of ansamycins | 68166.63931 | 272963.9244 | 4.00436236 | 2.03E-48 |
| Valine, leucine and isoleucine biosynthesis | 45443.77236 | 164904.5019 | 3.62875909 | 1.62E-49 |
| C5-Branched dibasic acid metabolism | 39013.06457 | 147248.7235 | 3.77434393 | 2.76E-52 |
| D-Glutamine and D-glutamate metabolism | 41988.3411 | 146768.4057 | 3.49545616 | 2.28E-47 |
| Fatty acid biosynthesis | 40312.62485 | 138441.0414 | 3.43418574 | 9.81E-48 |
| Biosynthesis of vancomycin group antibiotics | 34937.08905 | 142711.5753 | 4.08481586 | 5.05E-44 |
| Lipoic acid metabolism | 34088.27708 | 135246.2559 | 3.96752982 | 9.95E-53 |
| Pantothenate and CoA biosynthesis | 37915.59253 | 131628.3737 | 3.4716159 | 7.23E-48 |
| Peptidoglycan biosynthesis | 36608.55013 | 116573.5792 | 3.18432658 | 1.50E-44 |
| Aminoacyl-tRNA biosynthesis | 33256.87591 | 114992.5418 | 3.45770728 | 1.67E-47 |
| D-Alanine metabolism | 36143.82436 | 110988.2922 | 3.07074014 | 1.33E-44 |
| One carbon pool by folate | 35461.01853 | 111875.6987 | 3.15489242 | 1.24E-43 |
| Ribosome | 32005.92301 | 111181.2471 | 3.473771 | 5.34E-47 |
| Protein export | 31810.36342 | 106945.0704 | 3.36195689 | 2.98E-47 |
| Bacterial chemotaxis | 32701.73875 | 100313.0922 | 3.06751555 | 5.95E-43 |
| Lysine biosynthesis | 30938.50681 | 103832.2538 | 3.35608484 | 5.12E-47 |
| Streptomycin biosynthesis | 29469.82457 | 104101.3858 | 3.53247389 | 1.77E-44 |
| Cell cycle - Caulobacter | 30807.67852 | 100085.3931 | 3.24871583 | 5.25E-46 |
| Mismatch repair | 31993.4083 | 98777.12872 | 3.08742125 | 4.30E-44 |
| Lipopolysaccharide biosynthesis | 22453.01964 | 102293.0169 | 4.55586903 | 1.50E-52 |
| Carbon fixation pathways in prokaryotes | 27935.07041 | 97524.87904 | 3.49112702 | 1.33E-48 |
| Carbon fixation in photosynthetic organisms | 29513.83851 | 95870.30926 | 3.2483172 | 2.68E-44 |
| Alanine, aspartate and glutamate metabolism | 32329.53215 | 91248.24439 | 2.82244246 | 3.08E-39 |
| Homologous recombination | 28954.25548 | 90660.78662 | 3.13117313 | 1.13E-44 |
| Pyruvate metabolism | 28978.89366 | 88063.79392 | 3.03889427 | 1.48E-43 |
| Terpenoid backbone biosynthesis | 28439.82492 | 88886.86425 | 3.12543641 | 8.85E-45 |
| Flagellar assembly | 21264.46507 | 91478.82144 | 4.30195733 | 1.05E-50 |
| Pentose phosphate pathway | 30268.62144 | 84734.12286 | 2.79940476 | 3.91E-41 |
| Citrate cycle (TCA cycle) | 27157.62791 | 85574.30543 | 3.15102283 | 1.58E-45 |
| Phenylalanine, tyrosine and tryptophan biosynthesis | 25583.57428 | 87316.26289 | 3.41298139 | 5.04E-48 |
| Sulfur relay system | 28563.62101 | 80983.24551 | 2.83518835 | 6.07E-42 |
| Thiamine metabolism | 29591.19312 | 81337.0636 | 2.74869159 | 8.38E-41 |
| Synthesis and degradation of ketone bodies | 31840.7132 | 72374.94228 | 2.27303144 | 2.77E-32 |
| Glycine, serine and threonine metabolism | 25957.47761 | 77376.28739 | 2.98088622 | 5.73E-44 |
| Nicotinate and nicotinamide metabolism | 23930.13381 | 79238.64096 | 3.31124939 | 1.39E-45 |
| Histidine metabolism | 26952.44024 | 75980.65849 | 2.81906417 | 2.64E-42 |
| Biotin metabolism | 27645.78078 | 73046.12621 | 2.64221607 | 1.39E-38 |
| Folate biosynthesis | 28168.40837 | 72621.72064 | 2.57812652 | 1.21E-37 |
| DNA replication | 24511.59906 | 75371.37758 | 3.07492699 | 5.48E-44 |
| Glycolysis / Gluconeogenesis | 25113.20885 | 71843.57046 | 2.86078816 | 1.24E-41 |
| Propanoate metabolism | 21889.60133 | 69692.24077 | 3.18380585 | 3.96E-46 |
| Cysteine and methionine metabolism | 24112.73794 | 66985.77139 | 2.77802428 | 8.88E-40 |
| Vitamin B6 metabolism | 23351.44075 | 66159.75509 | 2.83321941 | 5.13E-42 |
| Pyrimidine metabolism | 22425.55377 | 65846.25846 | 2.9362155 | 3.34E-41 |
| Bacterial secretion system | 20994.34367 | 63529.48269 | 3.02602852 | 5.87E-44 |
| Selenocompound metabolism | 23357.17928 | 59097.37875 | 2.53015906 | 1.00E-35 |
| Base excision repair | 20275.38756 | 60246.56958 | 2.97141396 | 2.28E-42 |
| Butanoate metabolism | 20563.35424 | 55555.60203 | 2.70167996 | 1.42E-39 |
| RNA polymerase | 19144.41981 | 57768.18872 | 3.01749488 | 2.04E-38 |
| Tetracycline biosynthesis | 11829.50101 | 62709.25312 | 5.3010903 | 2.31E-51 |
| Drug metabolism - other enzymes | 22107.28504 | 53813.25732 | 2.43418661 | 1.77E-30 |
| Purine metabolism | 18467.74071 | 54734.6585 | 2.9637983 | 6.93E-43 |
| Riboflavin metabolism | 17817.6471 | 53560.66677 | 3.006046 | 1.13E-43 |
| Amino sugar and nucleotide sugar metabolism | 18704.02657 | 50433.62545 | 2.69640472 | 7.97E-36 |
| Glyoxylate and dicarboxylate metabolism | 17922.35762 | 45072.70473 | 2.51488703 | 2.15E-36 |
| Nucleotide excision repair | 15201.80126 | 47968.14579 | 3.15542513 | 3.80E-43 |
| Arginine and proline metabolism | 16888.38661 | 43873.42004 | 2.59784555 | 3.81E-38 |
| ABC transporters | 17763.91448 | 42462.90301 | 2.39040235 | 5.83E-33 |
| Valine, leucine and isoleucine degradation | 19030.49032 | 40292.8667 | 2.11727948 | 3.93E-25 |
| Glutathione metabolism | 18321.1817 | 38994.95335 | 2.1284082 | 2.02E-16 |
| beta-Alanine metabolism | 16610.90899 | 39922.228 | 2.40337407 | 5.00E-35 |
| Sulfur metabolism | 18863.31786 | 38308.52968 | 2.03084791 | 1.12E-23 |
| Galactose metabolism | 15953.79765 | 40963.01591 | 2.56760282 | 2.52E-33 |
| Taurine and hypotaurine metabolism | 16917.06228 | 37184.64314 | 2.19805558 | 1.56E-27 |
| Biosynthesis of unsaturated fatty acids | 13276.5596 | 39710.95242 | 2.99105744 | 1.08E-42 |
| Starch and sucrose metabolism | 15305.24906 | 38394.83027 | 2.50860539 | 4.32E-31 |
| Fatty acid degradation | 17296.95101 | 33666.50686 | 1.94638389 | 1.88E-22 |
| Oxidative phosphorylation | 12195.7106 | 38830.48058 | 3.18394572 | 4.41E-45 |
| Nitrotoluene degradation | 6035.772962 | 41962.20314 | 6.95225009 | 2.97E-60 |
| Porphyrin and chlorophyll metabolism | 13857.98218 | 34542.89613 | 2.49263534 | 6.44E-36 |
| Zeatin biosynthesis | 12116.63744 | 37022.92789 | 3.05554475 | 2.29E-38 |
| Glycerophospholipid metabolism | 12951.1365 | 34724.29401 | 2.68117736 | 1.54E-39 |
| RNA degradation | 12635.49331 | 34872.13094 | 2.75985512 | 8.31E-40 |
| Fructose and mannose metabolism | 15808.78174 | 33331.60699 | 2.1084235 | 2.69E-22 |
| Photosynthesis | 9072.425597 | 34210.2634 | 3.7707957 | 4.72E-30 |
| Toluene degradation | 11188.83369 | 30702.01367 | 2.74398695 | 6.22E-40 |
| Pentose and glucuronate interconversions | 12857.80191 | 29906.02219 | 2.32590472 | 5.90E-30 |
| Geraniol degradation | 16239.12044 | 23789.95725 | 1.46497819 | 0.00042623 |
| Tropane, piperidine and pyridine alkaloid biosynthesis | 9942.195611 | 29411.11646 | 2.95821141 | 3.52E-37 |
| Nitrogen metabolism | 13799.86074 | 25893.69092 | 1.87637335 | 1.38E-20 |
| Phenylalanine metabolism | 10431.83365 | 25526.06191 | 2.44693913 | 5.31E-33 |
| Tryptophan metabolism | 11552.62903 | 24479.40499 | 2.11894668 | 3.89E-26 |
| Cyanoamino acid metabolism | 13011.2862 | 24667.60865 | 1.89586243 | 4.59E-08 |
| Ubiquinone and other terpenoid-quinone biosynthesis | 12881.86737 | 23392.64374 | 1.81593577 | 1.54E-16 |
| Two-component system | 10867.55241 | 25154.53667 | 2.314646 | 1.21E-32 |
| Methane metabolism | 9430.810685 | 26705.18313 | 2.83169539 | 2.22E-41 |
| Glycerolipid metabolism | 10164.09588 | 23113.38289 | 2.27402252 | 1.17E-31 |
| Tyrosine metabolism | 9316.52607 | 22276.98188 | 2.39112537 | 1.72E-30 |
| Chloroalkane and chloroalkene degradation | 10342.22567 | 21246.55162 | 2.05435003 | 0.00423471 |
| D-Arginine and D-ornithine metabolism | 10167.47437 | 19627.9794 | 1.93046756 | 2.51E-19 |
| Ascorbate and aldarate metabolism | 8677.625923 | 19575.04282 | 2.25580625 | 2.55E-31 |
| Lysine degradation | 9785.721913 | 18017.7144 | 1.84122485 | 8.51E-19 |
| Secondary bile acid biosynthesis | 10844.00463 | 19070.1231 | 1.75858677 | 0.026893156 |
| Inositol phosphate metabolism | 6357.394792 | 19715.87143 | 3.10125013 | 7.77E-43 |
| Benzoate degradation | 8936.614085 | 16422.76778 | 1.83769464 | 1.06E-19 |
| Caprolactam degradation | 8569.936282 | 16295.80242 | 1.901508 | 1.27E-10 |
| Phosphotransferase system (PTS) | 8056.388615 | 16906.43856 | 2.09851329 | 0.00167571 |
| Metabolism of xenobiotics by cytochrome P450 | 5675.78052 | 15207.88994 | 2.67943587 | 0.004405587 |
| Other glycan degradation | 15043.11548 | 11795.22438 | 0.78409452 | 0.027812295 |
| Aminobenzoate degradation | 6471.281859 | 14255.71871 | 2.20292038 | 2.97E-27 |
| Styrene degradation | 7485.025196 | 12852.74693 | 1.71712808 | 4.03E-09 |
| Limonene and pinene degradation | 7375.717773 | 13258.38396 | 1.79757203 | 0.01960655 |
| Biofilm formation - Vibrio cholerae | 4822.869473 | 15308.27159 | 3.17410033 | 1.75E-42 |
| Peroxisome | 6708.924175 | 12041.64557 | 1.79486983 | 1.04E-15 |
| Bisphenol degradation | 5187.338442 | 11414.76681 | 2.20050551 | 0.003976581 |
| Chlorocyclohexane and chlorobenzene degradation | 4567.509156 | 10626.9925 | 2.32664941 | 1.37E-17 |
| Fluorobenzoate degradation | 5100.780932 | 8569.818398 | 1.68009929 | 5.82E-10 |
| Dioxin degradation | 4341.661061 | 8851.044859 | 2.03863101 | 2.77E-05 |
| Atrazine degradation | 4114.564385 | 8172.511884 | 1.98623988 | 7.54E-09 |
| Plant-pathogen interaction | 3795.301298 | 8246.018679 | 2.17269145 | 5.40E-26 |
| Ethylbenzene degradation | 3077.671537 | 7522.549856 | 2.44423414 | 0.000601757 |
| Polyketide sugar unit biosynthesis | 4412.399824 | 7358.621761 | 1.66771418 | 1.35E-12 |
| Phosphonate and phosphinate metabolism | 3878.624074 | 7090.179522 | 1.82801411 | 1.68E-05 |
| Xylene degradation | 1842.902303 | 5216.992936 | 2.83085703 | 8.32E-06 |
| Penicillin and cephalosporin biosynthesis | 2284.995626 | 4618.424053 | 2.02119601 | 9.62E-12 |
| N-Glycan biosynthesis | 1143.457538 | 5820.106056 | 5.08991883 | 4.04E-52 |
| Glycosaminoglycan degradation | 4937.535003 | 3816.272902 | 0.77291055 | 0.007932393 |
| Ribosome biogenesis in eukaryotes | 1508.622948 | 4357.024652 | 2.88808059 | 1.21E-42 |
| Sphingolipid metabolism | 4002.22845 | 3109.682717 | 0.77698781 | 0.001633888 |
| Insulin signaling pathway | 1404.154886 | 4321.718529 | 3.07780756 | 1.91E-40 |
| Non-homologous end-joining | 1642.266762 | 3786.361614 | 2.30557039 | 4.40E-10 |
| Carotenoid biosynthesis | 2073.684802 | 3146.047216 | 1.51712894 | 6.99E-14 |
| Naphthalene degradation | 300.2098948 | 4807.822982 | 16.01487181 | 6.44E-09 |
| Staphylococcus aureus infection | 2034.895892 | 2967.999963 | 1.45855126 | 8.68E-07 |
| Retinol metabolism | 1389.577311 | 2693.928369 | 1.9386675 | 0.007702033 |
| Epithelial cell signaling in Helicobacter pylori infection | 2115.749351 | 1732.640127 | 0.81892504 | 2.34E-05 |
| Polycyclic aromatic hydrocarbon degradation | 1081.53719 | 2255.320336 | 2.08529152 | 2.94E-09 |
| RNA transport | 1006.844057 | 2515.869322 | 2.49876761 | 2.89E-36 |
| Protein processing in endoplasmic reticulum | 827.0601442 | 2447.526097 | 2.95930848 | 3.53E-34 |
| Novobiocin biosynthesis | 1891.353121 | 789.5905643 | 0.4174739 | 5.30E-28 |
| Neomycin, kanamycin and gentamicin biosynthesis | 1118.171019 | 1124.827116 | 1.00595266 | 1.74E-14 |
| Betalain biosynthesis | 534.1351732 | 1162.11755 | 2.17569935 | 2.37E-10 |
| Flavonoid biosynthesis | 372.0138615 | 1128.256461 | 3.03283447 | 2.82E-20 |
| Steroid biosynthesis | 286.1768078 | 947.2995865 | 3.31018993 | 1.37E-27 |
| Arachidonic acid metabolism | 752.8938797 | 717.0533306 | 0.95239628 | 2.88E-08 |
| Lysosome | 599.0162043 | 926.2524971 | 1.54628955 | 3.87E-07 |
| NOD-like receptor signaling pathway | 790.7283446 | 607.4754874 | 0.76824802 | 5.22E-07 |
| Steroid hormone biosynthesis | 655.4898225 | 636.7475703 | 0.97140726 | 0.001269059 |
| Photosynthesis - antenna proteins | 148.9102745 | 663.3803151 | 4.45489955 | 0.01203876 |
| Protein digestion and absorption | 642.0840139 | 352.0220355 | 0.54824918 | 3.58E-06 |
| Meiosis - yeast | 185.8525931 | 476.0600776 | 2.56149279 | 2.09E-05 |
| Proteasome | 213.5891727 | 408.202647 | 1.91115796 | 1.02E-07 |
| Alzheimer disease | 358.7123662 | 140.3337807 | 0.39121534 | 5.09E-20 |
| Phenylpropanoid biosynthesis | 298.0175061 | 11.63495654 | 0.03904118 | 3.14E-12 |
| Renin-angiotensin system | 81.37261169 | 147.7253769 | 1.8154189 | 4.24E-05 |
| Basal transcription factors | 151.8144862 | 119.4927104 | 0.78709689 | 5.40E-14 |
| PPAR signaling pathway | 164.6078403 | 22.90213171 | 0.13913148 | 0.001962948 |
| Biosynthesis of type II polyketide backbone | 96.95131342 | 73.49999623 | 0.75811243 | 1.53E-07 |
| Bacterial invasion of epithelial cells | 39.41907922 | 104.5812608 | 2.65306199 | 1.02E-22 |
| Vibrio cholerae infection | 33.39275152 | 93.44280554 | 2.79829608 | 5.23E-27 |
| Huntington disease | 109.6035113 | 16.06708315 | 0.14659278 | 4.54E-13 |
| Pathways in cancer | 81.09177489 | 37.87467228 | 0.46705936 | 3.69E-05 |
| Shigellosis | 9.4258013 | 79.54541375 | 8.43911422 | 1.94E-52 |
| Parkinson disease | 139.5882745 | 15.17902195 | 0.10874138 | 1.19E-33 |
| Indole alkaloid biosynthesis | 76.8697316 | 6.059051 | 0.07882233 | 1.35E-13 |
| Isoflavonoid biosynthesis | 30.47991039 | 45.9758102 | 1.50839716 | 0.00013879 |
| Adipocytokine signaling pathway | 104.5788442 | 0 | 0 | 3.05E-06 |
| Toxoplasmosis | 15.30543377 | 23.6230286 | 1.54344065 | 0.008323284 |
| mRNA surveillance pathway | 30.14710433 | 29.04141175 | 0.96332342 | 2.83E-11 |
| Systemic lupus erythematosus | 17.88034719 | 18.25800976 | 1.02112166 | 1.53E-18 |
| Sesquiterpenoid and triterpenoid biosynthesis | 14.22520779 | 3.97840576 | 0.27967295 | 9.92E-23 |
| Chagas disease (American trypanosomiasis) | 1.40139307 | 5.08446098 | 3.62814764 | 0.00650055 |
| Pathogenic Escherichia coli infection | 3.23537749 | 5.95905477 | 1.84184219 | 0.008557035 |
| Dorso-ventral axis formation | 0.49242294 | 4.95731508 | 10.06718948 | 2.02E-14 |
| Endocytosis | 2.45843723 | 6.18407605 | 2.51545005 | 1.49E-07 |
| Wnt signaling pathway | 2.47591861 | 1.98364523 | 0.80117546 | 0.009397887 |
| Biosynthesis of 12-, 14- and 16-membered macrolides | 3.03049004 | 0.91626364 | 0.30234834 | 2.27E-15 |
| p53 signaling pathway | 4.84223506 | 0 | 0 | 0.000205629 |
| Clavulanic acid biosynthesis | 2.22222381 | 0.10938692 | 0.04922408 | 4.45E-17 |
| Cell cycle | 1.6179684 | 0.00039069 | 0.00024147 | 3.39E-14 |
| Bile secretion | 1.66971212 | 0.05535499 | 0.03315241 | 3.29E-05 |
| Vasopressin-regulated water reabsorption | 1.23185714 | 0.32740266 | 0.26577973 | 0.000149057 |
| ECM-receptor interaction | 0.52501861 | 0.39606829 | 0.75438905 | 7.84E-15 |
| Neuroactive ligand-receptor interaction | 0.3992355 | 0.46327273 | 1.16039964 | 3.08E-07 |
| Brassinosteroid biosynthesis | 0.63549784 | 0.31219512 | 0.49126072 | 1.80E-15 |
| Olfactory transduction | 0.54691688 | 0.01191796 | 0.02179117 | 1.39E-12 |
| Salivary secretion | 0.37335065 | 0.07629911 | 0.20436315 | 3.41E-13 |
| Phagosome | 0.08979048 | 0.18934634 | 2.10875752 | 1.81E-11 |
| Complement and coagulation cascades | 0.00259697 | 0.28691774 | 110.4817429 | 0.00082977 |
| Glycosphingolipid biosynthesis - lacto and neolacto series | 0.28504632 | 0.00017051 | 0.00059818 | 1.98E-19 |
| Vascular smooth muscle contraction | 0.07966277 | 0 | 0 | 8.71E-06 |
| Regulation of actin cytoskeleton | 0.04400476 | 0 | 0 | 0.000205629 |
| Plant hormone signal transduction | 0.01111732 | 0 | 0 | 3.05E-06 |
| ErbB signaling pathway | 0.01096623 | 0.00432373 | 0.39427621 | 0.003698516 |
| Glycosaminoglycan biosynthesis - chondroitin sulfate / dermatan sulfate | 0.01298745 | 0 | 0 | 0.015438884 |
| Ubiquitin mediated proteolysis | 0.00230866 | 0.00587583 | 2.54512858 | 0.008808612 |
| Malaria | 0.01564026 | 0 | 0 | 0.048233546 |
| Cell cycle - yeast | 0.00245844 | 0 | 0 | 0.015438884 |

**Table S9C. The abundance of 6 rate-limiting enzymes linked to the propanoate biosynthesis pathway in BC_tissue samples.**

| **Samples** | ***acs*** | ***pduW*** | ***ackA*** | ***pct*** | ***acdA*** | ***prpE*** |
| --- | --- | --- | --- | --- | --- | --- |
| Hoskinson_2022 | 27067.67 | 7.69 | 12945.33 | 7662.66 | 1.33 | 1271.29 |
| Hoskinson_2022 | 84060.82 | 0 | 29766.36 | 25133 | 0 | 199.83 |
| Hoskinson_2022 | 94647.39 | 0 | 55712.55 | 22368.5 | 0 | 1478.8 |
| Hoskinson_2022 | 57724.88 | 9.35 | 32910.93 | 144.42 | 8.33 | 1657.13 |
| Hoskinson_2022 | 81997.88 | 0.64 | 41438.56 | 0.75 | 0 | 20628.54 |
| Hoskinson_2022 | 104580.67 | 68.71 | 73949.68 | 69.75 | 0.67 | 2721.37 |
| Hoskinson_2022 | 86087.98 | 0 | 72939.93 | 1.25 | 0 | 7.7 |
| Hoskinson_2022 | 108998.69 | 1 | 63682.07 | 9 | 0 | 7804.43 |
| Hoskinson_2022 | 55469.72 | 0 | 43018.79 | 3520.25 | 550 | 6122.87 |
| Hoskinson_2022 | 91650.91 | 0.33 | 33698.68 | 11064.83 | 0 | 314.73 |
| Hoskinson_2022 | 87240.73 | 0 | 26804.05 | 21216 | 0 | 29152.97 |
| Hoskinson_2022 | 98424.62 | 0 | 43884.55 | 1.25 | 0 | 5923.83 |
| Hoskinson_2022 | 97466.05 | 0 | 51297.89 | 0 | 0 | 1284.43 |
| Hoskinson_2022 | 26477.37 | 0.25 | 73401.86 | 579.25 | 544 | 598 |
| Hoskinson_2022 | 108939.66 | 0.33 | 54883.1 | 9.08 | 1.33 | 165.1 |
| Hoskinson_2022 | 86469.6 | 0.81 | 20985.9 | 13.34 | 0 | 10686.18 |
| Hoskinson_2022 | 79827.78 | 5.9 | 49540.36 | 1393.98 | 0 | 7473.31 |
| Hoskinson_2022 | 102331.06 | 1.81 | 64816.6 | 2.81 | 0 | 11643.93 |
| Hoskinson_2022 | 57662.49 | 4.29 | 53645.07 | 17.71 | 0 | 4604.2 |
| Hoskinson_2022 | 83509.46 | 10460.71 | 55714.71 | 10492.64 | 0 | 33.82 |
| Hoskinson_2022 | 86113.81 | 10892.33 | 58382.27 | 10928.33 | 0 | 53.33 |
| Hoskinson_2022 | 140046.59 | 2.92 | 54048.57 | 18.78 | 0 | 19956.64 |
| Hoskinson_2022 | 112021.03 | 0.81 | 55997.57 | 3142.09 | 0 | 2328.5 |
| Hoskinson_2022 | 45528.93 | 1.67 | 46398.85 | 622.07 | 0 | 10077.3 |
| Hoskinson_2022 | 101401.1 | 8.86 | 56132.09 | 40.03 | 0 | 108.5 |
| Hoskinson_2022 | 117983.52 | 1.33 | 48640.02 | 17.33 | 0 | 519.33 |
| Hoskinson_2022 | 84525.76 | 7 | 98250.35 | 9 | 0 | 10551.77 |
| Hoskinson_2022 | 186876.42 | 1.47 | 95651.59 | 4504.72 | 0 | 11541.6 |
| Hoskinson_2022 | 108772.24 | 0.47 | 69073.4 | 2811.97 | 0 | 9835.2 |
| Hoskinson_2022 | 202772.99 | 0 | 81770.74 | 1292 | 426 | 45665.2 |
| Hoskinson_2022 | 114018.73 | 8.75 | 48316.71 | 92 | 0 | 16879 |
| Hoskinson_2022 | 85618.33 | 1 | 42162.16 | 15.75 | 0 | 30.75 |
| Hoskinson_2022 | 60131.07 | 4.09 | 37644.15 | 4574.31 | 0 | 4597.28 |
| Hoskinson_2022 | 10132.07 | 206.61 | 32356.41 | 225.15 | 0 | 10.94 |
| Hoskinson_2022 | 88817.13 | 1 | 37666.81 | 17.25 | 1.67 | 125.07 |
| Hoskinson_2022 | 85878.83 | 1.83 | 37126.5 | 705.33 | 0.33 | 7277.33 |
| Hoskinson_2022 | 66147.64 | 0 | 34667.58 | 19 | 116 | 6474.95 |
| Hoskinson_2022 | 64065.57 | 169 | 32888.91 | 18044.5 | 0 | 3908.6 |
| Hoskinson_2022 | 37749.91 | 0 | 26323.47 | 8.5 | 0 | 2981.38 |
| Hoskinson_2022 | 180448.59 | 815.33 | 2295.68 | 904.83 | 19 | 59502.85 |
| Hoskinson_2022 | 101140.69 | 1 | 89145.55 | 1837.17 | 0 | 1328.7 |
| Hoskinson_2022 | 61955.37 | 1013.81 | 60481.86 | 1017.17 | 0.33 | 169.04 |
| Hoskinson_2022 | 91482.46 | 190.47 | 42868.05 | 5119.33 | 0 | 7304.04 |
| Hoskinson_2022 | 76277.23 | 1.25 | 55708.49 | 7.25 | 0.33 | 1613.3 |
| Hoskinson_2022 | 39811.4 | 2 | 28036.15 | 7.75 | 0 | 6962.4 |
| Hoskinson_2022 | 58526.12 | 0.39 | 23294.5 | 5365.75 | 0 | 5424.06 |
| Esposito_2022 | 13487.3 | 6 | 5669.97 | 6 | 0 | 353 |
| Esposito_2022 | 26084.39 | 0 | 10668.24 | 325 | 0 | 1284.75 |
| Esposito_2022 | 20814.3 | 0 | 6641.55 | 3 | 0 | 2039.25 |
| Esposito_2022 | 6269.59 | 0 | 4424.25 | 28 | 0 | 1379.75 |
| Esposito_2022 | 8973.79 | 0 | 5295.05 | 449.6 | 0 | 776.68 |
| Esposito_2022 | 15737.16 | 0 | 7031.06 | 666.67 | 0 | 2252.05 |
| Esposito_2022 | 8196.83 | 0 | 2787.5 | 0 | 0 | 1503.5 |
| Esposito_2022 | 9027.04 | 3 | 2380.97 | 17.03 | 0 | 1491.26 |
| Esposito_2022 | 27952.9 | 0 | 7856.4 | 0 | 0 | 5391.4 |
| Esposito_2022 | 9846.49 | 0 | 3961.85 | 15.5 | 0 | 1465 |
| Esposito_2022 | 6326.5 | 0 | 3056.5 | 228.5 | 0 | 921 |
| Esposito_2022 | 16134.82 | 0 | 6442.58 | 118 | 0 | 1272.7 |
| Esposito_2022 | 26707.75 | 0 | 9092.75 | 213 | 1 | 4146.75 |
| Esposito_2022 | 31587.85 | 0 | 12925.81 | 58 | 265 | 3285 |
| Esposito_2022 | 11049.16 | 0 | 5034.33 | 1267 | 0 | 2263 |
| Esposito_2022 | 19220 | 0 | 5918 | 1 | 0 | 4479.5 |
| Esposito_2022 | 8197.65 | 0 | 3272.08 | 145.17 | 0 | 1295 |
| Esposito_2022 | 45318.46 | 0 | 15328.53 | 1302 | 0 | 4352.5 |
| Esposito_2022 | 20342.1 | 1.67 | 9906.1 | 1226.17 | 0 | 2151.5 |
| Esposito_2022 | 16594.22 | 0 | 7181.76 | 383.7 | 0 | 1298.5 |
| Esposito_2022 | 9792.84 | 0 | 3534.44 | 88.33 | 0 | 1768.83 |
| Esposito_2022 | 14139.66 | 0 | 2936.33 | 0 | 0 | 1127 |
| Esposito_2022 | 61323.46 | 0 | 26294.45 | 10 | 0 | 7921.5 |
| Esposito_2022 | 116257.48 | 0 | 34437.98 | 1730.5 | 0 | 14411.67 |
| Esposito_2022 | 15305.42 | 0.33 | 5667.05 | 594.83 | 0 | 1550 |
| Esposito_2022 | 11928.59 | 0 | 4682.17 | 98 | 0 | 587.67 |
| Esposito_2022 | 12940.92 | 0 | 4281.05 | 298.3 | 0 | 2113.8 |
| Esposito_2022 | 5361.48 | 0 | 2547.81 | 1 | 0 | 195 |
| Esposito_2022 | 18029.69 | 0.67 | 6997.94 | 1064.17 | 0 | 2635 |
| Esposito_2022 | 9723.78 | 0 | 4372.7 | 216.15 | 0 | 2459.7 |
| Esposito_2022 | 20381.37 | 0.33 | 8272.51 | 371.33 | 0 | 2493.6 |
| Esposito_2022 | 16605.18 | 0 | 5902.47 | 1777.47 | 0 | 2104.63 |
| Esposito_2022 | 20907.14 | 0 | 7572.04 | 1819.5 | 0 | 3244.83 |
| Esposito_2022 | 31532.3 | 1 | 12196.89 | 330.7 | 0 | 3408.36 |
| Liu_2023 | 55239.48 | 6.96 | 36440.87 | 3534.42 | 1 | 377.46 |
| Liu_2023 | 22223.01 | 2.62 | 41531.43 | 1646.33 | 2 | 166.64 |
| Liu_2023 | 22917.7 | 5.9 | 38945.55 | 6306 | 3 | 778.87 |
| Liu_2023 | 121619.77 | 5.81 | 18213.52 | 1487.34 | 2 | 272.91 |
| Liu_2023 | 26453.61 | 7.9 | 41050.14 | 3912.29 | 0 | 394.07 |
| Liu_2023 | 25752.52 | 5.76 | 39224.72 | 3949.5 | 6 | 359.23 |
| Liu_2023 | 27671.49 | 3.33 | 41832.07 | 3873.83 | 0 | 354 |
| Liu_2023 | 25564.59 | 7.29 | 39637.05 | 4133.65 | 1 | 357.54 |
| Liu_2023 | 25625.83 | 6.47 | 37668.99 | 3641.13 | 4 | 616.11 |
| Liu_2023 | 28242.61 | 5.53 | 42593.23 | 4197.92 | 6 | 516.99 |
| Liu_2023 | 27284.79 | 11.33 | 41476.52 | 3980.21 | 0 | 475.45 |
| Liu_2023 | 26441.79 | 6.9 | 40794.9 | 4232.64 | 12 | 526.93 |
| Liu_2023 | 25885.66 | 6.33 | 38659.56 | 3757.91 | 1 | 536.27 |
| Liu_2023 | 24436.42 | 9.86 | 37062.24 | 3571.76 | 0 | 415.69 |
| Liu_2023 | 21377.36 | 21.9 | 29252.12 | 1666.55 | 6 | 344.84 |
| Liu_2023 | 27873.97 | 17.14 | 42151.44 | 4054.07 | 6 | 523.07 |
| Liu_2023 | 27181.71 | 14.24 | 41669.48 | 4004.9 | 6 | 490.02 |
| Liu_2023 | 25404.66 | 8.43 | 38565.76 | 3694.12 | 0 | 331.09 |
| Liu_2023 | 79675.81 | 7.53 | 30377.58 | 2685.5 | 5 | 313.36 |
| Liu_2023 | 63237.26 | 0 | 589.26 | 0 | 0 | 588.26 |
| Liu_2023 | 27635.03 | 7.86 | 41094.57 | 4305.57 | 0 | 418.43 |
| Liu_2023 | 28128.37 | 10.29 | 42784.28 | 4142.35 | 4 | 539.67 |
| Liu_2023 | 27370.64 | 4.24 | 40516.46 | 4033.17 | 8 | 488.22 |
| Liu_2023 | 28380.32 | 28.33 | 39737.72 | 3675.49 | 1 | 983.43 |
| Liu_2023 | 68000.3 | 5.14 | 27499.84 | 2557.92 | 4 | 353.95 |
| Liu_2023 | 27294.74 | 24.24 | 39678.42 | 3464.28 | 6 | 1139.32 |
| Liu_2023 | 26235.9 | 10.71 | 40138.44 | 3735.73 | 0 | 394.41 |
| Liu_2023 | 26333.62 | 21.14 | 39435.27 | 3881.04 | 5 | 506.29 |
| Liu_2023 | 19653.57 | 7.67 | 34625.77 | 3499.9 | 0 | 233.6 |
| Liu_2023 | 27843.31 | 7.96 | 42184.83 | 3880.4 | 1 | 478.59 |
| Liu_2023 | 26446.19 | 14.57 | 38798.17 | 4258.15 | 4 | 691.43 |
| Liu_2023 | 28483.12 | 14.24 | 42789.2 | 4354.82 | 1 | 555.57 |
| Liu_2023 | 22737.28 | 6.76 | 40026.22 | 2817.55 | 1 | 360.2 |
| Liu_2023 | 26338.79 | 6.67 | 40178.16 | 4107.25 | 0 | 408.5 |
| Liu_2023 | 130135.62 | 10.62 | 17888.08 | 1625.33 | 2 | 235.47 |
| Liu_2023 | 27098.83 | 5.33 | 41075.32 | 3943.58 | 1 | 333.8 |
| Liu_2023 | 27205.57 | 8.76 | 42353.02 | 4098.91 | 1 | 270.28 |
| Liu_2023 | 24349.39 | 13.14 | 33523.57 | 2896.7 | 19 | 527.97 |
| Liu_2023 | 27048.7 | 8.76 | 41406.54 | 4220.33 | 1 | 321.43 |
| Liu_2023 | 27256.09 | 6.53 | 41922.38 | 4062.09 | 0 | 324.31 |
| Liu_2023 | 66977.7 | 17.04 | 25896.15 | 2534.41 | 8 | 328.05 |
| Liu_2023 | 26987.24 | 9.71 | 40487.09 | 3560.33 | 1 | 351.51 |
| Liu_2023 | 28709.31 | 5.67 | 44484.72 | 4362.05 | 3 | 402.1 |
| Liu_2023 | 25473.32 | 5.96 | 39424.89 | 3991.15 | 1 | 384.29 |
| Liu_2023 | 61596.61 | 20.96 | 29513.88 | 2764.75 | 2 | 347.66 |
| Liu_2023 | 27350.01 | 10.9 | 42045.35 | 4132.47 | 3 | 504.27 |
| Liu_2023 | 26996.8 | 4.96 | 40825.49 | 3779.84 | 5 | 411.14 |
| Liu_2023 | 37468.16 | 3.62 | 28007.02 | 2506.91 | 3 | 329.19 |
| Liu_2023 | 27296.8 | 6.57 | 41624.89 | 4427.98 | 6 | 417.77 |
| Liu_2023 | 25611.81 | 7.57 | 39332.36 | 3621 | 0 | 362.67 |
| Liu_2023 | 26555.08 | 4.71 | 40777.92 | 4097.58 | 1 | 386.41 |
| Liu_2023 | 27237.25 | 5.9 | 42398.21 | 4431.48 | 0 | 367.14 |
| Liu_2023 | 26936.37 | 5.96 | 40723.03 | 3684.42 | 1 | 440.62 |
| Liu_2023 | 27070.08 | 10.33 | 41419.72 | 3925.74 | 2 | 403.03 |
| Liu_2023 | 44263.55 | 5.38 | 37144.79 | 3400 | 0 | 542.33 |
| Liu_2023 | 26806.03 | 6.24 | 40949.41 | 3655.07 | 2 | 355.67 |
| Liu_2023 | 26502.29 | 16.04 | 40221.17 | 4071.04 | 0 | 427.81 |
| Liu_2023 | 54599.69 | 11.76 | 34085 | 3117.08 | 21 | 521.26 |
| Liu_2023 | 81730.49 | 11.29 | 24740.19 | 2548.83 | 0 | 282.65 |
| Liu_2023 | 7289.48 | 28.04 | 73436.36 | 678.67 | 10 | 749.58 |
| Liu_2023 | 16226.07 | 52 | 71923.08 | 759.29 | 211 | 2327.68 |
| Liu_2023 | 6922.2 | 21.96 | 85377.71 | 719.97 | 26 | 956.46 |
| Liu_2023 | 8509.75 | 76.43 | 69373.71 | 801.33 | 27 | 1017.72 |
| Liu_2023 | 4805.52 | 55.81 | 73479.49 | 484.25 | 20 | 621.94 |
| Liu_2023 | 5447.78 | 34.47 | 69873.39 | 508.82 | 25 | 555.52 |
| Liu_2023 | 58679.23 | 154.14 | 36609.09 | 4001.39 | 713 | 10506.75 |
| Liu_2023 | 62311.36 | 57.67 | 34340.21 | 3982.22 | 584 | 10013.71 |
| Liu_2023 | 7028.07 | 17.96 | 60863.88 | 1595.92 | 8 | 646.53 |
| Liu_2023 | 54073.94 | 114.67 | 35040.38 | 4233.29 | 447 | 9864.21 |
| Liu_2023 | 5551.29 | 71.76 | 86184.46 | 620.07 | 17 | 606.13 |
| Kartti_2023 | 34353.53 | 318.59 | 20920.56 | 772.22 | 125.66 | 3998.34 |
| Kartti_2023 | 32191.61 | 397.71 | 21210.88 | 1295.67 | 289.33 | 3541.56 |
| Kartti_2023 | 61354.47 | 886.53 | 36659.34 | 2627.67 | 0 | 5042.73 |
| Kartti_2023 | 38835.7 | 90.75 | 21911.01 | 196.75 | 20 | 3825.99 |
| Kartti_2023 | 29819.16 | 11.95 | 15828.18 | 256.2 | 223 | 4104.3 |
| Kartti_2023 | 32236.78 | 199.21 | 21933.1 | 365.6 | 274.67 | 3837.5 |
| Kartti_2023 | 39426.4 | 15.26 | 21238.44 | 457.74 | 311.33 | 5155.88 |
| Kartti_2023 | 35288.22 | 15.15 | 18503.55 | 357.33 | 231.33 | 4322.08 |
| Kartti_2023 | 42320.64 | 30.33 | 22127.88 | 573.67 | 349 | 5592.47 |
| Kartti_2023 | 38845.37 | 246.69 | 23996.07 | 1864.4 | 46 | 4168.62 |
| Kartti_2023 | 20080.2 | 295.96 | 11810.13 | 525.01 | 371 | 2130.37 |
| Kartti_2023 | 19896.75 | 184.16 | 12651.23 | 300.2 | 99.34 | 2358.35 |
| Kartti_2023 | 35649.82 | 281.2 | 20895.04 | 773.06 | 202.66 | 5536.41 |
| Kartti_2023 | 48483.89 | 189.37 | 29370.15 | 2064.32 | 625 | 6442.09 |
| Kartti_2023 | 17070.37 | 223.64 | 10341.88 | 489.88 | 110.67 | 2384.71 |
| Kartti_2023 | 26585.03 | 410.77 | 16104.6 | 807.36 | 167.33 | 3020.93 |
| Kartti_2023 | 52418.1 | 66.58 | 32388.3 | 806.58 | 0 | 6889.43 |
| Kartti_2023 | 23412.65 | 166.94 | 14292.99 | 478.66 | 136.01 | 3526.51 |
| Kartti_2023 | 17138.23 | 75.91 | 9660.88 | 261.03 | 91.66 | 1641.21 |
| Kartti_2023 | 16766.77 | 150.56 | 9633.11 | 358.92 | 173.67 | 2553.25 |
| Kartti_2023 | 7669.23 | 28.58 | 4097.68 | 97.81 | 0 | 1392.57 |
| Kartti_2023 | 28025.96 | 419.95 | 14393.86 | 1039.57 | 379.67 | 3754.7 |
| Kartti_2023 | 18397.9 | 142.28 | 11136.85 | 449.92 | 167.33 | 1692.95 |
| Kartti_2023 | 35147.52 | 305.14 | 18344.74 | 1287.62 | 181.34 | 5563.87 |
| Kartti_2023 | 29422.71 | 256.11 | 15884.41 | 1104.35 | 438 | 3853.23 |
| Kartti_2023 | 37022.91 | 784.25 | 24472.46 | 1120.75 | 815 | 4929.57 |
| Kartti_2023 | 37908.24 | 217.6 | 24413.33 | 517.5 | 684.33 | 5986.03 |
| Kartti_2023 | 29685.23 | 25 | 12689.14 | 28.33 | 241 | 2437.57 |
| Kartti_2023 | 26587.64 | 206.92 | 17337.4 | 989.38 | 310.34 | 4155.48 |
| Kartti_2023 | 25997.42 | 320.2 | 16542.95 | 562.82 | 139 | 3902.06 |
| Kartti_2023 | 17796.07 | 270.98 | 11520.33 | 234.67 | 220 | 1900.78 |
| Kartti_2023 | 66175.8 | 362.81 | 37825.85 | 839.67 | 119 | 9671.44 |
| Kartti_2023 | 44961.17 | 204.95 | 26865.51 | 924.81 | 2.33 | 6530.83 |
| Kartti_2023 | 16056.61 | 207.62 | 10801.48 | 452.05 | 37 | 2431.3 |
| Kartti_2023 | 24188.32 | 92.77 | 19919.17 | 1894.41 | 151.33 | 3474.01 |
| Kartti_2023 | 20465.27 | 213.77 | 21731.56 | 2744.96 | 27.67 | 1590.87 |
| Kartti_2023 | 28787.64 | 374.93 | 16461.92 | 746.79 | 94.99 | 2426.26 |
| Kartti_2023 | 22733.72 | 253.14 | 14622.28 | 500.41 | 256.34 | 3100.71 |
| Kartti_2023 | 31315.33 | 169.53 | 17076.54 | 1643.53 | 0 | 3694.8 |
| Kartti_2023 | 44221.99 | 268.7 | 27635.77 | 1174.34 | 330.66 | 3805.59 |
| Kartti_2023 | 31626 | 389.1 | 21161.31 | 617.31 | 45 | 3473.59 |
| Kartti_2023 | 37905.41 | 352.06 | 22305.24 | 1109.25 | 345.67 | 4682.66 |
| Kartti_2023 | 38482.44 | 130.81 | 31620.61 | 2493.82 | 65.67 | 5315.19 |
| Kartti_2023 | 40339.66 | 232.82 | 28647.5 | 1967.32 | 407.33 | 6654.13 |
| Kartti_2023 | 19962.65 | 281.33 | 14440.19 | 497.08 | 165.67 | 2720.26 |
| Kartti_2023 | 48146.35 | 39.71 | 31637.53 | 1927 | 0 | 7921.24 |
| Kartti_2023 | 34579.9 | 161.75 | 19853.62 | 706.58 | 324 | 4499.64 |
| Kartti_2023 | 42991.32 | 529.52 | 26467.82 | 1113.17 | 370 | 5081.46 |
| Kartti_2023 | 34978.41 | 324.58 | 28522.37 | 1356.25 | 537.67 | 3250.07 |
| Kartti_2023 | 25188.24 | 441.46 | 14829.21 | 764 | 267 | 2642.38 |
| Kartti_2023 | 94234.85 | 89.5 | 54339.01 | 3903.83 | 620 | 11022.81 |
| German_2023 | 179469.1 | 1 | 108351.09 | 1486.17 | 8849 | 6291.77 |
| German_2023 | 24005.83 | 5.75 | 15973.65 | 269.08 | 658 | 1309.68 |
| German_2023 | 4910.48 | 7.08 | 3908.36 | 60.58 | 204 | 321.5 |
| German_2023 | 15200.75 | 11.5 | 11872.2 | 57 | 402 | 423.95 |
| German_2023 | 265153.18 | 86.67 | 246117.53 | 484.67 | 426 | 1778.4 |
| German_2023 | 18955.5 | 160 | 14252.45 | 754 | 437 | 1428.9 |
| German_2023 | 37802.04 | 8.33 | 35191.78 | 278 | 192 | 372.57 |
| German_2023 | 314035.73 | 207.25 | 297895.89 | 558.92 | 408 | 4399.57 |
| German_2023 | 51732.67 | 1537.5 | 224542.13 | 2306.5 | 75 | 1546.15 |
| German_2023 | 223285.35 | 0 | 182850.35 | 1622.83 | 2156 | 7920.65 |
| German_2023 | 40917.83 | 15.67 | 37546.08 | 86.67 | 225 | 529.75 |
| German_2023 | 14236.94 | 2.37 | 9007.55 | 791.91 | 98 | 1427.13 |
| German_2023 | 8057.79 | 1.25 | 6319.03 | 100.78 | 167 | 277.88 |
| German_2023 | 167375.28 | 44.33 | 161342 | 431.96 | 618 | 1128.33 |
| German_2023 | 4127.59 | 2 | 2949.75 | 45 | 58 | 231.52 |
| German_2023 | 9698.2 | 4 | 5700.28 | 71 | 289 | 164.73 |
| German_2023 | 34155.15 | 11.25 | 24083.36 | 259.36 | 576 | 2253.2 |
| German_2023 | 13092.71 | 4.85 | 10022.79 | 255.71 | 268 | 461.62 |
| German_2023 | 52630.11 | 4 | 21057.19 | 11 | 2786 | 2643 |
| German_2023 | 38015.36 | 60.5 | 21286.17 | 820.5 | 1036 | 3809.38 |
| German_2023 | 60521.56 | 53.04 | 40773.55 | 438.75 | 1116 | 3000.4 |
| German_2023 | 449523.96 | 3.14 | 227656.27 | 88368.17 | 840 | 113491.87 |
| German_2023 | 449309.92 | 0 | 209209.35 | 104022.67 | 979 | 127290.37 |
| German_2023 | 526068.5 | 714.45 | 286511.27 | 877.36 | 4270 | 1993.2 |
| German_2023 | 26047.15 | 24.18 | 22081.45 | 41.33 | 315 | 555 |
| German_2023 | 6407.23 | 18.5 | 3921.72 | 324.5 | 258 | 272.6 |
| German_2023 | 465649.08 | 11637.14 | 296774.18 | 3558 | 1160 | 8415.2 |
| German_2023 | 21486.38 | 2 | 16975.67 | 171 | 110 | 733 |
| German_2023 | 36533.05 | 45.14 | 55466.15 | 161.17 | 373 | 454.21 |
| German_2023 | 57904.01 | 12.5 | 27985.94 | 1013.5 | 526 | 3333.23 |

**Table S9D. The abundance of 6 rate-limiting enzymes linked to the propanoate biosynthesis pathway in BC_adjacent samples.**

| **Samples** | ***acs*** | ***pduW*** | ***ackA*** | ***pct*** | ***acdA*** | ***prpE*** |
| --- | --- | --- | --- | --- | --- | --- |
| Hoskinson_2022 | 15102.04 | 0 | 34017.86 | 2310.25 | 0 | 3443.88 |
| Hoskinson_2022 | 35784.8 | 0.67 | 53635.63 | 9441.17 | 0 | 5356.3 |
| Hoskinson_2022 | 81254.25 | 0 | 32369.34 | 17485.5 | 0 | 274.6 |
| Hoskinson_2022 | 23707.25 | 305.62 | 63886.75 | 7952.12 | 2782 | 1157.5 |
| Hoskinson_2022 | 114698.62 | 0.47 | 59521.39 | 17340.33 | 0 | 939.34 |
| Hoskinson_2022 | 114679.69 | 0 | 46568.81 | 5.25 | 0.33 | 209.7 |
| Hoskinson_2022 | 70569 | 2 | 43036.86 | 4444.75 | 0 | 7095.3 |
| Hoskinson_2022 | 97274.79 | 0.14 | 50693.59 | 1869 | 0.67 | 14637.14 |
| Hoskinson_2022 | 130321.96 | 1 | 62756.72 | 7132.75 | 0 | 15641.83 |
| Hoskinson_2022 | 83319.35 | 0.25 | 63318.85 | 2.25 | 0 | 1338.4 |
| Hoskinson_2022 | 21124.15 | 0 | 85025.4 | 570 | 0 | 1315.4 |
| Hoskinson_2022 | 95933.75 | 0.58 | 50549.95 | 6466 | 0 | 6909.28 |
| Hoskinson_2022 | 105195.13 | 0.25 | 52982.38 | 12833 | 1 | 13878.53 |
| Hoskinson_2022 | 66583.37 | 0.29 | 43405.2 | 3.25 | 0 | 7458.41 |
| Hoskinson_2022 | 111394.84 | 0.14 | 36773.04 | 8472.39 | 0 | 27571.13 |
| Hoskinson_2022 | 103414.31 | 0 | 58323.18 | 3202.5 | 0 | 5845.6 |
| Hoskinson_2022 | 111320.73 | 0 | 56562.56 | 248 | 0 | 4964 |
| Hoskinson_2022 | 21474.5 | 15074.33 | 19348.17 | 16724.33 | 0 | 370 |
| Hoskinson_2022 | 110094.26 | 1 | 47464.01 | 293.5 | 0 | 7337.22 |
| Hoskinson_2022 | 60141.38 | 723.61 | 43263.49 | 1702.84 | 0 | 7072.99 |
| Hoskinson_2022 | 42414.27 | 1927.33 | 27235.64 | 1999.87 | 0 | 2241.05 |
| Hoskinson_2022 | 58923 | 0 | 73356.73 | 14251.08 | 0 | 10020.57 |
| Hoskinson_2022 | 144335.31 | 0.14 | 75729.05 | 3474.22 | 0 | 4247.1 |
| Hoskinson_2022 | 94135.51 | 20.12 | 48561.06 | 12141.33 | 0 | 22762.4 |
| Hoskinson_2022 | 205196.53 | 0 | 129974.53 | 0.5 | 0 | 1732.2 |
| Hoskinson_2022 | 131701.96 | 1.67 | 96028.63 | 8.92 | 0 | 7601.06 |
| Hoskinson_2022 | 83999.3 | 4215.28 | 53289.95 | 12073.89 | 0 | 13888.54 |
| Hoskinson_2022 | 45649.05 | 1.67 | 28001.24 | 3.67 | 2 | 94.3 |
| Hoskinson_2022 | 84119.73 | 3.14 | 35835.57 | 10109.93 | 0 | 15172.64 |
| Hoskinson_2022 | 37929.23 | 0.33 | 48423.52 | 19479.83 | 1 | 12566.4 |
| Hoskinson_2022 | 22060.12 | 6.9 | 41599.38 | 9.4 | 0 | 2368.5 |
| Hoskinson_2022 | 81411.13 | 1.47 | 38967.8 | 2127.33 | 0 | 17367.54 |
| Hoskinson_2022 | 62196.41 | 0.14 | 37124.11 | 15086.14 | 0 | 53.1 |
| Hoskinson_2022 | 101932.39 | 1 | 40868.06 | 16272.5 | 0 | 6336 |
| Hoskinson_2022 | 79602.91 | 1122.81 | 40477.25 | 26363.31 | 0 | 228.2 |
| Hoskinson_2022 | 88157.6 | 0 | 37020.77 | 1942.75 | 0 | 16140.6 |
| Hoskinson_2022 | 40564.61 | 4.54 | 24603.61 | 744 | 0.33 | 5793.34 |
| Hoskinson_2022 | 71865.02 | 0.33 | 42420.28 | 1555.41 | 0 | 5830.53 |
| Hoskinson_2022 | 29992.08 | 0.33 | 38409.68 | 143.33 | 0 | 22.25 |
| Hoskinson_2022 | 64099.84 | 0.58 | 25028.16 | 6452.41 | 0.33 | 16721.58 |
| Hoskinson_2022 | 91914.19 | 0 | 38469.78 | 19044 | 0 | 11466.45 |
| Hoskinson_2022 | 105198.85 | 0 | 45737.11 | 18.5 | 0 | 10547.6 |
| Hoskinson_2022 | 57498.86 | 0.96 | 33134.84 | 386.17 | 0 | 4604.29 |
| Hoskinson_2022 | 94985.59 | 0 | 38855.92 | 13.83 | 0 | 3337.7 |
| Hoskinson_2022 | 52955.21 | 0.33 | 28564.94 | 807.16 | 1.33 | 5431.75 |
| Hoskinson_2022 | 104397.53 | 0.58 | 58256.94 | 1091.58 | 0 | 2259.73 |
| Hoskinson_2022 | 26749.46 | 0 | 41684.58 | 24 | 1 | 673.8 |
| Hoskinson_2022 | 12494.85 | 11341.9 | 22204.77 | 11411.25 | 0 | 54.77 |
| Hoskinson_2022 | 67522.15 | 0 | 50276.22 | 10475.5 | 0 | 17574.17 |
| Esposito_2022 | 15589.58 | 0 | 5588.75 | 1.5 | 0 | 243.67 |
| Esposito_2022 | 13383.83 | 0 | 3604.44 | 355.5 | 0 | 1193.93 |
| Esposito_2022 | 1875.5 | 0 | 505.7 | 28.5 | 0 | 192 |
| Esposito_2022 | 12051.48 | 0 | 2236.24 | 277 | 0 | 879.4 |
| Esposito_2022 | 3213.82 | 0 | 1169.66 | 73 | 0 | 410.67 |
| Esposito_2022 | 134350.31 | 0 | 66792.32 | 6760.99 | 0 | 15289.4 |
| Esposito_2022 | 5960.66 | 0 | 2597.08 | 0.67 | 0 | 47.83 |
| Esposito_2022 | 8199.33 | 0 | 1895.14 | 75.33 | 0 | 689.96 |
| Esposito_2022 | 3779.32 | 0 | 1365.31 | 159.5 | 0.33 | 436.3 |
| Esposito_2022 | 3566.05 | 0 | 896.96 | 179 | 0 | 424.97 |
| Esposito_2022 | 3418.21 | 0 | 896.98 | 117 | 0 | 407.7 |
| Esposito_2022 | 40348.86 | 0 | 10565.42 | 972.4 | 0 | 2987.05 |
| Esposito_2022 | 13344.61 | 0 | 779.61 | 35.5 | 0 | 175.5 |
| Esposito_2022 | 30977.14 | 0 | 14521.57 | 314.5 | 0 | 2053.53 |
| Esposito_2022 | 11116.29 | 0 | 3208.84 | 142.5 | 0 | 1314.57 |
| Esposito_2022 | 5189.7 | 0 | 1691.47 | 311.5 | 0 | 718.4 |
| Esposito_2022 | 43668.75 | 0 | 11477.3 | 199.03 | 0 | 2201.83 |
| Esposito_2022 | 20057.75 | 0 | 7781.25 | 61.5 | 0 | 2044 |
| Esposito_2022 | 10151.17 | 0 | 2590.38 | 749 | 0 | 1725.33 |
| Esposito_2022 | 12052.6 | 1 | 2619.82 | 486.33 | 0 | 750.26 |
| Esposito_2022 | 3909.84 | 0 | 845.49 | 130.5 | 0 | 305.03 |
| Esposito_2022 | 1277.56 | 0 | 227.23 | 25.5 | 0 | 47.9 |
| Esposito_2022 | 16889.7 | 0 | 6261.2 | 239 | 0 | 2991.2 |
| Esposito_2022 | 19700.5 | 0 | 17665.91 | 130 | 0 | 3170.5 |
| Esposito_2022 | 24407.93 | 1 | 11940.85 | 448.67 | 3 | 3303.96 |
| Esposito_2022 | 6304.63 | 0 | 1572.47 | 129 | 0 | 403.3 |
| Esposito_2022 | 2709.87 | 0 | 679.26 | 67.5 | 0 | 224.2 |
| Esposito_2022 | 4220.66 | 0 | 1316.61 | 88.28 | 0 | 499.7 |
| Esposito_2022 | 6426.94 | 0 | 2283.06 | 330.12 | 0.33 | 596.92 |
| Esposito_2022 | 5434.43 | 0 | 1409.7 | 109.1 | 0 | 385.2 |
| Esposito_2022 | 23079.87 | 5 | 7896.06 | 653 | 0 | 2805.04 |
| Esposito_2022 | 9805.45 | 0 | 1030.29 | 173.5 | 0 | 271.67 |
| Esposito_2022 | 50254.85 | 0 | 14261.17 | 1303 | 0 | 4373.33 |
| Esposito_2022 | 8949.22 | 0 | 1954.9 | 209.5 | 0 | 634.56 |
| Kartti_2023 | 14283.26 | 101.08 | 8365 | 250.48 | 216.33 | 1300.88 |
| Kartti_2023 | 19586.71 | 158.74 | 10535.54 | 374.34 | 221.33 | 2141.7 |
| Kartti_2023 | 17114.65 | 313.07 | 10589.5 | 440.87 | 191.66 | 2068.64 |
| Kartti_2023 | 26351.37 | 328.85 | 16334.1 | 558.52 | 176.49 | 3421.01 |
| Kartti_2023 | 15471.81 | 191.79 | 9644.98 | 526.27 | 92.01 | 1997.57 |
| Kartti_2023 | 39971.47 | 173.3 | 23116.01 | 570.55 | 653.67 | 4921.32 |
| Kartti_2023 | 23225.68 | 191.29 | 13069.6 | 271.65 | 350.66 | 2763.49 |
| Kartti_2023 | 17817.96 | 159.04 | 10070.37 | 290.01 | 153.33 | 2540.83 |
| Kartti_2023 | 15370.92 | 250.5 | 12898.64 | 416.11 | 197.33 | 1710.11 |
| Kartti_2023 | 26708.09 | 300.25 | 15991.24 | 756.47 | 309.33 | 3276.43 |
| Kartti_2023 | 28680.51 | 223.48 | 16692.52 | 485.71 | 276.67 | 3471.46 |
| Kartti_2023 | 38696.53 | 141.82 | 21662.81 | 376.25 | 434 | 4862.34 |
| Kartti_2023 | 30539.64 | 230.8 | 16685.91 | 881.85 | 536.67 | 4892.39 |
| Kartti_2023 | 25988.78 | 276.32 | 16094.91 | 1128.77 | 271 | 3372.69 |
| Kartti_2023 | 23182.7 | 253.32 | 11948.83 | 339.34 | 225 | 3062.78 |
| Kartti_2023 | 19886.31 | 264.98 | 12145.32 | 585.51 | 272.99 | 2217.55 |
| Kartti_2023 | 22639.18 | 345.98 | 13754.32 | 634.64 | 307.01 | 3095.27 |
| Kartti_2023 | 16320.19 | 211.87 | 10366.94 | 484.43 | 127.99 | 1956.42 |
| Kartti_2023 | 28868.65 | 418.38 | 16827.62 | 1088.05 | 280.34 | 2922.72 |
| Kartti_2023 | 33455.6 | 216.47 | 19209.64 | 352.33 | 12 | 3418.41 |
| Kartti_2023 | 32138.39 | 410.61 | 20722.69 | 783.73 | 115.33 | 3540.9 |
| Kartti_2023 | 53038.53 | 489.51 | 30128.65 | 1859.72 | 553 | 5186.17 |
| Kartti_2023 | 44078.31 | 373.32 | 25422.3 | 1012.61 | 87.67 | 5260.35 |
| Kartti_2023 | 43942.21 | 358.05 | 25073.25 | 935.53 | 21.34 | 5210.13 |
| Kartti_2023 | 81707.49 | 1017.47 | 60100.02 | 4899.31 | 698 | 3656.41 |
| Kartti_2023 | 34112.5 | 241.48 | 18273.85 | 549.22 | 98 | 4165.53 |
| Kartti_2023 | 34547.41 | 24.57 | 17406.58 | 174.66 | 252.67 | 4743.35 |
| Kartti_2023 | 36388.82 | 562.37 | 31738.66 | 1954.07 | 537.34 | 3757.69 |
| Kartti_2023 | 29665.33 | 400.02 | 19420.66 | 703.01 | 146.67 | 3346.6 |
| Kartti_2023 | 18734.42 | 0.25 | 9546.3 | 221.5 | 181.67 | 2427.48 |
| Kartti_2023 | 29401.54 | 87.02 | 16332.12 | 632.42 | 34 | 3432.7 |
| Kartti_2023 | 23382.11 | 221.55 | 14876.58 | 779.64 | 139.67 | 3085.89 |
| Kartti_2023 | 23545.03 | 156.8 | 13395.63 | 493.56 | 401.67 | 3258.45 |
| Kartti_2023 | 38680.84 | 3.89 | 19978.34 | 321.8 | 216 | 6339.53 |
| Kartti_2023 | 26495.12 | 113.4 | 16081.41 | 781.9 | 292 | 2731.46 |
| Kartti_2023 | 16956.78 | 125.4 | 12355.7 | 272.33 | 51.67 | 1755.34 |
| Kartti_2023 | 42321.69 | 591.15 | 32927.64 | 507.62 | 169 | 3384.67 |
| Kartti_2023 | 38766.59 | 500.7 | 23536.46 | 841.37 | 118 | 4251.76 |
| Kartti_2023 | 13973.42 | 163.6 | 7719.5 | 353.78 | 143 | 1873.21 |
| Kartti_2023 | 48993.7 | 22 | 27137.26 | 887.67 | 0 | 7585.37 |
| Kartti_2023 | 40842.9 | 73.88 | 32696.54 | 3202.1 | 144.67 | 5727.11 |
| Kartti_2023 | 56102.96 | 189.65 | 30188.86 | 839.8 | 424.67 | 5179.28 |
| Kartti_2023 | 27360.11 | 363.18 | 15742.95 | 815.96 | 130.66 | 3207.68 |
| Kartti_2023 | 78878.72 | 132.1 | 50675.75 | 1058 | 386.33 | 10169.71 |
| Kartti_2023 | 23863.93 | 258.95 | 15047.81 | 643.45 | 161.33 | 3316.14 |
| Kartti_2023 | 44685.14 | 5.67 | 42982.65 | 3923.67 | 3.34 | 3552.2 |
| Kartti_2023 | 54612.03 | 39.46 | 30828.35 | 887.08 | 0 | 7197.74 |
| Kartti_2023 | 57064.65 | 384.29 | 28739.75 | 919.6 | 231.67 | 4816.58 |
| Kartti_2023 | 41619.59 | 210.58 | 33826.33 | 2062.58 | 85 | 6461.48 |
| Kartti_2023 | 39663.95 | 280.42 | 25645.86 | 903.75 | 86 | 1793.3 |
| Kartti_2023 | 31808.9 | 24.25 | 39646.68 | 361.58 | 9.33 | 5589.31 |
| Kartti_2023 | 32472.74 | 3 | 16534.55 | 363.15 | 203 | 4295.73 |
| German_2023 | 199552.8 | 0 | 66561.64 | 11551.03 | 8859 | 25405.86 |
| German_2023 | 163754.64 | 1 | 127580.78 | 1763 | 2073 | 4785 |
| German_2023 | 447532.65 | 0.25 | 211003.24 | 7980.08 | 8787 | 24467.18 |
| German_2023 | 290921.34 | 0 | 272227.99 | 1675.17 | 2823 | 2396.07 |
| German_2023 | 17509.08 | 11 | 10169.86 | 119 | 298 | 559.26 |
| German_2023 | 28605.06 | 0 | 28750.71 | 175.5 | 360 | 442.4 |
| German_2023 | 117360.12 | 1 | 81683.64 | 812 | 3939 | 5421.7 |
| German_2023 | 130797.78 | 145.25 | 120962.86 | 315.42 | 548 | 1138.22 |
| German_2023 | 45288.81 | 2 | 29752.03 | 183.33 | 1870 | 1959.43 |
| German_2023 | 61568.23 | 0 | 57027.46 | 340.5 | 477 | 863.4 |
| German_2023 | 161840.39 | 40.58 | 137480.82 | 851.94 | 323 | 2649.23 |
| German_2023 | 75709.41 | 0 | 73270.33 | 197.53 | 205 | 850.69 |
| German_2023 | 72652.54 | 0 | 70632 | 552.33 | 216.33 | 788.93 |
| German_2023 | 37399.55 | 233.83 | 33389.89 | 41.83 | 143 | 691.4 |
| German_2023 | 600064.94 | 0 | 274812.4 | 20072.83 | 611 | 142653.49 |
| German_2023 | 85125.66 | 35.08 | 78171.08 | 187.83 | 510 | 1726.75 |
| German_2023 | 218522.24 | 9 | 175025.07 | 1450.5 | 1802 | 3182 |
| German_2023 | 412630.22 | 80.5 | 363817.98 | 9800.75 | 3426 | 12589.07 |
| German_2023 | 259052.25 | 83.25 | 247444.76 | 488.25 | 975 | 2068.73 |
| German_2023 | 211467.19 | 52.75 | 174590.92 | 1305.25 | 4878 | 1385.4 |
| German_2023 | 114135.5 | 17.33 | 11327.42 | 453.33 | 0 | 9181.1 |
| German_2023 | 114081.9 | 3 | 98597.04 | 105.83 | 1285 | 765.16 |
| German_2023 | 160117.16 | 107.1 | 86515.69 | 16774.58 | 12235 | 25907.25 |
| German_2023 | 9422.69 | 7 | 5369.86 | 56 | 149 | 689.6 |
| German_2023 | 5937.98 | 2.25 | 3905.65 | 36.25 | 42 | 355.17 |
| German_2023 | 92865.66 | 35.5 | 48163.25 | 1156 | 1468 | 9249.65 |
| German_2023 | 91904.4 | 265.47 | 82090.76 | 421.83 | 832 | 1796.67 |
| German_2023 | 94538.22 | 1 | 143602.92 | 2869 | 3113 | 5556.6 |
| German_2023 | 51034.08 | 2 | 47513.64 | 330.83 | 321 | 821.83 |
| German_2023 | 409761.04 | 2.25 | 321079.51 | 5069.45 | 11861 | 13378.2 |
| German_2023 | 191629.51 | 1.25 | 171483.19 | 4.58 | 3019 | 628.93 |
| German_2023 | 294234.03 | 0.25 | 235782.55 | 3629.2 | 3328 | 13957.17 |
| German_2023 | 126513.04 | 41.17 | 102534.19 | 1255.17 | 325 | 5079.34 |
| German_2023 | 118799.11 | 359.43 | 94882.36 | 1702.9 | 1269 | 4749.73 |
| German_2023 | 7214.22 | 9.87 | 5121.32 | 37.58 | 65 | 490.01 |
| German_2023 | 485508.83 | 8 | 435583.31 | 2868.5 | 2637 | 12644.72 |
| German_2023 | 43291.53 | 4.25 | 36970.26 | 890.32 | 387 | 1233.22 |
| German_2023 | 465582.97 | 29998.5 | 308028.23 | 1145.5 | 1297 | 15973.27 |
| German_2023 | 190932.85 | 103.42 | 181426.01 | 119.17 | 806 | 3335.61 |
| German_2023 | 243714.3 | 0.64 | 152990.4 | 3635.5 | 5261 | 15893.24 |
| German_2023 | 166387.86 | 0.25 | 119848.62 | 815.33 | 3624 | 9497.71 |
| German_2023 | 211392.25 | 124.04 | 130356 | 33141.33 | 751 | 43252.04 |
| German_2023 | 341886.29 | 8.75 | 190129.64 | 66236.58 | 692 | 82324.06 |
| German_2023 | 91010.8 | 7.33 | 82006.17 | 335.83 | 353 | 2371.63 |
| German_2023 | 92101.64 | 0.25 | 80229.45 | 501.5 | 175 | 1725.85 |
| German_2023 | 50035 | 49.33 | 40179.32 | 294 | 240 | 4100.19 |
| German_2023 | 69738.21 | 2233 | 39275.59 | 552.11 | 1474 | 6027.87 |
| German_2023 | 10027.79 | 0 | 4416.56 | 77 | 88 | 541.4 |
| German_2023 | 409164.24 | 0 | 188008.37 | 92175.5 | 990 | 119823.7 |
| German_2023 | 423708.04 | 127.67 | 191471.57 | 101013.84 | 256 | 123166.77 |
| German_2023 | 92050.33 | 107.86 | 43214.23 | 11923.33 | 900 | 19384.56 |
| German_2023 | 41694.39 | 182.39 | 29670.49 | 65.45 | 656 | 1467.9 |
| German_2023 | 79120.76 | 0.57 | 67857.94 | 134.5 | 985 | 1277.3 |
| German_2023 | 68248.84 | 139.25 | 52578.33 | 1015 | 1613 | 3005.73 |
| German_2023 | 310434.85 | 2 | 142184.77 | 70942.17 | 914 | 91888.53 |
| German_2023 | 113986.74 | 0 | 76591.49 | 234.5 | 2610 | 2875.57 |
| German_2023 | 172425.23 | 0 | 82903.74 | 32134.67 | 371 | 45157.94 |
| German_2023 | 478936.47 | 145.1 | 482074.02 | 1516.67 | 2082 | 5444.28 |
| German_2023 | 12854.58 | 13 | 17272 | 120 | 121 | 1287.82 |
| German_2023 | 139104.2 | 2.9 | 118766.36 | 464.83 | 3299 | 1839.53 |

**Table S9E. The abundance of 6 rate-limiting enzymes linked to the propanoate biosynthesis pathway in normal_tissue samples.**

| **Samples** | ***acs*** | ***pduW*** | ***ackA*** | ***pct*** | ***acdA*** | ***prpE*** |
| --- | --- | --- | --- | --- | --- | --- |
| Hoskinson_2022 | 22367.57 | 5.39 | 45444.47 | 4747.75 | 256.67 | 2080.34 |
| Hoskinson_2022 | 80350.5 | 2516.43 | 33888.92 | 6124.54 | 1 | 23628.84 |
| Hoskinson_2022 | 21841.27 | 148.57 | 42134.19 | 1295.25 | 402 | 5181.62 |
| Hoskinson_2022 | 57121.94 | 0 | 22977.94 | 41.75 | 0 | 15266.65 |
| Hoskinson_2022 | 42789.48 | 0 | 24949.84 | 0 | 0 | 25.01 |
| Hoskinson_2022 | 64099.6 | 0 | 39875.26 | 1.5 | 0 | 365 |
| Hoskinson_2022 | 55229.68 | 0 | 22394 | 763 | 0 | 1933 |
| Hoskinson_2022 | 126259.87 | 0 | 84509.52 | 17744.75 | 0.33 | 37403.83 |
| Hoskinson_2022 | 34326.43 | 0 | 18713.8 | 0 | 0 | 103.28 |
| Hoskinson_2022 | 66007.57 | 1.81 | 37174.05 | 390.42 | 0 | 4141.14 |
| Hoskinson_2022 | 72492.36 | 1.25 | 48107.17 | 4023.25 | 0 | 8255.15 |
| Hoskinson_2022 | 101472.19 | 1 | 55883.36 | 11504.25 | 0 | 11522.2 |
| Hoskinson_2022 | 71713.9 | 0.33 | 48979.13 | 5379.33 | 0 | 5502.87 |
| Hoskinson_2022 | 32055.06 | 0.33 | 8422.19 | 1.58 | 375.33 | 3366.97 |
| Hoskinson_2022 | 90303.03 | 0.14 | 39760.76 | 9010.25 | 0 | 16995.34 |
| Hoskinson_2022 | 103533.75 | 4.5 | 50154.04 | 17465.75 | 2.67 | 19644.2 |
| Hoskinson_2022 | 23154.39 | 0 | 29948.2 | 3461 | 0 | 4163 |
| Hoskinson_2022 | 150173.32 | 0 | 77302.6 | 17987.75 | 0 | 22214 |
| Hoskinson_2022 | 103746.95 | 0 | 56685.93 | 7955.75 | 0 | 9114.85 |
| Hoskinson_2022 | 112259.72 | 0 | 58467.38 | 4453 | 0 | 5645.33 |
| Hoskinson_2022 | 108495.64 | 1050.33 | 49945.38 | 10808.33 | 0 | 22068 |
| Hoskinson_2022 | 137477.83 | 0 | 71180.21 | 15325 | 0.33 | 21017.25 |
| Hoskinson_2022 | 99652.65 | 0 | 49204.2 | 3.5 | 0.67 | 13989 |
| Hoskinson_2022 | 86355.12 | 0 | 33504.2 | 1061.5 | 0 | 11341.83 |
| Hoskinson_2022 | 88886.71 | 421.58 | 53565.68 | 4936.08 | 0 | 13219.92 |
| Hoskinson_2022 | 84097.16 | 0.25 | 48055.77 | 10913.25 | 0 | 10924.5 |
| Hoskinson_2022 | 56147.19 | 0.33 | 34869.5 | 9332.83 | 0 | 17813.83 |
| Hoskinson_2022 | 68395.53 | 0 | 51956.62 | 12226 | 0 | 13420 |
| Hoskinson_2022 | 125192.88 | 0 | 58307.11 | 14056 | 0 | 21127.96 |
| Hoskinson_2022 | 148974.3 | 0 | 51494.3 | 11577.5 | 0 | 21214.63 |
| Hoskinson_2022 | 53790.39 | 0 | 21150.83 | 2139 | 0.33 | 3833.72 |
| Hoskinson_2022 | 43096.01 | 0 | 29281.52 | 29 | 0 | 2.6 |
| Hoskinson_2022 | 58332.62 | 0 | 25127.57 | 0 | 0 | 580 |
| Hoskinson_2022 | 87888.78 | 0 | 52914.11 | 4.5 | 0 | 2 |
| Hoskinson_2022 | 84800.55 | 0 | 42968.59 | 5 | 0 | 1152.1 |
| Hoskinson_2022 | 79929.27 | 0 | 43429.25 | 42 | 0 | 1827.5 |
| Hoskinson_2022 | 100289.12 | 0.14 | 45697.78 | 1659.5 | 0 | 3841.14 |
| Hoskinson_2022 | 103681.8 | 0 | 53283.89 | 2 | 0 | 32 |
| Hoskinson_2022 | 75996.27 | 0 | 55875.52 | 0 | 0 | 3177.33 |
| Hoskinson_2022 | 79499.41 | 121.67 | 45693.54 | 121.67 | 0 | 1241 |
| Hoskinson_2022 | 102025.74 | 0 | 45645.86 | 0 | 0 | 1594 |
| Hoskinson_2022 | 79020.99 | 0.14 | 50165.98 | 1 | 0 | 5747.14 |
| Hoskinson_2022 | 45253.89 | 0 | 21233.22 | 5.5 | 0 | 589.8 |
| Hoskinson_2022 | 41509.44 | 0 | 29435.44 | 3 | 0 | 4473.1 |
| Hoskinson_2022 | 43445.14 | 0 | 27181.23 | 1 | 0 | 45.81 |
| Hoskinson_2022 | 46849.76 | 0 | 24543.95 | 1.5 | 0 | 60.55 |
| Hoskinson_2022 | 101635.81 | 513.28 | 57708.65 | 513.14 | 0 | 9380.14 |
| Hoskinson_2022 | 61658.98 | 0.33 | 50481.64 | 2332.33 | 0 | 287.45 |
| Hoskinson_2022 | 63261.66 | 0.33 | 46351.31 | 7802.33 | 0 | 9026.82 |
| German_2023 | 153707.07 | 85.5 | 220088.78 | 3897 | 183 | 48234.65 |
| German_2023 | 69825.43 | 54 | 100670.16 | 1308.4 | 240 | 29540.37 |
| German_2023 | 118879.87 | 20 | 188114.07 | 2326.71 | 366 | 47562.45 |
| German_2023 | 41398.79 | 2.42 | 38513.99 | 172.72 | 210 | 247.35 |
| German_2023 | 16768.26 | 2.25 | 14382.07 | 301.32 | 444 | 600.75 |
| German_2023 | 186880.94 | 145.53 | 239069.17 | 1733.77 | 416.67 | 47270.81 |
| German_2023 | 211368.39 | 48.25 | 365827.49 | 3919.65 | 914 | 89344.1 |
| German_2023 | 216291.37 | 26 | 304942.22 | 2770.75 | 605 | 78134.16 |
| German_2023 | 276101.16 | 71.92 | 339677.24 | 2625.82 | 498 | 59704.79 |
| German_2023 | 179028.14 | 24.25 | 190389.91 | 1629.13 | 245 | 60271.47 |
| German_2023 | 86877.03 | 246.5 | 74234.75 | 384.33 | 256 | 2632.37 |
| German_2023 | 162539.65 | 20.5 | 208803.15 | 3271.5 | 429 | 48520.47 |
| German_2023 | 185050.64 | 28.47 | 181629.28 | 802.66 | 306 | 1562.57 |
| German_2023 | 38409.5 | 12.33 | 30444.8 | 96.46 | 229 | 680.63 |
| German_2023 | 71012.01 | 8 | 69144.42 | 108 | 168 | 486.21 |
| German_2023 | 184095.15 | 71 | 250205.75 | 1925 | 513 | 50678.93 |
| German_2023 | 82127.25 | 8.33 | 122716.19 | 917.93 | 190 | 25692.2 |
| German_2023 | 135983.99 | 0 | 188510.3 | 4188.76 | 1427 | 53356.1 |
| German_2023 | 22841.36 | 5.33 | 16147.57 | 339.93 | 344 | 1708.24 |
| German_2023 | 55266.79 | 37 | 60977.12 | 344 | 139 | 12020.68 |
| German_2023 | 79755.51 | 5.14 | 108223.81 | 1482.54 | 261 | 25916.51 |
| German_2023 | 51667.69 | 2.75 | 66817.17 | 412.9 | 77 | 8716.6 |
| German_2023 | 35304.01 | 0 | 26502.58 | 41.67 | 189 | 1103.87 |
| German_2023 | 46149.87 | 3 | 44201.08 | 145 | 150 | 472.15 |
| German_2023 | 96809.34 | 0 | 123827.91 | 1726.22 | 127 | 30437.15 |
| German_2023 | 207712.68 | 36.5 | 301038.86 | 3564.35 | 703 | 52236.75 |
| German_2023 | 304194.09 | 0 | 192435.36 | 38961.57 | 3200 | 49898.89 |
| German_2023 | 182201.82 | 29.58 | 253254.37 | 2803.58 | 415 | 60100.8 |
| German_2023 | 226854.83 | 0 | 309472 | 5032 | 3131 | 78716.3 |
| German_2023 | 84112.13 | 83.75 | 106145.59 | 1287.5 | 276 | 22819.19 |
| German_2023 | 114399.06 | 29.08 | 135267.79 | 712.78 | 105 | 14954.33 |
| German_2023 | 74195.27 | 48.17 | 98738.46 | 1130.17 | 490 | 30474.8 |
| German_2023 | 162583.82 | 0 | 147763.69 | 825 | 485 | 3529.9 |
| German_2023 | 74481.01 | 34 | 64548.6 | 589.1 | 156 | 1681.33 |
| German_2023 | 725240.56 | 0 | 708756.14 | 1172 | 1481 | 2264.25 |
| German_2023 | 15520.21 | 2.25 | 12764.23 | 287.75 | 0 | 588.67 |
| German_2023 | 74591.25 | 0.25 | 41074.05 | 530.75 | 303 | 10268.78 |
| German_2023 | 127062.41 | 37.83 | 125972.57 | 57.83 | 107 | 248.05 |
| German_2023 | 164962.31 | 102.42 | 124854.13 | 151.62 | 443 | 2754.6 |
| German_2023 | 5292.33 | 0.25 | 4607.17 | 160.25 | 0 | 351.5 |
| German_2023 | 129722.16 | 500 | 114087.01 | 1084 | 2069 | 2280 |
| German_2023 | 227058.47 | 153 | 276369.75 | 2057.56 | 200 | 52150.23 |
| German_2023 | 183350.09 | 35.58 | 285558.14 | 3426.98 | 219 | 70199.12 |
| German_2023 | 182942.37 | 0 | 178958.64 | 96 | 220 | 374.8 |
| German_2023 | 10695.56 | 0 | 10367.1 | 16 | 19 | 318.95 |
| German_2023 | 284939.77 | 4.75 | 283054.09 | 331.25 | 0 | 646.7 |
| German_2023 | 8491.72 | 0.25 | 7747.17 | 148.5 | 4 | 449.2 |
| German_2023 | 100802.11 | 6.88 | 95627.78 | 77.88 | 404 | 484.78 |
| German_2023 | 278921.3 | 7.25 | 195671.13 | 1075.65 | 79 | 5196.24 |
| German_2023 | 27179 | 8 | 26239.82 | 46 | 89 | 204.28 |
| German_2023 | 154878.4 | 518.6 | 233460.79 | 2736.18 | 297 | 61336.44 |
| German_2023 | 130713.68 | 0 | 109616.85 | 1827 | 4096 | 1344.9 |
| German_2023 | 335122.2 | 2 | 320060.2 | 379 | 1957 | 1936.37 |
| German_2023 | 184718.01 | 0 | 181966.87 | 106 | 164 | 247.9 |
| German_2023 | 111699.85 | 3.33 | 100000.61 | 336.83 | 335 | 2257.65 |
| German_2023 | 11326.87 | 2.47 | 10916.15 | 56.83 | 0 | 236.04 |
| German_2023 | 138532 | 1.25 | 117754.34 | 620.45 | 2293 | 1311.1 |
| German_2023 | 20843.24 | 24 | 18184.79 | 84.6 | 109 | 380.9 |
| German_2023 | 59473.27 | 5 | 45193.48 | 372.5 | 757 | 843.2 |
| German_2023 | 154462.45 | 4.75 | 152256.8 | 90.95 | 151 | 589.85 |
| German_2023 | 307464.34 | 2.25 | 286018.41 | 122.25 | 1346 | 1578.92 |
| German_2023 | 153308.95 | 537.75 | 118866.59 | 1365.55 | 1126 | 11793.9 |
| German_2023 | 271346.16 | 1.25 | 165721.66 | 1475.95 | 5816 | 6864 |
| German_2023 | 412774.32 | 501 | 346962.84 | 1685 | 3655 | 15658.13 |
| German_2023 | 59352.94 | 1.25 | 37775.36 | 1819.53 | 1004 | 6178.34 |
| German_2023 | 127491.78 | 0 | 99305 | 1900.2 | 445 | 6158.61 |
| German_2023 | 355682.32 | 884.75 | 243692.68 | 5948.28 | 8592 | 14074.43 |
| German_2023 | 101990.53 | 1361.5 | 66144.57 | 1104.1 | 2457 | 5412.1 |
| German_2023 | 174553.97 | 269 | 164187.48 | 617 | 1624 | 1143.15 |
| German_2023 | 244884.27 | 0 | 100428.68 | 4815.3 | 5859 | 19824.83 |
| German_2023 | 195071.59 | 1059.31 | 202726.82 | 8370.73 | 2 | 13154.46 |
| German_2023 | 156992.22 | 0.67 | 97090.23 | 1627 | 1419 | 9100.8 |
| German_2023 | 206947.27 | 1742.5 | 162697.55 | 1034.85 | 3083 | 5510.7 |
| German_2023 | 24788.61 | 4 | 18558.7 | 1061.36 | 51 | 2897.09 |
| German_2023 | 4908.2 | 4 | 3097.95 | 219.5 | 45 | 392.38 |
| German_2023 | 36418.76 | 38.5 | 28205.06 | 738.7 | 848 | 1326.73 |
| German_2023 | 336160.49 | 3215 | 241235.79 | 3257 | 3436 | 22118.94 |
| German_2023 | 373036.73 | 4178.25 | 273339.27 | 2217.58 | 4768 | 13828.08 |
| German_2023 | 571709.69 | 1 | 313960.46 | 32155.4 | 31678 | 38120.16 |
| German_2023 | 262281.09 | 5113.25 | 148481.46 | 2066.75 | 1235 | 9631.9 |
| German_2023 | 98994.66 | 150.67 | 124477.86 | 519.92 | 697 | 4481.8 |
| German_2023 | 250510.86 | 52 | 181311.41 | 2421.4 | 7199 | 10140.65 |
| German_2023 | 359261.73 | 1467.5 | 234775.24 | 3772.4 | 8127 | 14055 |
| German_2023 | 282139.77 | 3406 | 198037.05 | 2151.5 | 4527 | 8627.2 |
| German_2023 | 196966.11 | 5.5 | 130103.23 | 3911.07 | 1517 | 6639.4 |
| German_2023 | 14111.8 | 1 | 13243.23 | 225 | 0 | 420.01 |
| German_2023 | 313163.84 | 0 | 192022.42 | 4525 | 2843 | 52820.17 |
| German_2023 | 162764.76 | 1 | 133876.51 | 1253.5 | 1818 | 3050.7 |
| German_2023 | 11805.98 | 0 | 9955.45 | 106 | 22 | 490.4 |
| German_2023 | 270668.28 | 322 | 188916.05 | 10759.3 | 7168 | 14307.2 |
| German_2023 | 235339.14 | 119.83 | 159732.73 | 12475.33 | 5 | 34187.23 |
| German_2023 | 259516.54 | 20.08 | 235995.41 | 2002.11 | 11 | 4846.21 |
| German_2023 | 253895.09 | 4.25 | 244046.88 | 1540.25 | 661 | 1666.75 |
| German_2023 | 233256.6 | 1 | 218581.12 | 774 | 920 | 1467.6 |
| German_2023 | 88199.37 | 6.75 | 77117.03 | 3899.32 | 0 | 4771.94 |
| German_2023 | 185706.33 | 2 | 173067.75 | 2101.03 | 0 | 8810.65 |
| German_2023 | 101874.68 | 0 | 82843.51 | 3479.33 | 370 | 5713.8 |
| German_2023 | 82399.71 | 12 | 76946.79 | 1803.67 | 188 | 2497.07 |
| German_2023 | 269944.91 | 386.5 | 191471.4 | 5869.64 | 1348 | 22932.12 |
| German_2023 | 13543.64 | 57.42 | 11296.13 | 47.25 | 114 | 569.36 |
| German_2023 | 11720.9 | 28.67 | 8301.95 | 15.67 | 61 | 236.72 |
| German_2023 | 79452.59 | 14.75 | 69105.59 | 443.75 | 906 | 808.5 |
| German_2023 | 139413.78 | 13.25 | 127657.21 | 767.25 | 428 | 5357.67 |
| German_2023 | 288673.88 | 0 | 182098.46 | 28459.2 | 3405 | 51391 |
| German_2023 | 210263.7 | 0 | 168528.79 | 7613.3 | 2115 | 13917.13 |
| German_2023 | 216601.99 | 8.83 | 159701.45 | 1641.36 | 2193 | 25505.12 |
| German_2023 | 170349.35 | 0 | 121301.85 | 397.67 | 4009 | 1736.57 |
| German_2023 | 180017.4 | 17.83 | 174505.27 | 999.33 | 0 | 1877.23 |
| German_2023 | 42723.46 | 0 | 32813.82 | 95.2 | 386 | 542.1 |
| German_2023 | 72073.94 | 2044 | 50520.62 | 584 | 648 | 2083.8 |
| German_2023 | 110291.31 | 34.47 | 86554.6 | 559.33 | 1214 | 9554.64 |
| German_2023 | 155573.45 | 0 | 143612.74 | 958.2 | 1222 | 1857.8 |
| German_2023 | 15766.22 | 1 | 9920.44 | 58 | 464 | 607.2 |
| German_2023 | 58785.03 | 9.25 | 50601.72 | 788.45 | 464 | 2772.75 |
| German_2023 | 29509.21 | 0 | 17458.54 | 545 | 1079 | 1588.5 |
| German_2023 | 103455.57 | 5.25 | 77128.96 | 717.5 | 1051 | 7399.9 |
| German_2023 | 79697.11 | 0 | 61916.69 | 1051.17 | 965 | 2523.37 |
| German_2023 | 289529.45 | 700 | 122710.46 | 26775.78 | 0 | 52481.93 |
| German_2023 | 128791.09 | 1.75 | 80940.04 | 13528.75 | 2 | 20215.43 |
| German_2023 | 79307.51 | 0.25 | 75973.51 | 255.55 | 0 | 1046.06 |
| German_2023 | 213494.96 | 121.96 | 276009.16 | 3393.42 | 2018 | 95513.72 |
| German_2023 | 158144.7 | 8 | 125729.09 | 1422.67 | 1391 | 5139.43 |
| German_2023 | 194658.05 | 0 | 303967.58 | 4677 | 367 | 73224.3 |
| German_2023 | 169754.36 | 1.67 | 278268.29 | 7618.27 | 3847 | 68676.07 |
| German_2023 | 240605.02 | 1975.5 | 291573.43 | 3173.5 | 743 | 51047.42 |
| German_2023 | 115337.1 | 20.25 | 172213.31 | 2350.54 | 509 | 45025.97 |
| German_2023 | 241451.47 | 1.25 | 228309.14 | 91.75 | 641 | 1850.8 |
| German_2023 | 160978.6 | 0 | 156879.81 | 43 | 323 | 284.3 |
| German_2023 | 144713.08 | 72 | 229519.11 | 4083.8 | 418 | 64571.64 |
| German_2023 | 41873.93 | 0 | 25765.49 | 1111 | 1017 | 3433.3 |
| German_2023 | 10328.84 | 0 | 4520.24 | 1 | 895 | 336.5 |
| German_2023 | 201411.37 | 0 | 127827.24 | 7126 | 7849 | 11877.14 |
| German_2023 | 355886.07 | 1 | 319164.41 | 335.64 | 830 | 1471.9 |
| German_2023 | 31808.32 | 2.75 | 27385.36 | 183.95 | 166 | 461.73 |
| German_2023 | 174457.71 | 8.5 | 243394.96 | 12771.86 | 855 | 70080.96 |
| German_2023 | 475550.42 | 3.67 | 466095.25 | 188.67 | 1162 | 545.7 |
| German_2023 | 83286.65 | 0 | 76633.64 | 404.17 | 416 | 844.07 |
| German_2023 | 24690.5 | 0 | 24690.05 | 0 | 13 | 145 |
| German_2023 | 894142.08 | 1 | 888614.07 | 183 | 468 | 1012.2 |
| German_2023 | 26065.6 | 1 | 25618.85 | 16 | 80 | 32.6 |
| German_2023 | 64319.23 | 14 | 61222.33 | 119 | 105 | 469.3 |
| German_2023 | 106876.21 | 0.33 | 106204.13 | 203.66 | 23 | 488.06 |
| German_2023 | 81525.89 | 0.67 | 78612 | 109.34 | 455 | 278.57 |
| German_2023 | 24084.57 | 0 | 20223.69 | 242.37 | 20 | 1934.79 |
| German_2023 | 388748.94 | 67 | 385591.88 | 183.33 | 123 | 253.16 |
| German_2023 | 205668.65 | 0 | 186302.97 | 807.67 | 701 | 3054.27 |
| German_2023 | 60309.95 | 0 | 59414.17 | 2 | 53 | 129.98 |
| German_2023 | 254157.12 | 0 | 224604.99 | 2878 | 1644 | 2839.64 |
| German_2023 | 95927.18 | 1 | 92400.18 | 38.5 | 161 | 227.1 |
| German_2023 | 212911.92 | 0 | 205051.21 | 511.37 | 377 | 1895.93 |
| German_2023 | 180853.52 | 38 | 174245.36 | 231.83 | 725 | 834.53 |
| German_2023 | 164170.27 | 0 | 145992.6 | 2429.67 | 625 | 4307.07 |
| German_2023 | 279293.74 | 0 | 257195.21 | 1805.6 | 787 | 2920.4 |
| German_2023 | 205081.13 | 0 | 189703.86 | 373.5 | 1342 | 1483.93 |
| German_2023 | 291482.17 | 27.75 | 262235.28 | 1361.25 | 3346 | 3396.6 |
| German_2023 | 199444.5 | 32.67 | 344457.15 | 6014.67 | 941 | 68990.33 |
| German_2023 | 4006.51 | 1 | 3269.29 | 94.44 | 0 | 385.9 |
| German_2023 | 9205.73 | 0.14 | 9009.31 | 122 | 0 | 305.84 |
| German_2023 | 188621.5 | 0 | 132769.08 | 2638 | 1782 | 3422.32 |
| German_2023 | 114621.84 | 17 | 102258.57 | 324.77 | 103 | 1049.07 |
| German_2023 | 83231.4 | 0 | 73761.98 | 437 | 255 | 1440.9 |
| German_2023 | 97344.54 | 0 | 87707.89 | 134.2 | 1043 | 474.85 |
| German_2023 | 59615.04 | 0 | 54347.13 | 144.5 | 188 | 489.7 |
| German_2023 | 93798.71 | 0 | 85852.78 | 240.2 | 757 | 1482 |
| German_2023 | 222418.56 | 0 | 214821.19 | 262 | 1088 | 1265.6 |
| German_2023 | 213901.58 | 0.33 | 191157.29 | 1435.33 | 0 | 2481.44 |
| German_2023 | 167948.35 | 0.14 | 150660.44 | 1489.67 | 0 | 3516.91 |
| German_2023 | 26375.34 | 1.75 | 20540.36 | 56.75 | 187 | 901.29 |
| German_2023 | 69543.94 | 0.25 | 63267.79 | 50.25 | 720 | 321.8 |
| German_2023 | 282657.2 | 2 | 279098.95 | 301.5 | 837 | 475.8 |
| German_2023 | 108644.24 | 283.29 | 167958.17 | 1521.6 | 155 | 41584.75 |
| German_2023 | 232520.07 | 0.14 | 227769.69 | 407 | 0 | 1370.74 |
| German_2023 | 80058.39 | 0 | 76766.14 | 300 | 0 | 1876.8 |
| German_2023 | 271174.75 | 0.33 | 266468.45 | 61.33 | 298 | 311.9 |
| German_2023 | 62843.52 | 1 | 59006.93 | 83.45 | 203 | 588.81 |
| German_2023 | 191748.92 | 20.67 | 184550.83 | 128.67 | 989 | 1235.23 |
| German_2023 | 20136.72 | 9 | 17969.22 | 44 | 122 | 346.5 |
| German_2023 | 281706.18 | 0 | 277379.44 | 773.9 | 0 | 1304.95 |
| German_2023 | 3994.9 | 3 | 2561.03 | 56.5 | 155 | 118.1 |
| German_2023 | 107291.7 | 11.25 | 98529.75 | 512.25 | 939 | 1332.25 |
| German_2023 | 142093.7 | 219.75 | 125280.44 | 100.45 | 2402 | 948.95 |
| German_2023 | 78257.27 | 0.83 | 77972.77 | 565.83 | 583 | 999.8 |
| German_2023 | 263450.96 | 345.25 | 240396.75 | 367.78 | 920 | 1166.55 |
| German_2023 | 118065.04 | 52.25 | 91089.77 | 1262.5 | 379 | 4844.35 |
| German_2023 | 183024.84 | 1504.67 | 130162.84 | 3167.67 | 4432 | 4622.17 |
| German_2023 | 146284.15 | 109 | 123404.86 | 560.2 | 1739 | 1185.38 |
| German_2023 | 102644.31 | 177 | 90939.57 | 3863.25 | 0 | 7234.68 |
| German_2023 | 159731.14 | 160.33 | 122026.3 | 960.89 | 428 | 4910.33 |
| German_2023 | 279857.95 | 210.75 | 225375.29 | 6014.75 | 7609 | 9648.12 |
| German_2023 | 231022.98 | 1 | 207183.58 | 1340.2 | 1379 | 3977.37 |
| German_2023 | 110769.37 | 0 | 77994.74 | 1425 | 3436 | 2214.8 |
| German_2023 | 244284.2 | 403.81 | 98080.45 | 4471.67 | 362 | 9843.74 |
| German_2023 | 288834.71 | 1 | 239542.16 | 4786.2 | 2940 | 9402 |
| German_2023 | 226057.28 | 2407.83 | 180822.19 | 1383.16 | 804 | 3641.03 |
| German_2023 | 278381.33 | 3.5 | 237728.38 | 9689.6 | 1521 | 10625.5 |
| German_2023 | 208271.6 | 445.83 | 195147.02 | 1419.53 | 431 | 2528.18 |
| German_2023 | 377713.59 | 349.5 | 167039.95 | 7090.5 | 15893 | 42863.78 |
| German_2023 | 271511.85 | 0 | 259892.92 | 71.7 | 603 | 1005.2 |
| German_2023 | 237366.78 | 0.25 | 236301.18 | 253.15 | 11 | 1912.3 |
| German_2023 | 113455.37 | 8.67 | 105087.97 | 303.67 | 358 | 719.17 |
| German_2023 | 167318.61 | 42 | 89802.32 | 1307.5 | 4788 | 12799.38 |
| German_2023 | 54177.17 | 9 | 44371.63 | 418.6 | 182 | 1691.94 |
| German_2023 | 274204.66 | 23.5 | 258069.41 | 2773 | 0 | 5746.05 |
| German_2023 | 4871.29 | 5.17 | 4023.93 | 79.34 | 4 | 496.82 |
| German_2023 | 206867.59 | 0 | 189172.73 | 879.67 | 1041.67 | 1441.67 |
| German_2023 | 285565.97 | 125 | 274513.63 | 1239 | 236 | 2265.43 |
| German_2023 | 3345.99 | 0 | 2753.84 | 96.5 | 0 | 334.5 |
| German_2023 | 69103.05 | 0.14 | 58842.17 | 1449.18 | 11 | 2376.2 |
| German_2023 | 225410.17 | 2 | 189869.09 | 1045 | 2266 | 1921.9 |
| German_2023 | 58294.77 | 59.25 | 46225.37 | 1322.45 | 1160 | 1784.85 |
| German_2023 | 26558.93 | 9 | 24134.78 | 158 | 213 | 353.6 |
| German_2023 | 90049.81 | 0.33 | 44131.29 | 4179.69 | 2 | 14515.5 |
| German_2023 | 35849.64 | 3.75 | 33985.32 | 108.75 | 210 | 245.82 |
| German_2023 | 116108.06 | 0 | 99084.73 | 244 | 1949 | 1585.4 |
| German_2023 | 72761.97 | 0 | 62183.92 | 1446 | 1292 | 2015.9 |
| German_2023 | 22487.49 | 0 | 13886.34 | 301.17 | 899 | 1060.2 |
| German_2023 | 88545.67 | 2666 | 71008.77 | 506 | 378 | 2460.9 |
| German_2023 | 70002.08 | 154 | 56116.1 | 405.58 | 1720 | 610.83 |
| German_2023 | 90001.9 | 0 | 82861.73 | 18 | 521 | 1119.9 |
| German_2023 | 132080.64 | 72.25 | 119649.05 | 119.65 | 1856 | 674.8 |
| German_2023 | 300206.57 | 60.25 | 157429.58 | 1113.08 | 206 | 3237.76 |
| German_2023 | 252354.8 | 76.83 | 191434.77 | 8218.63 | 202 | 18142.13 |
| German_2023 | 31979.45 | 33 | 27445.64 | 1243 | 109 | 2331.7 |
| German_2023 | 80002.86 | 1.25 | 66011.51 | 6.75 | 1115 | 385.3 |
| German_2023 | 172151.38 | 1981 | 146579.56 | 47 | 706 | 2789.7 |
| German_2023 | 236297.02 | 12.92 | 185900.65 | 9759.74 | 9 | 26132.28 |
| German_2023 | 159491.91 | 18.5 | 274386.64 | 3551.5 | 179 | 59754.02 |
| German_2023 | 128303.97 | 164.33 | 209558.8 | 2997.65 | 509 | 50461.83 |
| German_2023 | 204406.79 | 2617 | 276806.51 | 1881.67 | 1604 | 51964.25 |
| German_2023 | 198478.46 | 1923.33 | 258165.34 | 8434.47 | 1164 | 35645.28 |
| German_2023 | 145899.24 | 0.33 | 268872.09 | 2529.33 | 1169 | 47729.95 |
| German_2023 | 205490.85 | 17.76 | 240819.18 | 4169.53 | 555 | 73960.33 |
| German_2023 | 306662.35 | 45.63 | 331430.25 | 4193.32 | 648 | 123393.53 |
| German_2023 | 200045.07 | 0 | 247193.66 | 3298 | 218 | 55644.54 |
| German_2023 | 123103.6 | 34.42 | 186116.35 | 1342.06 | 419 | 37719.66 |
| German_2023 | 178485.97 | 42.5 | 228891.21 | 2125.5 | 576 | 51723.62 |
| German_2023 | 227037.12 | 20 | 295178.53 | 1572.67 | 1024 | 61470.11 |
| German_2023 | 152952.42 | 0.75 | 276364.71 | 2590 | 1425 | 43241.37 |
| German_2023 | 254483.59 | 137.5 | 262310.95 | 3408.5 | 415 | 43811.48 |
| German_2023 | 214049.73 | 31.33 | 254040.89 | 1742.33 | 334 | 46496.78 |
| German_2023 | 137621.73 | 10.21 | 153133.13 | 1178.68 | 238 | 32136.41 |
| German_2023 | 87330.99 | 74.56 | 125240.47 | 2220.53 | 159 | 35189.18 |
| German_2023 | 132486.51 | 4 | 91307.89 | 14186 | 623 | 19418.36 |
| German_2023 | 480408.73 | 0 | 467718 | 257 | 480 | 1017.8 |
| German_2023 | 325100.19 | 115.92 | 277448.49 | 762.75 | 114 | 9265.61 |
| German_2023 | 186626.95 | 173 | 158176.98 | 1466.73 | 20 | 4249.53 |
| German_2023 | 496625.1 | 6.43 | 443093.52 | 28753 | 366 | 27340.83 |
| German_2023 | 149478.52 | 1.25 | 146827.7 | 248.25 | 77 | 307.23 |
| German_2023 | 558985.32 | 45.25 | 725802.27 | 5612.25 | 381 | 138805.81 |
| German_2023 | 385855.7 | 187.25 | 368258.87 | 363.25 | 1431 | 1240.2 |
| German_2023 | 458361.8 | 3 | 451547.3 | 446 | 756 | 344.8 |
| German_2023 | 206833.18 | 264.58 | 168734.88 | 1429.5 | 1089 | 5979.22 |
| German_2023 | 247856.42 | 1 | 317893.52 | 3822.86 | 868 | 95304.08 |
| German_2023 | 304487.99 | 0 | 295009.67 | 506 | 1027 | 5403.83 |
| German_2023 | 306090.76 | 12.5 | 278498.83 | 1001 | 538 | 11778.7 |
| German_2023 | 10620.57 | 5.75 | 7799.39 | 210.62 | 117 | 611.67 |
| German_2023 | 371221.32 | 3.25 | 364092.83 | 261.25 | 440 | 601.73 |
| German_2023 | 237800.3 | 1 | 215265.23 | 3912.5 | 3352 | 9708.57 |
| German_2023 | 301493.22 | 39.5 | 290255.97 | 713 | 2244 | 1496.73 |
| German_2023 | 446728.19 | 0.58 | 438216.46 | 234.58 | 894 | 516.1 |
| German_2023 | 69971.29 | 0.25 | 40635.04 | 2999.25 | 29 | 4935.8 |
| German_2023 | 334033.61 | 0 | 312461.14 | 540 | 2820 | 1694.6 |
| German_2023 | 22320.1 | 0.14 | 15194.68 | 3346 | 0 | 4894.07 |
| German_2023 | 33380.02 | 0 | 18475.87 | 3378 | 285 | 7016.73 |
| German_2023 | 241819.94 | 0 | 208694.85 | 1141.2 | 3330 | 8848.5 |
| German_2023 | 286216.9 | 165 | 240219.23 | 2122.3 | 3715 | 4136.73 |
| German_2023 | 181236.16 | 58.43 | 281461.3 | 2598.04 | 258 | 71292.08 |
| German_2023 | 64118.62 | 0.25 | 56042.66 | 578.42 | 0 | 1764.07 |
| German_2023 | 157131.49 | 1 | 102700.37 | 1217.5 | 3944 | 4891.42 |
| German_2023 | 232502.29 | 0 | 195336.41 | 9104.87 | 3306 | 12889.9 |
| German_2023 | 493531.74 | 0.25 | 207401.43 | 9651.75 | 54676.33 | 49457.15 |
| German_2023 | 376457.43 | 1.58 | 365270.07 | 906.58 | 1315 | 1893.05 |
| German_2023 | 234170.34 | 1.25 | 228477.59 | 137.75 | 183 | 648.8 |
| German_2023 | 381928.23 | 24.25 | 304608.03 | 5241.25 | 3445 | 13100.51 |
| German_2023 | 173168.01 | 19 | 127905.62 | 1918.68 | 0 | 11121.6 |
| German_2023 | 172255.14 | 32.39 | 184964.97 | 3409.53 | 500 | 45756.96 |
| German_2023 | 61154.35 | 10.75 | 58296.07 | 80.35 | 167 | 852.92 |
| German_2023 | 330544.69 | 1068 | 159460.04 | 55306.17 | 5320 | 80210.03 |
| German_2023 | 19317.72 | 18 | 16149.56 | 297.83 | 248 | 518.58 |
| German_2023 | 20650.2 | 1047 | 13419.57 | 60 | 152 | 483.95 |
| German_2023 | 26431.11 | 0 | 20954.61 | 74 | 324 | 266.1 |
| German_2023 | 157148.75 | 3.61 | 228906.42 | 1373.25 | 171 | 41510.78 |
| German_2023 | 198664.9 | 1 | 238742.7 | 10021 | 4791 | 64463.45 |
| German_2023 | 12499.76 | 36 | 7532.32 | 136 | 127 | 426.8 |
| German_2023 | 196417.09 | 53.74 | 207249.26 | 2983.57 | 365 | 72830.65 |
| German_2023 | 21247.88 | 0 | 14158.36 | 89 | 393 | 1100.5 |
| German_2023 | 309812.78 | 0 | 126758.39 | 63994 | 1110 | 94163.2 |
| German_2023 | 253891.9 | 154.97 | 158159.55 | 24962.3 | 1535 | 35223.54 |
| German_2023 | 302498.7 | 1 | 167230.69 | 25483.67 | 1766 | 46018.2 |
| German_2023 | 59047.75 | 22.91 | 37817.38 | 1923.18 | 813.67 | 4983.11 |
| German_2023 | 192950.75 | 51.72 | 275964.87 | 2411.41 | 1366.33 | 91885.79 |
| German_2023 | 24955.71 | 0 | 14638.42 | 212 | 485 | 918.45 |
| German_2023 | 314438.63 | 48.25 | 206299.18 | 18292.42 | 2251 | 29686.42 |
| German_2023 | 141662.82 | 77.5 | 236164.81 | 3900.04 | 113 | 58093.65 |
| German_2023 | 310634.1 | 20150.29 | 232761.3 | 1671.5 | 731 | 32816.19 |
| German_2023 | 150660.12 | 0 | 72896.37 | 1514.67 | 6306 | 15988.09 |
| German_2023 | 39952.81 | 0.33 | 19009.82 | 409.33 | 221 | 1654.35 |
| German_2023 | 401134.94 | 0.14 | 283074.87 | 5180.67 | 9674 | 14774 |
| German_2023 | 216007.54 | 77.47 | 137031.91 | 14682.2 | 933 | 20275.52 |
| German_2023 | 29075.73 | 348 | 19943.63 | 129 | 704 | 555.75 |
| German_2023 | 65459.12 | 0 | 54488.94 | 6 | 650 | 657.45 |
| German_2023 | 164653.6 | 46.33 | 267101.02 | 3219.51 | 139 | 67801.67 |
| German_2023 | 231445.85 | 0 | 218016.29 | 266.5 | 1695 | 780.3 |
| German_2023 | 37726.58 | 0 | 28648.08 | 1 | 1033 | 504.2 |
| German_2023 | 299391.74 | 141.17 | 361011.32 | 2827.42 | 392 | 77644.35 |
| German_2023 | 123512.31 | 0 | 68108.39 | 904.5 | 916 | 8301.98 |
| German_2023 | 156185.29 | 0 | 140573.1 | 249 | 1347 | 3061.43 |
| German_2023 | 856912.96 | 1 | 825714.2 | 2544.5 | 2879 | 5561.2 |
| German_2023 | 227569.58 | 0 | 221703.57 | 229 | 428 | 553.03 |
| German_2023 | 83459.66 | 8 | 74702.17 | 12.5 | 1396 | 373.9 |
| German_2023 | 190493.32 | 0 | 150815.92 | 411.67 | 1119 | 867.97 |
| German_2023 | 284945.61 | 37.79 | 278981.78 | 102.5 | 944 | 379.38 |
| German_2023 | 244385.15 | 0 | 238259.57 | 172 | 869 | 336.5 |
| German_2023 | 269960.03 | 0 | 263684.3 | 297.83 | 674 | 683.13 |
| German_2023 | 298130.34 | 0 | 288501.64 | 135.5 | 777 | 580.9 |
| German_2023 | 293161.79 | 15 | 289210.66 | 88.33 | 325 | 345.83 |
| German_2023 | 138316.34 | 18.33 | 119802.56 | 5598.38 | 0 | 18829.3 |
| German_2023 | 174776.25 | 1767 | 330388.75 | 3211.66 | 918 | 69446.74 |
| German_2023 | 287807.75 | 0 | 282459.93 | 1.5 | 385 | 849.81 |
| German_2023 | 41552.42 | 3.68 | 38856.19 | 129.25 | 138 | 481.43 |
| German_2023 | 107849.42 | 0.25 | 94813.08 | 634.25 | 647 | 1082.63 |
| German_2023 | 195144.69 | 56.86 | 237909.63 | 3451.4 | 196 | 68649.31 |
| German_2023 | 54759.54 | 5 | 48965.29 | 324 | 709 | 748.6 |
| German_2023 | 151940.53 | 9.25 | 141955.26 | 105.25 | 408 | 997.83 |
| German_2023 | 127433.64 | 0 | 98544.9 | 12974 | 0 | 16036.49 |
| German_2023 | 207539.5 | 0.75 | 185055.86 | 934.5 | 1045 | 1858.05 |
| German_2023 | 54075.43 | 0 | 57476.6 | 2553.33 | 274 | 6797.46 |
| German_2023 | 144307.79 | 510 | 125525.71 | 526 | 622 | 2230.3 |
| German_2023 | 159525.5 | 0 | 65554.3 | 2058.7 | 883 | 14154.19 |
| German_2023 | 19066.5 | 0 | 18938.43 | 357 | 0 | 1022.14 |
| German_2023 | 21321.06 | 9 | 15085.06 | 230 | 193 | 1519.73 |
| German_2023 | 273882.72 | 0 | 246659.66 | 419 | 2411 | 2852.88 |
| German_2023 | 264177.2 | 8 | 237213 | 955.4 | 1634 | 7503.46 |
| German_2023 | 188307.53 | 0.75 | 180037.72 | 475.75 | 963 | 776.13 |
| German_2023 | 101766.68 | 522 | 63730.29 | 495.83 | 3689 | 224.36 |
| German_2023 | 155914.69 | 1 | 77837.46 | 2347.37 | 4226 | 10896.31 |
| German_2023 | 261574.18 | 0 | 214590.58 | 162.33 | 2955 | 2388.63 |
| German_2023 | 172952.3 | 0 | 165658.75 | 532 | 571 | 1384.2 |
| German_2023 | 192859.85 | 1 | 185871.67 | 363.9 | 950 | 964.55 |
| German_2023 | 166072.2 | 98 | 303244.2 | 5034.4 | 1307 | 66337.85 |
| German_2023 | 378395.61 | 78.33 | 369252.19 | 1218.33 | 944 | 2236.41 |
| German_2023 | 272773.14 | 0 | 261930.39 | 628 | 646 | 737.34 |
| German_2023 | 180156.63 | 1 | 161495.92 | 619 | 2094 | 1140.6 |
| German_2023 | 239807.34 | 3 | 231737.59 | 1481 | 830 | 954.63 |
| German_2023 | 214275.72 | 0 | 194626.15 | 358 | 1253 | 640.7 |
| German_2023 | 171225.94 | 39.51 | 262610.15 | 3421.76 | 196 | 56336.1 |
| German_2023 | 158865.88 | 0 | 118258.55 | 3718.5 | 0 | 7788.63 |
| German_2023 | 240382.78 | 0 | 216584.36 | 1900 | 2813 | 2054.91 |
| German_2023 | 286307.97 | 2 | 278920.27 | 450.8 | 1160 | 887.8 |
| German_2023 | 276216.95 | 0 | 265753.73 | 14.3 | 1234 | 208.2 |
| German_2023 | 148568.26 | 12 | 110931.15 | 1279.03 | 983 | 5766.86 |
| German_2023 | 199374.55 | 2 | 195205.81 | 267 | 705 | 514.2 |
| German_2023 | 229100 | 2 | 214303.34 | 1064.83 | 665 | 3216.18 |
| German_2023 | 124504.95 | 5 | 57029.39 | 5108.66 | 7475.33 | 3959.09 |
| German_2023 | 334464.43 | 0 | 323998.93 | 473.33 | 933 | 822.76 |
| German_2023 | 73556.88 | 0.5 | 37302.71 | 7398 | 0 | 7994.24 |
| German_2023 | 125905.51 | 102.5 | 122190.37 | 21.5 | 414 | 319.15 |
| German_2023 | 74957.41 | 0 | 52313.02 | 1086.2 | 3228 | 1682.2 |
| German_2023 | 407501.92 | 5 | 400842.34 | 1013.5 | 1043 | 892.06 |
| German_2023 | 264257.93 | 1 | 259374.76 | 1059 | 400 | 1117.9 |
| German_2023 | 96359.08 | 285.29 | 55063.73 | 1045.2 | 1132 | 4835.39 |
| German_2023 | 130087.68 | 1.75 | 52714.72 | 129.25 | 398 | 5273.88 |
| German_2023 | 10382.59 | 3 | 7708.49 | 156.67 | 221 | 448.67 |
| German_2023 | 269851.52 | 0 | 253118.17 | 1403.67 | 590 | 2903.69 |
| German_2023 | 345891.65 | 11 | 65329.09 | 1908.67 | 5688.67 | 2078.15 |
| German_2023 | 463084.9 | 6.42 | 434701.19 | 178.92 | 268 | 614.5 |
| German_2023 | 313554.13 | 16 | 290006.29 | 435.33 | 730 | 1640.63 |
| German_2023 | 258799.25 | 11 | 253974.17 | 147 | 845 | 644.03 |
| German_2023 | 426865.35 | 28.83 | 413686.8 | 537.36 | 1439 | 1526.43 |
| German_2023 | 516622.96 | 131 | 498087.78 | 2123 | 1969 | 4906.89 |
| German_2023 | 72569.74 | 0.25 | 60729.2 | 1207.75 | 0 | 2735.19 |
| German_2023 | 37234.47 | 80.67 | 32366.68 | 543.67 | 481 | 952.3 |
| German_2023 | 18361.61 | 0 | 16247.62 | 403.5 | 39 | 588.6 |
| German_2023 | 172118.95 | 0 | 229916.55 | 2708 | 488 | 44648.47 |
| German_2023 | 324878.96 | 890 | 311666.18 | 831.5 | 819 | 2342.61 |
| German_2023 | 373281.44 | 11.81 | 338645.55 | 2473.75 | 7114 | 10862.98 |
| German_2023 | 207459.46 | 0 | 183394.99 | 3359.2 | 1125 | 3414.02 |
| German_2023 | 242322.46 | 0 | 240133.51 | 77.5 | 94 | 246.6 |
| German_2023 | 156229.01 | 5399.25 | 131525.37 | 193.08 | 159 | 1343.33 |
| German_2023 | 221680.86 | 108.25 | 214590.87 | 251.75 | 204 | 1362.8 |
| German_2023 | 145857.25 | 1.33 | 126758.03 | 3869.83 | 0 | 5389.8 |
| German_2023 | 260177.62 | 6.33 | 244161.93 | 513.73 | 352 | 3734.98 |
| German_2023 | 148188.32 | 12 | 143243.9 | 217 | 500 | 694.83 |
| German_2023 | 266074.46 | 51.25 | 257365.09 | 249.25 | 862 | 1148.4 |
| German_2023 | 116757.47 | 33.54 | 31949.69 | 2156.41 | 0 | 2328.45 |
| German_2023 | 308724.8 | 18 | 300671.2 | 167.4 | 865 | 451.3 |
| German_2023 | 271494.1 | 0 | 260476.49 | 543 | 515 | 1867.13 |
| German_2023 | 178841.11 | 7 | 173074.45 | 512.4 | 769 | 1516.3 |
| German_2023 | 98105.07 | 2 | 81264.54 | 1009.5 | 899 | 3036.8 |
| German_2023 | 34152.65 | 0.25 | 31532.17 | 680.75 | 17 | 1086.3 |
| German_2023 | 251515.14 | 76 | 224647.29 | 1257.5 | 865 | 3038.35 |
| German_2023 | 233367.2 | 38.5 | 222379.28 | 555.5 | 678 | 833.07 |
| German_2023 | 123289.88 | 93 | 52850.08 | 13247.94 | 0 | 21645.03 |
| German_2023 | 211340.45 | 12.25 | 298755.12 | 3876.05 | 1048 | 98343.7 |
| German_2023 | 320744.28 | 0 | 295973.5 | 4172 | 2531 | 3384.06 |
| German_2023 | 330938.07 | 13.33 | 319512.21 | 588.33 | 647 | 1591.94 |
| German_2023 | 93586.54 | 153 | 85941.06 | 865 | 232 | 2244.9 |
| German_2023 | 162172.44 | 15 | 136295.88 | 1511.67 | 1634 | 2372.47 |
| German_2023 | 68725.93 | 72.92 | 56463.65 | 208.75 | 156 | 1542.48 |
| German_2023 | 216905.92 | 1576 | 146735.45 | 890.67 | 670 | 10069.97 |
| German_2023 | 234826.87 | 3734.33 | 341431.4 | 2531.33 | 1660 | 59755.15 |
| German_2023 | 105987.15 | 0 | 76917.65 | 1887 | 3331 | 13121.4 |
