# Supplementary material for: Microbial diagnostic features identified across populations possess potential antitumor properties in breast cancer
Source: mSystems. 2025 Jun 23;10(7):e00271-25. doi: 10.1128/msystems.00271-25 (PMC12282184; doi:10.1128/msystems.00271-25)
Supplement: Table S10 — 16S rRNA sequencing result of C. acnes strains. [file msystems.00271-25-s0010.doc]

**Table S10. 16S rRNA sequencing result of *C. acnes* strains.**

| ***C. acnes* strain** | **16S rRNA sequencing results** |
| --- | --- |
| strain_A | CAGTCGAACGGAAAGGCCCTGCTTTTGTGGGGTGCTCGAGTGGCGAACGGGTGAGTAACACGTGAGTAACCTGCCCTTGACTTTGGGATAACTTCAGGAAACTGGGGCTAATACCGGATAGGAGCTCCTGCTGCATGGTGGGGGTTGGAAAGTTTCGGCGGTTGGGGATGGACTCGCGGCTTATCAGCTTGTTGGTGGGGTAGTGGCTTACCAAGGCTTTGACGGGTAGCCGGCCTGAGAGGGTGACCGGCCACATTGGGACTGAGATACGGCCCAGACTCCTACGGGAGGCAGCAGTGGGGAATATTGCACAATGGGTGGAAGCCTGATGCAGCAACGCCGCGTGCGGGATGACGGCCTTCGGGTTGTAAACCGCTTTCGCCTGTGACGAAGCGTGAGTGACGGTAATGGGTAAAGAAGCACCGGCTAACTACGTGCCAGCAGCCGCGGTGATACGTAGGGTGCGAGCGTTGTCCGGATTTATTGGGCGTAAAGGGCTCGTAGGTGGTTGATCGCGTCGGAAGTGTAATCTTGGGGCTTAACCCTGAGCGTGCTTTCGATACGGGTTGACTTGAGGAAGGTAGGGGAGAATGGAATTCCTGGTGGAGCGGTGGAATGCGCAGATATCAGGAGGAACACCAGTGGCGAAGGCGGTTCTCTGGGCCTTTCCTGACGCTGAGGAGCGAAAGCGTGGGGAGCGAACAGGCTTAGATACCCTGGTAGTCCACGCTGTAAACGGTGGGTACTAGGTGTGGGGTCCATTCCACGGGTTCCGTGCCGTAGCTAACGCTTTAAGTACCCCGCCTGGGGAGTACGGCCGCAAGGCTAAAACTCAAAGGAATTGACGGGGCCCCGCACAAGCGGCGGAGCATGCGGATTAATTCGATGCAACGCGTAGAACCTTACCTGGGTTTGACATGGATCGGGAGTGCTCAGAGATGGGTGTGCCTCTTTTGGGGTCGGTTCACAGGTGGTGCATGGCTGTCGTCAGCTCGTGTCGTGAGATGTTGGGTTAAGTCCCGCAACGAGCGCAACCCTTGTTCACTGTTGCCAGCACGTTATGGTGGGGACTCAGTGGAGACCGCCGGGGTCAACTCGGAGGAAGGTGGGGATGACGTCAAGTCATCATGCCCCTTATGTCCAGGGCTTCACGCATGCTACAATGGCTGGTACAGAGAGTGGCGAGCCTGTGAGGGTGAGCGAATCTCGGAAAGCCGGTCTCAGTTCGGATTGGGGTCTGCAACTCGACCTCATGAAGTCGGAGTCGCTAGTAATCGCAGATCAGCAACGCTGCGGTGAATACGTTCCCGGGGCTTGTACACACCGCCCGTCAAGTCATGAAAGTTGGTAACACCCGAAGCCGGTGGCCTAACCGTTGTGGG |
| strain_B | AGTCGAACGGAAAGGCCCTGCTTTTGTGGGGTGCTCGAGTGGCGAACGGGTGAGTAACACGTGAGTAACCTGCCCTTGACTTTGGGATAACTTCAGGAAACTGGGGCTAATACCGGATAGGAGCTCCTGCTGCATGGTGGGGGTTGGAAAGTTTCGGCGGTTGGGGATGGACTCGCGGCTTATCAGCTTGTTGGTGGGGTAGTGGCTTACCAAGGCTTTGACGGGTAGCCGGCCTGAGAGGGTGACCGGCCACATTGGGACTGAGATACGGCCCAGACTCCTACGGGAGGCAGCAGTGGGGAATATTGCACAATGGGCGGAAGCCTGATGCAGCAACGCCGCGTGCGGGATGACGGCCTTCGGGTTGTAAACCGCTTTCGCCTGTGACGAAGCGTGAGTGACGGTAATGGGTAAAGAAGCACCGGCTAACTACGTGCCAGCAGCCGCGGTGATACGTAGGGTGCGAGCGTTGTCCGGATTTATTGGGCGTAAAGGGCTCGTAGGTGGTTGATCGCGTCGGAAGTGTAATCTTGGGGCTTAACCCTGAGCGTGCTTTCGATACGGGTTGACTTGAGGAAGGTAGGGGAGAATGGAATTCCTGGTGGAGCGGTGGAATGCGCAGATATCAGGAGGAACACCAGTGGCGAAGGCGGTTCTCTGGGCCTTTCCTGACGCTGAGGAGCGAAAGCGTGGGGAGCGAACAGGCTTAGATACCCTGGTAGTCCACGCTGTAAACGGTGGGTACTAGGTGTGGGGTCCATTCCACGGGTTCCGTGCCGTAGCTAACGCTTTAAGTACCCCGCCTGGGGAGTACGGCCGCAAGGCTAAAACTCAAAGGAATTGACGGGGCCCCGCACAAGCGGCGGAGCATGCGGATTAATTCGATGCAACGCGTAGAACCTTACCTGGGTTTGACATGGATCGGGAGTGCTCAGAGATGGGTGTGCCTCTTTTGGGGTCGGTTCACAGGTGGTGCATGGCTGTCGTCAGCTCGTGTCGTGAGATGTTGGGTTAAGTCCCGCAACGAGCGCAACCCTTGTTCACTGTTGCCAGCACGTTATGGTGGGGACTCAGTGGAGACCGCCGGGGTCAACTCGGAGGAAGGTGGGGATGACGTCAAGTCATCATGCCCCTTATGTCCAGGGCTTCACGCATGCTACAATGGCTGGTACAGAGAGTGGCGAGCCTGTGAGGGTGAGCGAATCTCGGAA  AGCCGGTCTCAGTTCGGATTGGGGTCTGCAACTCGACCTCATGAAGTCGGAGTCGCTAGTAATCGCAGATCAGCAACGCTGCGGTGAATACGTTCCCGGGGCTTGTACACACCGCCCGTCAAGTCATGAAAGTTGGTAACACCCGAAGCCGGTGGCCTAACCGTTGTGGGGGA |
